# Discovery of potent thrombin inhibitors from a protease-focused DNA-encoded chemical library

**DOI:** 10.1073/pnas.2005447117

**Published:** 2020-07-08

**Authors:** Surendra Dawadi, Nicholas Simmons, Gabriella Miklossy, Kurt M. Bohren, John C. Faver, Melek Nihan Ucisik, Pranavanand Nyshadham, Zhifeng Yu, Martin M. Matzuk

**Affiliations:** ^a^Center for Drug Discovery, Department of Pathology & Immunology, Baylor College of Medicine, Houston, TX 77030

**Keywords:** DNA-encoded chemical library, focused DECL, protease inhibitor, COVID-19, SARS-CoV-2

## Abstract

DNA-encoded chemical libraries are collections of compounds individually coupled to unique DNA tags serving as amplifiable identification barcodes. By bridging split-and-pool combinatorial synthesis with the ligation of unique encoding DNA oligomers, million- to billion-member libraries can be synthesized for use in hundreds of healthcare target screens. Although structural diversity and desirable molecular property ranges generally guide DNA-encoded chemical library design, recent reports have highlighted the utility of focused DNA-encoded chemical libraries that are structurally biased for a class of protein targets. Herein, a protease-focused DNA-encoded chemical library was designed that utilizes chemotypes known to engage conserved catalytic protease residues. The three-cycle library features functional moieties such as guanidine, which interacts strongly with aspartate of the protease catalytic triad, as well as mild electrophiles such as sulfonamide, urea, and carbamate. We developed a DNA-compatible method for guanidinylation of amines and reduction of nitriles. Employing these optimized reactions, we constructed a 9.8-million-membered DNA-encoded chemical library. Affinity selection of the library with thrombin, a common protease, revealed a number of enriched features which ultimately led to the discovery of a 1 nM inhibitor of thrombin. Thus, structurally focused DNA-encoded chemical libraries have tremendous potential to find clinically useful high-affinity hits for the rapid discovery of drugs for targets (e.g., proteases) with essential functions in infectious diseases (e.g., severe acute respiratory syndrome coronavirus 2) and relevant healthcare conditions (e.g., male contraception).

## Significance

To rapidly identify small-molecule lead compounds to target healthcare-associated proteases, we constructed a unique 9.8-million-membered protease-focused DNA-encoded chemical library. Affinity selection of this library with a healthcare-relevant protease (i.e., thrombin, a key protein necessary for blood coagulation) revealed potent inhibitors in the first screening attempt. Our results emphasize the utility of a structurally focused DNA-encoded chemical library approach to rapidly uncover hits for healthcare targets (e.g., proteases) where no drug exists (e.g., male contraception) and for emerging diseases (e.g., coronavirus disease 2019).

DNA-encoded chemical libraries (DECLs) are a powerful tool for target-directed hit discovery (1–4). By bridging split-and-pool combinatorial synthesis approaches with the ligation of unique encoding DNA oligomers, million- to billion-member libraries may be synthesized at micromole scales for use in hundreds of target screens. Although DECLs are often built as a general screening resource and the significant cost and reagent savings theoretically enable routine screens of an organization’s entire DECL collection for any compatible target, in certain cases it is desirable to design and use focused DECLs for a specific target. These focused libraries are particularly suitable for targets in which mechanistic details, structural information, or relevant chemical matter are well-known (5, 6). Use of focused DECLs may bolster chances for robust hit generation as well as enable deeper and broader exploration of structure–activity features if combined with curated building block selection and deep sequencing. Focused DECLs may also empower designs with motifs not normally included in general DECLs such as DECLs with covalent modifiers (7) and DECLs incorporating macrocycles (8) and complex natural products (9).

Proteases, enzymes which catalyze the hydrolysis of peptidic bonds in proteins, have been an intensely studied class of targets for drug discovery due to their involvement in various pathophysiological processes (e.g., protein degradation, protein processing, and signal transduction pathways). Mechanistically, proteases generally fall into five classes: cysteine proteases, serine proteases, metalloproteases, threonine proteases, and aspartic proteases (10). After activation of the amide with key residues, three of the classes—cysteine, serine, and threonine—utilize the namesake residue to attack the amide carbonyl group, where metalloproteases and aspartic proteases use an activated water. Strategies to directly target proteases with small molecules have generally focused on engaging the active-site catalytic triad, leading to peptidomimetic inhibitors that either react with a nucleophilic residue in a covalent fashion or interact with the critical catalytic residues (11). This strategy has been particularly successful for inhibitors of serine proteases, which are designed to reveal a mild electrophile such as sulfonamide to interact with the serine hydroxyl group and a positively charged guanidine/benzamidine to interact with a nearby aspartic acid carboxylate (12). Clinically, there have been numerous successes including angiotensin-converting enzyme inhibitors for cardiovascular disorders (13), thrombin inhibitors for thromboembolism and bleeding disorders (14, 15), and HIV protease inhibitors in the treatment of HIV and AIDS (16), among others (17, 18). Recently, viral main protease (M^pro^, a cysteine protease) has presented as an attractive drug target for the development of novel therapeutics in the COVID-19 (coronavirus disease 2019) pandemic caused by severe acute respiratory syndrome coronavirus 2 (SARS-CoV-2), and a number of broad-spectrum inhibitors have been developed that target this main protease (19). Another target in the COVID-19 pandemic is human transmembrane serine protease 2 (TMPRSS2), which functions in the processing of the SARS-CoV-2 spike (S) protein (20). Proteases also play important roles in reproduction, and several serine proteases and serine protease-like proteins are essential for normal fertility and therefore could be unique targets for nonhormonal male contraception (i.e., a male pill). For example, among the proteins essential for male fertility are the following: TMPRSS12, a testis-specific transmembrane serine protease required for sperm motility and uterotubal junction migration (21) and structurally similar to TMPRSS2; serine protease 55 (PRSS55), a glycosylphosphatidylinositol-anchored testis-specific serine protease that is necessary for sperm uterotubal junction migration and sperm–oocyte binding (22, 23); serine protease 37 (PRSS37), a testis-specific inactive serine protease-like protein required for sperm uterotubal junction migration and fertilization (24); and ovochymase 2 (OVCH2), an epididymis-specific secreted serine protease required for maturation of sperm (25). Thus, there are multiple healthcare areas that could benefit from advances in the production of unique protease-biased chemical matter for inhibiting this important target class.

**Fig. 1.**
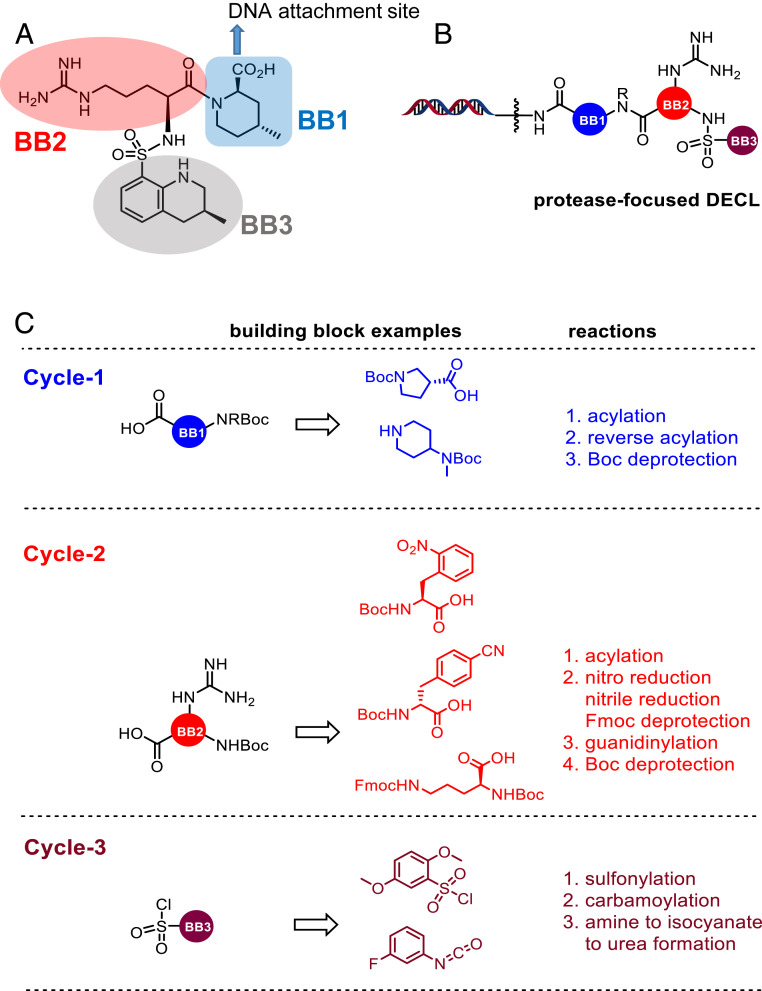
Protease-focused DECL design. (*A*) Structure of argatroban, a thrombin inhibitor in clinical use. Argatroban served as a prototype for the design of the protease-focused DECL described in this study. (*B*) Design of a guanidine containing three-cycle protease-focused DECL. The library features guanidine, as well as a sulfonamide, urea, and carbamate moiety, which are commonly found in protease inhibitors. (*C*) Examples of various types of building blocks in each cycle of the library build and a list of reactions required to accomplish the synthesis of this library.

Here, we report on a protease-focused DECL and DECL screening strategies designed to engage the protease catalytic triad. Since many of the known protease inhibitors contain guanidine, sulfonamide, urea, and carbamate moieties, we incorporated these functional groups into the design of this library (26, 27). We utilized the structure of argatroban (Fig. 1*A*), an arginine containing peptidomimetic thrombin inhibitor, as inspiration for the design of this library since it bears a positively charged guanidinium group flanked by two hydrophobic groups (28). Retrosynthetically, argatroban could be portioned into sections that could be connected in a three-cycle DECL. The piperidine carboxylic acid can be viewed as building block 1 (BB1) which can be covalently linked to DNA via an amide linker. Interestingly, this carboxylate is situated in the direction to open space of the thrombin binding pocket (29), which makes it correctly positioned for a DNA linkage site. The arginine moiety of argatroban can be visualized as BB2 and the aryl sulfonamide as BB3. Thus, the proposed protease DECL (Fig. 1*B*) could feature a BB1 set of various –*N*Boc amino acids and –*N*Boc diamines connected by amide bonds to a DNA-attached amino or carboxyl terminated linker, respectively. After –*N*Boc deprotection, a variety of different trifunctional cores containing a free carboxyl group, primary –*N*HBoc amine and orthogonally masked amine (–*N*Fmoc, nitro, or nitrile group) could be connected by amidation to serve as BB2. After deprotection of the orthogonally masked Fmoc group, and reduction of nitro or nitrile groups and subsequent guanidinylation, the remaining –*N*HBoc amine could be deprotected and functionalized with BB3 through sulfonylation, carbamolyation, and carbamylation (Fig. 1*C*). Critical to this design is both the synthesis and maintenance of the guanidine moiety through multistep DECL synthesis. However, to the best of our knowledge there have been no reports of guanidines within published DECLs, although a benzamidine-containing dual pharmacophore DECL has been reported (30).

## Results and Discussion

To evaluate the synthetic feasibility of this DECL design, we looked at the availability of DNA-compatible chemical reactions for the construction of this library (Fig. 1*C*). The acylation reaction, where an amine functionalized DNA is coupled with a carboxylic acid building block, or vice versa, forming an amide bond is one of most common reactions in DECL technology. Similarly, reports of deprotection conditions of –*N*Boc and –*N*Fmoc groups are also common. The reactions of an amine with electrophiles such as sulfonyl chlorides and isocyanates to produce sulfonamides and ureas, respectively, are also known (31). Several conditions for the reduction of aromatic nitro groups to the amine have also been reported (32, 33) Although the conversion of an amine to an isocyanate followed by reaction with nucleophiles such as amine and phenol to form urea and carbamate (34) is known in the presence of DNA, it has not been utilized in the construction of a reported DECL. Overall two of our planned reactions, the conversion of amine to guanidine and the reduction of nitriles to amines, would require optimization to find a general, DNA-compatible process.

### Optimization of DNA-Compatible Guanidinylation of Amines and Reduction of Nitriles.

**Fig. 2.**
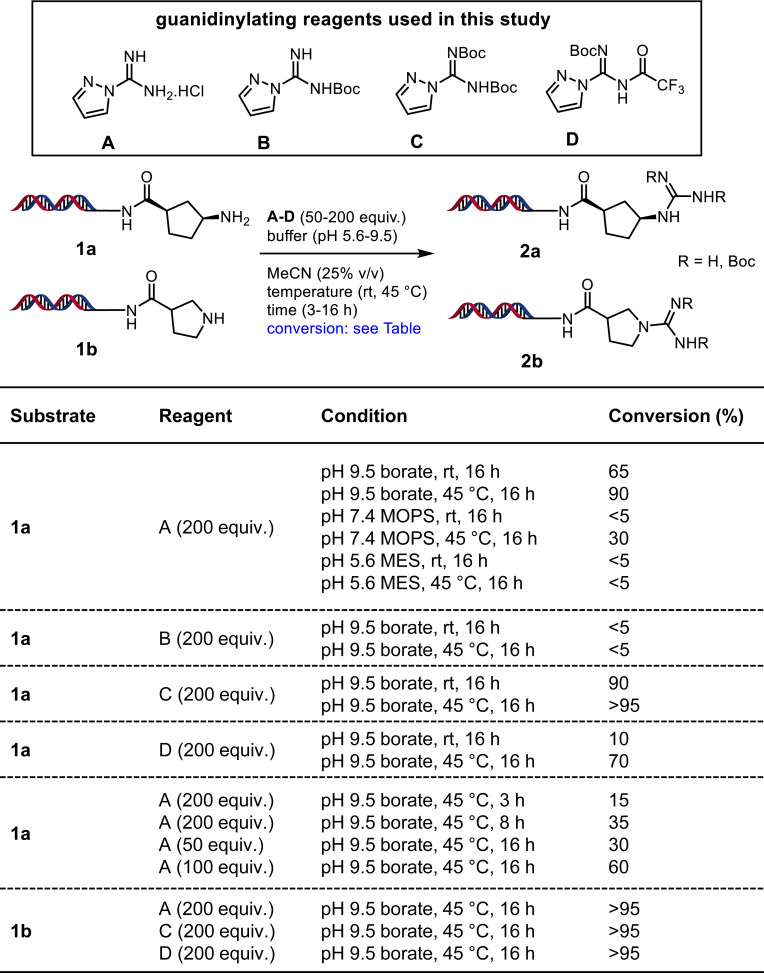
Optimization of reaction conditions for guanidinylation of amines.

**Fig. 3.**
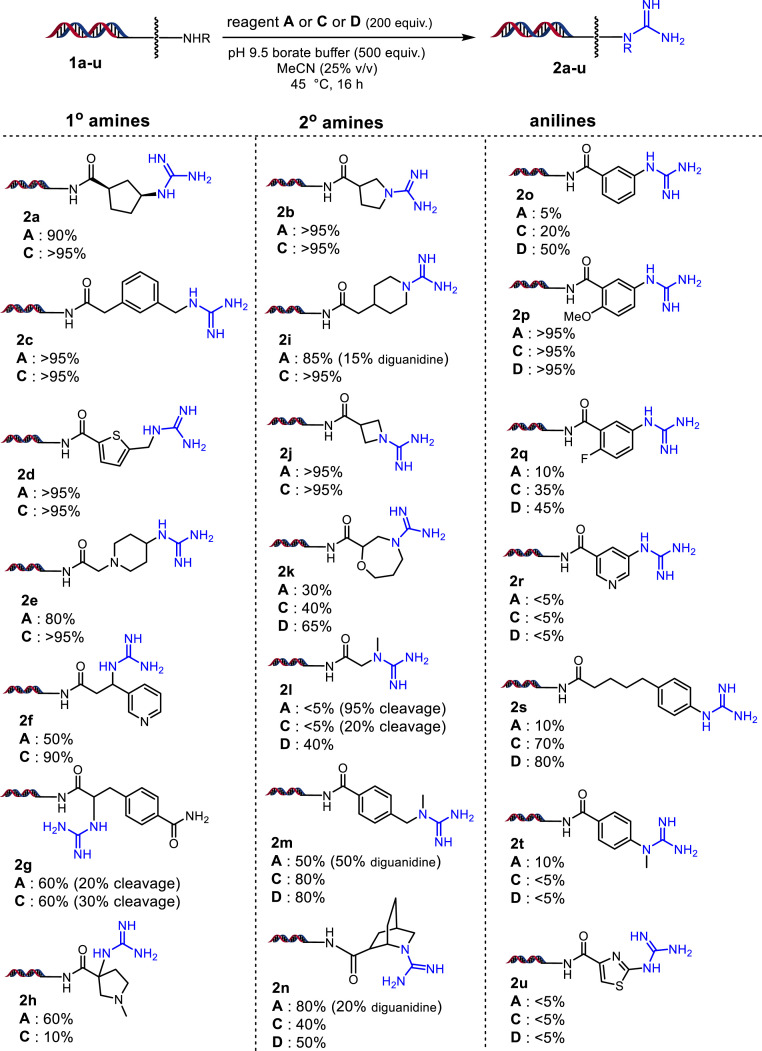
Scope of DNA-compatible guanidinylation of amines and anilines.

To explore the DNA-compatible guanidine formation, we studied four commercially available 1*H*-pyrazole-1-carboxamidine–based guanidinylation reagents A through D (Fig. 2) (35). The DNA linked primary and secondary amine substrates **1a** and **1b** were prepared from the DNA starting material DNA headpiece (DNA-HP **3**; see *SI Appendix*, Scheme S1) by acylation with corresponding –*N*HBoc amino acid followed by the –*N*Boc deprotection (*SI Appendix*, Fig. S2). When **1a** and **1b** were treated with reagents A through D in various reaction conditions (Fig. 2 and see *SI Appendix* for detailed optimization conditions), we observed formation of guanidine products **2a** and **2b**. Among different buffer systems (pH range from 5.6 to 9.9) studied, pH 9.5 borate buffer gave better conversions. Warming the reaction to 45 °C gave higher conversions in a shorter time. Overall, we determined that 45 °C for 16 h (overnight) in pH 9.5 borate buffer (25% vol/vol MeCN) with either reagent A or C gave excellent conversions with substrate **1a**, whereas substrate **1b** provided excellent conversions with reagents A, C, and D. We employed these optimal conditions to study the substrate scope of guanidinylation. The amines **1c**–**n** and anilines **1o**–**u** are obtained from acylation of DNA-HP **3** with commercially available –*N*Boc amino acids followed by –*N*Boc deprotection (*SI Appendix*, Fig. S2). We performed the guanidinylation of these substrates and the products **2a**–**u** were obtained in various conversions (Fig. 3). Primary and unhindered secondary amines readily undergo guanidinylation, whereas anilines have a wide range of reactivity depending upon electronics and sterics. With some secondary amines such as **1k** and most anilines, reagent D gave significantly better conversions. Regardless of the reagent and conditions used, the electron-deficient anilines such as **1q**, **1r**, and **1u** are poor substrates for guanidine formation, which is also observed with the secondary aniline **2t**. Importantly, in the library synthesis setting, we avoided using such building blocks containing electron-deficient anilines in cycle-2 chemistry. Overall, this result suggested that we should use reagent D for guanidinylation of anilines and some secondary amines whereas reagents A and C are more preferable for the guanidinylation of reactive amines.

Next, we studied the reduction of nitriles (see *SI Appendix* for detail optimization condition). Among several conditions studied, the best outcomes were observed while utilizing NaBH_4_ as hydride donor in the presence of following additives: Raney Ni and NiCl_2_ (*SI Appendix*, Table S2). A mixture of Raney Ni and NaBH_4_ in 40% MeOH in pH 5.6 MES [2-(*N*-morpholino)ethanesulfonic acid] buffer reduced both aromatic and aliphatic nitrile groups with excellent conversions within 1 h at room temperature. Also, Raney Ni is known to be compatible with DNA and has been previously employed for nitro reduction (34). One important consideration provided while using Raney Ni was the removal of metal particles by centrifugation before EtOH precipitation of DNA material to prevent DNA damage in subsequent step especially if it is the –*N*Boc deprotection.

### Chemistry Validation and Library Construction.

After optimization of the guanidinylation and nitrile reduction, we performed the validation of these two new reactions by the synthesis and analysis of three authentic on-DNA compounds **41**–**43** (*SI Appendix*, Scheme S2). A more detailed three-cycle chemistry validation process that mimics the library design was performed to prove the feasibility and versatility of our synthetic methods (*SI Appendix*, Scheme S3). Importantly, we need to confirm that the guanidine moiety formed in cycle-2 does not impact cycle-3 amine capping reactions. The validation process started with compound **1b**, which was acylated with six cycle-2 BBs that were representative of the three kinds of trifunctional groups (i.e., two nitros, two nitriles, and two –*N*Fmoc groups) to afford **44a**–**f**. The subsequent nitro and nitrile reduction and Fmoc deprotection proceeded with excellent conversion to provide **45a**–**f**. The guanidinylation of newly formed amines and anilines went smoothly with reagent A or D and the subsequent –*N*Boc deprotection provided compounds **47a**–**f**. The newly exposed primary amine underwent *N*-capping reactions with a sulfonyl chloride, isocyanate, or anhydride cycle-3 BB to produce a sulfonamide, urea, or amide bond. In addition to those reactions, the primary amine also underwent isocyanate formation using dipyridin-2-yl carbonate followed by reaction with amines and phenols to produce the corresponding urea and carbamate. These reactions collectively afforded the final products **48a**–**l**. The chemistry validation also confirmed that the guanidine has no reactivity toward cycle-3 BBs. Additionally, we confirmed that the reaction conditions have negligible impact on DNA recovery and integrity, which were evaluated with optical density measurement (ultraviolet absorbance at 260 nm) and ligation efficiency.

After establishing all optimized reactions work well in a DECL synthesis setting, the next step was to select the building blocks. Due to two –*N*Boc deprotection reactions that require heating of the DNA material at 80 °C for >24 h, there are some limitations on cycle-1 BB selection. If we use alpha –*N*HBoc amino acids, the amide bond is cleaved by BB2 amino group during the –*N*Boc deprotection during cycle 2 (*SI Appendix*, Fig. S4*A*). Similarly, if we use primary –*N*HBoc amino acids, then the amide –NH reacts with isocyanate intramolecularly during cycle 3 to produce a stable hydantoin (*SI Appendix*, Fig. S4*B*). These results suggest only nonalpha secondary –*N*Boc amino acids can be used in cycle 1. For cycle 2, the BB has to be a primary –*N*HBoc amino acid, preferably containing a nitro, nitrile, or –*N*Fmoc group. We also decided to include BBs that do not have this third functional group and do not undergo guanidine formation but preferably have some guanidine bioisosteres. For cycle-3 *N*-capping reactions, a huge collection of BBs is available and they were selected based on diversity calculations and reactivity.

**Fig. 4.**
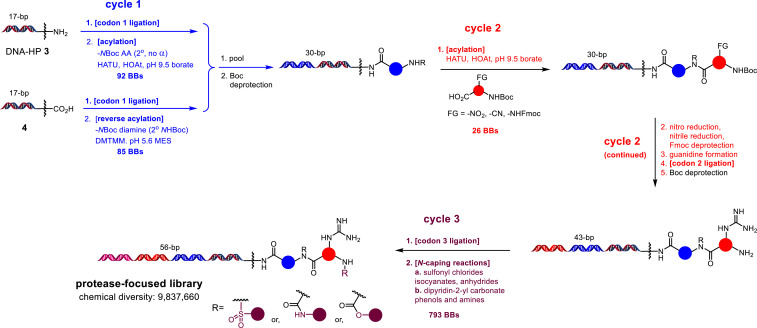
Construction of a 9.8-million-member protease-focused DECL.

We proceeded to construct the library (Fig. 4 and see *SI Appendix* for detailed methods). The synthesis began with two DNA starting materials, amine **3** and carboxylic acid **4**. They were split into a desired number of wells, ligated with cycle-1 codons, and reacted with –*N*Boc amino acids and –*N*Boc diamines. Both individual ligations and acylations were monitored by liquid chromatography–mass spectrometry (LC-MS). After the completion of cycle 1, the DNA materials were pooled and –*N*Boc deprotection was performed. The DNA material was split based on the number of cycle-2 BBs and acylation reactions were carried out. Based on the nature of the BBs, nitro reduction, nitrile reduction, Fmoc deprotection, or guanidine formation reactions were performed. After the completion of these cycle-2 reactions, we performed codon-2 ligations which were monitored by gel electrophoresis. After the completion of ligation, DNA materials were pooled and –*N*Boc deprotection was performed. The DNA material was split and codon-3 ligations were carried out followed by the *N*-capping reactions with cycle-3 BBs. Importantly, each cycle pooled material is used as a blank in the next cycle to encode the unreacted materials in those cycles. After completion of the main library build, the entire library material was ligated with a duplexed pair of 12-mer oligonucleotides to encode the library structure/design. Small portions of this tagged library material were further ligated with two oligonucleotides called closing primers to enable later amplification. At this point, the library material was ready for selection experiments.

### Affinity Selection with Thrombin.

**Fig. 5.**
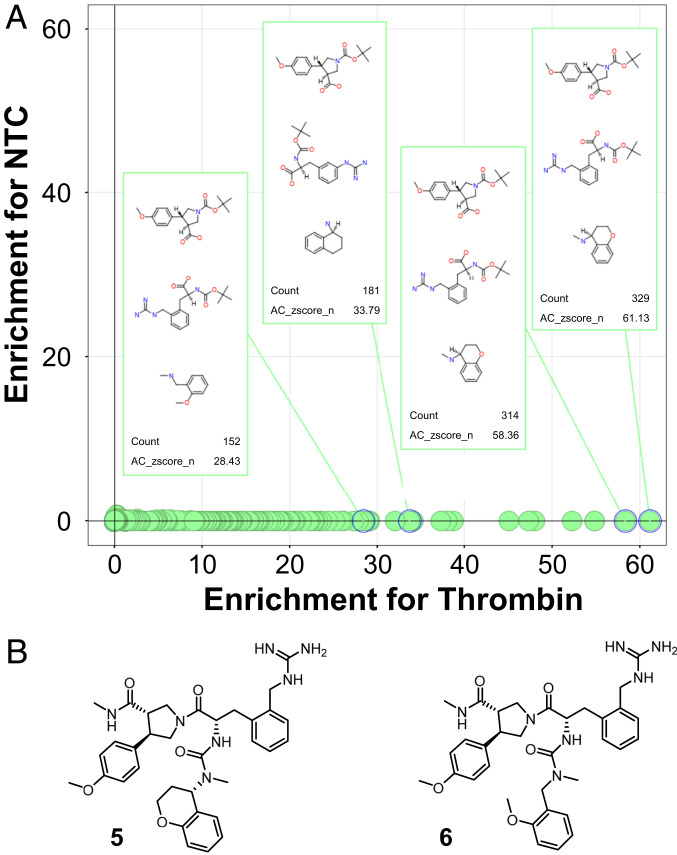
Discovery of thrombin inhibitors from the protease-focused DECL. (*A*) Analysis of enrichment of building blocks in the selection of thrombin with the library. The resulting enrichment values (AC-zscore-n) (36) were compared by plotting the enrichment in the target sample (*x* axis) against enrichment in the NTC sample (*y* axis). The higher the number in the *x* axis, the stronger the enrichment of synthons. (*B*) Structure of the top two thrombin hits **5** and **6** identified from the selection experiment. In the library pool, these molecules are attached to the DNA via the linker at the methyl amide site.

After completion of the build of this 9.8-million-member library and prior to the selection experiment with proteins, the quality of the DECL was evaluated with a naïve sequencing codon distribution report (*SI Appendix*, Fig. S8*A*). Further analysis of the relative codon distributions between each of the cycle-2 subpools confirmed that the codons representing all building blocks were observed (*SI Appendix*, Fig. S8*B*). The *N*Fmoc codon distribution served as a control for DNA integrity when compared to the nitrile reduction method. The observed codon counts were comparable between the building blocks undergoing mild *N*Fmoc deprotection and the ones undergoing nitrile reduction, which was additional evidence of DNA stability under newly developed reaction conditions. After these evaluations of DNA integrity, we performed a selection experiment to find small-molecule binders of thrombin within the library. Selection assays were performed on streptavidin-coated magnetic beads, which were pretreated with biotinylated human thrombin. The binders were then eluted and the DNA was amplified by PCR and analyzed by next-generation Illumina sequencing in comparison with no-target control (NTC). The enrichment of n-synthons (i.e., the encoded association of a chemical building block and unique DNA sequence) for thrombin in comparison with NTC were plotted (Fig. 5*A*) (36). The enrichment of each n-synthon was measured using a normalized z-score metric. The resulting enrichment values (AC-zscore-n) were compared by plotting the enrichment in the target sample (*x* axis) against enrichment in the NTC sample (*y* axis). We observed a strong enrichment of a number of three-cycle trisynthons displaying structure–enrichment relationships. The same trisynthons encoded by different DNA tags were similarly enriched, which confirmed the robustness of the selection result. The enriched features enabled us to identify the structure of the best binders **5** and **6** (Fig. 5*B*). In the library, these molecules are covalently attached to DNA at the methyl amide site.

### Hit Resynthesis and Evaluation of Thrombin Inhibition.

**Fig. 6.**
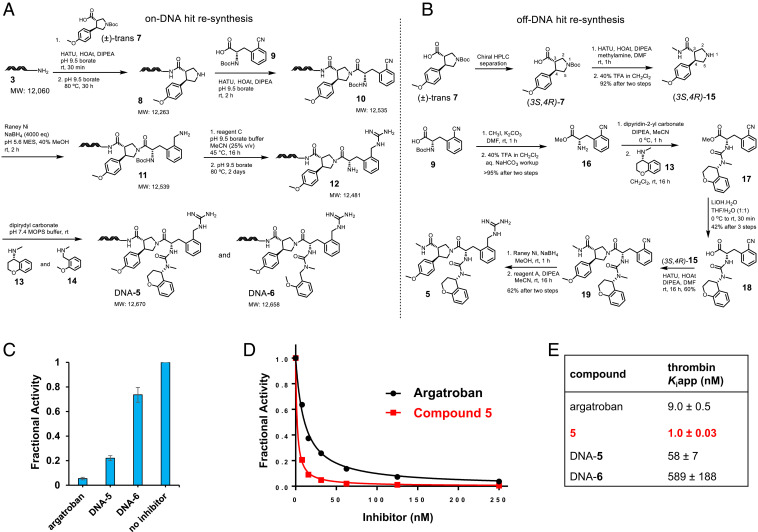
Synthesis and biological evaluation of thrombin hits. (*A*) On-DNA resynthesis of two hits to obtain small-molecule DNA-conjugates DNA-**5** and DNA-**6**. (*B*) Off-DNA resynthesis of the top hit **5**. (*C*) Inhibition of thrombin by DNA-**5** and DNA-**6** compared with argatroban. (*D*) Dose–response curves of thrombin inhibition by compound **5** and argatroban. (*E*) Inhibition constant (apparent *K*_i_) values of synthesized hits. DNA-**5** is 10-fold more potent than DNA-**6**. Compound **5** is 10-fold more potent than argatroban against thrombin.

The identified hits were first synthesized on-DNA. The successful completion of on-DNA synthesis confirmed that the hits are indeed produced in the library. Also, by performing this we were able to identify any byproducts that might have formed during the library build. Thus, we prepared DNA-**5** and DNA-**6** on DNA-HP using the synthetic sequence exactly as in the library synthesis (Fig. 6*A*). DNA-HP **3** on acylation with (±)-**7** followed by –*N*Boc deprotection condition afforded **8**. We anticipate the formation of two diastereomers of **8**. Compound **8** underwent amidation with **9** to provide **10**. Nitrile reduction of **10** afforded **11** which underwent guanidinylation followed by –*N*Boc deprotection to provide **12**. The primary amine **12** produced an isocyanate intermediate in the presence of the reagent dipyridin-2-yl carbonate, which was treated with two amines **13** and **14** to afford urea DNA-**5** and DNA-**6**, respectively. We did not observe formation of any significant by-products along the synthesis and the final products were purified by high-performance LC (HPLC). We studied the thrombin inhibitory potency of DNA-**5** and DNA-**6** (Fig. 6*C*) and observed that DNA-**5** (58 nM), which is the top hit, is about 10-fold more potent than DNA-**6** (589 nM) (Fig. 6*E*).

Our analysis of the on-DNA potency of DNA-**5** and DNA-**6** led to the decision to synthesize isomerically pure **5** off-DNA (Fig. 6*B*). The homochiral 3*S*,4*R*-enantiomer of **7** was the most enriched cycle-1 building block in the selection experiment and we desired to use this pure enantiomer for off-DNA hit resynthesis. The starting material **7** was commercially available as a racemic mixture and the enantiomers of **7** were separated by chiral HPLC with retention times of 9 and 11 min. The absolute configuration of each enantiomer was determined by chemical derivatization to a known compound with reported optical rotation (37). Once we established that the enantiomer with retention time of 9 min was the desired 3*S*,4*R* isomer of **7**, this enantiomer was coupled with methylamine using HATU to provide methyl amide, which under –*N*Boc deprotection condition provided isomerically pure (3*S*,4*R*)-**15**. The synthesis of the other part of the molecule began with compound **9**, which when treated with iodomethane and K_2_CO_3_ afforded the methyl ester which in turn underwent –*N*Boc deprotection to provide free amine **16**. When **16** was treated with dipyridin-2-yl carbonate at 0 °C, the amine was converted to isocyanate, which when treated with freshly prepared secondary amine **13** afforded the urea **17**. The primary amine **16** is prone to react with the isocyanate obtained from itself and produce dimeric urea in 50% yield. Unfortunately, various purification methods were unable to separate the desired product **17** from this by-product. Both **17** and the by-product were taken forward for ester hydrolysis with LiOH at 0 °C. Purification by silica gel chromatography afforded the acid **18** in 42% yield in three steps. We confirmed that compound **18** is a single stereoisomer by optical rotation and NMR, which suggested racemization did not occur during ester hydrolysis. The acid **18** was coupled with amine **15** using HATU to afford **19**, which upon nitrile reduction and guanidinylation with reagent A provided the final product **5**. With purified compound **5** in hand, we examined the inhibitory potency with thrombin. Compound **5** inhibited thrombin very strongly in a dose–response manner (Fig. 6*D*) with an inhibition constant of 1 nM. Compound **5** is about 10-fold more potent thrombin inhibitor than the clinical drug argatroban (9 nM) (Fig. 6*E*).

In summary, starting with known protease pharmacophores, we designed and constructed a protease-focused DECL. Selection of this library with thrombin revealed a number of potent thrombin binders. Our best thrombin inhibitor, urea- and guanidine-functionalized compound **5**, showed a remarkable inhibitory potency (*K*_i_ = 1 nM). The discovery of **5** has led to the initiation of a medicinal chemistry project to further optimize this small molecule thrombin inhibitor with a goal of identifying novel anticoagulants for the treatment of blood clotting complications. In vitro metabolism studies as well as in vivo clotting time studies such as diluted thrombin time and activated partial thromboplastin time are currently ongoing. Additionally, a number of viral, bacterial, and contraception-relevant proteases are undergoing affinity selection with our validated DECL. We anticipate that this approach of utilizing structurally focused DECLs for protease drug discovery can be applied to a wide range of protein targets of therapeutic interest.

## Materials and Methods

Procedure for reactions on DNA, protocols of library synthesis, protocol of affinity selection, procedures of hit resynthesis, ultra-performance LC–MS chromatograms of on-DNA compounds, and NMR characterization data of synthetic compounds are available in *SI Appendix*.

### Data Availability.

The paper and *SI Appendix* contain all datasets generated during this study.

## Supplementary Material

Supplementary File
