# Supplementary material for: Discovery of potent thrombin inhibitors from a protease-focused DNA-encoded chemical library
Source: Proc Natl Acad Sci U S A. 2020 Jul 8;117(29):16782–9. doi: 10.1073/pnas.2005447117 (PMC7382296; doi:10.1073/pnas.2005447117)
Supplement: Supplementary File [file pnas.2005447117.sapp.pdf]

PNAS
www.pnas.org

Supplementary Information for

Discovery of potent thrombin inhibitors from a protease-focused DNA-encoded chemical library

Surendra Dawadi,* Nicholas Simmons, Gabriella Miklossy, Kurt M. Bohren, John C. Faver, Melek Nihan Ucisik, Pranavanand Nyshadham, Zhifeng Yu, and Martin M. Matzuk*

Center for Drug Discovery, Department of Pathology and Immunology, Baylor College of Medicine, Houston, Texas 77030, United States

Surendra Dawadi; Martin M. Matzuk
Email: dawadi@bcm.edu or mmatzuk@bcm.edu

www.pnas.org/cgi/doi/10.1073/pnas.2005447117

Table of Contents

1. General Information

1a) Materials and instrumentation……………………………………………………..…..…….. S3
1b) General analytical procedure for oligonucleotides………………………………..……....S4
1c) General procedure for the isolation of oligonucleotides……………………………..…….S4
1d) General procedure for DNA ligation and gel electrophoresis…………………………..…… S4

2. General Procedure for Chemical Reactions on DNA

2a) DNA-HP synthesis…..………………….……….……………………………….….…..... S5
2b) Acylation using HATU/HOAt………………………….……………………………………….S6
2c) Reverse acylation using DMTMM…………...………………………...……………………S6
2d) -*N*Boc deprotection…………………………………..……………………………….S6
2e) -*N*Fmoc deprotection…………………………………………………………...……. S6
2f) Nitro reduction…..……………………………………………………………………… S6
2g) Nitrile reduction………………………..………………………………………..……… S7
2h) Guanidine formation…………..………………………..……………………………………S7
2i) Reaction of amines with electrophiles……………………….…...……………………..... S7
2j) Conversion of primary amines to isocyanates followed by reaction with nucleophiles……... S7

3. Optimization of Guanidine Formation

3a) Substrate preparation…..……………………….……….…………………………….…… S8
3b) Guanidine formation…………………………….…………………………………………S9

4. Optimization of Nitrile Reduction

4a) Substrate preparation…..……………………….……….………………………….….. S10
4b) Nitrile reduction………………………….………………………………………… S11
4c) Validation of two reactions: nitrile reduction followed by guanidine formation…………... S11

5. Construction of Guanidine Functionalized DNA-Encoded Chemical Library

5a) Chemistry validation…..……………………….……….……………………………….…..S12
5b) Library synthesis……………………….…………………………………………………... S15

6. Selection with Thrombin

6a) Affinity selection…..…………………….……….…………………………….……… …S19
6b) Cheminformatics and hit identification……………….………………………………………S19
6c) On-DNA hit re-synthesis…..……………………….……….………………………………S20
6d) Off-DNA hit re-synthesis…..……………………….……….………………………...……… S21
6e) *In-vitro* assay of hits with thrombin…..……………………….……….……...………..... S24

7. Mass and NMR Spectra

7a) LC/MS traces, *m/z* peaks, and deconvoluted mass spectra of DTSU and DNA-HP…….. …S25
7b) LC/MS traces and deconvoluted mass spectra of guanidine formation…………….. ……..S27
7c) LC/MS traces and deconvoluted mass spectra of nitrile reduction……………………….. ..S76
7d) LC/MS traces and deconvoluted mass spectra of chemistry validation process…....... …..S101
7e) LC/MS traces and deconvoluted mass spectra of on-DNA hit re-synthesis……………......S137
7f) ^{1}H and ^{13}C NMR spectra of off-DNA hit re-synthesis…..……………………………........S143

1. General Information

1a. Materials and instrumentation. The starting material for the synthesis of DNA-encoded chemical libraries, DTSU ("DEC-Tec Starting Unit") **20** (Figure S1), and other 5'-phosphorylated oligonucleotides were obtained from LGC Biosearch Technologies and assessed for purity through the general analytical procedure for DNA oligonucleotides. T4 DNA ligase was obtained from Enzymatics (Qiagen) and the activity was experimentally determined through test DNA oligomer ligations. Standard reagents and solvents were acquired from commercial sources and used without further purification. Chemical building blocks were sourced from a variety of suppliers and solutions were prepared in acetonitrile or mixed aqueous acetonitrile, and aliquots (200 mM) were stored in Tracetraq barcoded tubes (Biosero) at –80 °C. Barcoded tubes were read using a SampleScan 96 scanner (BiomicroLab) and decoded using Vortex software (Dotmatics). All buffers, including HEPES 10X ligation buffer (300 mM HEPES, 100 mM $MgCl_2$, 100 mM dithiothreitol, 10 mM adenosine triphosphate, pH 7.8), basic borate buffer (250 mM sodium borate/boric acid, pH 9.5), MOPS buffer (250 mM MOPS, pH 7.4), and MES buffer (250 mM MES, pH 5.6) were prepared in-house. Library working solutions were prepared using DNase/RNase-free ultra-pure water (Invitrogen), HPLC-grade acetonitrile (Fisher) or high-purity absolute ethanol (Koptec). LC/MS running solvents were made from Optima LC/MS grade water (Fisher), Optima LC/MS grade methanol (Fisher), 99+% purity hexafluoroisopropanol (Sigma) and HPLC-grade triethylamine (Fisher). Solutions were generally transferred or pooled utilizing Biotix brand pipette tips and reservoirs (various sizes), reactions were generally performed in polypropylene, 96-well, deep-well plates (USA Scientific, various sizes), plates were sealed for incubation with AlumaSeal II foil seals (Excel Scientific) and large volume DNA precipitations were performed in polypropylene 250 mL screw-cap bottles (from various vendors). Heated reactions were either performed in ep384 Mastercyclers (Eppendorf) or in laboratory ovens (Fisher). Solutions were centrifuged in either Avanti J-30I or Allegra X-15R centrifuges (Beckman-Coulter). Optical density measurements were made using a Biophotometer (Eppendorf). Resynthesized hits were purified using Teledyne ISCO CombiFlash system equipped with C-18 column. Organic compounds were characterized using a Brooker 600 MHz or 800 MHz NMR spectroscopy. HRMS measurements were performed using ThermoFisher Scientific Q Exactive instrument. Biochemical validation of hit compounds with thrombin was performed using a commercial Thrombin Inhibitor Screening Kit (Fluorometric, Catalog Number MAK243, Sigma-Aldrich). Linear progress curves were obtained in kinetic mode using a Spectramax M5 plate reader (Molecular Devices, San Jose, CA).

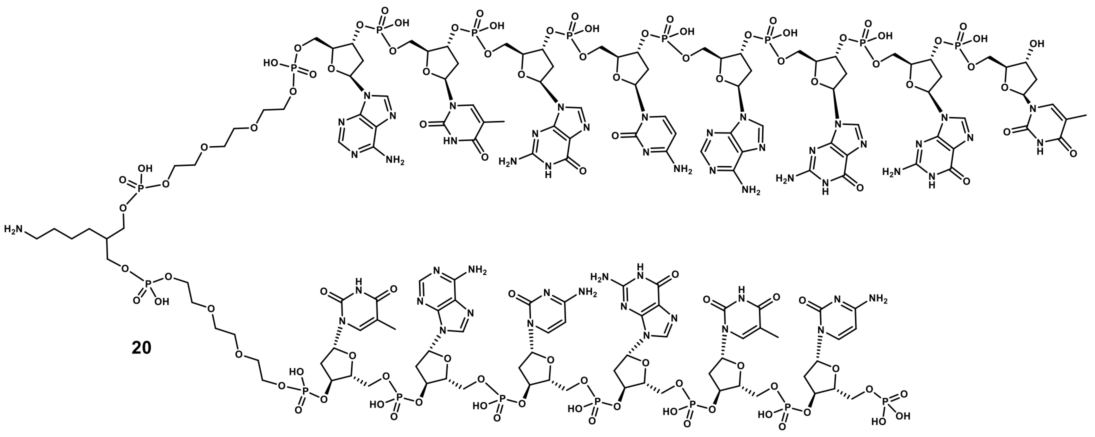

20

Figure S1. Structure of DTSU **20** (5'-Phos-CTGCAT-Spacer 9-Amino C7-Spacer 9-ATGCAGGT 3').

1b. General analytical procedure for the analysis of DNA oligonucleotide compositions. A Vanquish UHPLC system was integrated with LTQ XL ion trap mass spectrometer (ThermoFisher Scientific) for LC/MS analysis of oligonucleotides. Injection amounts were typically 5–10 µL containing 50–100 pmol DNA analyte. Following LC/MS Parameters were used:

I) LC settings

Column: Thermo DNAPac RP (2.1 x 50 mm, 4µm)
Solvent A: 15 mM triethylamine (TEA)/100 mM hexafluoroisopropanol (HFIP) in water
Solvent B: 15 mM TEA/100 mM HFIP in 50% methanol
Solvent C: Methanol
Flow rate: 0.65 mL/min
Run time: 2 min
Column temperature: 100 °C (post column cooler at 40 °C)

II) MS settings

Source: ESI in negative mode
Spray voltage: 4100 V
Source heater temperature: 390 °C
Sheath Gas: 28 (instrument units)
Auxiliary Gas: 8 (instrument units)
Sweep Gas: 2 (instrument units)
Capillary temperature: 350 °C
Capillary voltage: -33.0 V
Tube lens: -92.0 V
MS Scan: 500 – 2000 *m/z*

The data analysis was performed by exporting the raw instrument data (.RAW) to an automated biomolecule deconvolution and reporting software (ProMass) which uses a novel algorithm known as ZNova to produce artifact-free mass spectra. The percent abundance of each species in the deconvoluted mass spectra were used to calculate the conversion of chemical reactions.

1c. General procedure for the isolation of oligonucleotides from aqueous solutions (ethanol precipitation). Based on the DNA solution volume *n*, *n*/25 volume of a 5 M NaCl stock solution was added and the solution was gently mixed. Then absolute ethanol (3*n* volume, 75% *v/v* final ethanol concentration) was added, the solution was thoroughly mixed, and then stored at -20 °C overnight to precipitate the DNA. The resulting slurry was centrifuged (10,000 ×g for 1 h), the supernatant decanted, an addition 2*n* volume of chilled 70% ethanol (*v/v*) was added, and the pellet was centrifuged again (10,000 ×g for 30 min). After decantation of the supernatant, the pellet was dried (in open air or under gentle vacuum) and reconstituted in neutral water or buffer (to a concentration of ~1 mM; assessed by optical density measurements). The solution was then centrifuged (10,000 ×g for 10 min) to pellet any leftover solids (unremoved chemical building blocks or byproducts, denatured ligase, etc.), and the solution was transferred to leave these solids behind. The DNA may undergo a second round of precipitation if the purity is insufficient as assessed by the general analytical procedure. In addition, if the initial solution contains high amounts of organic co-solvent or chaotropic reagents (e.g., piperidine), the solution may be initially diluted with neutral water (by *n* or 2*n*) to enhance the overall precipitation yield.

1d. General procedure for DNA ligation and gel electrophoresis. To a ~1 mM solution of the HP-containing library intermediate (1 equiv.), a premixed solution of the duplex oligonucleotide ("codon") with the appropriate overhang was added (1 mM stock solution in neutral water, 1.05-1.1 equiv.). Separately, a

master-mix consisting of additional water, HEPES 10X ligation buffer, and 200X T4 DNA ligase was prepared and immediately added to the previous DNA mixture with mixing and incubated at rt for overnight. The concentration of the DTSU in the final solution was 0.24 mM (the amount of HEPES 10X ligation buffer was 1/10th and the amount of T4 DNA ligase was 1/200th of the final volume). After the overnight incubation, the ligation progress was assessed by gel electrophoresis. If incomplete, additional buffer, ligase or codon may be added. Gel electrophoresis was performed with a Bio-Rad PowerPac 1000 power supply. Typically, ligation samples were run on a denaturing 6% TBE-Urea gel (Invitrogen) in TBE buffer at 150 V for 40 min. Gels were stained with ethidium bromide, visualized with a Gel Doc (Bio rad), and assessed for transformation into a new, higher-MW band of ligation product (Figure 5A-C).

2. General Procedure for Chemical Reactions on DNA

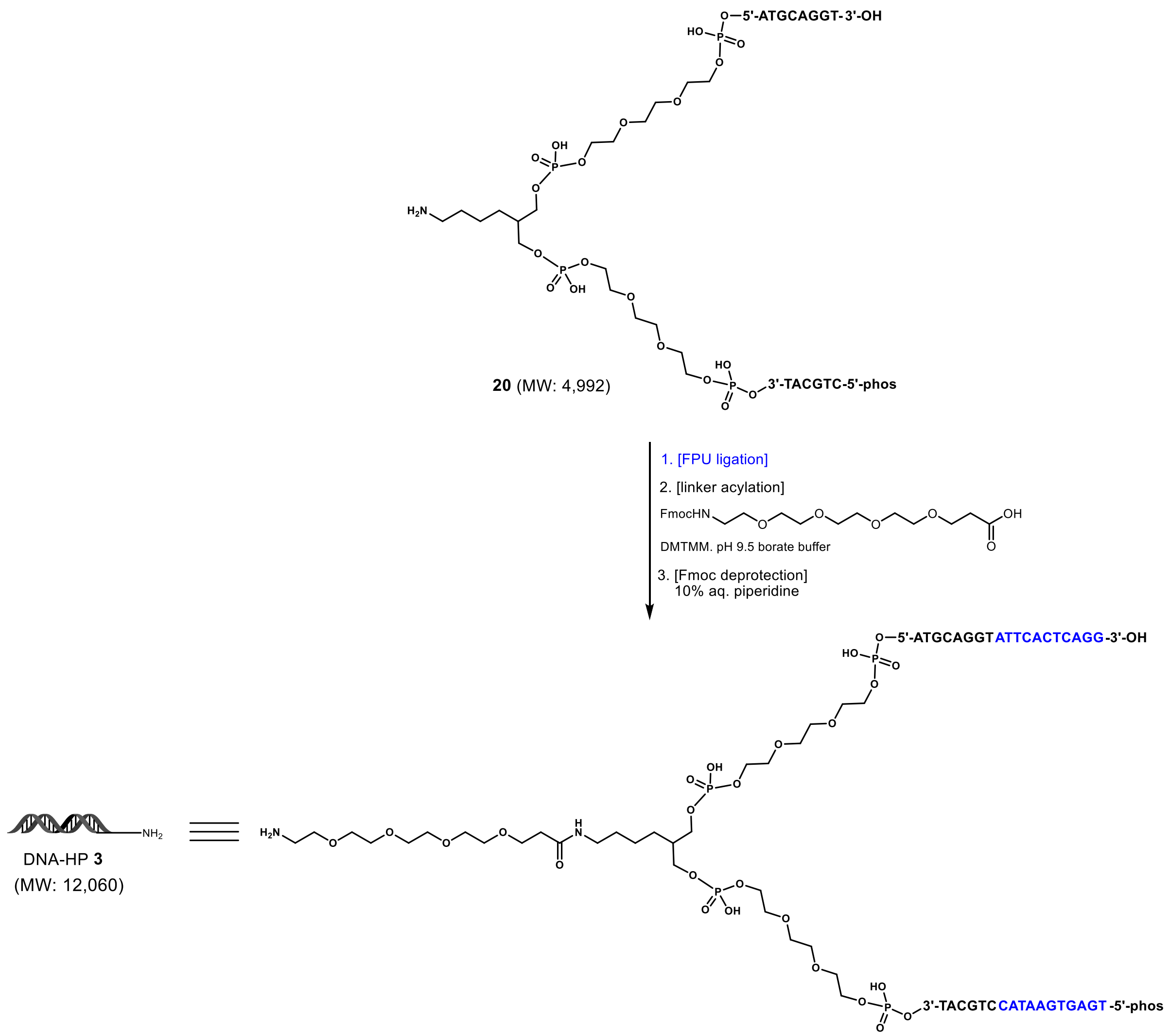

Scheme S1. Synthesis of DNA-HP (DNA-Headpiece) **3**.

2a. DNA-HP synthesis. To a solution of DTSU **20** (10 µmol, 12.1 mM in H_2O, 1 equiv.) in a 250-mL centrifuge bottle, two 5'-Phos 11-mer DNA oligomers FPU ("forward primer unit", FPU-upper = 5'-phos-ATTCACTCAGG, FPU-lower = 5'-phos-TGAGTGAATAC) was added (10.5 µmol each, ~10 mM

solution in H_2O, 1.05 equiv.) and the reaction was performed using the general ligation procedure. After the overnight incubation, the ligation progress was assessed by LC/MS (conversion to the expected product, MW = 11,814). The ligation product was isolated by ethanol precipitation and reconstituted in 10 mL H_2O to give an approximate 1 mM solution. To this solution (10 µmol, 1 mM, 1 equiv.), aqueous sodium borate buffer (5,000 µmol, 20 mL, 250 mM, 500 equiv., pH 9.5), MeCN (2.5 mL), and a solution of Fmoc-15-amino-4,7,10,13-tetraoxapentadecanoic acid (1,000 µmol, 2.0 mL, 500 mM in MeCN, 100 equiv.) was added. After brief mixing, DMTMM (1,000 µmol, 2.0 mL, 500 mM in H_2O, 100 equiv.) was added and the solution was incubated at rt for 2 h. With verification of reaction completion by LC/MS (by formation of expected product, MW = 12,284), the DNA was isolated by ethanol precipitation. To the reconstituted DNA solution (~1 mM, in 10 mL H_2O), an aq. solution of piperidine (10 mL of 10% piperidine *v/v*) was added. After incubation at rt for 3 h, the full deprotection of *N*-Fmoc was verified by LC/MS (formation of expected product, MW = 12,060). After DNA isolation by ethanol precipitation method, the pellets were reconstituted in H_2O (~10 mL) to give a 1.01 mM solution of **3** as assessed by OD measurements. The DNA-headpiece (“DNA-HP”) **3** was used in the next steps without further purification.

2b. Acylation using HATU/HOAt. To a solution of carboxylic acid building block (1000 nmol, 5 µL, 200 mM, 100 equiv.) in MeCN (or 50% DMSO in MeCN) was added a pre-mixed solution of HOAt and DIPEA (1000 nmol each, 10 µL, 100 mM each in MeCN, 100 equiv. each) and HATU (1000 nmol, 5 µL, 200 mM in MeCN, 100 equiv.) and the mixture was kept at rt for 30 min. In another tube, to a solution of amine functionalized DNA (10 nmol, 10 µL, 1.0 mM in H_2O, 1 equiv.) was added pH 9.5 borate buffer (5000 nmol, 20 µL, 250 mM, 500 equiv.). To this DNA solution was added the previously prepared acid mixture and the reaction was allowed to incubate at rt overnight. After the completion of reaction as assessed by LC/MS, DNA was isolated by ethanol precipitation.

2c. Reverse acylation using DMTMM. To a solution of carboxylic acid functionalized DNA (10 nmol, 10 µL, 1.0 mM in H_2O, 1 equiv.) was added pH 5.6 MES buffer (5000 nmol, 20 µL, 250 mM, 500 equiv.), MeCN (10 µL, 30% *v/v*), amine (1000 nmol, 5 µL, 200 mM in MeCN, 100 equiv.), and DMTMM (1000 nmol, 5 µL, 200 mM in H_2O, 100 equiv.) sequentially. The reaction was allowed to incubate at rt overnight and DNA was isolated by ethanol precipitation.

2d. -*N*Boc deprotection. To a solution of DNA containing –*N*Boc group (10 nmol, 10 µL, 1.0 mM in H_2O, 1 equiv.) was added pH 9.5 borate buffer (5000 nmol, 20 µL, 250 mM, 500 equiv.) and the reaction mixture was allowed to incubate at 80 °C. After the completion of reaction, which took 24–48 h depending on the nature of amine, DNA was isolated by ethanol precipitation.

2e. -*N*Fmoc deprotection. To a solution of DNA containing –*N*Fmoc group (10 nmol, 10 µL, 1.0 mM in H_2O, 1 equiv.) an aq. solution of piperidine (10 µL of 10% piperidine *v/v*) was added. After incubation at rt for 3 h, DNA was isolated by ethanol precipitation.

2f. Nitro reduction. Reduction of aromatic nitro compounds to anilines was carried out using a previously reported method (S1). Briefly, to a solution of DNA containing nitro group (10 nmol, 10 µL, 1.0 mM in H_2O, 1 equiv.) was added EtOH (12 µL, 30% *v/v*), 1.5 M NaOH (5000 nmol, 3.3 µL, 500 equiv.), and hypodiboric acid (1500 nmol, 15 µL, 100 mM in H_2O, 150 equiv.) sequentially. After incubation at rt for 2 h, DNA was isolated by ethanol precipitation.

2g. Nitrile reduction. To a solution of DNA-conjugated cyano substrates (50 nmol, 50 μL, 1.0 mM in H_2O, 1 equiv.) was added pH 5.6 MES buffer (25000 nmol, 100 μL, 250 mM in H_2O, 500 equiv.), MeOH (128 μL, 40% *v/v*), and Raney Nickel (2 μL, 50% *w/v* in H_2O) and the mixture was briefly vortexed and kept at rt for 10 min. To the mixture was added a freshly prepared $NaBH_4$ solution (200000 nmol, 40 μL, 5000 mM in pH 5.6 MES buffer, 4000 equiv.) and the mixture was incubated at rt for 2 h at which point the effervescence formation subsided. The metal was pelleted by centrifugation (15,000 ×g for 10 min), the supernatant was carefully transferred to a fresh tube. The metal was washed with 100 μL H_2O by repeating the centrifugation process. The supernatants were combined and DNA was isolated by ethanol precipitation.

2h. Guanidine formation. To a solution of amine or aniline functionalized DNA (10 nmol, 10 μL, 1.0 mM in H_2O, 1 equiv.) was added pH 9.5 borate buffer (5000 nmol, 20 μL, 250 mM, 500 equiv.), and reagent A or B or C or D (2000 nmol, 10 μL, 200 mM in MeCN [reagent A was prepared in 1:1 H_2O/MeCN], 200 equiv.). The reaction was allowed to incubate at 45 °C overnight and DNA was isolated by ethanol precipitation.

2i. Reaction of amines with electrophiles. To a solution of amine functionalized DNA (10 nmol, 10 μL, 1.0 mM in H_2O, 1 equiv.) was added pH 9.5 borate buffer (5000 nmol, 20 μL, 250 mM, 500 equiv.), and electrophiles such as sulfonyl chlorides or isocyanates or anhydrides (2000 nmol, 10 μL, 200 mM in MeCN, 200 equiv.). The reaction was allowed to incubate at rt overnight and DNA was isolated by ethanol precipitation.

2j. Conversion of primary amine to isocyanates followed by reaction with nucleophiles. To a solution of primary amine functionalized DNA (10 nmol, 10 μL, 1.0 mM in H_2O, 1 equiv.) was added pH 7.4 MOPS buffer (5000 nmol, 20 μL, 250 mM, 500 equiv.), and di-2-pyridyl carbonate (1000 nmol, 5 μL, 200 mM in MeCN, 100 equiv.) and the mixture was kept at rt for 1 h. To avoid the formation of carbamate byproduct due to the reaction between intermediate isocyanate and residual EtOH, the DNA was well dried in speed-vac before use for this reaction. After the complete conversion primary amine to isocyante as assessed by LC/MS, nucleophiles such as amines, anilines, and phenols (2000 nmol, 10 μL, 200 mM in MeCN, 200 equiv.) were added. The reaction was allowed to incubate at rt overnight and DNA was isolated by ethanol precipitation.

3. Optimization of Guanidine Formation

3a) Substrate preparation. Compounds **1a-u** were obtained from DNA-HP **3** and twenty-one commercially available –*N*Boc amino acids using HATU/HOAt acylation method followed by using the general –*N*Boc deprotection method. The structure of the products and the conversions are listed below.

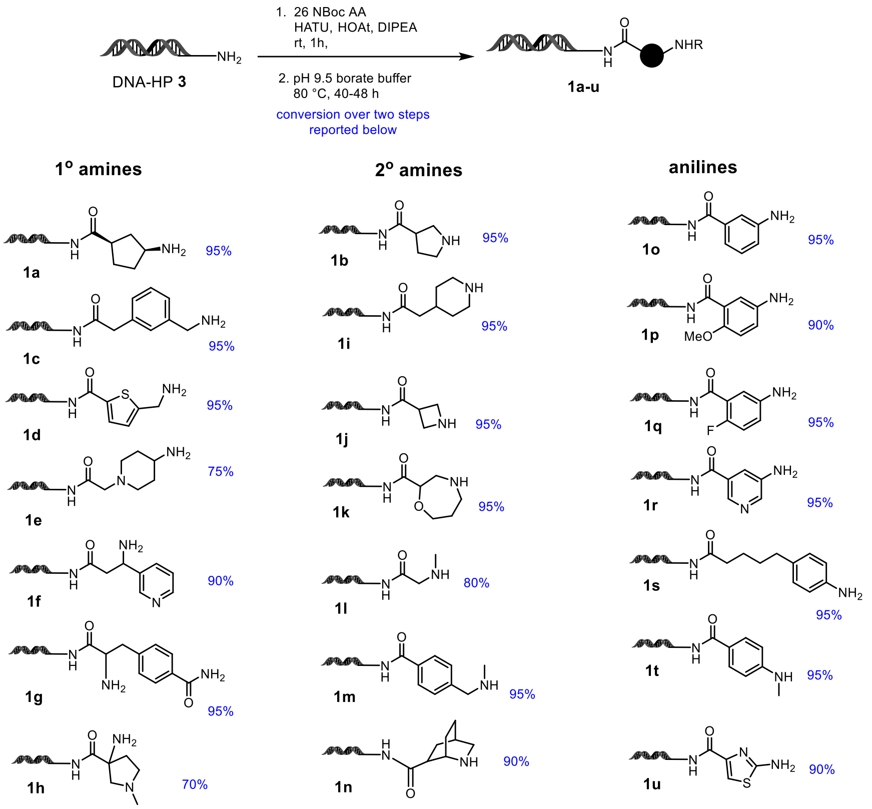

Figure S2. Structure and yield of amines and anilines synthesized to study substrate scope of guanidinylation.

3b) Guanidine formation

Table S1. Optimization of DNA-compatible guanidine formation conditions employing primary amine **1a** and secondary amine **1b.**

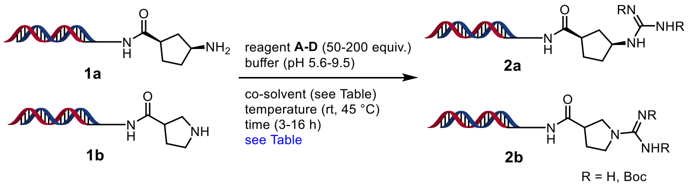

Entry	Substrate	Product	Reagent	Condition	Co-solvent	Conversion (%)
1	**1a**	**2a**	A (200 equiv.)	pH 9.5 borate, rt, 16 h<br>**pH 9.5 borate, 45 °C, 16 h**<br>pH 7.4 MOPS, rt, 16 h<br>pH 7.4 MOPS, 45 °C, 16 h<br>pH 5.6 MES, rt, 16 h<br>pH 5.6 MES, 45 °C, 16 h	MeCN (25% *v/v*)	65<br>**90**<br><5<br>30<br><5<br><5
2	**1a**	**2a**	B (200 equiv.)	pH 9.5 borate, rt, 16 h<br>pH 9.5 borate, 45 °C, 16 h<br>pH 7.4 MOPS, rt, 16 h<br>pH 7.4 MOPS, 45 °C, 16 h<br>pH 5.6 MES, rt, 16 h<br>pH 5.6 MES, 45 °C, 16 h	MeCN (25% *v/v*)	<5<br><5<br><5<br><5<br><5<br><5
3 [a]	**1a**	**2a**	C (200 equiv.)	**pH 9.5 borate, rt, 16 h**<br>**pH 9.5 borate, 45 °C, 16 h**<br>pH 7.4 MOPS, rt, 16 h<br>pH 7.4 MOPS, 45 °C, 16 h<br>pH 5.6 MES, rt, 16 h<br>pH 5.6 MES, 45 °C, 16 h	MeCN (25% *v/v*)	**90** (85:15:0)<br>**>95** (0:40:60)<br>10 (100:0:0)<br>80 (0:0:100)<br><5<br><5
4 [b]	**1a**	**2a**	D (200 equiv.)	pH 9.5 borate, rt, 16 h<br>pH 9.5 borate, 45 °C, 16 h<br>pH 7.4 MOPS, rt, 16 h<br>pH 7.4 MOPS, 45 °C, 16 h<br>pH 5.6 MES, rt, 16 h<br>pH 5.6 MES, 45 °C, 16 h	MeCN (25% *v/v*)	10<br>70<br><5<br>50<br><5<br><5
6	**1a**	**2a**	A (200 equiv.)<br>A (200 equiv.)<br>C (200 equiv.)<br>C (200 equiv.)<br>A (50 equiv.)<br>A (100 equiv.)<br>C (50 equiv.)<br>C (100 equiv.)	pH 9.5 borate, 45 °C, 3 h<br>pH 9.5 borate, 45 °C, 8 h<br>pH 9.5 borate, 45 °C, 3 h<br>pH 9.5 borate, 45 °C, 8 h<br>pH 9.5 borate, 45 °C, 16 h<br>pH 9.5 borate, 45 °C, 16 h<br>pH 9.5 borate, 45 °C, 16 h<br>pH 9.5 borate, 45 °C, 16 h	MeCN (25% *v/v*)	15<br>35<br>70<br>80<br>30<br>60<br>80<br>85
7 [a]	**1b**	**2b**	A (200 equiv.)<br>A (200 equiv.)<br>A (200 equiv.)<br>B (200 equiv.)<br>C (200 equiv.)<br>D (200 equiv.)	**pH 9.5 borate, 45 °C, 16 h**<br>pH 9.5 borate, 45 °C, 16 h<br>pH 9.5 borate, 45 °C, 16 h<br>pH 9.5 borate, 45 °C, 16 h<br>**pH 9.5 borate, 45 °C, 16 h**<br>**pH 9.5 borate, 45 °C, 16 h**	MeCN (25% *v/v*)<br>MeCN (40% *v/v*)<br>EtOH (30% *v/v*)<br>MeCN (25% *v/v*)<br>MeCN (25% *v/v*)<br>MeCN (25% *v/v*)	**>95**<br>>95<br>>95<br>20<br>**>95** (50:40:10)<br>**>95**
8	**1b**	**2b**	A (200 equiv.)<br>A (200 equiv.)<br>C (200 equiv.)<br>C (200 equiv.)<br>A (50 equiv.)<br>A (100 equiv.)<br>C (50 equiv.)<br>C (100 equiv.)	pH 9.5 borate, 45 °C, 3 h<br>pH 9.5 borate, 45 °C, 8 h<br>pH 9.5 borate, 45 °C, 3 h<br>pH 9.5 borate, 45 °C, 8 h<br>pH 9.5 borate, 45 °C, 16 h<br>pH 9.5 borate, 45 °C, 16 h<br>pH 9.5 borate, 45 °C, 16 h<br>pH 9.5 borate, 45 °C, 16 h	MeCN (25% *v/v*)	70<br>95<br>80<br>90<br>90<br>>95<br>90<br>95

[a] For reagent C, ratio of bis-Boc guanidine/mono-Boc guanidine/free guanidine is provided in parenthesis. [b] Reagents B, and D can generate products with or without the protecting groups; the conversion reported is the sum of all possible forms.

4. Optimization of Nitrile Reduction

4a) Substrate preparation. The cyano functionalized DNA-conjugates **21** and **22** were obtained from DTSU **20** and 4-cyanobenzoic acid and 1-cyano cyclopropanecarboxylic acid using HATU/HOAt acylation method, respectively. Compounds **23-27** were obtained from DNA-HP **3** and five cyano-acids using HATU/HOAt acylation method.

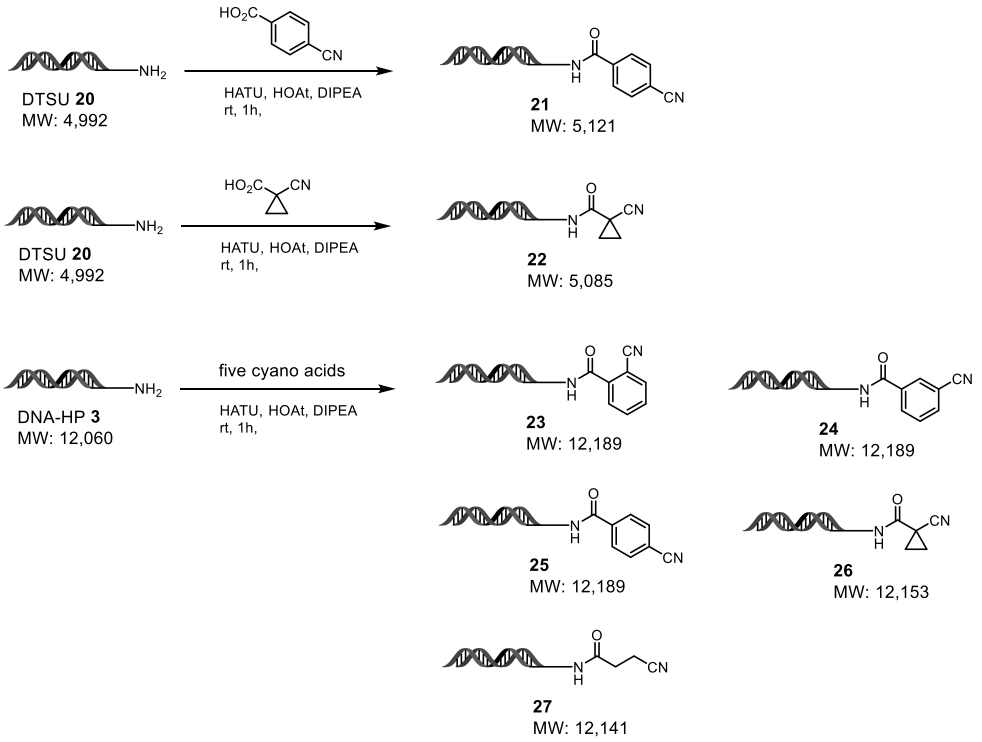

Figure S3. Preparation of nitrile functionalized DNA-conjugates for the study of nitrile reduction.

4b) Nitrile reduction

Table S2. Optimization of DNA-compatible nitrile reduction conditions employing substrates **21-27.** Raney Ni in the presence of $NaBH_4$ in pH 5.6 MES buffer at room temperature gave the best conversions.

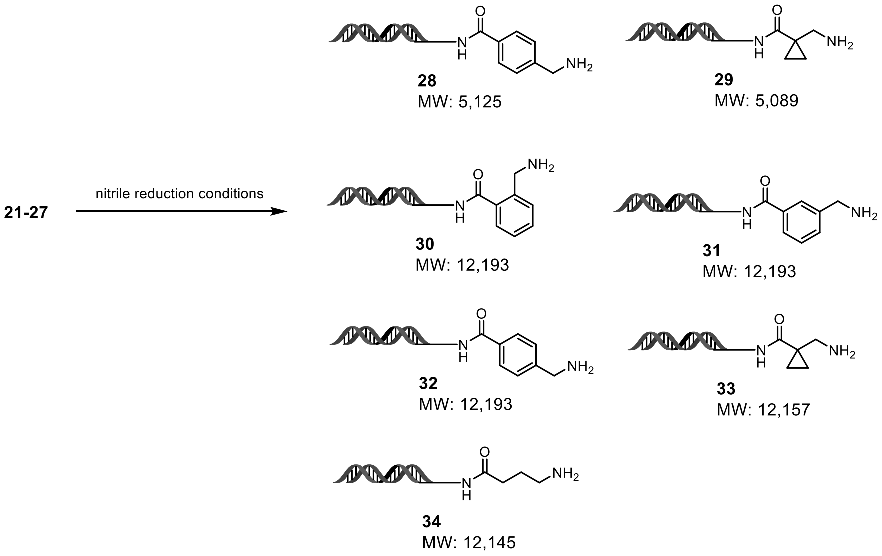

Entry	Substrate	Product	Reducing agent	Condition	Conversion (%)
1	**21**	**28**	Hypodiboric acid<br>Hypodiboric acid + Raney Ni<br>Hypodiboric acid + Pd (0)<br>Hypodiboric acid + Pt (0)	3 pH systems studied (total 12 reactions)	Not detected<br>Not detected<br>Not detected (DNA damage)<br>Not detected (DNA damage)
2	**21**	**28**	**$NaBH_4$ + $NiCl_2$.6H_2O**<br>$NaBH_4$ + $InCl_3$<br>$NaBH_4$ + $CuSO_4$	3 pH systems studied (total 9 reactions)	**40 (pH 5.6 MES)**<br>Not detected<br>Not detected
3	**21**	**28**	**$NaBH_4$ + $NiCl_2$.6H_2O**	6 pH systems studied + EtOH (25% *v/v*) (total 6 reactions)	Not detected (500 eq NaOH)<br>Not detected (pH 9.5 borate)<br>18 (pH 8.2 borate)<br>20 (pH 7.4 MOPS)<br>**50 (pH 5.6 MES)**<br>50 (pH 5.6 MES)
4	**21**<br>**22**	**28**<br>**29**	**$NaBH_4$ (4000 equiv.) + Raney Ni**	6 pH systems studied + 40% co-solvents (MeOH, EtOH, MeCN, MISOP) (total 24 reactions)	**>95 (pH 5.6 MES, 40% MeOH)**<br>**90 (pH 5.6 MES, 40% MeOH)**
6	**23**<br>**24**<br>**25**<br>**26**<br>**27**	**30**<br>**31**<br>**32**<br>**33**<br>**34**	**$NaBH_4$ (4000 equiv.) + Raney Ni**	**pH 5.6 MES, 40% MeOH, rt, 1 h**	80<br>**>95**<br>**>95**<br>**>95**<br>50

4c) Validation of two reactions: nitrile reduction followed by guanidine formation. We studied the formation of guanidine on the primary amine produced by nitrile reduction using guanidinylating reagent A (Scheme S2). This reaction sequence further validates the library design. The validation began with on-DNA secondary amine **1b**.

Compounds **35-37** were obtained from **1b** and three commercially available cyano-acids, using the general HATU/HOAt acylation method.

Compounds **38-40** were obtained from **35-37** using the general nitrile reduction method.

Compounds **41-43** were obtained from benzylic amines **38-40** using the general guanidine formation method employing guanidinylating reagent A. Each intermediate and products was analyzed by UPLC-MS methods.

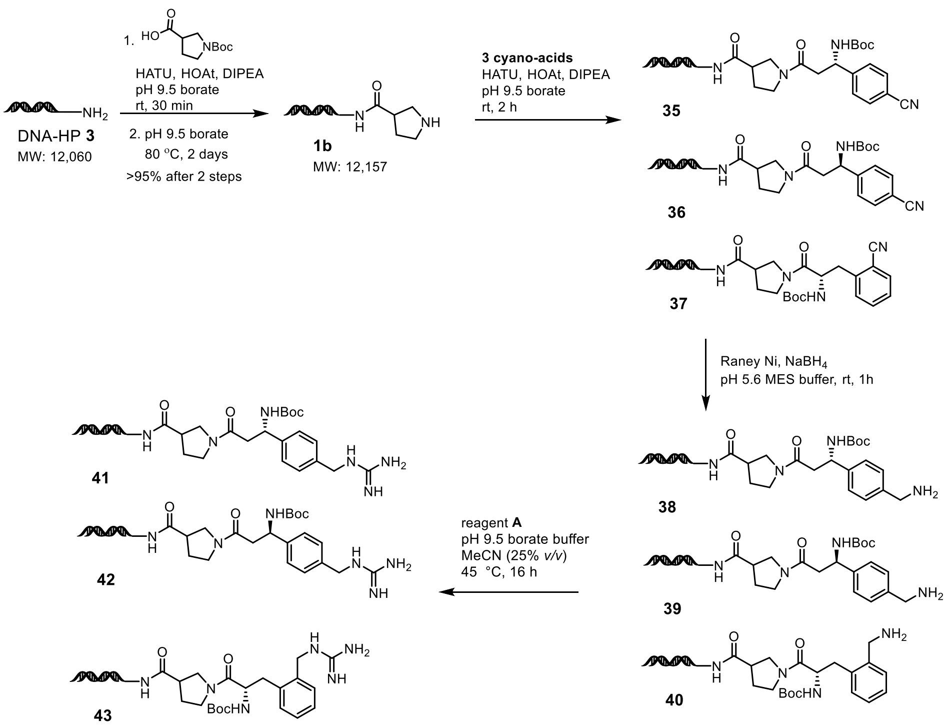

Scheme S2. Validation of nitrile reduction and guanidine formation by the synthesis of products **41-43**.

5. Construction of Guanidine Functionalized DNA-Encoded Chemical Library

5a) Chemistry validation

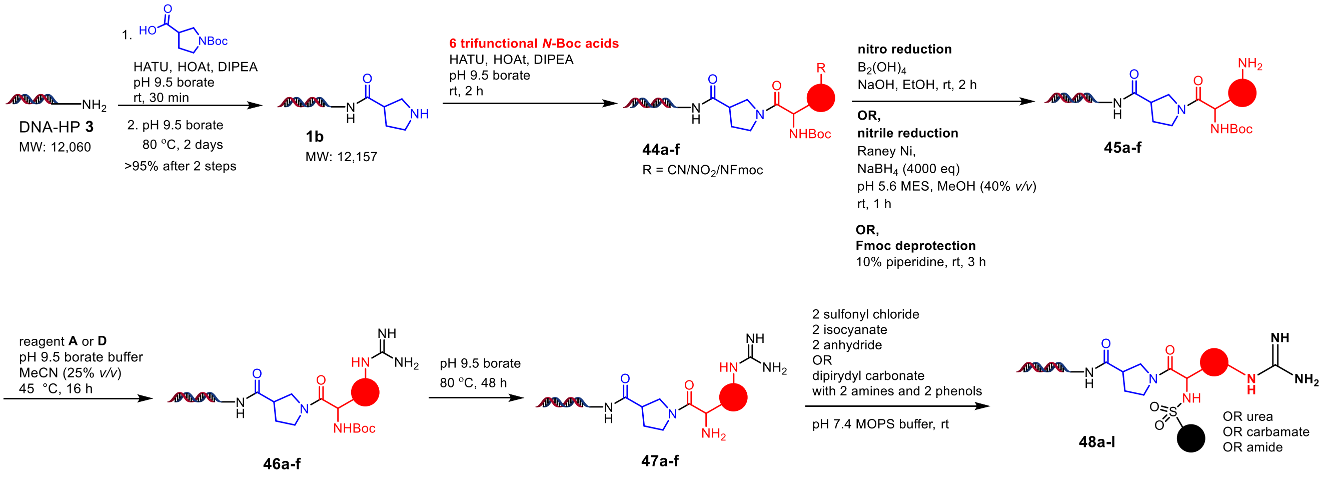

Scheme S3. Validation of chemical reactions that will be employed in protease-focused DECL construction using selected building blocks in each cycle.

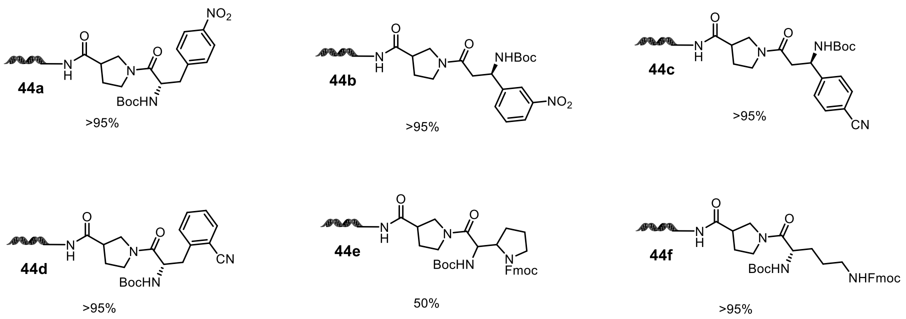

44a-f. Compounds **44a-f** were obtained from **1b** and six commercially available –*N*HBoc amino acids (Boc-4-nitro-L-phenylalanine, Boc-(*R*)-3-amino-3-(3-nitrophenyl)propionic acid, Boc-4-cyano-L-*β*-phenylalanine, Boc-2-cyano-L-phenylalanine, 2-(2-tert-Butoxycarbonylamino-2-carboxy-ethyl)-pyrrolidine-1-carboxylic acid 9*H*-fluoren-9-ylmethyl ester, *N*-alpha-Boc-*N*-delta-Fmoc-L-ornithine, respectively) using the general HATU/HOAt acylation method.

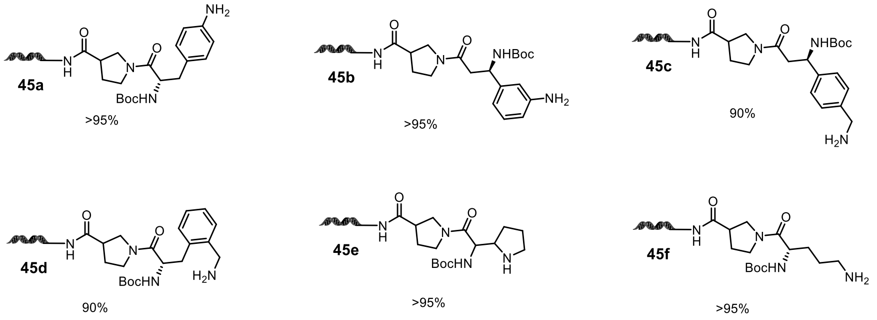

45a-f. Compounds **45a-b** were obtained from **44a-b** using the general nitro reduction. Compounds **45c-d** were obtained from **44c-d** using the general nitrile reduction method. Compounds **45e-f** were obtained from **44e-f** using the general –*N*Fmoc deprotection method.

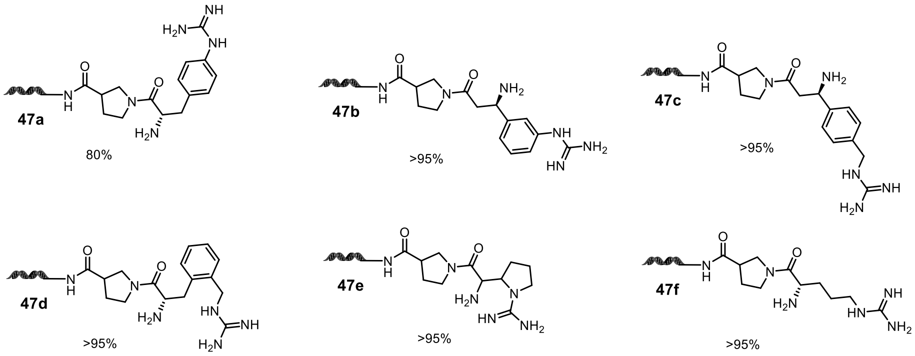

47a-f. Compounds **46a-f** were obtained from **45a-f** using the general guanidine formation method. For **46a**, **46b**, and **46e** reagent D was used and for **46c**, **46d**, and **46f**, reagent A was used. Compounds **47a-f** were obtained from **46a-f** using the general –*N*Boc deprotection method.

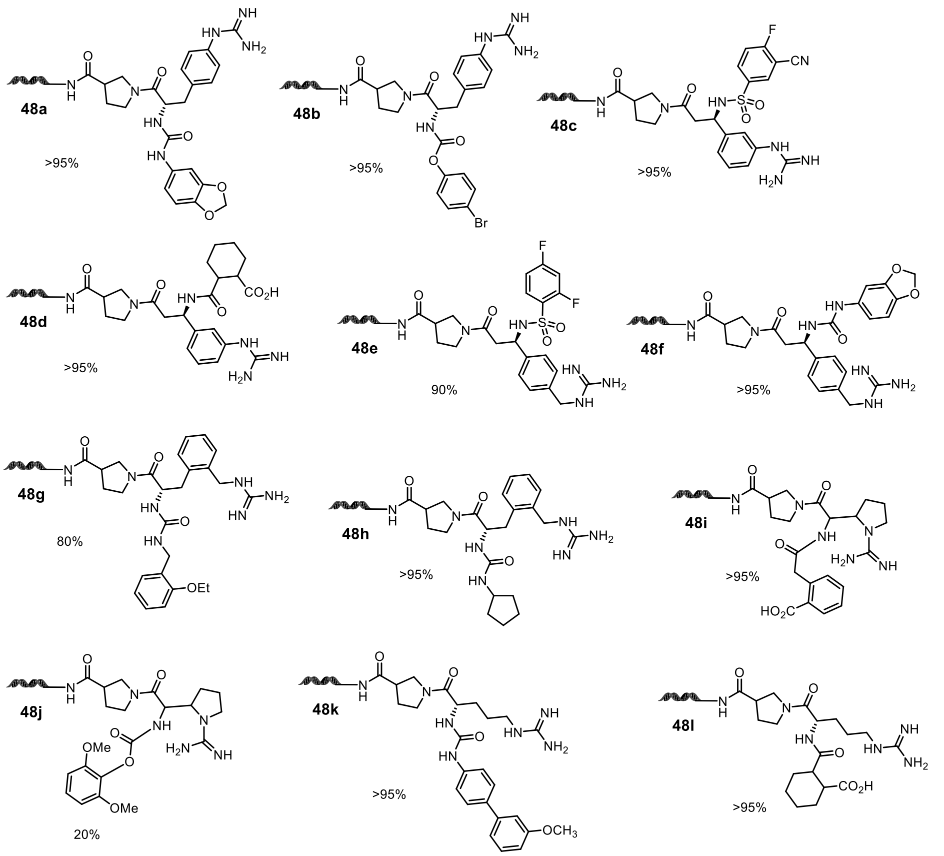

48a-l. Compound **48a** was obtained from **47a** and 3,4-(methylenedioxy)phenyl isocyanate using the general method 2i. Compound **48b** was obtained from **47a** and 4-bromophenol using the general method 2j. Compound **48c** was obtained from **47b** and 3-cyano-4-fluorobenzenesulfonyl chloride using the general method 2i. Compound **48d** was obtained from **47b** and *trans*-1,2-cyclohexanedicarboxylic anhydride using the general method 2i. Compound **48e** was obtained from **47c** and 2,4-difluorobenzenesulfonyl chloride using the general method 2i. Compound **48f** was obtained from **47c** and 3,4-(methylenedioxy)phenyl isocyanate using the general method 2i. Compound **48g** was obtained from **47d** and (2-ethoxyphenyl)methanamine using the general method 2j. Compound **48h** was obtained from **47d** and cyclopentyl isocyanate using the general method 2i. Compound **48i** was obtained from **47e** and homophthalic anhydride using the general method 2i. Compound **48j** was obtained from **47e** and 2,6-dimethoxyphenol using the general method 2j. Compound **48k** was obtained from **47f** and 4-(3-Methoxyphenyl)aniline using the general method 2j. Compound **48l** was obtained from **47f** and *trans*-1,2-cyclohexanedicarboxylic anhydride using the general method 2i.

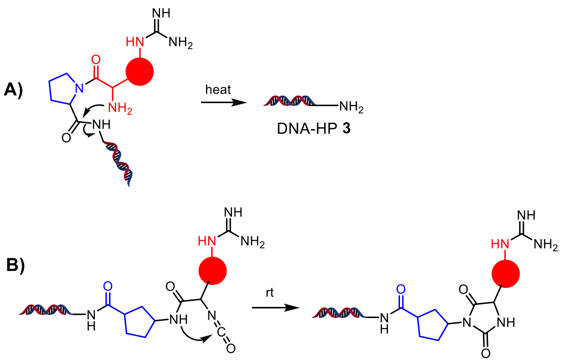

Figure S4. (A) If α-amino acid is used in cycle-1, then amide bond is cleaved by cycle-2 amino group during the Boc deprotection step, producing the DNA-HP **3**. (B) If primary -*N*HBoc amino acid is used in cycle-1, then the amide -NH reacts with isocyanate intramolecularly in cycle-3 to produce stable hydantoin.

5b) Library synthesis

Architecture of the main library build. The DECL build described is a three-cycle split-and-pool combinatorial library, produced through three iterative cycles each containing a chemical reaction phase, an encoding DNA oligonucleotide (codon) ligation phase with a final pooling phase. The library is constructed on DNA-HP **3**, which had been further diversified on the "small molecule end" with two different amino- or carboxy-terminating linkers (Figure S5). Overhangs between codons are two base pairs and encoding regions within codons are eleven base pairs. Specific details and principles related to the overall oligonucleotide sequence design utilized in our DECL production pipeline have been discussed previously (S2).

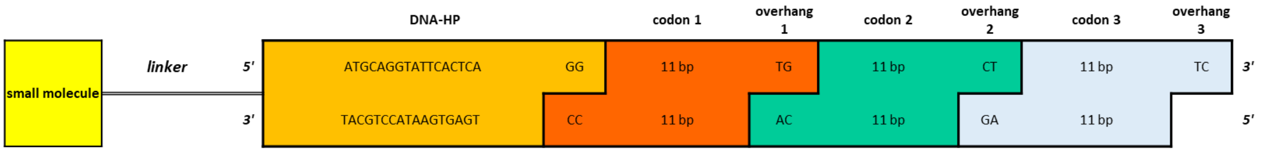

Figure S5. The structure of a completed main library build which consists an oligonucleotide portion and a small molecule portion covalently attached with each other with a linker. Separately assembled/ligated oligonucleotides (codons) in each three cycles are shown in alternate colors.

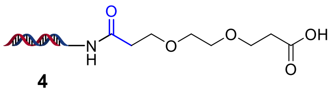

4

MW: 12,248

Synthesis of 4. To a solution of DNA-HP **3** (5 µmol, 5 mL, 1.0 mM in H_2O, 1 equiv.) was added pH 9.5 borate buffer (2500 µmol, 10 mL, 250 mM, 500 equiv.), MeCN (1.3 mL), 3,3'-(ethane-1,2-diylbis(oxy))dipropionic acid (500 µmol, 1 mL, 500 mM in MeCN, 100 equiv.), and DMTMM (500 µmol, 1 mL, 500 mM in H_2O, 100 equiv.) sequentially. The reaction was allowed to incubate at rt overnight and DNA was isolated by ethanol precipitation. The DNA pellets were reconstituted in H_2O (5 mL) to give a ~1.0 mM solution of **4**.

Cycle 1. A 1 mM solution of each **3** and **4** was split into 186 and 186 wells in 96-well plates (22.5 nmol/well) respectively to make a total of four plates. To each well was added an aliquot of one of 372 codon-1 stock solutions (25 nmol, 25 µL, 1 mM in H_2O, 1.1 equiv.) and ligase master-mix (46.5 µL containing 36.7 µL H_2O, 9.4 µL of 10X ligation buffer, and 0.47 µL of T4 DNA ligase). The ligation was allowed to proceed

at rt for overnight and the completion of ligation was confirmed by LC-MS analysis of each wells as well as by gel electrophoresis of selected wells (Figure S6A). The product DNA was isolated by ethanol precipitation and reconstituted in 22.5 μL H_2O to make ~1.0 mM solution. After the ligation was complete, acylation was carried out in the two plates containing DNA-HP **3** with 92 –*N*Boc amino acid using the general HATU/HOAt acylation method. Each building block is thus encoded twice as well as two wells were reserved for blanks (without acylation reaction, no building block was added but all the reagents were added). Reverse-acylation was carried out in the two plates containing carboxylic acid functionalized DNA **4** with 92 –*N*Boc di-amines using the general DMTMM reverse-acylation method. Again, each building block is encoded twice in addition to two blanks. Each well is assessed for the completion of reaction by LC/MS. Seven building blocks in reverse-acylation plates underwent less than 30% conversion, and those wells were discarded. DNA was isolated by ethanol precipitation and reconstituted in H_2O (22.5 μL). DNA material was pooled (except those seven wells) to give a 9 mL of 0.93 mM solution. To this solution was added pH 9.5 borate buffer (18 mL) and Boc deprotection was carried out using the general –*N*Boc deprotection method. After 48 h, the DNA was isolated by ethanol precipitation and reconstituted in 7.0 mL H_2O to make ~1.03 mM solution based on OD measurement.

Cycle 2. The pooled DNA material from cycle-1 was split into 140 wells (42 nmol/well) in a total of four 96-well plates. The first plate utilized 42 wells in six rows, with each rows containing seven wells for each seven building blocks (–*N*HBoc amino acid containing a nitro group). The second plate utilized 36 wells in six rows, with each rows containing six wells for each six building blocks (–*N*HBoc amino acid containing a nitrile group). The third plate utilized 48 wells in six rows, with each rows containing eight wells for each eight building blocks (–*N*HBoc amino acid containing a -*N*HFmoc group). The fourth plate utilized 14 wells, five wells in each two rows for each five building blocks (–*N*HBoc amino acid with no other functional group, these wells do not undergo guanidine formation), third row containing two wells for two blanks (no building block added), and fourth two wells for two blanks that undergoes guanidinylation of cycle-1 material. Each building blocks and the blanks were encoded twice. To each well acylation reactions was performed using the 26 cycle-2 building blocks mentioned above and the general HATU/HOAt acylation method. DNA was isolated by ethanol precipitation and reconstituted in H_2O to make ~1 mM solution. No further reactions were performed in the first two rows of plates 1-4. Plate-1 rows 3-6 underwent nitro reduction, plate-2 rows 3-6 underwent nitrile reduction, plate-3 rows 3-6 underwent Fmoc deprotection. After those reactions were complete, DNA was isolated by ethanol precipitation and reconstituted in H_2O to make ~1 mM solution. Furthermore, plates1-3 rows 5-6 underwent guanidine formation as well as the two wells of plate-4 row 4. After the completion of guanidinylation, DNA was isolated by ethanol precipitation and reconstituted in H_2O to make ~1 mM solution. To each well was added an aliquot of one of 140 codon-2 stock solutions and the ligation was allowed to proceed at rt for overnight and the completion of ligation was confirmed by gel electrophoresis of selected wells (Figure S6B). The product DNA was isolated by ethanol precipitation and reconstituted in H_2O. DNA material was pooled to give a total 10 mL solution. To this solution was added pH 9.5 borate buffer (20 mL) and Boc deprotection was carried out using the general –*N*Boc deprotection method. After 30 h, the DNA was isolated by ethanol precipitation and reconstituted in H_2O (8.0 mL) to make ~0.65 mM solution based on OD measurement.

Cycle 3. The pooled DNA material from cycle-2 was split into 795 wells (6.2 nmol/well) in a total of ten plates. To each well was added an aliquot of one of 795 codon-3 stock solutions and the ligation was allowed to proceed at rt for overnight and the completion of ligation was confirmed by gel electrophoresis of selected

wells (Figure S6C). The product DNA was isolated by ethanol precipitation and reconstituted in H_2O (10 μL) to give ~0.62 mM solution. Each building blocks and the blanks were encoded once. To 94 wells in plate-1, 93 sulfonyl chlorides were added and the reactions were performed using the general method 2i. The last well is for encoding the blank, so no building block was added. To 91 wells in plate-2, 91 isocyanates were added and the reactions were performed using the general method 2i. To 10 wells in plate-3, 10 anhydrides were added and the reactions were performed using the general method 2i. To 24 wells in plate-4, the reactions were performed with 23 phenols using the general method 2j. The last well is for encoding the blank with isocyanate formation, so dipyridyl carbonate was added but not the building blocks. To 192 wells in plates5-6, the reactions were performed with 192 anilines using the general method 2j. To 384 wells in plates7-10, the reactions were performed with 384 anilines using the general method 2j. After overnight, DNA was isolated by ethanol precipitation and reconstituted in H_2O and pooled together (final volume: 4.0 mL) to make ~1.2 mM solution based on OD measurement. This makes a 59% recovery of DNA material after 3 cycles of chemistry and ligations.

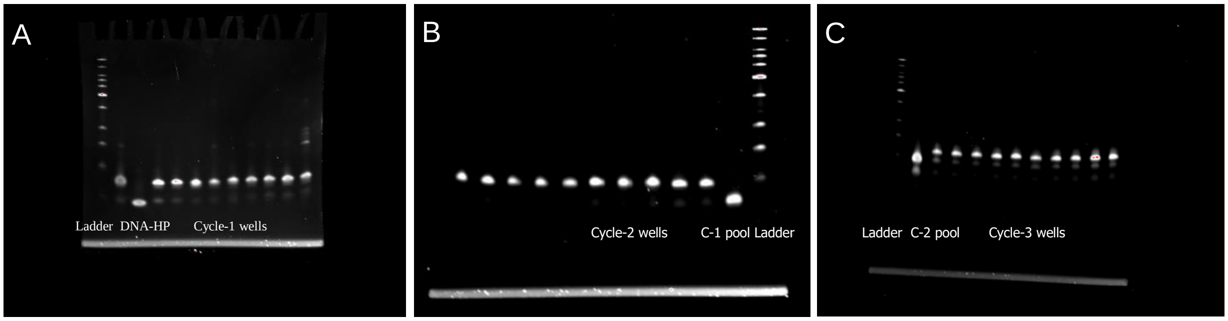

Figure S6. (A) 6% TBE-urea denaturing gel of selected wells that demonstrates the completion of codon-1 ligation with DNA-HP **3**. (B) Gel of codon-2 ligation with C-1 pool DNA material. (C) Gel of codon-3 ligation with C-2 pool DNA material.

Preparation of amplifiable DEL samples ("shots") for naïve sequencing and selection experiments. After completion of the main library builds, the entire library material was ligated with a duplexed pair of 12-bp oligonucleotides to encode the library structure/design. The completion of ligation was confirmed by gel electrophoresis (Figure S7A). After DNA isolation by ethanol precipitation, small portions of this material were further ligated with two oligonucleotides called closing primer (CP) containing a region to encode experimental usage, a degenerate region as an amplification control, a segment to increase sequencing base diversity, and a reverse primer region to enable post-selection PCR amplification (the purposes/design of these components are discussed elsewhere) (S2). The completion of the CP ligation was confirmed by gel electrophoresis (Figure S7B).

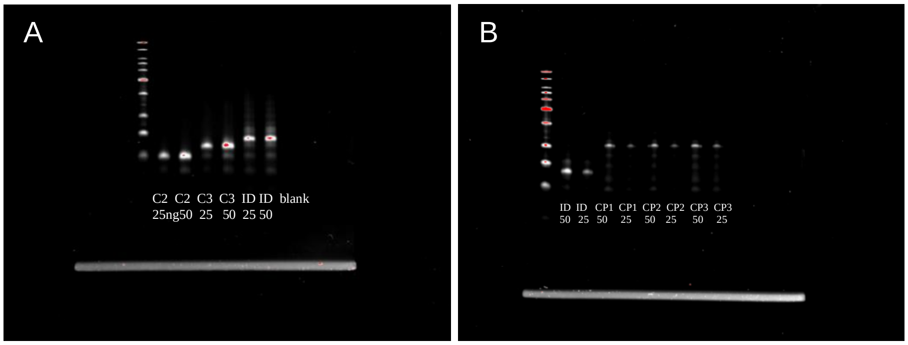

Figure S7. (A) 6% TBE-urea denaturing gel demonstrating the completion of library ID ligation. (B) 6% TBE-urea denaturing gel demonstrating the completion of closing primer (CP) ligation.

Naïve sequencing. An amplifiable DECL shot was quantified by quantitative Real-Time PCR (qPCR) and then a total of 3×10^7 DNA copies were amplified by PCR with primers adaptors to add the sequences compatible with Illumina sequencing flowcells. Platinum Taq DNA Polymerase High Fidelity (Thermo Fisher Scientific) PCR reagent was used for PCR amplification. A total of 13 PCR cycles were used for amplification and the following PCR conditions were used (Initial denaturation at 95 °C for 2.5 min, denaturation at 95 °C for 30 s, annealing at 58 °C for 30 s, extension at 72 °C for 1 min and final extension at 72 °C for 10 min). PCR library temple was purified using Agencount AMPure XP SPRI beads according to the manufacturer's instructions. The purified library was analyzed in Bioanalyzer 2100 (Agilent Technologies, Santa Clara, CA) by using Agilent high sensitivity DNA kit to verify library size and concentration before clustering. Illumina NextSeq 500 was used for sequencing. The sequenced data was analyzed for population distribution of codons. We separately analyzed populations of cycle 1, 2, and 3 mono-synthons as well as all di-synthons involving pairs of cycles. All codon sequences were observed after naïve sequencing, and distributions of codon populations were nominal (Figure S8), with the exception of some low populated codons in cycle 3, which encoded a subset of sulfonyl chloride building blocks (appearing at around 40-50% of the mean population).

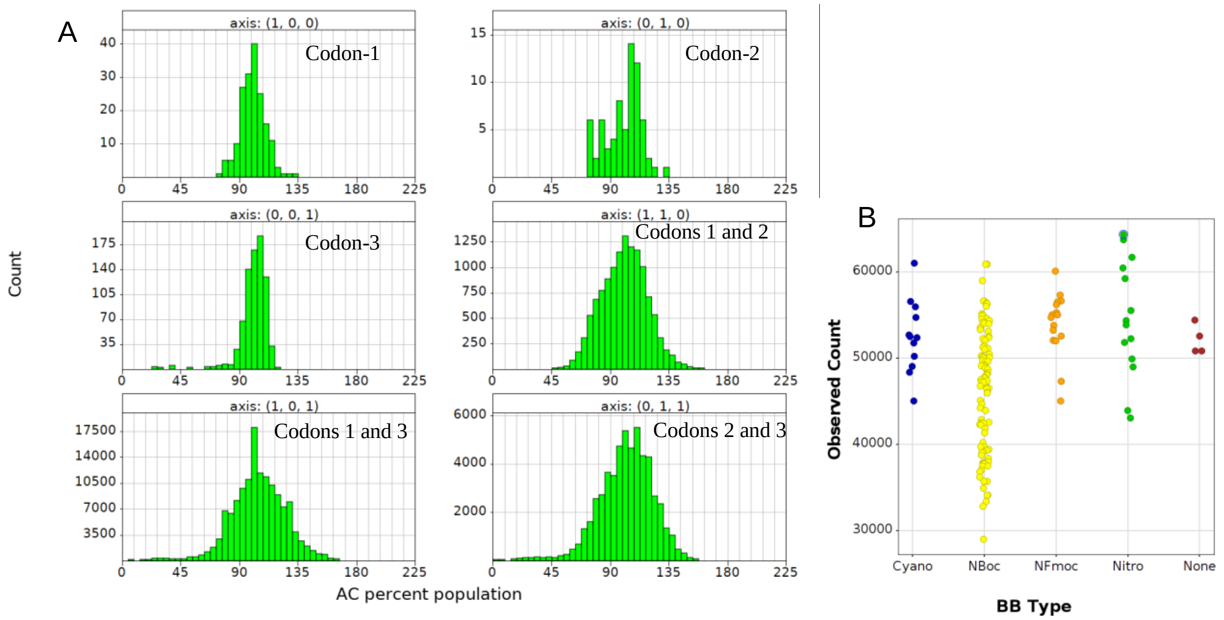

Figure S8. Codon distribution analysis of protease-focused DNA-encoded chemical library. (A) Distribution of codons representing mono-synthons and various combination of di-synthons. All codons were represented well except few in cycle-3 encoding sulfonyl chlorides. (B) Analysis of the relative codon distributions between each of the cycle 2 subpools. Codons encoding all building blocks were observed. The *N*Fmoc codon distribution is a control for DNA integrity when compared to the new nitrile reduction method. This is an additional evidence of DNA stability under newly developed the reaction conditions.

6. Selection with Thrombin

6a) Affinity selection. 0.5 µM biotinylated human thrombin (Novagen, # 69672-3) was immobilized on streptavidin C1 magnetic beads (ThermoFisher, # 65001) in the selection buffer (20 mM Tris-HCl, pH 8.0, 150 mM NaCl, 1 mM CHAPS, 2.5 mM $CaCl_2$) for 30 min at rt with gentle shaking. The beads were washed three times with wash buffer (20 mM Tris-HCl, pH 8.0, 150 mM NaCl, 1 mM CHAPS, 2.5 mM CaCl2, 0.5mM biotin) and once with the selection buffer. Library selection was then performed by incubating the thrombin-coated beads with the DNA-encoded chemical library where 1 million copies of each compound were present in the selection buffer containing 0.1 mg/ml sheared salmon sperm DNA (Invitrogen, 15632011) for 30 min at rt with continuous shaking. The Non-Target Control (NTC) in the absence of protein was performed in parallel as a reference. At the end of the incubation, beads were washed twice with the selection buffer without salmon sperm DNA by brief and vigorous vortex. The binding molecules were then eluted by heating the beads at 80 °C for 10 min in selection buffer without salmon sperm DNA. The resulting eluent was quantitated using qPCR and determined whether another round selection was needed. Totally 4 rounds of selection were performed, and the oligonucleotides in the final eluent were PCR amplified for 15 cycles using Platinum Taq DNA Polymerase High Fidelity (Thermo Fisher Scientific, 10966026) with denaturation at 95 °C, annealing at 58 °C, and extension at 72 °C using primers that incorporate complementary sequences to the library headpiece or tailpiece along with the Illumina READ 1 or READ 2 sequences required for Illumina clustering and sequencing. The amplified DNA was cleaned by Agencourt AMPure XP beads and quantitated with Agilent high sensitivity DNA kit using a Bioanalyzer before the sequencing.

6b) Cheminformatics and hit identification. Raw DNA sequence reads (in the form of FASTQ files), quality metrics, and sequencing index-to-sample attribute value pairs were obtained from Illumina BaseSpace at the conclusion of sequencing. Samples were linked to their respective FASTQ files based on their sequencing index (DTSU) and were expanded into individual experiments if they were part of a larger pool. Individual samples were then decoded by perfectly matching individual oligonucleotide sub-structures without gaps and in the order defined by the known DNA encoding structure. Valid DNA barcodes were annotated with the corresponding oligonucleotide sequence-to-building block lookup for each of the three codon cycles, which collectively represent a distinct small molecule within a specific DECL. The degenerate UMI (unique molecular identifier) portions of the DNA barcodes were accumulated into a list of UMIs for each unique codon tuple as a method to distinguish experimental vs. amplification events. Unique molecule counts were then evaluated using a directed-graph counting model as described previously (S2). The set of unique codon tuples with unique molecule counts was then aggregated across all possible combinations of codons (all n-synthons), and enrichment for each n-synthon was evaluated independently. Enrichment was evaluated with a normalized z-score metric which normalizes for sampling and library diversity (S2). The resulting enrichment values were then compared by plotting the enrichment in the target sample against enrichment in the NTC sample (Figure 4 in main text). DECL members with significant binding affinity for the target are detected by observing their component n-synthons as enriched on the target axis but not the NTC axis. Figure 5 (main text) highlights tri-synthon components of the thrombin hit series. Importantly, the measured enrichment of each displayed tri-synthons were observed in multiple encodings in each cycle.

6c) On-DNA re-synthesis of top two hits.

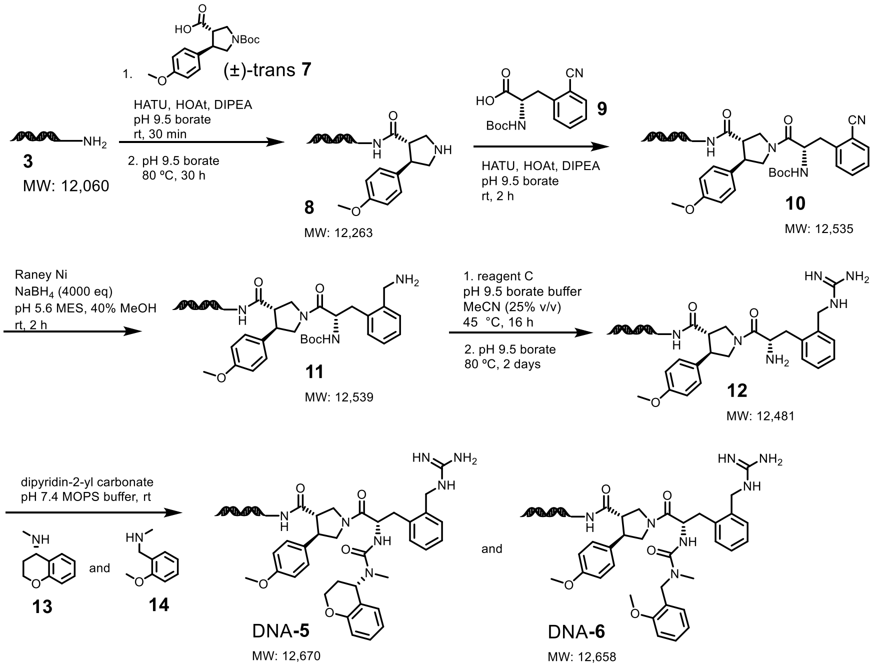

Scheme S4. Synthesis of thrombin hits as DNA conjugates, **DNA-5** and **DNA-6**. The DNA oligonucleotide used for this purpose is the 17-base pair DNA-HP **3**.

8. Compound **8** was obtained from DNA-HP **3** and (±)-*trans*-1-(*tert*-butoxycarbonyl)-4-(4-methoxyphenyl)pyrrolidine-3-carboxylic acid **7** using the general HATU/HOAt acylation method followed by the general –*N*Boc deprotection method. MS: calcd 12,263, found 12,264.

10. Compound **10** was obtained from **8** and (*S*)-2-((*tert*-butoxycarbonyl)amino)-3-(2-cyanophenyl)propanoic acid **9** using the general HATU/HOAt acylation method. MS: calcd 12,535, found 12,537.

11. Compound **11** was obtained from **10** using the general nitrile reduction method. MS: calcd 12,539, found 12,542.

12. Compound **12** was obtained from **11** using the general guanidine formation method followed by the general –*N*Boc deprotection method. MS: calcd 12,481, found 12,484.

DNA-5. Compound **DNA-5** was obtained from **12** using the general isocyanate formation method 2j followed by the addition of amine **13**. DNA-**5** was purified using Agilent 1260 Infinity II series HPLC system consisting of an autosampler, degasser, quaternary pump and a diode array detector coupled to an analytical scale fraction collector. The mobile phase comprised of following solvent systems: Solvent A: 0.1 M TEAA (pH 7.0) and Solvent B: Acetonitrile/0.1M TEAA, 40/60, v/v. HPLC separations were performed under gradient conditions (5% B at 1.0 min to 30% B at 4.0 min to 60% B at 11.0 min to 95% B at 12.0 min) at a flow rate of 3.0 ml/min using waters xbridge BEH C18 column (10 x 50 mm, 2.5 µm) with a run time of 15 minutes. UV detection was done at a wavelength of 260 nm. The compound eluted at a retention time of 10.1 min and the collected fractions were evaporated and lyophilized to obtain DNA-**5** as solid residue. MS: calcd 12,670, found 12,672.

DNA-6. Compound **DNA-6** was obtained from **12** using the general isocyanate formation method 2j followed by the addition of amine **14**. It is purified by C-18 reverse phase preparative HPLC method described above. MS: calcd 12,658, found 12,658.

6d) Off-DNA re-synthesis of the top hit

(+)-(3S,4R)-4-(4-methoxyphenyl)-N-methylpyrrolidine-3-carboxamide **((+)-15)**

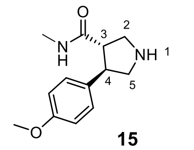

15

Chemical Formula: $C_{13}H_{18}N_2O_2$
Molecular Weight: 234.3

The (±)-7 enantiomer separation was carried out using Agilent 1260 Infinity prep HPLC system equipped with CHIRALPAK IC (21 × 250 mm, 10 µm) preparative column. Solvent system comprising of heptane (A) and ethanol (B) were used under gradient conditions (5% B at 1.0 min to 40% B at 10.0 min to 95% B at 11.0 min) at a flow rate of 20 ml/min with a run time of 15 minutes. UV detection was performed at a wavelength of 220 nm with a peak width > 0.025 min (0.5 s response time). The enantiomers were eluted at retention times of 9.4 min and 10.9 min respectively and collected using prep scale fraction collector. The collected fractions were evaporated to obtain enantiomers of **7** as white solids. To a solution of (3*S*,4*R*)-1-(*tert*-butoxycarbonyl)-4-(4-methoxyphenyl)pyrrolidine-3-carboxylic acid **7** (100 mg, 0.311 mmol, 1 equiv., retention time 9.4 min) in DMF (6.2 mL) was added HOAt/DIPEA mixture (6.2 mL, 100 mM each in MeCN, 2 equiv.), HATU (236 mg, 0.622 mmol, 2 equiv.), and methylamine hydrochloride (42 mg, 0.622 mmol, 2 equiv.) and the mixture was stirred at rt for 1 h. The reaction mixture was concentrated under reduced pressure, dissolved in EtOAc (10 mL), and washed with sat. $NaHCO_3$ (10 mL) and brine. The organic layer was dried (Na_2SO_4) and concentrated under reduced pressure. Purification by flash chromatography (SiO_2, MeOH/CH_2Cl_2 gradient) afforded the coupled product as a white foam. This product was dissolved in 40% TFA in CH_2Cl_2 (5 mL) and the mixture was stirred at rt for 30 min at which point the Boc deprotection was completed as monitored by LCMS. The reaction mixture was concentrated under reduced pressure and purified by C-18 reverse phase chromatography (10% MeCN/0.1% FA in H_2O to 100% MeCN) afforded the product **15** as a white solid (67 mg, 92%). $[\alpha]_D^{25}$ +108.7 (c 1.0, MeOH); ^{1}H NMR (600 MHz, CD_3OD) δ 2.64 (s, 3H), 3.14 (app q, *J* = 9.3 Hz, 1H), 3.35 (app t, *J* = 11.4 Hz, 1H), 3.51–3.47 (m, 1H), 3.64–3.59 (m, 1H), 3.72–3.69 (m, 2H), 3.77 (s, 3H), 6.91 (d, *J* = 8.7 Hz, 2H), 7.23 (d, *J* = 8.7 Hz, 2H); ^{13}C NMR (151 MHz, CD_3OD) δ 26.6, 49.2, 49.4, 52.3, 52.6, 55.9, 115.6, 129.7, 129.8, 161.0, 172.7; HRMS (ESI+) *m/z* calcd for $C_{13}H_{19}N_2O_2^+$ [(M + H)$^+$] 235.1441, found 235.1436 (Δ –1.9 ppm).

methyl (S)-2-amino-3-(2-cyanophenyl)propanoate **((+)-16)**

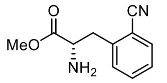

16

Chemical Formula: $C_{11}H_{12}N_2O_2$
Molecular Weight: 204.2

To a solution of (*S*)-2-((*tert*-butoxycarbonyl)amino)-3-(2-cyanophenyl)propanoic acid **9** (290 mg, 1 mmol, 1 equiv.) in DMF (10 mL) was added anhydrous K_2CO_3 (415 mg, 3 mmol, 3 equiv.), and iodomethane (421 mg, 3 mmol, 3 equiv.) and the mixture was stirred at rt for 1 h. The reaction mixture was filtered and concentrated under reduced pressure. Purification by flash chromatography (SiO_2, EtOAc/hexanes gradient) afforded the coupled product as a white foam. This product was dissolved in 40% TFA in CH_2Cl_2 (10 mL) and the mixture was stirred at rt for 30 min at which point the Boc deprotection was completed as monitored by LCMS. The reaction mixture was concentrated under reduced pressure, dissolved in CH_2Cl_2 and washed with sat. $NaHCO_3$ (10 mL). The organic layer was dried (Na_2SO_4) and concentrated under reduced pressure to afford the product **16** as a white solid (200 mg, >95%). $[\alpha]_D^{25}$ +25.1 (c 1.0, $CHCl_3$); ^{1}H NMR (600 MHz, $CDCl_3$) δ 1.70 (br s, 2H), 3.05 (dd, *J* = 13.9, 8.5 Hz, 1H), 3.30 (dd, *J* = 13.7, 5.4 Hz, 1H), 3.72 (s, 3H), 3.82–3.80 (m, 1H), 7.34

(app t, J = 7.6 Hz,1H), 7.37 (app d, J = 7.8 Hz, 1H), 7.53 (app t, J = 7.6 Hz, 1H), 7.63 (app d, J = 7.7 Hz, 1H); ^{13}C NMR (151 MHz, $CDCl_3$) δ 39.5, 52.4, 55.5, 113.4, 118.1, 127.5, 130.6, 132.9, 133.1, 141.7, 174.9; HRMS (ESI+) *m/z* calcd for $C_{11}H_{13}N_2O_2^+$ [(M + H)$^+$] 205.0972, found 205.0970 (Δ –0.7 ppm).

***(S)-2-(3-((S)-chroman-4-yl)-3-methylureido)-3-(2-cyanophenyl)propanoic acid* ((–)-18)**

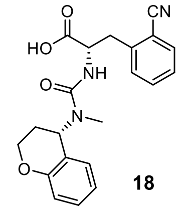

18

Chemical Formula: $C_{21}H_{21}N_3O_4$
Molecular Weight: 379.4

Step1: To a solution of **(+)-16** (200 mg, 0.98 mmol, 1 equiv.) in MeCN (3 mL) was added DIPEA (348 µL, 1.98 mmol, 2 equiv.) and the mixture was cooled to 0 ºC and stirred at that temperature for 10 min. Dipyridin-2-yl carbonate (440 mg, 2.0 mmol, 2 equiv.) was added and the mixture was stirred at 0 ºC for 1 h.

Step2: (*S*)-*N*-Methylchroman-4-amine **13** was freshly prepared by a previously reported selective monomethylation method (S3). To a solution of (*S*)-chroman-4-amine (free-amine, 200 mg, 1.34 mmol, 1 equiv.) in HFIP (1.4 mL, 13.4 mmol, 10 equiv.) was added methyl triflate (230 µL, 2.01 mmol, 1.5 equiv.) and the mixture was stirred at rt for exactly 1 h. The reaction mixture was quenched by adding 2N HCl (2 mL) and the volatiles were removed under reduced pressure. The mixture was neutralized by adding sat. $NaHCO_3$ and extracted with CH_2Cl_2 (3 × 5 mL). The combined organic layer was dried (Na_2SO_4) and concentrated under reduced pressure to afford the amine **13** as a pale yellow solid. The solid residue was dissolved in CH_2Cl_2 (3 mL) and added to the reaction mixture from step 1 and the mixture was stirred at rt for 16 h. The reaction mixture was concentrated under reduced pressure and purification by flash chromatography (SiO_2, EtOAc/hexanes gradient) afforded a mixture of the desired methyl ester **17** and a byproduct (total 208 mg). The byproduct was identified to be the dimer urea of **16**. Multiple attempts to separate the desired urea and the byproduct urea were unsuccessful and the mixture was taken for ester hydrolysis.

Step3: To the solution of this mixture in THF (5 mL) was added H_2O (5 mL) and the biphasic mixture was stirred vigorously at 0 ºC for 10 min. $LiOH·H_2O$ (68 mg, 1.62 mmol, ~3 equiv.) was added and the mixture was stirred at rt for 30 min. The layers were separated and to the aqueous layer was added 1N HCl dropwise to adjust the pH to ~3. The aqueous layer was extracted with EtOAC and the combined organic layer was dried (Na_2SO_4) and concentrated under reduced pressure. Purification by flash chromatography (SiO_2, $MeOH/CH_2Cl_2$ gradient) afforded the product **18** as a white foam (155 mg, 42% after 3 steps). R_f 0.30 (15% $MeOH/CH_2Cl_2$); $[\alpha]_D^{25}$ –129.8 (c 1.0, MeOH); ^{1}H NMR (600 MHz, CD_3OD) δ 1.97–1.93 (m, 1H), 2.12–2.06 (m, 1H), 2.55 (s, 3H), 3.26 (dd, J = 13.8, 10.0 Hz, 1H), 3.57 (dd, J = 13.9, 4.2 Hz, 1H), 4.11 (td, J = 11.4, 1.8 Hz, 1H), 4.29 (dt, J = 11.3, 3.7 Hz, 1H), 4.81 (dd, J = 9.4, 3.8 Hz, 1H), 5.49 (dd, J = 9.7, 6.5 Hz, 1H), 6.71 (br d, J = 7.0 Hz, 1H), 6.74 (d, J = 8.2 Hz, 1H), 6.82 (t, J = 7.4 Hz, 1H), 7.09 (app t, J = 7.9 Hz, 1H), 7.45 (t, J = 7.6 Hz, 1H), 7.53 (d, J = 7.8 Hz, 1H), 7.64 (t, J = 7.6 Hz, 1H), 7.73 (d, J = 7.4 Hz, 1H); ^{13}C NMR (151 MHz, CD_3OD) δ 27.8, 29.9, 37.8, 49.1, 51.4, 66.5, 114.4, 118.1, 119.2, 121.8, 123.1, 128.7, 128.9, 129.7, 132.2, 133.5, 134.1, 134.2, 143.5, 157.9, 160.6; HRMS (ESI+) *m/z* calcd for $C_{21}H_{22}N_3O_4^+$ [(M + H)$^+$] 380.1605, found 380.1608 (Δ 0.9 ppm).

***(3S,4R)-1-((S)-2-(3-((S)-chroman-4-yl)-3-methylureido)-3-(2-cyanophenyl)propanoyl)-4-(4-methoxyphenyl)-N-methylpyrrolidine-3-carboxamide* ((+)-19)**

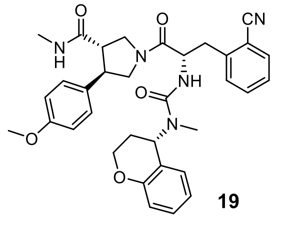

Chemical Formula: $C_{34}H_{37}N_5O_5$
Molecular Weight: 595.7

To a solution of **18** (29 mg, 0.077 mmol, 1 equiv.) in DMF (2 mL) was added HOAt/DIPEA mixture (1.54 mL, 100 mM each in MeCN, 2 equiv.), HATU (58 mg, 0.153 mmol, 2 equiv.), and a solution of **15** (29 mg, 0.123 mmol, 1.6 equiv.) in DMF (2 mL) and the mixture was stirred at rt for 16 h. The reaction mixture was concentrated under reduced pressure, dissolved in EtOAc (10 mL), and washed with sat. $NaHCO_3$ (10 mL) and brine. The organic layer was dried (Na_2SO_4) and concentrated under reduced pressure. Purification by flash chromatography (SiO_2, MeOH/CH_2Cl_2 gradient) afforded the coupled product **19** as a colorless oil (27 mg, 60%). R_f 0.45 (10% MeOH/CH_2Cl_2); $[\alpha]_D^{25}$ +22.8 (c 0.47, MeOH); ^{1}H NMR (600 MHz, CD_3OD, observed as a mixture of rotamers) δ 1.93–1.86 (m, 1H), 2.11–2.03 (m, 1H), 2.57 (s, 3H), 2.63–2.61 (four singlets, 3H), 3.01 (app q, *J* = 8.6 Hz, 0.61 H), 3.09 (app q, *J* = 7.9 Hz, 0.38 H), 3.24 (dd, *J* = 13.8, 9.2 Hz, 1H), 3.57–3.35 (m, 3H), 3.71 (t, *J* = 10.6 Hz, 0.64H), 3.77–3.76 (two singlets, 3H), 3.87 (t, *J* = 10.2 Hz, 0.4H), 3.97–3.92 (m, 1.2H), 4.04–4.00 (m, 0.8H), 4.13–4.07 (m, 1H), 4.29–4.26 (m, 1H), 4.99–4.95 (m, 0.62H), 5.10–5.07 (m, 0.38H), 5.47–5.44 (m, 1H), 6.54 (br dd, *J* = 19.2, 7.2 Hz, 0.5H), 6.66 (br dd, *J* = 20.0, 6.9 Hz, 0.5H), 6.75 (d, *J* = 8.2 Hz, 1H), 6.89–6.81 (m, 3H), 7.10 (app t, *J* = 7.7 Hz, 1H), 7.19–7.17 (m, 2H), 7.50–7.47 (m, 1H), 7.55 (d, *J* = 7.5 Hz, 1H), 7.66 (app t, *J* = 7.6 Hz, 1H), 7.75 (d, *J* = 7.7 Hz, 1H), 7.96–7.90 (m, 0.7H); ^{13}C NMR (151 MHz, CD_3OD, observed as a mixture of rotamers) δ 26.5, 27.8, 30.0, 37.7, 37.8, 47.4, 50.9, 51.3, 51.5, 53.7, 53.9, 54.0, 54.3, 54.5, 55.8, 66.4, 66.5, 114.4, 115.0, 115.3, 115.4, 118.2, 119.3, 119.4, 121.8, 122.9, 123.1, 128.7, 128.8, 128.9, 129.1, 129.6, 129.7, 129.8, 131.3, 131.8, 132.6, 132.7, 134.1, 134.2, 134.22, 134.3, 142.7, 142.8, 157.9, 160.0, 160.3, 160.6, 160.7, 171.9, 172.3, 173.4, 174.0; HRMS (ESI+) *m/z* calcd for $C_{34}H_{38}N_5O_5^+$ [(M + H)$^+$] 596.2867, found 596.2865 (Δ –0.3 ppm).

***(3S,4R)-1-((S)-2-(3-((S)-chroman-4-yl)-3-methylureido)-3-(2-(guanidinomethyl)phenyl)propanoyl)-4-(4-methoxyphenyl)-N-methylpyrrolidine-3-carboxamide* ((+)-5)**

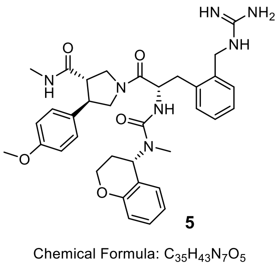

Chemical Formula: $C_{35}H_{43}N_7O_5$
Molecular Weight: 641.8

To a solution of **19** (18 mg, 0.030 mmol, 1 equiv.) in MeOH (4 mL) was added Raney Ni (25 µL, 50% wet in H_2O) followed by a freshly prepared solution of $NaBH_4$ (114 mg, 3.0 mmol, 100 equiv.) in MeOH (1 mL) and the mixture was stirred at rt for 1 h. The reaction mixture was filtered through a short pad of Celite and concentrated under reduced pressure. The residue was fractionated between H_2O and CH_2Cl_2. The organic layer was dried, evaporated and dissolved in MeCN (2 mL) and DIPEA (16 µL, 0.09 mmol, 3 equiv.) and 1*H*-pyrazole-1-carboximidamide hydrochloride (13 mg, 0.09 mmol, 3 equiv.) were added and the mixture was stirred at rt for 16 h. The reaction mixture was concentrated under reduced pressure. Purification by C-18 reverse phase chromatography (10% MeCN/0.1% FA in H_2O to 100% MeCN) afforded the product **5** as a white solid (12 mg, 62%) as a formate salt. $[\alpha]_D^{25}$ +30.0 (c 1.0, 9:1 MeOH/DMSO); ^{1}H NMR (600 MHz, CD_3OD, observed as a mixture of rotamers) δ 1.95–1.88 (m, 1H), 2.13–2.07 (m, 1H), 2.61–2.57 (three singlets, 6H), 2.82 (app q, *J* = 9.7 Hz, 0.36 H), 2.96 (app q, *J* = 9.4 Hz, 0.72 H), 3.14–3.08 (m, 1.7H), 3.54–3.21 (m, 5.3H), 2.77–2.75 (two singlets, 3H), 3.87 (dd, *J* = 12.2, 8.3 Hz, 0.8H), 3.98 (dd, *J* = 12.0, 8.0 Hz, 0.3H), 4.14–4.09 (m, 1H), 4.33–4.29 (m, 1H), 4.55 (ABq, Δδ = 60.9 Hz, *J* = 15.1 Hz, 1.5H), 4.62 (ABq, Δδ = 96.6 Hz, *J* = 15.4 Hz, 1.5H), 4.81 (submerged in H_2O peak, 0.63H), 5.03 (app t, *J* = 8.0 Hz, 0.37H), 5.52–5.49 (m, 1H), 6.57–

6.55 (m, 0.27H), 6.69–6.67 (m, 0.76H), 6.76–6.74 (m, 1H), 6.88–6.82 (m, 3H), 7.12–7.07 (m, 3H), 7.47–7.34 (m, 4H), 8.44 (br s, 1.3H, formate ion); ^{13}C NMR (151 MHz, CD_3OD, observed as a mixture of rotamers) δ 26.5, 27.8, 30.0, 36.2, 36.3, 43.9, 44.0, 47.5, 50.7, 51.0, 51.5, 51.6, 53.8, 53.9, 54.3, 54.4, 55.8, 66.4, 66.5, 115.3, 115.4, 118.2, 118.3, 121.8, 122.9, 128.7, 128.8, 128.9, 129.0, 129.1, 129.3, 129.4, 129.5, 129.6, 129.7, 129.8, 129.9, 131.1, 131.6, 133.0, 133.1, 136.1, 136.2, 136.7, 136.9, 157.8, 157.9, 158.8, 160.2, 160.4, 160.7, 160.8, 168.6, 172.3, 172.6, 173.4, 174.1; HRMS (ESI+) *m/z* calcd for $C_{35}H_{44}N_7O_5^+$ [(M + H)$^+$] 642.3398, found 642.3385 (Δ –2.0 ppm).

6e) *In-vitro* assay of hits with thrombin. A thrombin inhibitor screening kit (Sigma-Aldrich cat # MAK243) was used according to the manufacturer's instructions to measure the activity of thrombin in the presence and absence of inhibitors. The kit uses the ability of thrombin to cleave a synthetic AMC-based peptide (AMC = 7-amino-4-methylcoumarin) to release AMC. Upon release AMC becomes fluorescent which can be measured (λex = 350 nm, λem = 450 nm). The loss of fluorescence intensity due to inhibition of thrombin was measured in kinetic mode every 30 seconds for 10 minutes, and the linear part of the progress curves was used to obtain fractional activity for each curve. Apparent K_i values were calculated as described previously (S4).

References:

S1. H.-C. Du, N. Simmons, J. C. Faver, Z. Yu, M. Palaniappan, K. Riehle, M. M. Matzuk, A Mild, DNA-compatible nitro reduction using $B_2(OH)_4$. *Org. Lett.* **21**, 2194−2199 (2019).

S2. J. C. Faver, K. Riehle, D. R. Lancia, J. B. J. Milbank, C. S. Kollmann, N. Simmons, Z. Yu, M. M. Matzuk, Quantitative comparison of enrichment from DNA-encoded chemical library selections. *ACS Comb. Sci.* **21**, 75–82 (2019).

S3. T. Lebleu, X. Ma, J. Maddaluno, J. Legros, Selective monomethylation of primary amines with simple electrophiles. *Chem. Commun.* **50**, 1836–1838 (2014).

S4. P. Kuzmic, K. C. Elrod, L. M. Cregar, S. Sideris, R. Rai, J. W. Janc, High-throughput Screening of Enzyme Inhibitors: Simultaneous Determination of Tight-Binding Inhibition Constants and Enzyme Concentration. *Anal Biochem.* **286**, 45–50 (2000).

7. Representative LC/MS Traces, Mass and NMR spectra

7a) DNA-HP synthesis

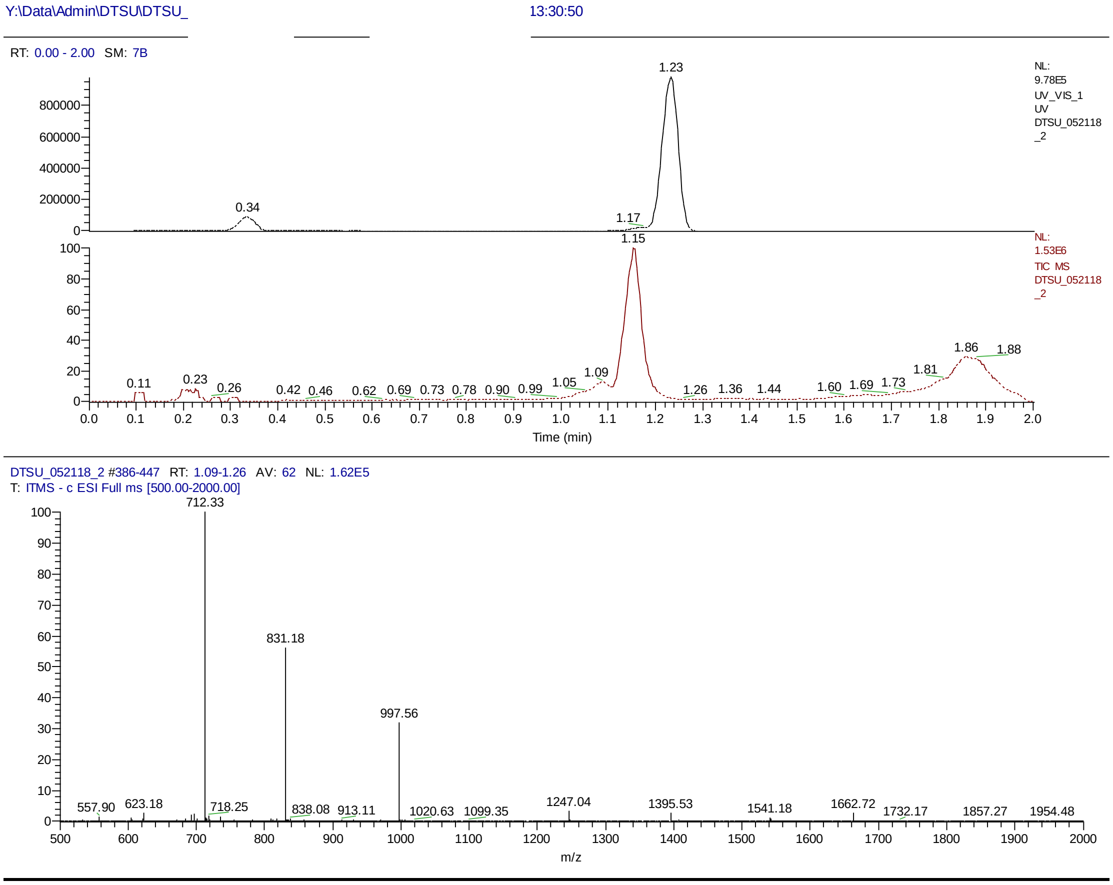

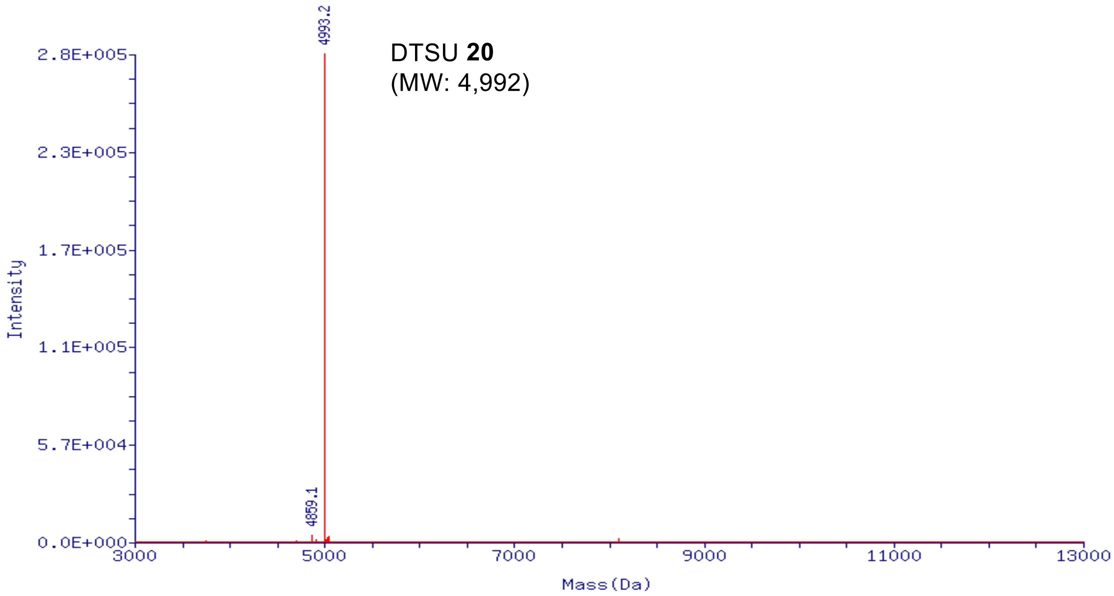

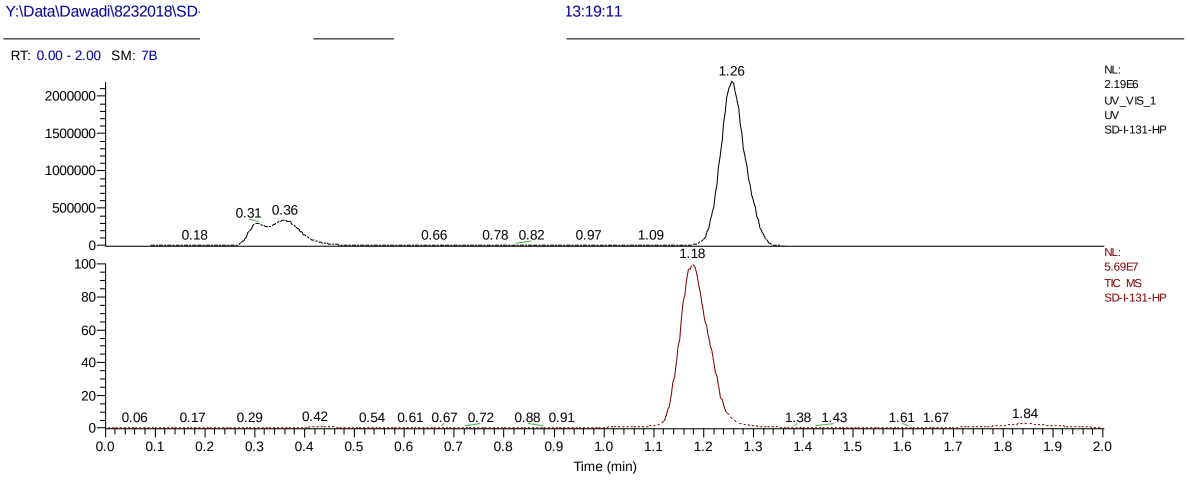
Y:\Data\Dawadi\8232018\SD-
13:19:11
RT: 0.00 - 2.00 SM: 7B
NL:
2.19E6
UV_VIS_1
UV
SD-I-131-HP
NL:
5.69E7
TIC MS
SD-I-131-HP
Time (min)

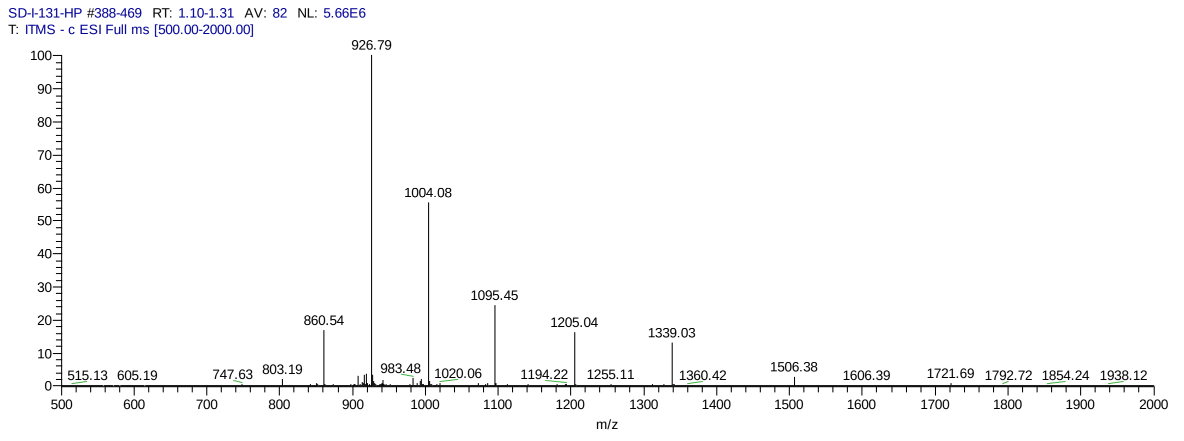
SD-I-131-HP #388-469 RT: 1.10-1.31 AV: 82 NL: 5.66E6
T: ITMS - c ESI Full ms [500.00-2000.00]
926.79
1004.08
1095.45
1205.04
1339.03
860.54
m/z

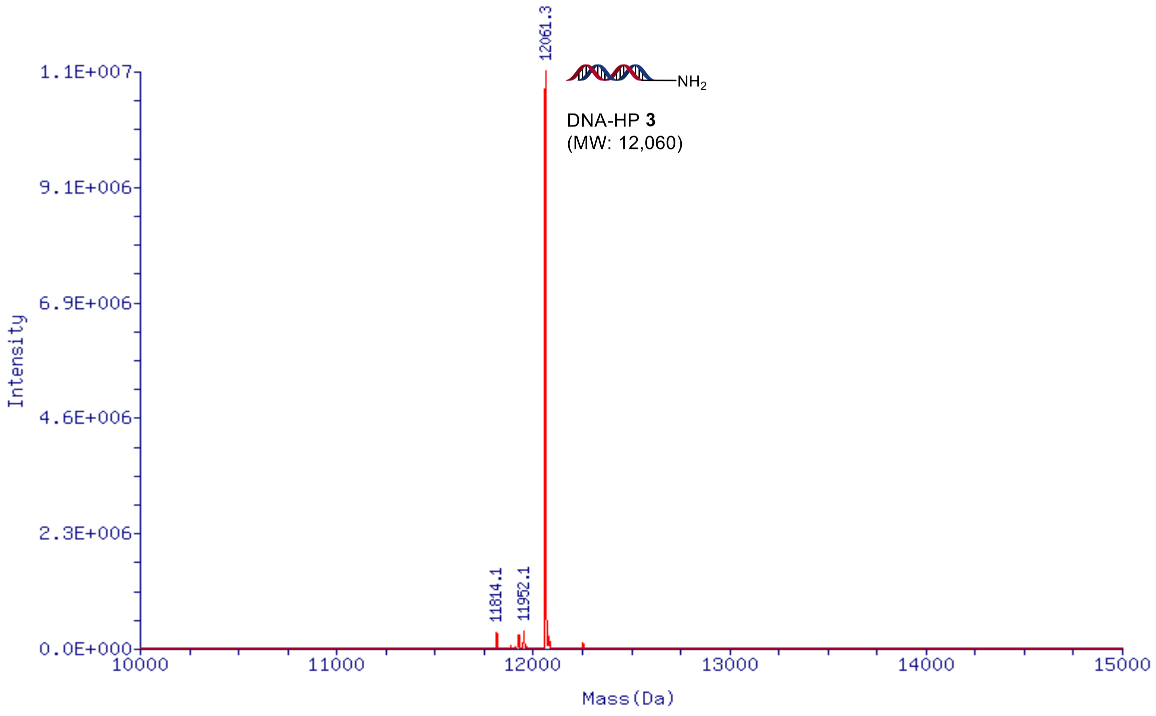
12061.3
11814.1
11952.1
NH2
DNA-HP 3
(MW: 12,060)
Intensity
Mass(Da)

7b) Substrate preparation and guanidine formation

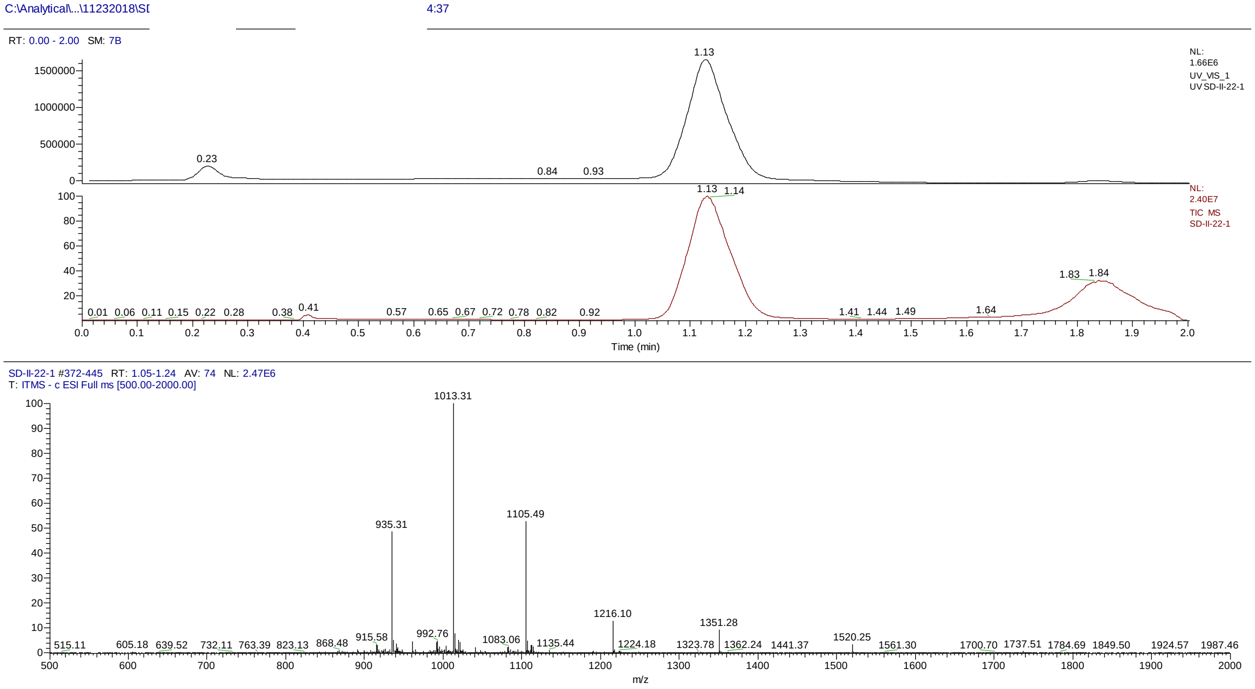

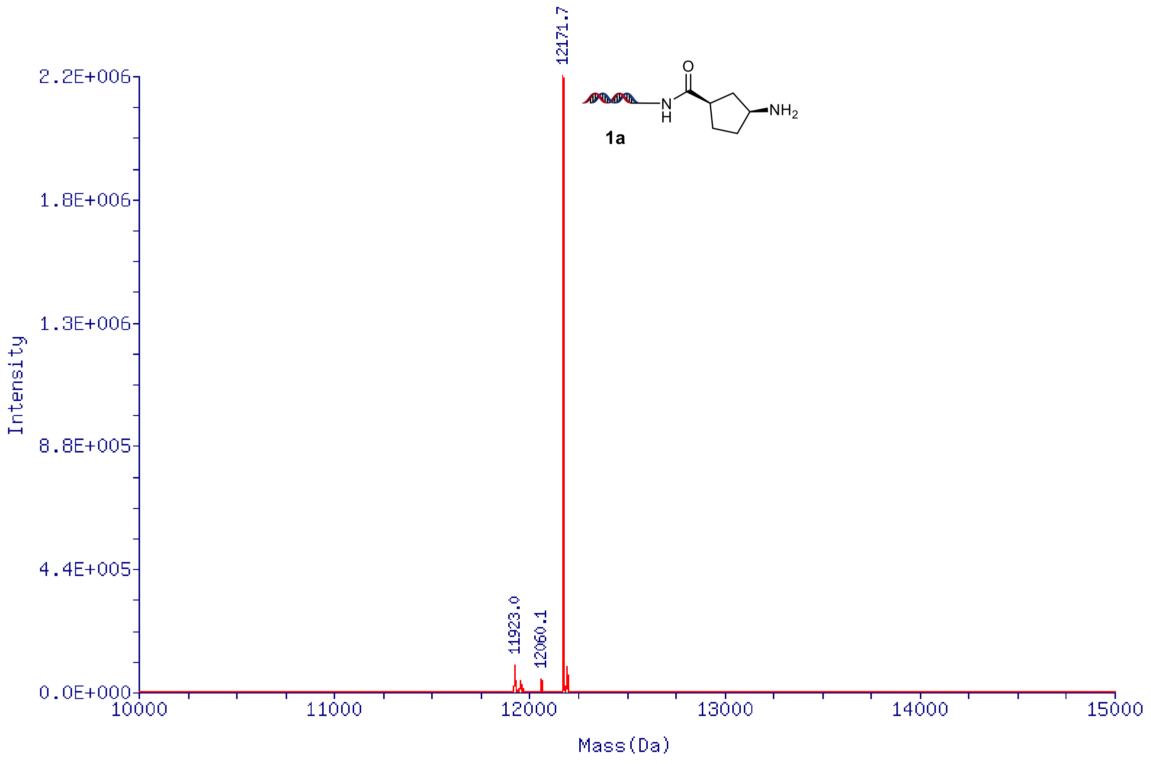

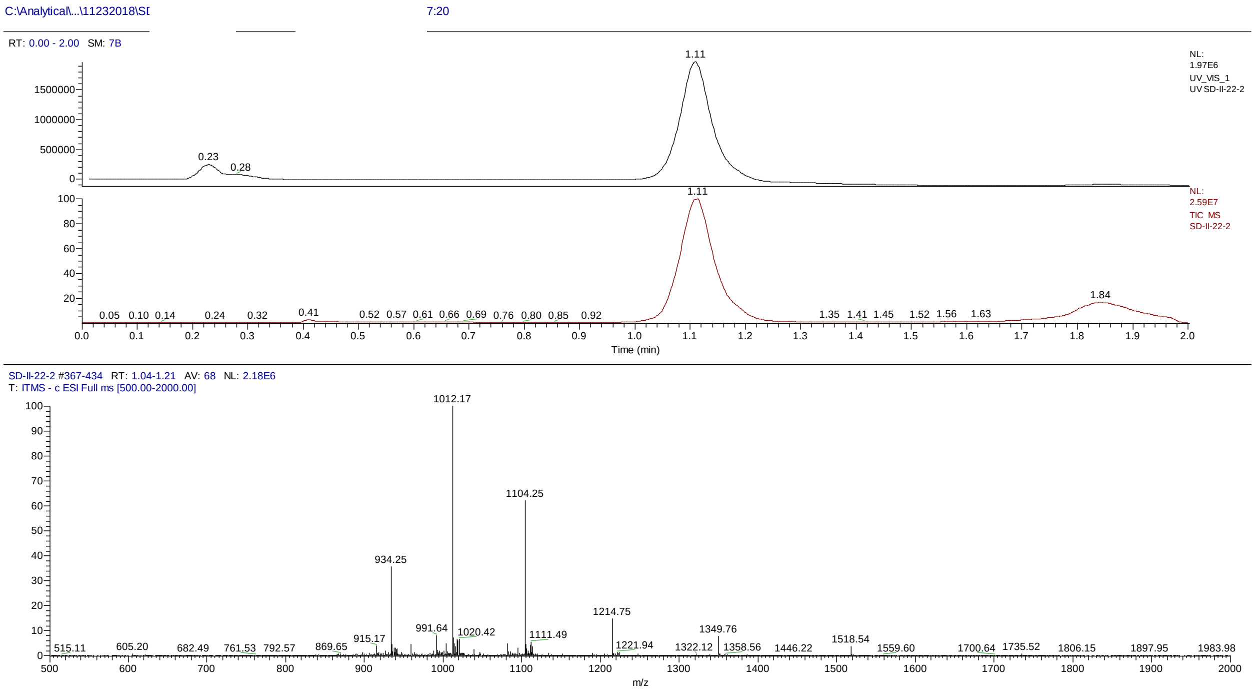
C:\Analytical\...\11232018\SI
7:20
RT: 0.00 - 2.00 SM: 7B
NL: 1.97E6 UV_VIS_1 UV SD-II-22-2
NL: 2.59E7 TIC MS SD-II-22-2
Time (min)
SD-II-22-2 #367-434 RT: 1.04-1.21 AV: 68 NL: 2.18E6
T: ITMS - c ESI Full ms [500.00-2000.00]
1012.17
1104.25
934.25
m/z

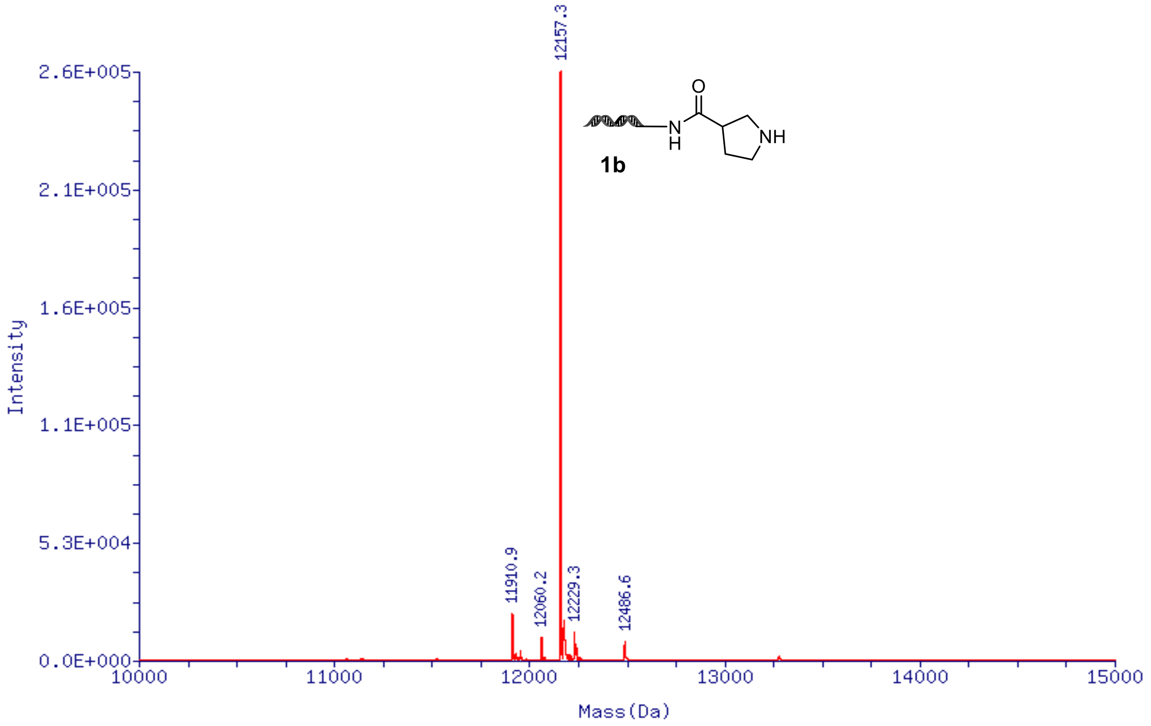
12157.3
11910.9
12060.2
12229.3
12486.6
Intensity
Mass(Da)
1b

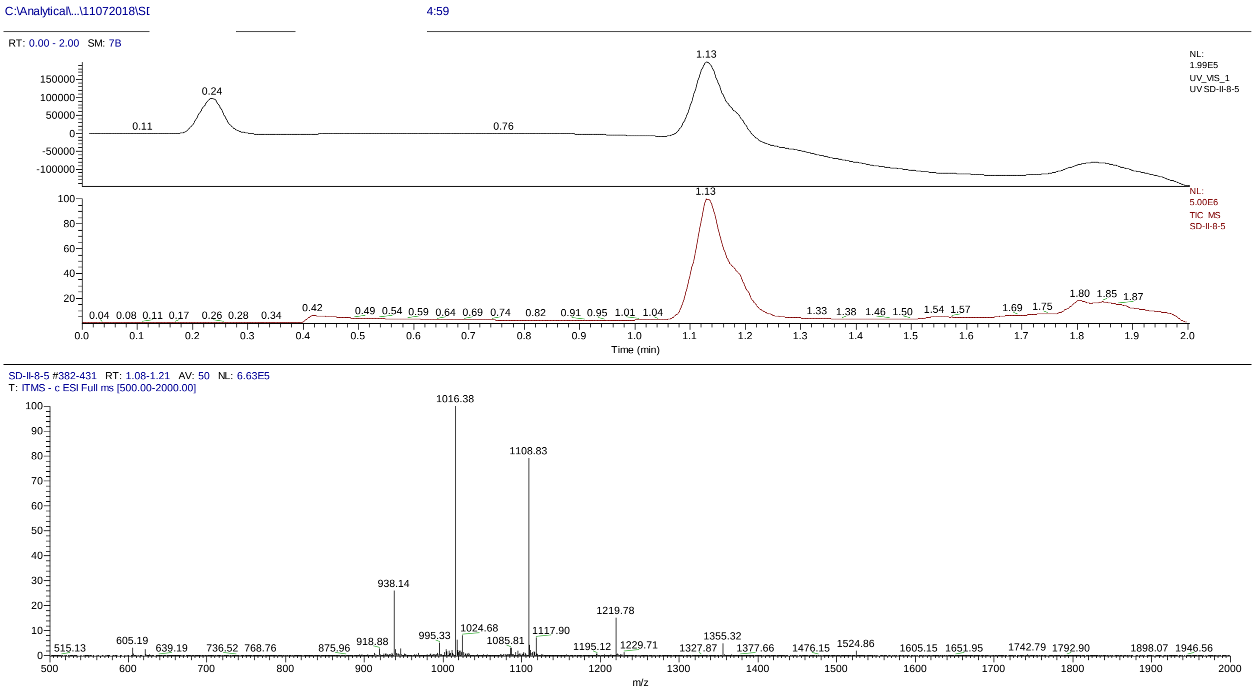
C:\Analytical\...\11072018\SI
4:59
RT: 0.00 - 2.00 SM: 7B
NL: 1.99E5 UV_VIS_1 UV SD-II-8-5
NL: 5.00E6 TIC MS SD-II-8-5
Time (min)
SD-II-8-5 #382-431 RT: 1.08-1.21 AV: 50 NL: 6.63E5
T: ITMS - c ESI Full ms [500.00-2000.00]
1016.38
1108.83
938.14
1219.78
m/z

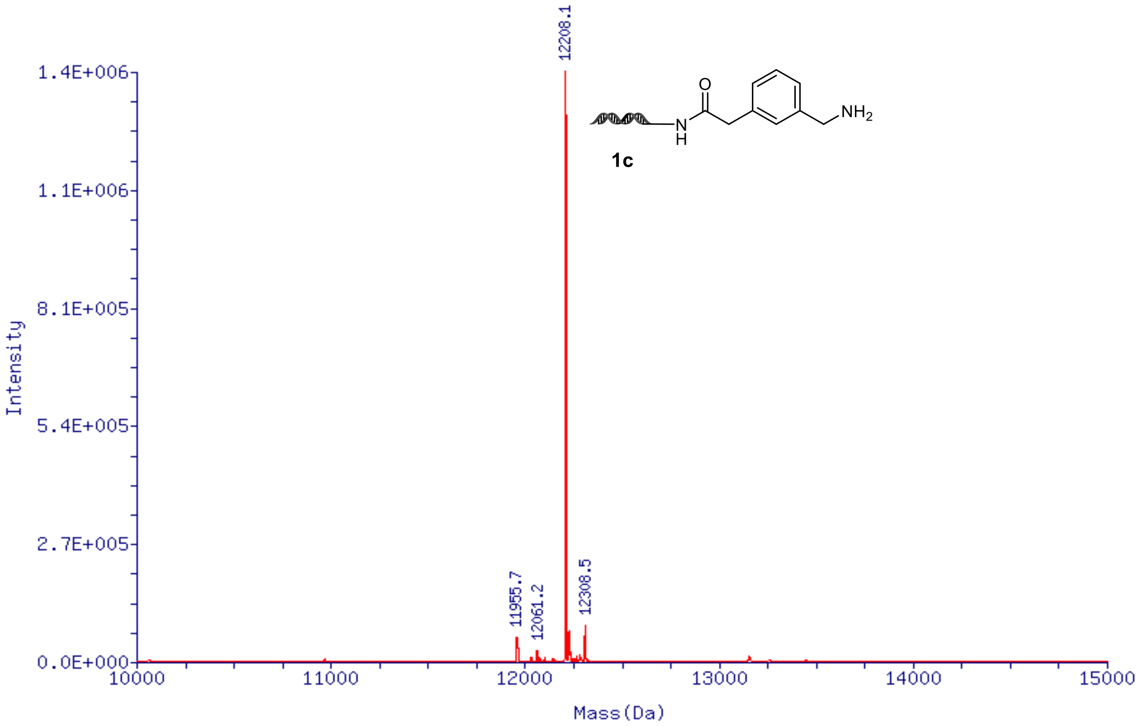
12208.1
11955.7
12061.2
12308.5
Intensity
Mass(Da)
NH2
1c

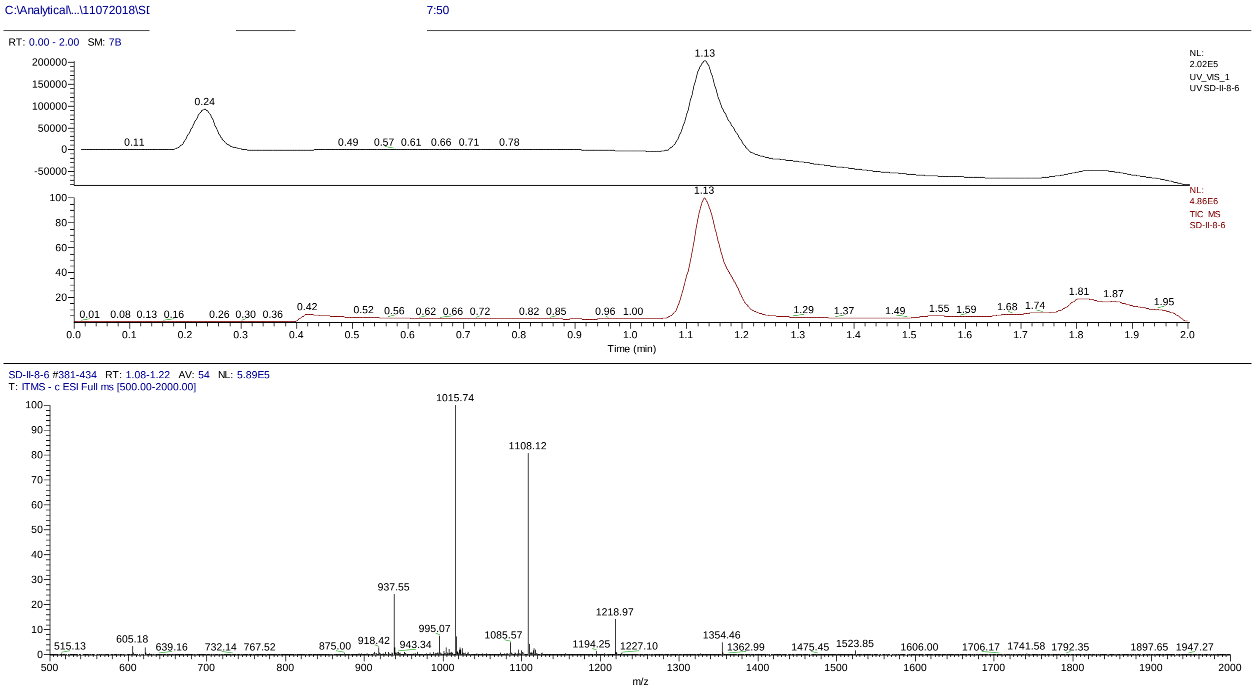

C:\Analytical\...\11072018\SD
7:50
RT: 0.00 - 2.00 SM: 7B
NL: 2.02E5 UV_VIS_1 UV SD-II-8-6
NL: 4.86E6 TIC MS SD-II-8-6
Time (min)
SD-II-8-6 #381-434 RT: 1.08-1.22 AV: 54 NL: 5.89E5
T: ITMS - c ESI Full ms [500.00-2000.00]
1015.74
1108.12
937.55
1218.97
995.07
1085.57
1354.46
m/z

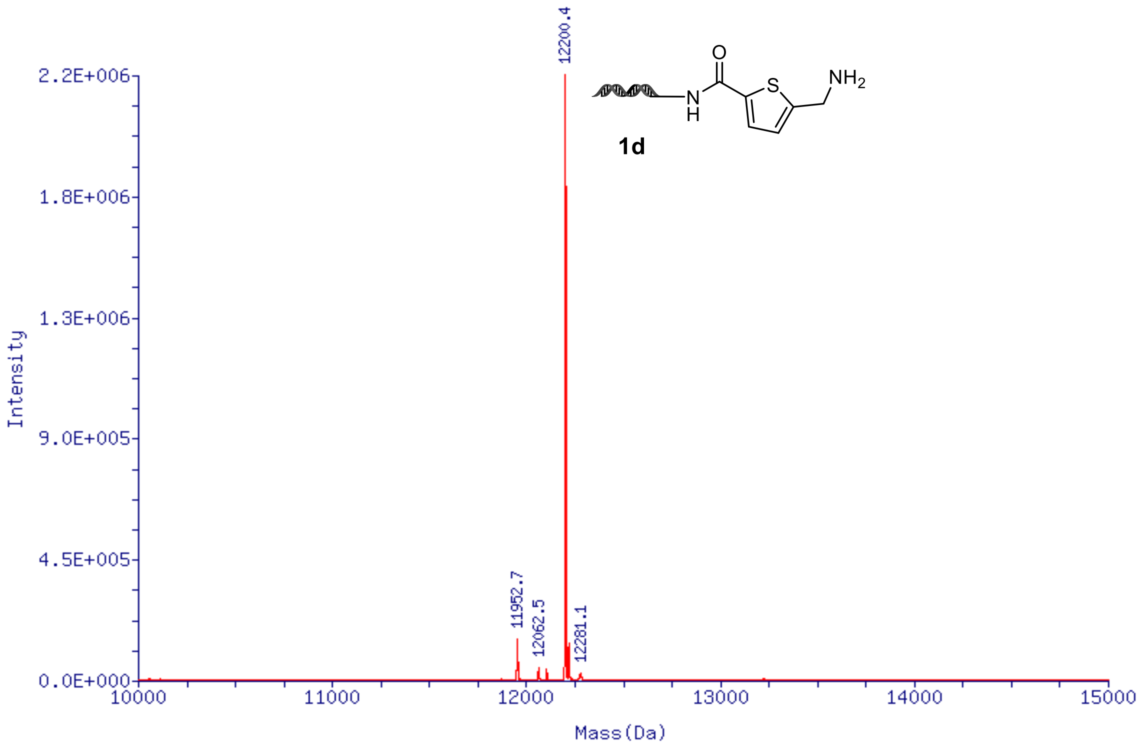

12200.4
11952.7
12062.5
12281.1
Intensity
Mass(Da)
1d

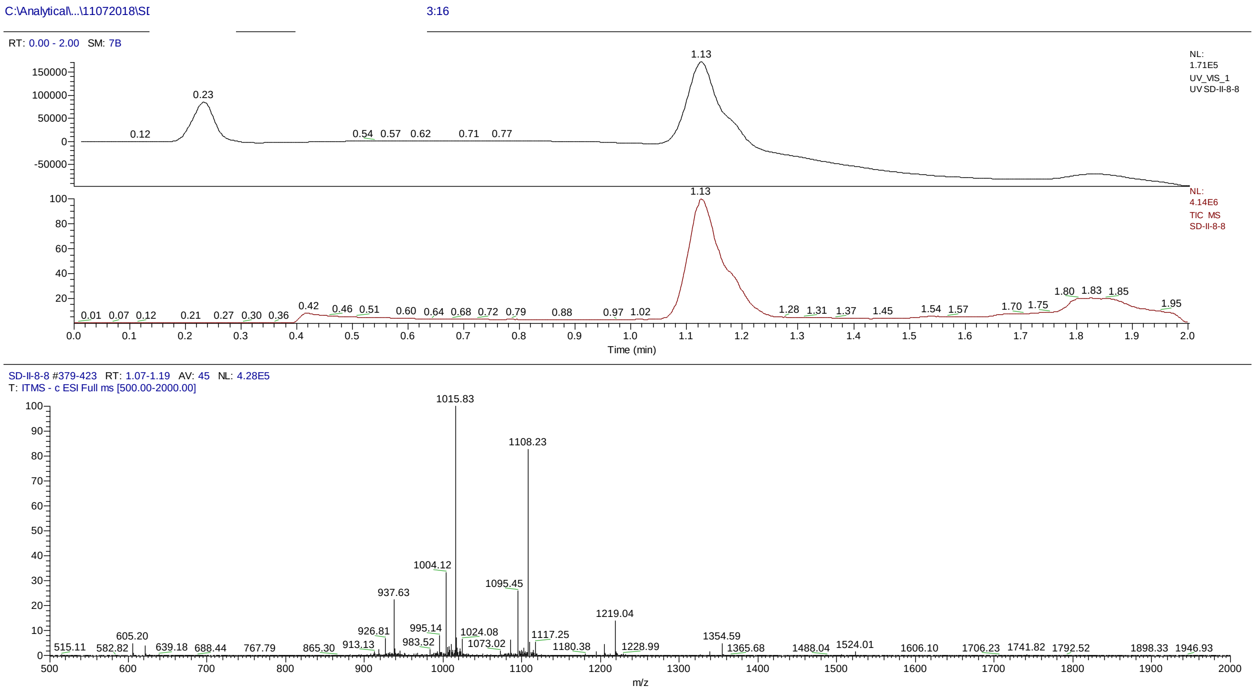
C:\Analytical\...\11072018\SI
3:16
RT: 0.00 - 2.00 SM: 7B
NL: 1.71E5 UV_VIS_1 UV SD-II-8-8
NL: 4.14E6 TIC MS SD-II-8-8
Time (min)
SD-II-8-8 #379-423 RT: 1.07-1.19 AV: 45 NL: 4.28E5
T: ITMS - c ESI Full ms [500.00-2000.00]
1015.83
1108.23
1004.12
1095.45
937.63
1219.04
m/z

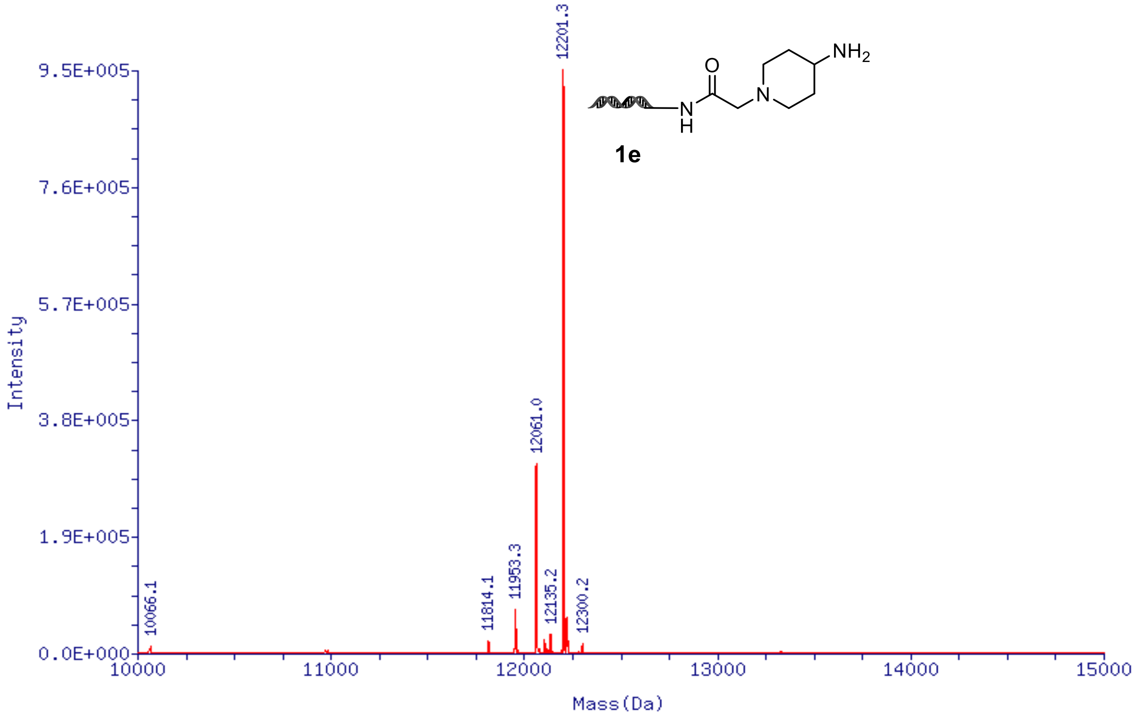
12201.3
12061.0
11953.3
11814.1
12135.2
12300.2
10066.1
Intensity
Mass(Da)
NH2
O
N
N
H
1e

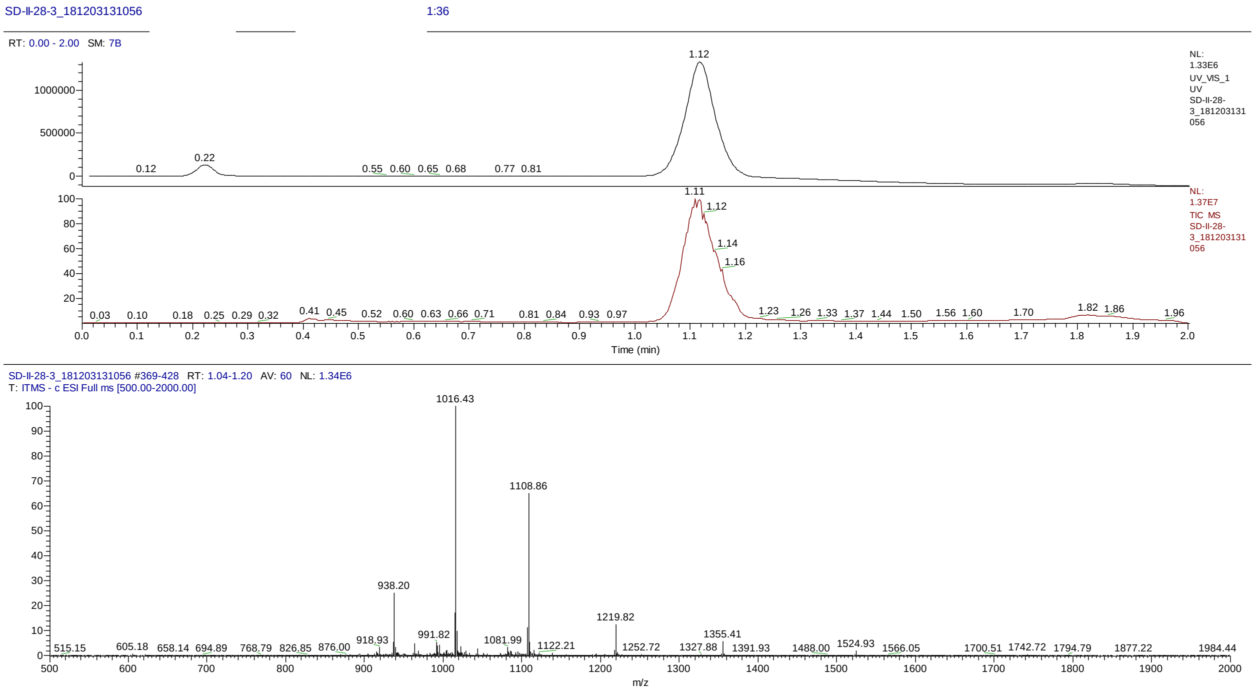
SD-II-28-3_181203131056
1:36
RT: 0.00 - 2.00 SM: 7B
NL: 1.33E6 UV_VIS_1 UV SD-II-28-3_181203131056
NL: 1.37E7 TIC MS SD-II-28-3_181203131056
Time (min)
SD-II-28-3_181203131056 #369-428 RT: 1.04-1.20 AV: 60 NL: 1.34E6
T: ITMS - c ESI Full ms [500.00-2000.00]
1016.43
1108.86
938.20
1219.82
1355.41
m/z

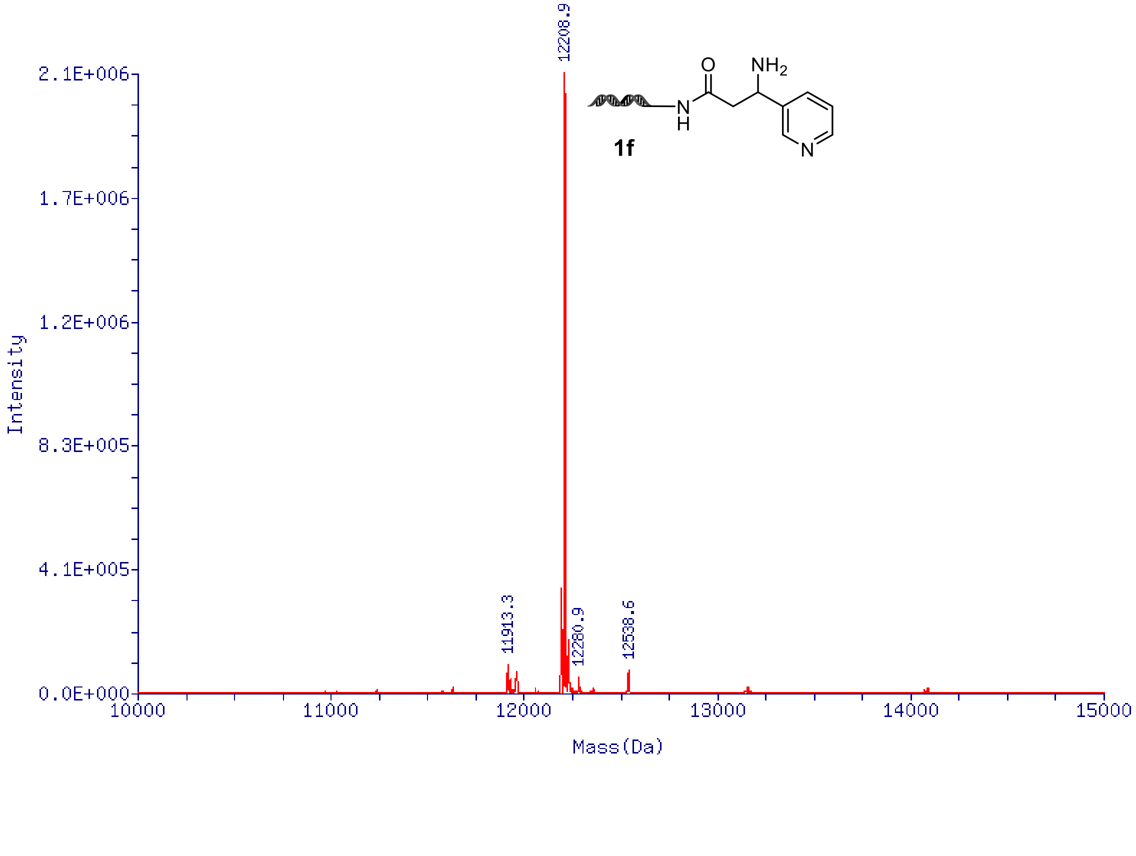
12208.9
11913.3
12280.9
12538.6
Intensity
Mass(Da)
1f

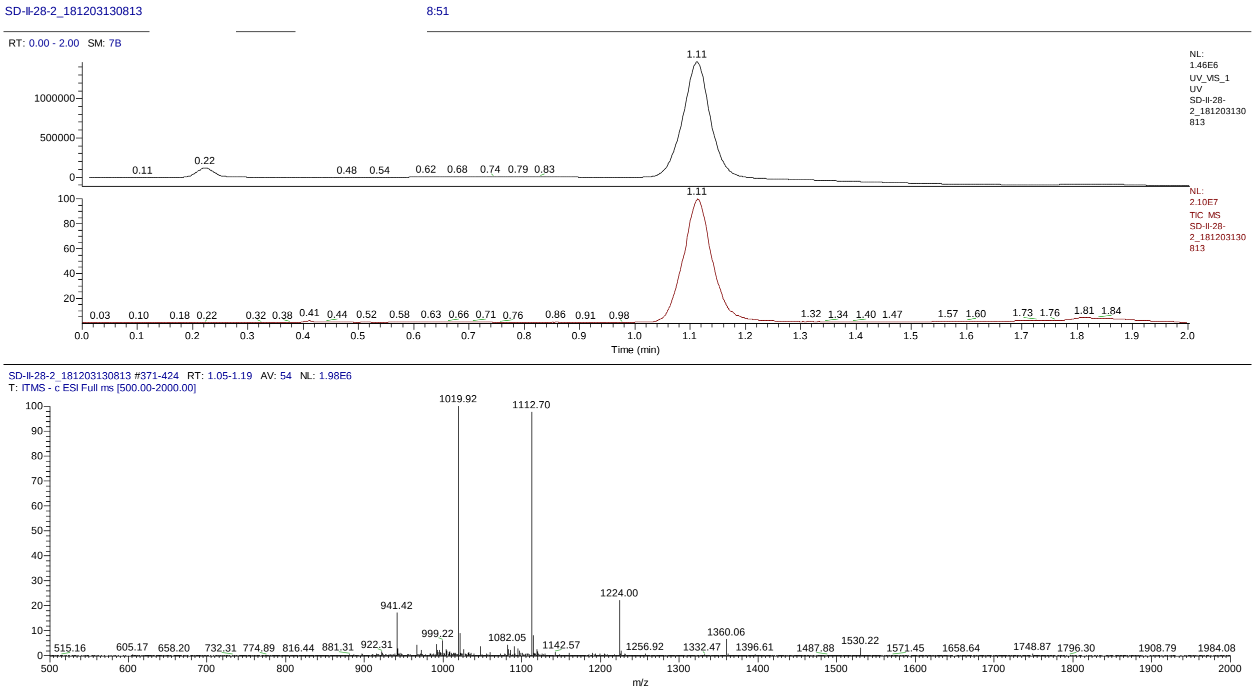
SD-II-28-2_181203130813
8:51
RT: 0.00 - 2.00 SM: 7B
NL: 1.46E6 UV_VIS_1 UV SD-II-28-2_181203130813
NL: 2.10E7 TIC MS SD-II-28-2_181203130813
Time (min)
SD-II-28-2_181203130813 #371-424 RT: 1.05-1.19 AV: 54 NL: 1.98E6
T: ITMS - c ESI Full ms [500.00-2000.00]
1019.92
1112.70
1224.00
941.42
1360.06
m/z

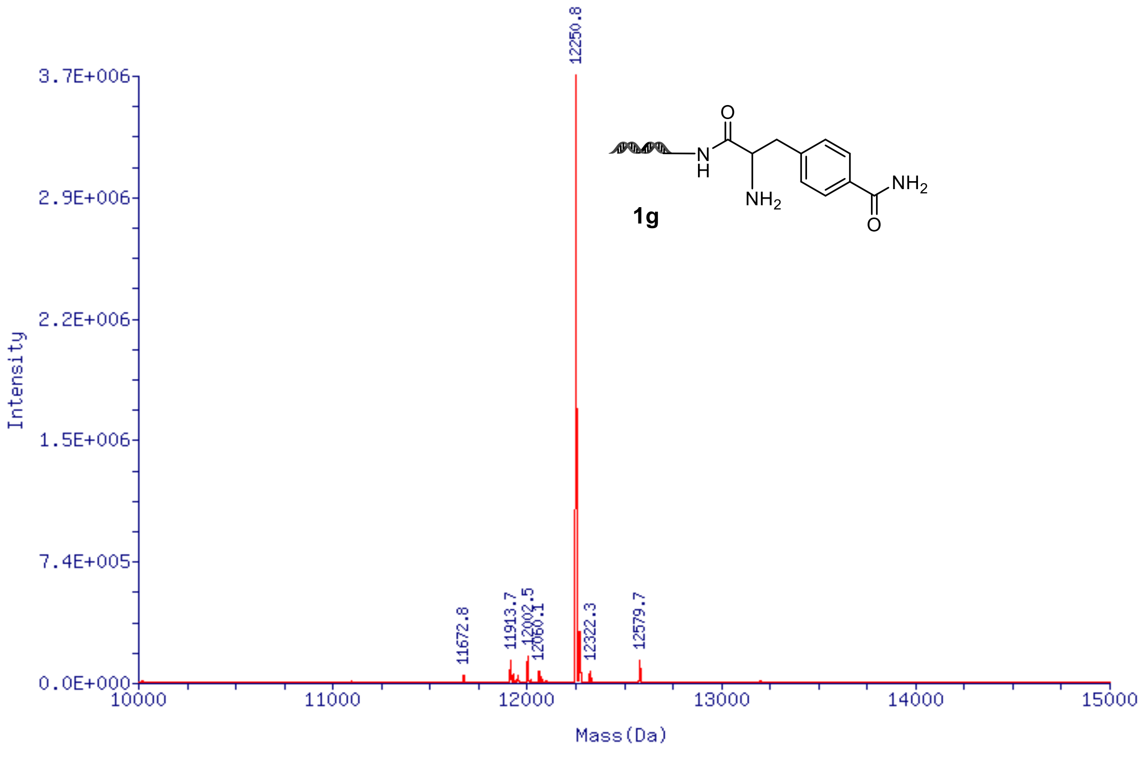
12250.8
11672.8
11913.7
12002.5
12060.1
12322.3
12579.7
Intensity
Mass(Da)
1g

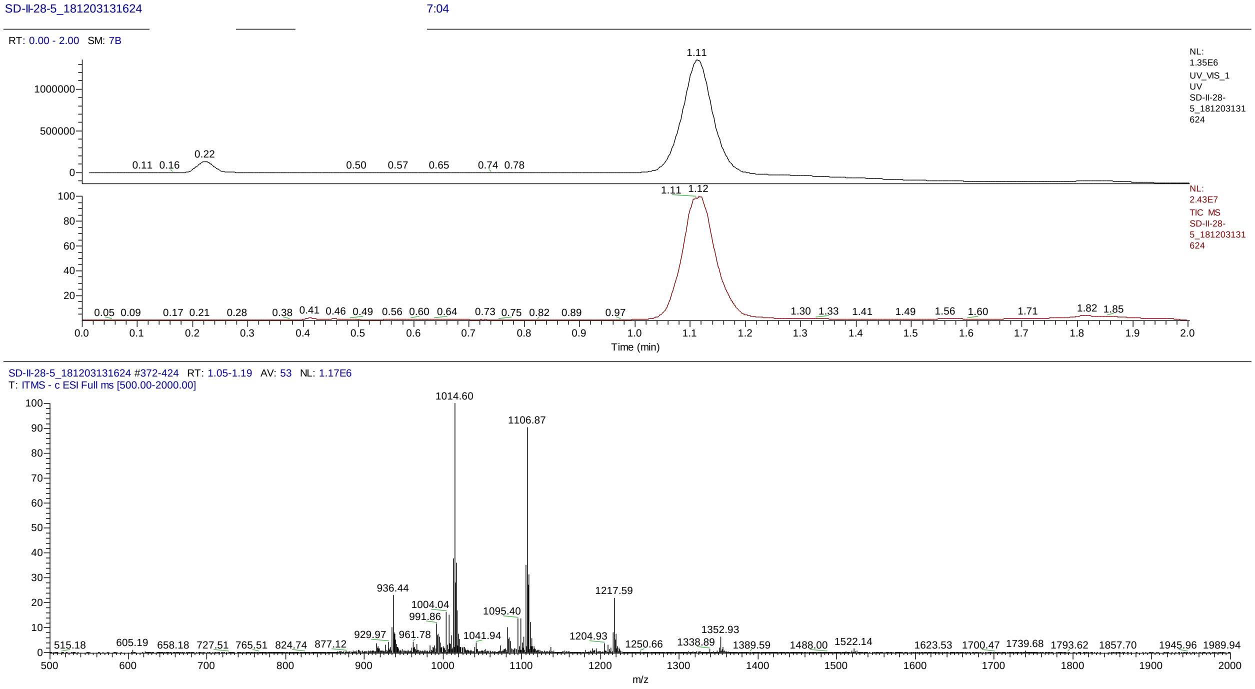
SD-II-28-5_181203131624
7:04
RT: 0.00 - 2.00 SM: 7B
NL: 1.35E6 UV_VIS_1 UV SD-II-28-5_181203131624
NL: 2.43E7 TIC MS SD-II-28-5_181203131624
Time (min)
SD-II-28-5_181203131624 #372-424 RT: 1.05-1.19 AV: 53 NL: 1.17E6
T: ITMS - c ESI Full ms [500.00-2000.00]
1014.60
1106.87
1217.59
936.44
m/z

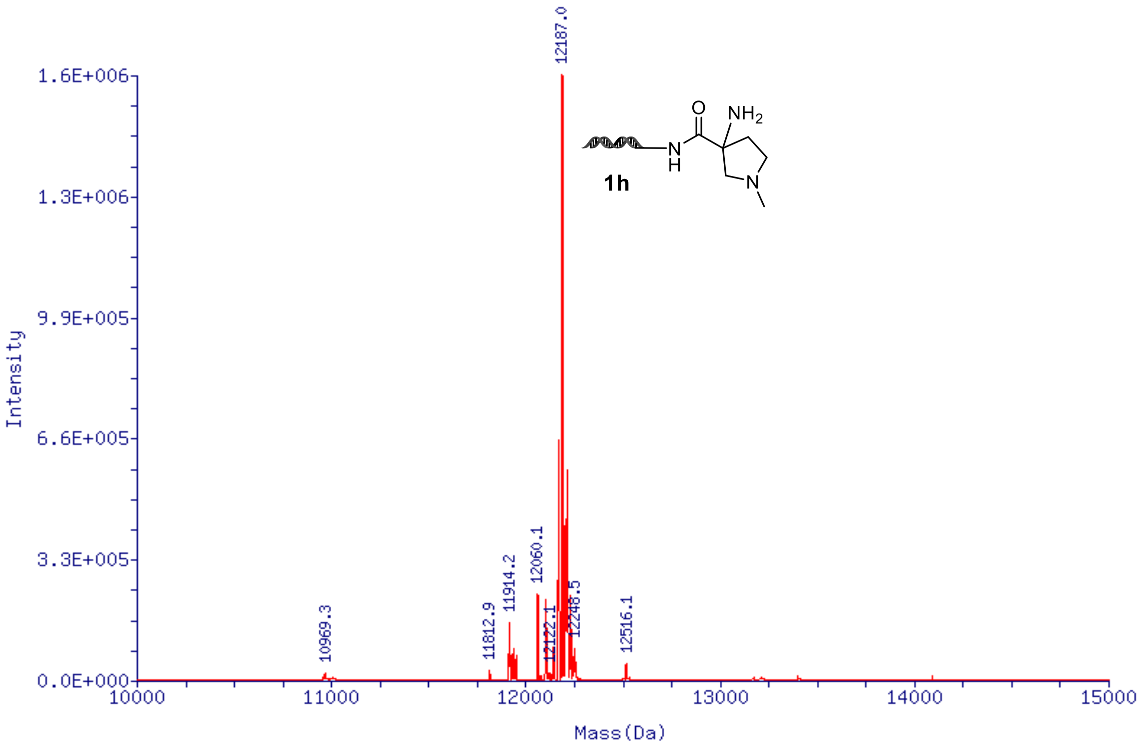
12187.0
Intensity
Mass(Da)
1h
NH2
O
N
H

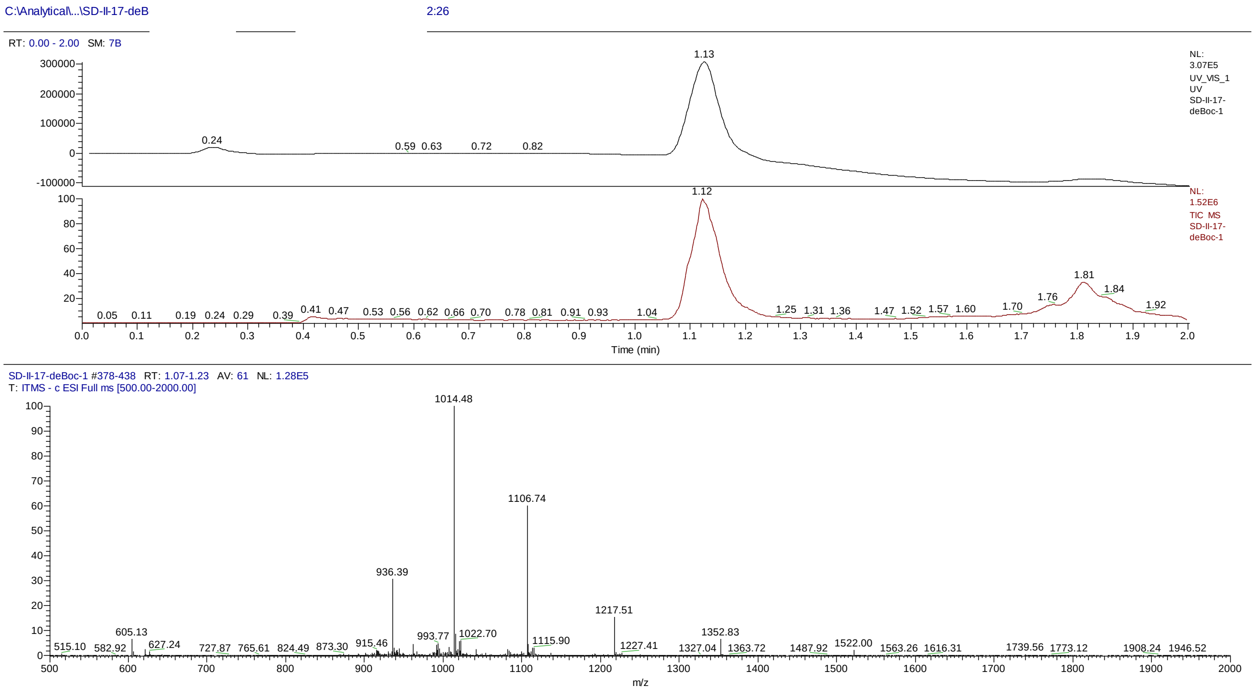
C:\Analytical\...\SD-II-17-deB
2:26
RT: 0.00 - 2.00 SM: 7B
NL: 3.07E5 UV_VIS_1 UV SD-II-17-deBoc-1
NL: 1.52E6 TIC MS SD-II-17-deBoc-1
Time (min)
SD-II-17-deBoc-1 #378-438 RT: 1.07-1.23 AV: 61 NL: 1.28E5
T: ITMS - c ESI Full ms [500.00-2000.00]
1014.48
1106.74
936.39
1217.51
1352.83
m/z

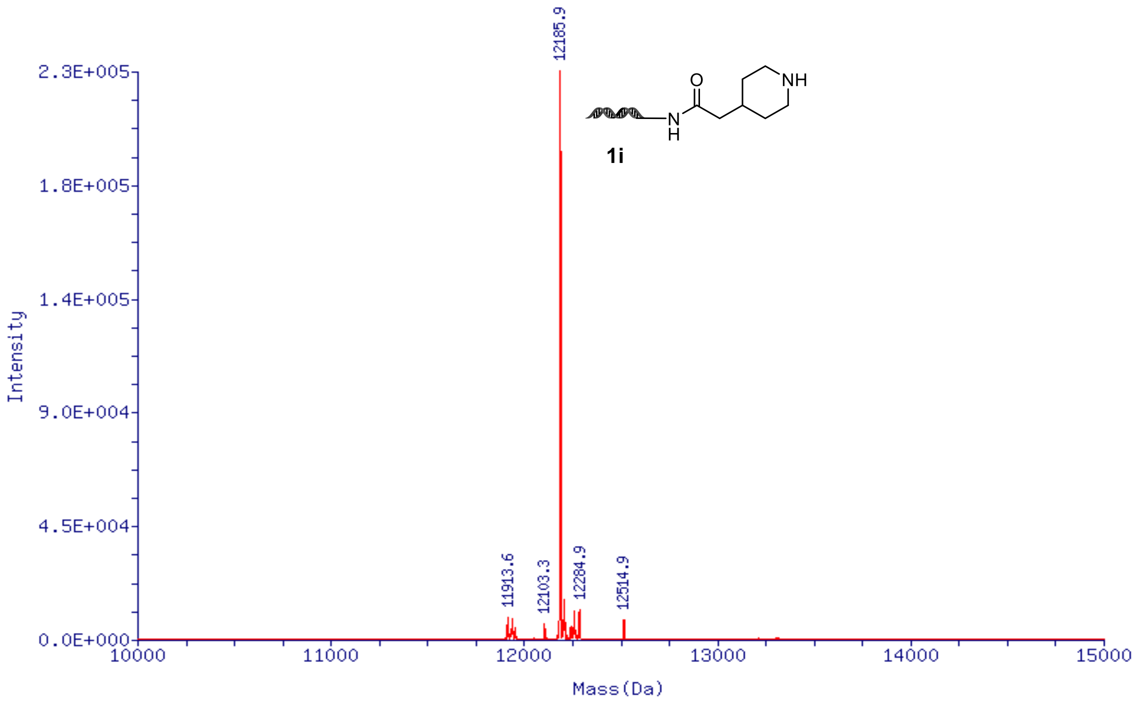
12185.9
11913.6
12103.3
12284.9
12514.9
Intensity
Mass(Da)
1i

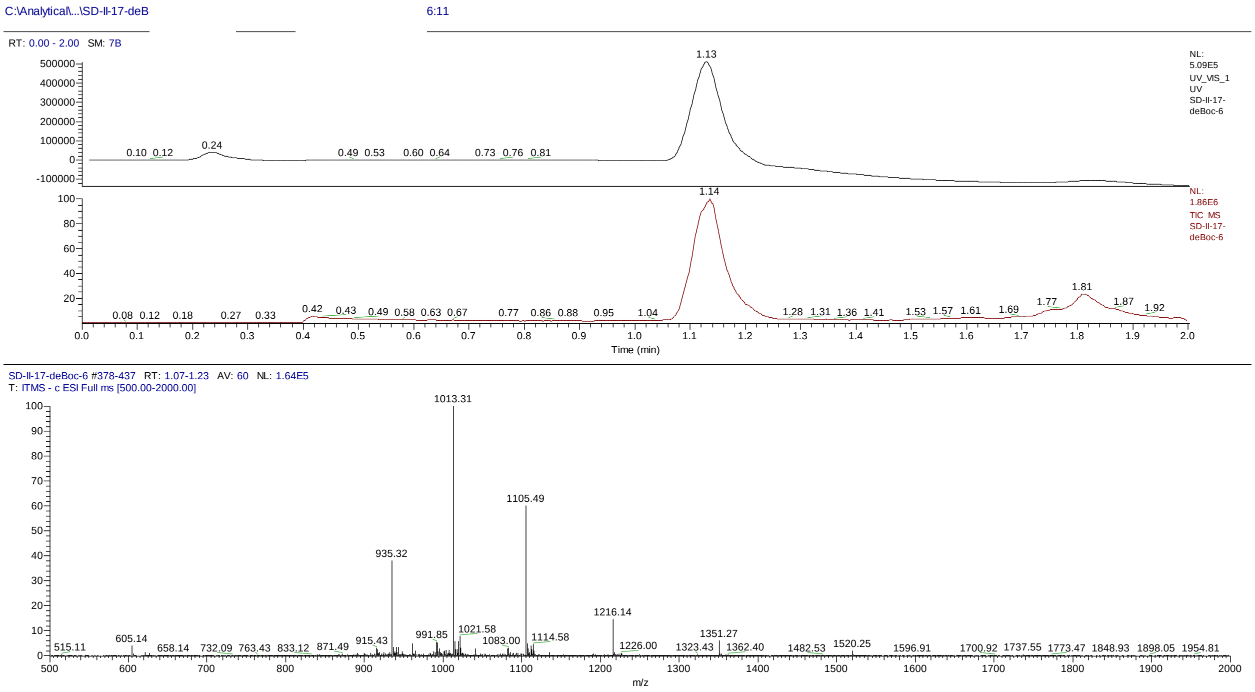
C:\Analytical\...\SD-II-17-deB
6:11
RT: 0.00 - 2.00 SM: 7B
NL: 5.09E5 UV_VIS_1 UV SD-II-17-deBoc-6
NL: 1.86E6 TIC MS SD-II-17-deBoc-6
Time (min)
SD-II-17-deBoc-6 #378-437 RT: 1.07-1.23 AV: 60 NL: 1.64E5
T: ITMS - c ESI Full ms [500.00-2000.00]
1013.31
1105.49
935.32
1216.14
1351.27
m/z

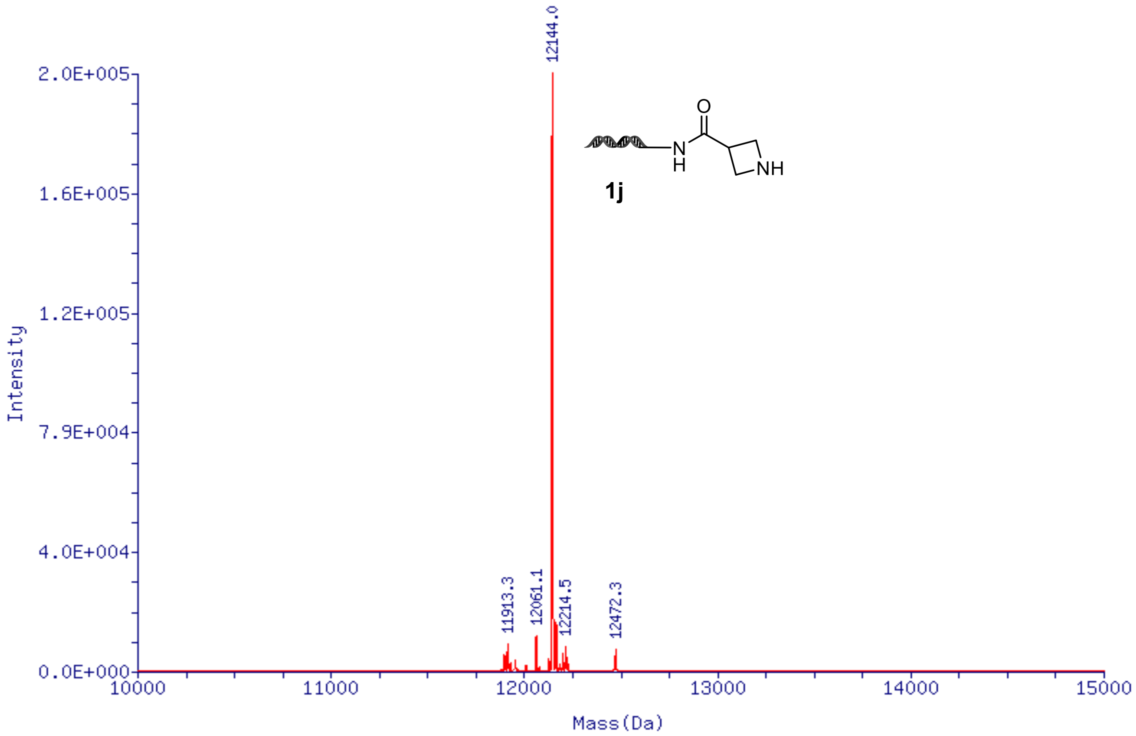
12144.0
11913.3
12061.1
12214.5
12472.3
Intensity
Mass(Da)
1j

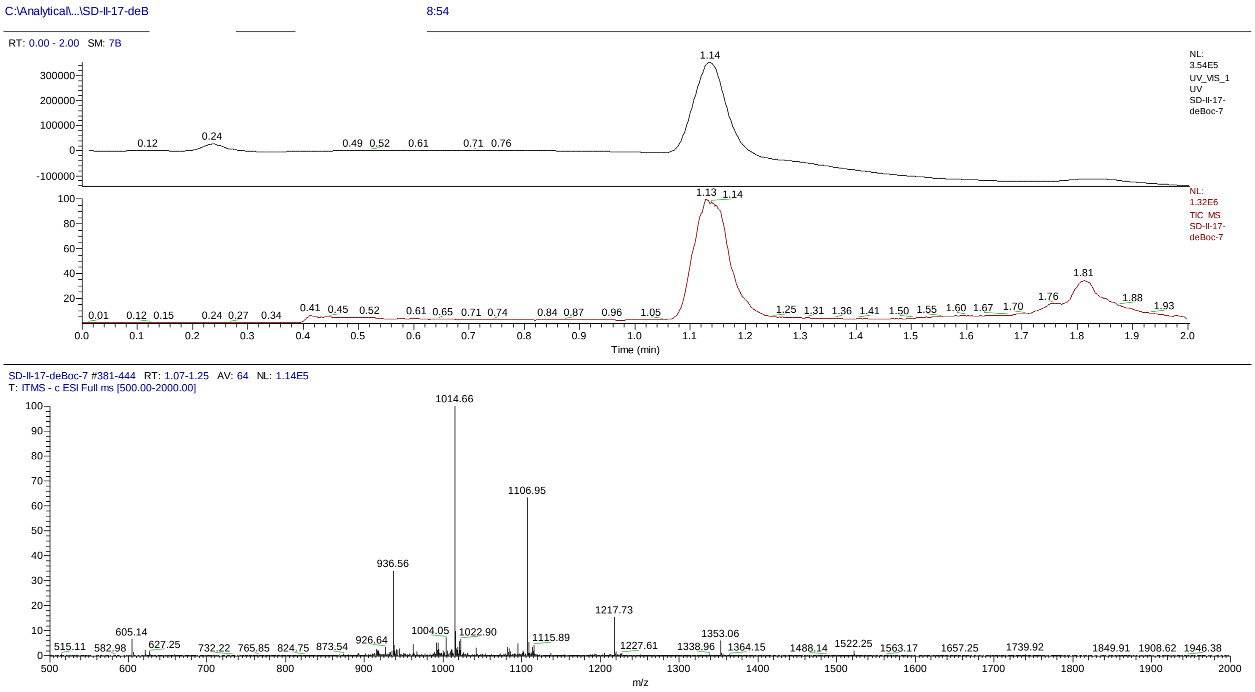
C:\Analytical\...\SD-II-17-deB
8:54
RT: 0.00 - 2.00 SM: 7B
NL: 3.54E5 UV_VIS_1 UV SD-II-17-deBoc-7
NL: 1.32E6 TIC MS SD-II-17-deBoc-7
Time (min)
SD-II-17-deBoc-7 #381-444 RT: 1.07-1.25 AV: 64 NL: 1.14E5
T: ITMS - c ESI Full ms [500.00-2000.00]
1014.66
1106.95
936.56
1217.73
m/z

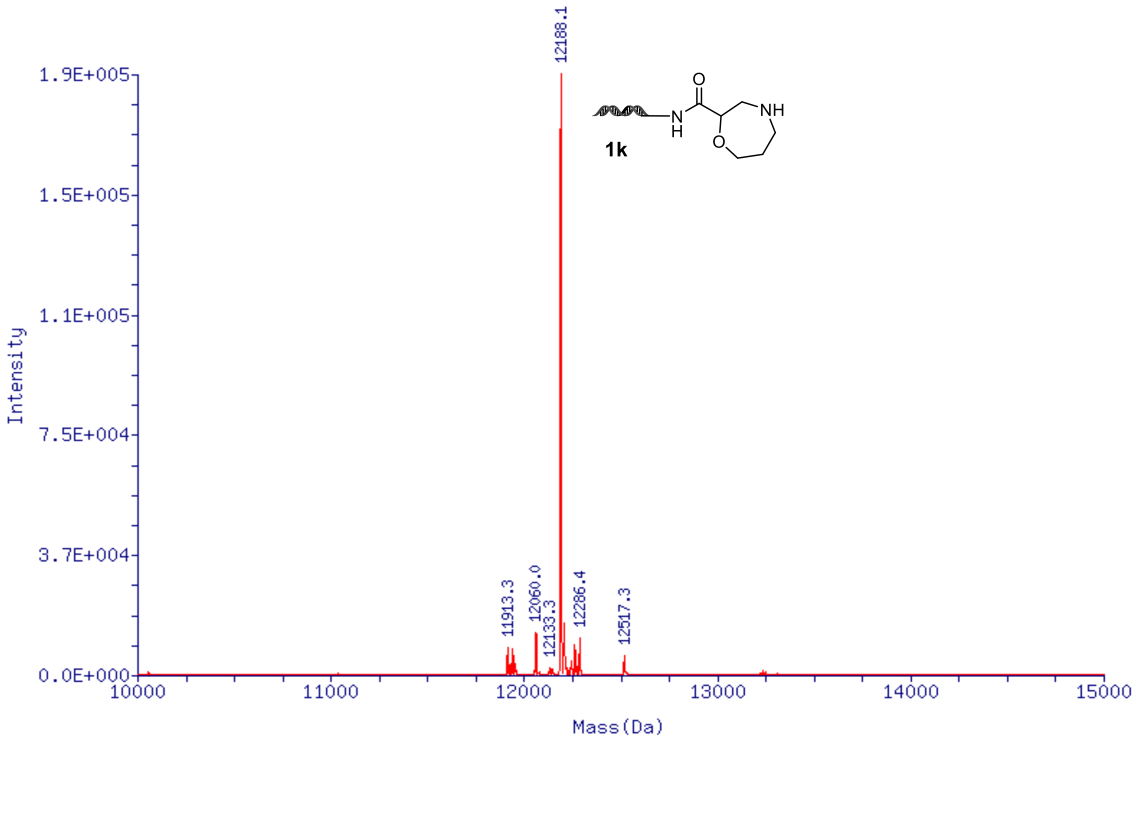
12188.1
11913.3
12060.0
12133.3
12286.4
12517.3
Intensity
Mass(Da)
1k

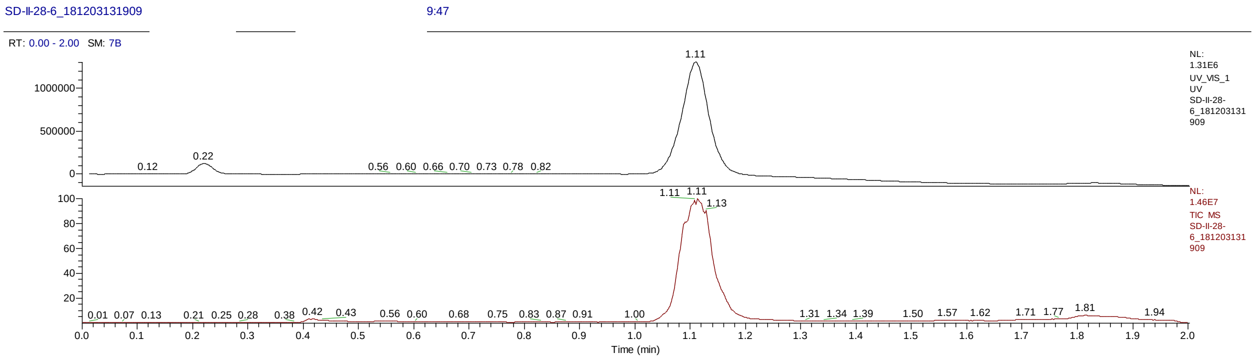

SD-II-28-6_181203131909
9:47
RT: 0.00 - 2.00 SM: 7B
NL: 1.31E6 UV_VIS_1 UV SD-II-28-6_181203131909
NL: 1.46E7 TIC MS SD-II-28-6_181203131909
Time (min)

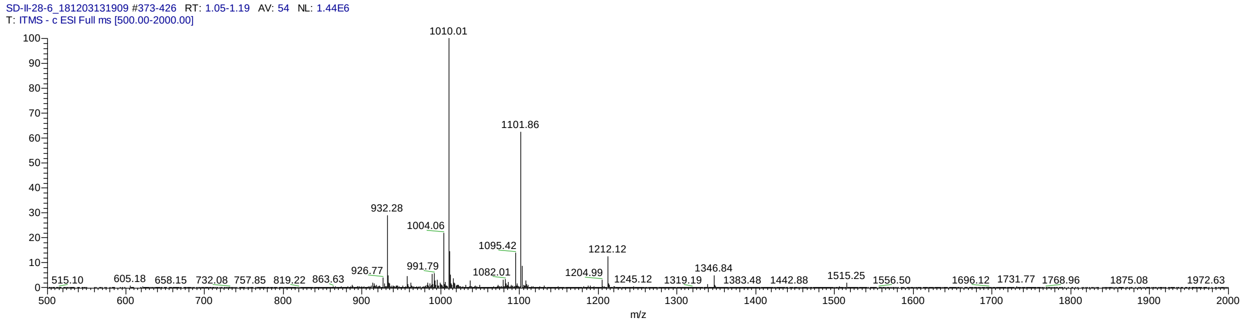

SD-II-28-6_181203131909 #373-426 RT: 1.05-1.19 AV: 54 NL: 1.44E6
T: ITMS - c ESI Full ms [500.00-2000.00]
1010.01
1101.86
932.28
1004.06
1095.42
1212.12
m/z

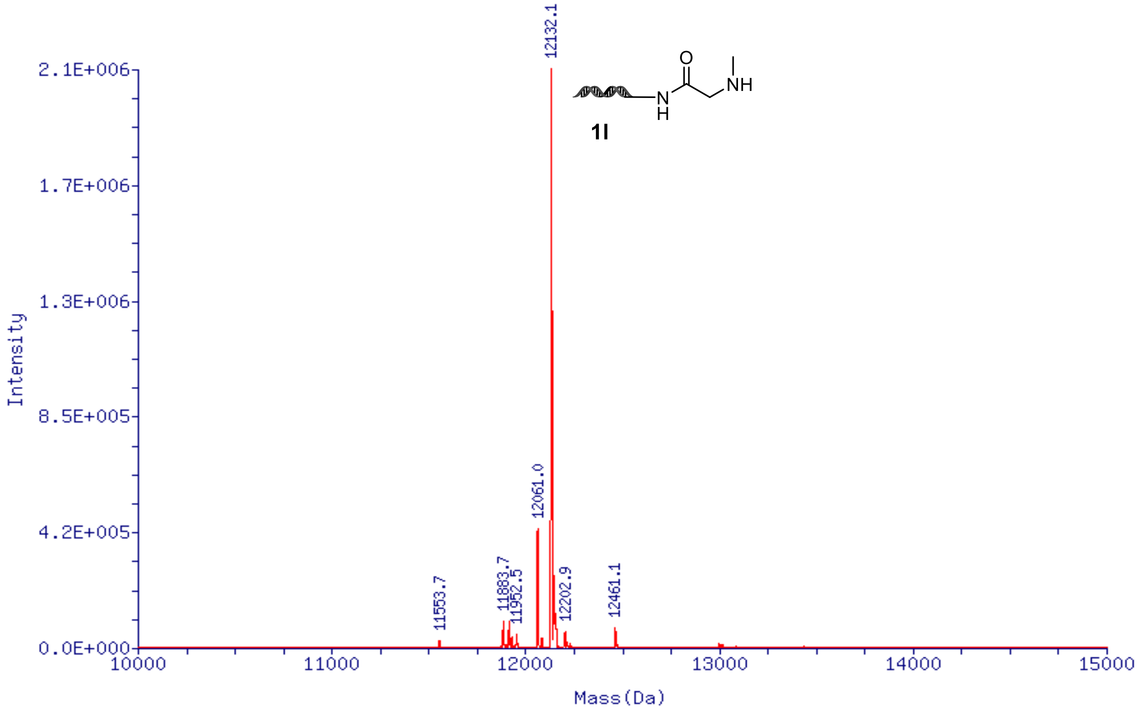

12132.1
12061.0
11553.7
11883.7
11952.5
12202.9
12461.1
Intensity
Mass(Da)
1l

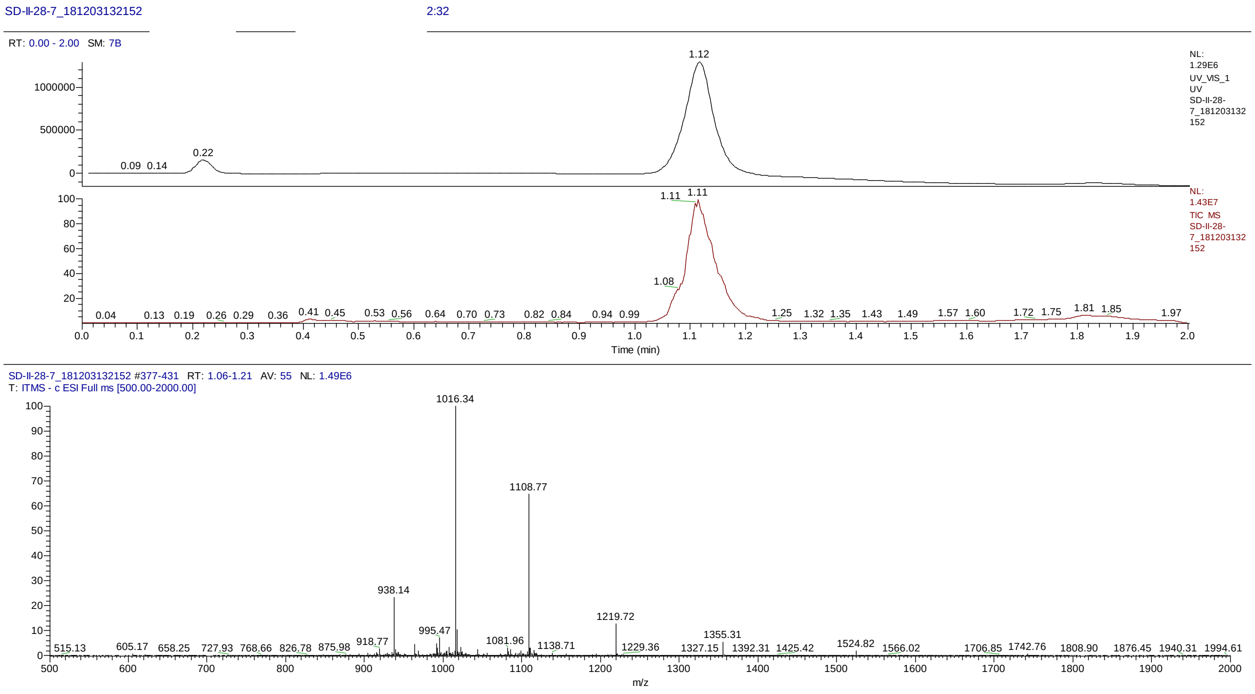
SD-II-28-7_181203132152
2:32
RT: 0.00 - 2.00 SM: 7B
NL: 1.29E6 UV_VIS_1 UV SD-II-28-7_181203132152
NL: 1.43E7 TIC MS SD-II-28-7_181203132152
Time (min)
SD-II-28-7_181203132152 #377-431 RT: 1.06-1.21 AV: 55 NL: 1.49E6
T: ITMS - c ESI Full ms [500.00-2000.00]
1016.34
1108.77
938.14
1219.72
995.47
1355.31
1081.96
1524.82
m/z

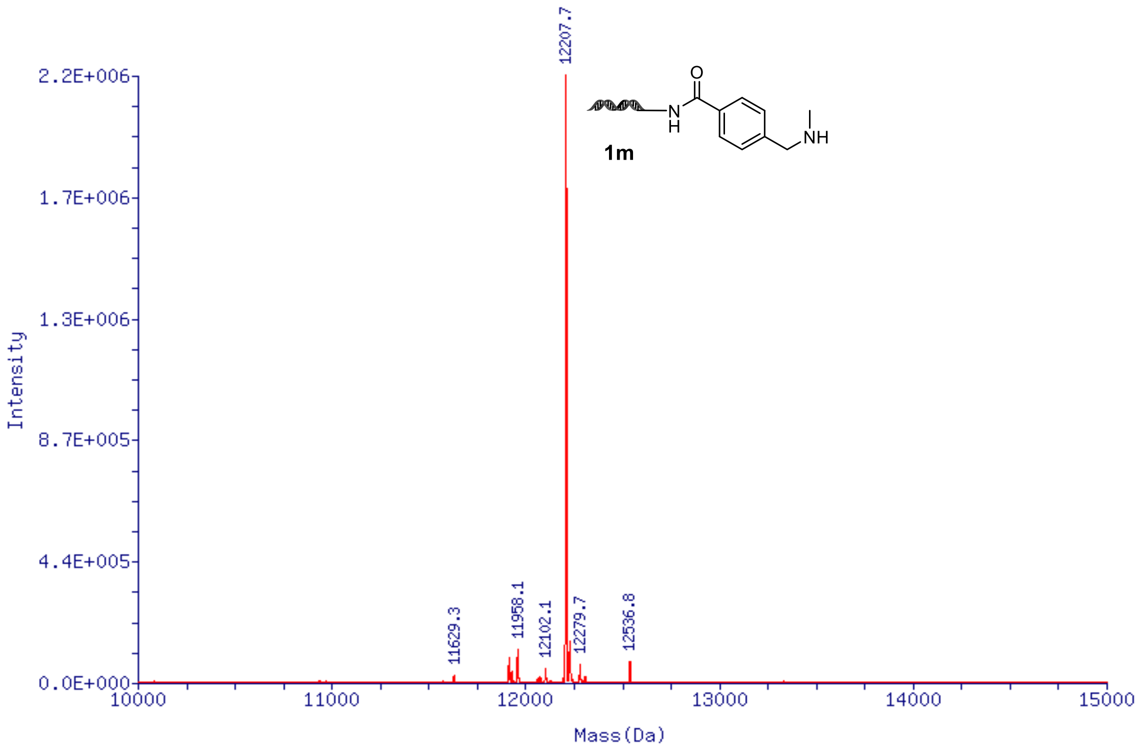
12207.7
1m
Intensity
Mass(Da)
11629.3
11958.1
12102.1
12279.7
12536.8

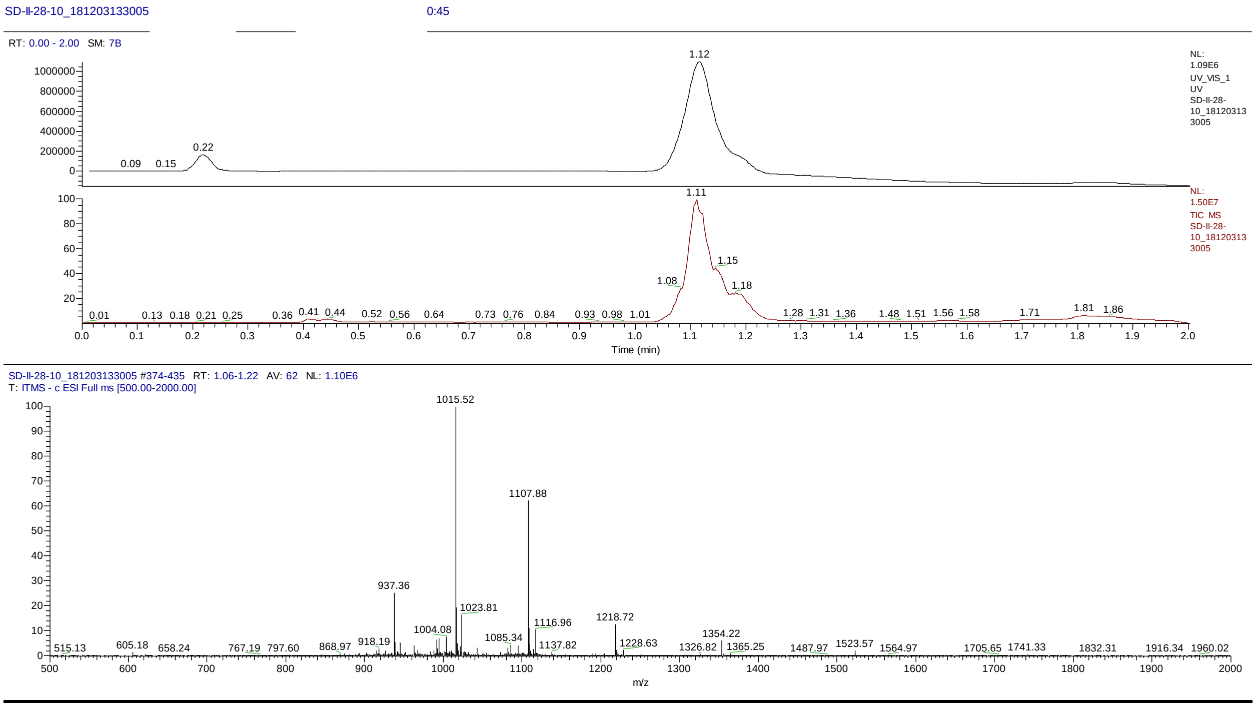
SD-II-28-10_181203133005
0:45
RT: 0.00 - 2.00 SM: 7B
NL: 1.09E6 UV_VIS_1 UV SD-II-28-10_181203133005
NL: 1.50E7 TIC MS SD-II-28-10_181203133005
Time (min)
SD-II-28-10_181203133005 #374-435 RT: 1.06-1.22 AV: 62 NL: 1.10E6
T: ITMS - c ESI Full ms [500.00-2000.00]
1015.52
1107.88
937.36
1023.81
1218.72
m/z

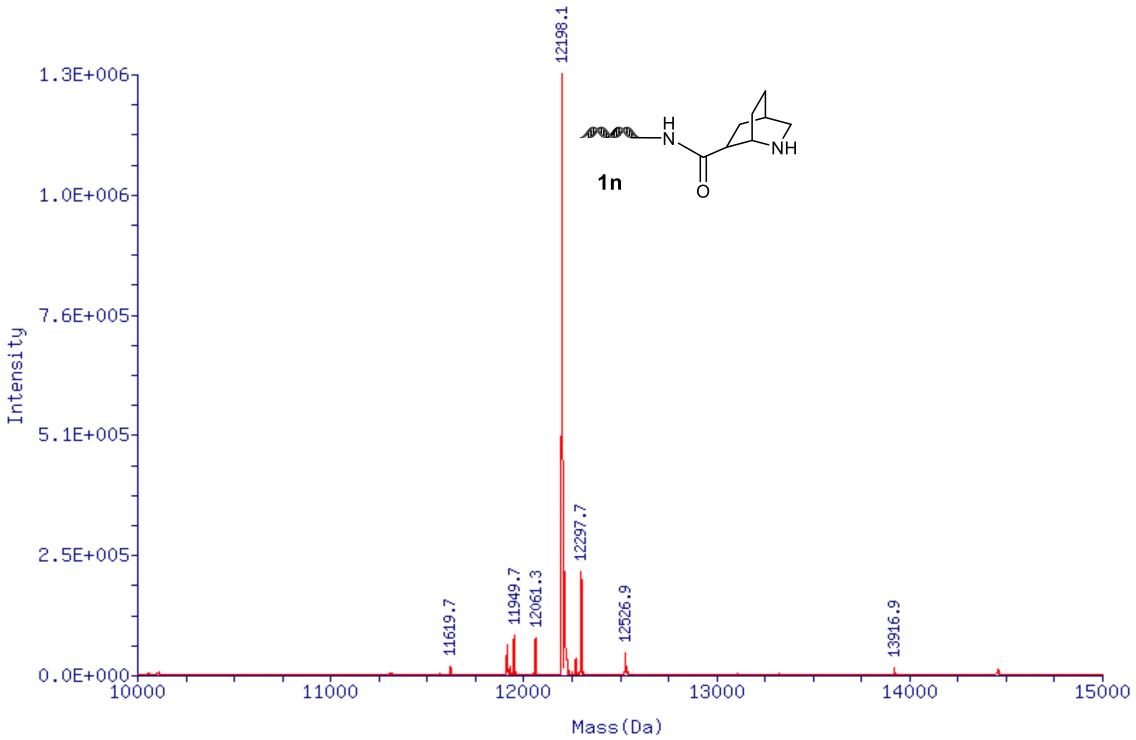
12198.1
12297.7
12061.3
11949.7
11619.7
12526.9
13916.9
Intensity
Mass(Da)
1n

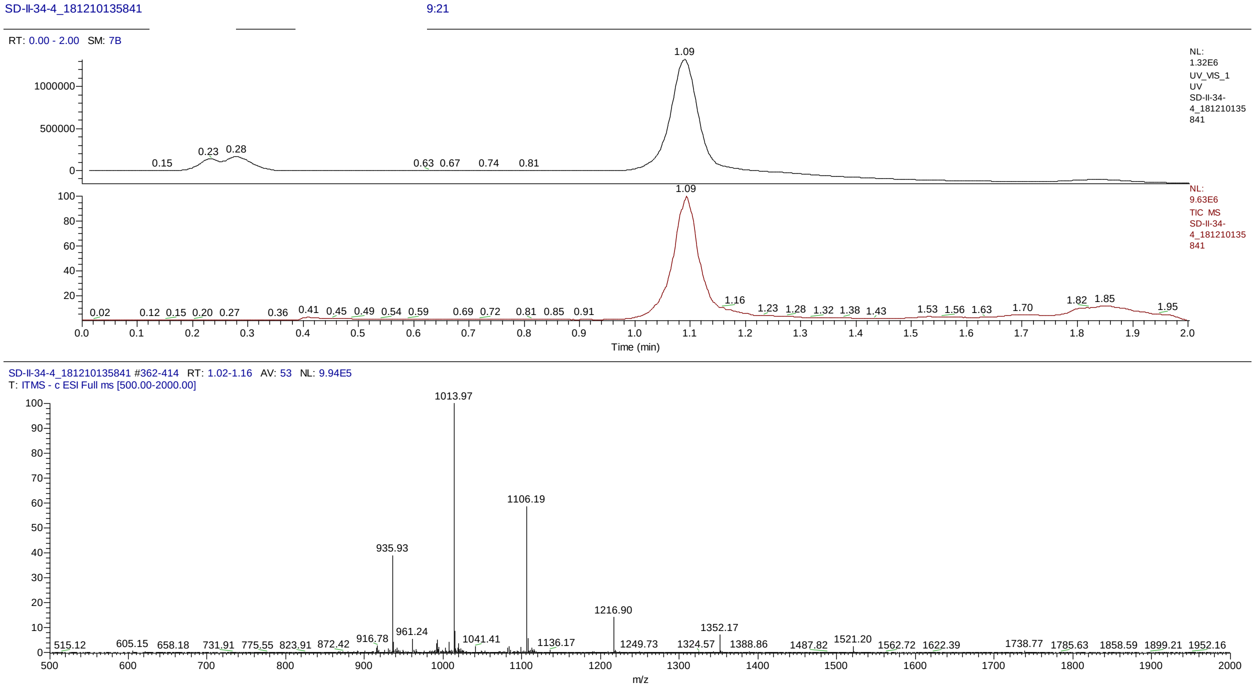
SD-II-34-4_181210135841
9:21
RT: 0.00 - 2.00 SM: 7B
NL: 1.32E6 UV_VIS_1 UV SD-II-34-4_181210135841
NL: 9.63E6 TIC MS SD-II-34-4_181210135841
Time (min)
SD-II-34-4_181210135841 #362-414 RT: 1.02-1.16 AV: 53 NL: 9.94E5
T: ITMS - c ESI Full ms [500.00-2000.00]
1013.97
1106.19
935.93
1216.90
1352.17
m/z

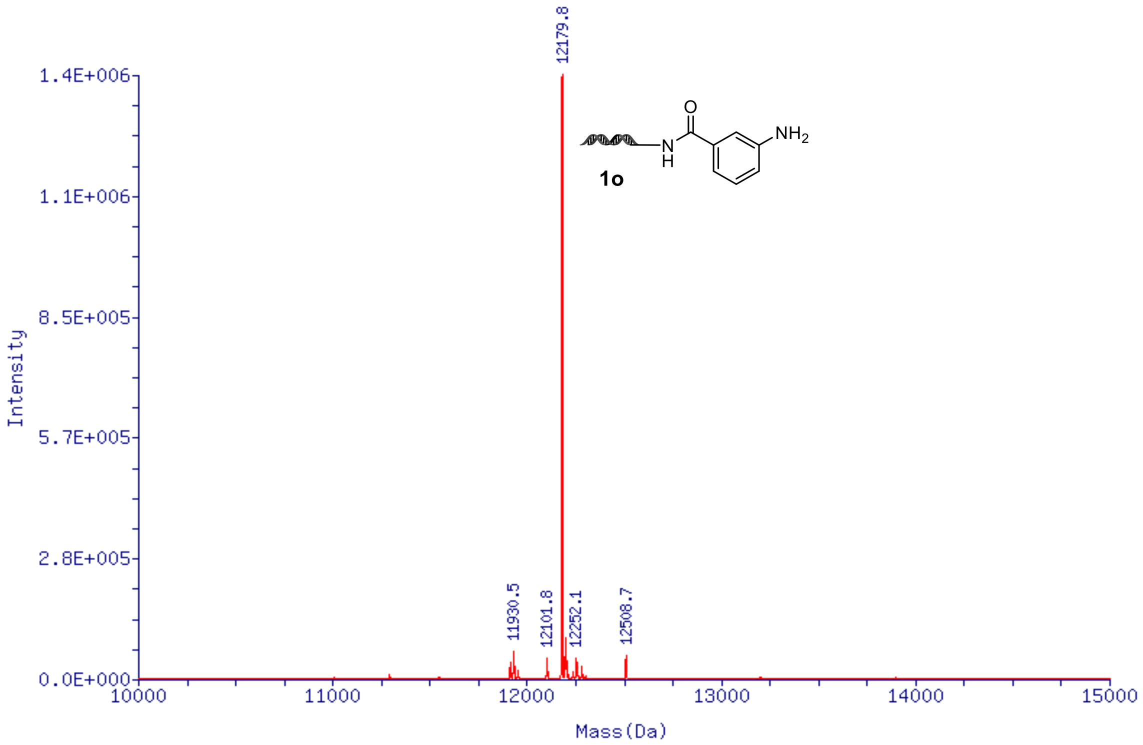
12179.8
11930.5
12101.8
12252.1
12508.7
Intensity
Mass(Da)
1o

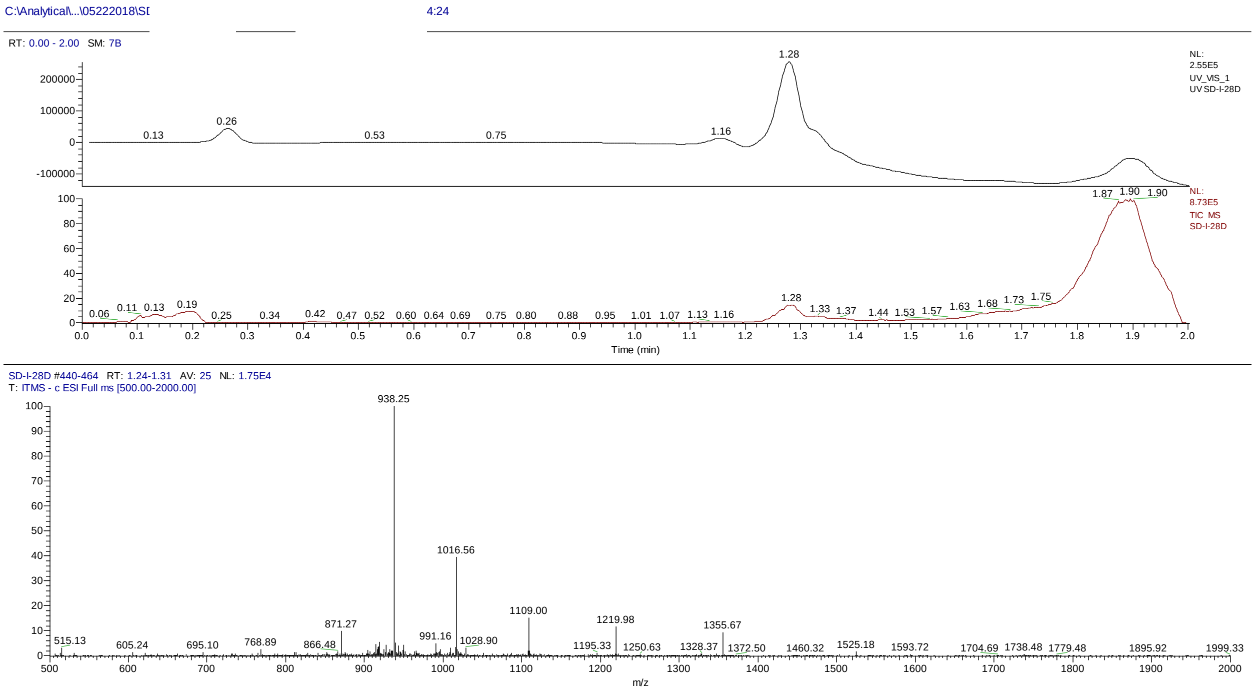

C:\Analytical\...\05222018\SI
4:24
RT: 0.00 - 2.00 SM: 7B
NL: 2.55E5 UV_VIS_1 UV SD-I-28D
NL: 8.73E5 TIC MS SD-I-28D
Time (min)
SD-I-28D #440-464 RT: 1.24-1.31 AV: 25 NL: 1.75E4
T: ITMS - c ESI Full ms [500.00-2000.00]
938.25
1016.56
1109.00
1219.98
1355.67
871.27
m/z

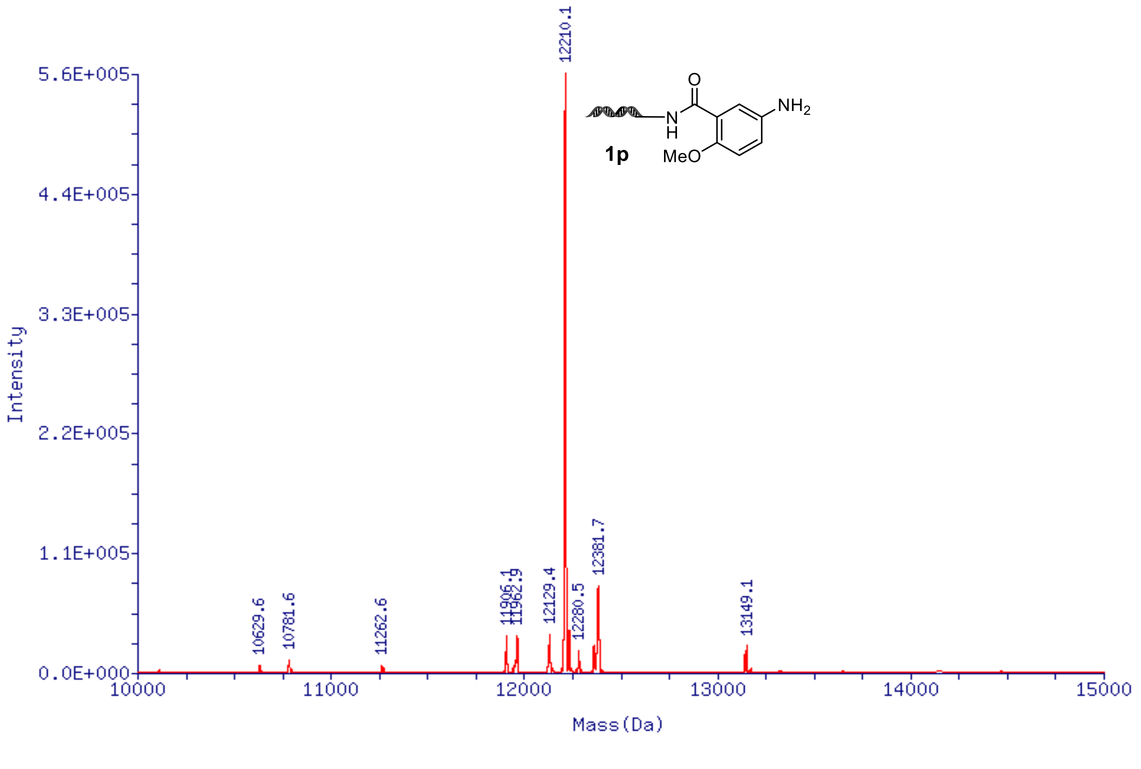

12210.1
1p
MeO
NH2
Intensity
Mass(Da)
10629.6
10781.6
11262.6
12129.4
12280.5
12381.7
13149.1

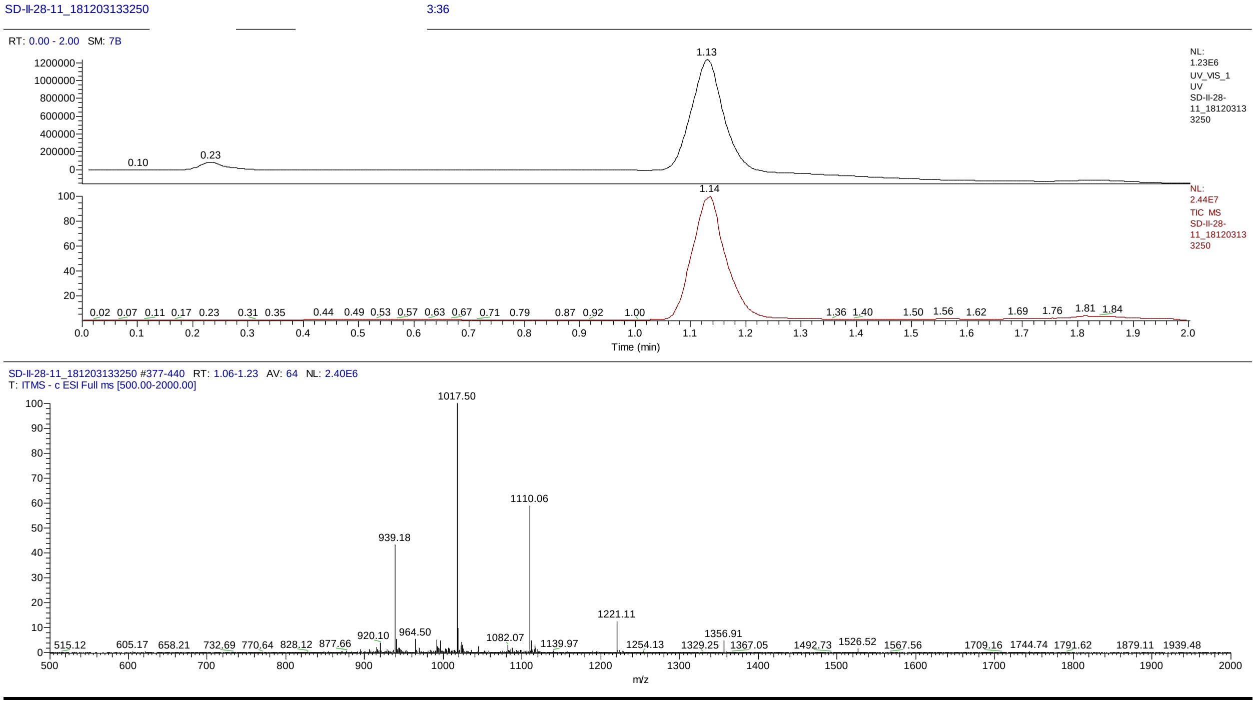
SD-II-28-11_181203133250
3:36
RT: 0.00 - 2.00 SM: 7B
1.13
NL: 1.23E6 UV_VIS_1 UV SD-II-28-11_181203133250
1.14
NL: 2.44E7 TIC MS SD-II-28-11_181203133250
Time (min)
SD-II-28-11_181203133250 #377-440 RT: 1.06-1.23 AV: 64 NL: 2.40E6
T: ITMS - c ESI Full ms [500.00-2000.00]
1017.50
1110.06
939.18
1221.11
m/z

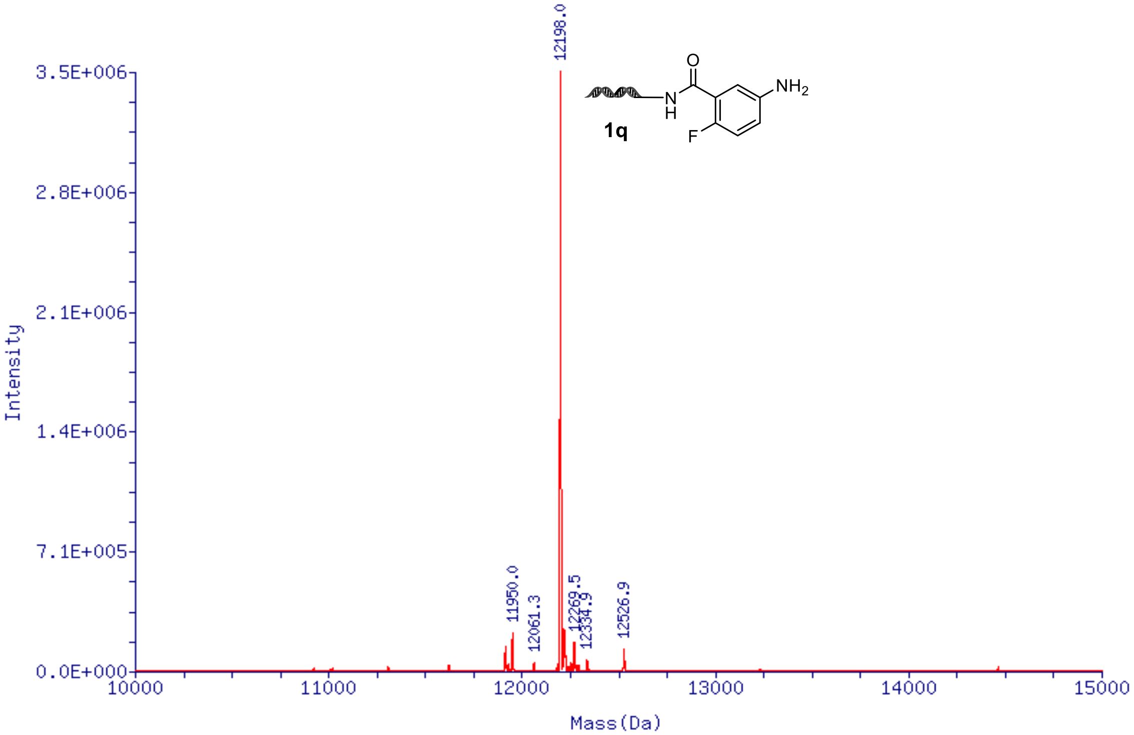
12198.0
1q
11950.0
12061.3
12269.5
12334.9
12526.9
Intensity
Mass(Da)

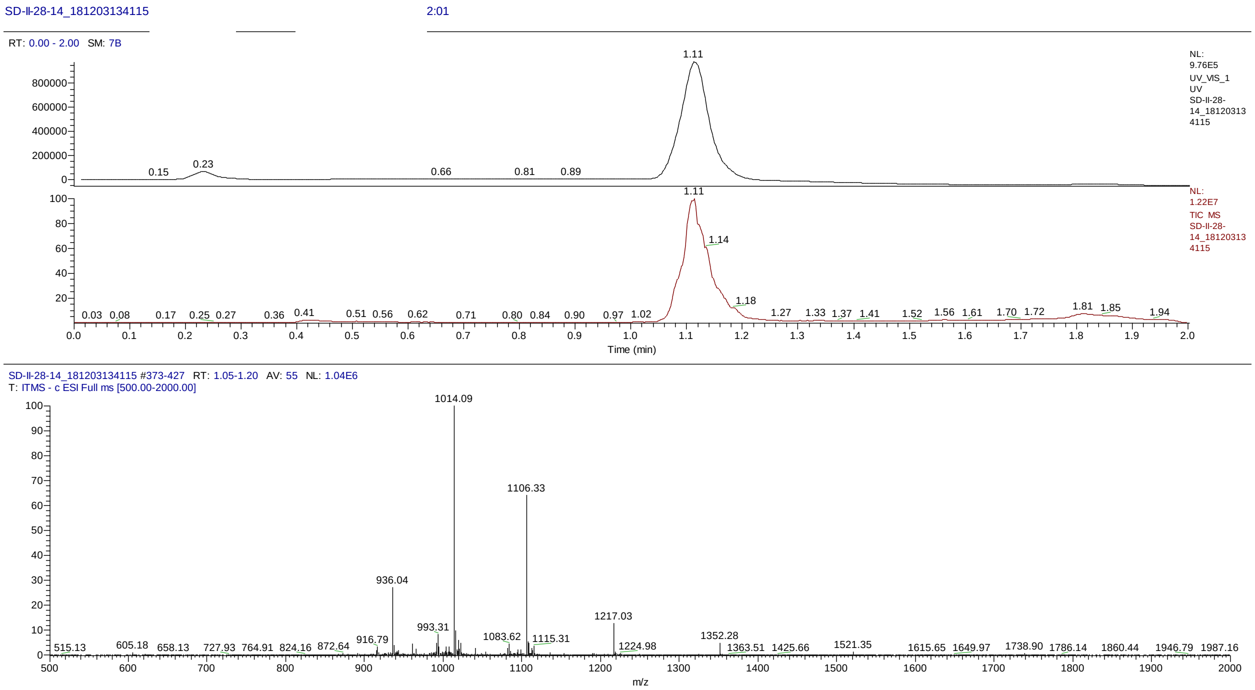
SD-II-28-14_181203134115
2:01
RT: 0.00 - 2.00 SM: 7B
NL: 9.76E5 UV_VIS_1 UV SD-II-28-14_181203134115
NL: 1.22E7 TIC MS SD-II-28-14_181203134115
Time (min)
SD-II-28-14_181203134115 #373-427 RT: 1.05-1.20 AV: 55 NL: 1.04E6
T: ITMS - c ESI Full ms [500.00-2000.00]
1014.09
1106.33
936.04
1217.03
m/z

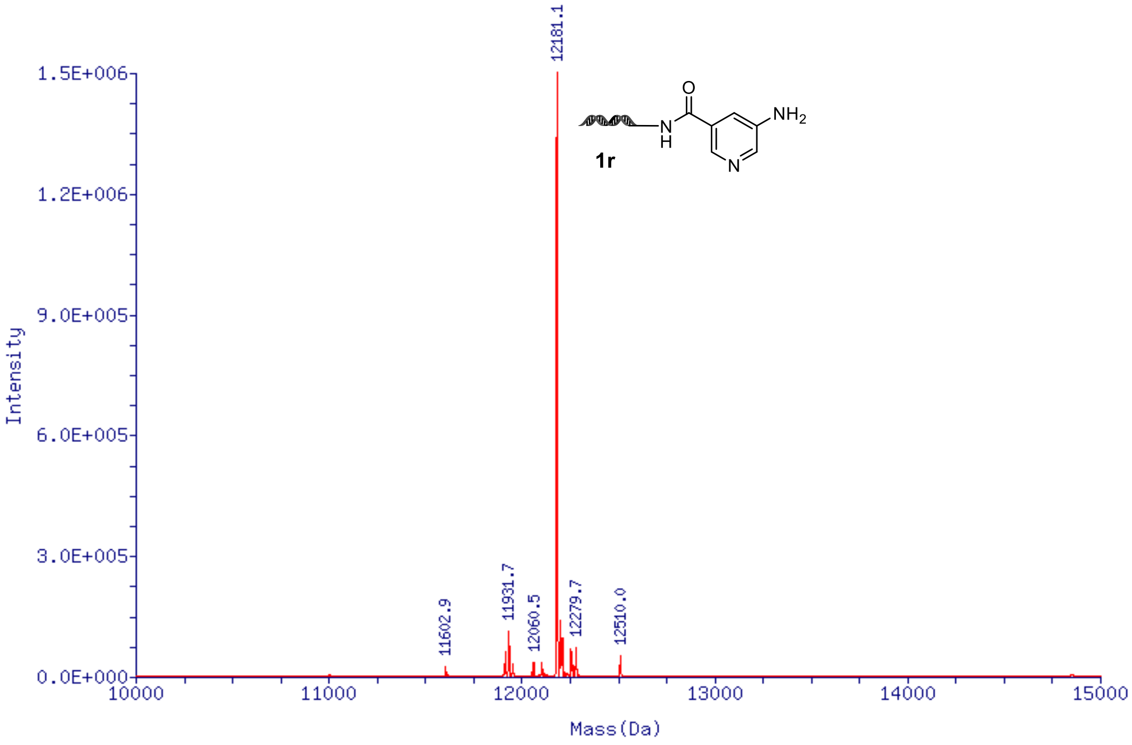
12181.1
11602.9
11931.7
12060.5
12279.7
12510.0
Intensity
Mass(Da)
NH2
1r

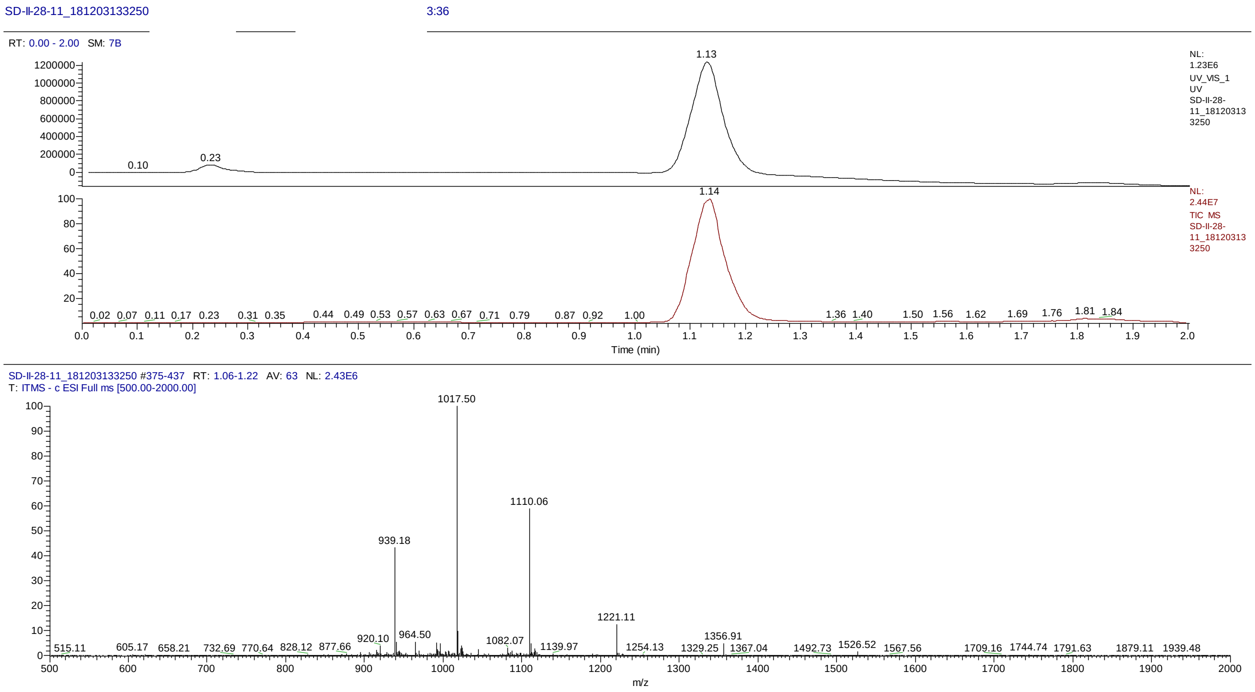
SD-II-28-11_181203133250
3:36
RT: 0.00 - 2.00 SM: 7B
NL: 1.23E6 UV_VIS_1 UV SD-II-28-11_181203133250
NL: 2.44E7 TIC MS SD-II-28-11_181203133250
Time (min)
SD-II-28-11_181203133250 #375-437 RT: 1.06-1.22 AV: 63 NL: 2.43E6
T: ITMS - c ESI Full ms [500.00-2000.00]
1017.50
1110.06
939.18
1221.11
m/z

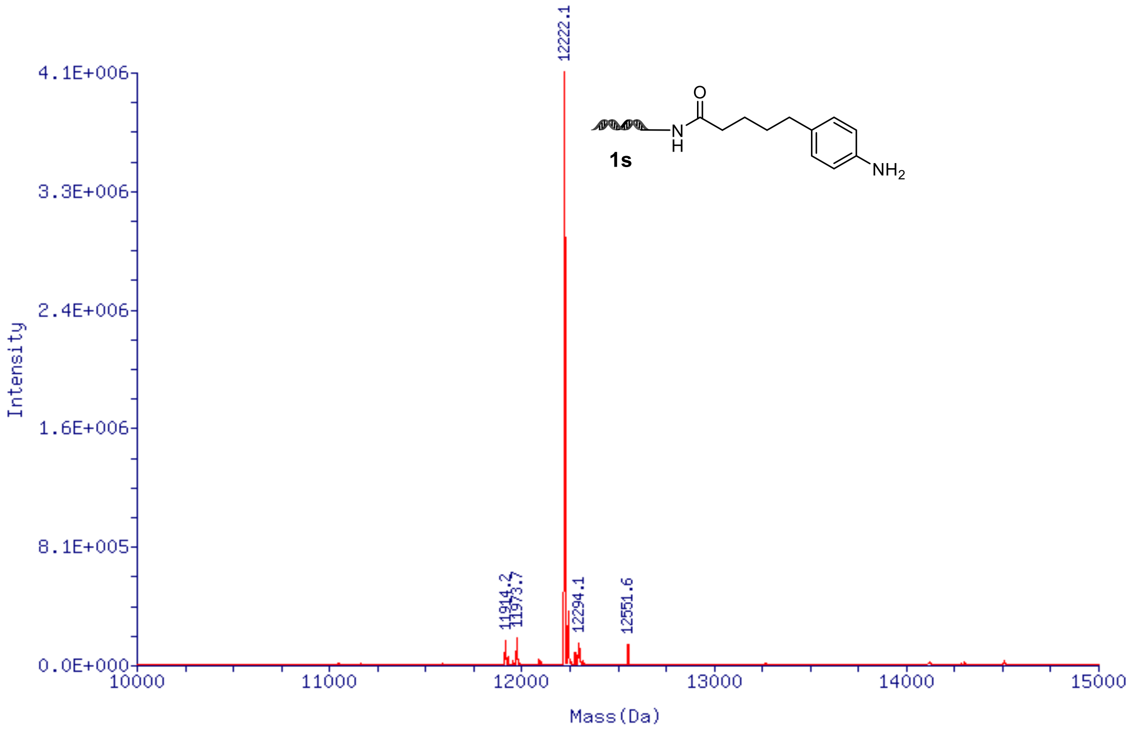
12222.1
1s
Intensity
Mass(Da)
11914.2
11973.7
12294.1
12551.6

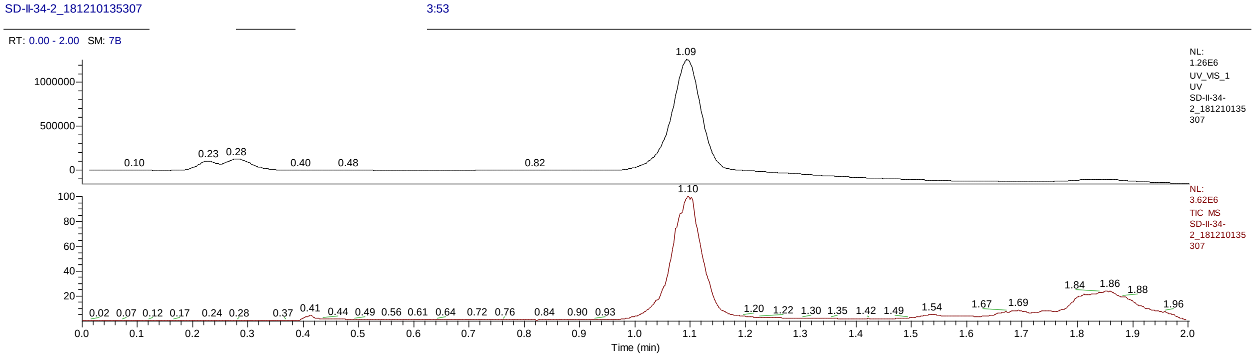
SD-II-34-2_181210135307
3:53
RT: 0.00 - 2.00 SM: 7B
NL: 1.26E6 UV_VIS_1 UV SD-II-34-2_181210135307
NL: 3.62E6 TIC MS SD-II-34-2_181210135307
Time (min)

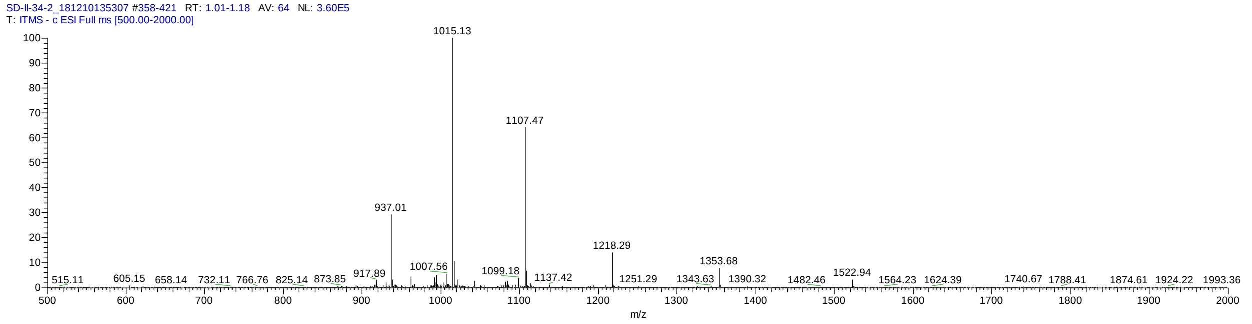
SD-II-34-2_181210135307 #358-421 RT: 1.01-1.18 AV: 64 NL: 3.60E5
T: ITMS - c ESI Full ms [500.00-2000.00]
1015.13
1107.47
937.01
1218.29
1353.68
m/z

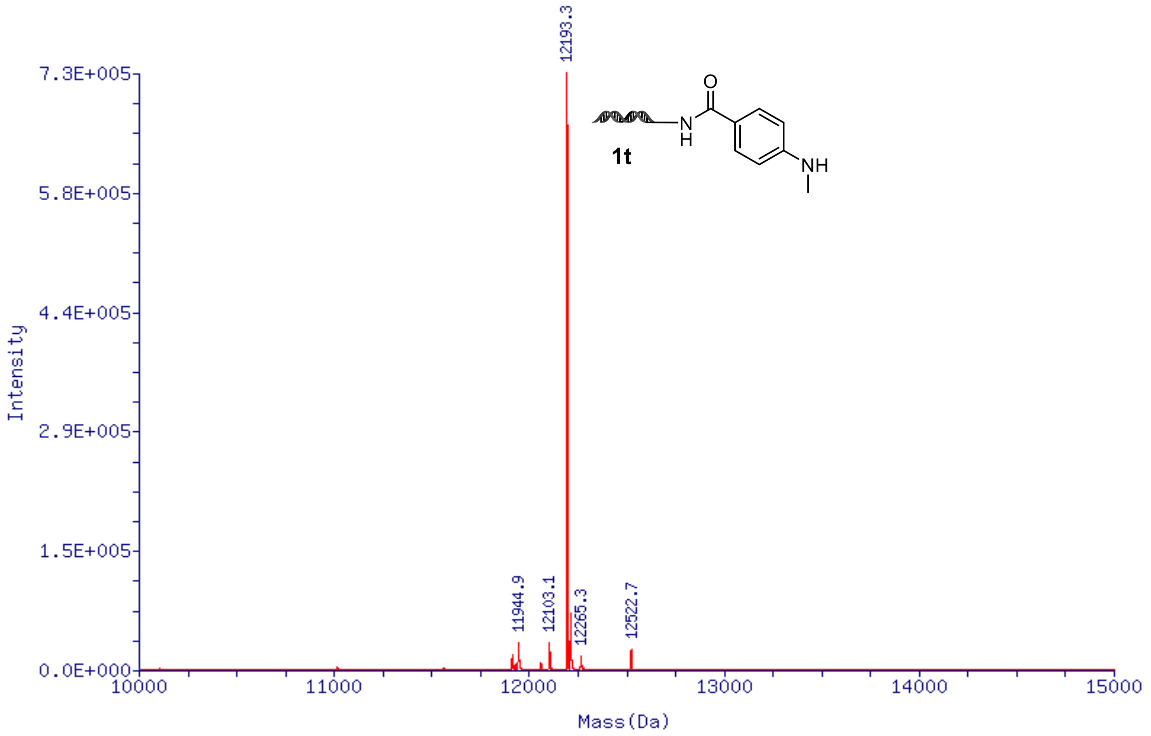
12193.3
11944.9
12103.1
12265.3
12522.7
1t
Intensity
Mass(Da)

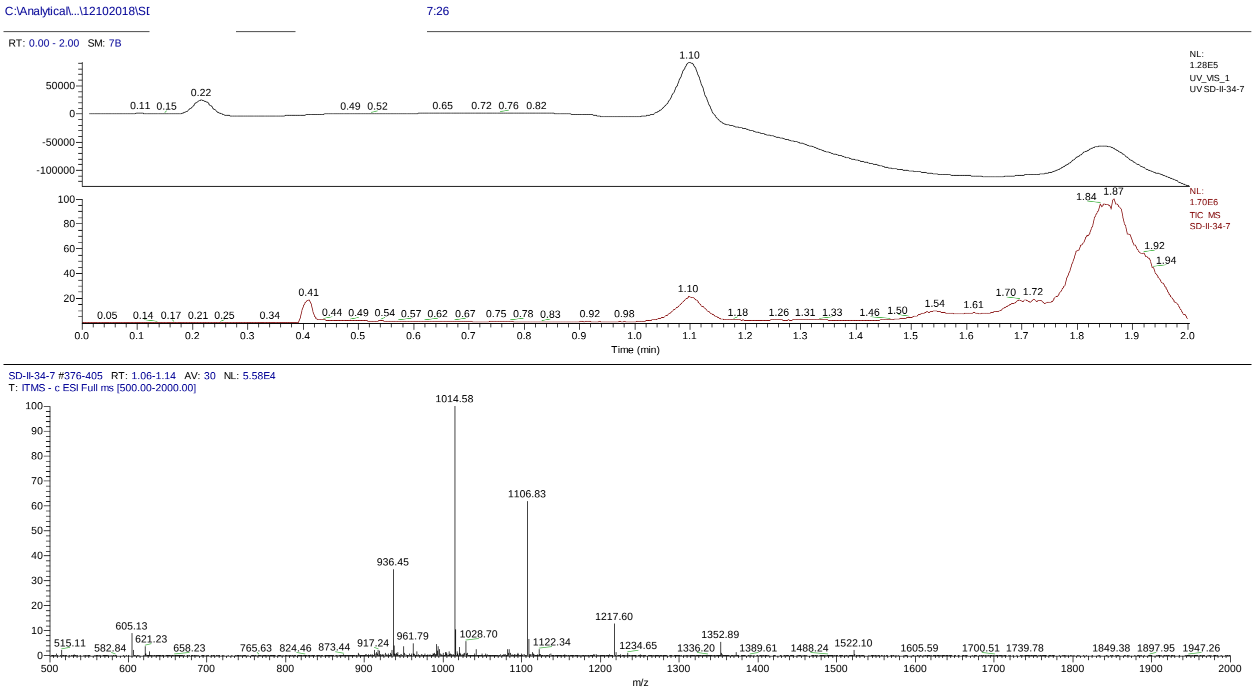
C:\Analytical\...\12102018\SI
7:26
RT: 0.00 - 2.00 SM: 7B
NL: 1.28E5 UV_VIS_1 UV SD-II-34-7
NL: 1.70E6 TIC MS SD-II-34-7
Time (min)
SD-II-34-7 #376-405 RT: 1.06-1.14 AV: 30 NL: 5.58E4
T: ITMS - c ESI Full ms [500.00-2000.00]
1014.58
1106.83
936.45
1217.60
m/z

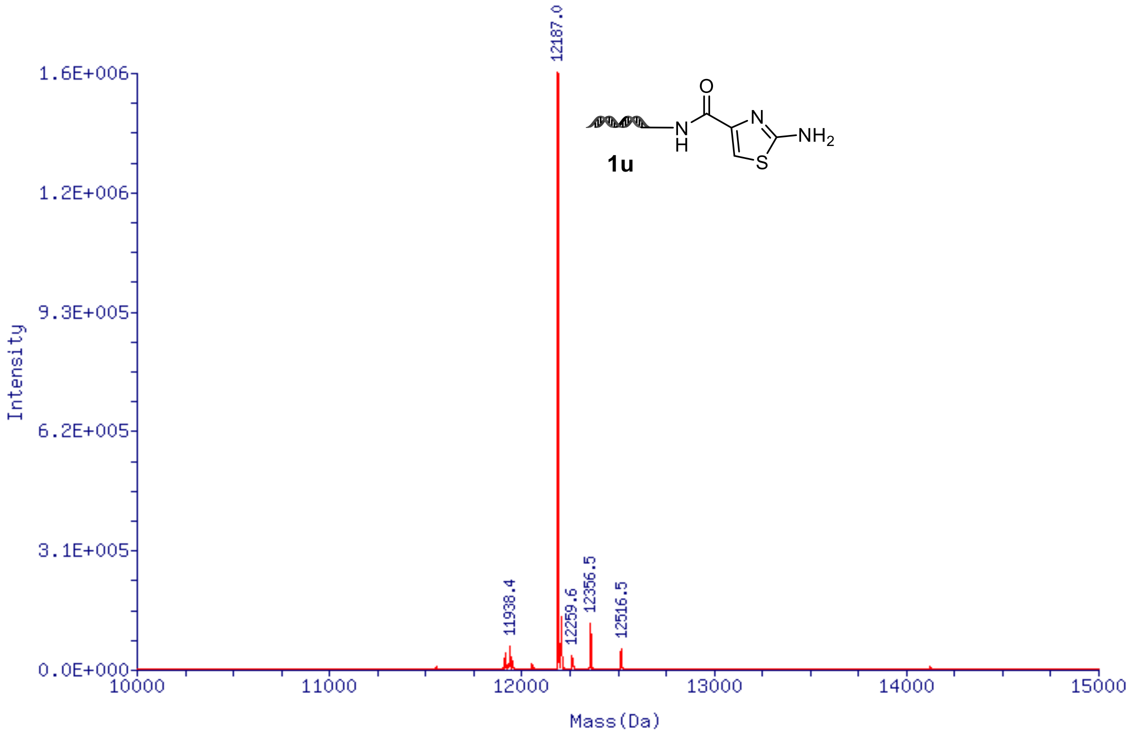
12187.0
11938.4
12259.6
12356.5
12516.5
Intensity
Mass(Da)
1u

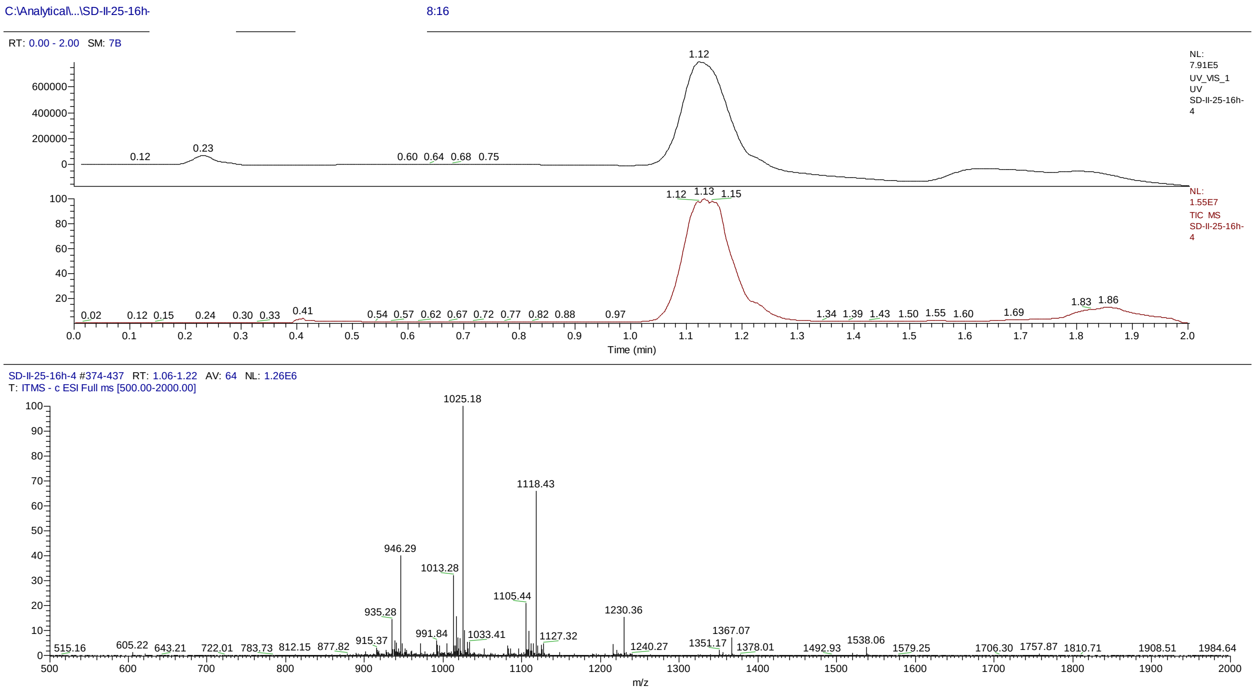
C:\Analytical\...\SD-II-25-16h-
8:16
RT: 0.00 - 2.00 SM: 7B
1.12
0.12
0.23
0.60 0.64 0.68 0.75
NL: 7.91E5 UV_VIS_1 UV SD-II-25-16h-4
1.12 1.13 1.15
0.02 0.12 0.15 0.24 0.30 0.33 0.41 0.54 0.57 0.62 0.67 0.72 0.77 0.82 0.88 0.97
1.34 1.39 1.43 1.50 1.55 1.60 1.69 1.83 1.86
NL: 1.55E7 TIC MS SD-II-25-16h-4
Time (min)
SD-II-25-16h-4 #374-437 RT: 1.06-1.22 AV: 64 NL: 1.26E6
T: ITMS - c ESI Full ms [500.00-2000.00]
1025.18
1118.43
946.29
1013.28
1105.44
935.28
1230.36
1367.07
991.84
1033.41
1127.32
915.37
1538.06
515.16 605.22 643.21 722.01 783.73 812.15 877.82 1240.27 1351.17 1378.01 1492.93 1579.25 1706.30 1757.87 1810.71 1908.51 1984.64
m/z

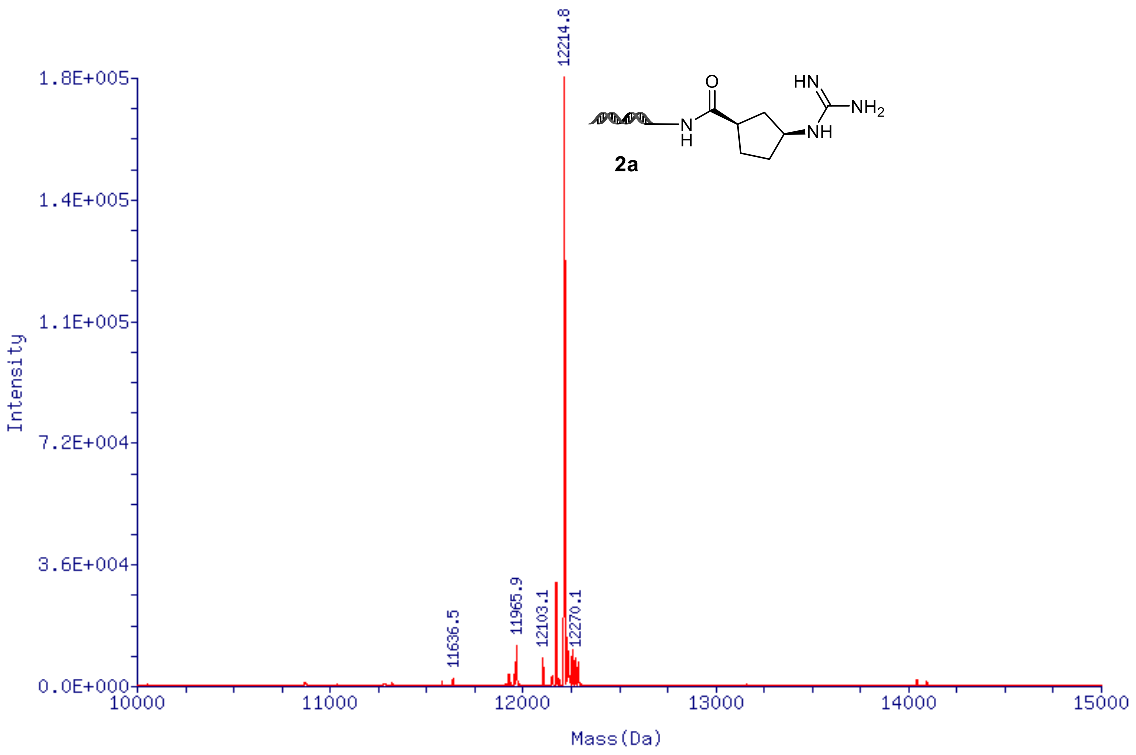
12214.8
2a
HN
O
NH2
NH
N
H
Intensity
11636.5
11965.9
12103.1
12270.1
Mass(Da)

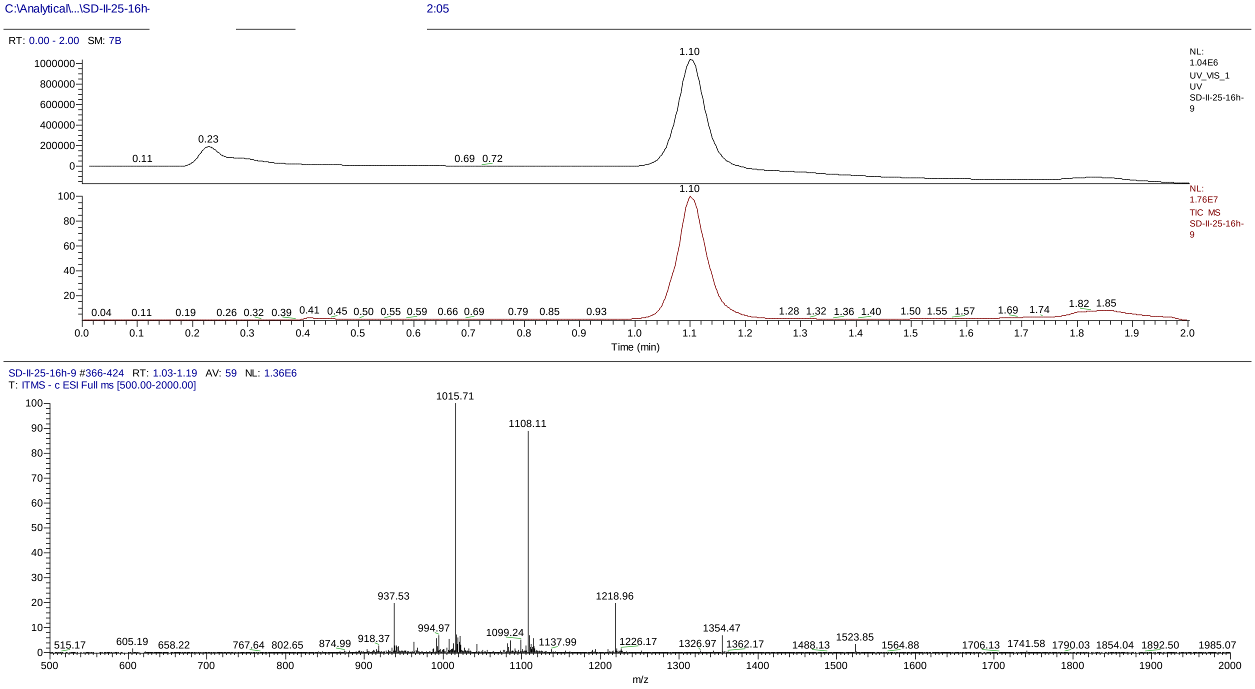
C:\Analytical\...\SD-II-25-16h-
2:05
RT: 0.00 - 2.00 SM: 7B
NL: 1.04E6 UV_VIS_1 UV SD-II-25-16h-9
NL: 1.76E7 TIC MS SD-II-25-16h-9
Time (min)
SD-II-25-16h-9 #366-424 RT: 1.03-1.19 AV: 59 NL: 1.36E6
T: ITMS - c ESI Full ms [500.00-2000.00]
1015.71
1108.11
1218.96
937.53
m/z

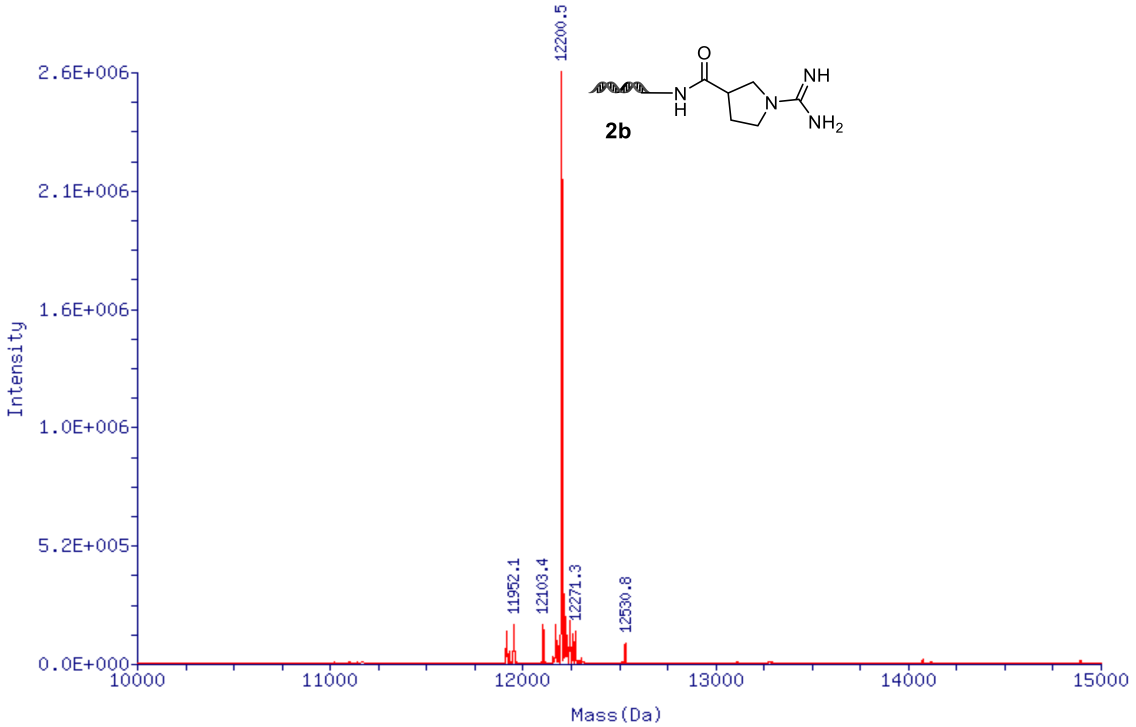
12200.5
11952.1
12103.4
12271.3
12530.8
Intensity
Mass(Da)
2b
O
N
H
N
NH
NH2

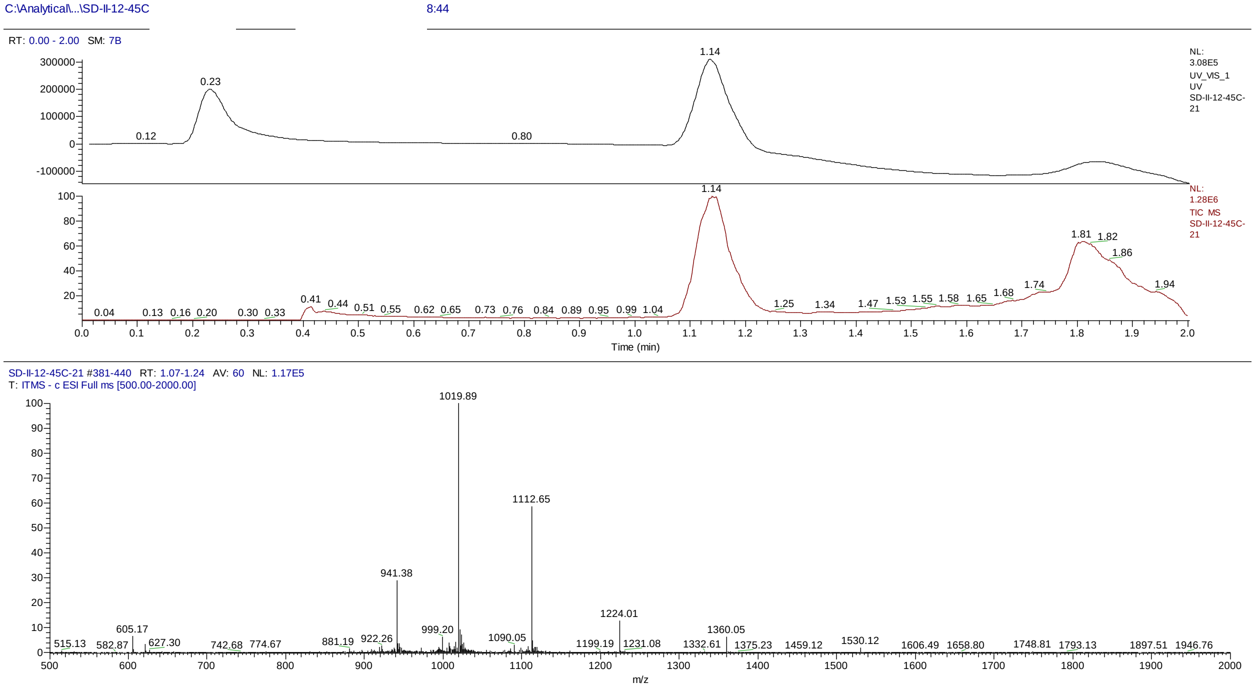
C:\Analytical\...\SD-II-12-45C
8:44
RT: 0.00 - 2.00 SM: 7B
NL: 3.08E5 UV_VIS_1 UV SD-II-12-45C-21
NL: 1.28E6 TIC MS SD-II-12-45C-21
Time (min)
SD-II-12-45C-21 #381-440 RT: 1.07-1.24 AV: 60 NL: 1.17E5
T: ITMS - c ESI Full ms [500.00-2000.00]
1019.89
1112.65
941.38
1224.01
m/z

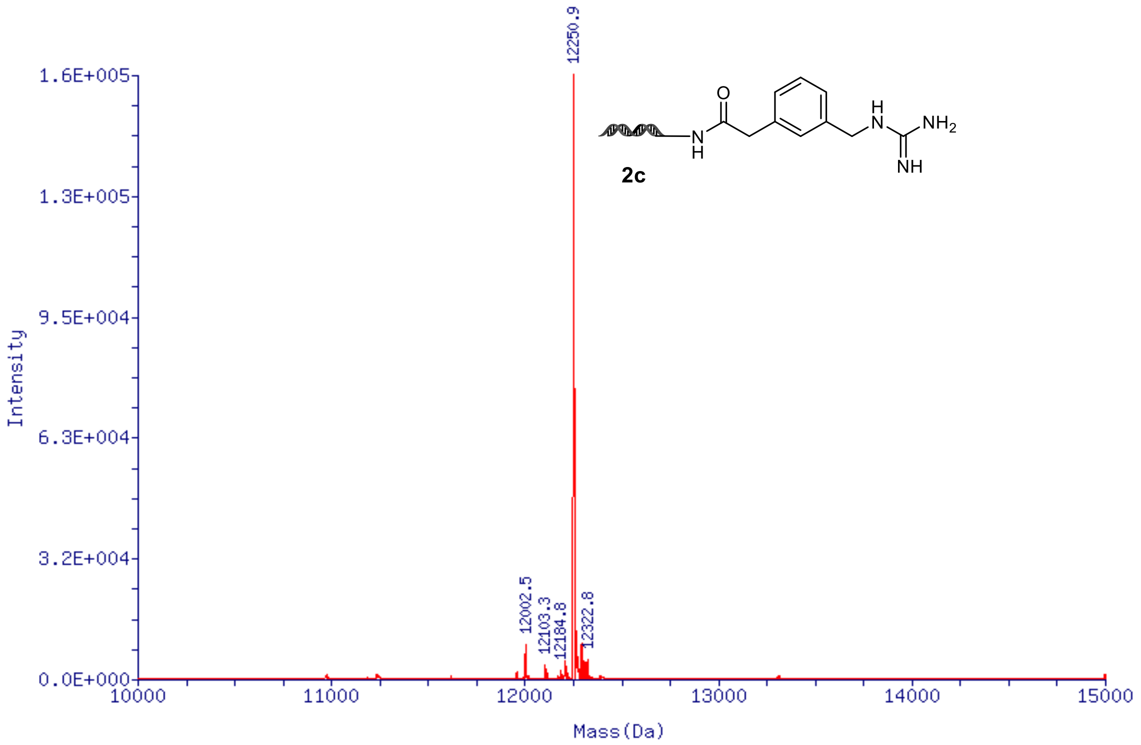
12250.9
12002.5
12103.3
12184.8
12322.8
Intensity
Mass(Da)
2c

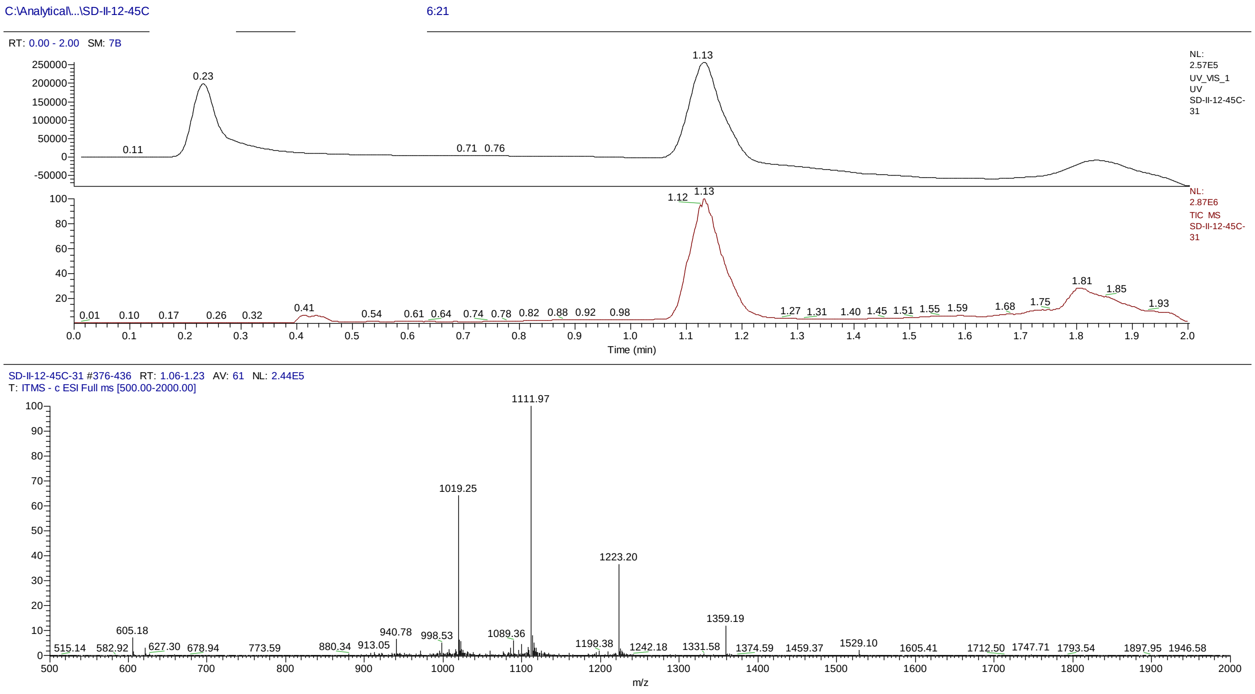
C:\Analytical\...\SD-II-12-45C
6:21
RT: 0.00 - 2.00 SM: 7B
NL: 2.57E5 UV_VIS_1 UV SD-II-12-45C-31
NL: 2.87E6 TIC MS SD-II-12-45C-31
Time (min)
SD-II-12-45C-31 #376-436 RT: 1.06-1.23 AV: 61 NL: 2.44E5
T: ITMS - c ESI Full ms [500.00-2000.00]
1111.97
1019.25
1223.20
1359.19
m/z

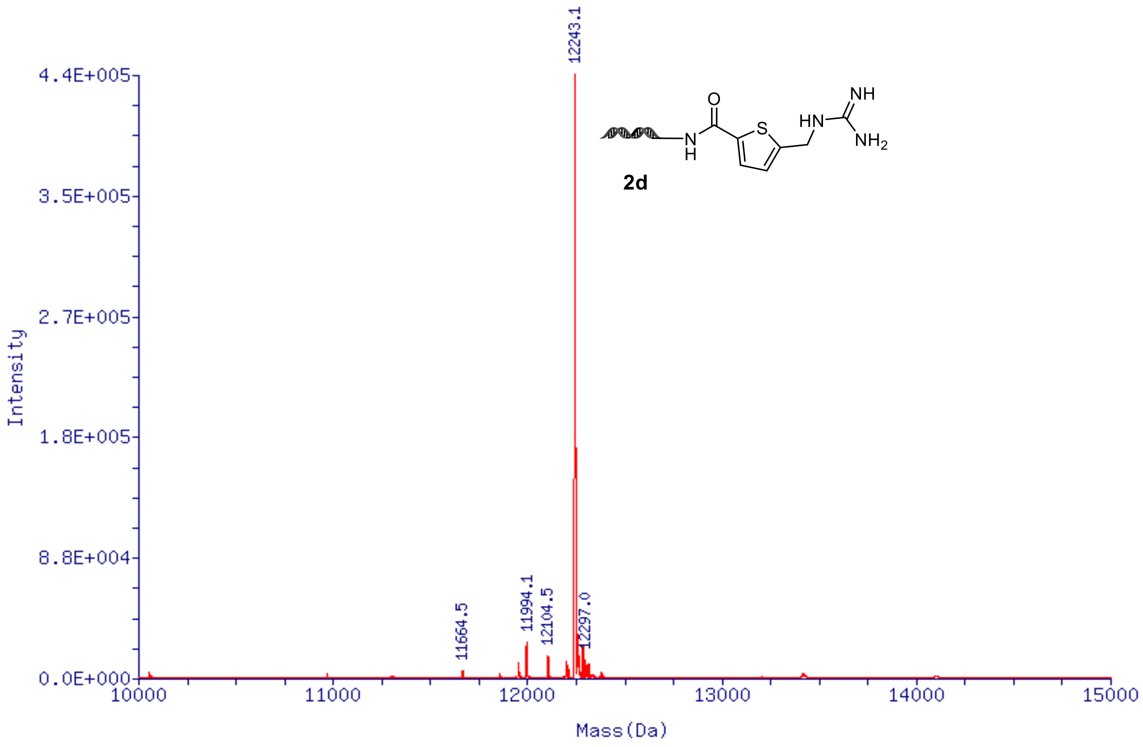
12243.1
11664.5
11994.1
12104.5
12297.0
Intensity
Mass(Da)
2d

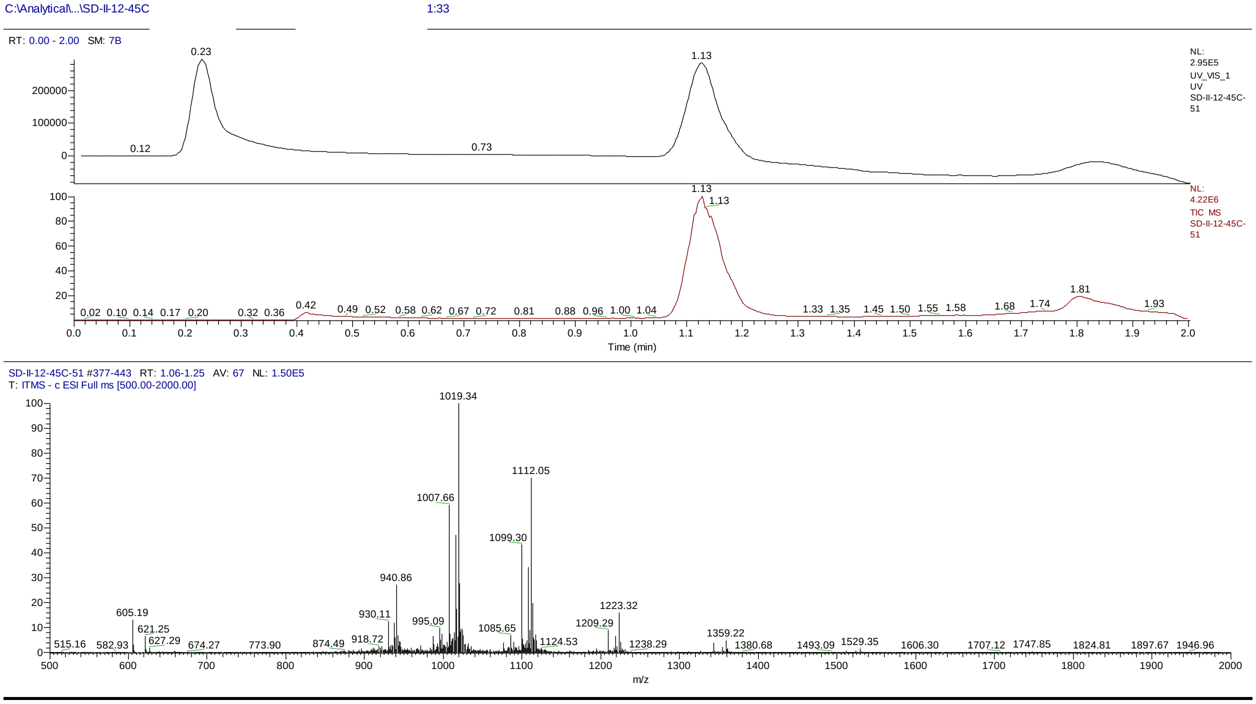

C:\Analytical\...\SD-II-12-45C
1:33
RT: 0.00 - 2.00 SM: 7B
NL: 2.95E5 UV_VIS_1 UV SD-II-12-45C-51
NL: 4.22E6 TIC MS SD-II-12-45C-51
Time (min)
SD-II-12-45C-51 #377-443 RT: 1.06-1.25 AV: 67 NL: 1.50E5
T: ITMS - c ESI Full ms [500.00-2000.00]
1019.34
1112.05
1007.66
1099.30
940.86
1223.32
605.19
m/z

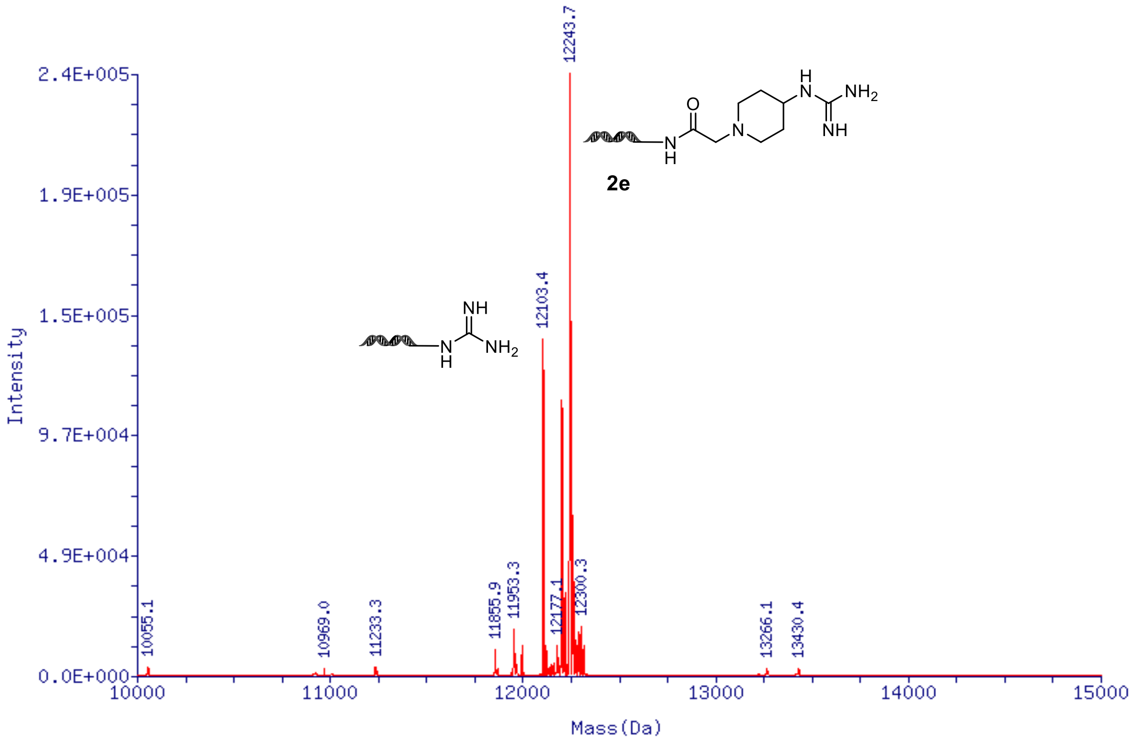

12243.7
12103.4
2e
Intensity
Mass(Da)

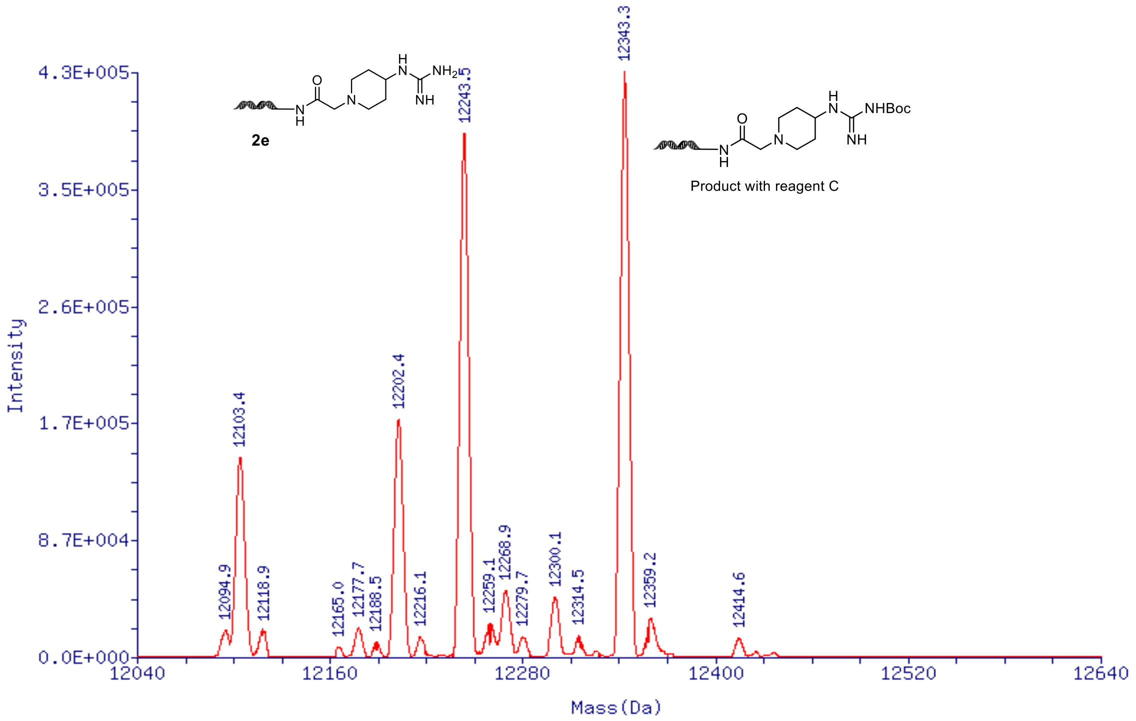

2e
Product with reagent C
Intensity
Mass(Da)
4.3E+005
3.5E+005
2.6E+005
1.7E+005
8.7E+004
0.0E+000
12040
12160
12280
12400
12520
12640
12094.9
12103.4
12118.9
12165.0
12177.7
12188.5
12202.4
12216.1
12243.5
12259.1
12268.9
12279.7
12300.1
12314.5
12343.3
12359.2
12414.6

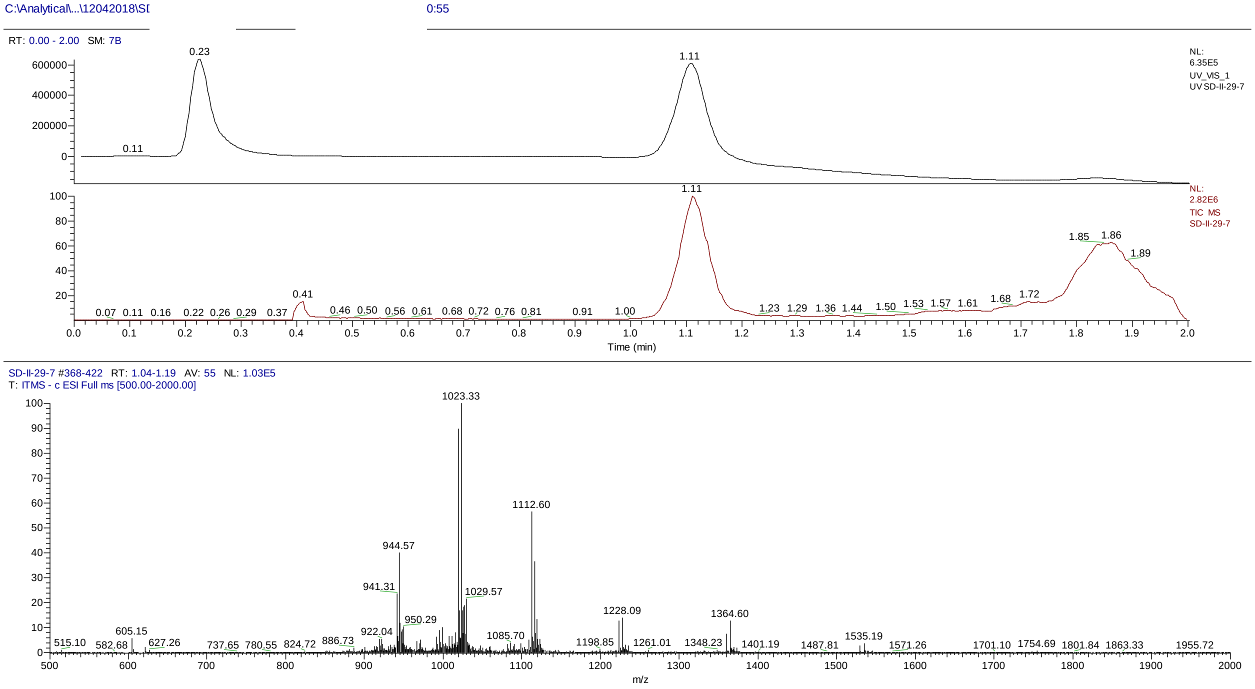
C:\Analytical\...\12042018\SI
0:55
RT: 0.00 - 2.00 SM: 7B
NL: 6.35E5 UV_VIS_1 UV SD-II-29-7
NL: 2.82E6 TIC MS SD-II-29-7
Time (min)
SD-II-29-7 #368-422 RT: 1.04-1.19 AV: 55 NL: 1.03E5
T: ITMS - c ESI Full ms [500.00-2000.00]
1023.33
1112.60
944.57
941.31
1029.57
950.29
1228.09
1364.60
m/z

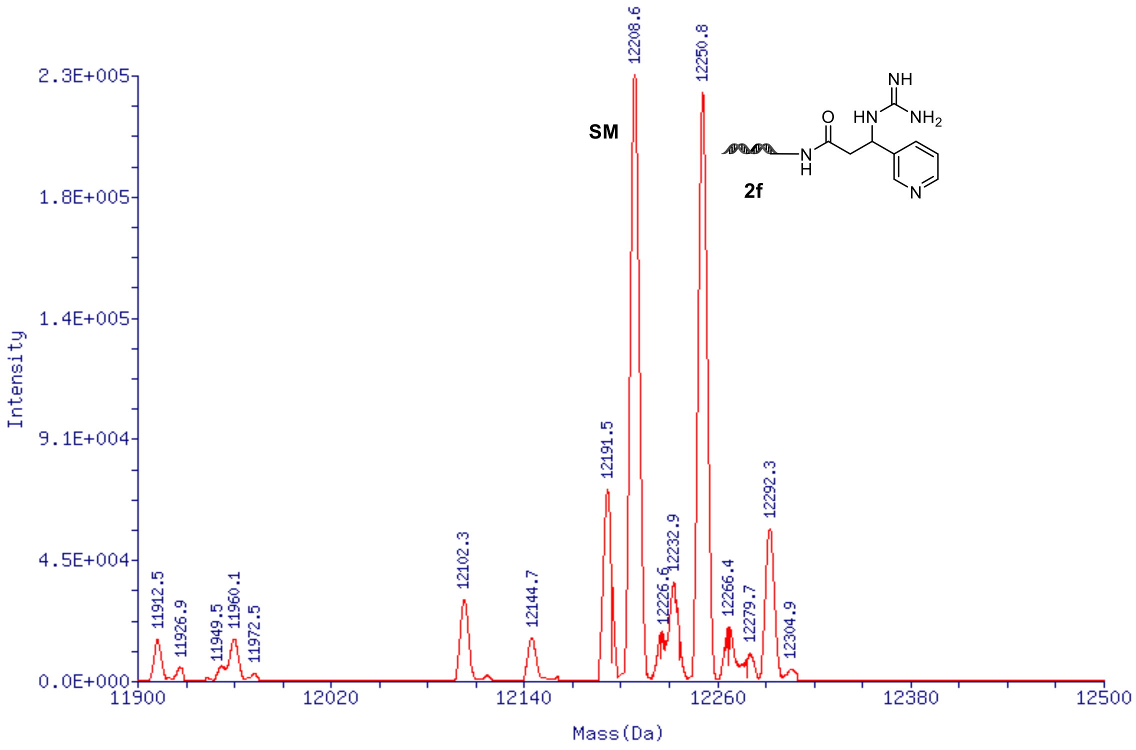
Intensity
Mass(Da)
SM
12208.6
12250.8
12191.5
12292.3
2f

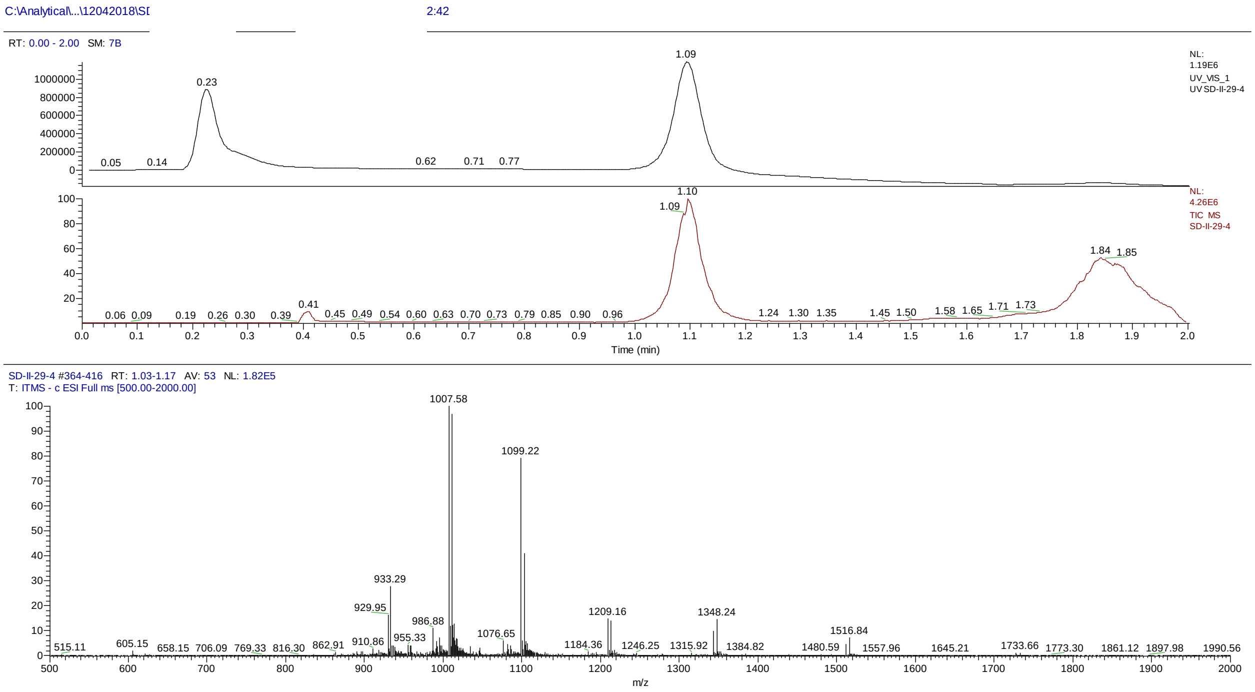
C:\Analytical\...\12042018\SI
2:42
RT: 0.00 - 2.00 SM: 7B
NL: 1.19E6 UV_VIS_1 UV SD-II-29-4
NL: 4.26E6 TIC MS SD-II-29-4
Time (min)
SD-II-29-4 #364-416 RT: 1.03-1.17 AV: 53 NL: 1.82E5
T: ITMS - c ESI Full ms [500.00-2000.00]
1007.58
1099.22
933.29
929.95
986.88
955.33
1209.16
1348.24
1516.84
m/z

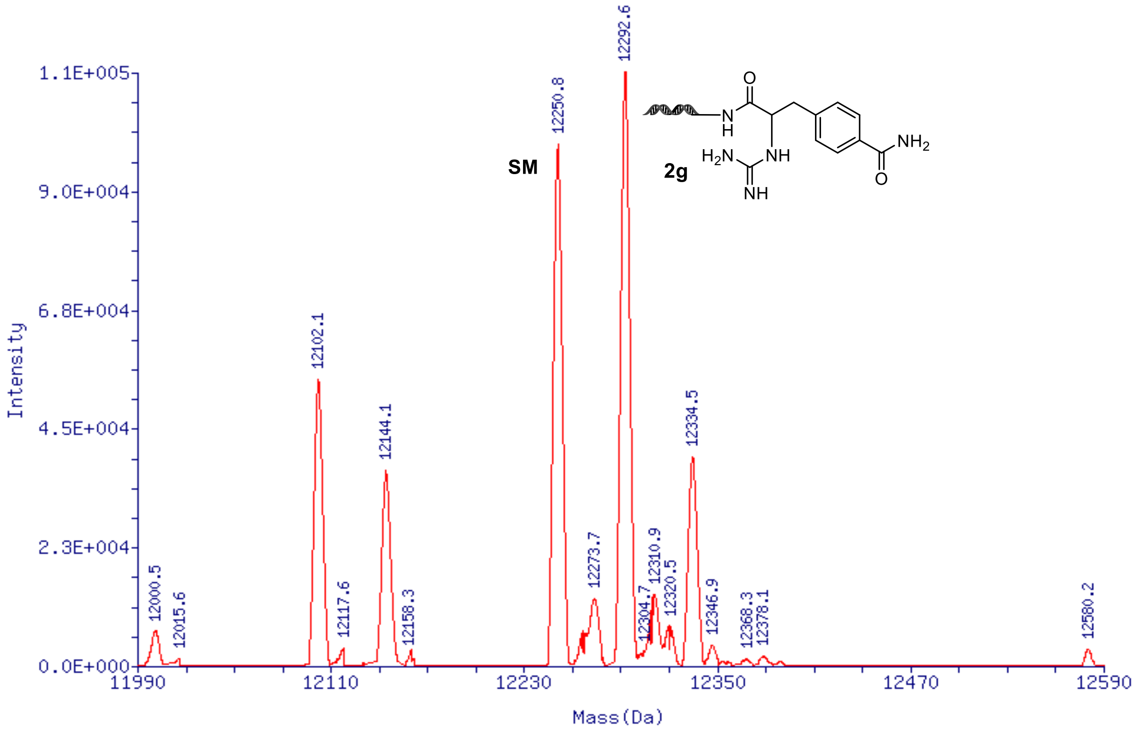
Intensity
Mass(Da)
SM
2g
12292.6
12250.8
12102.1
12144.1
12334.5
12273.7
12310.9
12320.5
12304.7
12000.5
12015.6
12117.6
12158.3
12346.9
12368.3
12378.1
12580.2

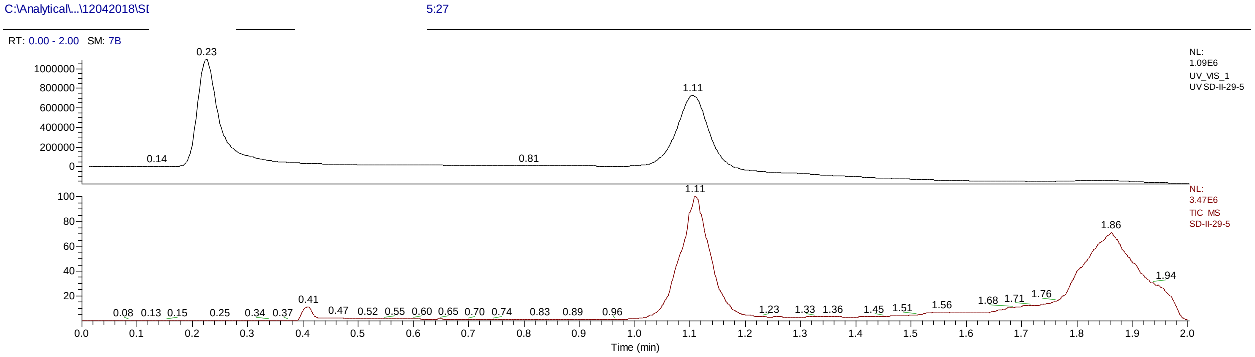
C:\Analytical\...\12042018\SD
5:27
RT: 0.00 - 2.00 SM: 7B
NL: 1.09E6 UV_VIS_1 UV SD-II-29-5
NL: 3.47E6 TIC MS SD-II-29-5
Time (min)

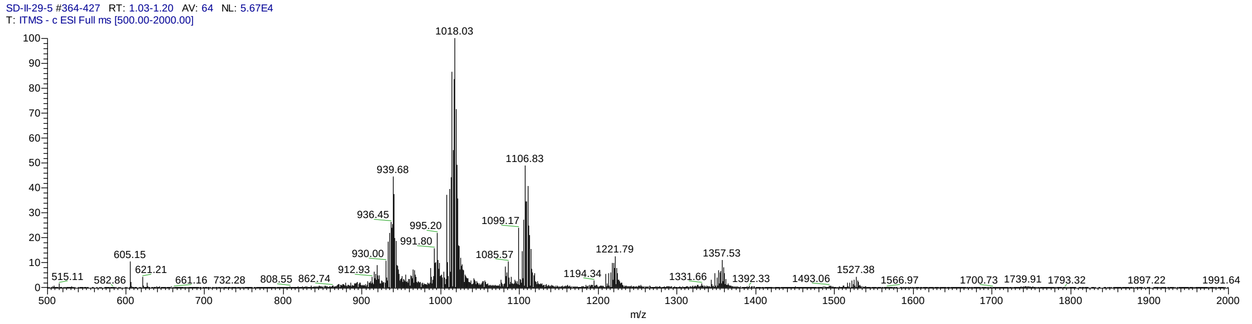
SD-II-29-5 #364-427 RT: 1.03-1.20 AV: 64 NL: 5.67E4
T: ITMS - c ESI Full ms [500.00-2000.00]
1018.03
1106.83
939.68
m/z

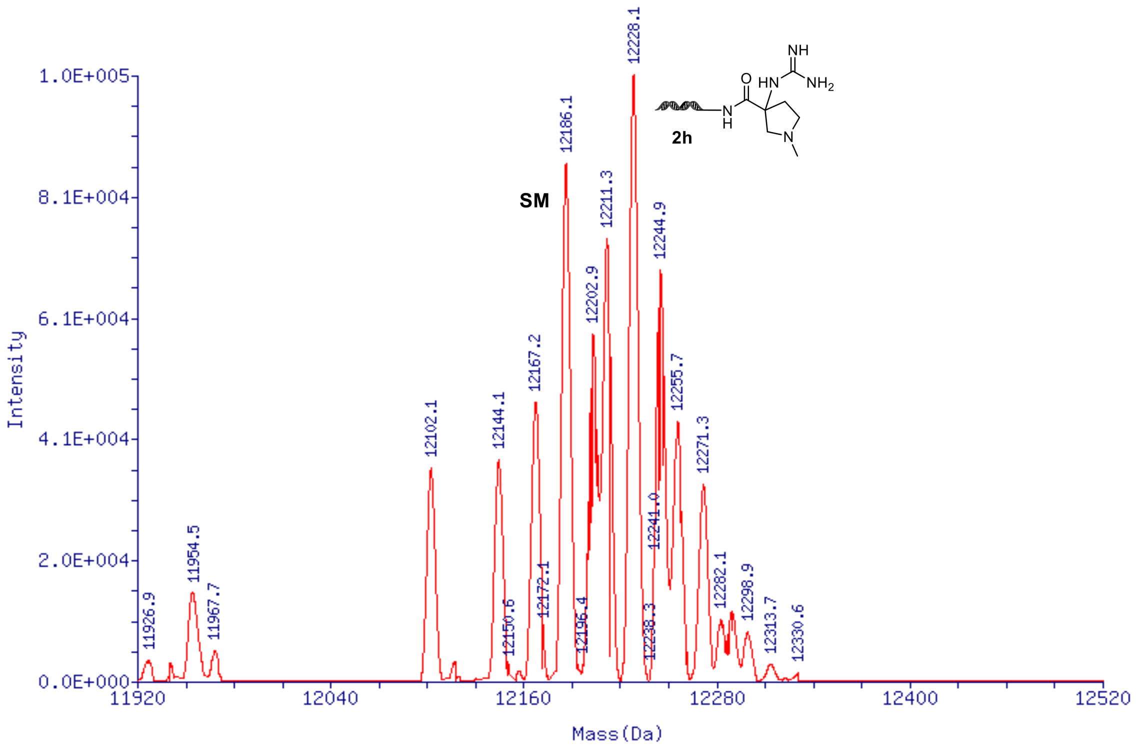
Intensity
Mass(Da)
SM
2h
12228.1
12186.1
12211.3
12244.9
12202.9
12167.2
12255.7
12144.1
12102.1
12271.3
11954.5

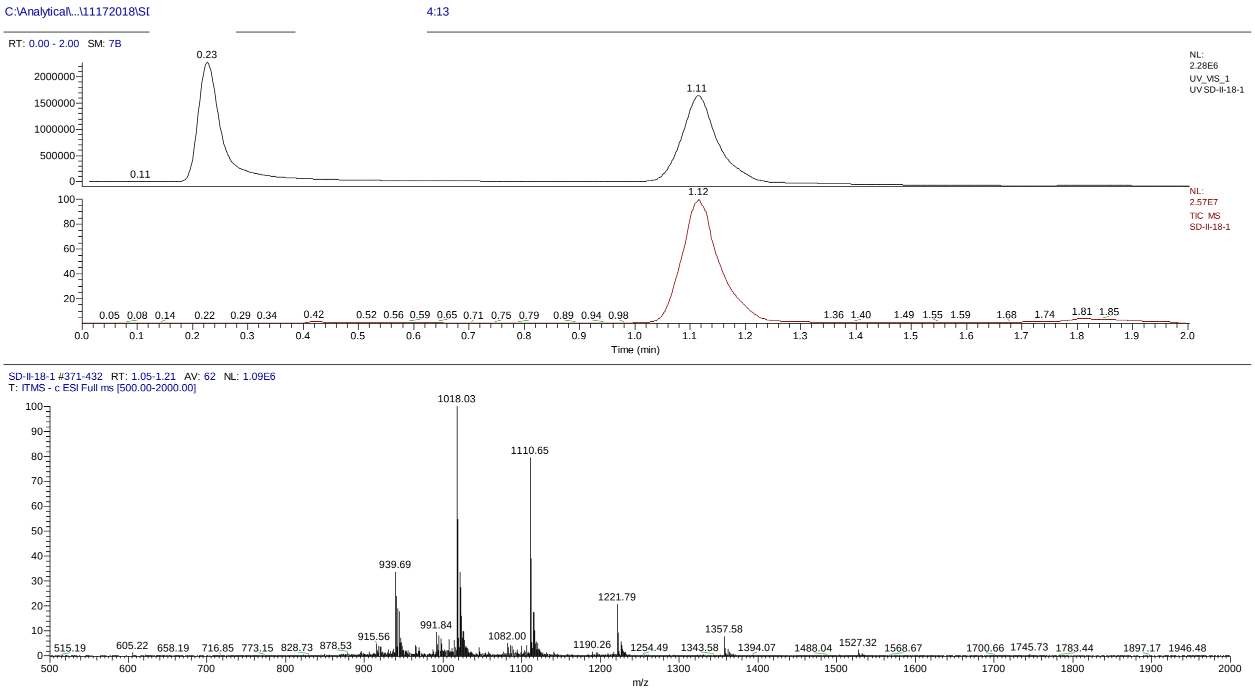
C:\Analytical\...\11172018\SI
4:13
RT: 0.00 - 2.00 SM: 7B
NL: 2.28E6 UV_VIS_1 UV SD-II-18-1
NL: 2.57E7 TIC MS SD-II-18-1
Time (min)
SD-II-18-1 #371-432 RT: 1.05-1.21 AV: 62 NL: 1.09E6
T: ITMS - c ESI Full ms [500.00-2000.00]
1018.03
1110.65
939.69
1221.79
1357.58
m/z

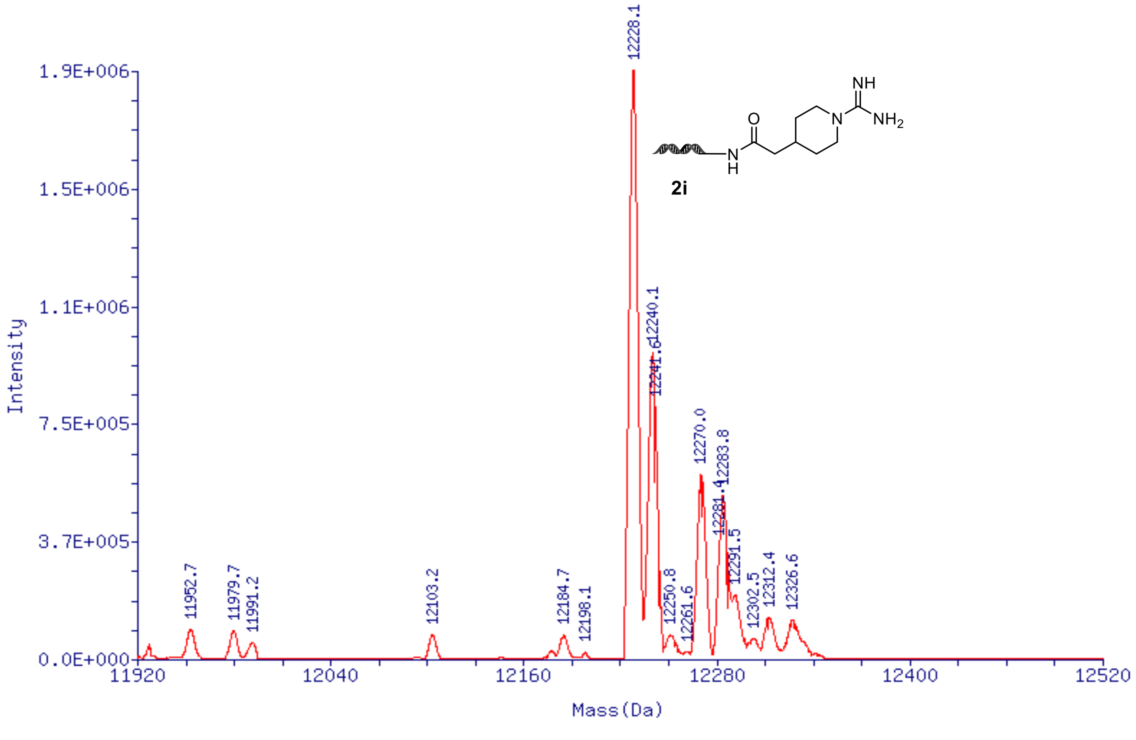
Intensity
Mass(Da)
12228.1
12240.1
12270.0
12283.8
2i

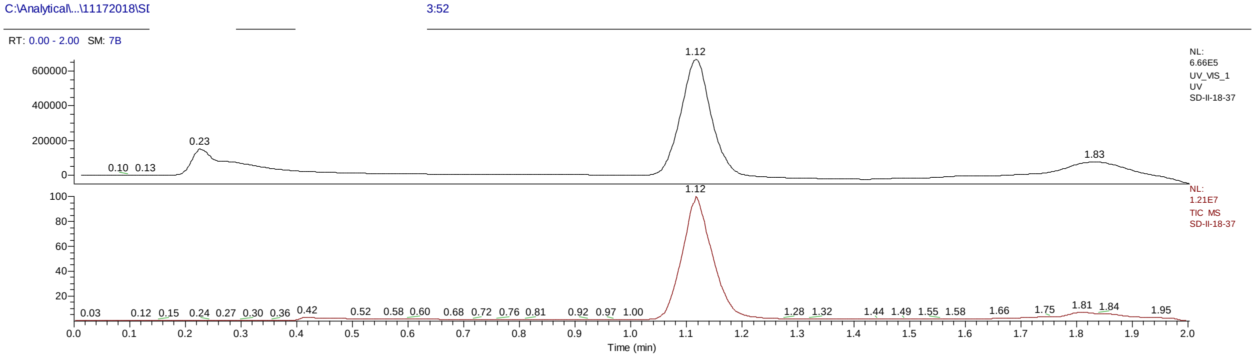

C:\Analytical\...\11172018\SD
3:52
RT: 0.00 - 2.00 SM: 7B
NL: 6.66E5 UV_VIS_1 UV SD-II-18-37
NL: 1.21E7 TIC MS SD-II-18-37
Time (min)

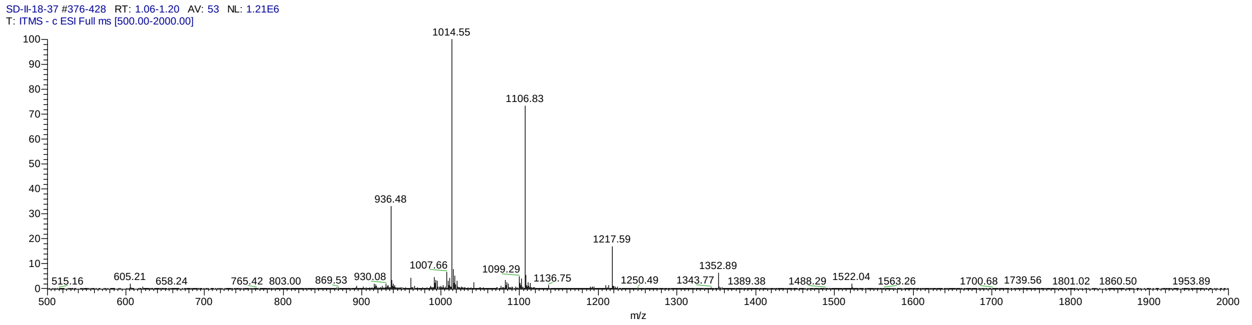

SD-II-18-37 #376-428 RT: 1.06-1.20 AV: 53 NL: 1.21E6
T: ITMS - c ESI Full ms [500.00-2000.00]
1014.55
1106.83
936.48
1217.59
1352.89
m/z

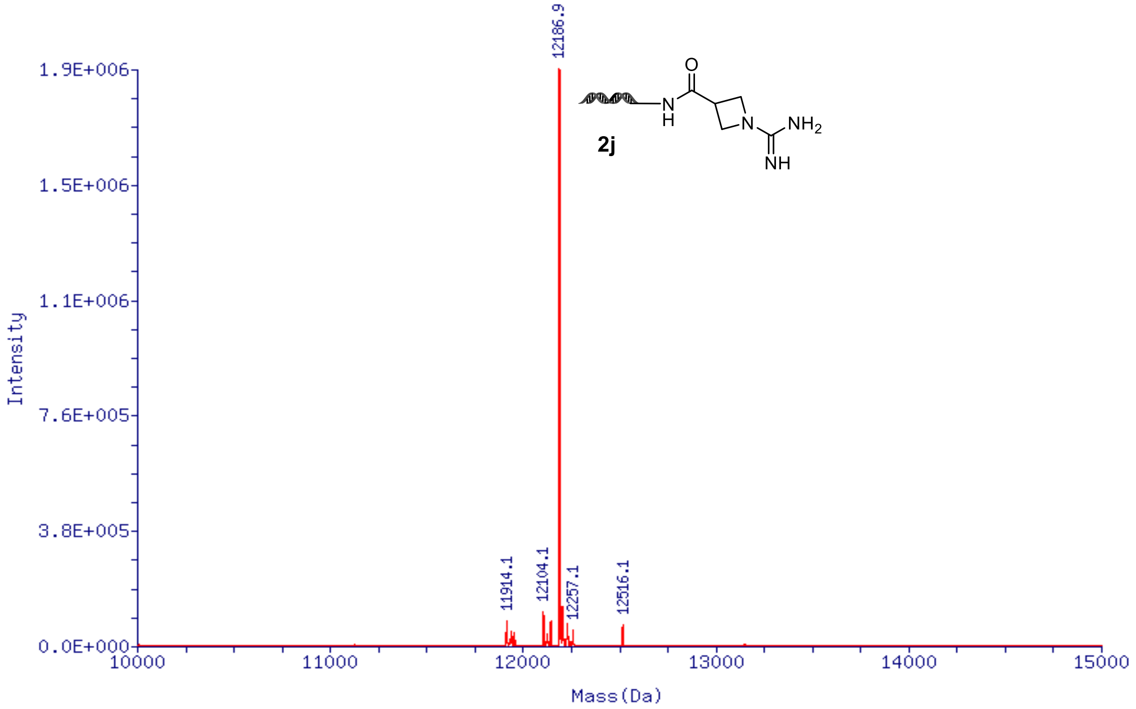

12186.9
11914.1
12104.1
12257.1
12516.1
Intensity
Mass(Da)
2j

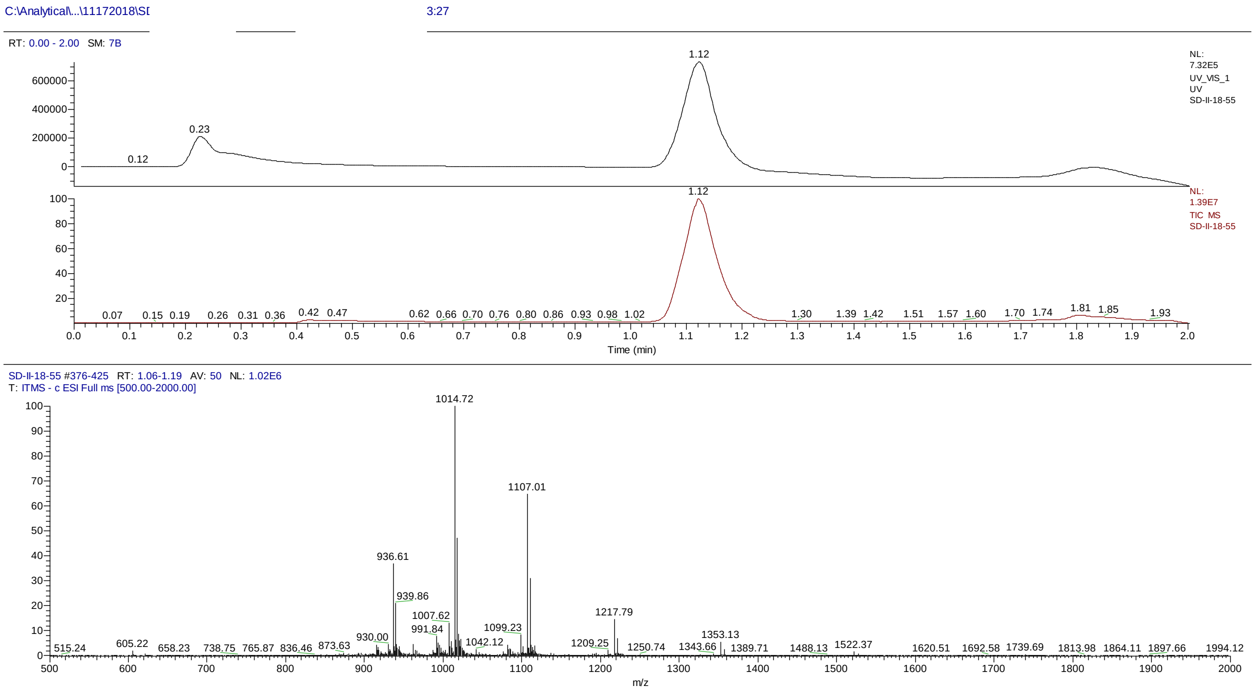

C:\Analytical\...\11172018\SI
3:27
RT: 0.00 - 2.00 SM: 7B
NL: 7.32E5 UV_VIS_1 UV SD-II-18-55
NL: 1.39E7 TIC MS SD-II-18-55
Time (min)
SD-II-18-55 #376-425 RT: 1.06-1.19 AV: 50 NL: 1.02E6
T: ITMS - c ESI Full ms [500.00-2000.00]
1014.72
1107.01
936.61
939.86
1007.62
991.84
1217.79
1353.13
m/z

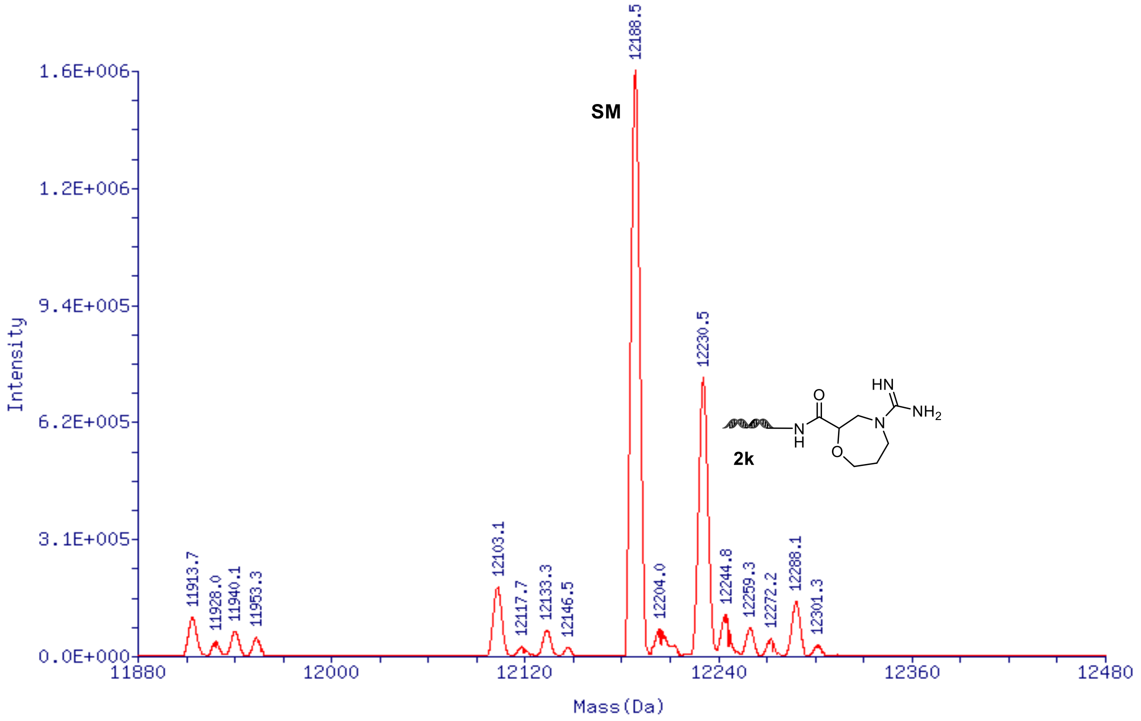

Intensity
Mass(Da)
SM
12188.5
12230.5
2k

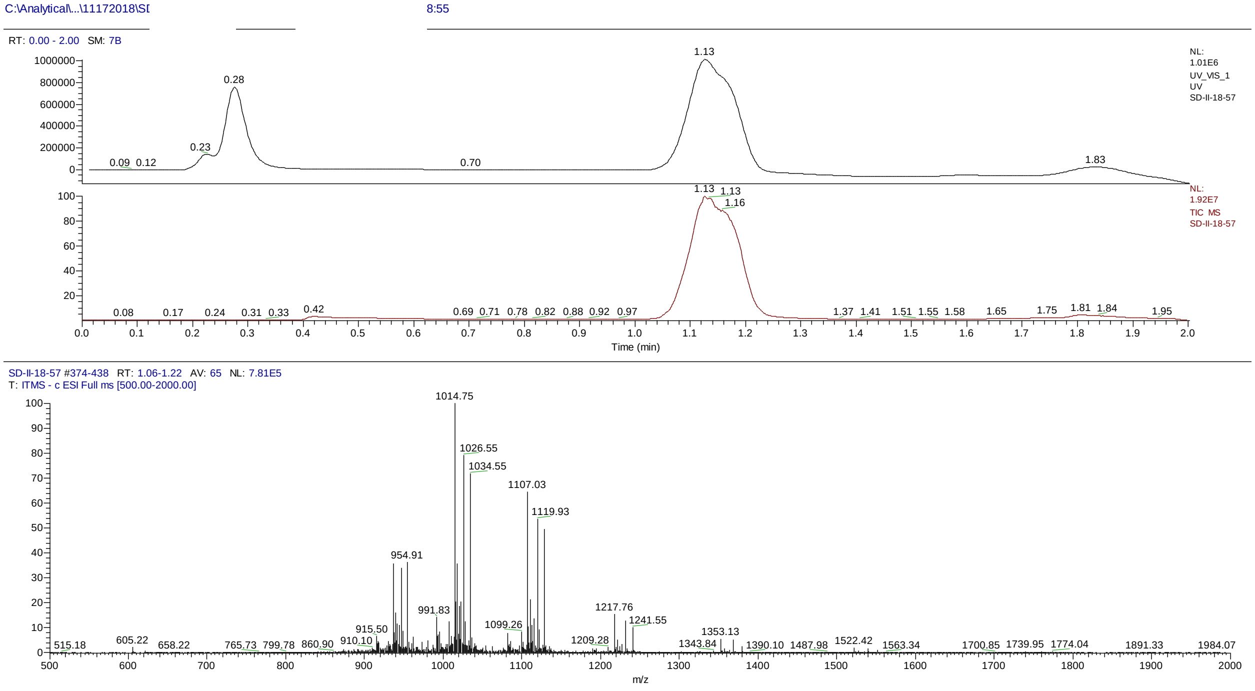
C:\Analytical\...\11172018\SI
8:55
RT: 0.00 - 2.00 SM: 7B
NL: 1.01E6 UV_VIS_1 UV SD-II-18-57
NL: 1.92E7 TIC MS SD-II-18-57
Time (min)
SD-II-18-57 #374-438 RT: 1.06-1.22 AV: 65 NL: 7.81E5
T: ITMS - c ESI Full ms [500.00-2000.00]
1014.75
1026.55
1034.55
1107.03
1119.93
954.91
991.83
915.50
1217.76
1241.55
1353.13
m/z

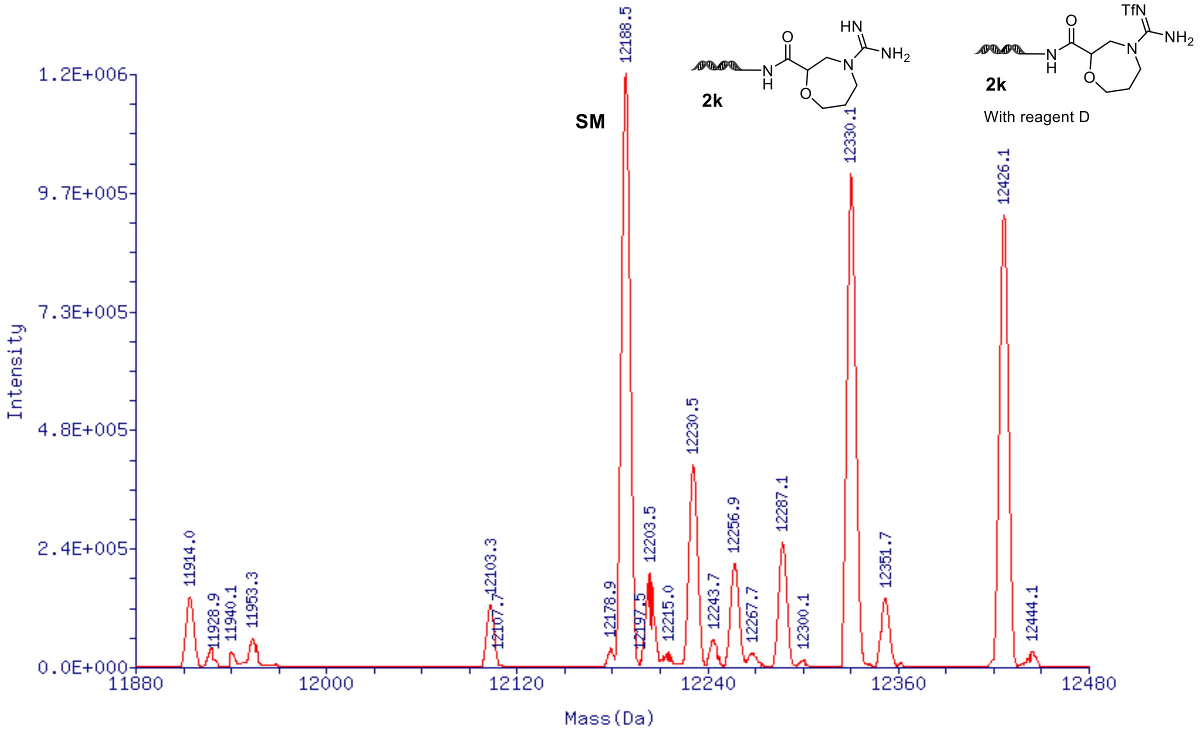
SM
2k
2k
With reagent D
HN
NH2
TfN
Intensity
Mass(Da)
12188.5
12330.1
12426.1
12230.5
12287.1
12256.9
12203.5
11914.0
12103.3
12351.7

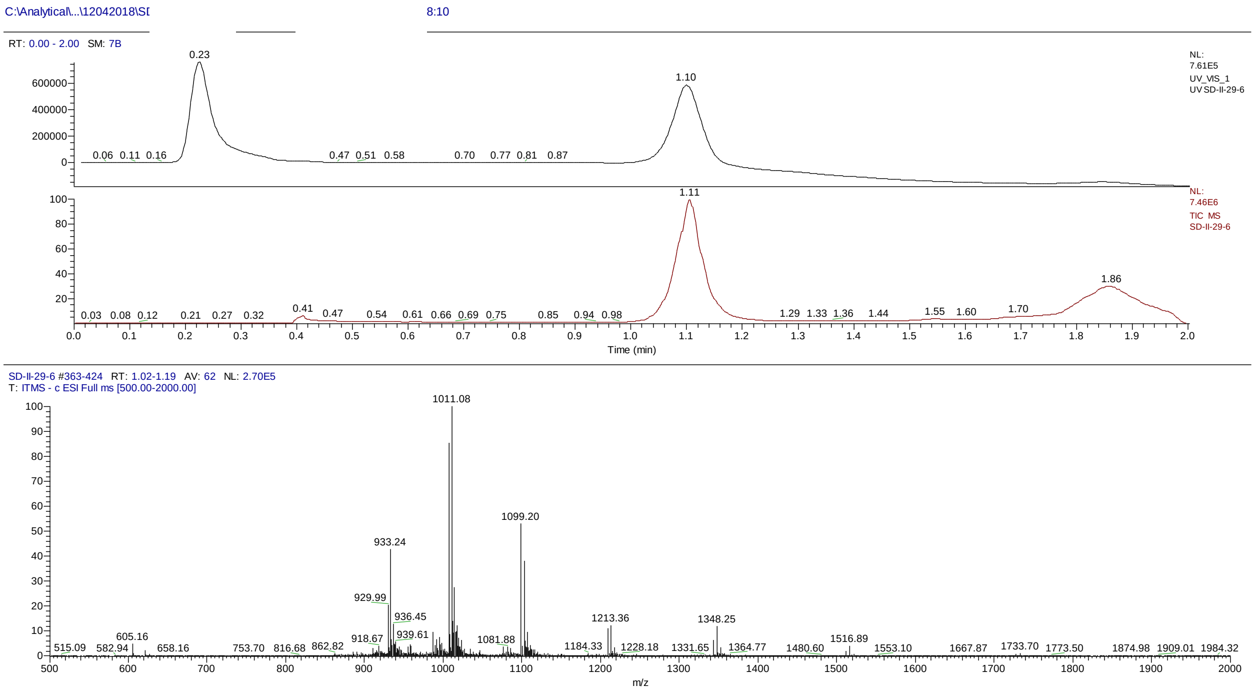
C:\Analytical\...\12042018\SI
8:10
RT: 0.00 - 2.00 SM: 7B
NL: 7.61E5 UV_VIS_1 UV SD-II-29-6
NL: 7.46E6 TIC MS SD-II-29-6
Time (min)
SD-II-29-6 #363-424 RT: 1.02-1.19 AV: 62 NL: 2.70E5
T: ITMS - c ESI Full ms [500.00-2000.00]
1011.08
1099.20
933.24
1213.36
1348.25
m/z

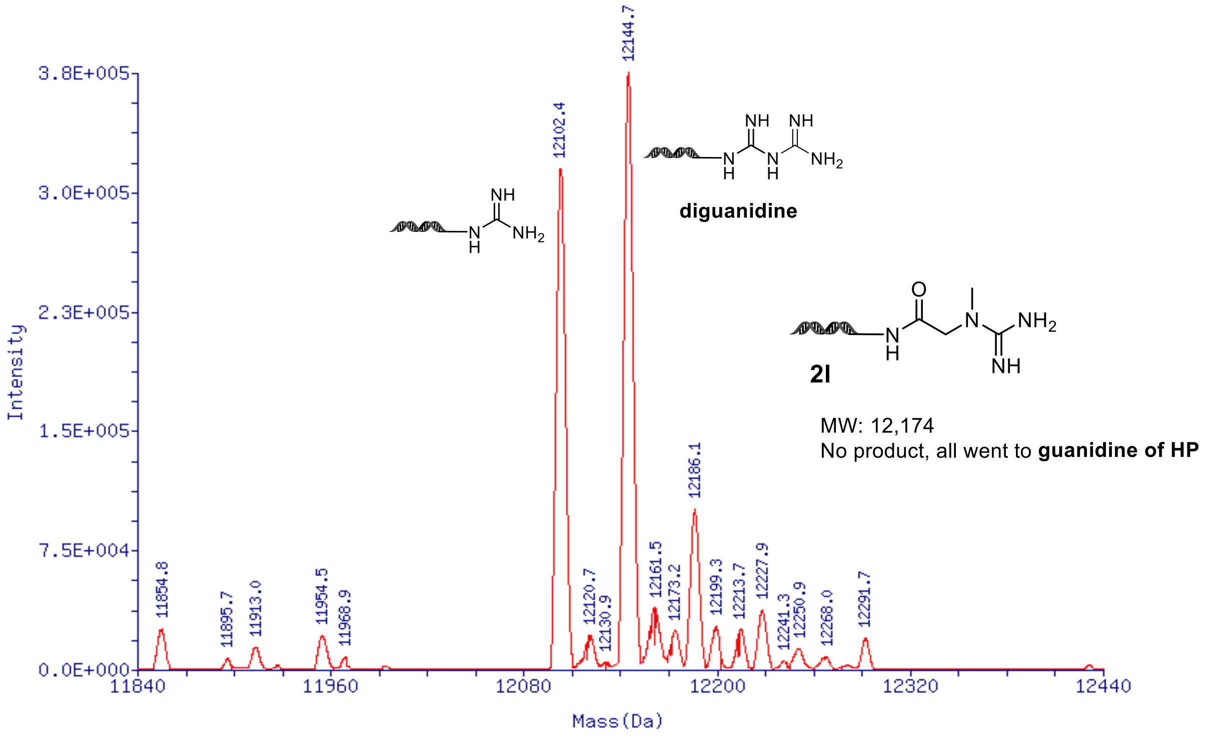
Intensity
Mass(Da)
12102.4
12144.7
12186.1
diguanidine
2l
MW: 12,174
No product, all went to **guanidine of HP**

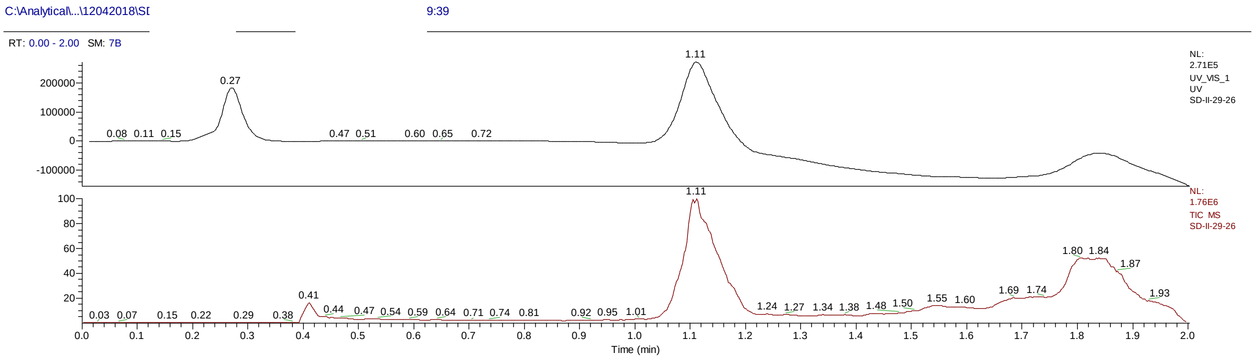
C:\Analytical\...\12042018\SI
9:39
RT: 0.00 - 2.00 SM: 7B
NL: 2.71E5 UV_VIS_1 UV SD-II-29-26
NL: 1.76E6 TIC MS SD-II-29-26
Time (min)

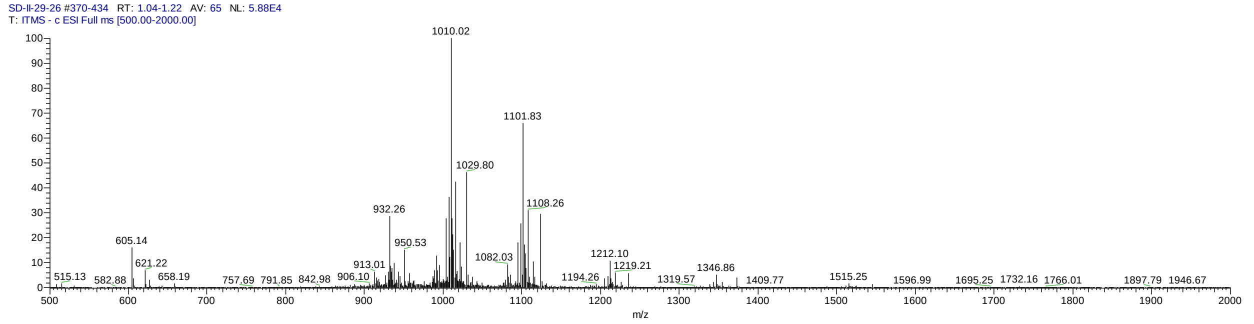
SD-II-29-26 #370-434 RT: 1.04-1.22 AV: 65 NL: 5.88E4
T: ITMS - c ESI Full ms [500.00-2000.00]
1010.02
1101.83
1029.80
1108.26
932.26
605.14
m/z

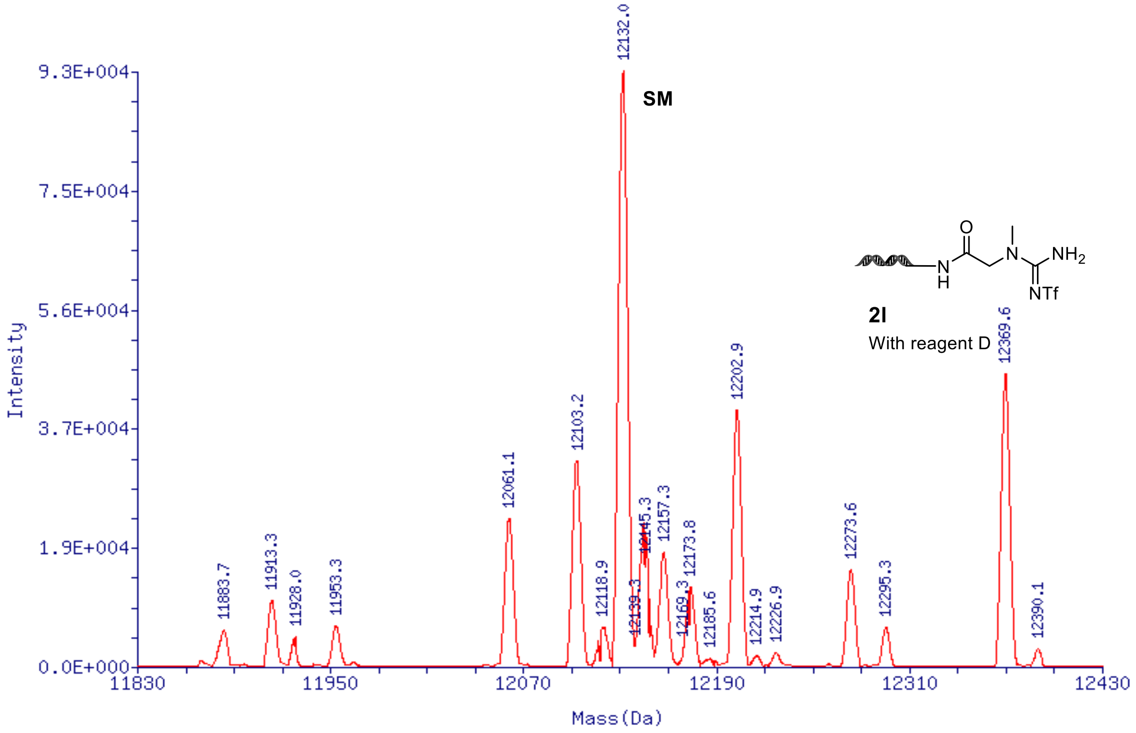
SM
12132.0
12369.6
12202.9
12103.2
12061.1
Intensity
Mass(Da)
2l
With reagent D

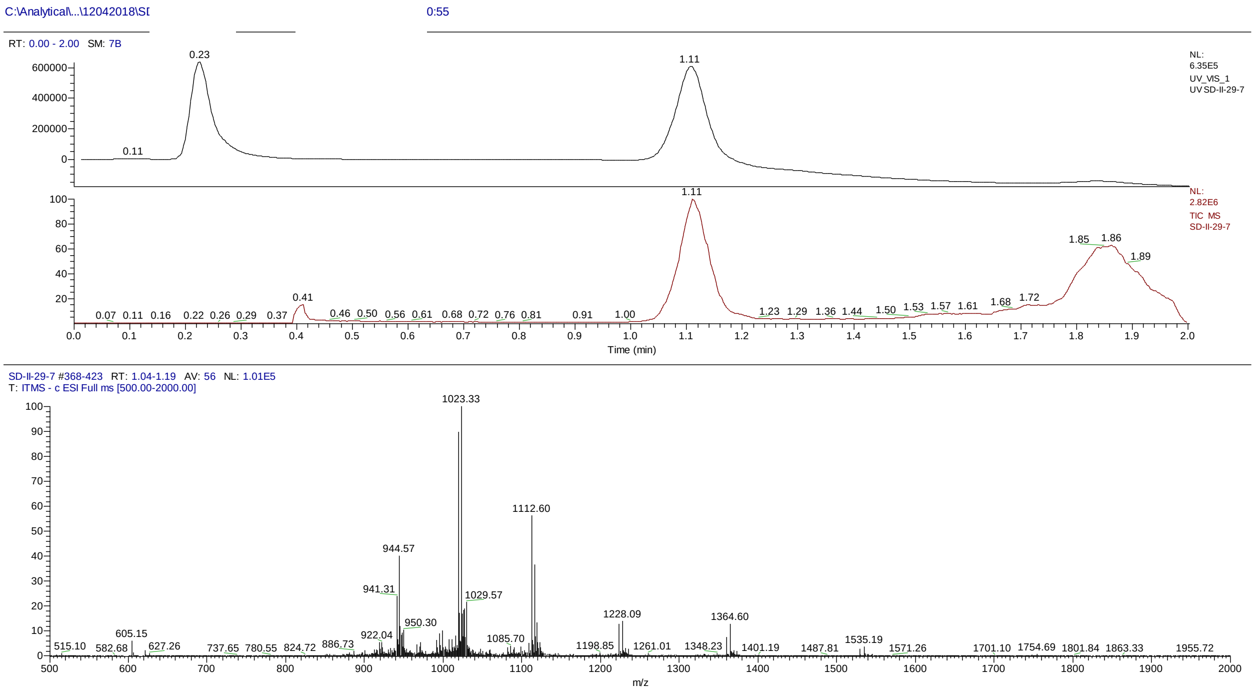
C:\Analytical\...\12042018\SI
0:55
RT: 0.00 - 2.00 SM: 7B
NL: 6.35E5 UV_VIS_1 UV SD-II-29-7
NL: 2.82E6 TIC MS SD-II-29-7
Time (min)
SD-II-29-7 #368-423 RT: 1.04-1.19 AV: 56 NL: 1.01E5
T: ITMS - c ESI Full ms [500.00-2000.00]
1023.33
1112.60
944.57
941.31
1029.57
1228.09
1364.60
m/z

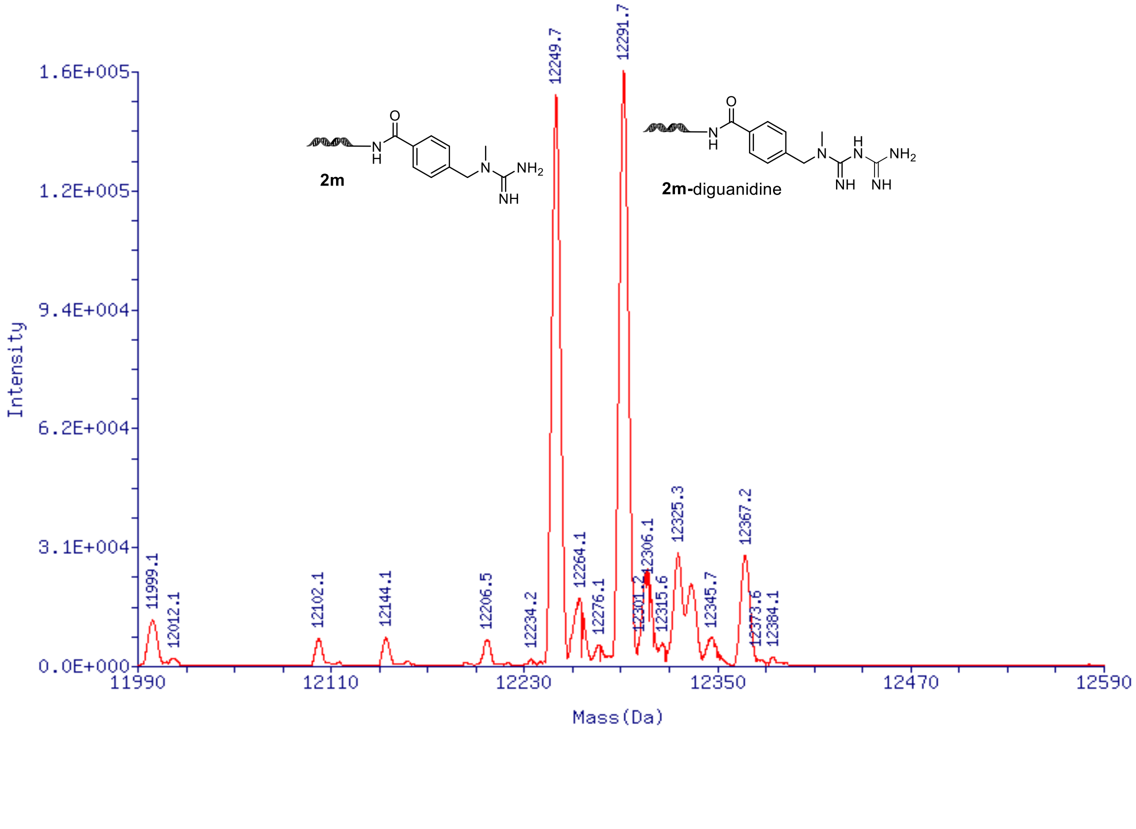
Intensity
Mass (Da)
12249.7
12291.7
2m
2m-diguanidine
12325.3
12367.2
11999.1
12102.1
12144.1
12206.5

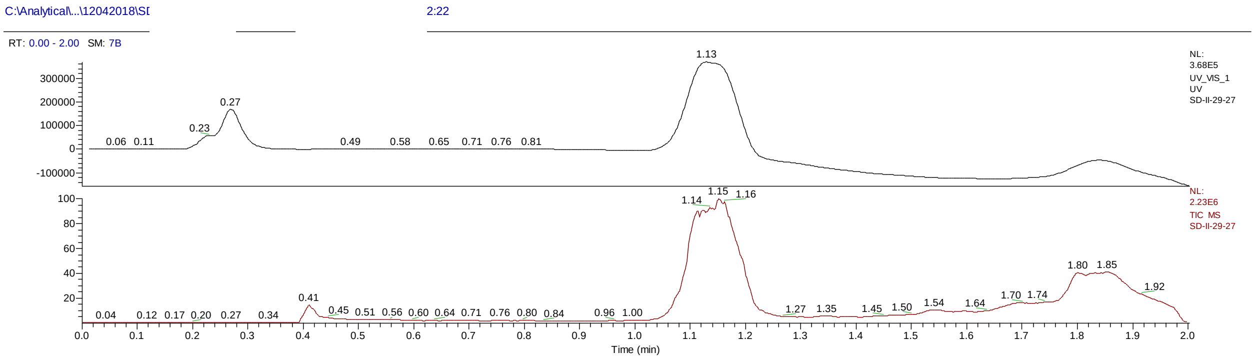
C:\Analytical\...\12042018\SI
2:22
RT: 0.00 - 2.00 SM: 7B
NL: 3.68E5 UV_VIS_1 UV SD-II-29-27
NL: 2.23E6 TIC MS SD-II-29-27
Time (min)

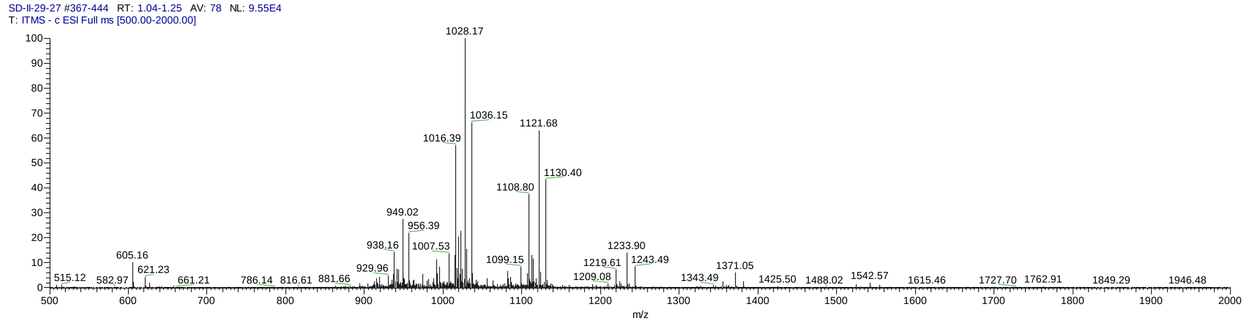
SD-II-29-27 #367-444 RT: 1.04-1.25 AV: 78 NL: 9.55E4
T: ITMS - c ESI Full ms [500.00-2000.00]
1028.17
1036.15
1121.68
1016.39
1130.40
1108.80
949.02
956.39
938.16
1007.53
1233.90
1243.49
1371.05
m/z

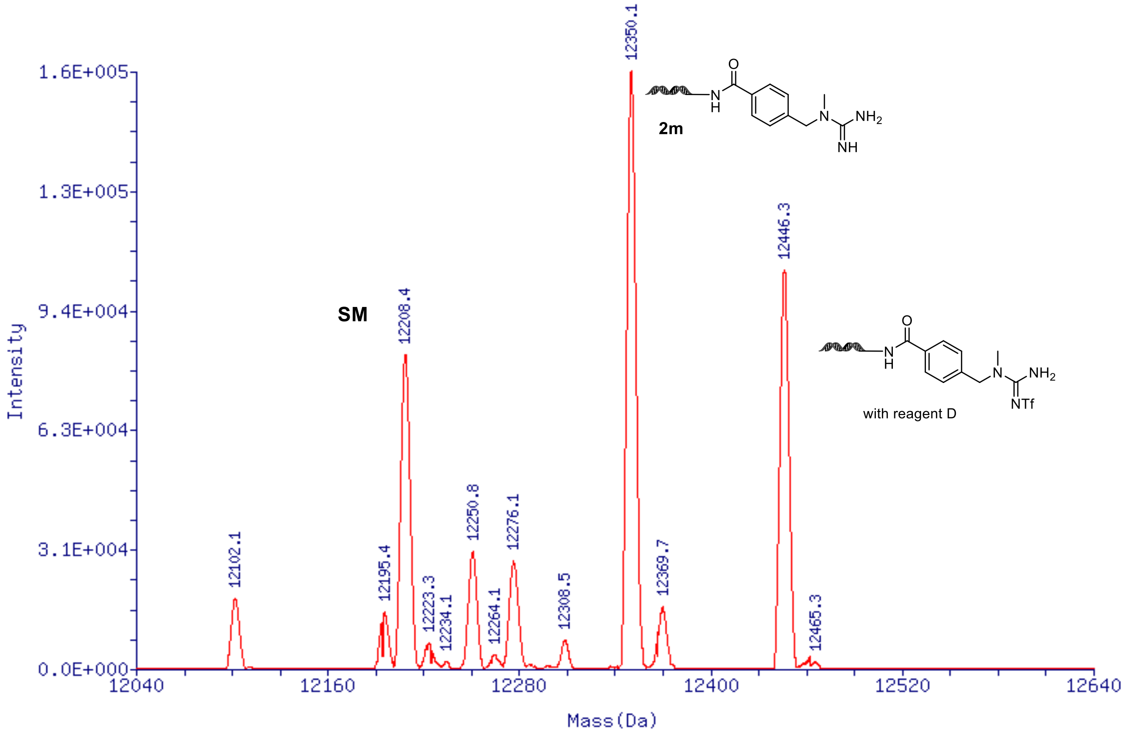
12350.1
2m
12446.3
SM
12208.4
with reagent D
12102.1
12195.4
12223.3
12234.1
12250.8
12264.1
12276.1
12308.5
12369.7
12465.3
Intensity
Mass(Da)

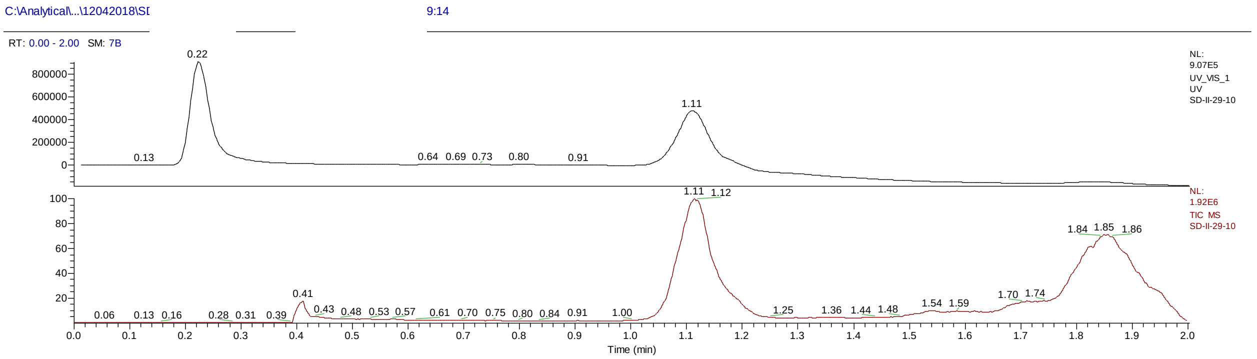
C:\Analytical\...\12042018\SD
9:14
RT: 0.00 - 2.00 SM: 7B
NL: 9.07E5 UV_VIS_1 UV SD-II-29-10
NL: 1.92E6 TIC MS SD-II-29-10
Time (min)

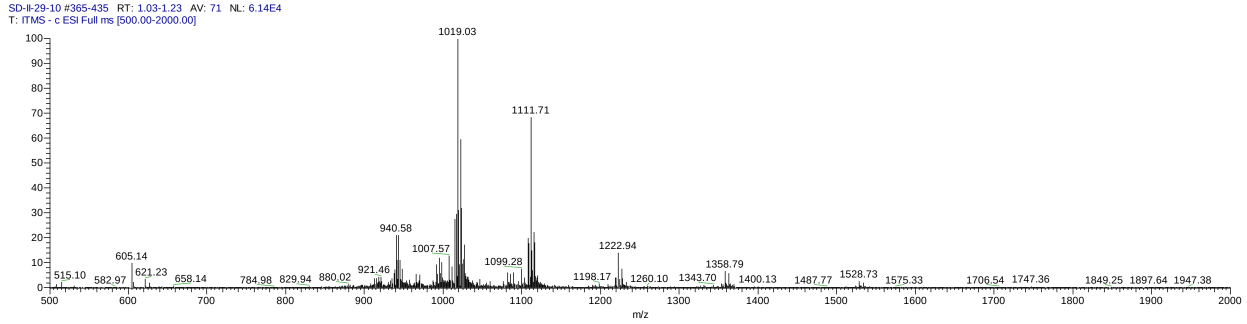
SD-II-29-10 #365-435 RT: 1.03-1.23 AV: 71 NL: 6.14E4
T: ITMS - c ESI Full ms [500.00-2000.00]
1019.03
1111.71
940.58
1222.94
m/z

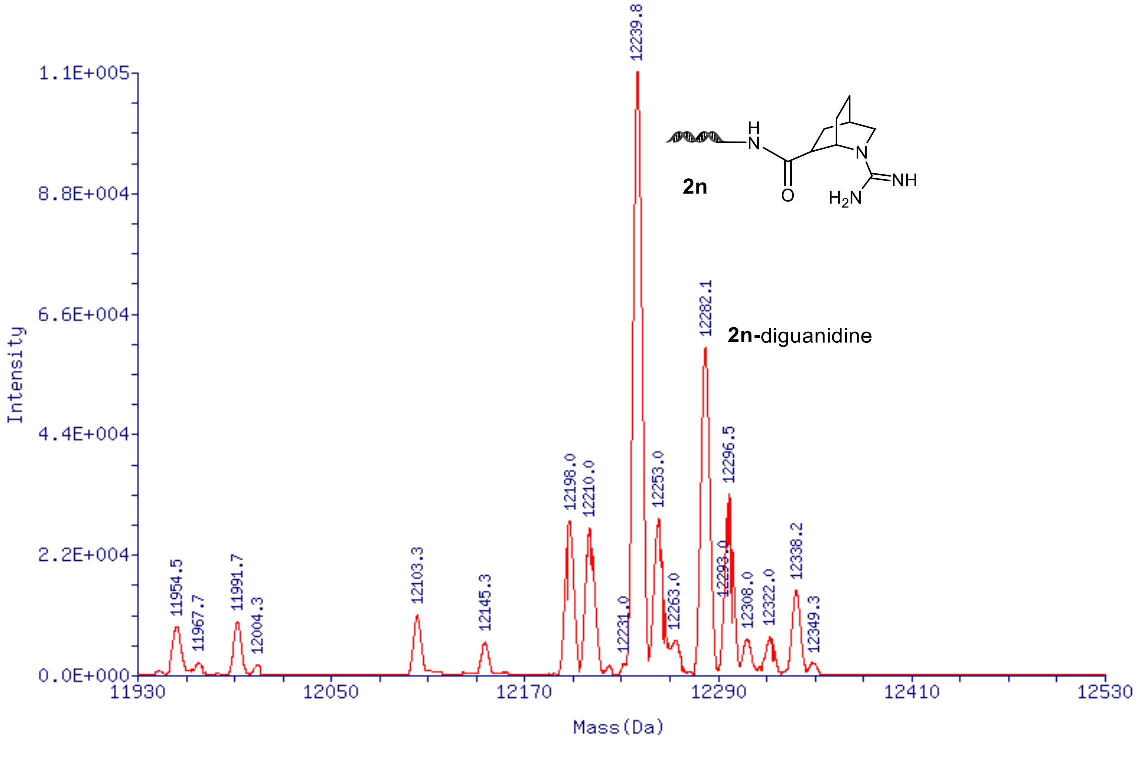
2n
2n-diguanidine
12239.8
12282.1
Intensity
Mass(Da)

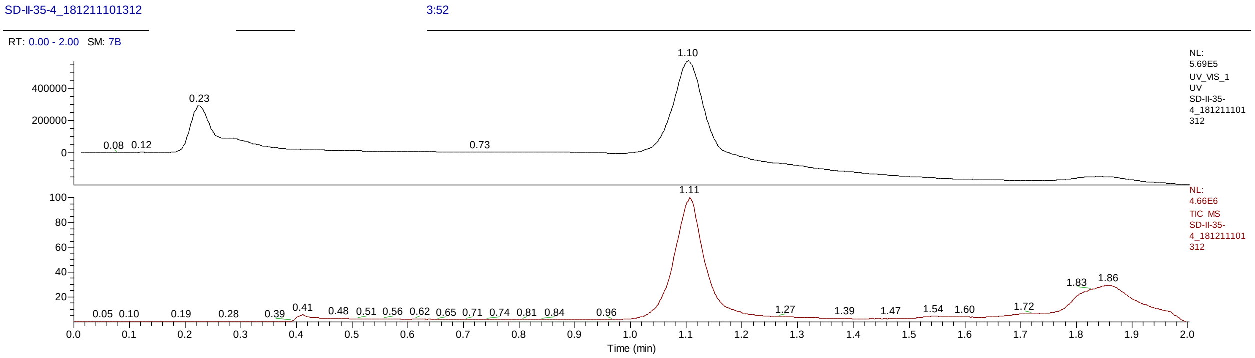
SD-II-35-4_181211101312
3:52
RT: 0.00 - 2.00 SM: 7B
NL: 5.69E5 UV_VIS_1 UV SD-II-35-4_181211101312
NL: 4.66E6 TIC MS SD-II-35-4_181211101312
Time (min)

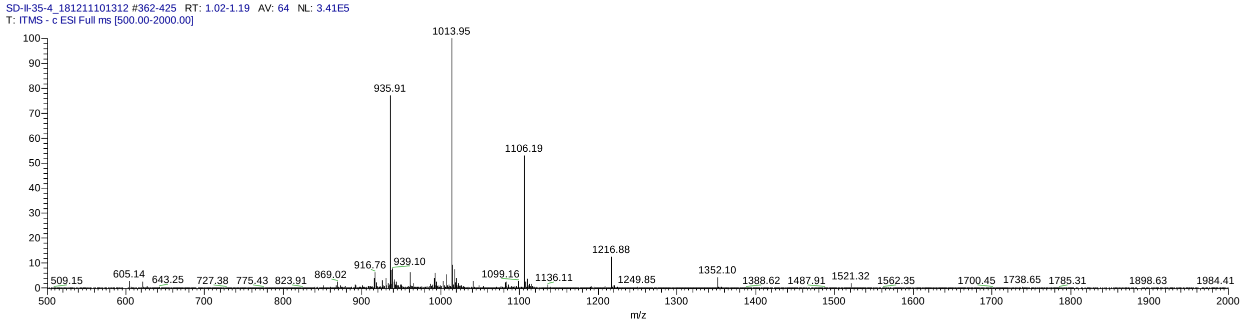
SD-II-35-4_181211101312 #362-425 RT: 1.02-1.19 AV: 64 NL: 3.41E5
T: ITMS - c ESI Full ms [500.00-2000.00]
1013.95
935.91
1106.19
1216.88
m/z

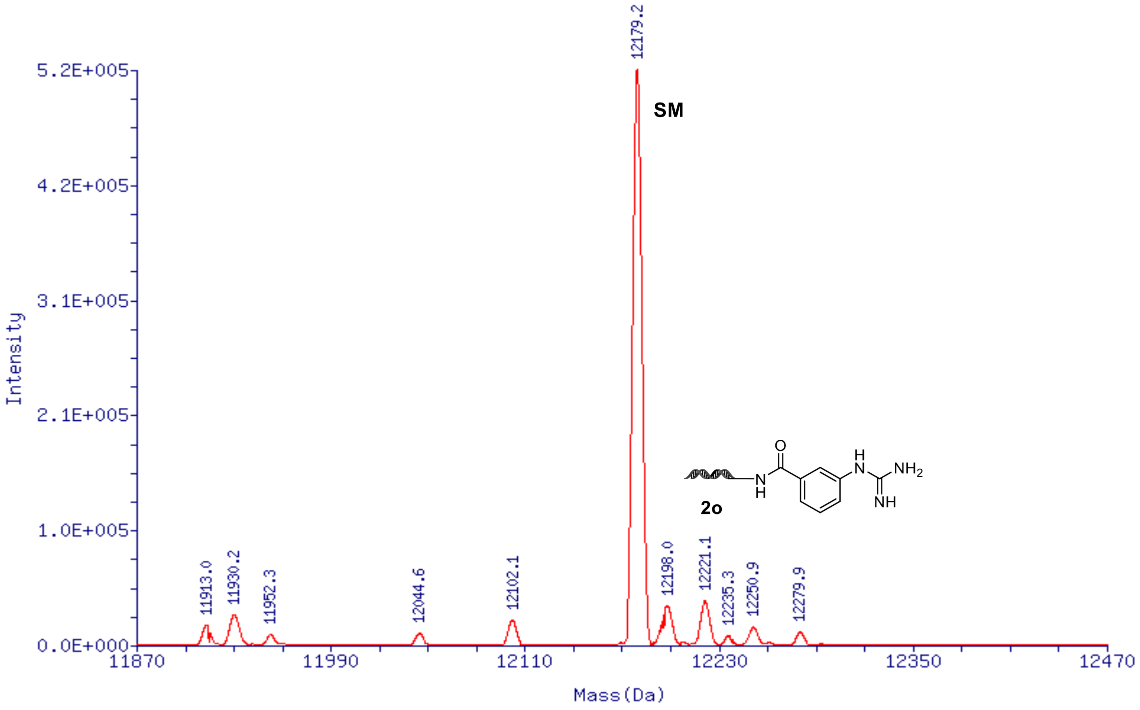
12179.2
SM
2o
11913.0
11930.2
11952.3
12044.6
12102.1
12198.0
12221.1
12235.3
12250.9
12279.9
Intensity
Mass(Da)

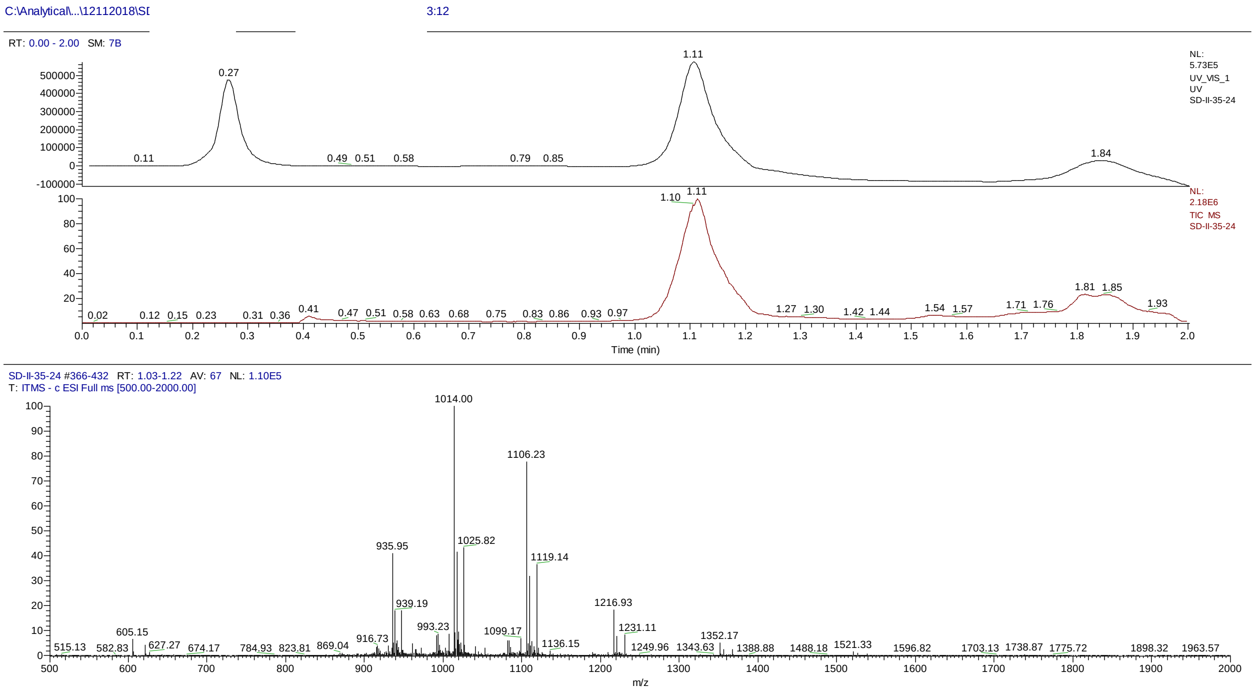
C:\Analytical\...\12112018\SI
3:12
RT: 0.00 - 2.00 SM: 7B
NL: 5.73E5 UV_VIS_1 UV SD-II-35-24
NL: 2.18E6 TIC MS SD-II-35-24
Time (min)
SD-II-35-24 #366-432 RT: 1.03-1.22 AV: 67 NL: 1.10E5
T: ITMS - c ESI Full ms [500.00-2000.00]
1014.00
1106.23
935.95
1025.82
1119.14
939.19
1216.93
993.23
1231.11
1352.17
m/z

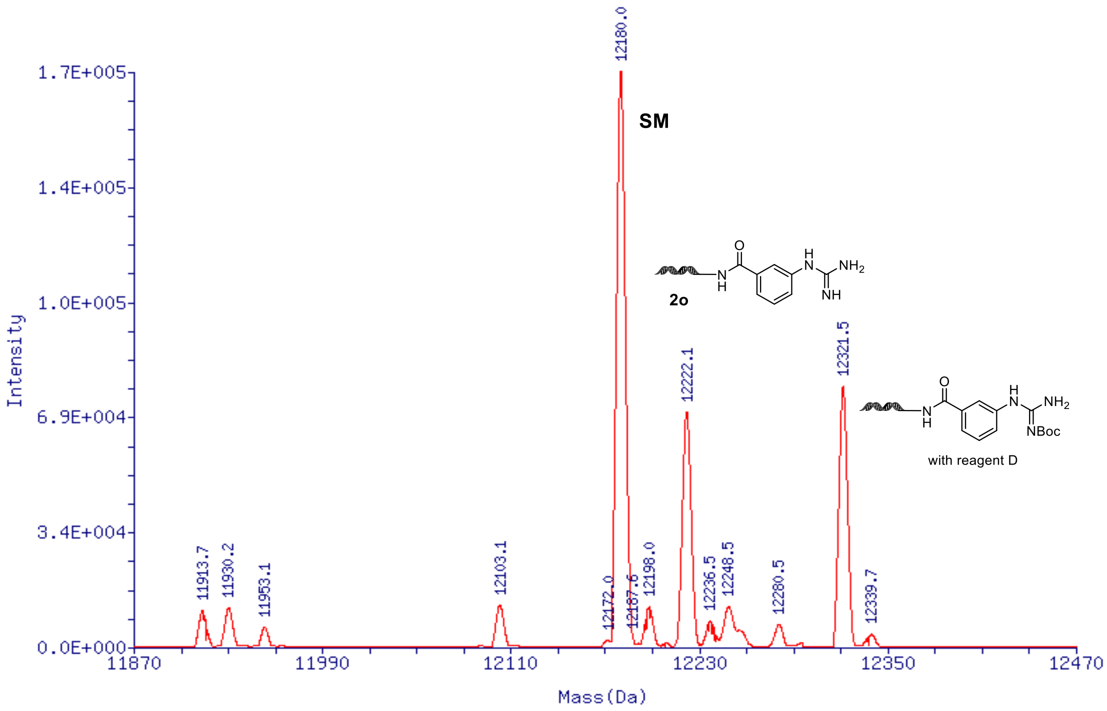
Intensity
Mass (Da)
12180.0
SM
2o
12222.1
12321.5
with reagent D
NBoc
11913.7
11930.2
11953.1
12103.1
12172.0
12187.6
12198.0
12236.5
12248.5
12280.5
12339.7

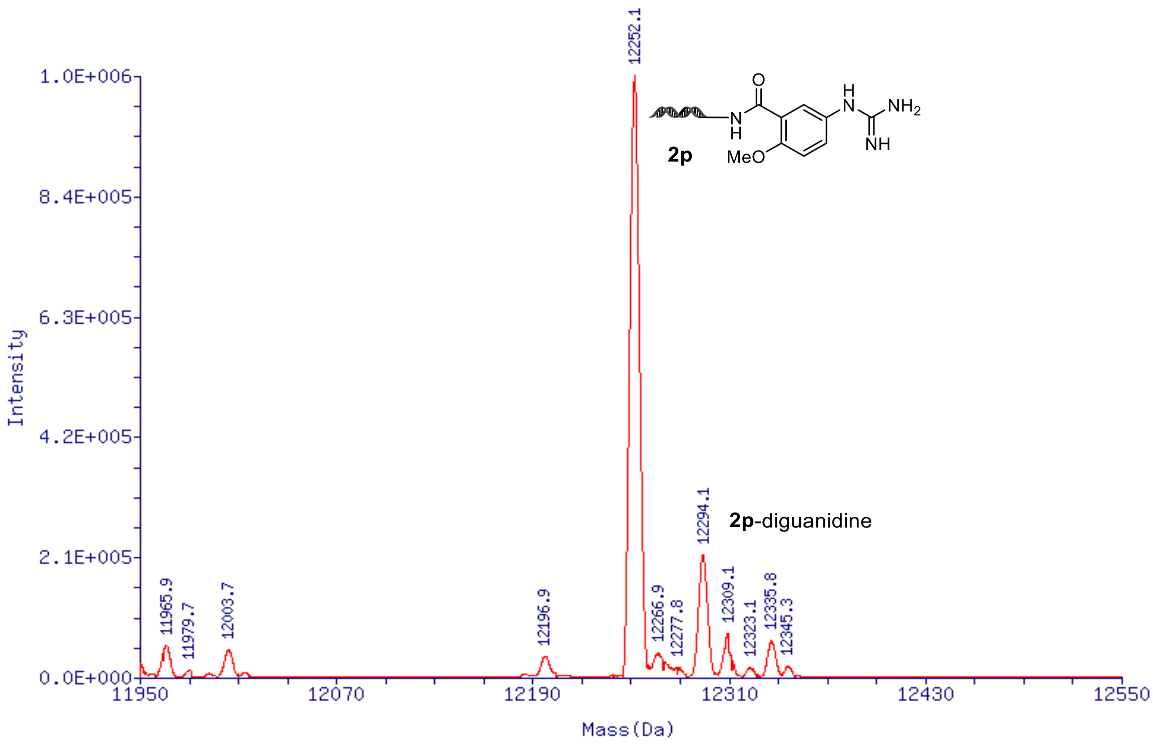

12252.1
1.0E+006
8.4E+005
6.3E+005
4.2E+005
2.1E+005
0.0E+000
Intensity
2p
MeO
2p-diguanidine
12294.1
11965.9
11979.7
12003.7
12196.9
12266.9
12277.8
12309.1
12323.1
12335.8
12345.3
11950
12070
12190
12310
12430
12550
Mass(Da)

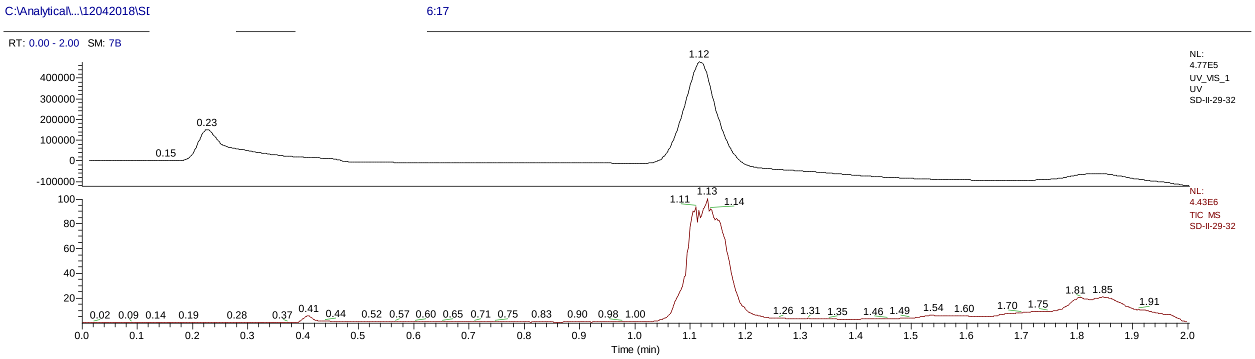
C:\Analytical\...\12042018\SI
6:17
RT: 0.00 - 2.00 SM: 7B
NL: 4.77E5 UV_VIS_1 UV SD-II-29-32
NL: 4.43E6 TIC MS SD-II-29-32
Time (min)

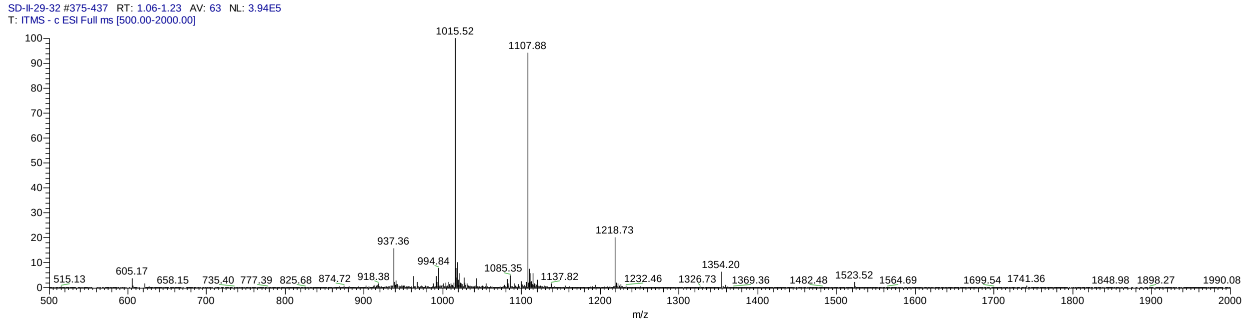
SD-II-29-32 #375-437 RT: 1.06-1.23 AV: 63 NL: 3.94E5
T: ITMS - c ESI Full ms [500.00-2000.00]
1015.52
1107.88
1218.73
937.36
m/z

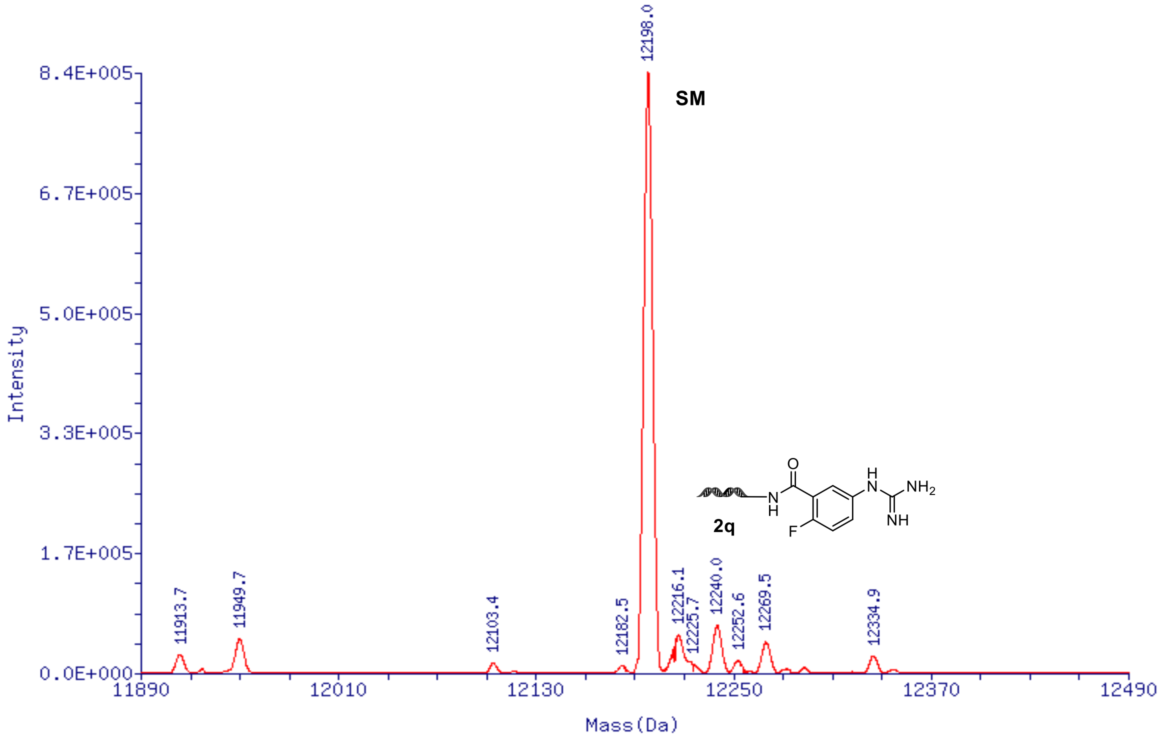
12198.0
SM
2q
Intensity
Mass(Da)

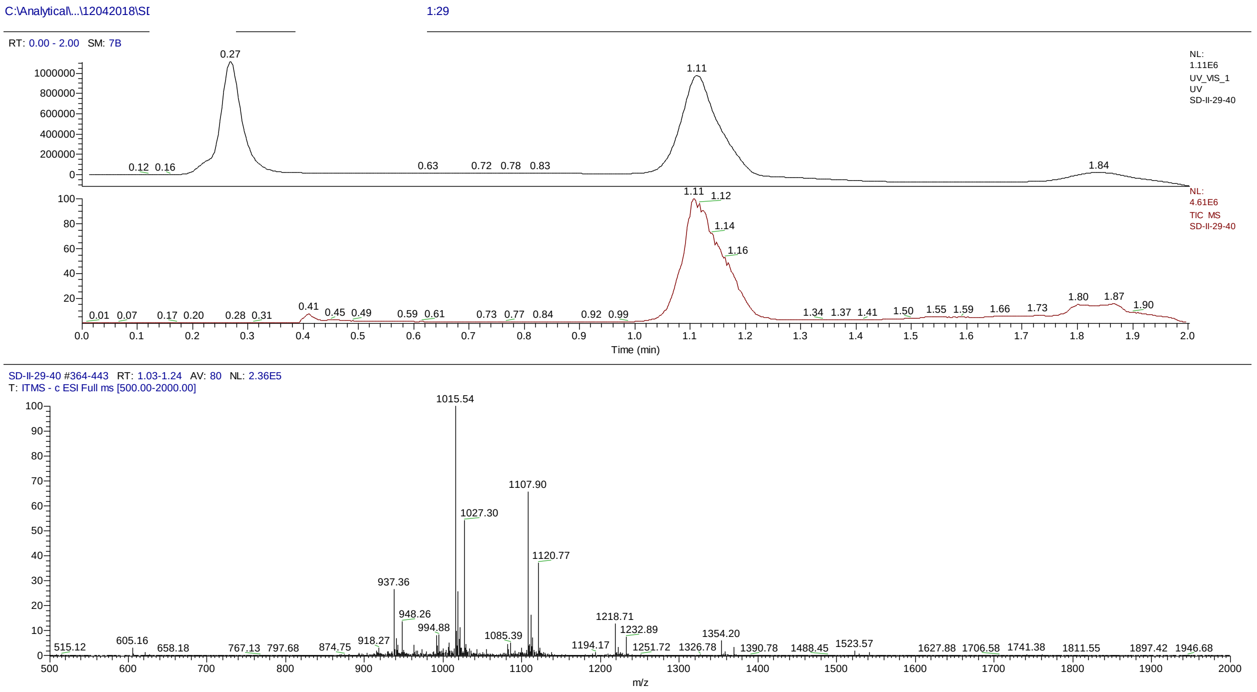
C:\Analytical\...\12042018\SI
1:29
RT: 0.00 - 2.00 SM: 7B
NL: 1.11E6 UV_VIS_1 UV SD-II-29-40
NL: 4.61E6 TIC MS SD-II-29-40
Time (min)
SD-II-29-40 #364-443 RT: 1.03-1.24 AV: 80 NL: 2.36E5
T: ITMS - c ESI Full ms [500.00-2000.00]
1015.54
1107.90
1027.30
1120.77
937.36
948.26
994.88
1218.71
1232.89
1354.20
m/z

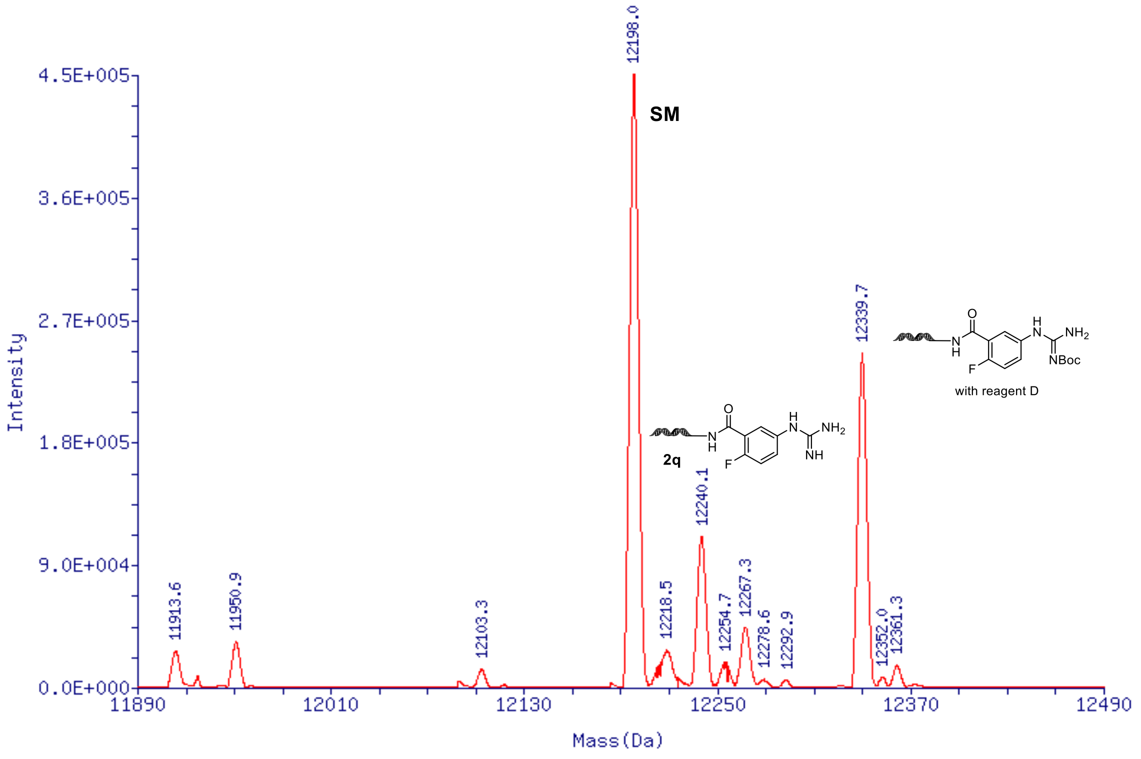
Intensity
Mass(Da)
12198.0
SM
12339.7
with reagent D
2q
12240.1
11913.6
11950.9
12103.3
12218.5
12254.7
12267.3
12278.6
12292.9
12352.0
12361.3

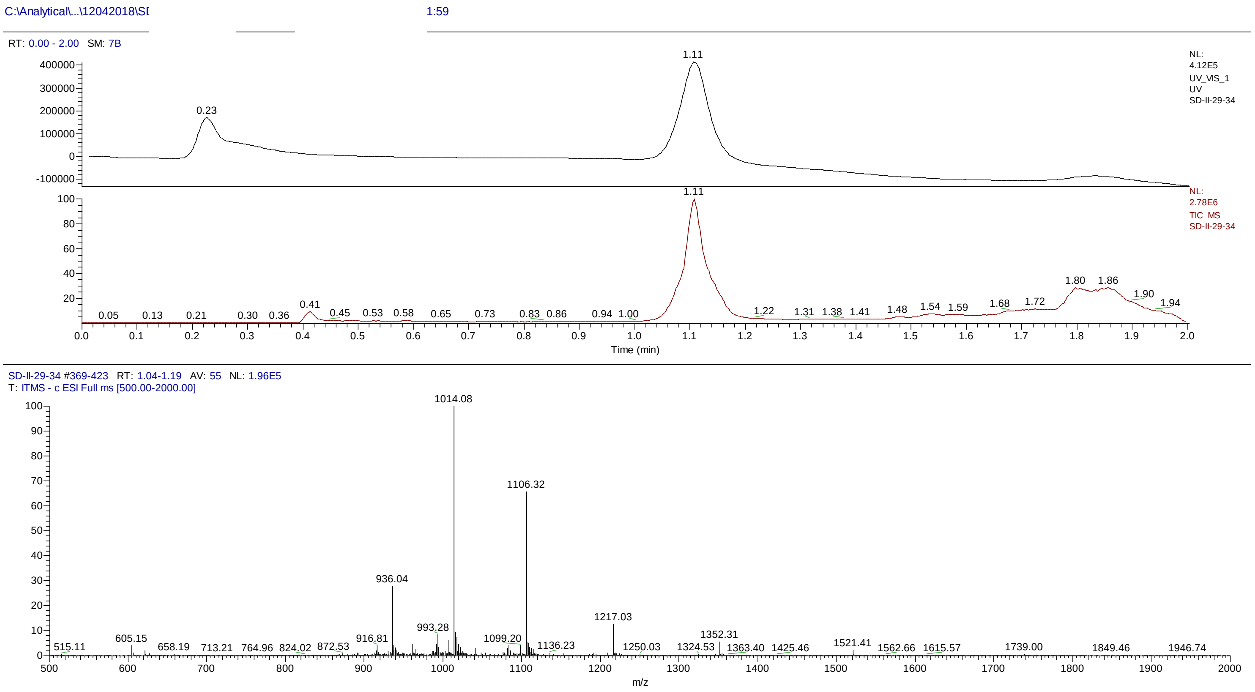
C:\Analytical\...\12042018\SI
1:59
RT: 0.00 - 2.00 SM: 7B
NL: 4.12E5 UV_VIS_1 UV SD-II-29-34
NL: 2.78E6 TIC MS SD-II-29-34
Time (min)
SD-II-29-34 #369-423 RT: 1.04-1.19 AV: 55 NL: 1.96E5
T: ITMS - c ESI Full ms [500.00-2000.00]
1014.08
1106.32
936.04
1217.03
m/z

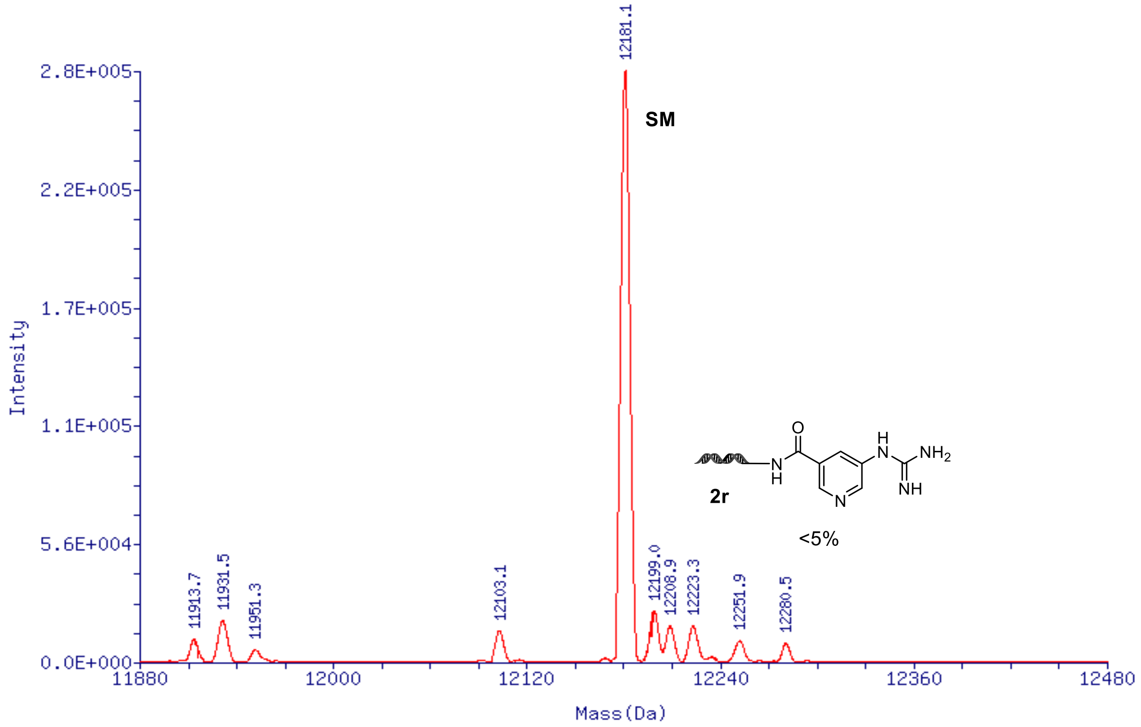
12181.1
SM
Intensity
Mass(Da)
2r
<5%
11913.7
11931.5
11951.3
12103.1
12199.0
12208.9
12223.3
12251.9
12280.5

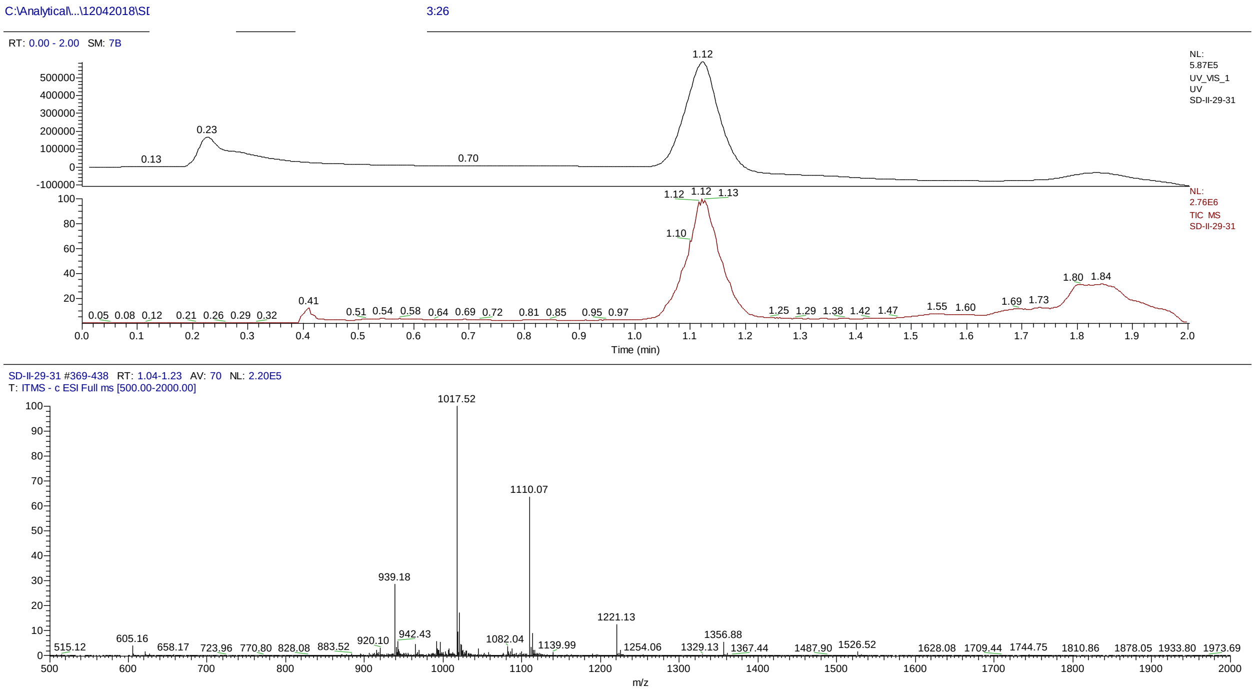
C:\Analytical\...\12042018\SI
3:26
RT: 0.00 - 2.00 SM: 7B
NL: 5.87E5 UV_VIS_1 UV SD-II-29-31
NL: 2.76E6 TIC MS SD-II-29-31
Time (min)
SD-II-29-31 #369-438 RT: 1.04-1.23 AV: 70 NL: 2.20E5
T: ITMS - c ESI Full ms [500.00-2000.00]
1017.52
1110.07
939.18
1221.13
m/z

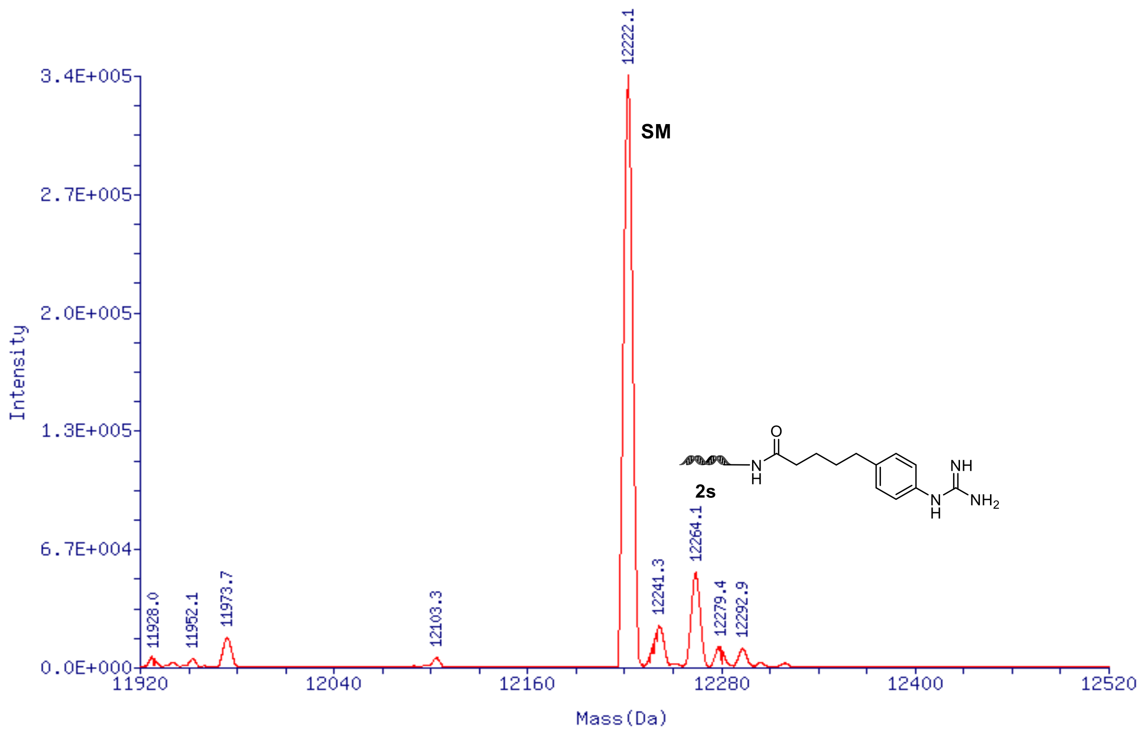
12222.1
SM
2s
12264.1
12241.3
12279.4
12292.9
11928.0
11952.1
11973.7
12103.3
Intensity
Mass(Da)

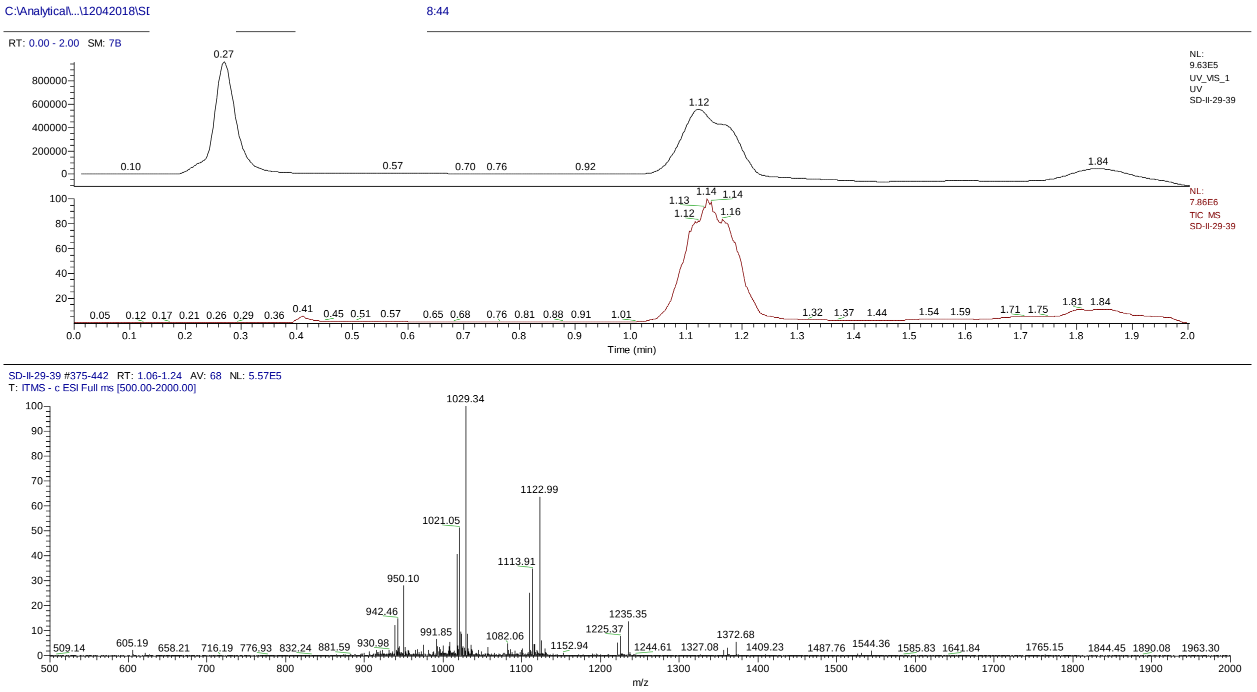
C:\Analytical\...\12042018\SI
8:44
RT: 0.00 - 2.00 SM: 7B
NL: 9.63E5 UV_VIS_1 UV SD-II-29-39
NL: 7.86E6 TIC MS SD-II-29-39
Time (min)
SD-II-29-39 #375-442 RT: 1.06-1.24 AV: 68 NL: 5.57E5
T: ITMS - c ESI Full ms [500.00-2000.00]
1029.34
1122.99
1021.05
1113.91
950.10
942.46
1235.35
1372.68
m/z

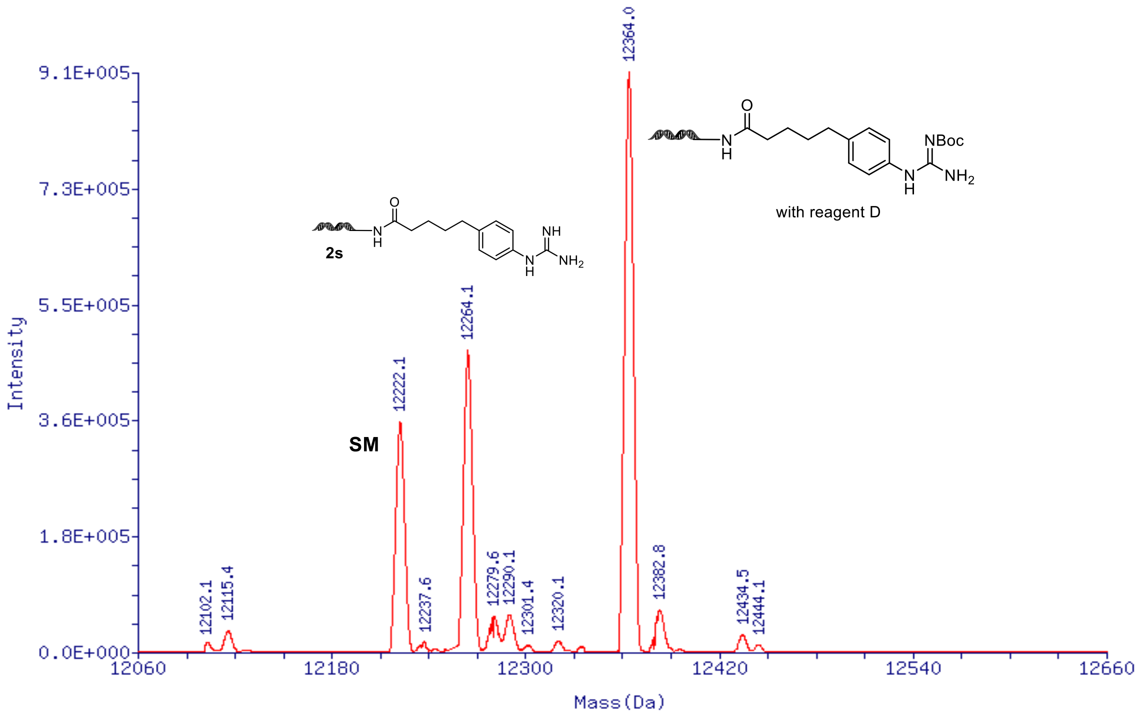
Intensity
Mass(Da)
12364.0
12264.1
12222.1
SM
2s
with reagent D
NBoc
NH

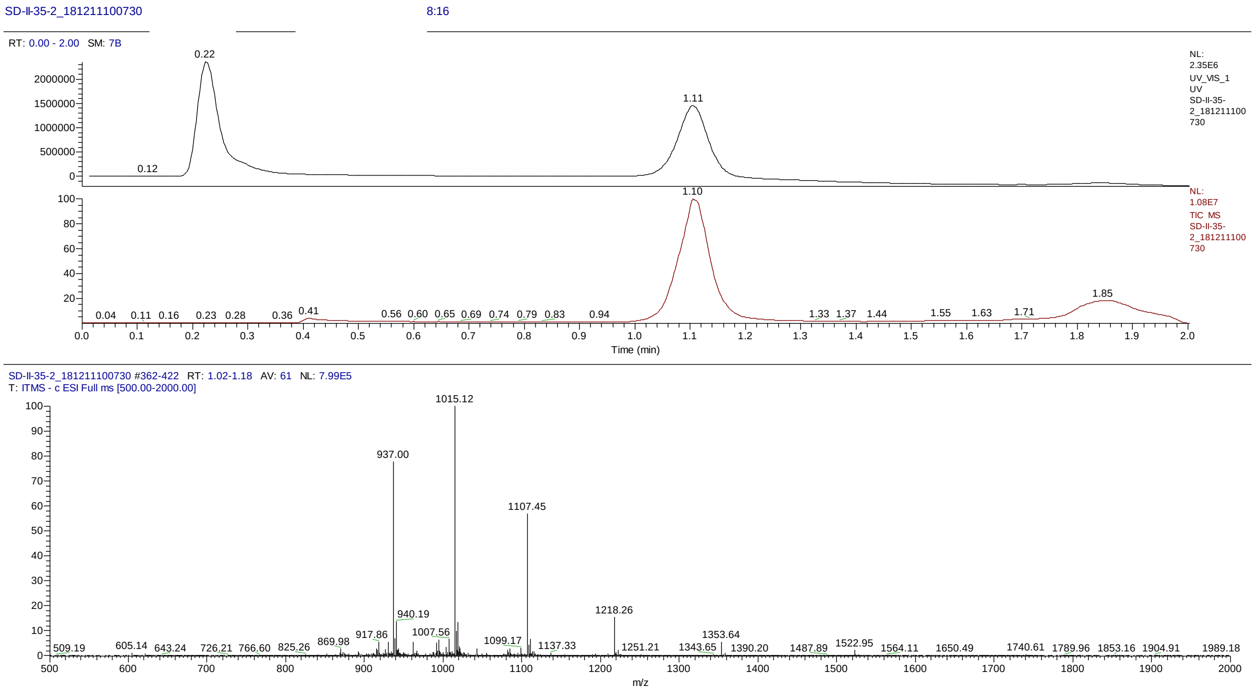

SD-II-35-2_181211100730
8:16
RT: 0.00 - 2.00 SM: 7B
NL: 2.35E6 UV_VIS_1 UV SD-II-35-2_181211100730
NL: 1.08E7 TIC MS SD-II-35-2_181211100730
Time (min)
SD-II-35-2_181211100730 #362-422 RT: 1.02-1.18 AV: 61 NL: 7.99E5
T: ITMS - c ESI Full ms [500.00-2000.00]
1015.12
937.00
1107.45
1218.26
m/z

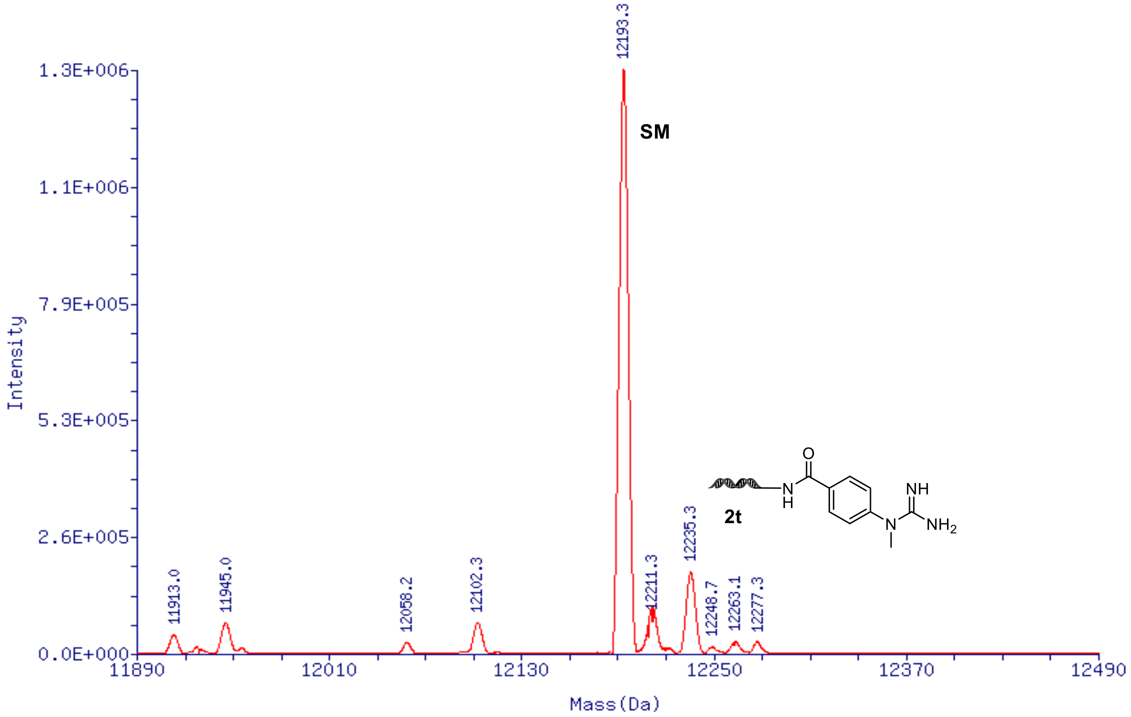

12193.3
SM
2t
Intensity
Mass(Da)
11913.0
11945.0
12058.2
12102.3
12211.3
12235.3
12248.7
12263.1
12277.3

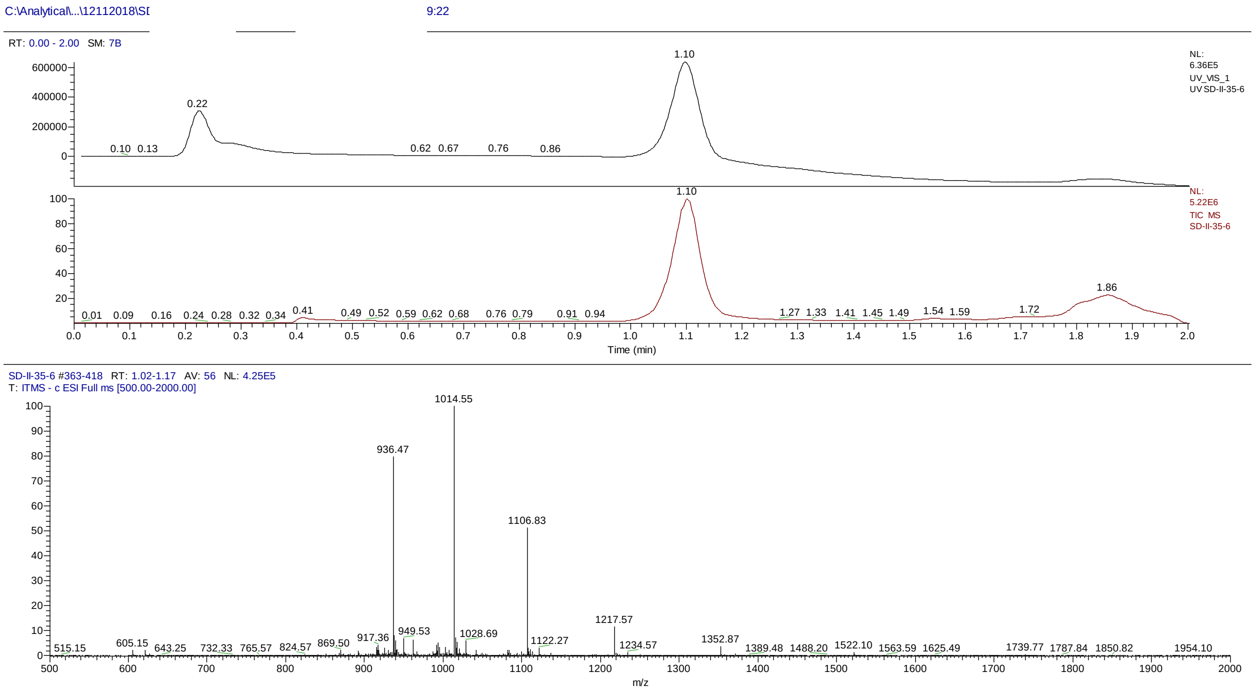
C:\Analytical\...\12112018\SI
9:22
RT: 0.00 - 2.00 SM: 7B
NL: 6.36E5 UV_VIS_1 UV SD-II-35-6
NL: 5.22E6 TIC MS SD-II-35-6
Time (min)
SD-II-35-6 #363-418 RT: 1.02-1.17 AV: 56 NL: 4.25E5
T: ITMS - c ESI Full ms [500.00-2000.00]
1014.55
936.47
1106.83
1217.57
m/z

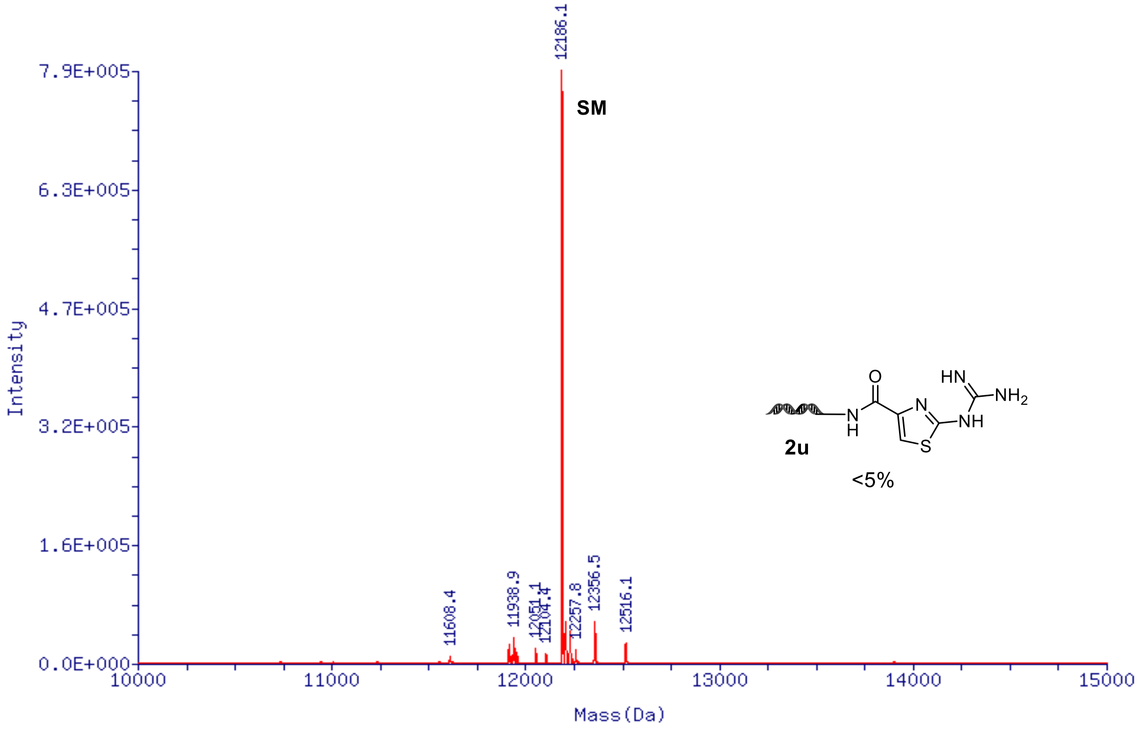
12186.1
SM
Intensity
Mass(Da)
11608.4
11938.9
12051.1
12104.4
12257.8
12356.5
12516.1
2u
<5%

7c) Nitrile reduction

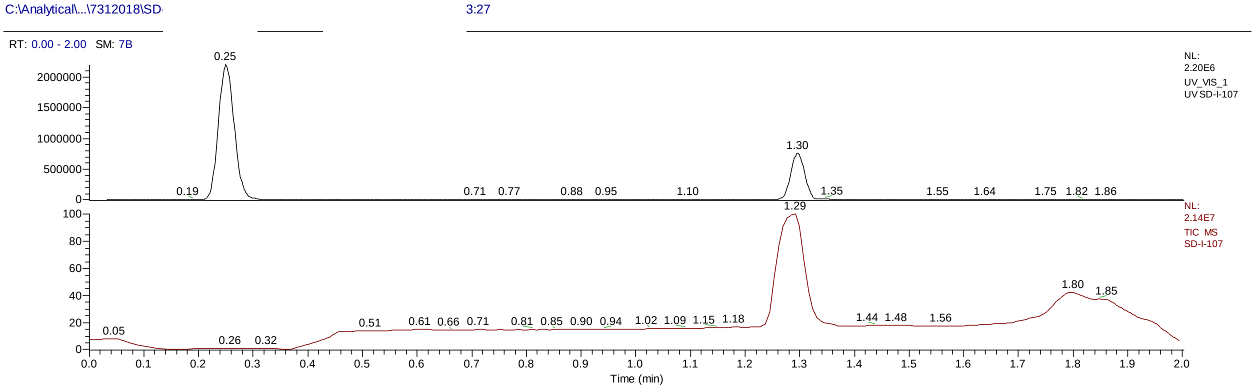

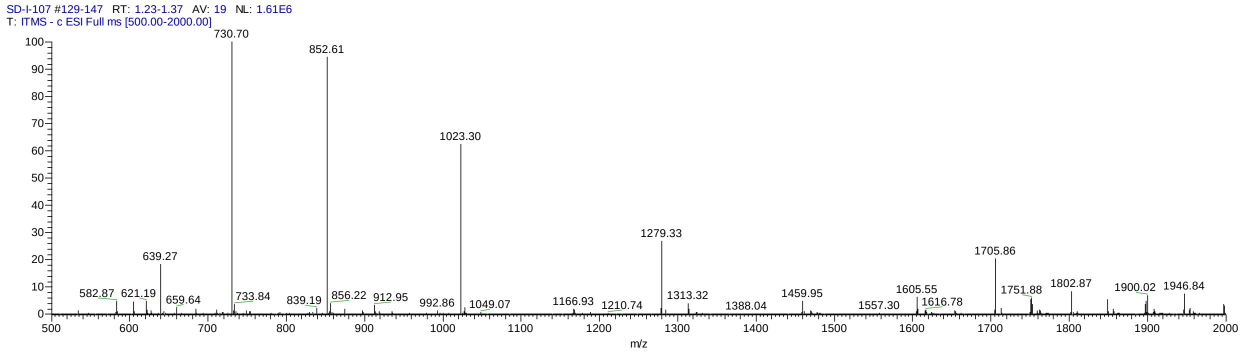

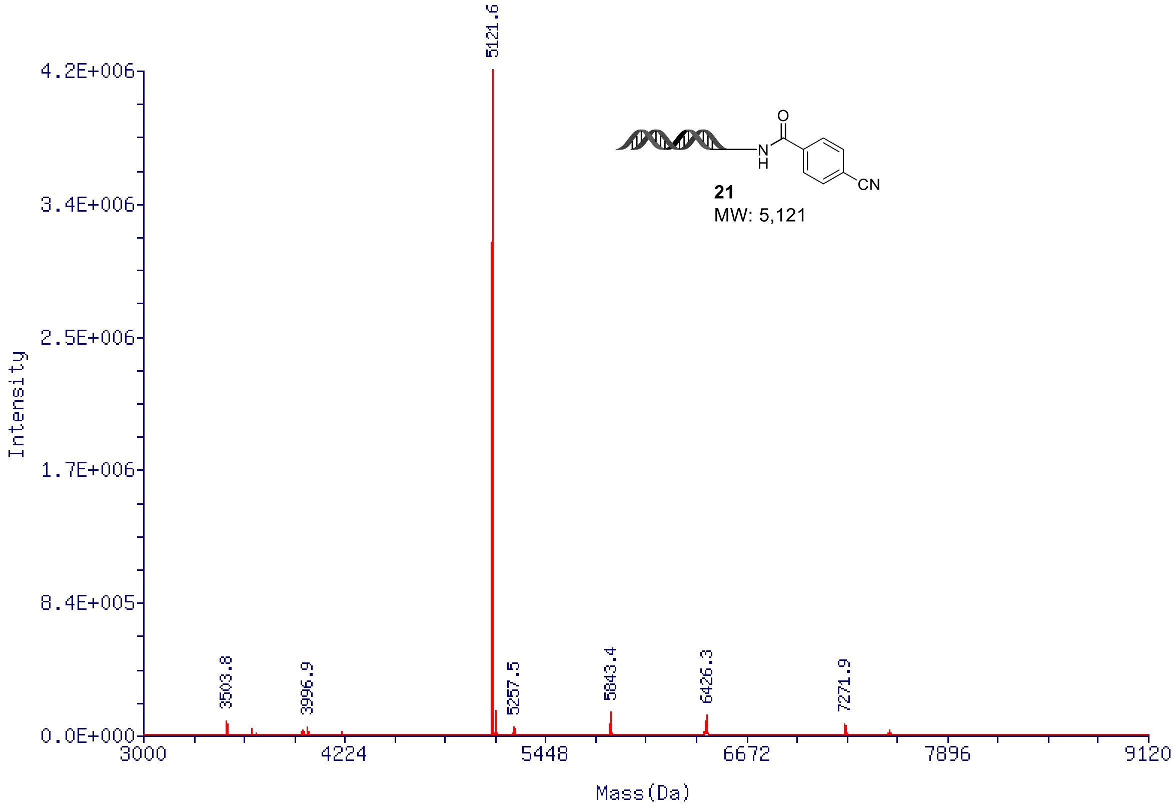

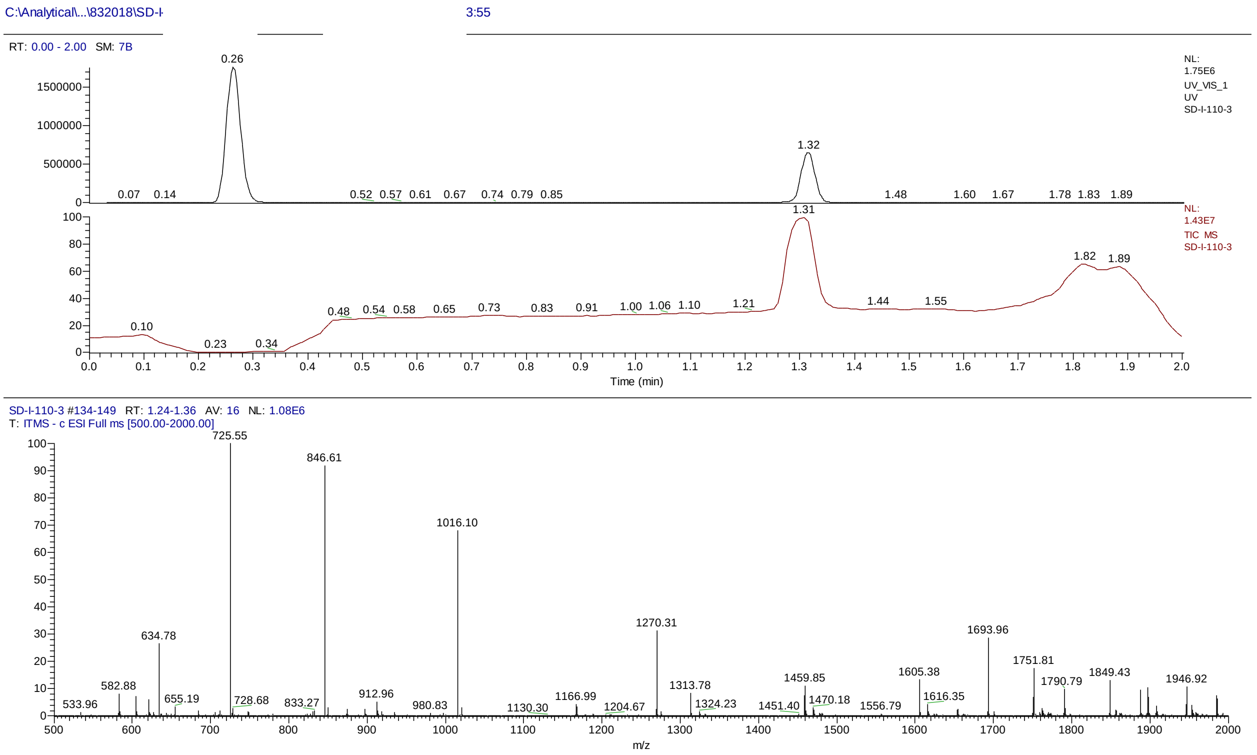

C:\Analytical\...\832018\SD-I
3:55
RT: 0.00 - 2.00 SM: 7B
NL: 1.75E6 UV_VIS_1 UV SD-I-110-3
NL: 1.43E7 TIC MS SD-I-110-3
Time (min)
SD-I-110-3 #134-149 RT: 1.24-1.36 AV: 16 NL: 1.08E6
T: ITMS - c ESI Full ms [500.00-2000.00]
725.55
846.61
1016.10
1270.31
1693.96
m/z

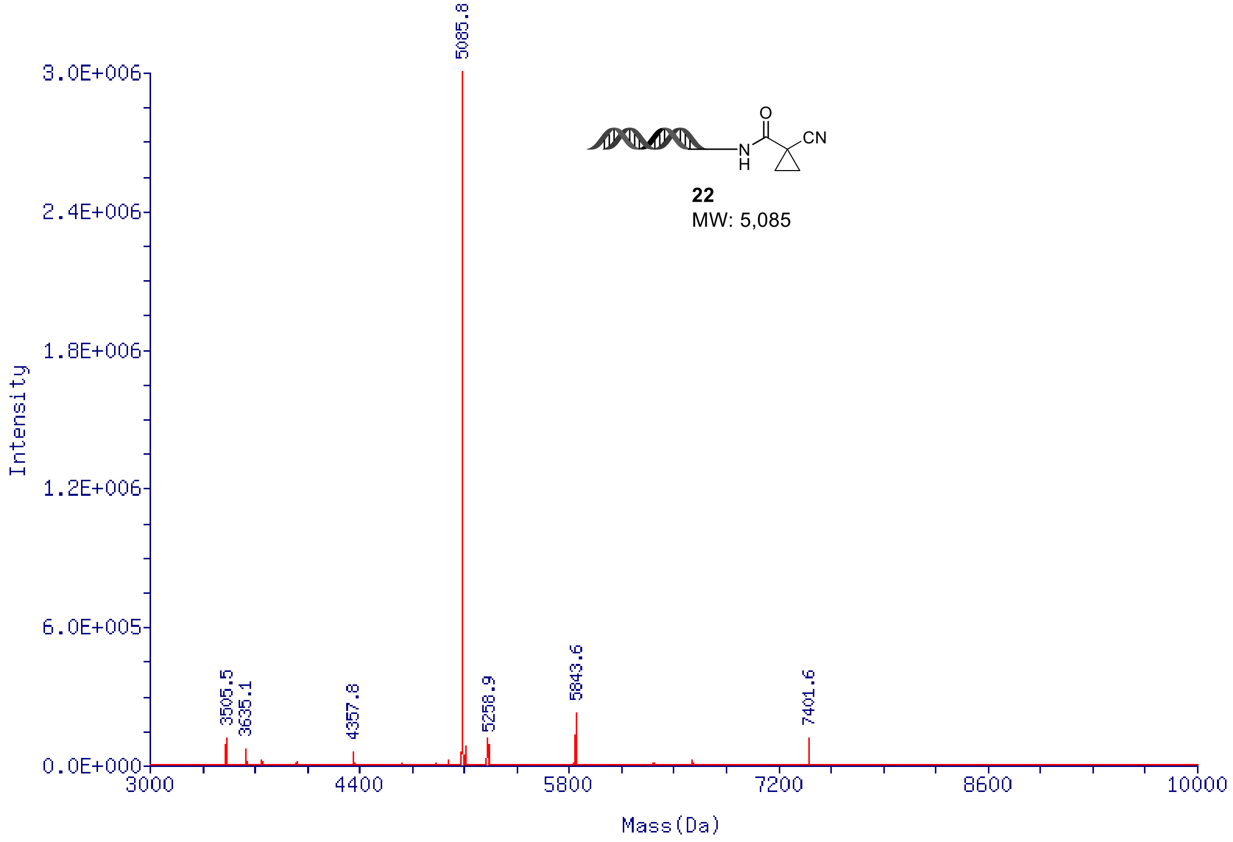

5085.8
3505.5
3635.1
4357.8
5258.9
5843.6
7401.6
Intensity
Mass(Da)
22
MW: 5,085

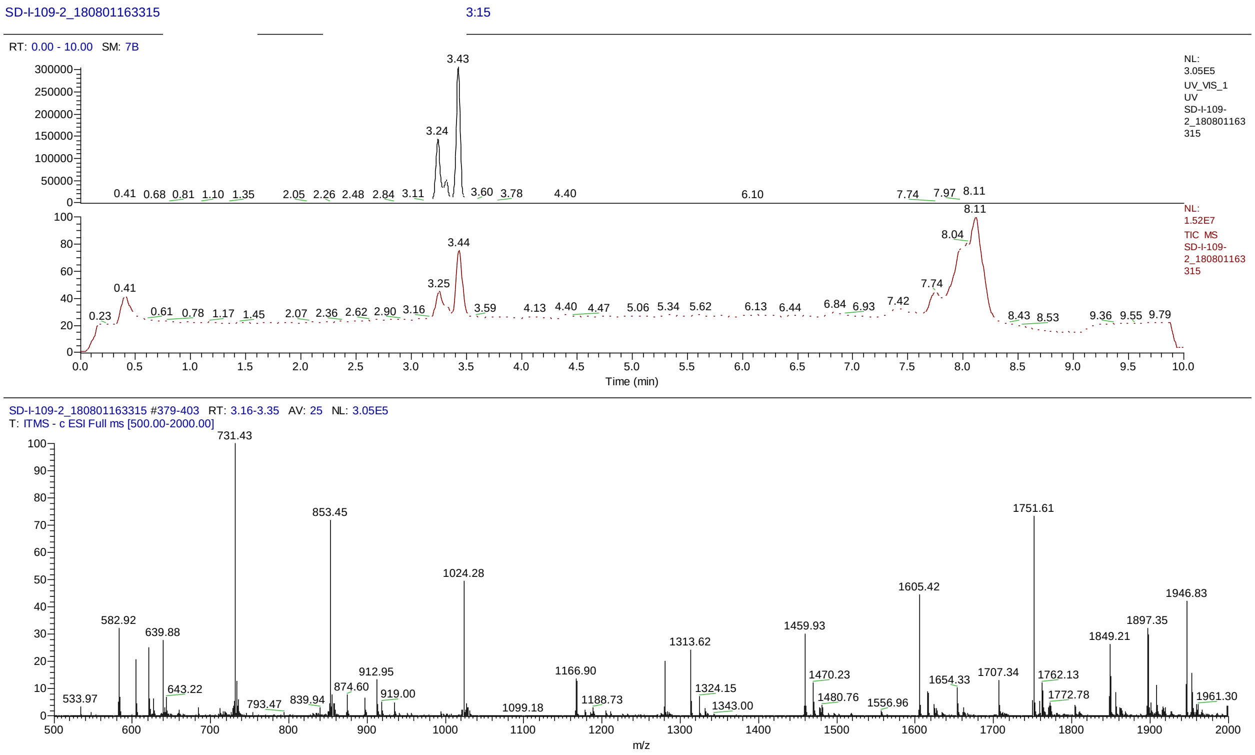
SD-I-109-2_180801163315
3:15
RT: 0.00 - 10.00 SM: 7B
NL: 3.05E5 UV_VIS_1 UV SD-I-109-2_180801163315
NL: 1.52E7 TIC MS SD-I-109-2_180801163315
Time (min)
SD-I-109-2_180801163315 #379-403 RT: 3.16-3.35 AV: 25 NL: 3.05E5
T: ITMS - c ESI Full ms [500.00-2000.00]
m/z

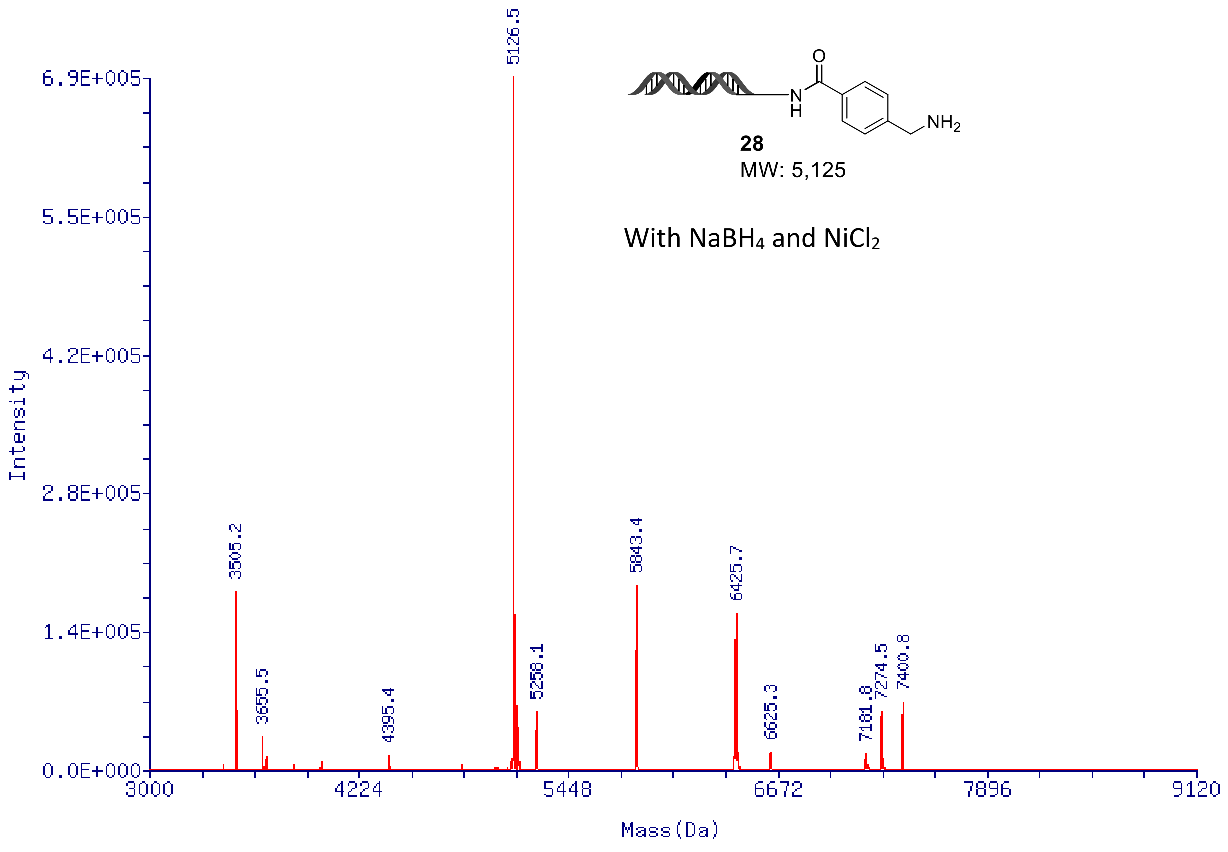
5126.5
3505.2
3655.5
4395.4
5258.1
5843.4
6425.7
6625.3
7181.8
7274.5
7400.8
Intensity
Mass(Da)
28
MW: 5,125
With NaBH4 and NiCl2

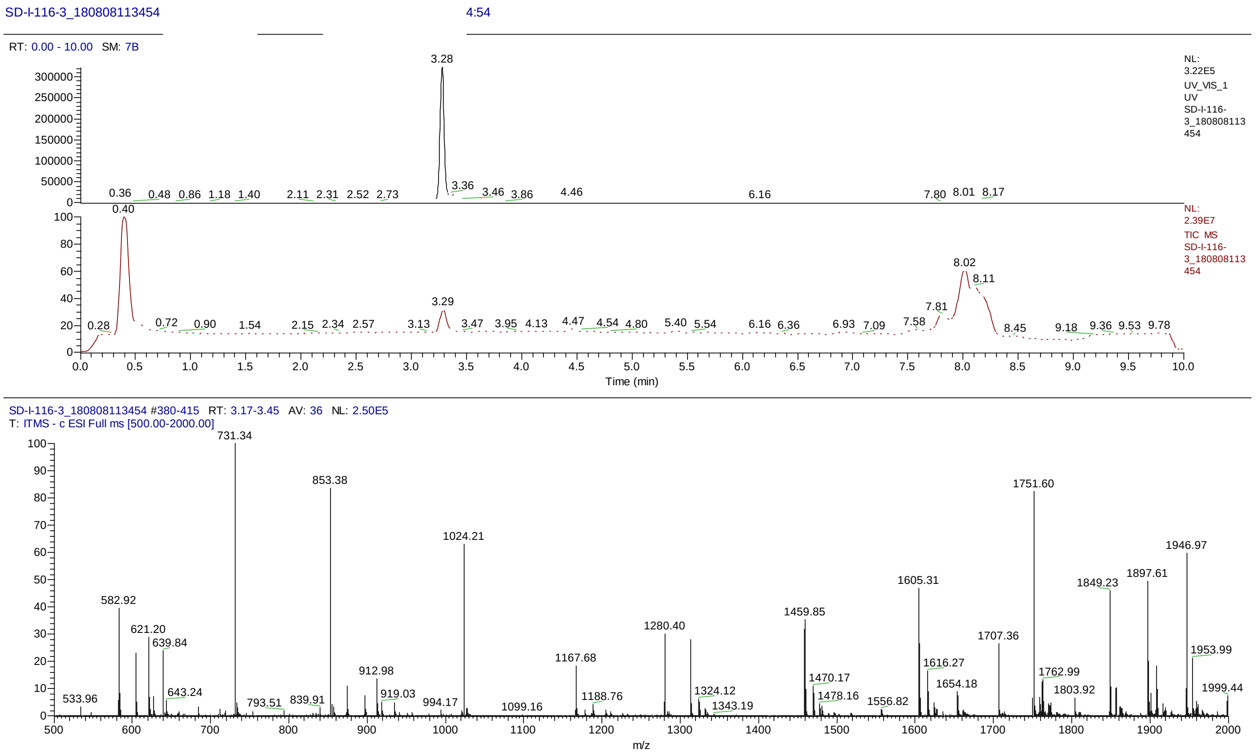
SD-I-116-3_180808113454
4:54
RT: 0.00 - 10.00 SM: 7B
NL: 3.22E5 UV_VIS_1 UV SD-I-116-3_180808113454
3.28
0.36 0.48 0.86 1.18 1.40 2.11 2.31 2.52 2.73 3.36 3.46 3.86 4.46 6.16 7.80 8.01 8.17
NL: 2.39E7 TIC MS SD-I-116-3_180808113454
0.40
0.28 0.72 0.90 1.54 2.15 2.34 2.57 3.13 3.29 3.47 3.95 4.13 4.47 4.54 4.80 5.40 5.54 6.16 6.36 6.93 7.09 7.58 7.81 8.02 8.11 8.45 9.18 9.36 9.53 9.78
Time (min)
SD-I-116-3_180808113454 #380-415 RT: 3.17-3.45 AV: 36 NL: 2.50E5
T: ITMS - c ESI Full ms [500.00-2000.00]
731.34
853.38
1024.21
1751.60
1946.97
1605.31
1849.23
1897.61
582.92
621.20
639.84
1459.85
1280.40
1707.36
1167.68
1953.99
1616.27
1762.99
912.98
1470.17
1654.18
1803.92
533.96
643.24
919.03
1324.12
1478.16
1999.44
793.51
839.91
994.17
1099.16
1188.76
1343.19
1556.82
m/z

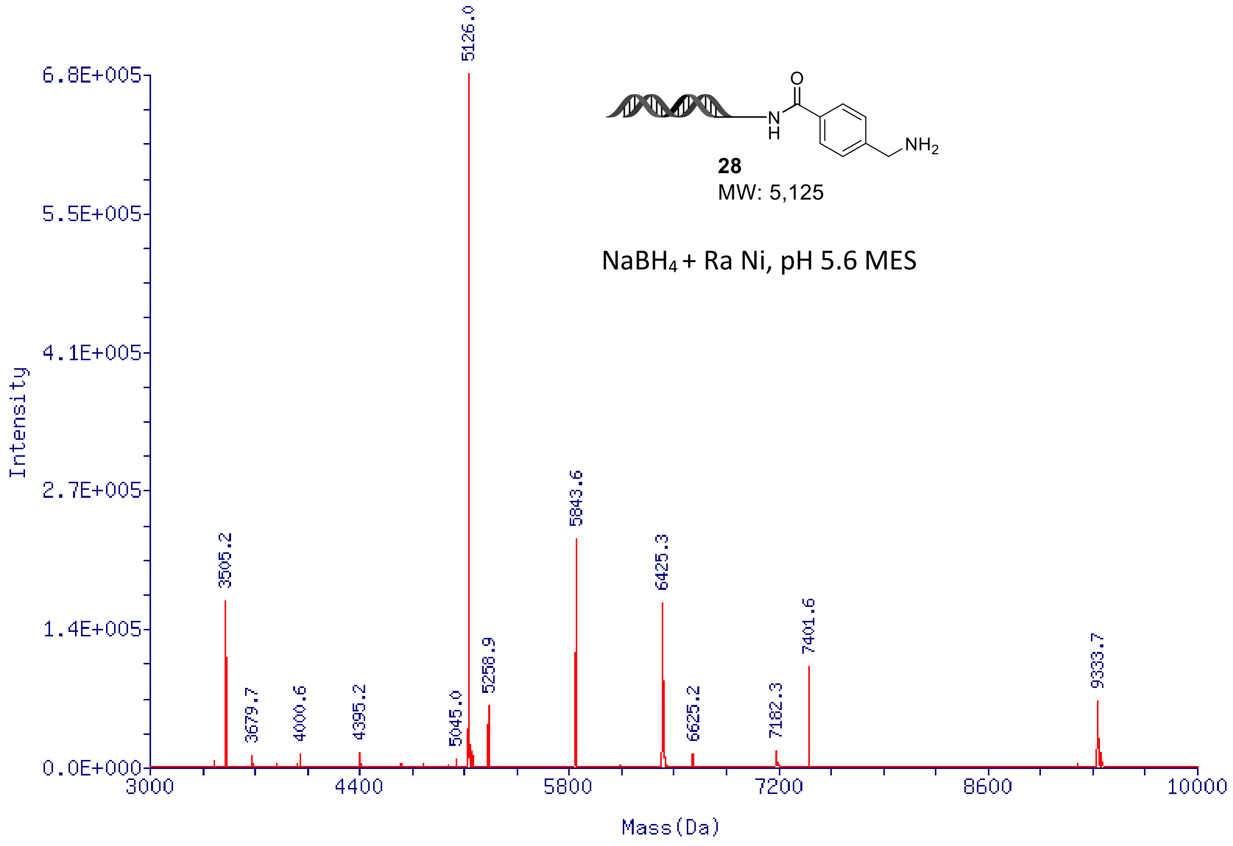
5126.0
28
MW: 5,125
NaBH4 + Ra Ni, pH 5.6 MES
Intensity
5843.6
3505.2
6425.3
7401.6
9333.7
5258.9
3679.7
4000.6
4395.2
5045.0
6625.2
7182.3
Mass(Da)

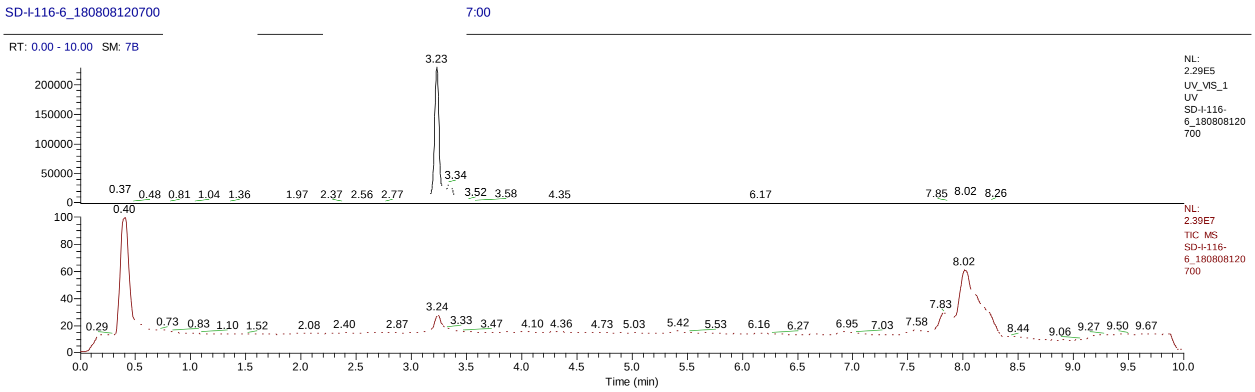
SD-I-116-6_180808120700
7:00
RT: 0.00 - 10.00 SM: 7B
NL: 2.29E5 UV_VIS_1 UV SD-I-116-6_180808120700
NL: 2.39E7 TIC MS SD-I-116-6_180808120700
3.23
0.40
8.02
Time (min)

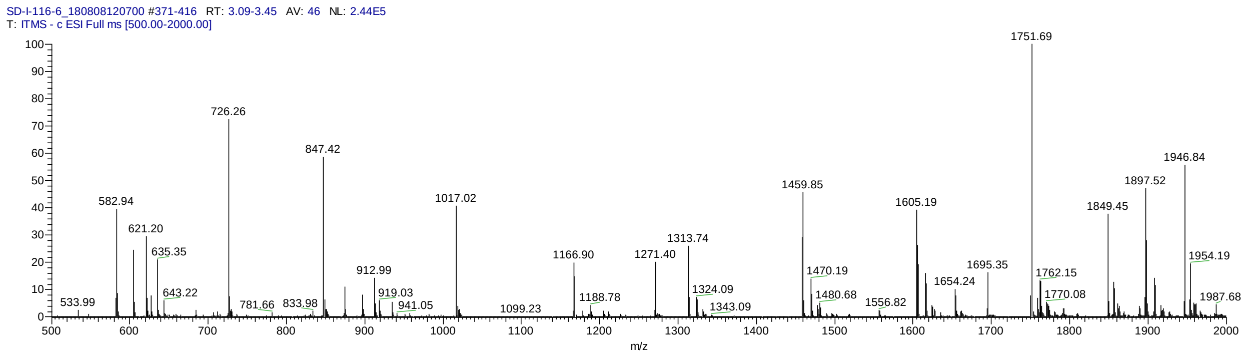
SD-I-116-6_180808120700 #371-416 RT: 3.09-3.45 AV: 46 NL: 2.44E5
T: ITMS - c ESI Full ms [500.00-2000.00]
1751.69
726.26
847.42
1017.02
1459.85
1605.19
1946.84
1897.52
1849.45
m/z

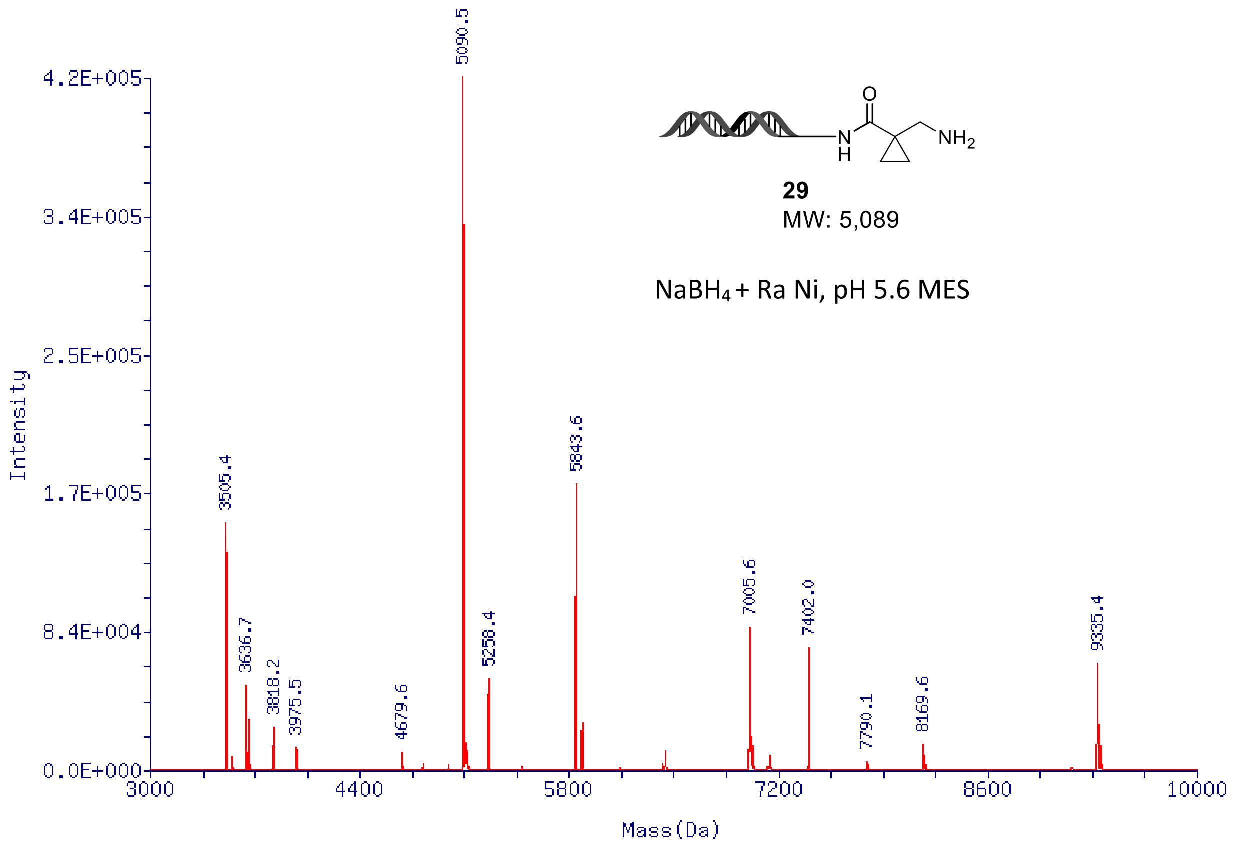
29
MW: 5,089
NaBH$_4$ + Ra Ni, pH 5.6 MES
Intensity
Mass(Da)
5090.5
5843.6
3505.4
7005.6
7402.0
9335.4

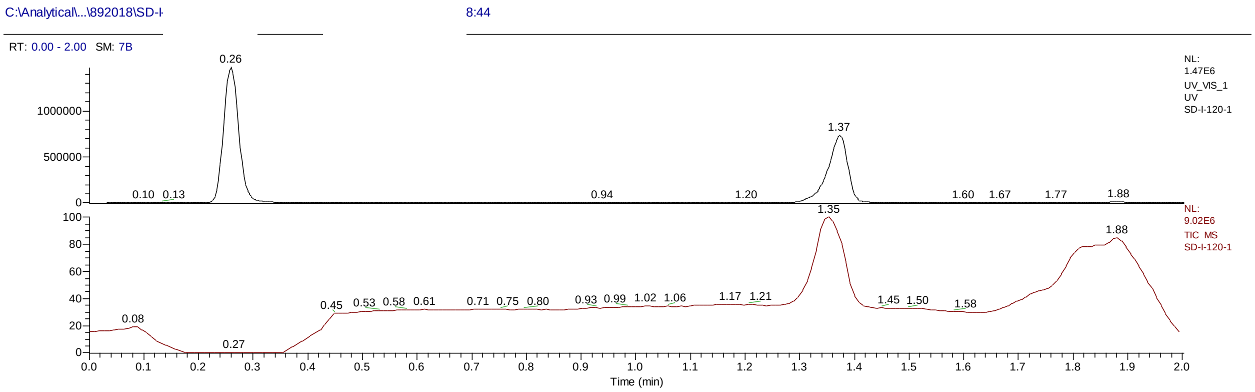
C:\Analytical\...\892018\SD-I
8:44
RT: 0.00 - 2.00 SM: 7B
NL: 1.47E6 UV_VIS_1 UV SD-I-120-1
0.26
1.37
0.10 0.13
0.94
1.20
1.60 1.67 1.77 1.88
NL: 9.02E6 TIC MS SD-I-120-1
1.35
1.88
0.08
0.27
0.45 0.53 0.58 0.61 0.71 0.75 0.80 0.93 0.99 1.02 1.06 1.17 1.21
1.45 1.50 1.58
Time (min)

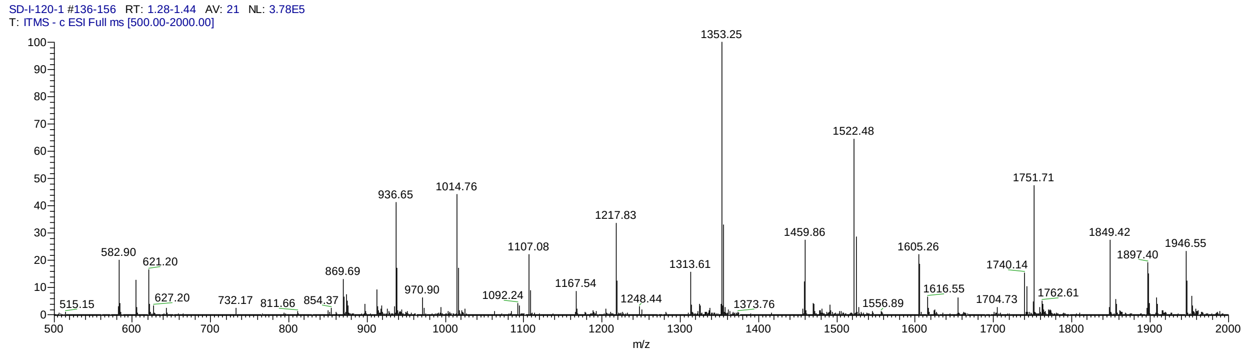
SD-I-120-1 #136-156 RT: 1.28-1.44 AV: 21 NL: 3.78E5
T: ITMS - c ESI Full ms [500.00-2000.00]
1353.25
1522.48
1751.71
936.65
1014.76
1217.83
1107.08
1459.86
1849.42
1946.55
1897.40
1605.26
582.90
621.20
869.69
1313.61
1740.14
1167.54
970.90
1616.55
1762.61
1092.24
1248.44
1704.73
515.15
627.20
732.17
811.66
854.37
1373.76
1556.89
m/z

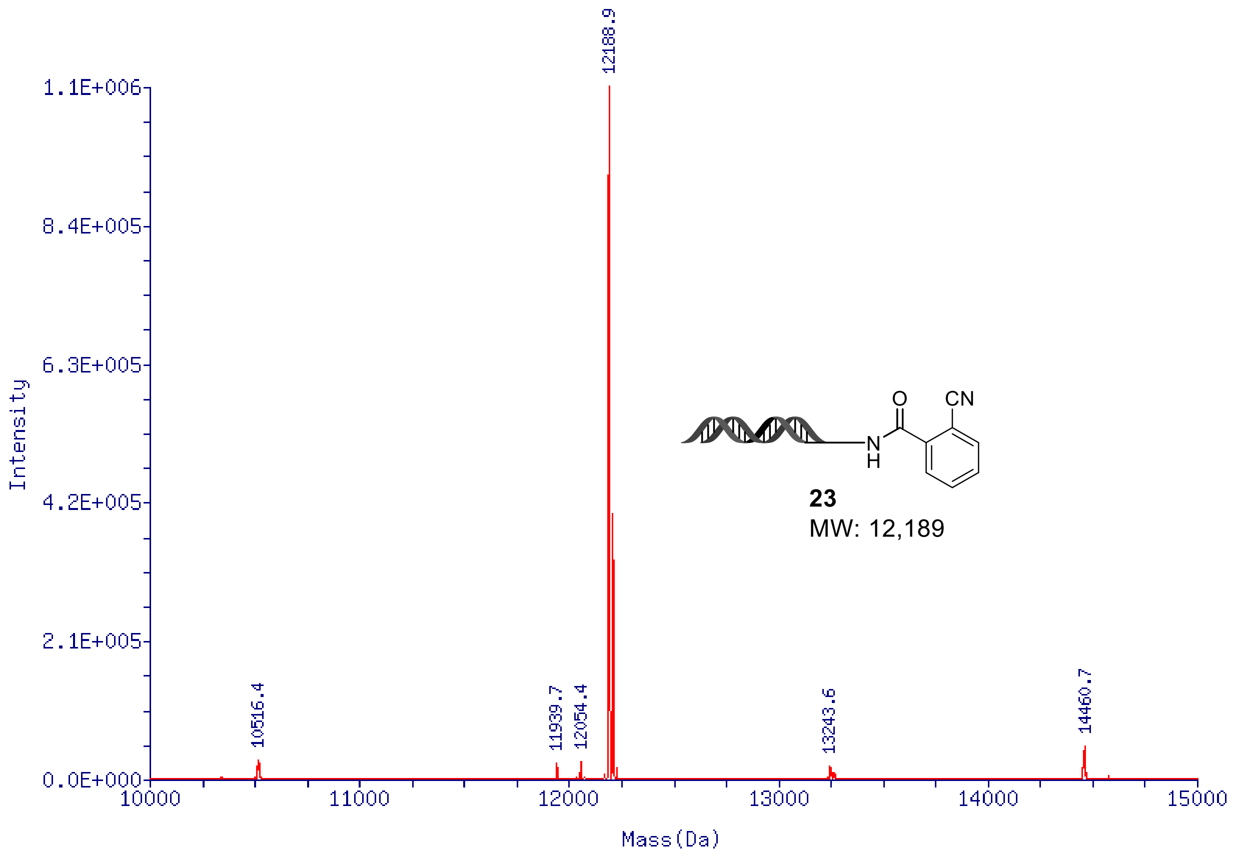
12188.9
Intensity
1.1E+006
8.4E+005
6.3E+005
4.2E+005
2.1E+005
0.0E+000
10516.4
11939.7
12054.4
13243.6
14460.7
Mass(Da)
O
CN
N
H
23
MW: 12,189

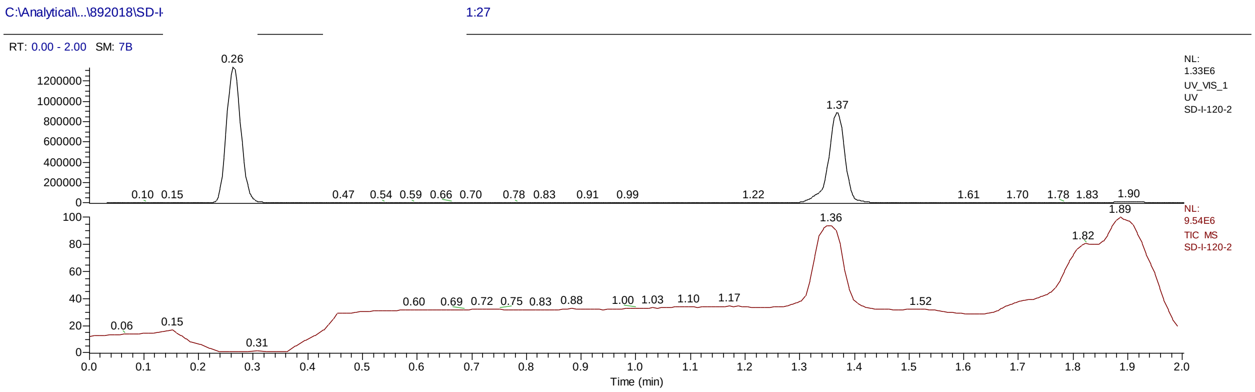
C:\Analytical\...\892018\SD-I
1:27
RT: 0.00 - 2.00 SM: 7B
NL: 1.33E6 UV_VIS_1 UV SD-I-120-2
0.26
1.37
NL: 9.54E6 TIC MS SD-I-120-2
1.36
1.89
1.82
Time (min)

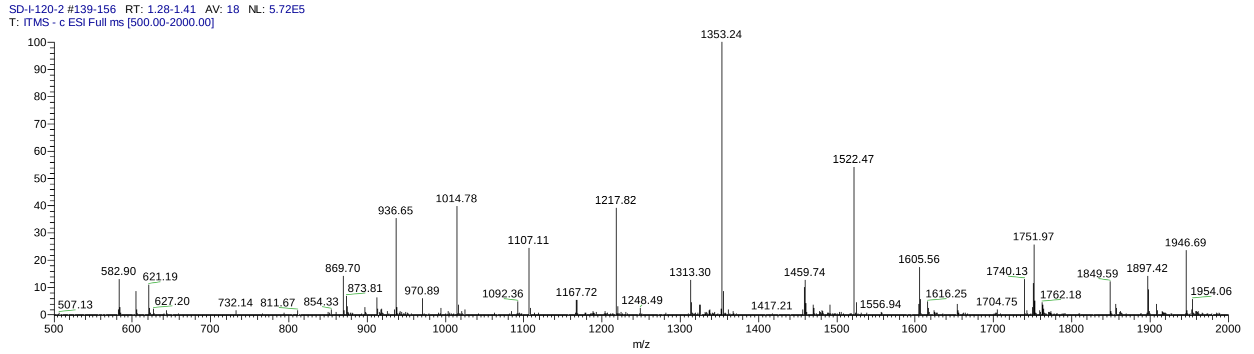
SD-I-120-2 #139-156 RT: 1.28-1.41 AV: 18 NL: 5.72E5
T: ITMS - c ESI Full ms [500.00-2000.00]
1353.24
1522.47
1014.78
1217.82
936.65
1107.11
1751.97
1946.69
m/z

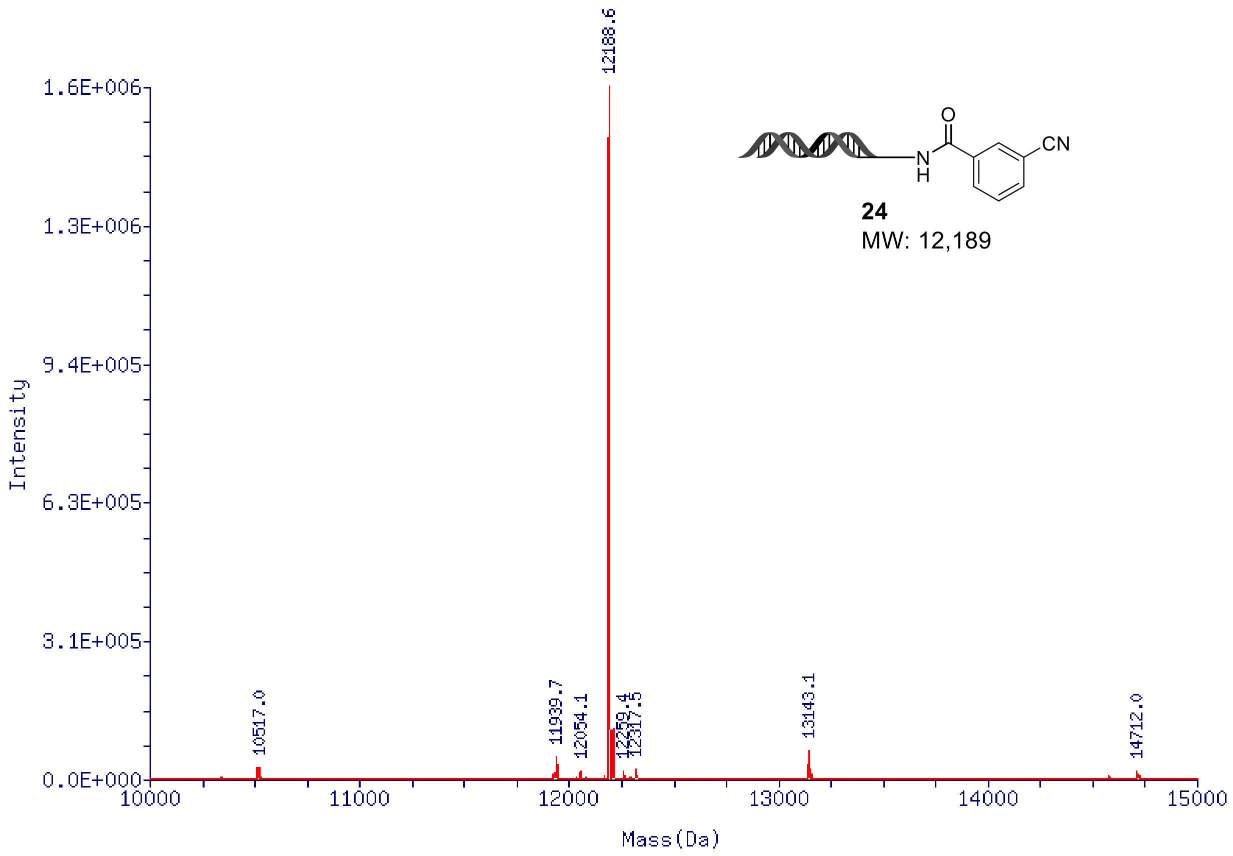
12188.6
10517.0
11939.7
12054.1
12259.4
12317.5
13143.1
14712.0
Intensity
Mass(Da)
CN
24
MW: 12,189

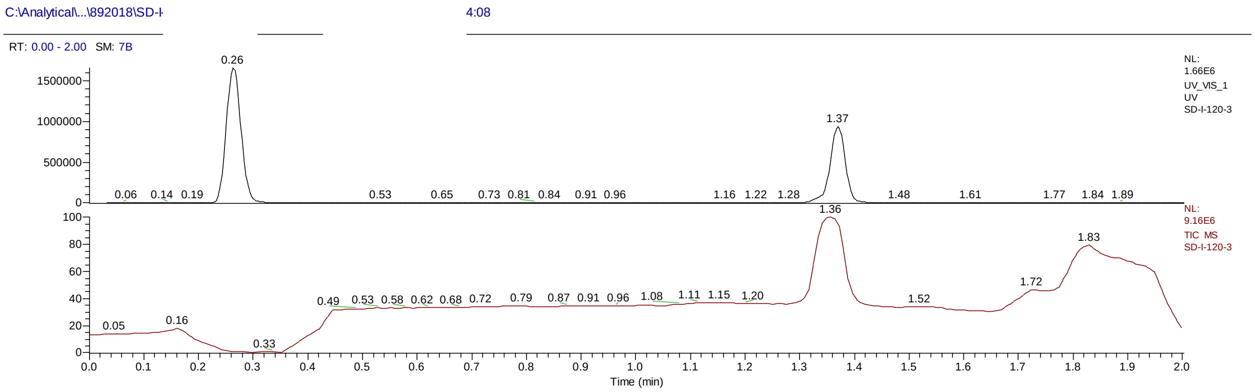
C:\Analytical\...\892018\SD-I
4:08
RT: 0.00 - 2.00 SM: 7B
NL: 1.66E6
UV_VIS_1
UV
SD-I-120-3
NL: 9.16E6
TIC MS
SD-I-120-3
Time (min)

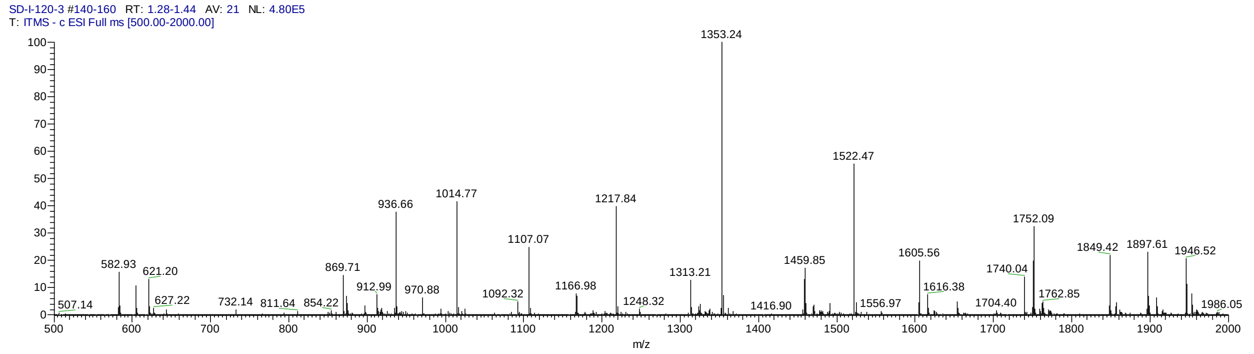
SD-I-120-3 #140-160 RT: 1.28-1.44 AV: 21 NL: 4.80E5
T: ITMS - c ESI Full ms [500.00-2000.00]
1353.24
1522.47
1752.09
1014.77
936.66
1217.84
1107.07
m/z

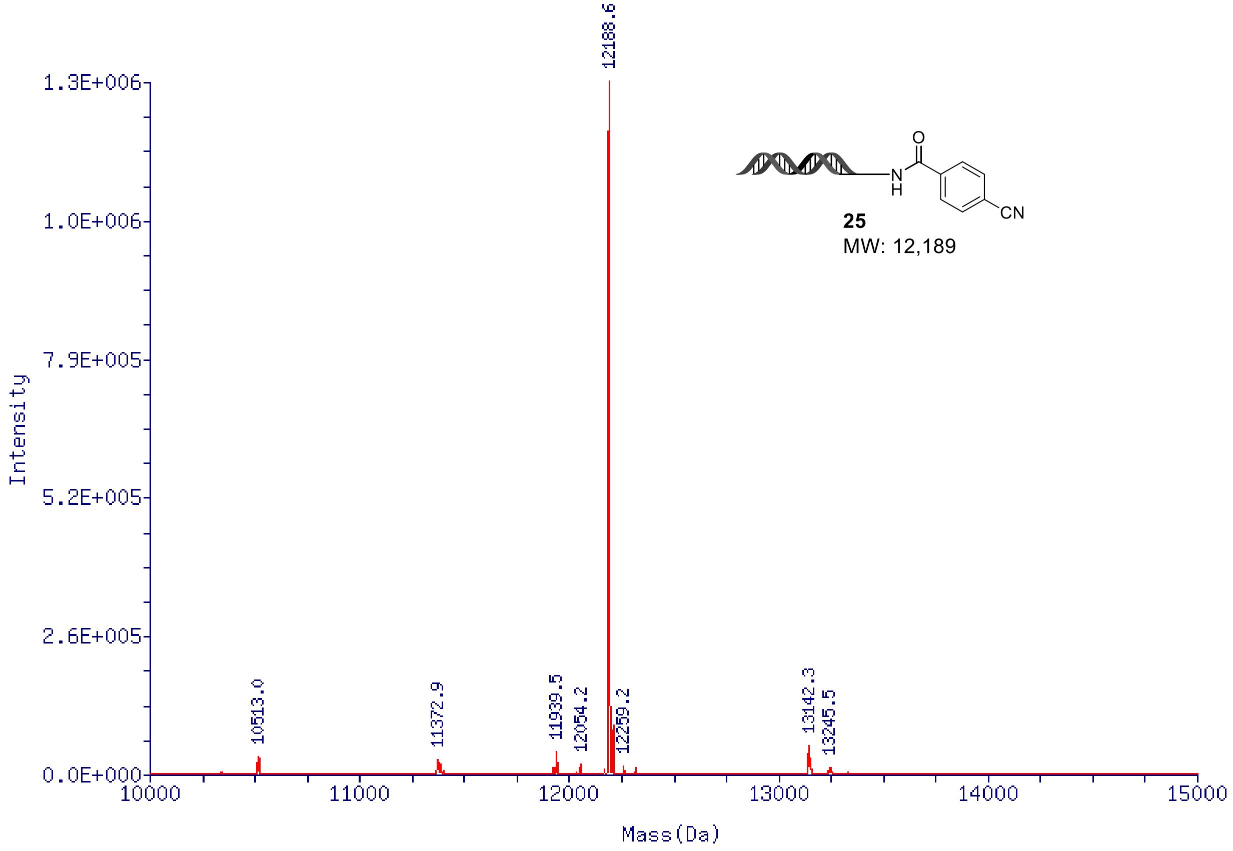
12188.6
10513.0
11372.9
11939.5
12054.2
12259.2
13142.3
13245.5
Intensity
Mass(Da)
25
MW: 12,189

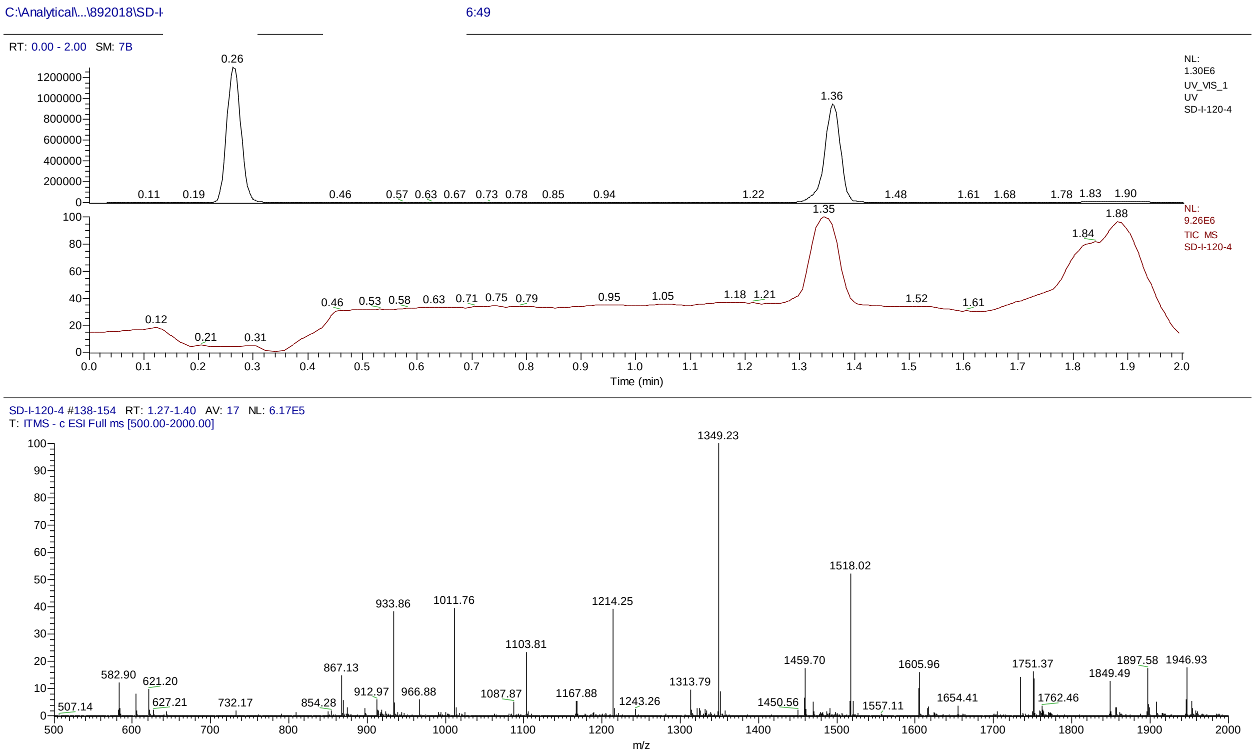
C:\Analytical\...\892018\SD-I
6:49
RT: 0.00 - 2.00 SM: 7B
NL: 1.30E6 UV_VIS_1 UV SD-I-120-4
NL: 9.26E6 TIC MS SD-I-120-4
Time (min)
SD-I-120-4 #138-154 RT: 1.27-1.40 AV: 17 NL: 6.17E5
T: ITMS - c ESI Full ms [500.00-2000.00]
1349.23
1518.02
1214.25
1011.76
933.86
1103.81
1946.93
1897.58
1849.49
1751.37
1605.96
1459.70
m/z

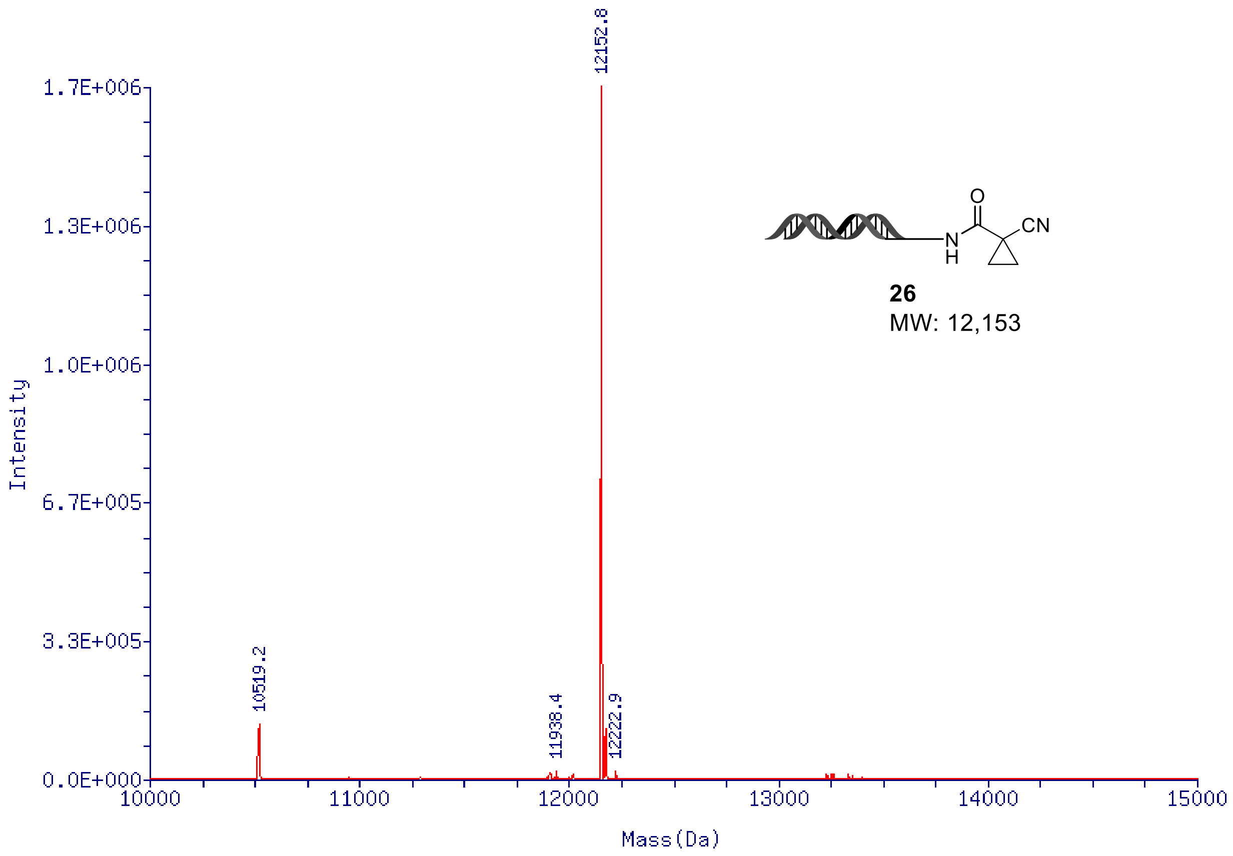
12152.8
10519.2
11938.4
12222.9
Intensity
Mass(Da)
O
CN
N
H

26
MW: 12,153

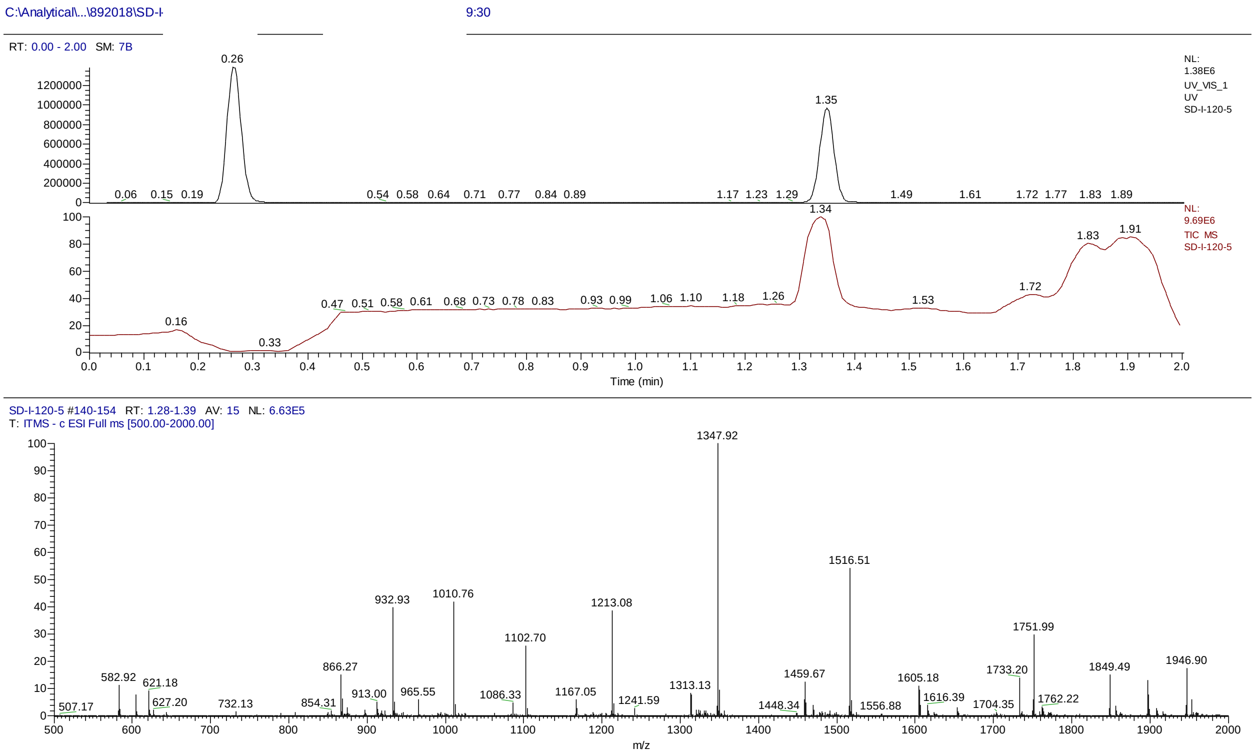
C:\Analytical\...\892018\SD-I
9:30
RT: 0.00 - 2.00 SM: 7B
NL: 1.38E6 UV_VIS_1 UV SD-I-120-5
NL: 9.69E6 TIC MS SD-I-120-5
Time (min)
SD-I-120-5 #140-154 RT: 1.28-1.39 AV: 15 NL: 6.63E5
T: ITMS - c ESI Full ms [500.00-2000.00]
1347.92
1516.51
1010.76
932.93
1213.08
1102.70
1751.99
1946.90
m/z

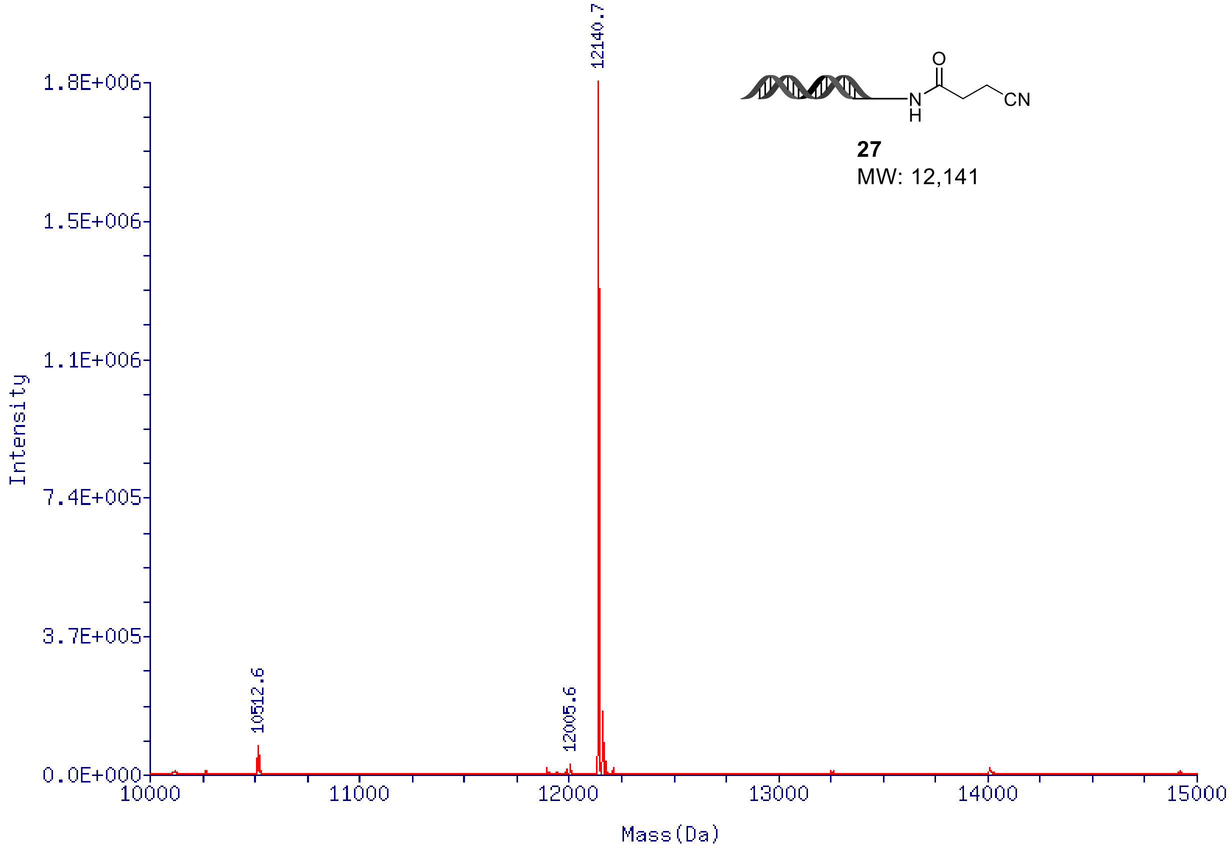
12140.7
10512.6
12005.6
Intensity
Mass(Da)

27
MW: 12,141

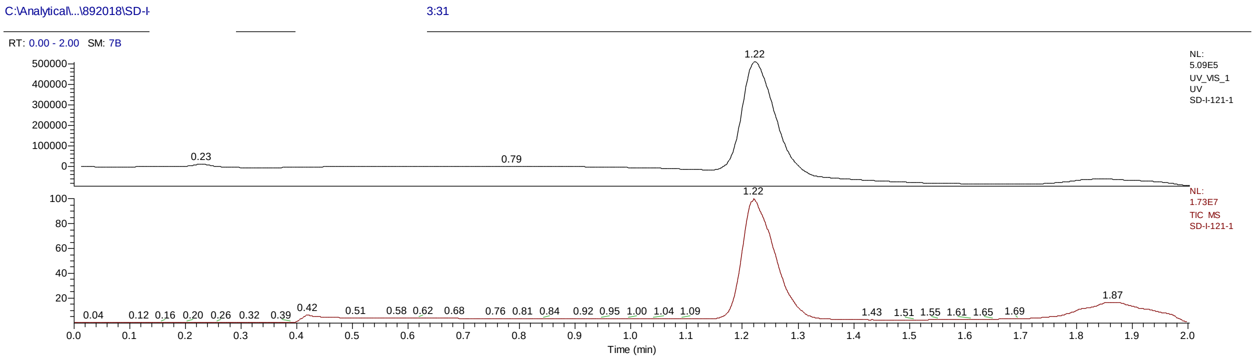
C:\Analytical\...\892018\SD-I
3:31
RT: 0.00 - 2.00 SM: 7B
NL: 5.09E5 UV_VIS_1 UV SD-I-121-1
NL: 1.73E7 TIC MS SD-I-121-1
Time (min)

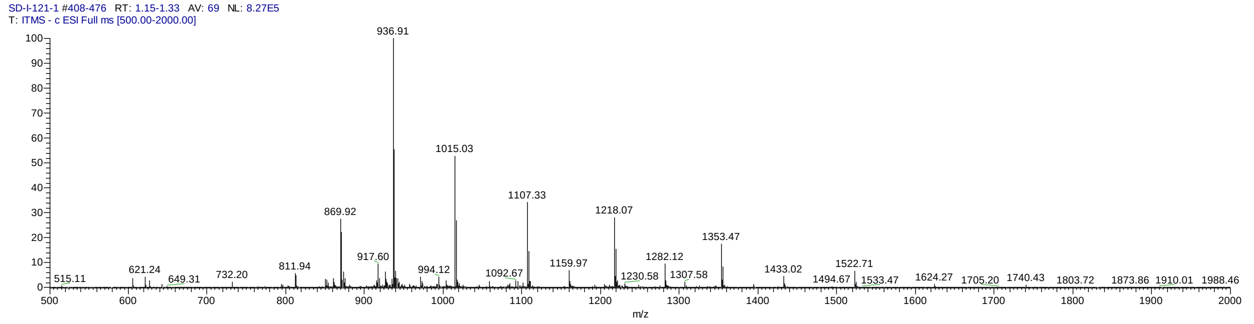
SD-I-121-1 #408-476 RT: 1.15-1.33 AV: 69 NL: 8.27E5
T: ITMS - c ESI Full ms [500.00-2000.00]
936.91
1015.03
1107.33
1218.07
869.92
1353.47
m/z

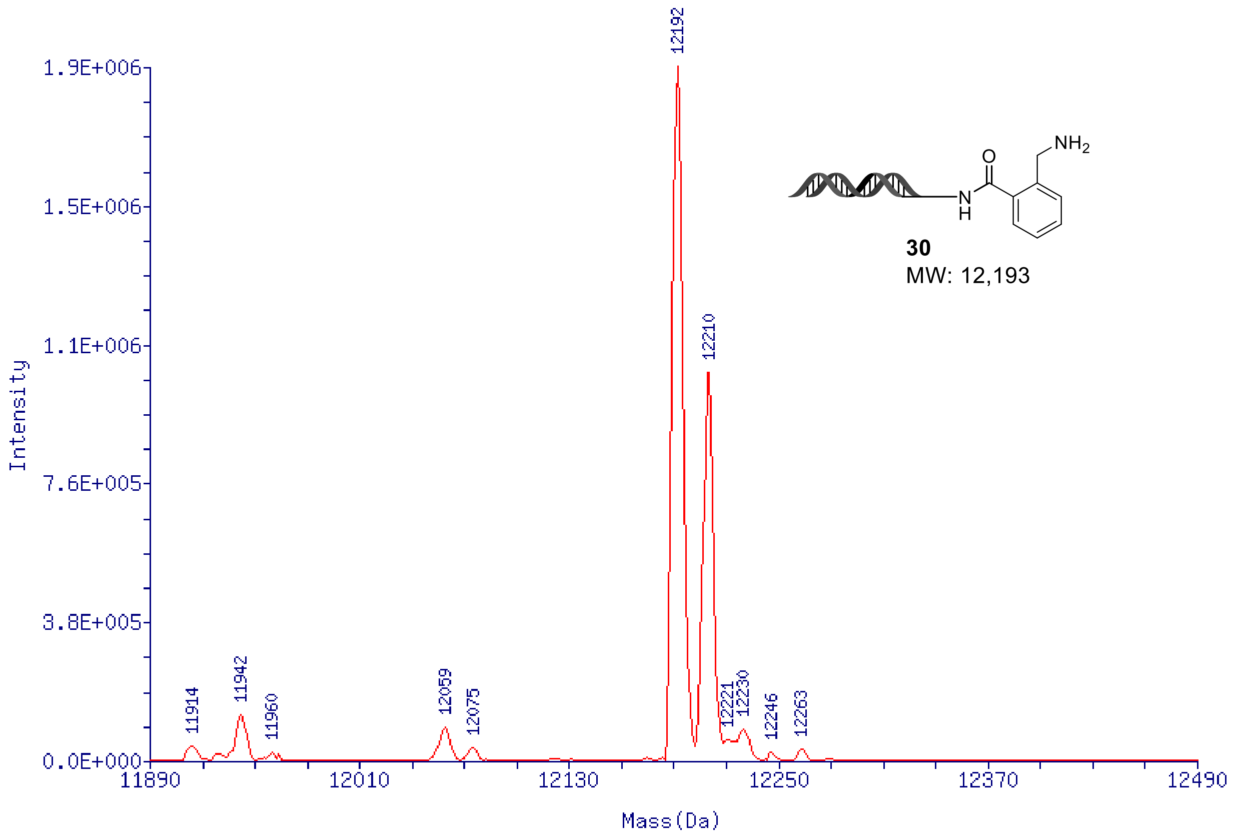
12192
12210
Intensity
Mass(Da)
30
MW: 12,193

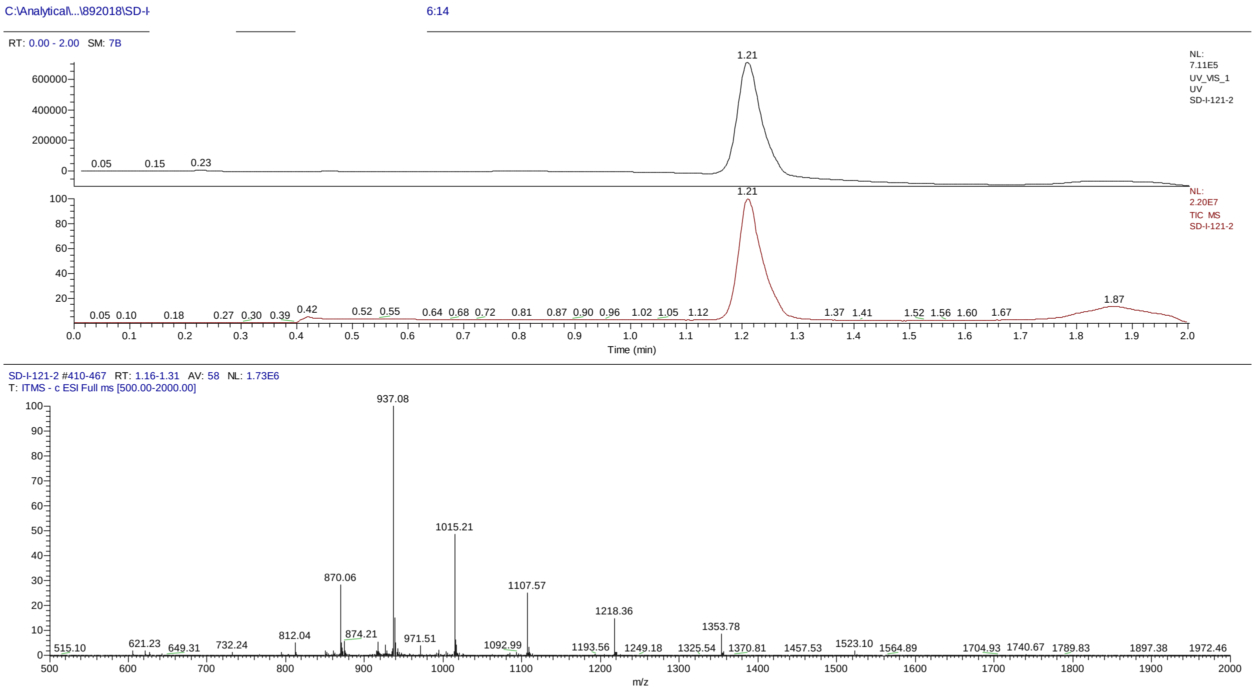
C:\Analytical\...\892018\SD-I
6:14
RT: 0.00 - 2.00 SM: 7B
NL: 7.11E5 UV_VIS_1 UV SD-I-121-2
NL: 2.20E7 TIC MS SD-I-121-2
1.21
Time (min)
SD-I-121-2 #410-467 RT: 1.16-1.31 AV: 58 NL: 1.73E6
T: ITMS - c ESI Full ms [500.00-2000.00]
937.08
1015.21
870.06
1107.57
1218.36
1353.78
m/z

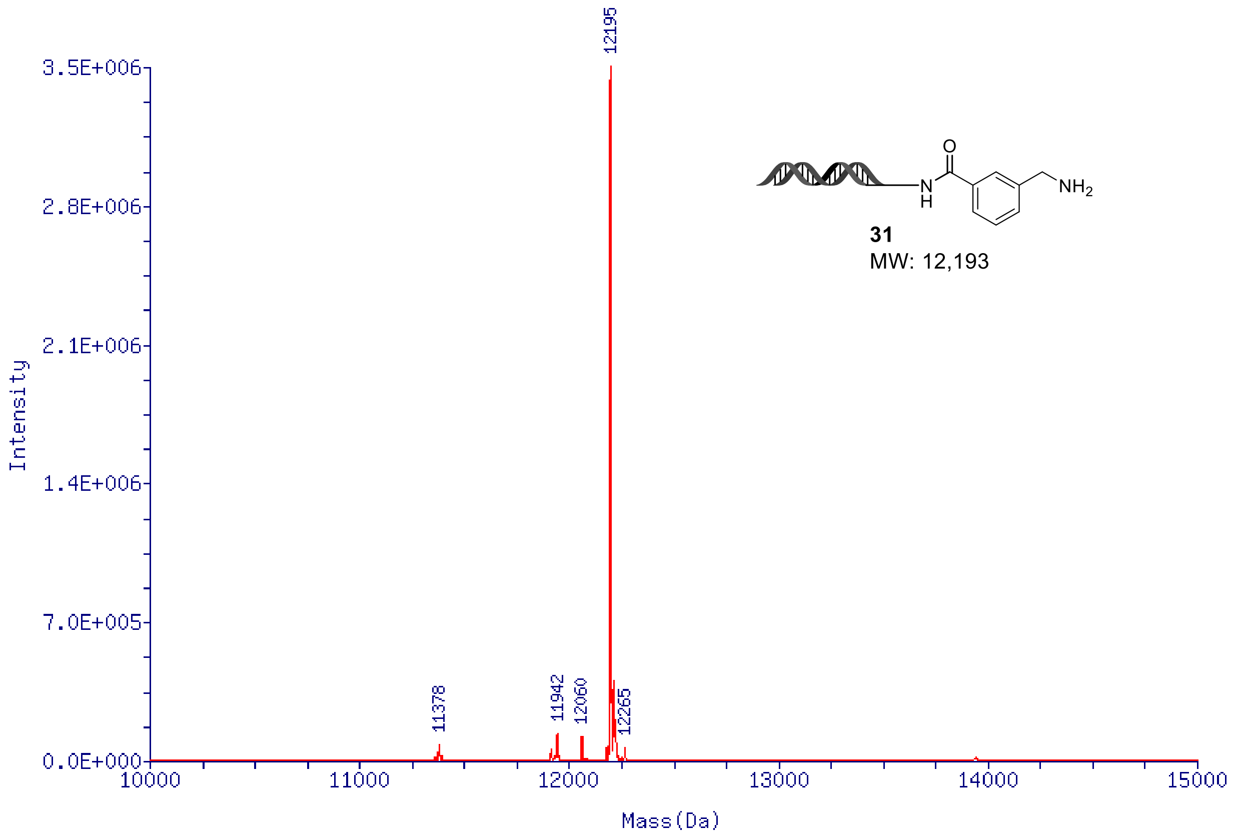
12195
11378
11942
12060
12265
Intensity
Mass(Da)
31
MW: 12,193

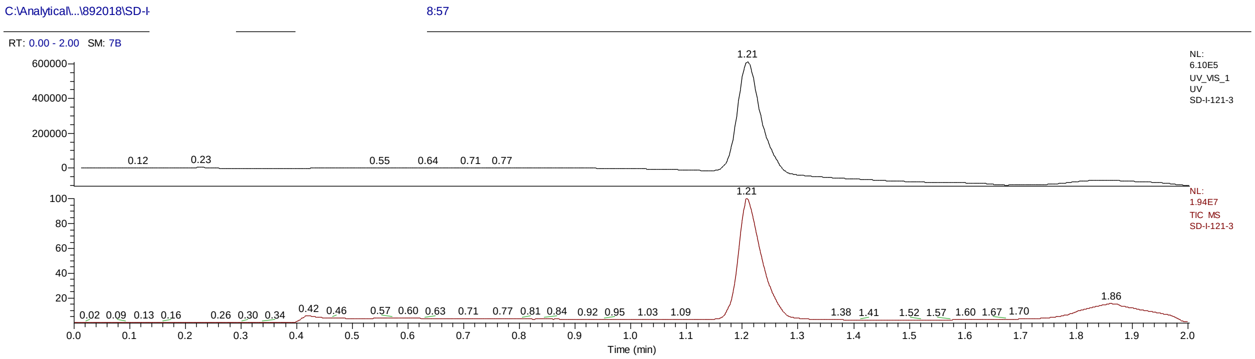

C:\Analytical\...\892018\SD-I
8:57
RT: 0.00 - 2.00 SM: 7B
NL: 6.10E5 UV_VIS_1 UV SD-I-121-3
NL: 1.94E7 TIC MS SD-I-121-3
Time (min)

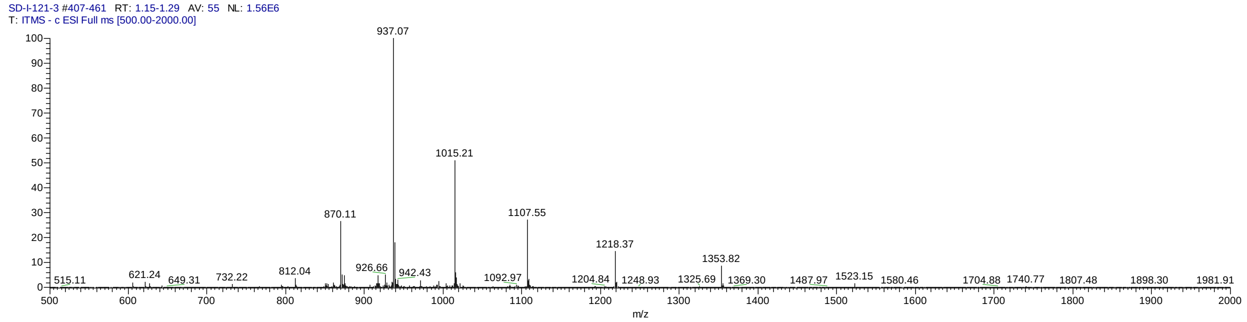

SD-I-121-3 #407-461 RT: 1.15-1.29 AV: 55 NL: 1.56E6
T: ITMS - c ESI Full ms [500.00-2000.00]
937.07
1015.21
1107.55
870.11
1218.37
1353.82
m/z

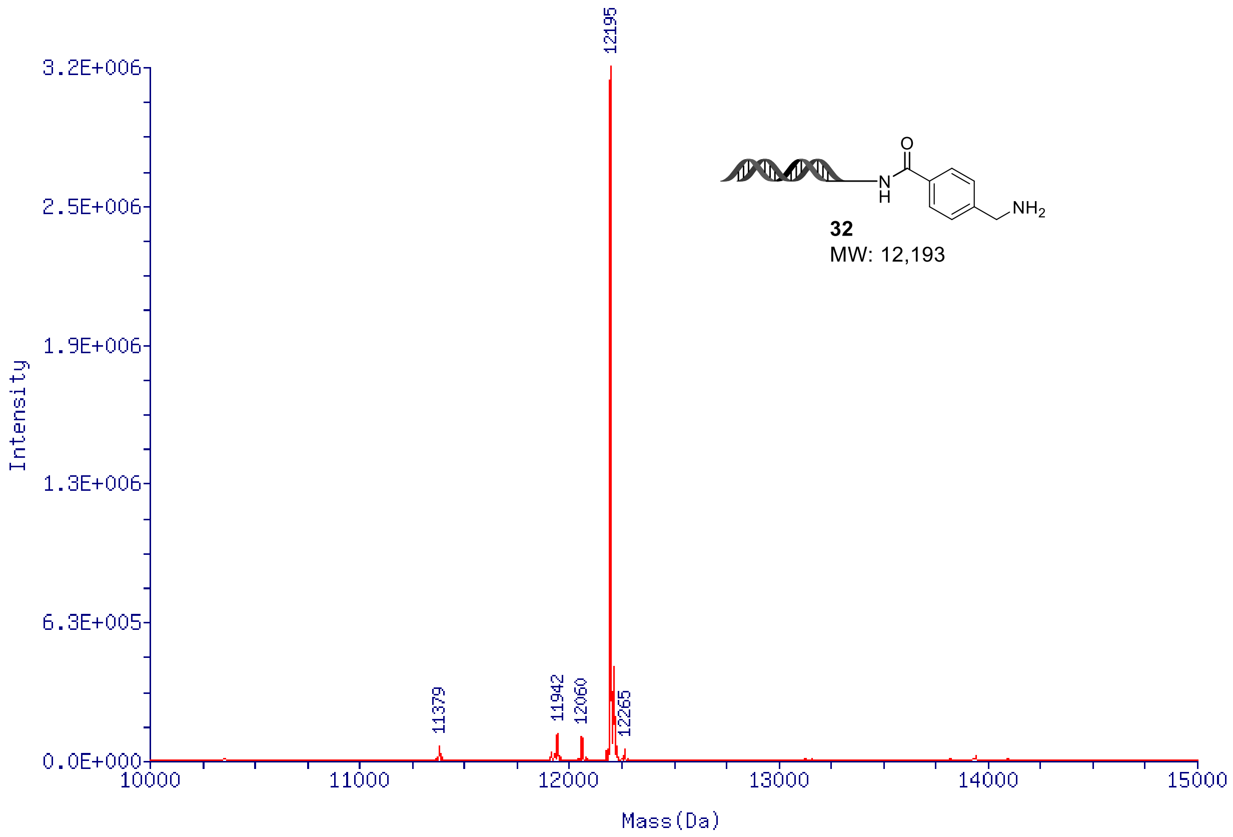

12195
11379
11942
12060
12265
Intensity
Mass(Da)
32
MW: 12,193

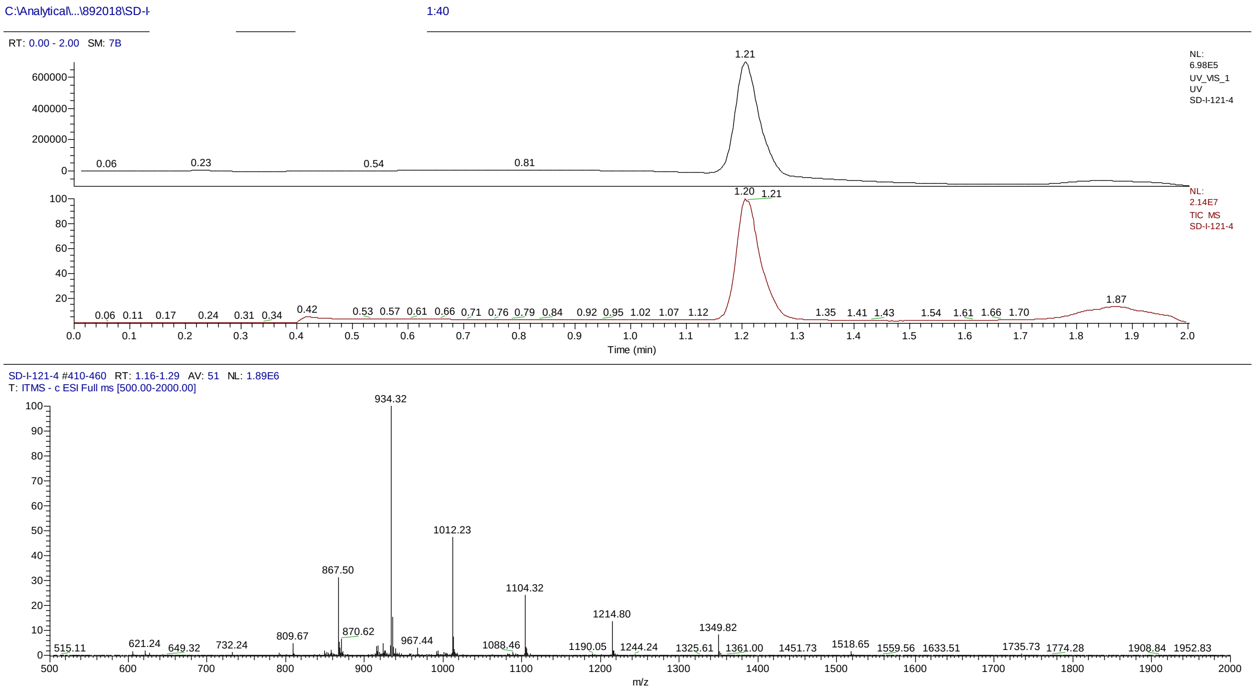
C:\Analytical\...\892018\SD-I
1:40
RT: 0.00 - 2.00 SM: 7B
NL: 6.98E5 UV_VIS_1 UV SD-I-121-4
NL: 2.14E7 TIC MS SD-I-121-4
Time (min)
SD-I-121-4 #410-460 RT: 1.16-1.29 AV: 51 NL: 1.89E6
T: ITMS - c ESI Full ms [500.00-2000.00]
934.32
1012.23
867.50
1104.32
1214.80
1349.82
m/z

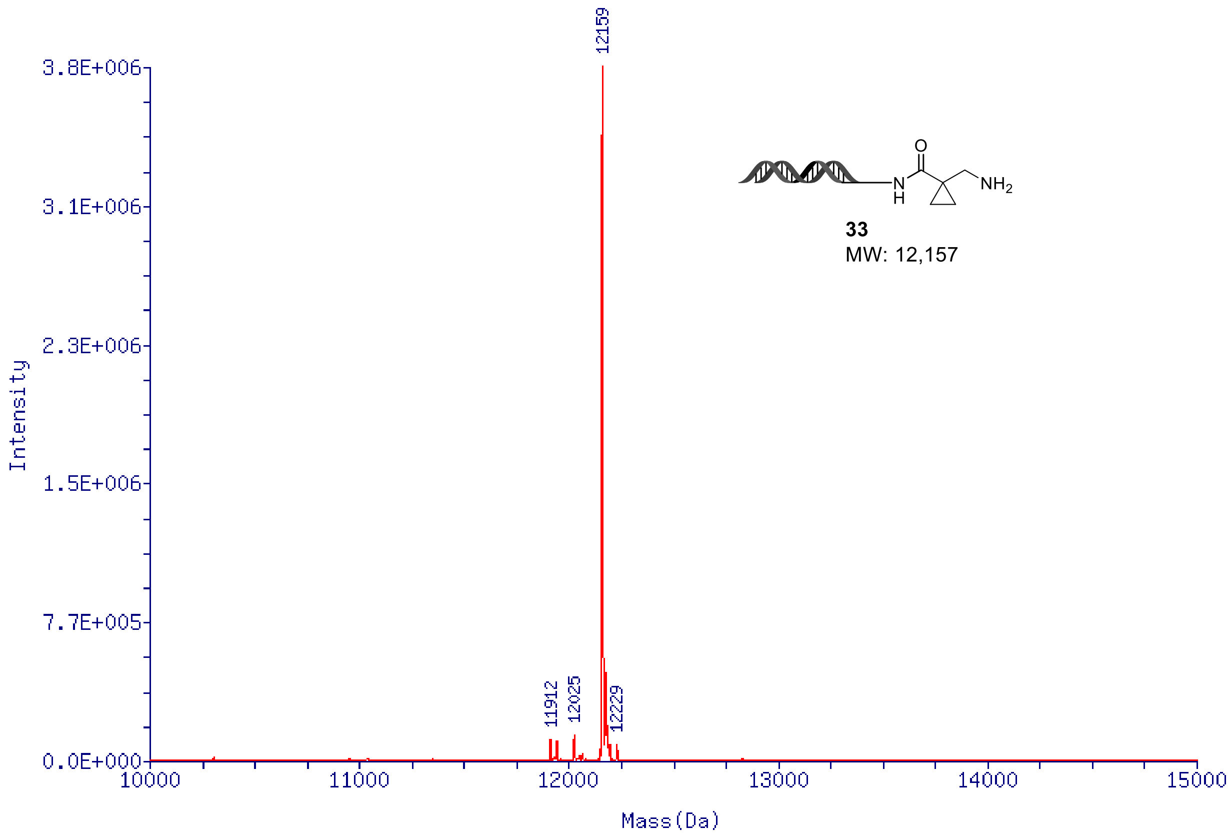
12159
11912
12025
12229
Intensity
Mass(Da)
33
MW: 12,157

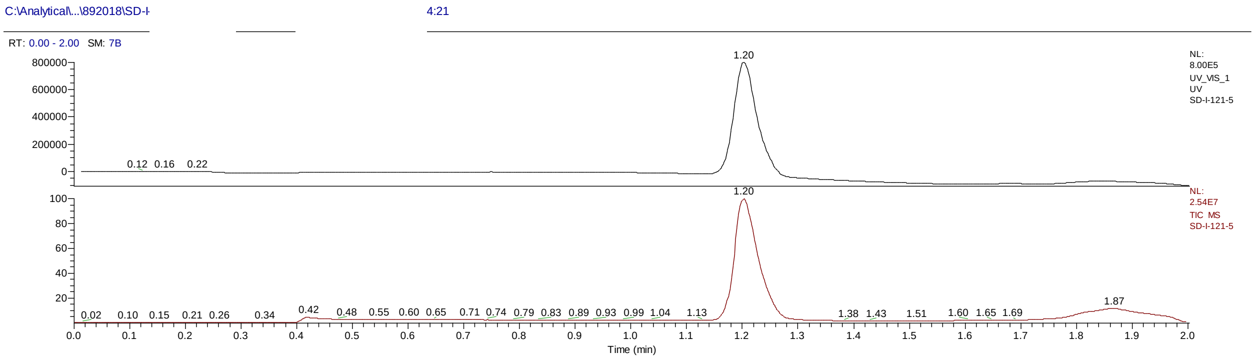
C:\Analytical\...\892018\SD-I
4:21
RT: 0.00 - 2.00 SM: 7B
NL: 8.00E5 UV_VIS_1 UV SD-I-121-5
NL: 2.54E7 TIC MS SD-I-121-5
Time (min)
1.20
1.87

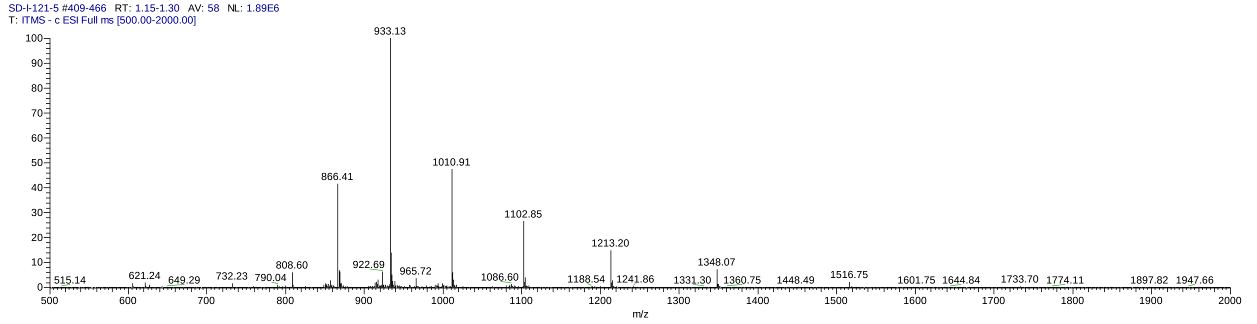
SD-I-121-5 #409-466 RT: 1.15-1.30 AV: 58 NL: 1.89E6
T: ITMS - c ESI Full ms [500.00-2000.00]
933.13
1010.91
866.41
1102.85
1213.20
1348.07
m/z

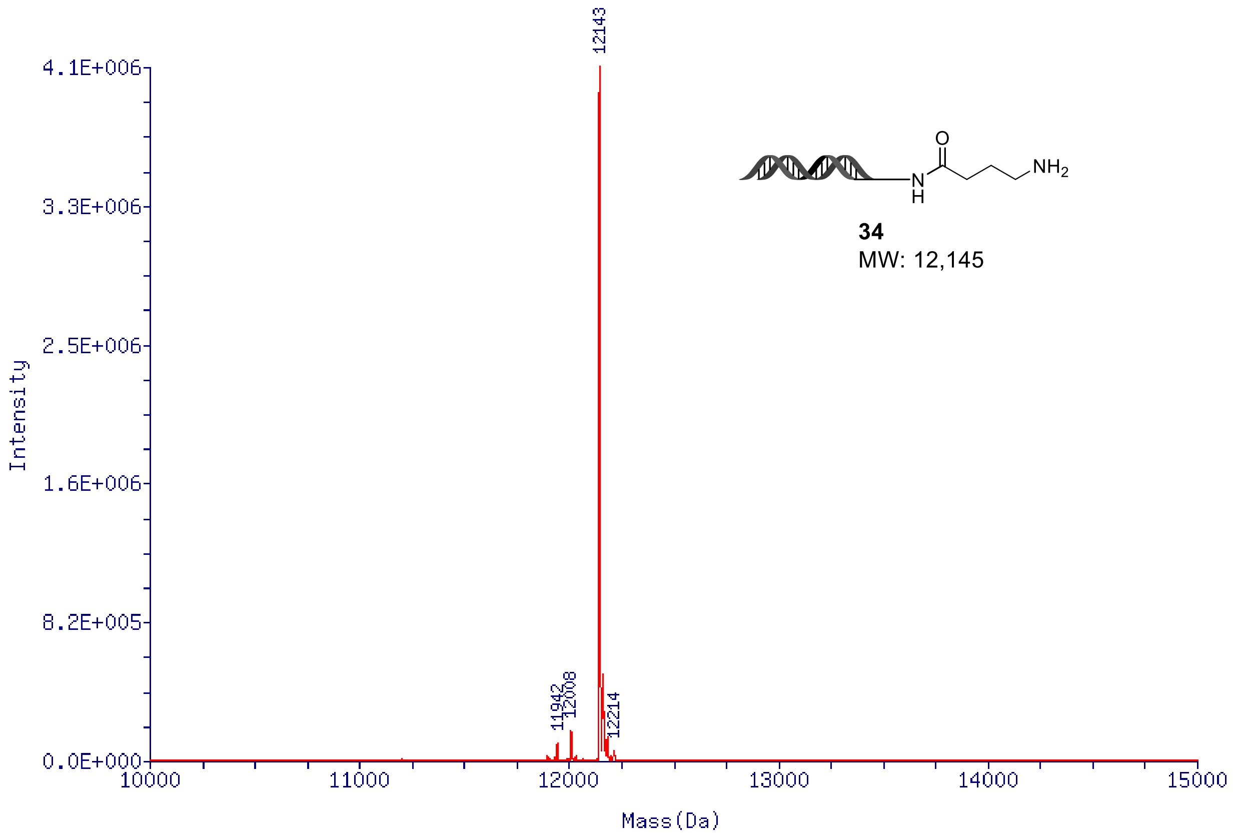
12143
11942
12008
12214
Intensity
Mass(Da)
34
MW: 12,145

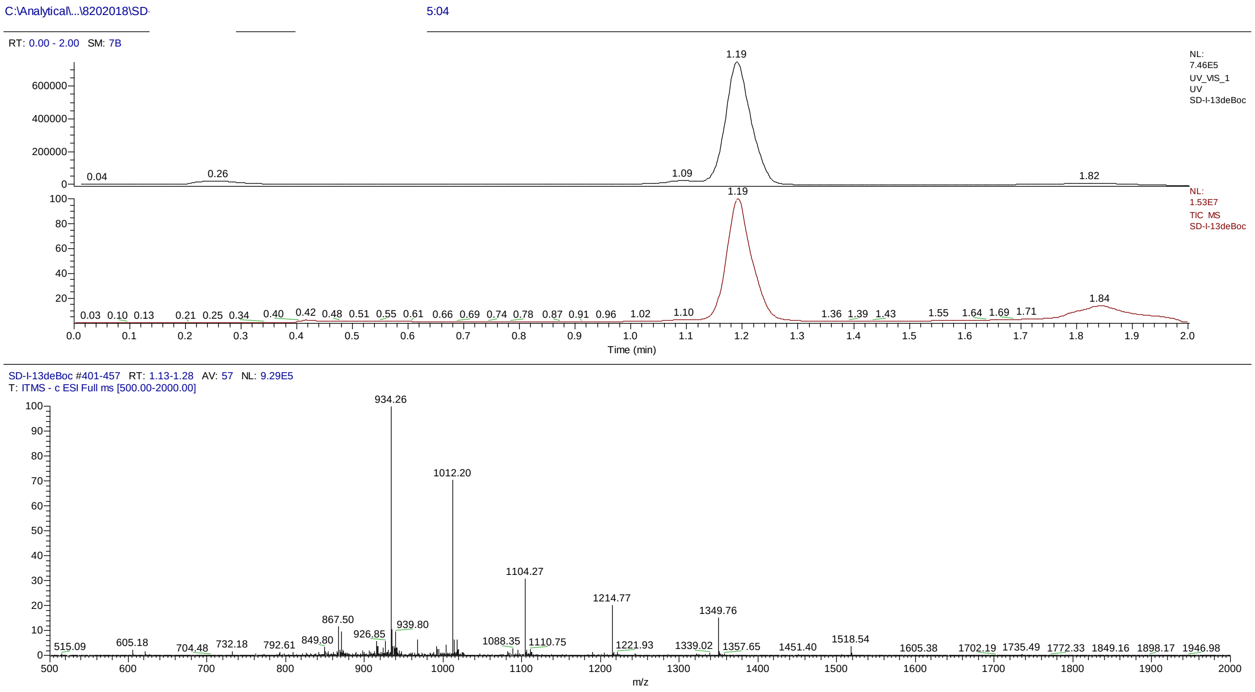
C:\Analytical\...\8202018\SD-
5:04
RT: 0.00 - 2.00 SM: 7B
NL: 7.46E5 UV_VIS_1 UV SD-I-13deBoc
NL: 1.53E7 TIC MS SD-I-13deBoc
Time (min)
SD-I-13deBoc #401-457 RT: 1.13-1.28 AV: 57 NL: 9.29E5
T: ITMS - c ESI Full ms [500.00-2000.00]
934.26
1012.20
1104.27
1214.77
1349.76
m/z

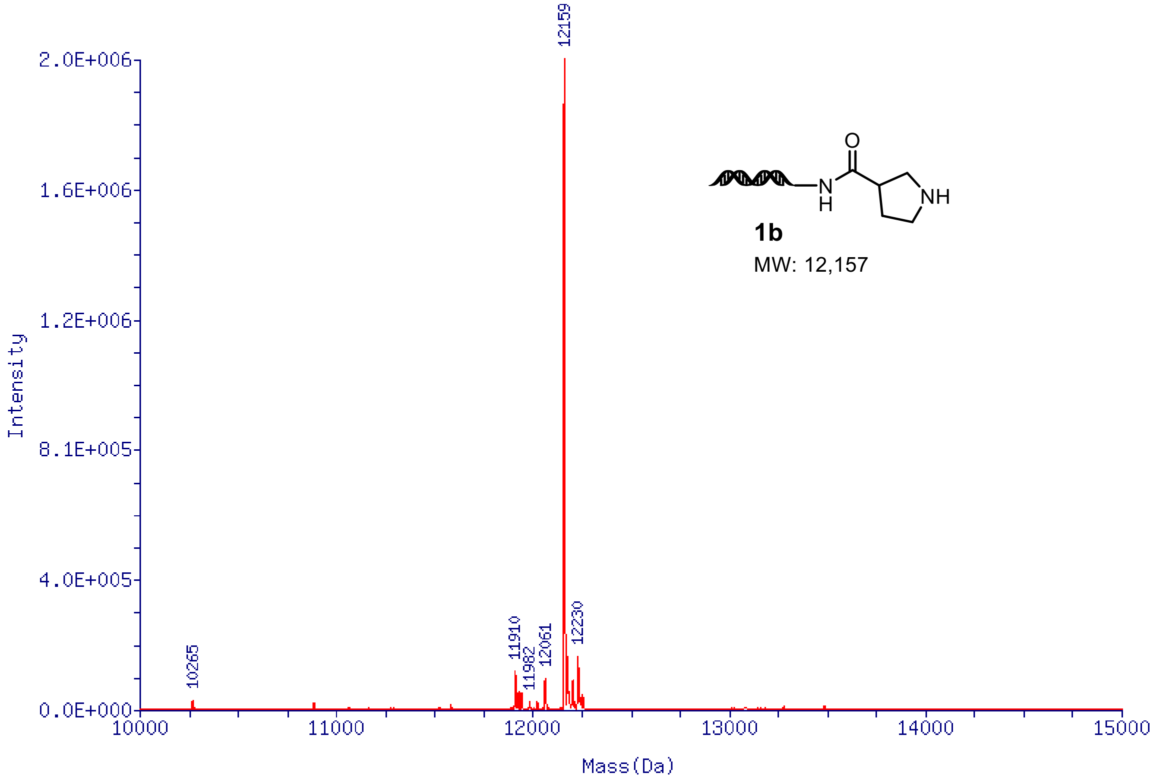
12159
10265
11910
11982
12061
12230
Intensity
Mass(Da)

1b

MW: 12,157

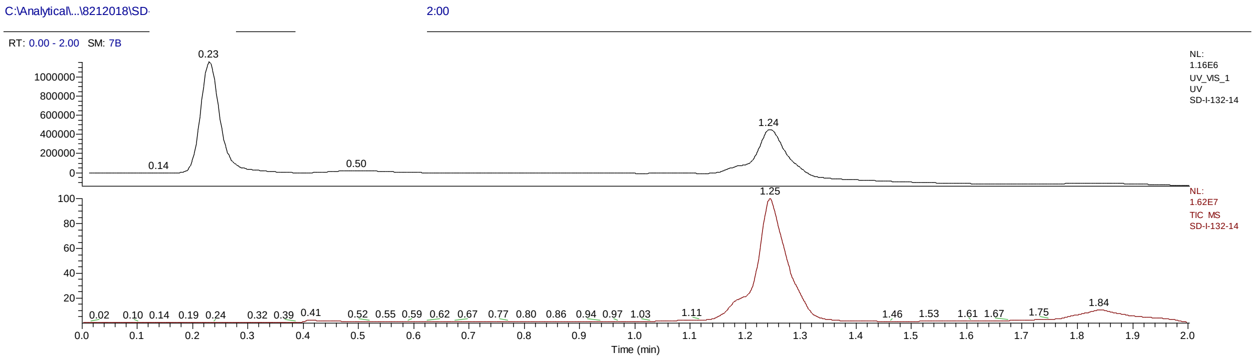
C:\Analytical\...\8212018\SD-
2:00
RT: 0.00 - 2.00 SM: 7B
NL: 1.16E6 UV_VIS_1 UV SD-I-132-14
NL: 1.62E7 TIC MS SD-I-132-14
Time (min)

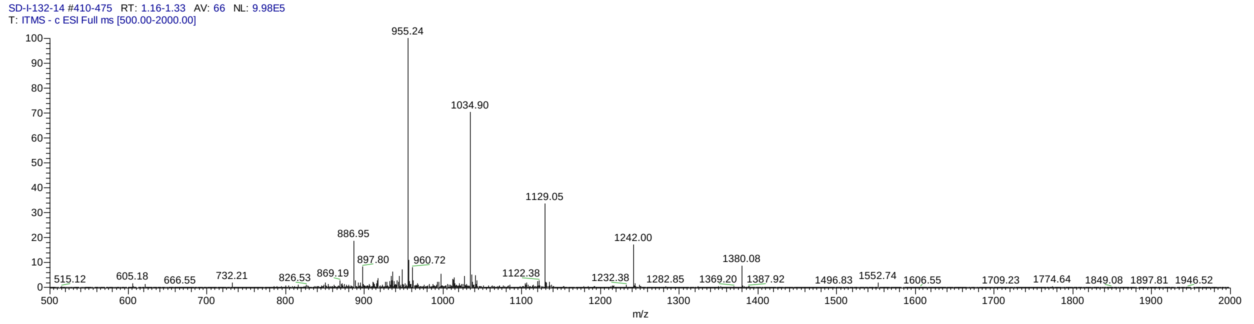
SD-I-132-14 #410-475 RT: 1.16-1.33 AV: 66 NL: 9.98E5
T: ITMS - c ESI Full ms [500.00-2000.00]
955.24
1034.90
1129.05
1242.00
886.95
1380.08
m/z

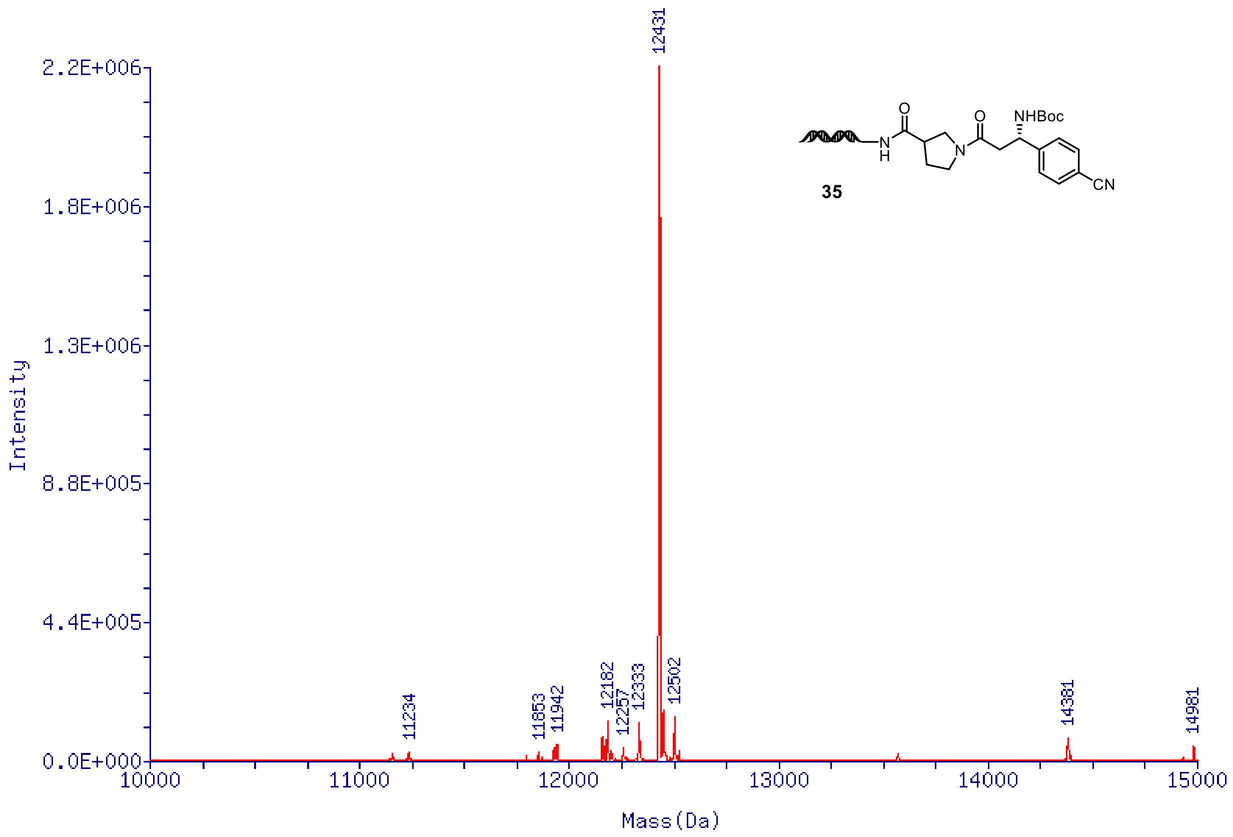
12431
Intensity
Mass(Da)
NHBoc
CN
35

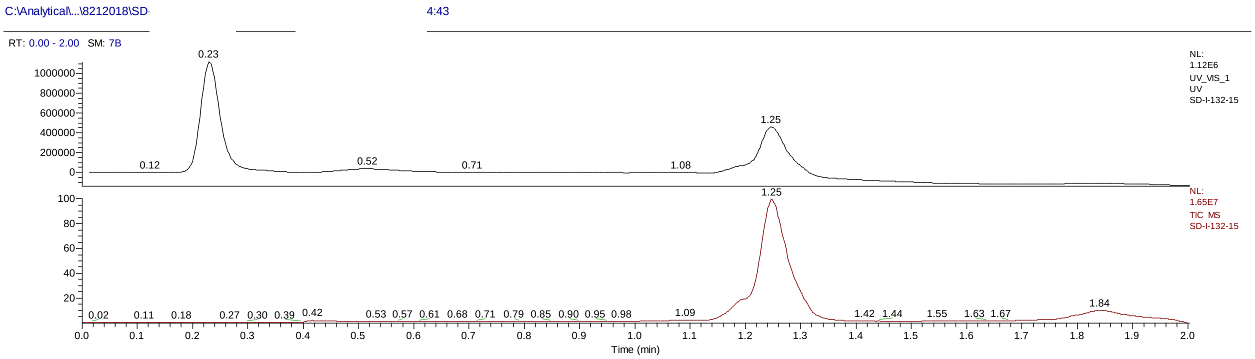
C:\Analytical\...\8212018\SD
4:43
RT: 0.00 - 2.00 SM: 7B
NL: 1.12E6 UV_VIS_1 UV SD-I-132-15
0.23
0.12
0.52
0.71
1.08
1.25
NL: 1.65E7 TIC MS SD-I-132-15
0.02 0.11 0.18 0.27 0.30 0.39 0.42 0.53 0.57 0.61 0.68 0.71 0.79 0.85 0.90 0.95 0.98 1.09 1.42 1.44 1.55 1.63 1.67 1.84
Time (min)

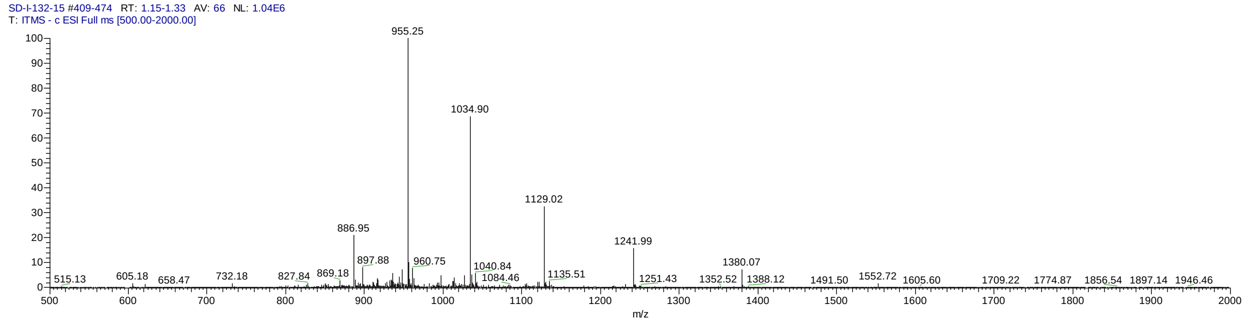
SD-I-132-15 #409-474 RT: 1.15-1.33 AV: 66 NL: 1.04E6
T: ITMS - c ESI Full ms [500.00-2000.00]
955.25
1034.90
1129.02
886.95
1241.99
897.88
960.75
1040.84
1084.46
1135.51
1380.07
515.13 605.18 658.47 732.18 827.84 869.18 1251.43 1352.52 1388.12 1491.50 1552.72 1605.60 1709.22 1774.87 1856.54 1897.14 1946.46
m/z

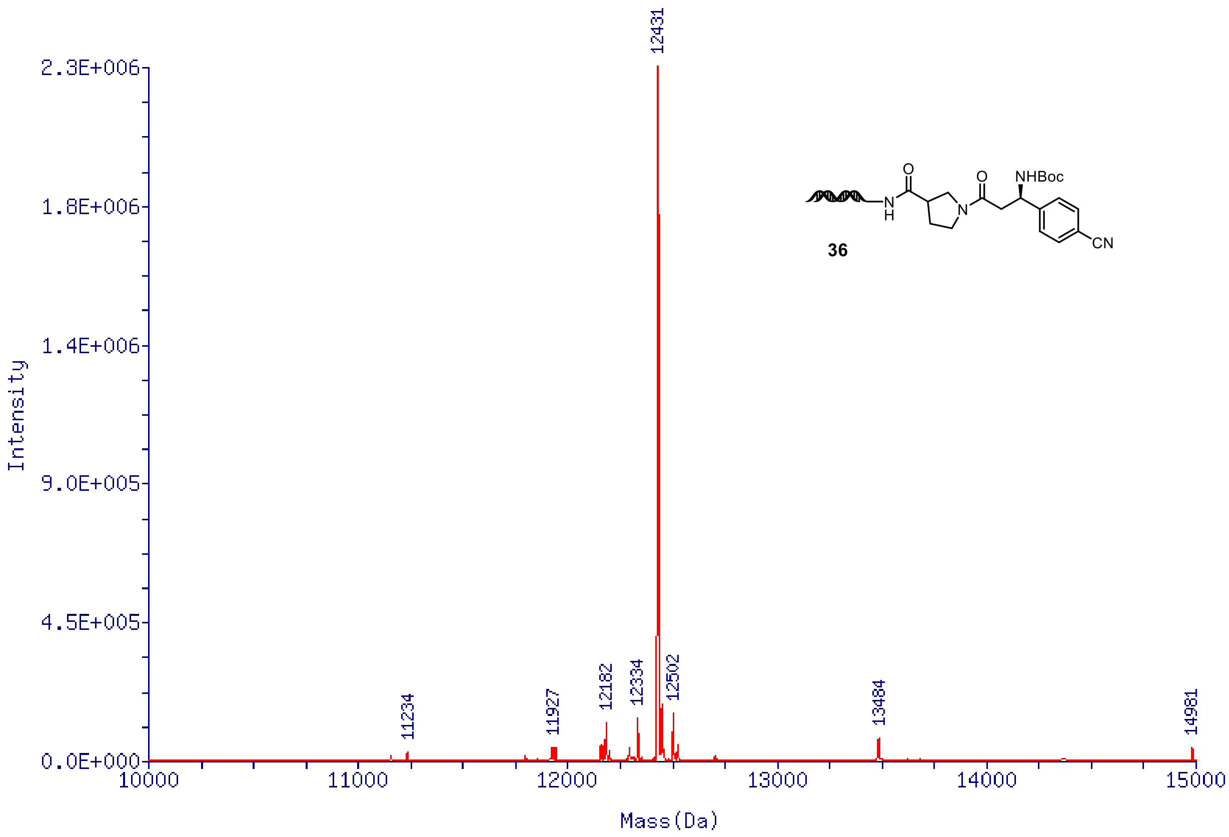
12431
11234
11927
12182
12334
12502
13484
14981
Intensity
Mass(Da)
NHBoc
CN
36

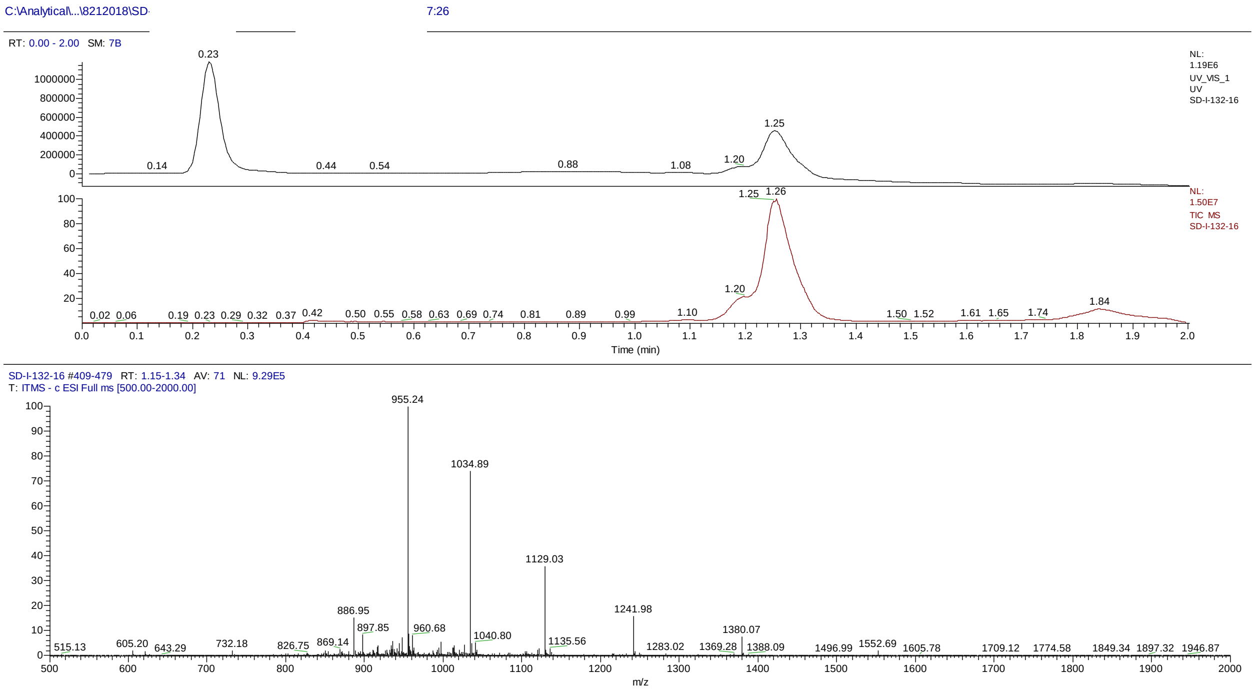
C:\Analytical\...\8212018\SD-
7:26
RT: 0.00 - 2.00 SM: 7B
NL: 1.19E6 UV_VIS_1 UV SD-I-132-16
NL: 1.50E7 TIC MS SD-I-132-16
Time (min)
SD-I-132-16 #409-479 RT: 1.15-1.34 AV: 71 NL: 9.29E5
T: ITMS - c ESI Full ms [500.00-2000.00]
955.24
1034.89
1129.03
1241.98
m/z

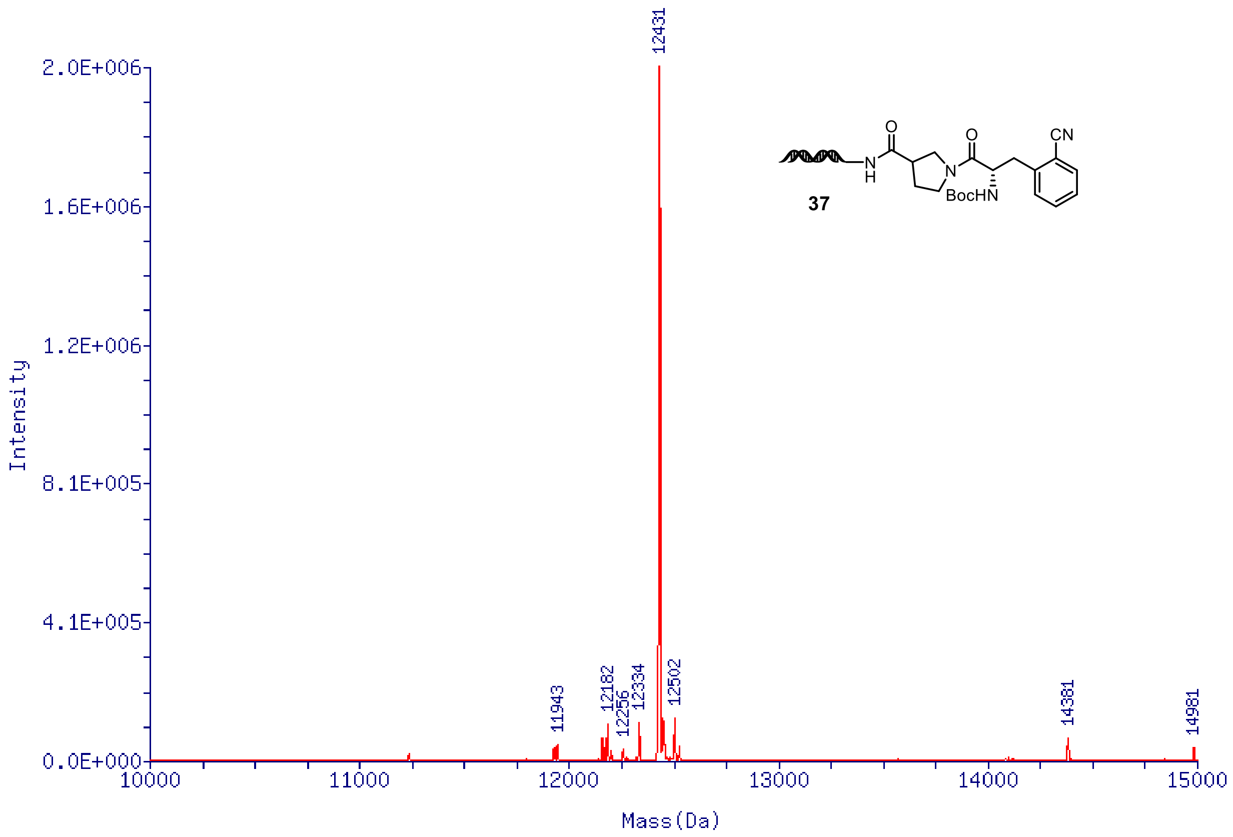
Intensity
Mass(Da)
12431
11943
12182
12256
12334
12502
14381
14981
CN
BocHN
37

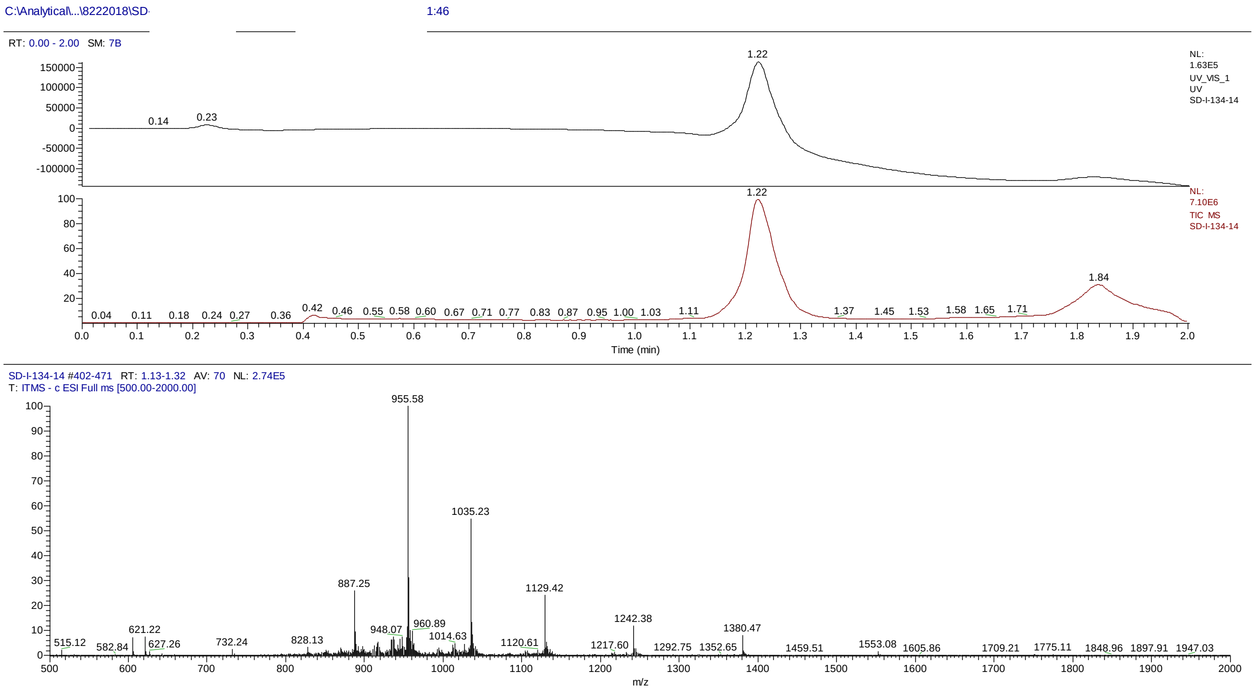
C:\Analytical\...\8222018\SD
1:46
RT: 0.00 - 2.00 SM: 7B
NL: 1.63E5 UV_VIS_1 UV SD-I-134-14
NL: 7.10E6 TIC MS SD-I-134-14
Time (min)
SD-I-134-14 #402-471 RT: 1.13-1.32 AV: 70 NL: 2.74E5
T: ITMS - c ESI Full ms [500.00-2000.00]
955.58
1035.23
887.25
1129.42
1242.38
1380.47
m/z

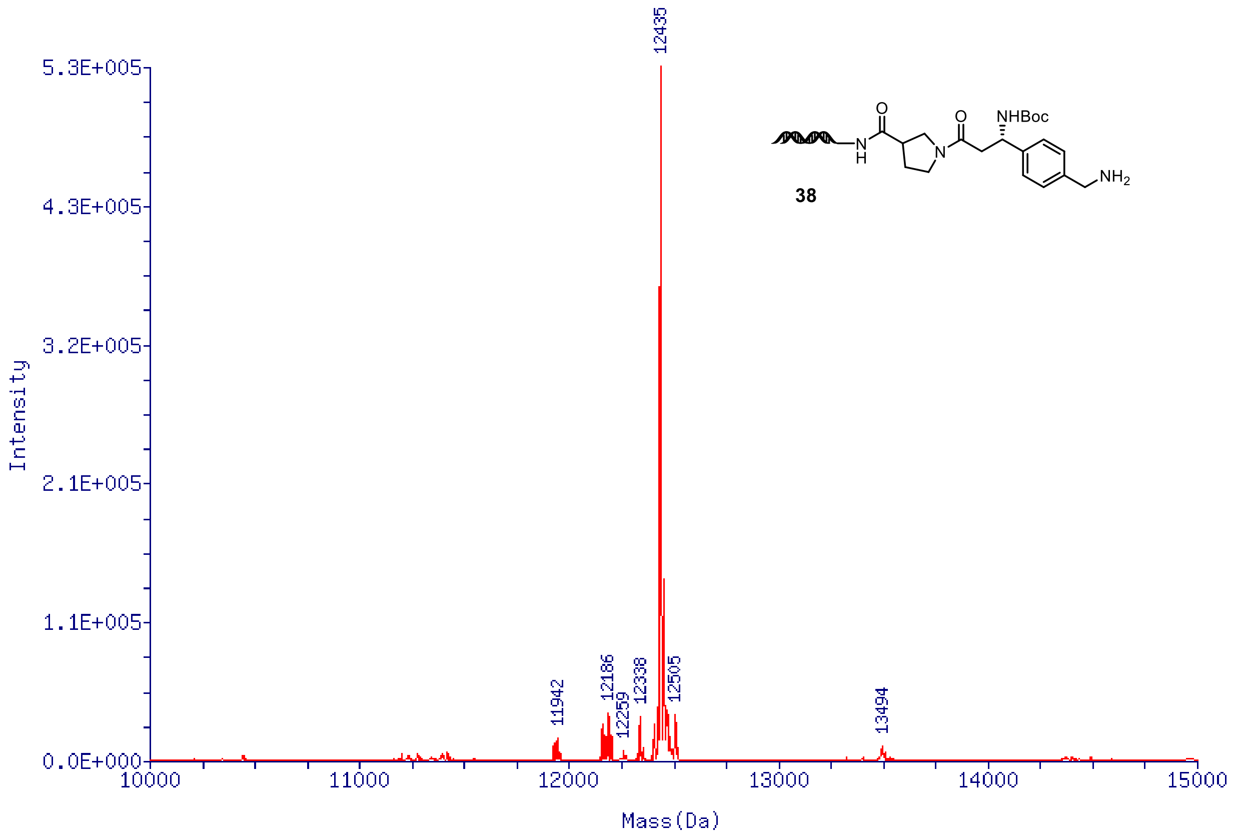
Intensity
Mass(Da)
12435
11942
12186
12259
12338
12505
13494
NHBoc
NH2
38

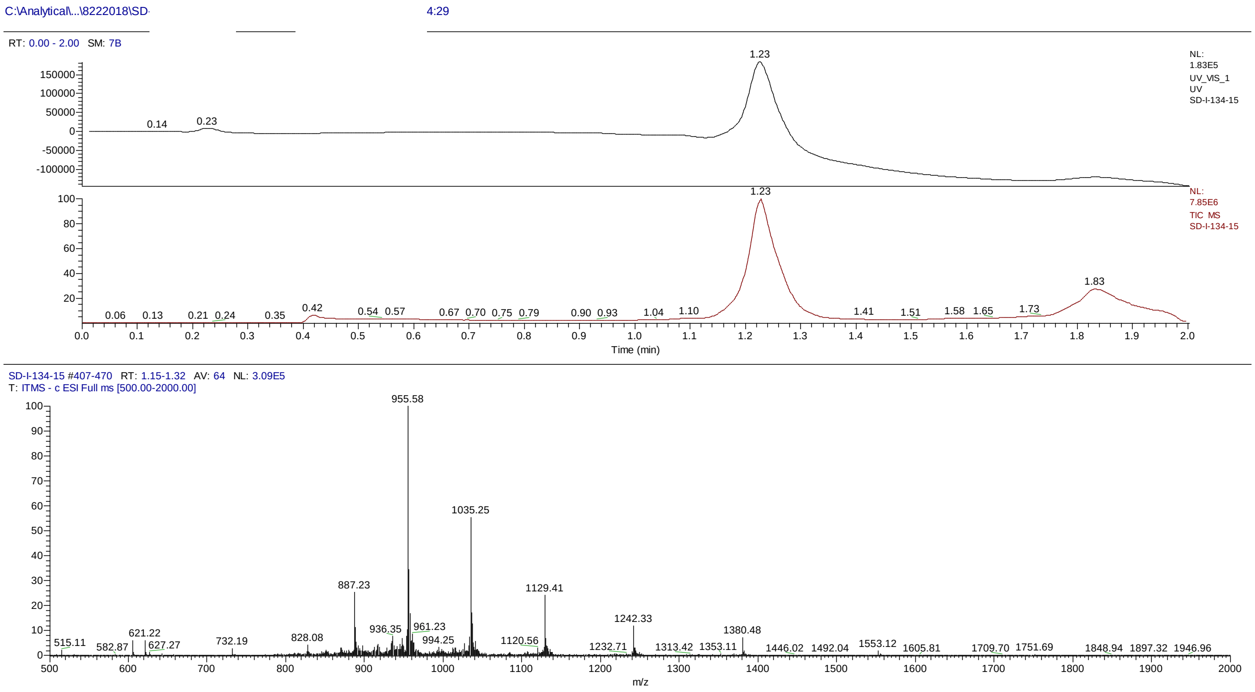
C:\Analytical\...\8222018\SD-
4:29
RT: 0.00 - 2.00 SM: 7B
NL: 1.83E5 UV_VIS_1 UV SD-I-134-15
NL: 7.85E6 TIC MS SD-I-134-15
Time (min)
SD-I-134-15 #407-470 RT: 1.15-1.32 AV: 64 NL: 3.09E5
T: ITMS - c ESI Full ms [500.00-2000.00]
955.58
1035.25
887.23
1129.41
1242.33
1380.48
m/z

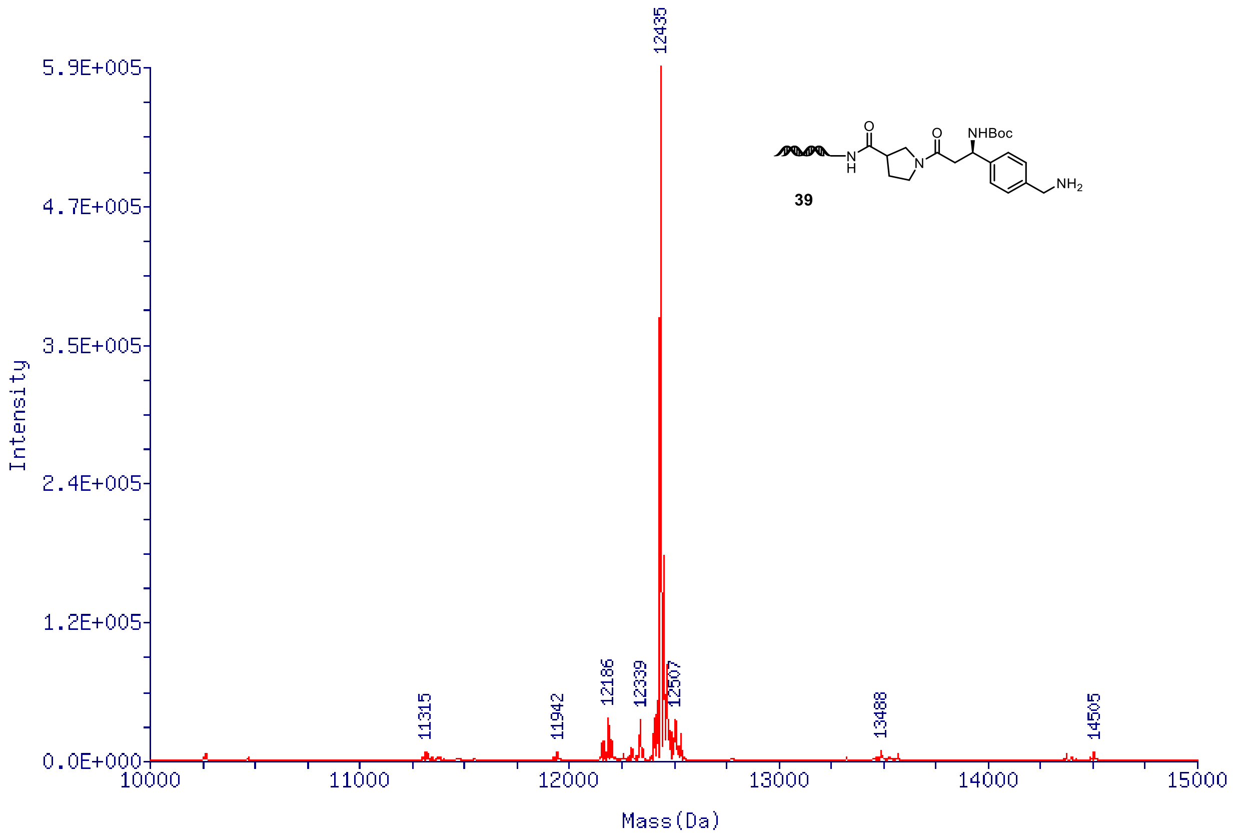
12435
12186
12339
12507
11315
11942
13488
14505
Intensity
Mass(Da)
NHBoc
NH2

39

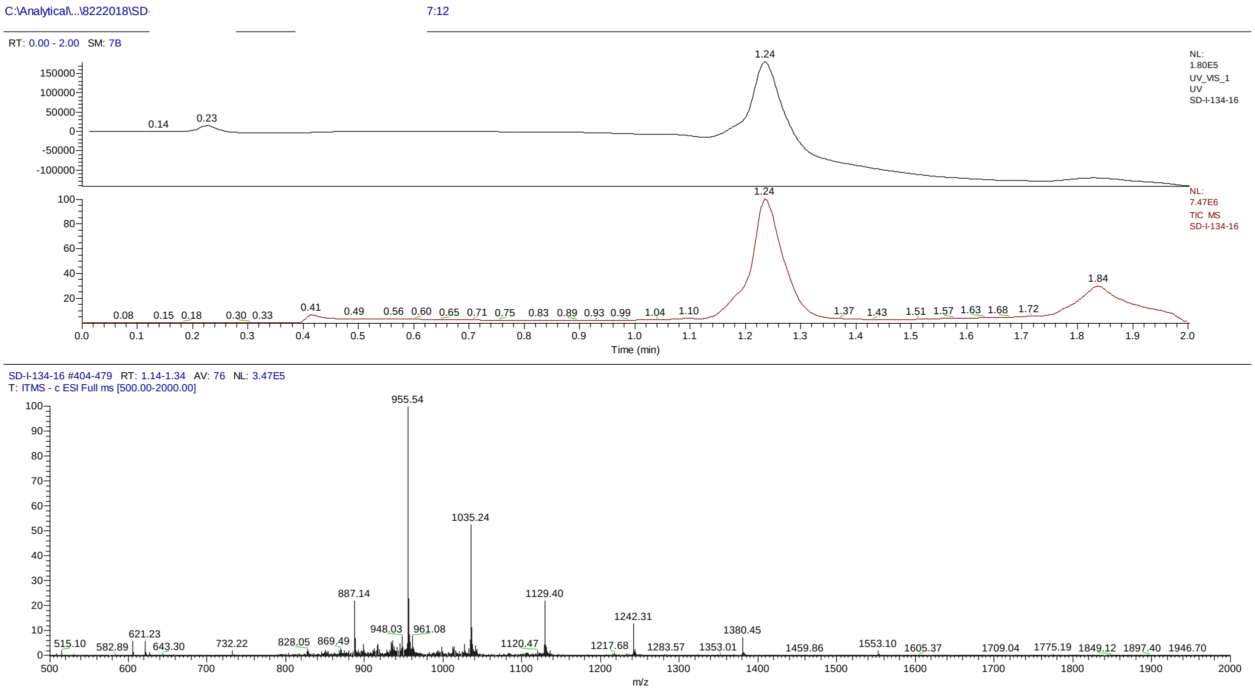
C:\Analytical\...\8222018\SD-
7:12
RT: 0.00 - 2.00 SM: 7B
NL: 1.80E5 UV_VIS_1 UV SD-I-134-16
NL: 7.47E6 TIC MS SD-I-134-16
1.24
1.84
Time (min)
SD-I-134-16 #404-479 RT: 1.14-1.34 AV: 76 NL: 3.47E5
T: ITMS - c ESI Full ms [500.00-2000.00]
955.54
1035.24
1129.40
887.14
1242.31
1380.45
m/z

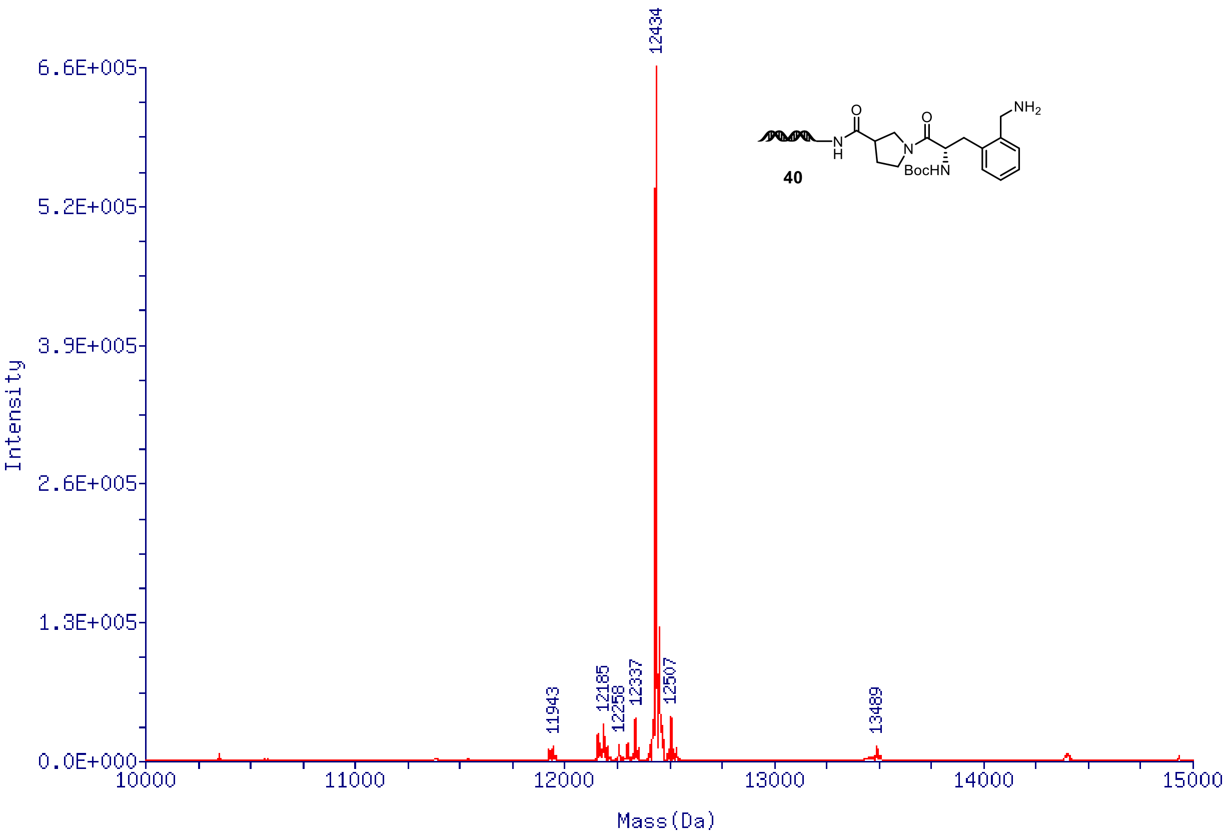
12434
Intensity
Mass(Da)
11943
12185
12258
12337
12507
13489
NH2
BocHN
40

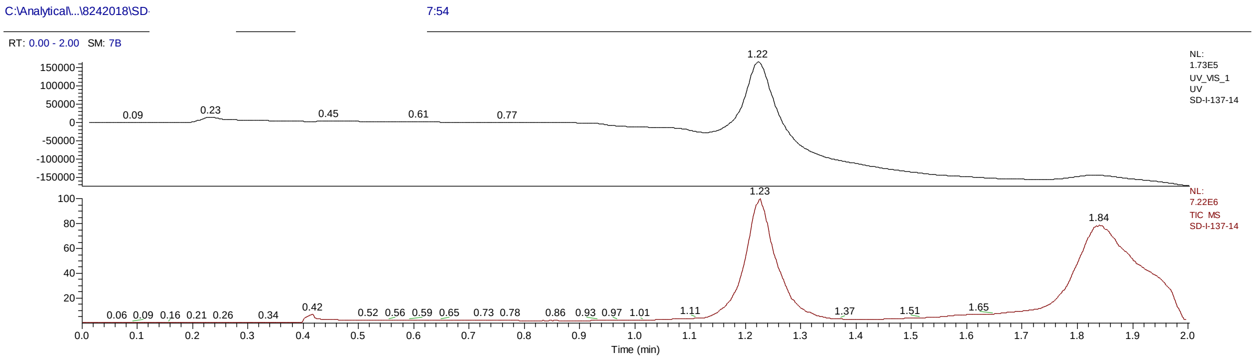
C:\Analytical\...\8242018\SD
7:54
RT: 0.00 - 2.00 SM: 7B
NL: 1.73E5 UV_VIS_1 UV SD-I-137-14
NL: 7.22E6 TIC MS SD-I-137-14
Time (min)

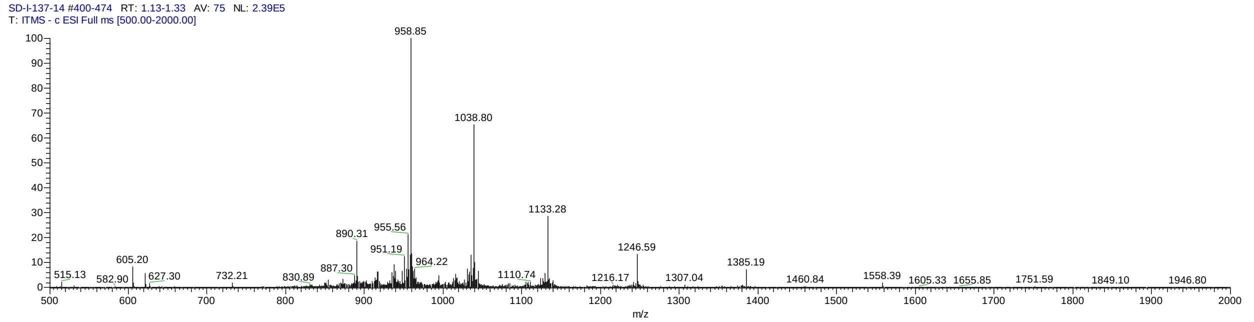
SD-I-137-14 #400-474 RT: 1.13-1.33 AV: 75 NL: 2.39E5
T: ITMS - c ESI Full ms [500.00-2000.00]
958.85
1038.80
1133.28
1246.59
1385.19
m/z

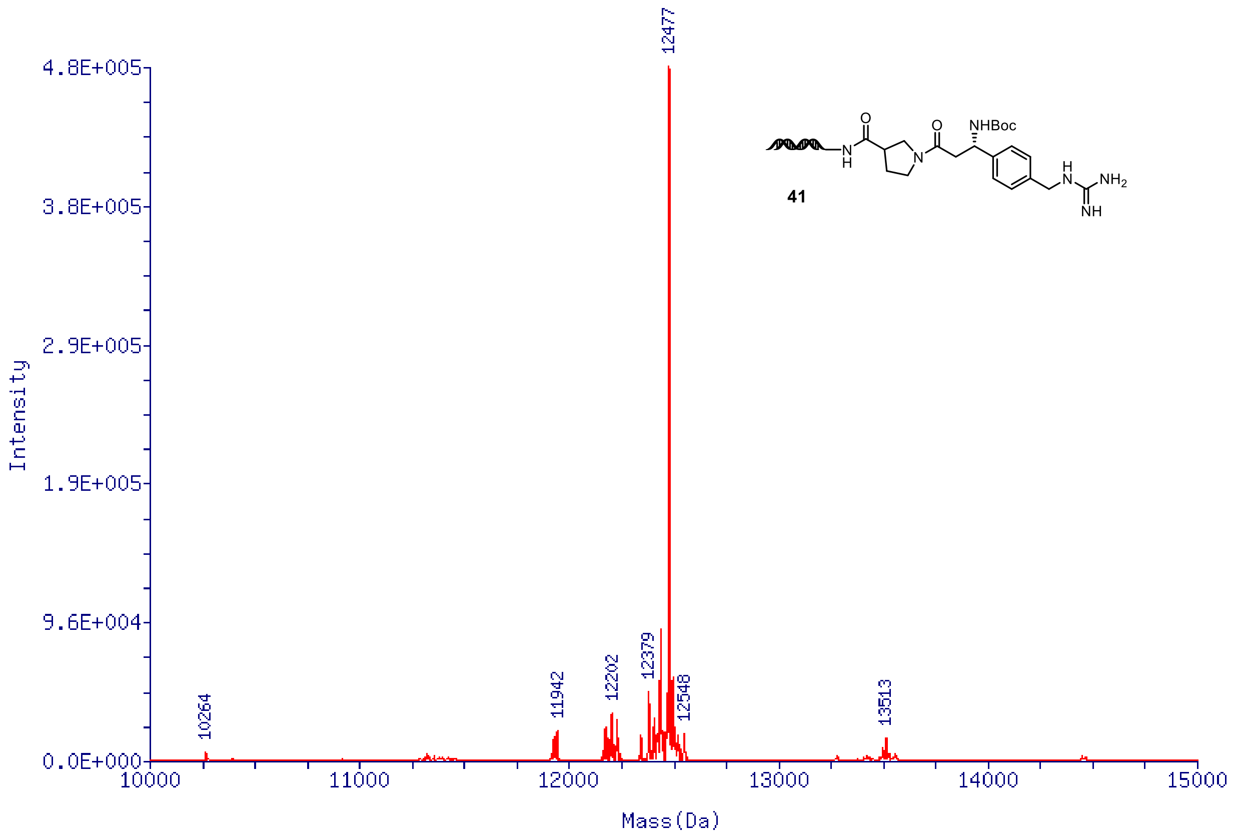
12477
Intensity
Mass(Da)
NHBoc
NH2
NH
41

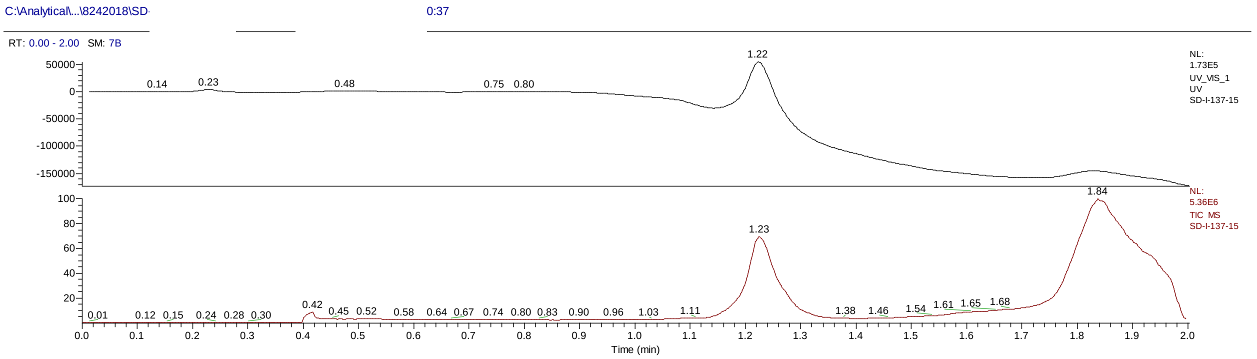
C:\Analytical\...\8242018\SD
0:37
RT: 0.00 - 2.00 SM: 7B
NL: 1.73E5 UV_VIS_1 UV SD-I-137-15
NL: 5.36E6 TIC MS SD-I-137-15
Time (min)

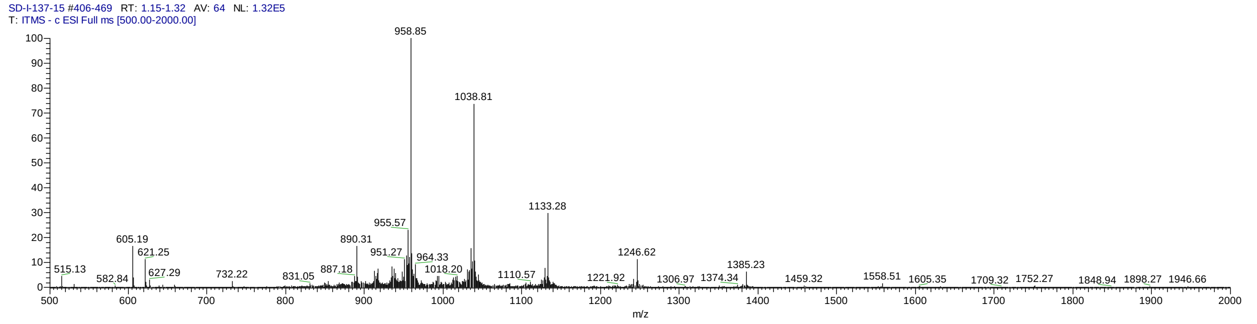
SD-I-137-15 #406-469 RT: 1.15-1.32 AV: 64 NL: 1.32E5
T: ITMS - c ESI Full ms [500.00-2000.00]
958.85
1038.81
1133.28
1246.62
605.19
m/z

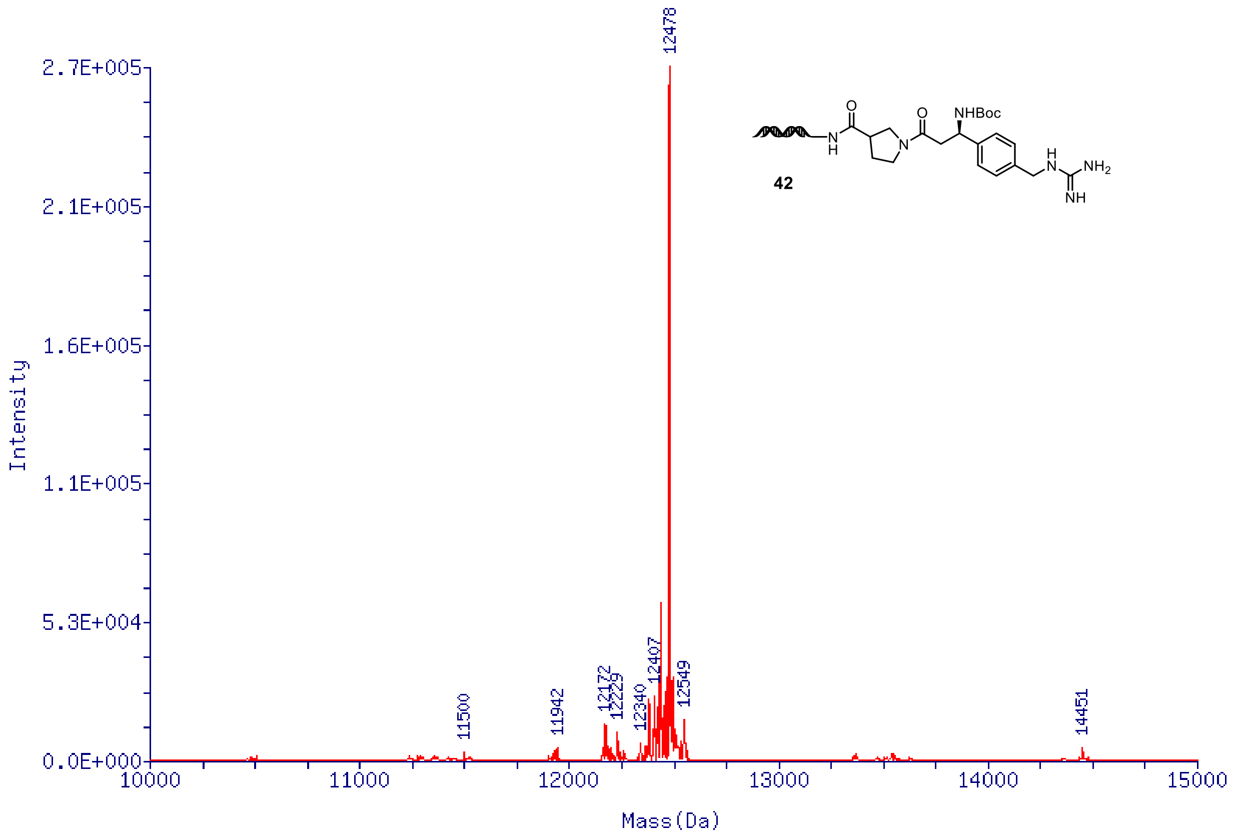
12478
Intensity
Mass(Da)
42
NHBoc

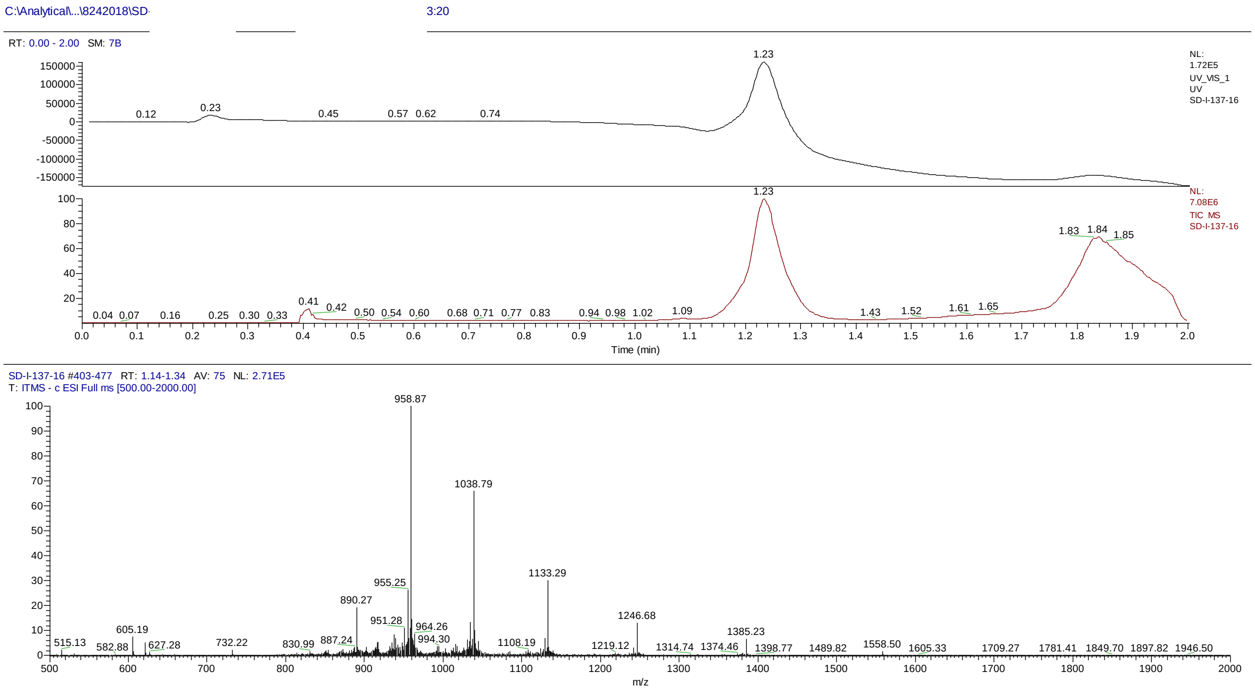
C:\Analytical\...\8242018\SD-
3:20
RT: 0.00 - 2.00 SM: 7B
NL: 1.72E5 UV_VIS_1 UV SD-I-137-16
NL: 7.08E6 TIC MS SD-I-137-16
Time (min)
SD-I-137-16 #403-477 RT: 1.14-1.34 AV: 75 NL: 2.71E5
T: ITMS - c ESI Full ms [500.00-2000.00]
958.87
1038.79
1133.29
1246.68
m/z

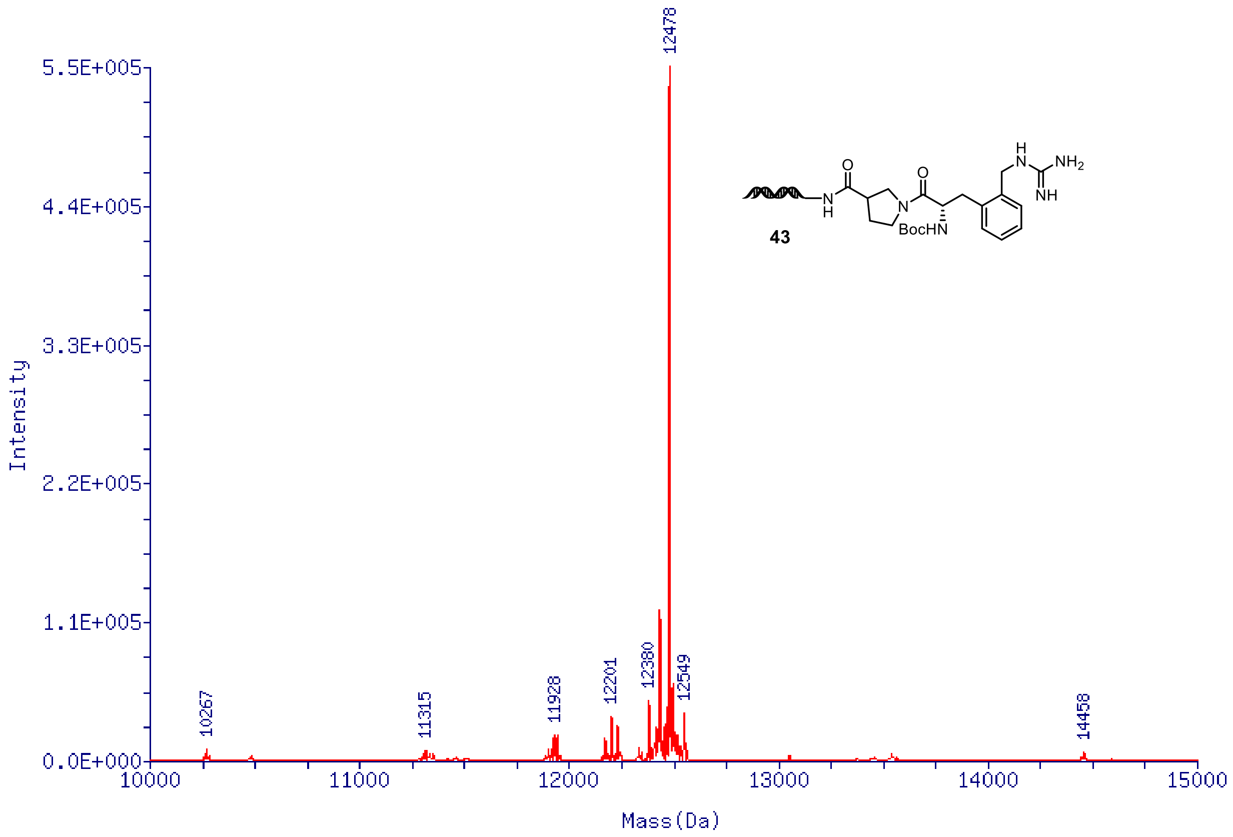
12478
Intensity
Mass(Da)
43
BocHN

7d) Chemistry validation

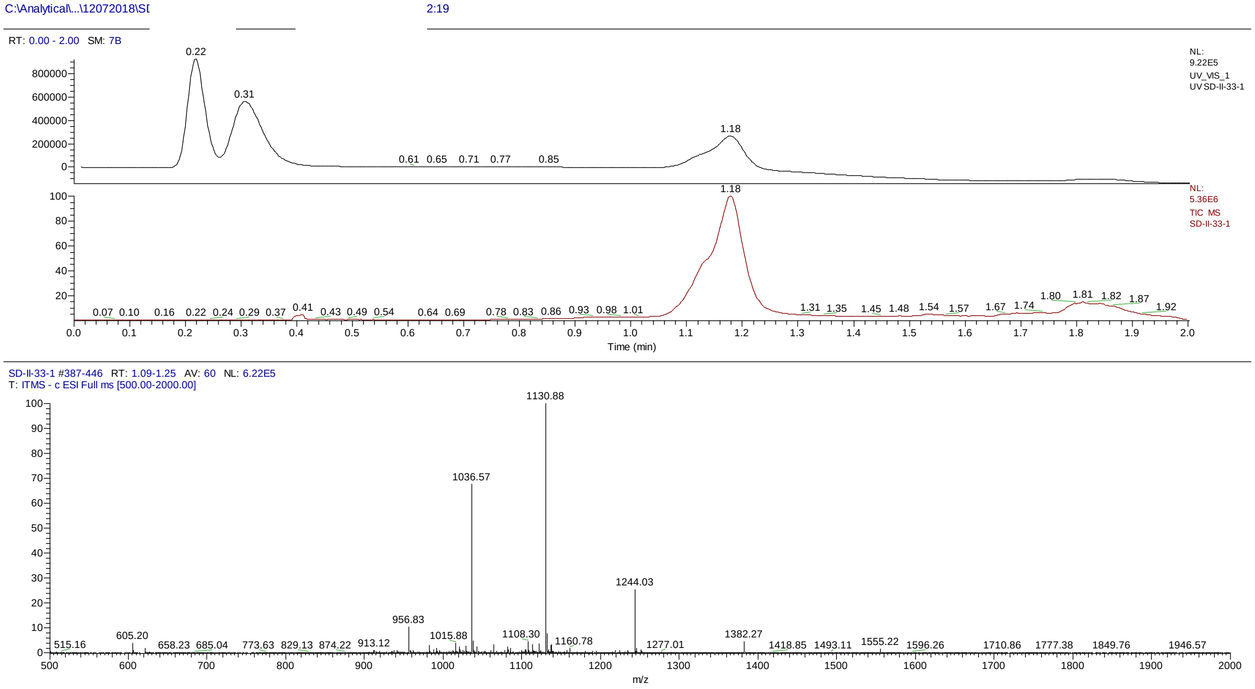

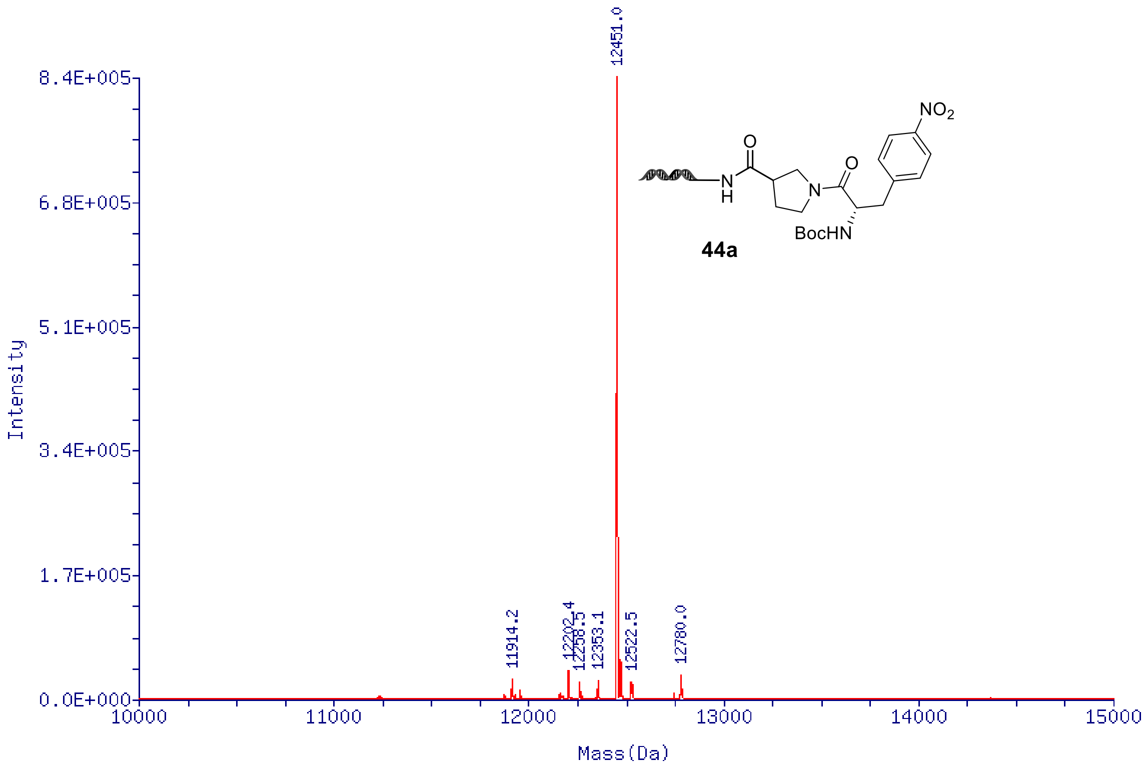

C:\Analytical\...\12072018\SI 5:12

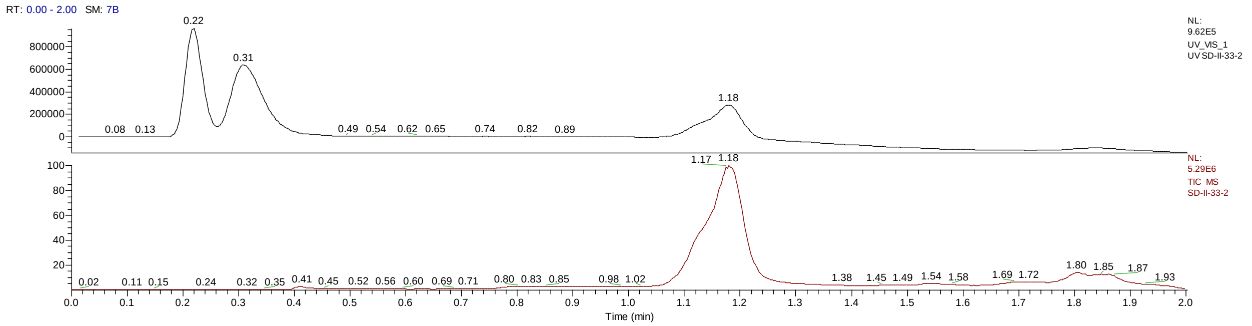

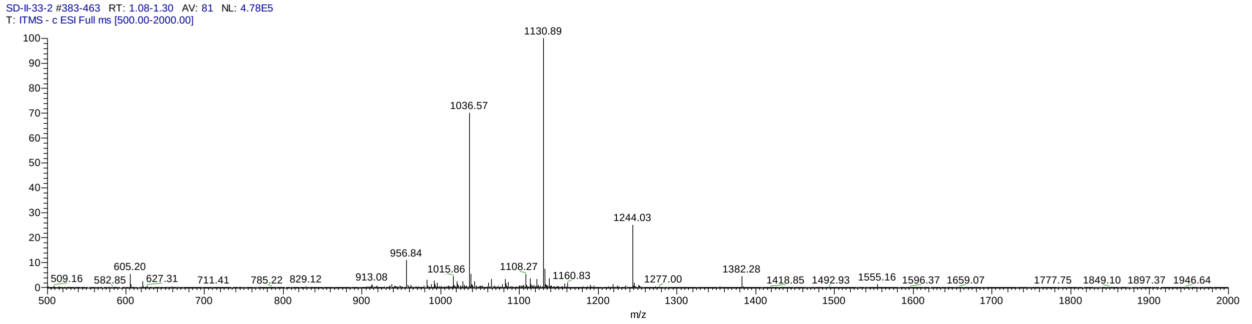

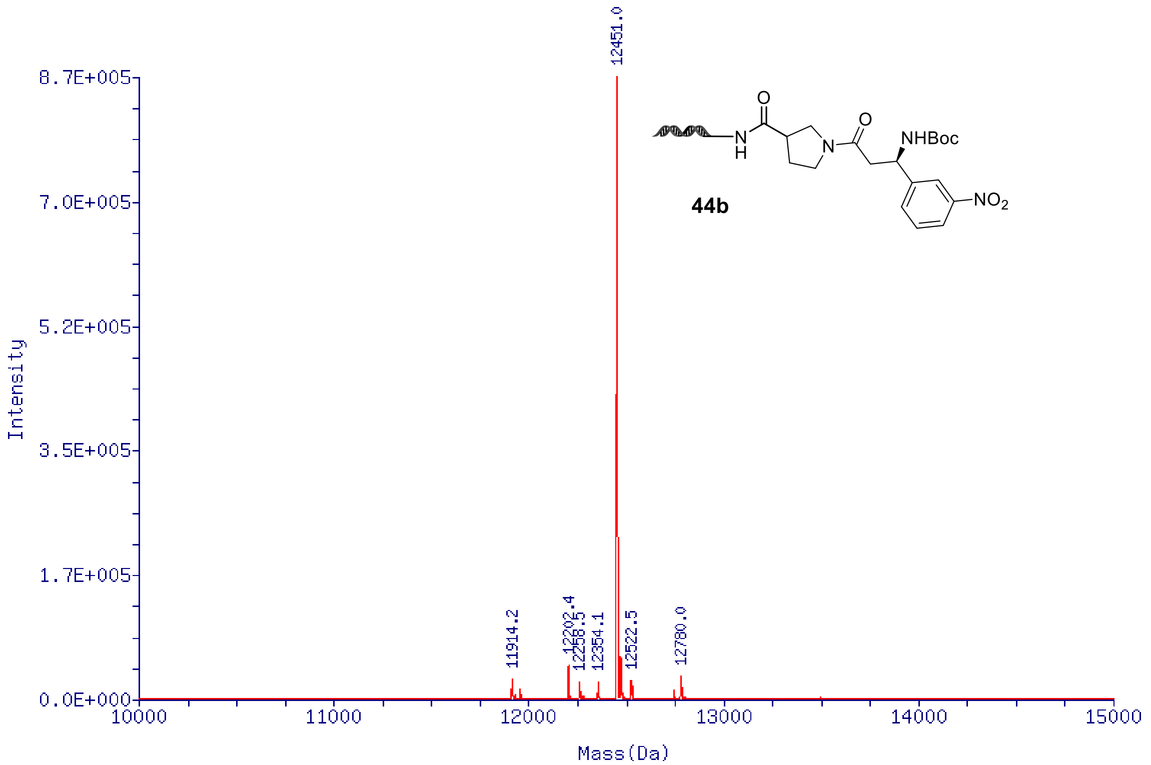

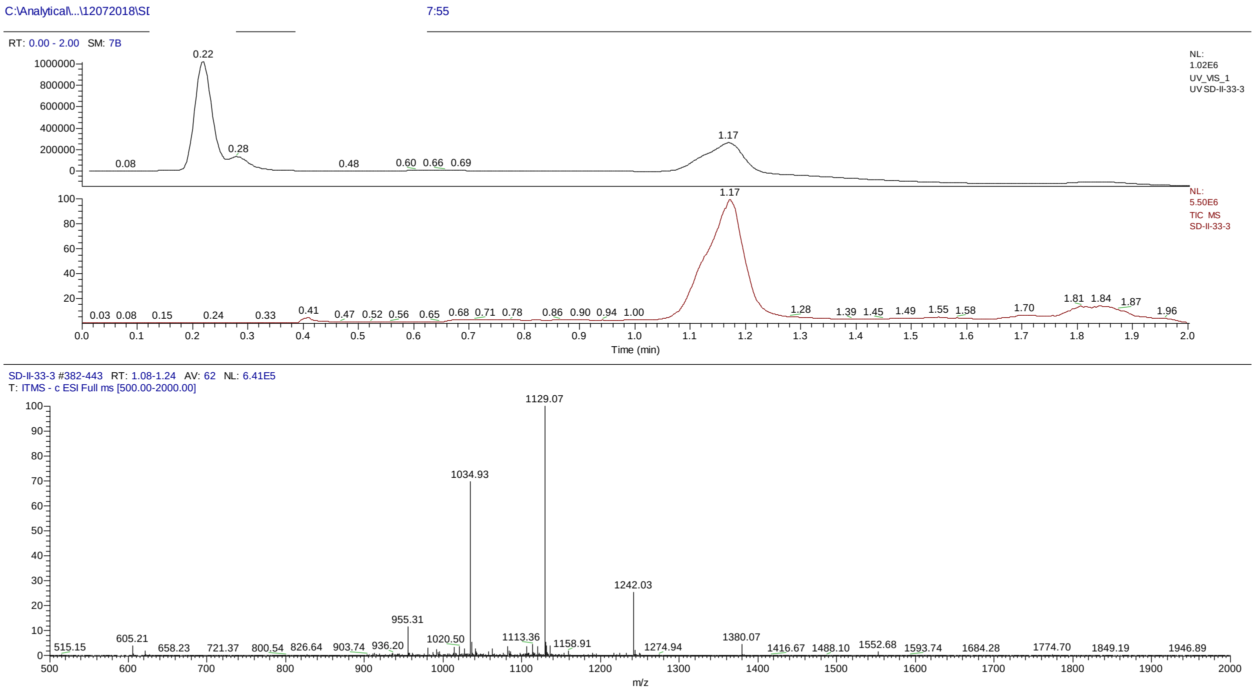
C:\Analytical\...\12072018\SI
7:55
RT: 0.00 - 2.00 SM: 7B
NL: 1.02E6 UV_VIS_1 UV SD-II-33-3
NL: 5.50E6 TIC MS SD-II-33-3
Time (min)
SD-II-33-3 #382-443 RT: 1.08-1.24 AV: 62 NL: 6.41E5
T: ITMS - c ESI Full ms [500.00-2000.00]
1129.07
1034.93
1242.03
955.31
1380.07
m/z

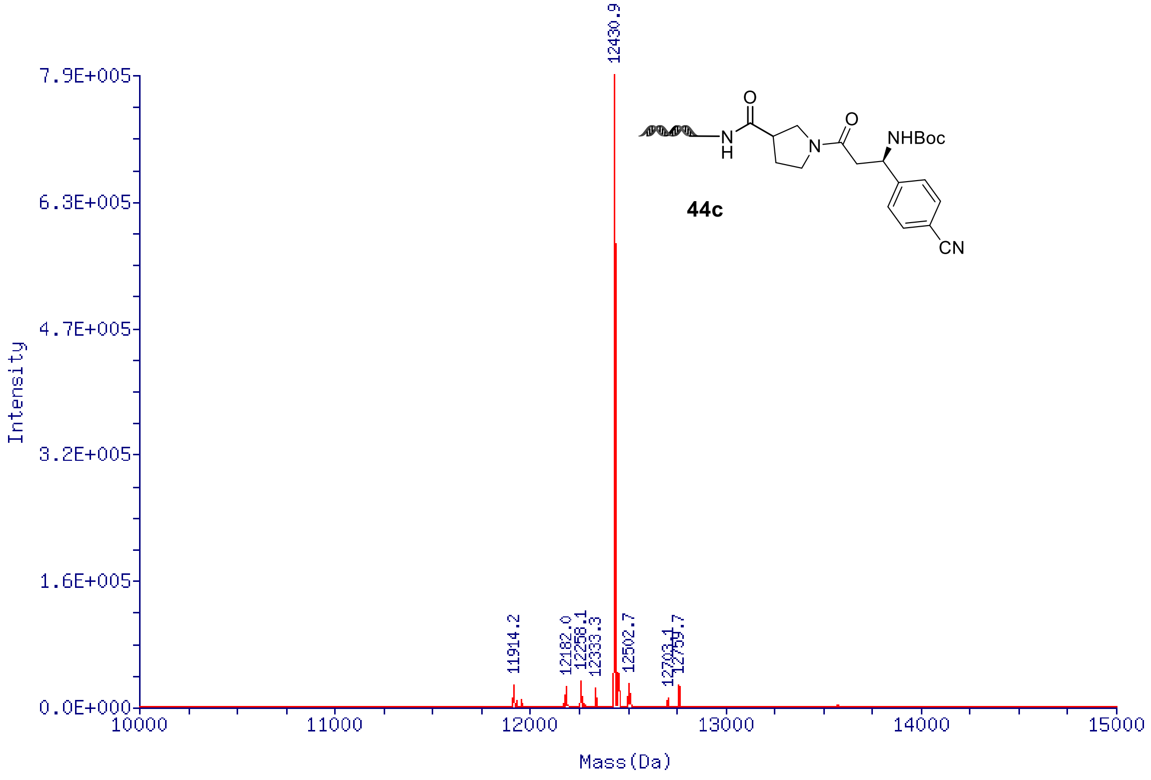
12430.9
Intensity
Mass(Da)
11914.2
12182.0
12258.1
12333.3
12502.7
12703.1
12759.7
NHBoc
CN
44c

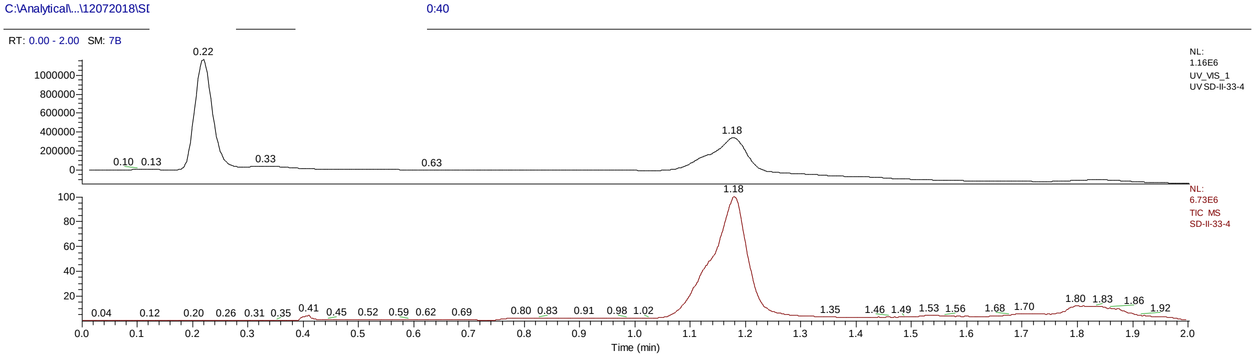
C:\Analytical\...\12072018\SI
0:40
RT: 0.00 - 2.00 SM: 7B
NL: 1.16E6
UV_VIS_1
UV SD-II-33-4
NL: 6.73E6
TIC MS
SD-II-33-4
Time (min)

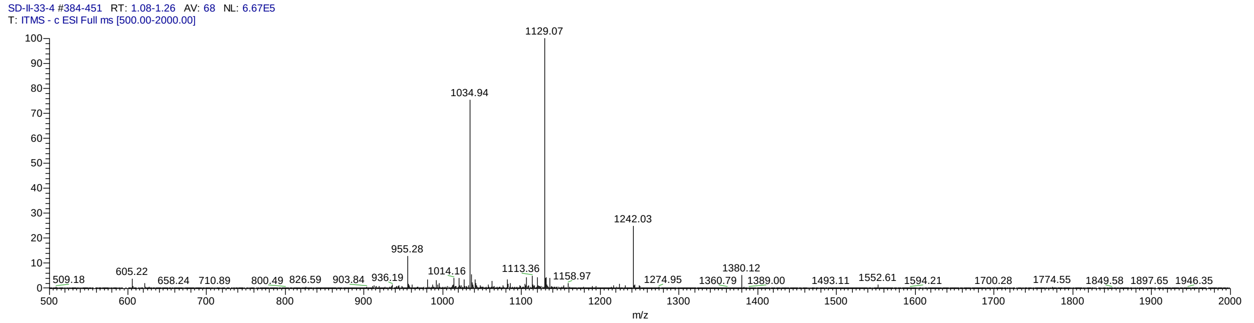
SD-II-33-4 #384-451 RT: 1.08-1.26 AV: 68 NL: 6.67E5
T: ITMS - c ESI Full ms [500.00-2000.00]
1129.07
1034.94
1242.03
955.28
m/z

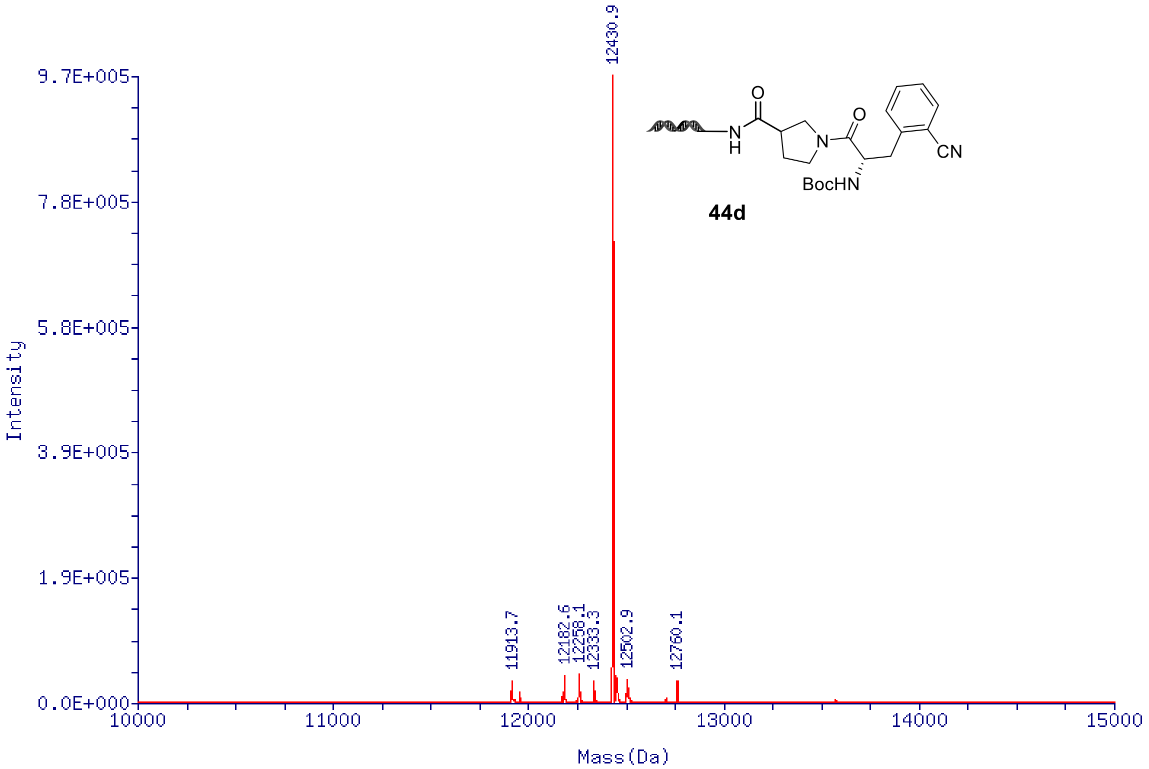
12430.9
11913.7
12182.6
12258.1
12333.3
12502.9
12760.1
Intensity
Mass(Da)
BocHN
CN

44d

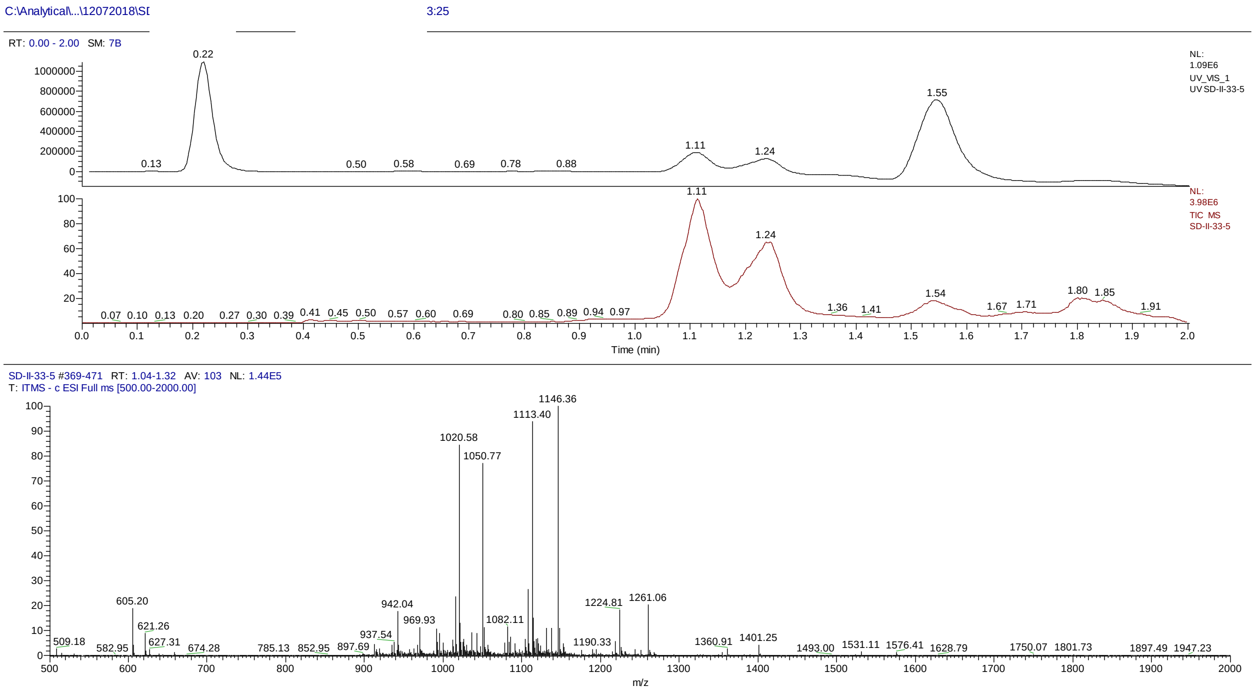
C:\Analytical\...\12072018\SI
3:25
RT: 0.00 - 2.00 SM: 7B
NL: 1.09E6 UV_VIS_1 UV SD-II-33-5
NL: 3.98E6 TIC MS SD-II-33-5
Time (min)
SD-II-33-5 #369-471 RT: 1.04-1.32 AV: 103 NL: 1.44E5
T: ITMS - c ESI Full ms [500.00-2000.00]
1146.36
1113.40
1020.58
1050.77
605.20
1261.06
1224.81
942.04
m/z

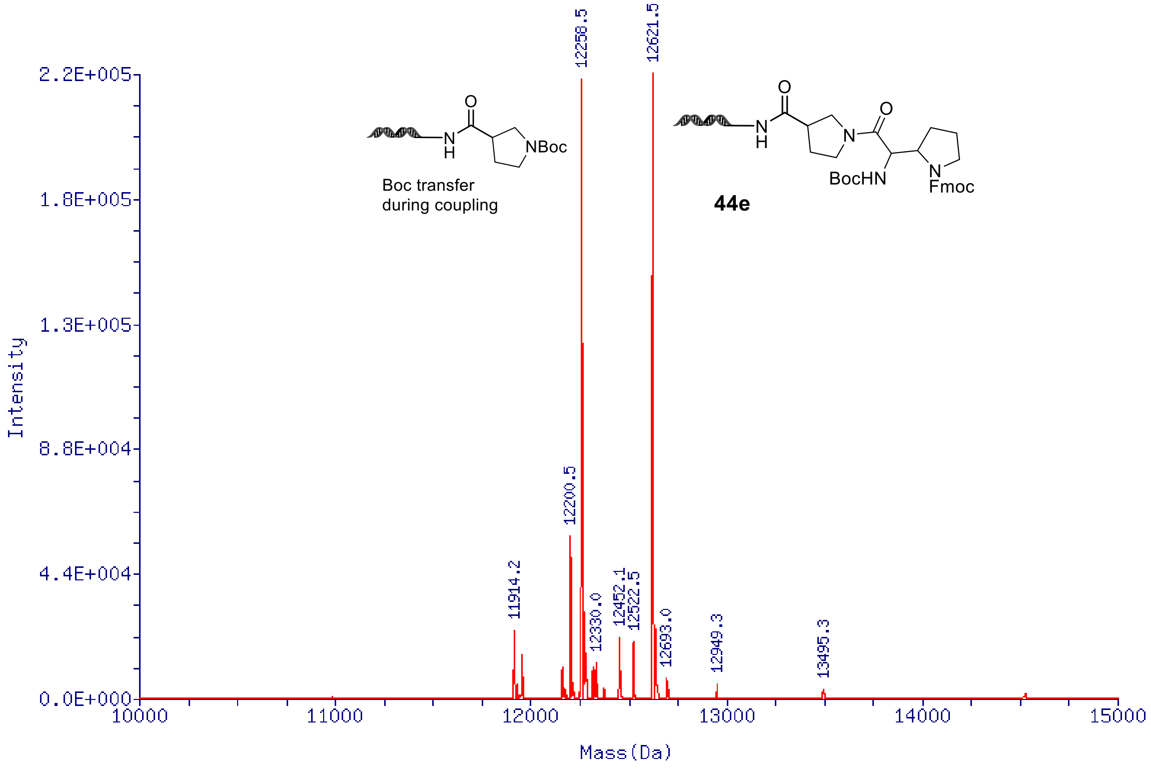
12258.5
12621.5
Boc transfer during coupling
NBoc
BocHN
Fmoc
44e
Intensity
Mass(Da)
12200.5
11914.2
12330.0
12452.1
12522.5
12693.0
12949.3
13495.3

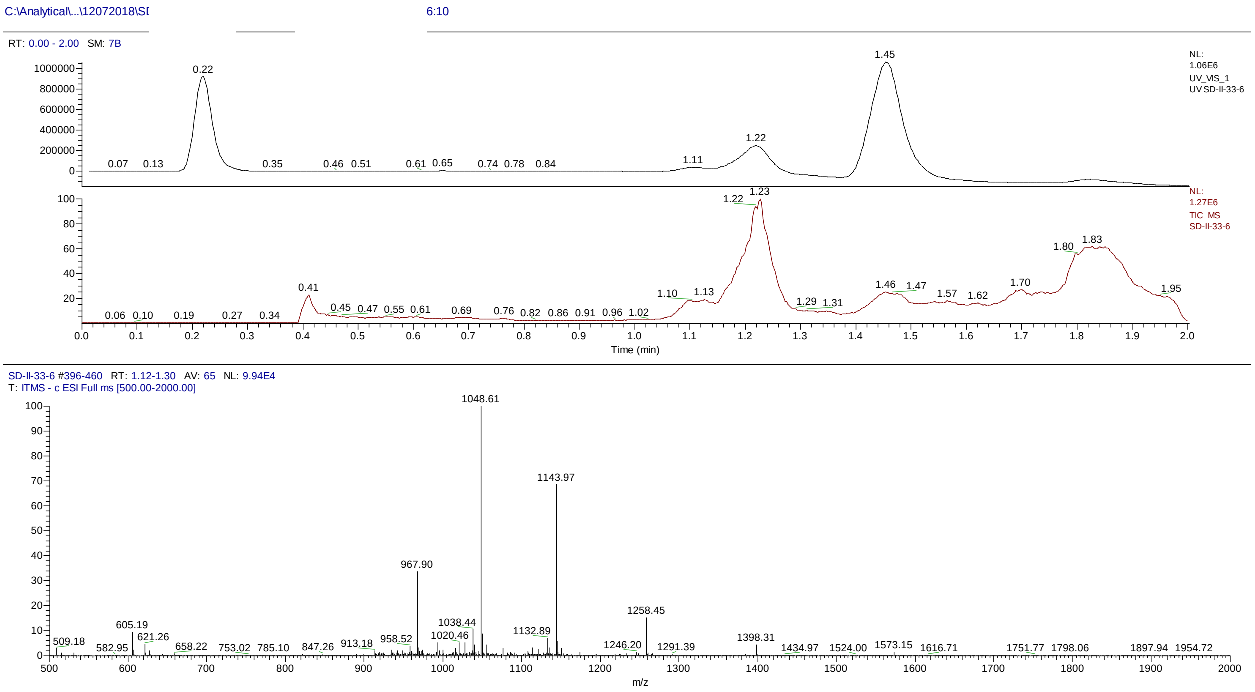

C:\Analytical\...\12072018\SI
6:10
RT: 0.00 - 2.00 SM: 7B
NL: 1.06E6 UV_VIS_1 UV SD-II-33-6
NL: 1.27E6 TIC MS SD-II-33-6
Time (min)
SD-II-33-6 #396-460 RT: 1.12-1.30 AV: 65 NL: 9.94E4
T: ITMS - c ESI Full ms [500.00-2000.00]
1048.61
1143.97
967.90
1258.45
m/z

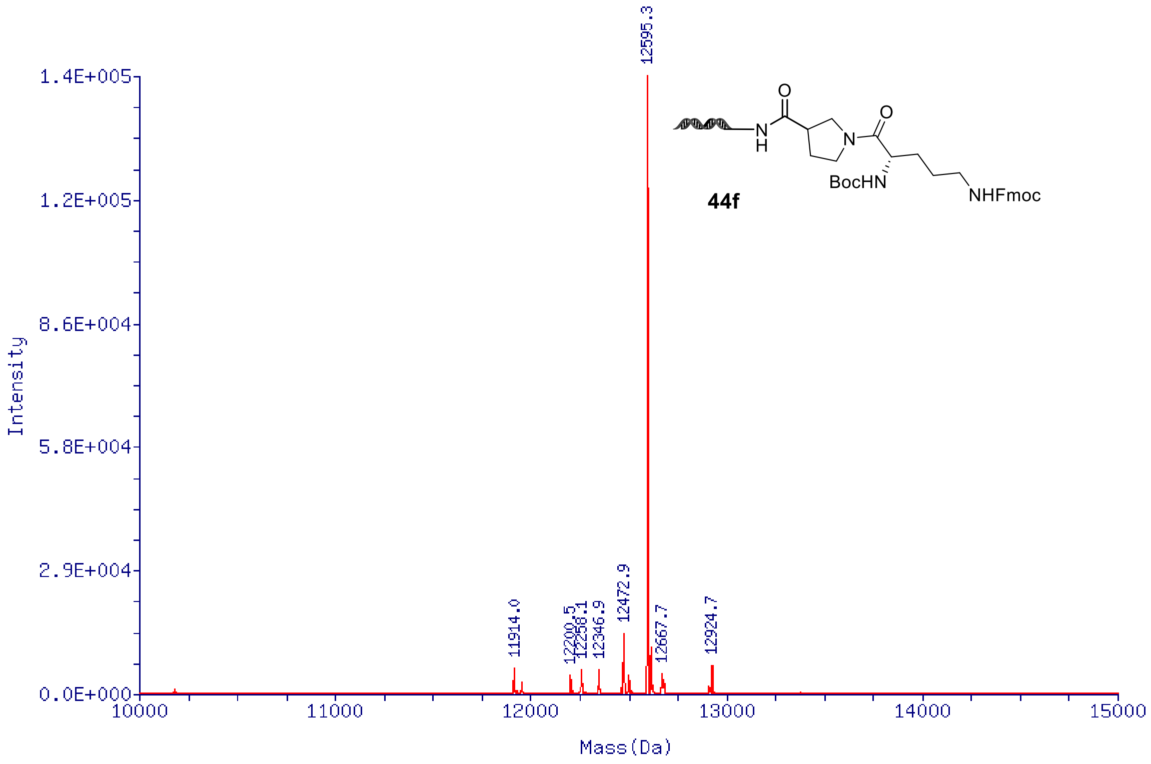

12595.3
Intensity
Mass(Da)
44f
BocHN
NHFmoc

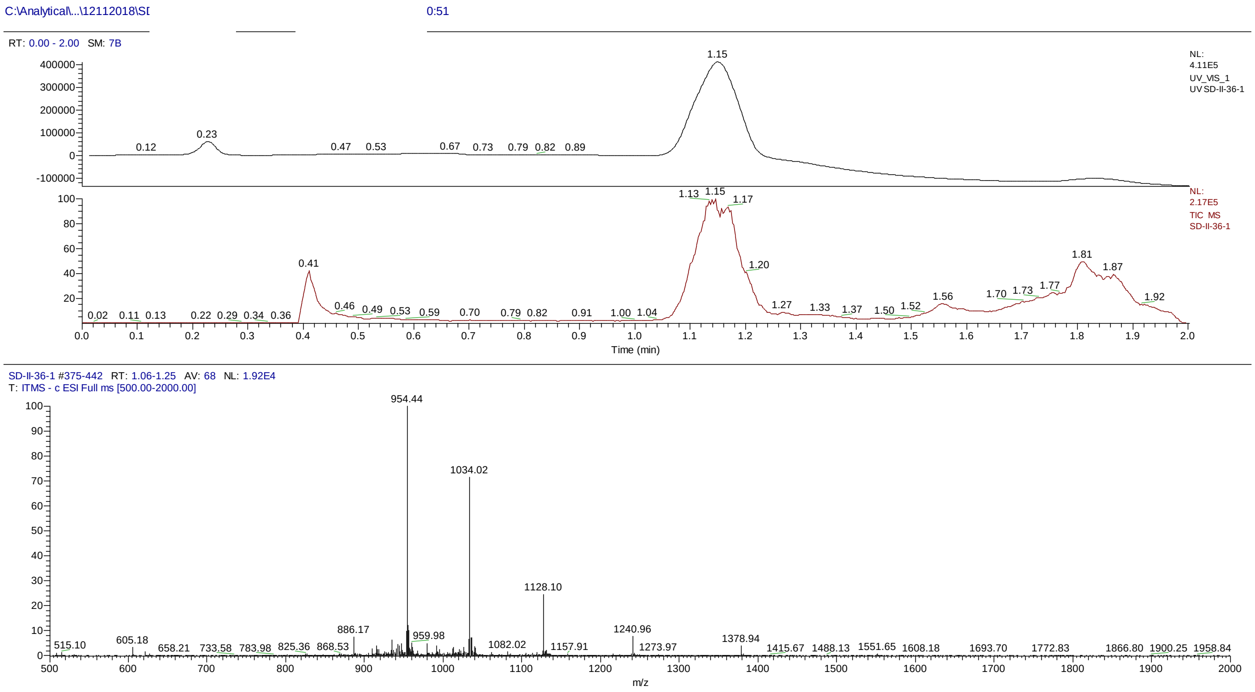
C:\Analytical\...\12112018\SI
0:51
RT: 0.00 - 2.00 SM: 7B
NL: 4.11E5 UV_VIS_1 UV SD-II-36-1
NL: 2.17E5 TIC MS SD-II-36-1
Time (min)
SD-II-36-1 #375-442 RT: 1.06-1.25 AV: 68 NL: 1.92E4
T: ITMS - c ESI Full ms [500.00-2000.00]
954.44
1034.02
1128.10
1240.96
1378.94
m/z

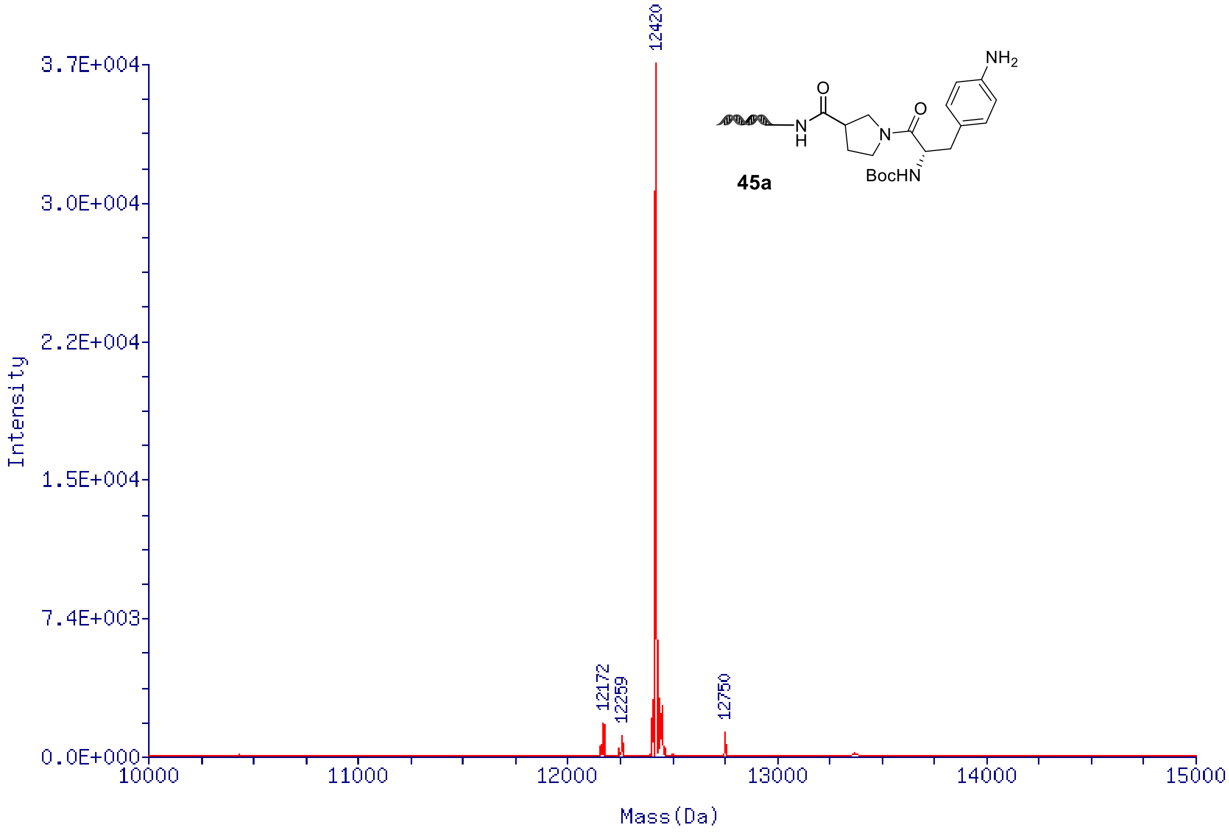
12420
12172
12259
12750
Intensity
Mass(Da)
NH2
O
O
N
H
N
BocHN
45a

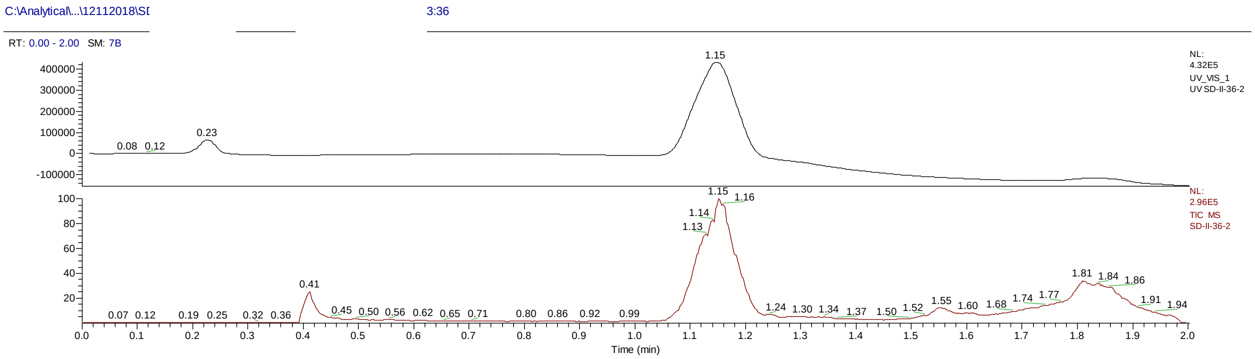
C:\Analytical\...\12112018\SI
3:36
RT: 0.00 - 2.00 SM: 7B
NL: 4.32E5 UV_VIS_1 UV SD-II-36-2
NL: 2.96E5 TIC MS SD-II-36-2
1.15
0.23
0.41
1.81
Time (min)

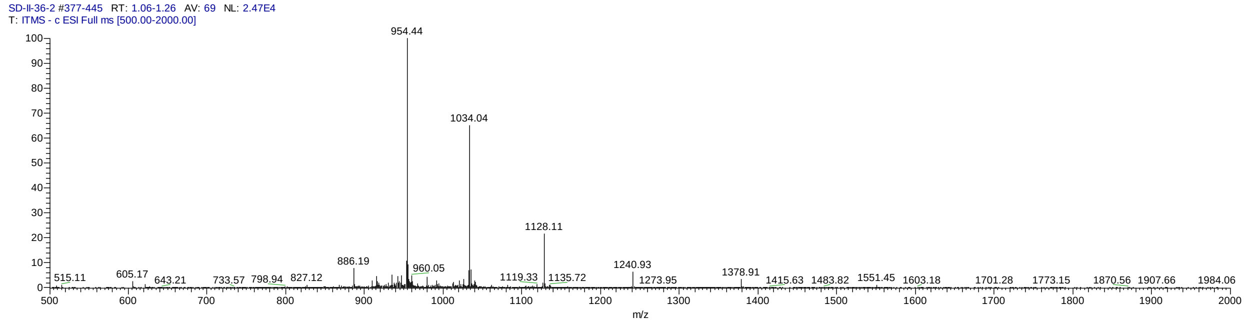
SD-II-36-2 #377-445 RT: 1.06-1.26 AV: 69 NL: 2.47E4
T: ITMS - c ESI Full ms [500.00-2000.00]
954.44
1034.04
1128.11
1240.93
886.19
960.05
m/z

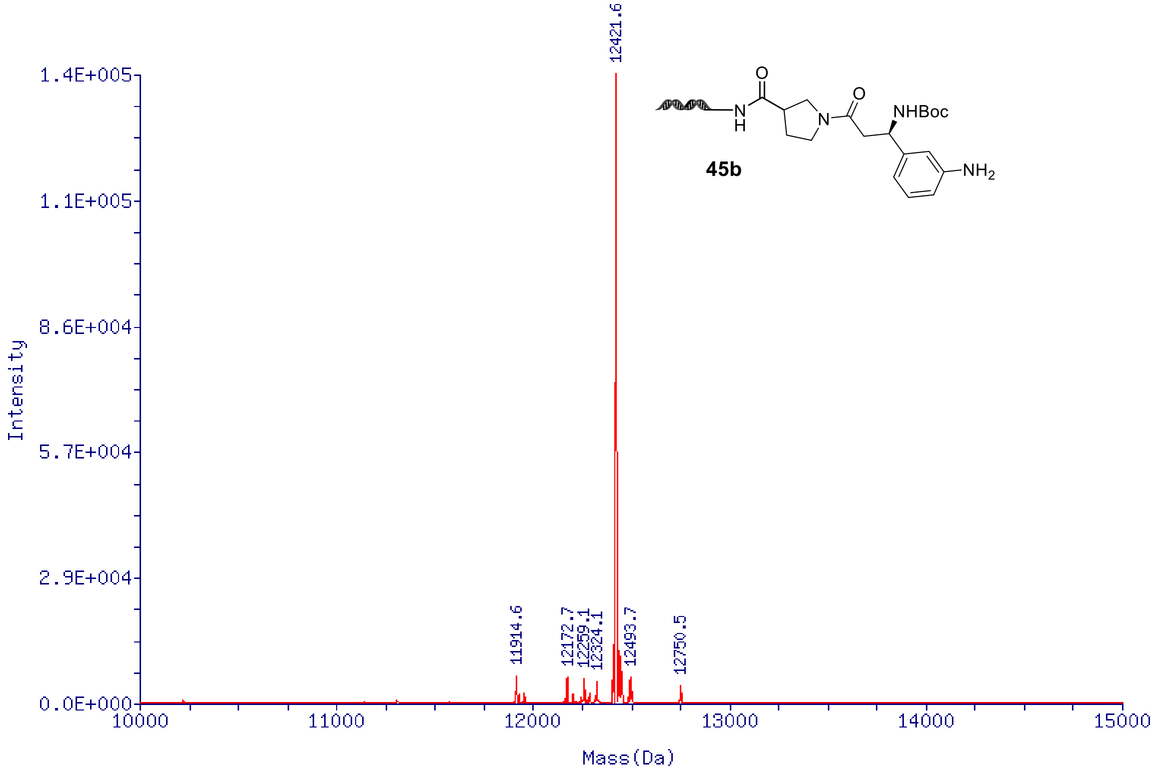
12421.6
11914.6
12172.7
12259.1
12324.1
12493.7
12750.5
Intensity
Mass(Da)
NHBoc
NH2
45b

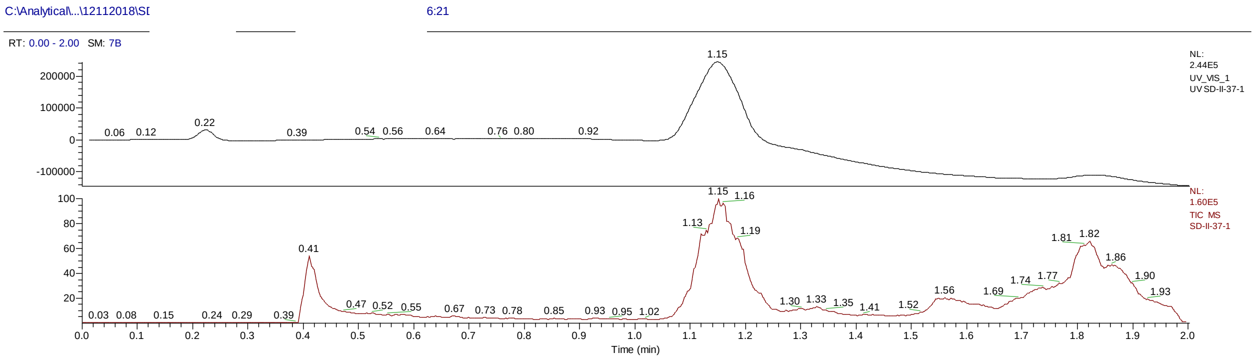
C:\Analytical\...\12112018\SI
6:21
RT: 0.00 - 2.00 SM: 7B
NL: 2.44E5 UV_VIS_1 UV SD-II-37-1
NL: 1.60E5 TIC MS SD-II-37-1
Time (min)

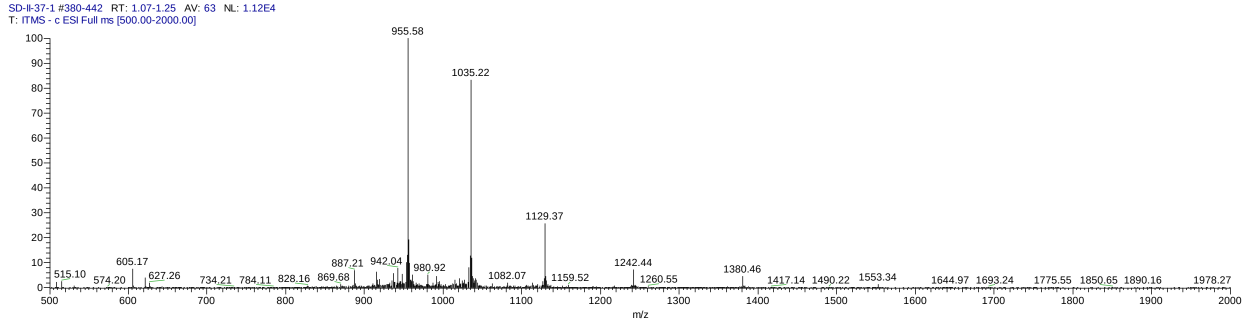
SD-II-37-1 #380-442 RT: 1.07-1.25 AV: 63 NL: 1.12E4
T: ITMS - c ESI Full ms [500.00-2000.00]
955.58
1035.22
1129.37
m/z

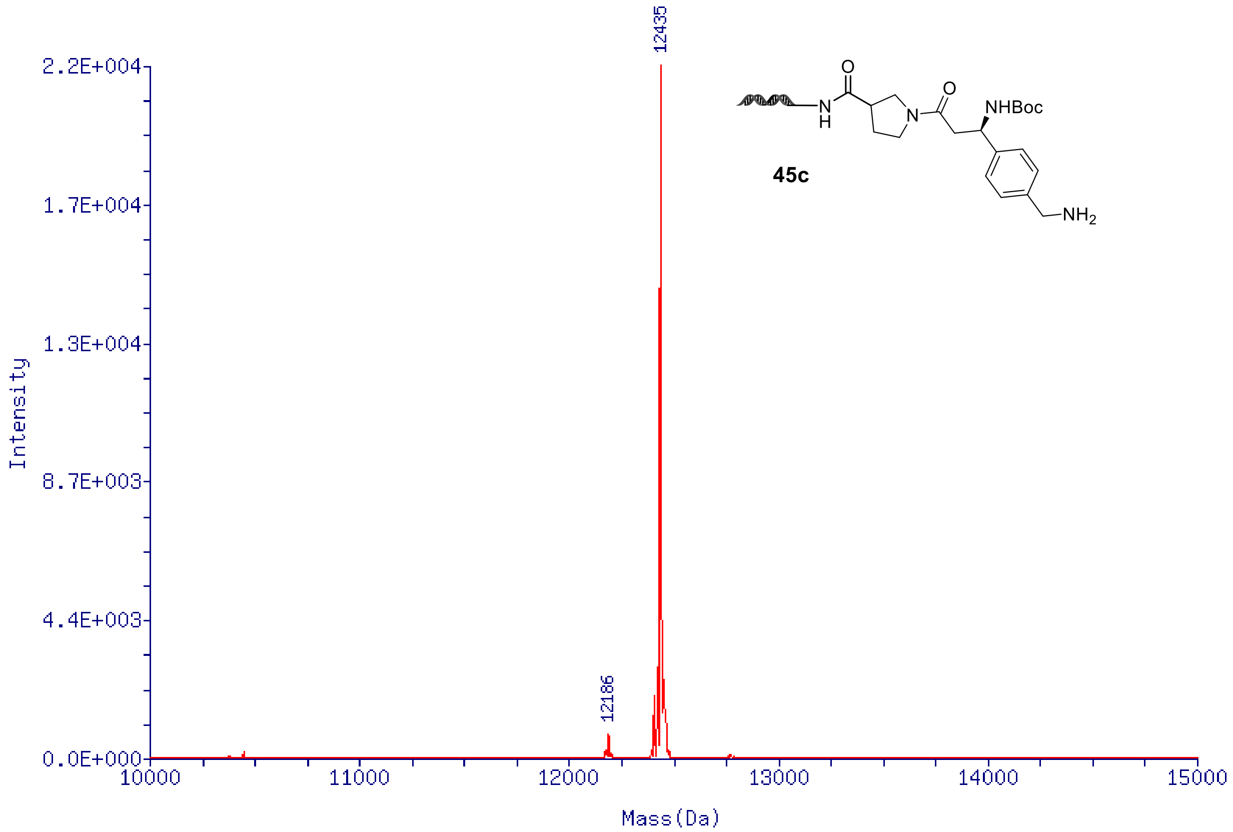
12435
12186
Intensity
Mass(Da)
NHBoc
NH2
45c

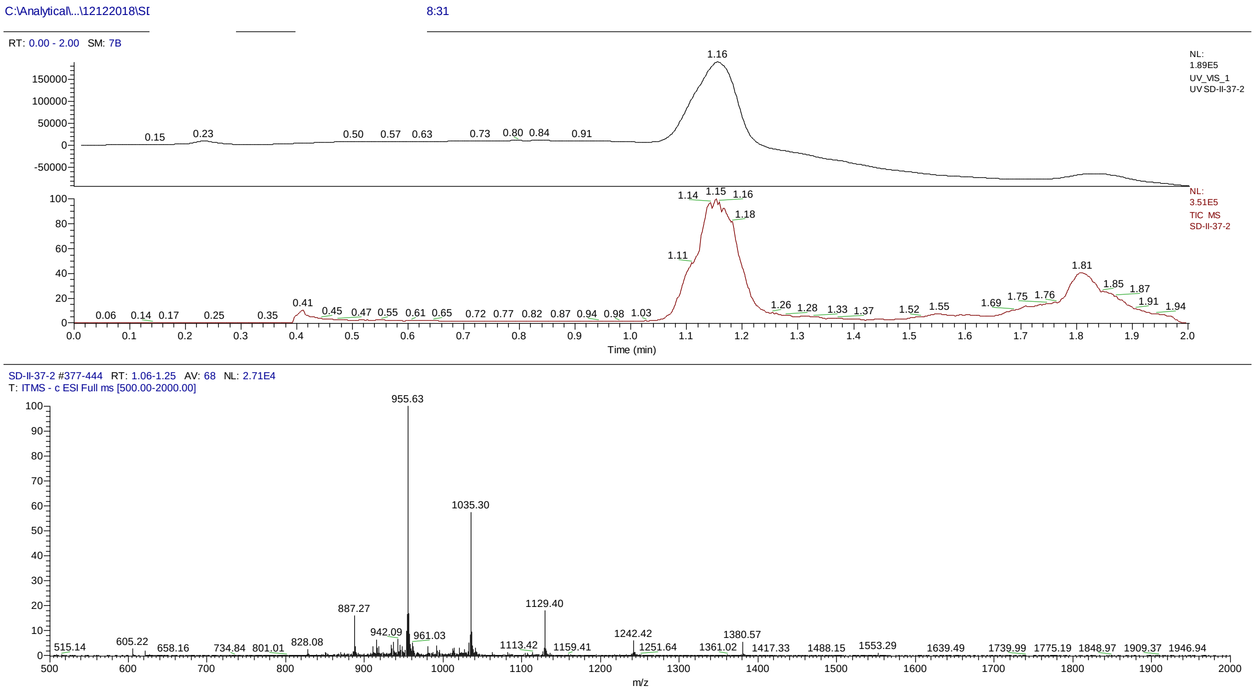
C:\Analytical\...\12122018\SI
8:31
RT: 0.00 - 2.00 SM: 7B
NL: 1.89E5 UV_VIS_1 UV SD-II-37-2
NL: 3.51E5 TIC MS SD-II-37-2
Time (min)
SD-II-37-2 #377-444 RT: 1.06-1.25 AV: 68 NL: 2.71E4
T: ITMS - c ESI Full ms [500.00-2000.00]
955.63
1035.30
1129.40
887.27
1242.42
1380.57
m/z

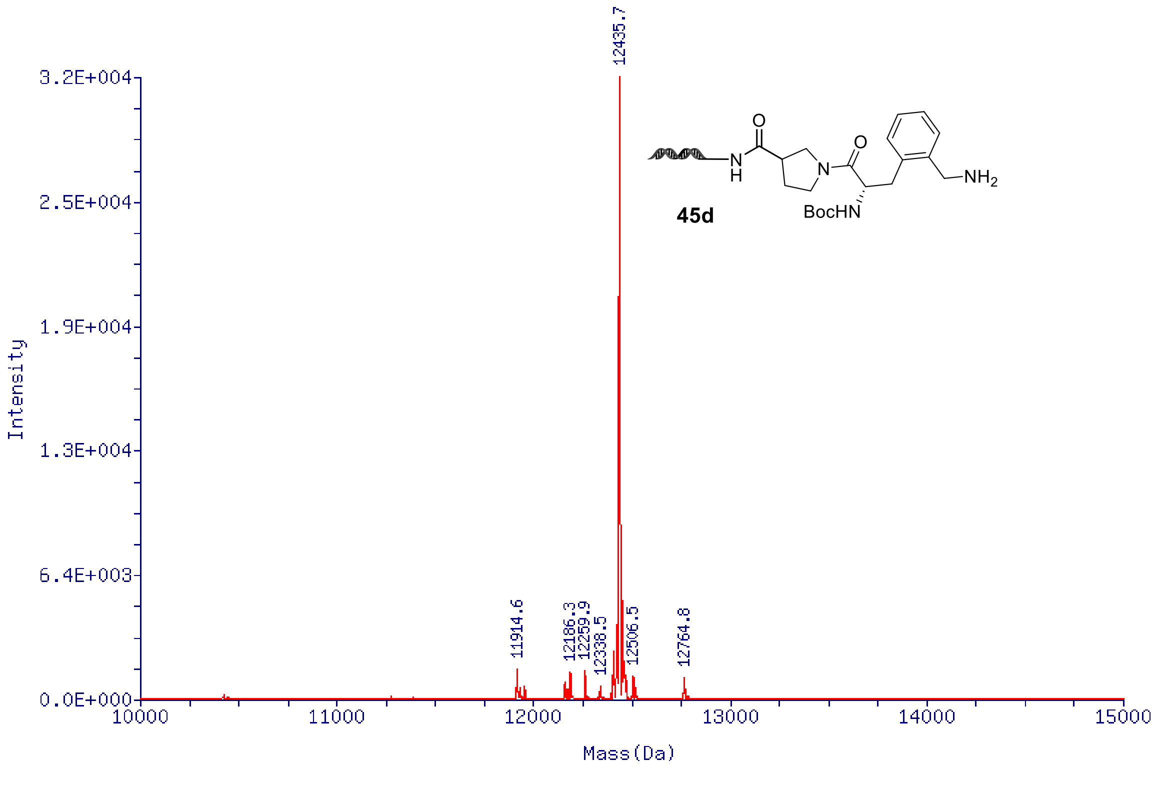
12435.7
11914.6
12186.3
12259.9
12338.5
12506.5
12764.8
Intensity
Mass(Da)
45d
BocHN
NH2

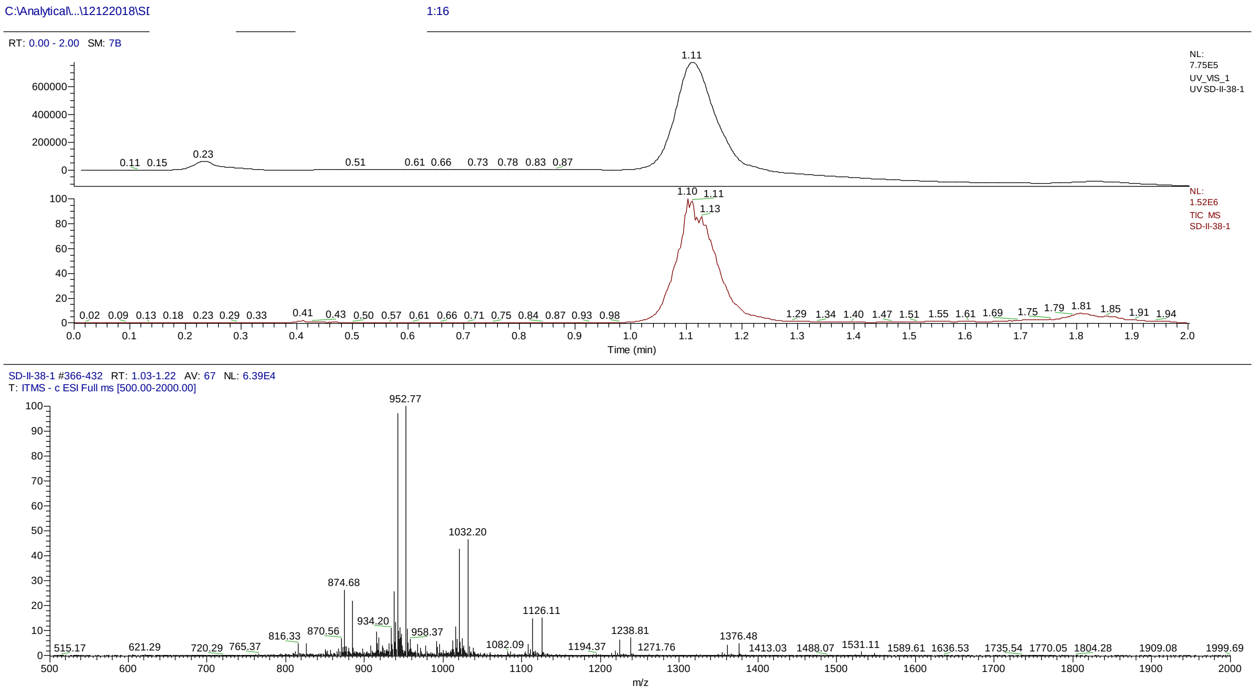
C:\Analytical\...\12122018\SI
1:16
RT: 0.00 - 2.00 SM: 7B
NL: 7.75E5 UV_VIS_1 UV SD-II-38-1
NL: 1.52E6 TIC MS SD-II-38-1
Time (min)
SD-II-38-1 #366-432 RT: 1.03-1.22 AV: 67 NL: 6.39E4
T: ITMS - c ESI Full ms [500.00-2000.00]
952.77
1032.20
874.68
1126.11
1238.81
1376.48
m/z

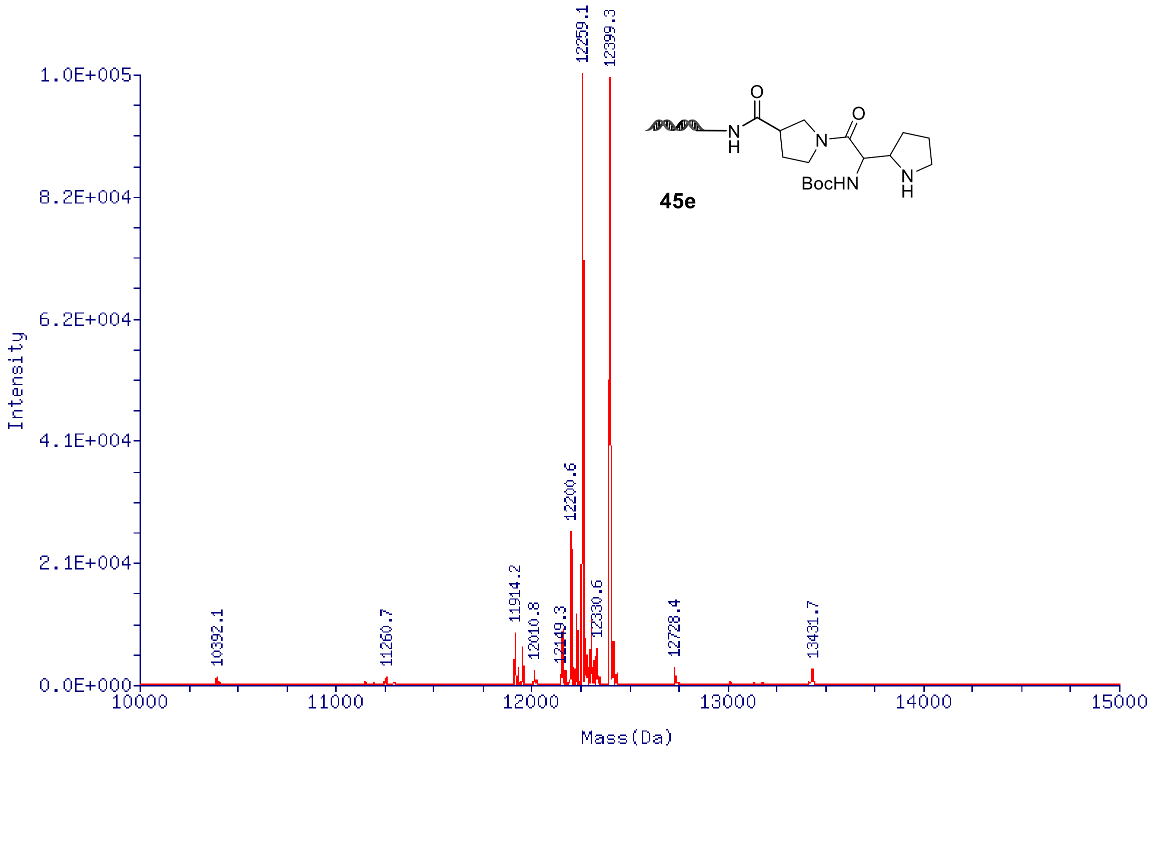
Intensity
Mass(Da)
12259.1
12399.3
12200.6
11914.2
12010.8
12149.3
12330.6
12728.4
13431.7
10392.1
11260.7
BocHN
45e

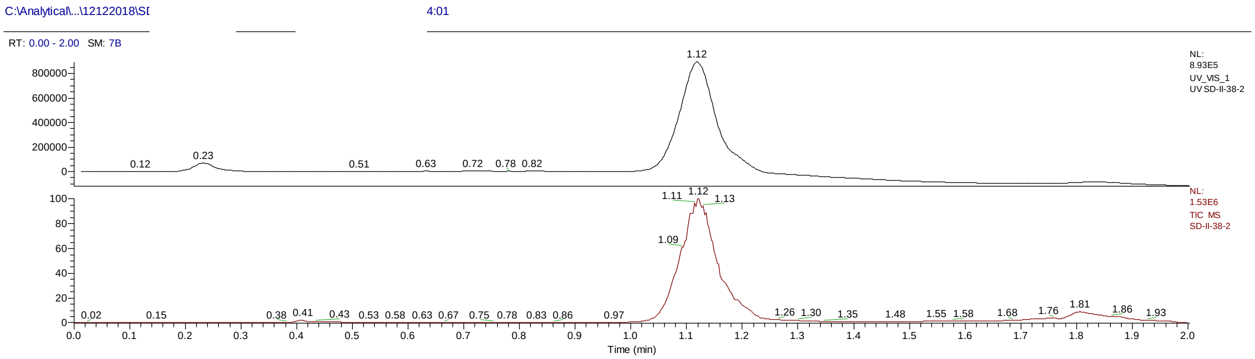
C:\Analytical\...\12122018\SI
4:01
RT: 0.00 - 2.00 SM: 7B
NL: 8.93E5 UV_VIS_1 UV SD-II-38-2
NL: 1.53E6 TIC MS SD-II-38-2
Time (min)

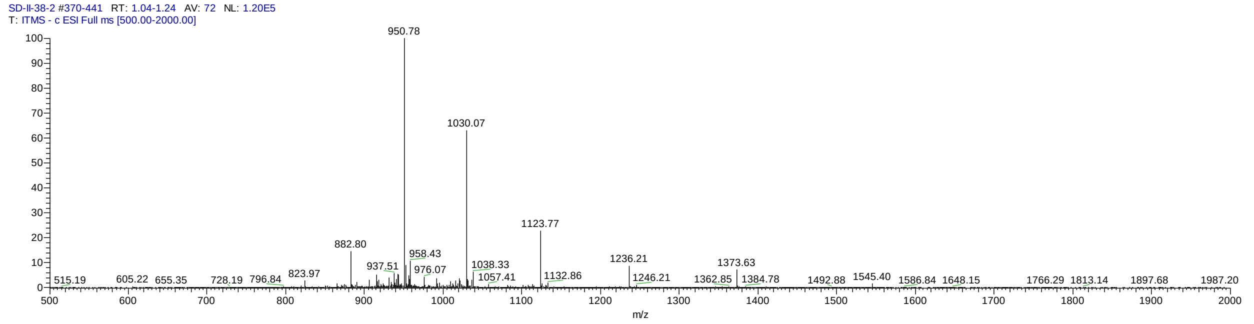
SD-II-38-2 #370-441 RT: 1.04-1.24 AV: 72 NL: 1.20E5
T: ITMS - c ESI Full ms [500.00-2000.00]
950.78
1030.07
1123.77
882.80
1236.21
1373.63
m/z

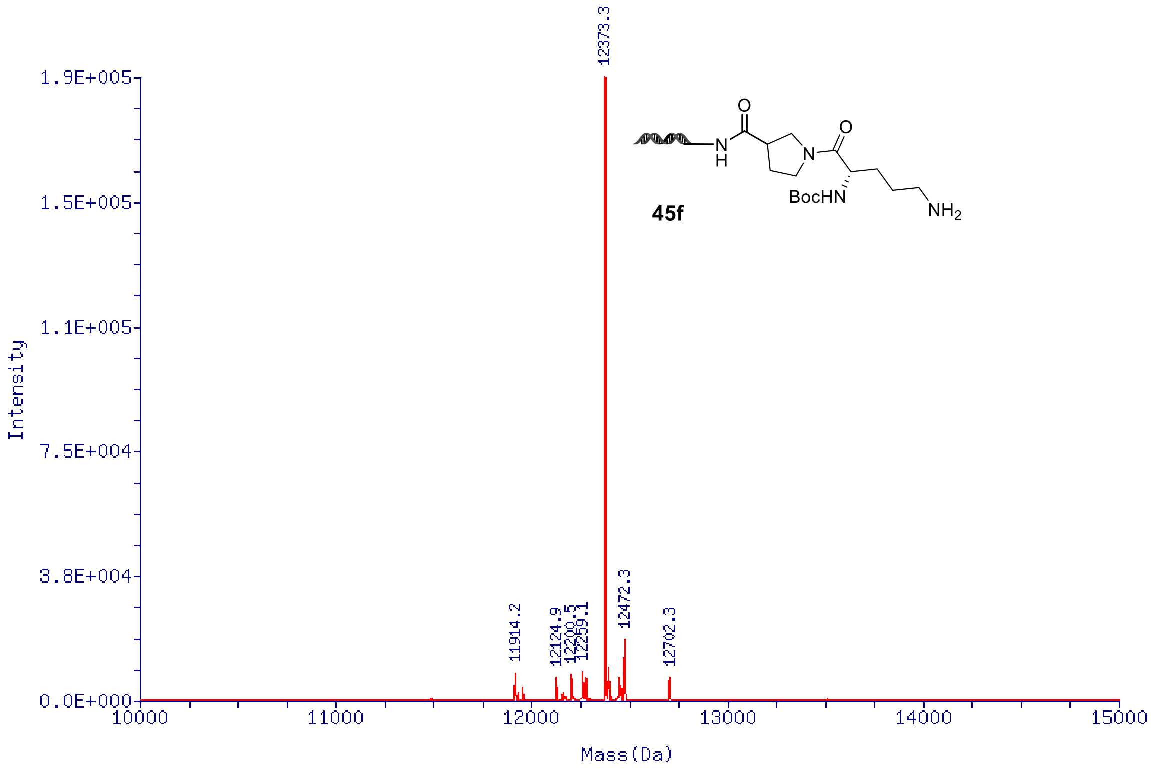
12373.3
11914.2
12124.9
12200.5
12259.1
12472.3
12702.3
Intensity
Mass(Da)
45f
BocHN
NH2

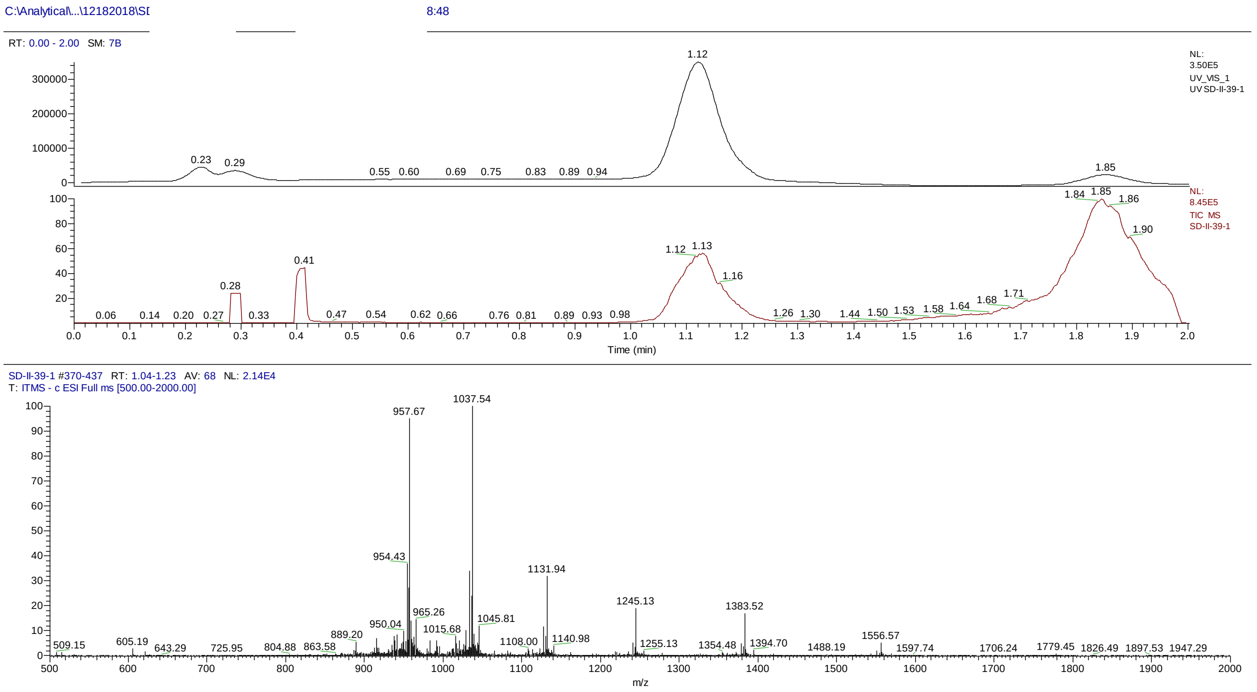
C:\Analytical\...\12182018\SI
8:48
RT: 0.00 - 2.00 SM: 7B
NL: 3.50E5 UV_VIS_1 UV SD-II-39-1
NL: 8.45E5 TIC MS SD-II-39-1
Time (min)
SD-II-39-1 #370-437 RT: 1.04-1.23 AV: 68 NL: 2.14E4
T: ITMS - c ESI Full ms [500.00-2000.00]
957.67
1037.54
954.43
1131.94
1245.13
1383.52
m/z

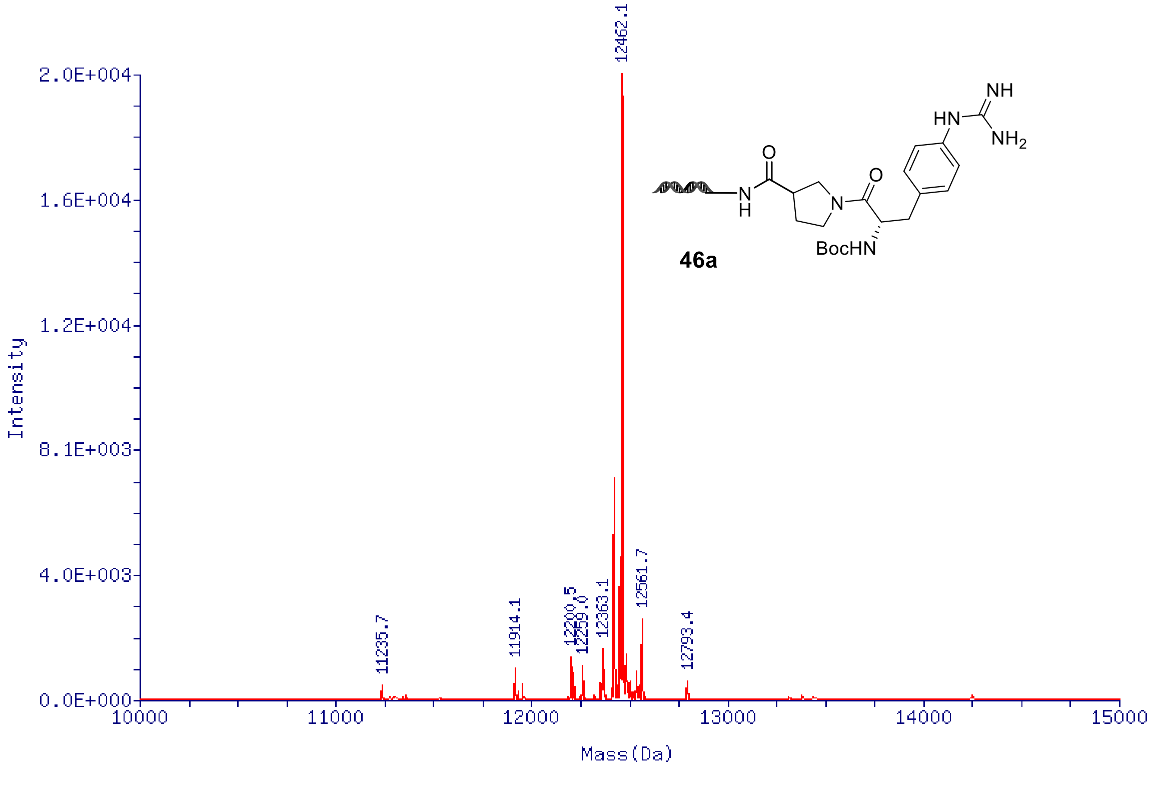
Intensity
Mass(Da)
12462.1
11235.7
11914.1
12200.5
12363.1
12561.7
12793.4
NH
HN
NH2
O
O
N
H
N
BocHN

46a

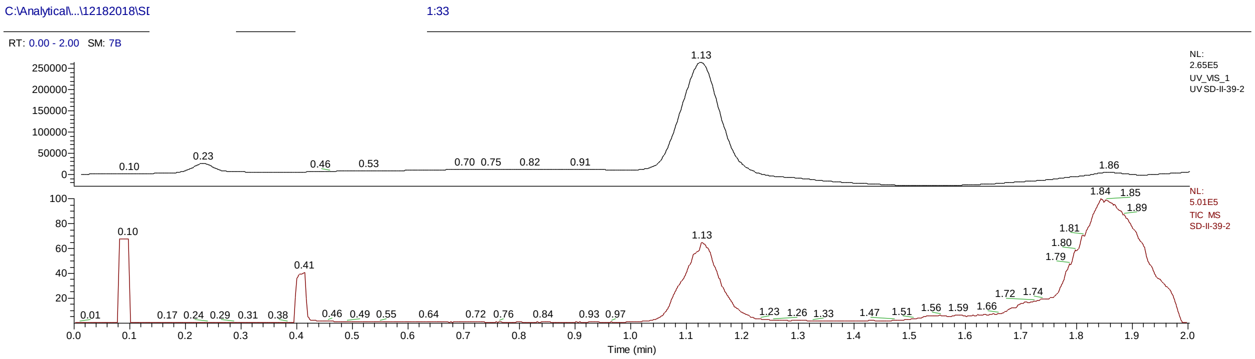
C:\Analytical\...\12182018\SI
1:33
RT: 0.00 - 2.00 SM: 7B
NL: 2.65E5 UV_VIS_1 UV SD-II-39-2
NL: 5.01E5 TIC MS SD-II-39-2
Time (min)

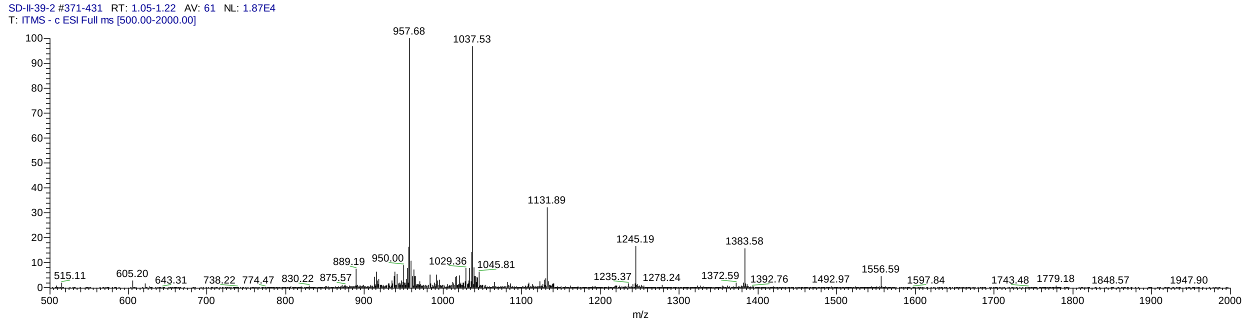
SD-II-39-2 #371-431 RT: 1.05-1.22 AV: 61 NL: 1.87E4
T: ITMS - c ESI Full ms [500.00-2000.00]
957.68
1037.53
1131.89
1245.19
1383.58
m/z

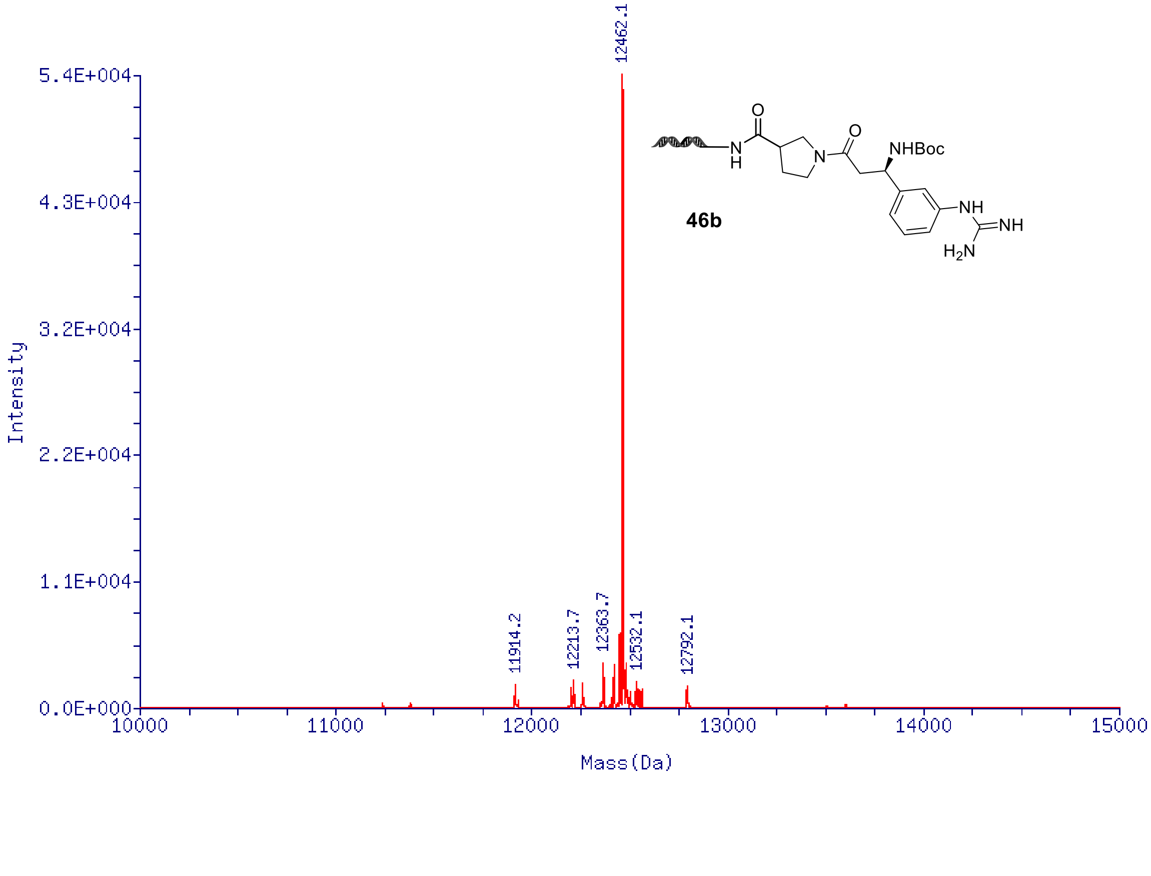
12462.1
11914.2
12213.7
12363.7
12532.1
12792.1
Intensity
Mass(Da)
NHBoc
46b

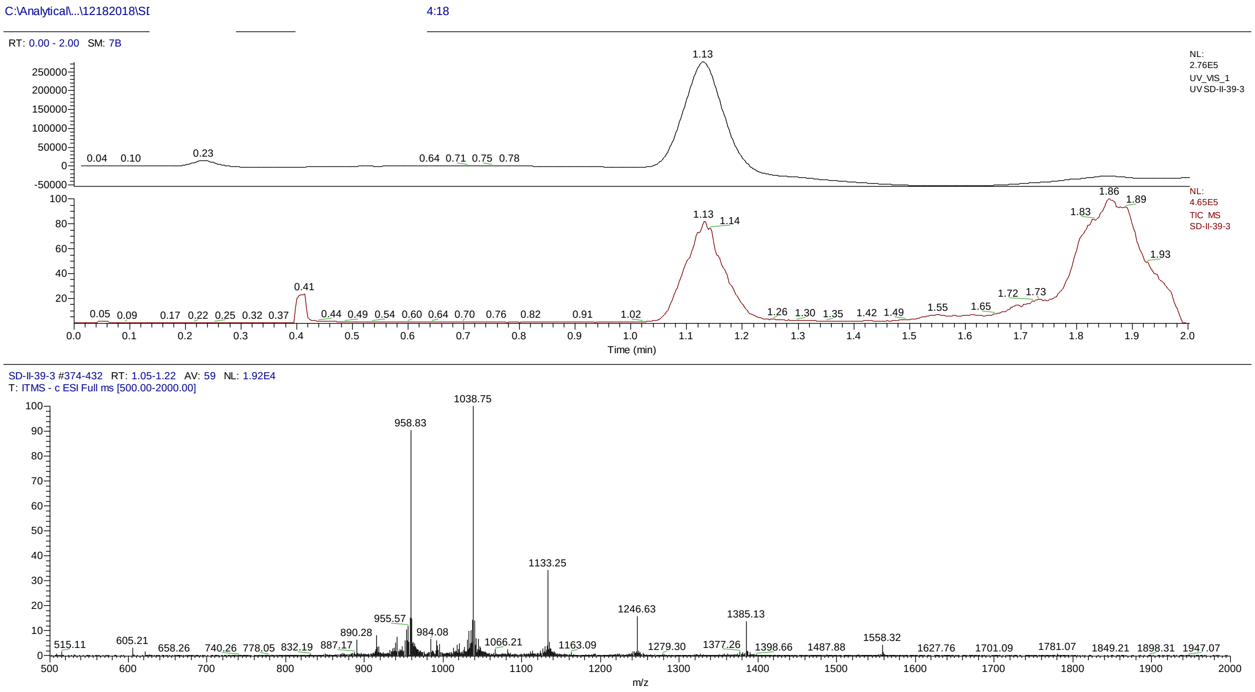
C:\Analytical\...\12182018\SI
4:18
RT: 0.00 - 2.00 SM: 7B
NL: 2.76E5 UV_VIS_1 UV SD-II-39-3
NL: 4.65E5 TIC MS SD-II-39-3
Time (min)
SD-II-39-3 #374-432 RT: 1.05-1.22 AV: 59 NL: 1.92E4
T: ITMS - c ESI Full ms [500.00-2000.00]
958.83
1038.75
1133.25
1246.63
1385.13
m/z

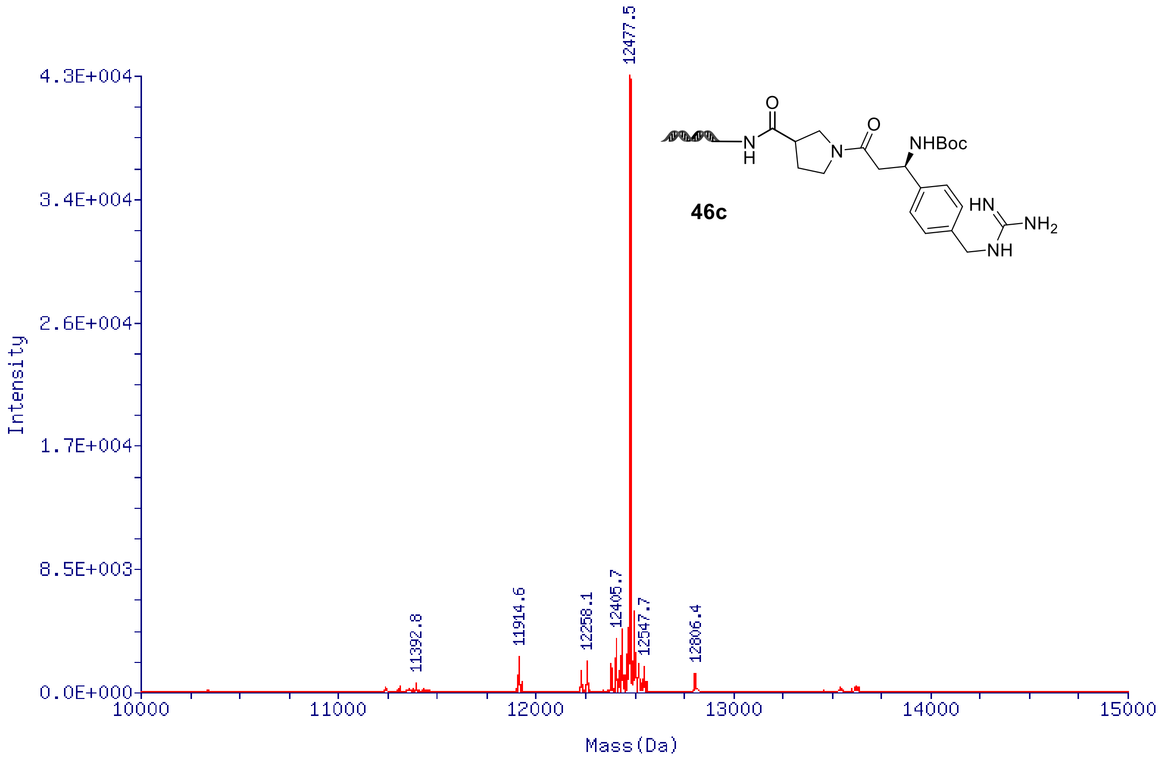
12477.5
11392.8
11914.6
12258.1
12405.7
12547.7
12806.4
Intensity
Mass(Da)
NHBoc
46c

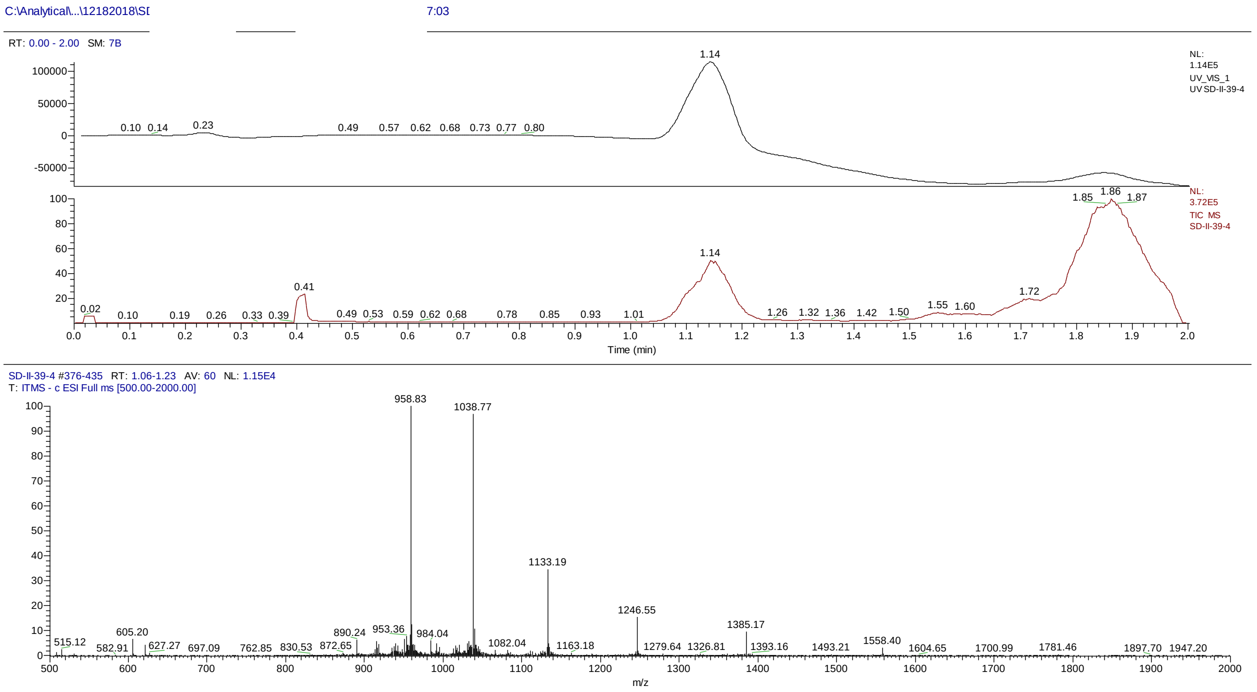
C:\Analytical\...\12182018\SI
7:03
RT: 0.00 - 2.00 SM: 7B
NL: 1.14E5 UV_VIS_1 UV SD-II-39-4
NL: 3.72E5 TIC MS SD-II-39-4
Time (min)
SD-II-39-4 #376-435 RT: 1.06-1.23 AV: 60 NL: 1.15E4
T: ITMS - c ESI Full ms [500.00-2000.00]
958.83
1038.77
1133.19
1246.55
1385.17
m/z

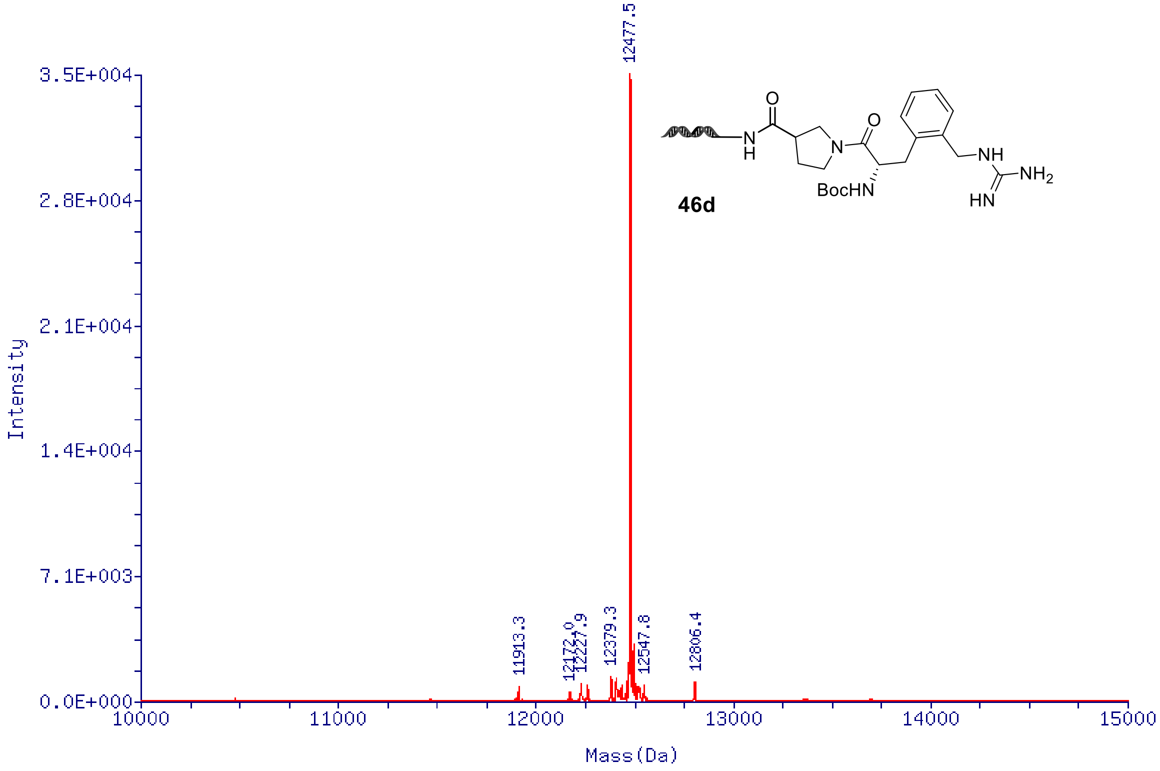
12477.5
Intensity
Mass(Da)
11913.3
12379.3
12547.8
12806.4
BocHN
HN
NH
NH2

46d

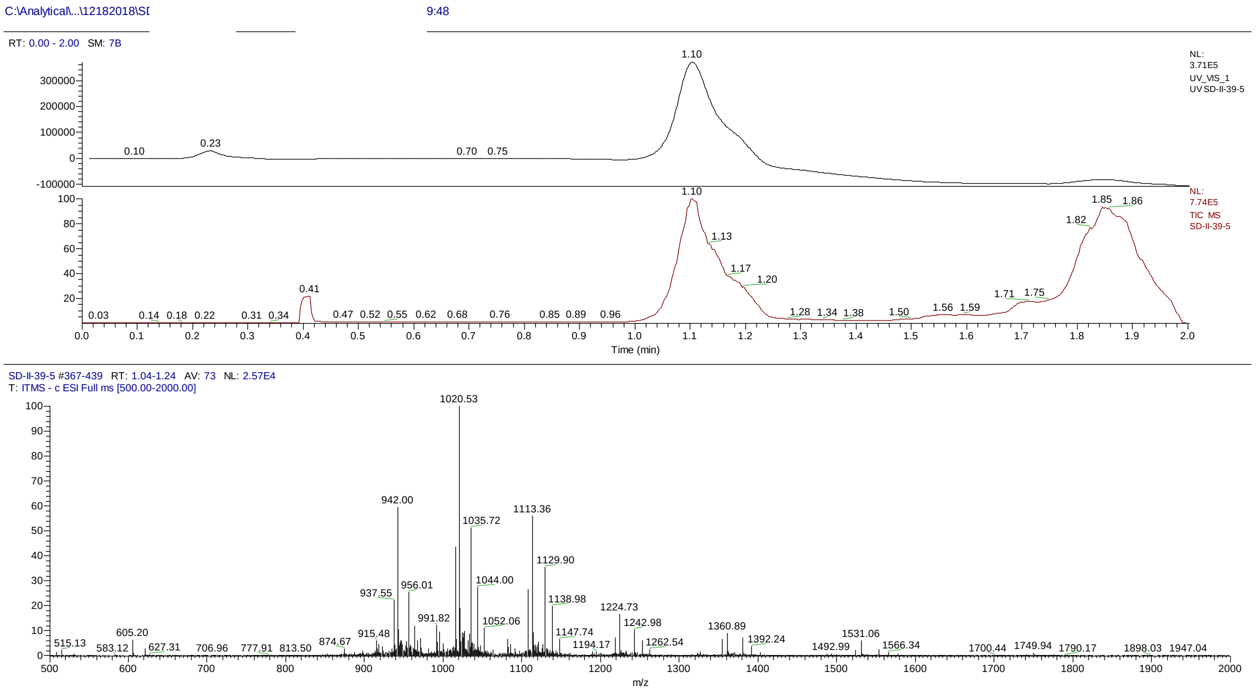
C:\Analytical\...\12182018\SI
9:48
RT: 0.00 - 2.00 SM: 7B
NL: 3.71E5
UV_VIS_1
UV SD-II-39-5
NL: 7.74E5
TIC MS
SD-II-39-5
Time (min)
SD-II-39-5 #367-439 RT: 1.04-1.24 AV: 73 NL: 2.57E4
T: ITMS - c ESI Full ms [500.00-2000.00]
1020.53
942.00
1113.36
1035.72
1129.90
1044.00
m/z

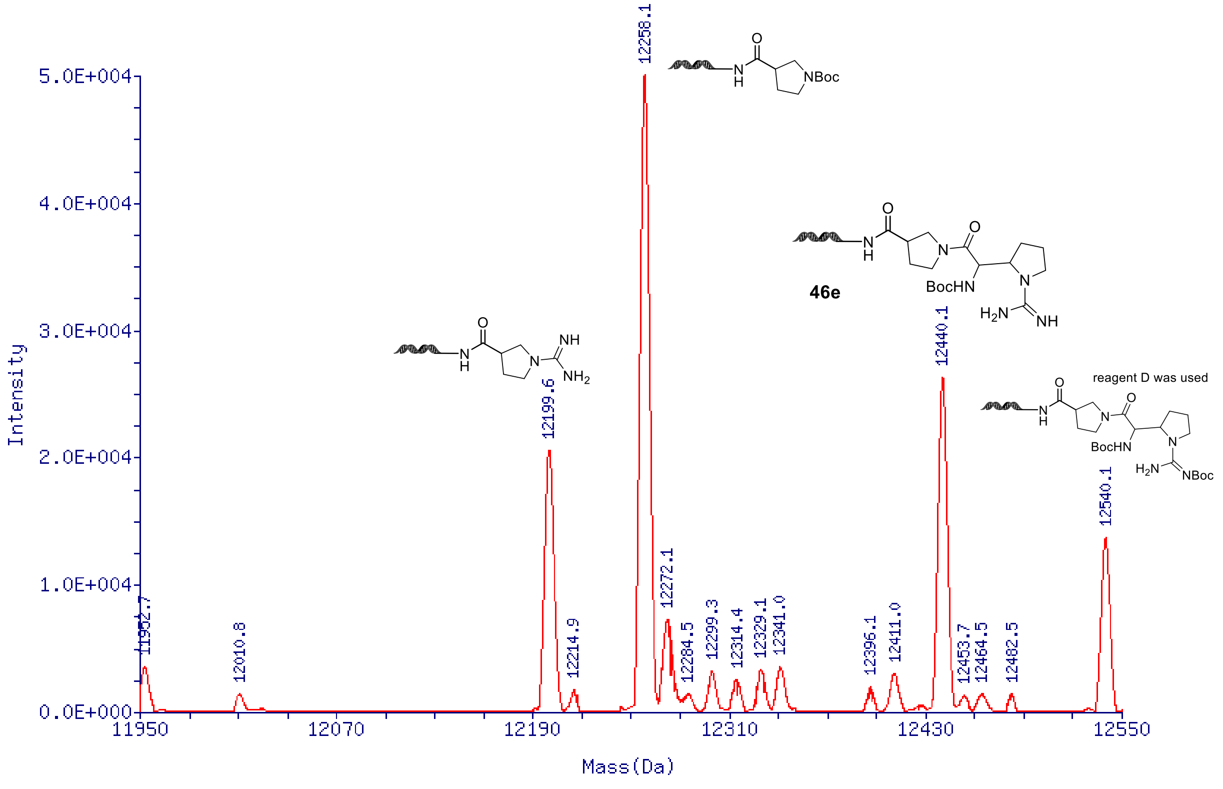
Intensity
Mass(Da)
12258.1
12199.6
12440.1
12540.1
46e
BocHN
NBoc
reagent D was used

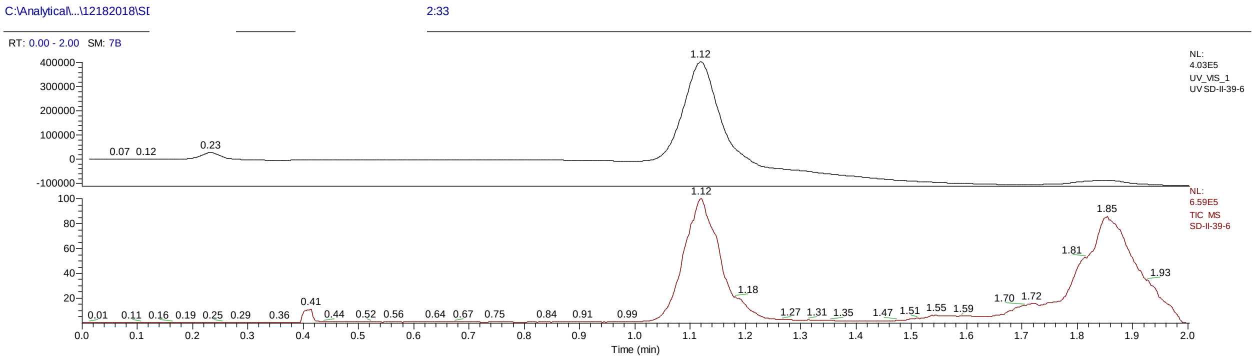
C:\Analytical\...\12182018\SI
2:33
RT: 0.00 - 2.00 SM: 7B
NL: 4.03E5 UV_VIS_1 UV SD-II-39-6
NL: 6.59E5 TIC MS SD-II-39-6
Time (min)

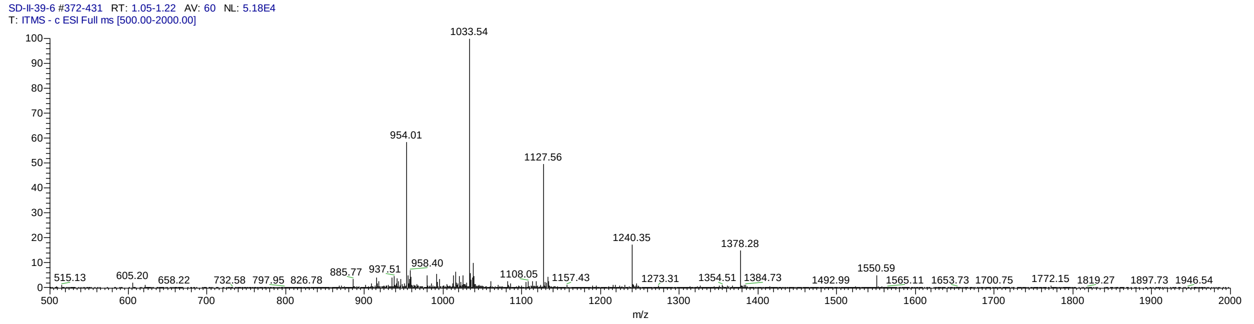
SD-II-39-6 #372-431 RT: 1.05-1.22 AV: 60 NL: 5.18E4
T: ITMS - c ESI Full ms [500.00-2000.00]
1033.54
954.01
1127.56
1240.35
1378.28
m/z

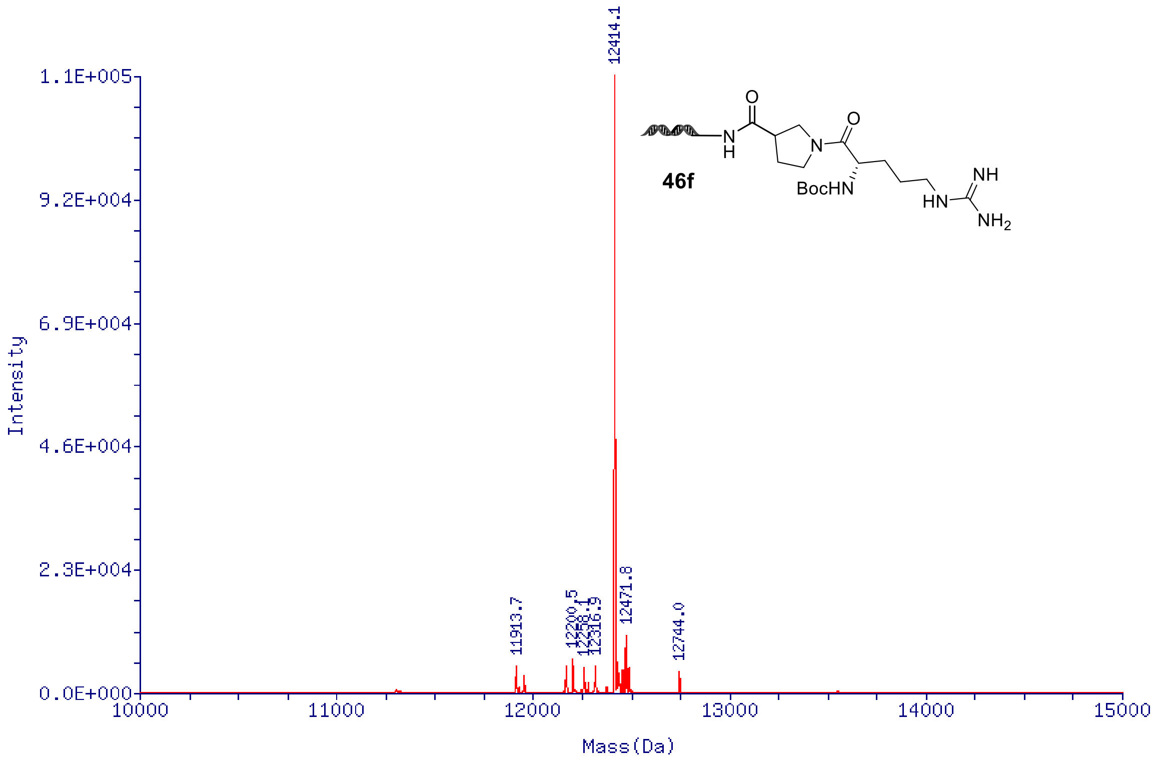
12414.1
11913.7
12471.8
12744.0
Intensity
Mass(Da)
46f
BocHN
NH
HN
NH2

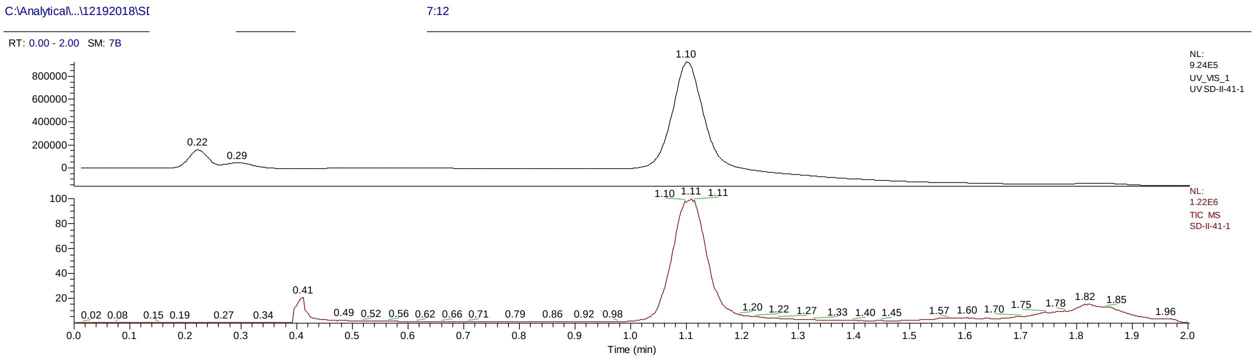
C:\Analytical\...\12192018\SI
7:12
RT: 0.00 - 2.00 SM: 7B
NL: 9.24E5 UV_VIS_1 UV SD-II-41-1
NL: 1.22E6 TIC MS SD-II-41-1
Time (min)

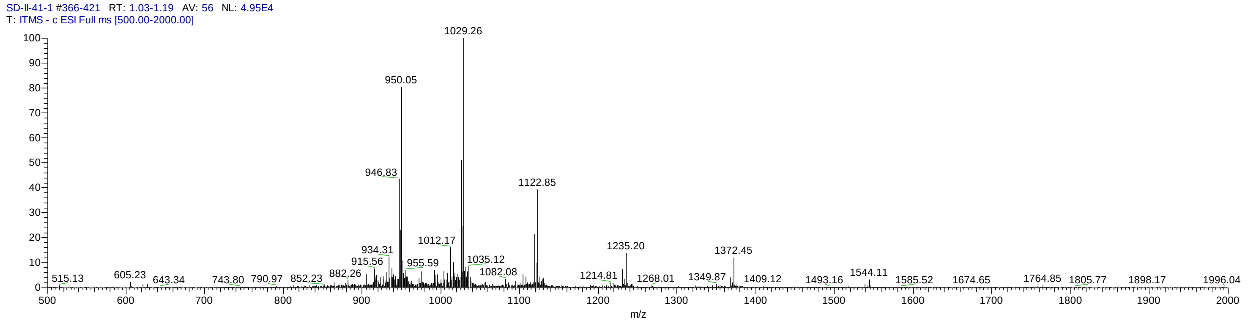
SD-II-41-1 #366-421 RT: 1.03-1.19 AV: 56 NL: 4.95E4
T: ITMS - c ESI Full ms [500.00-2000.00]
1029.26
950.05
946.83
1122.85
1235.20
1372.45
m/z

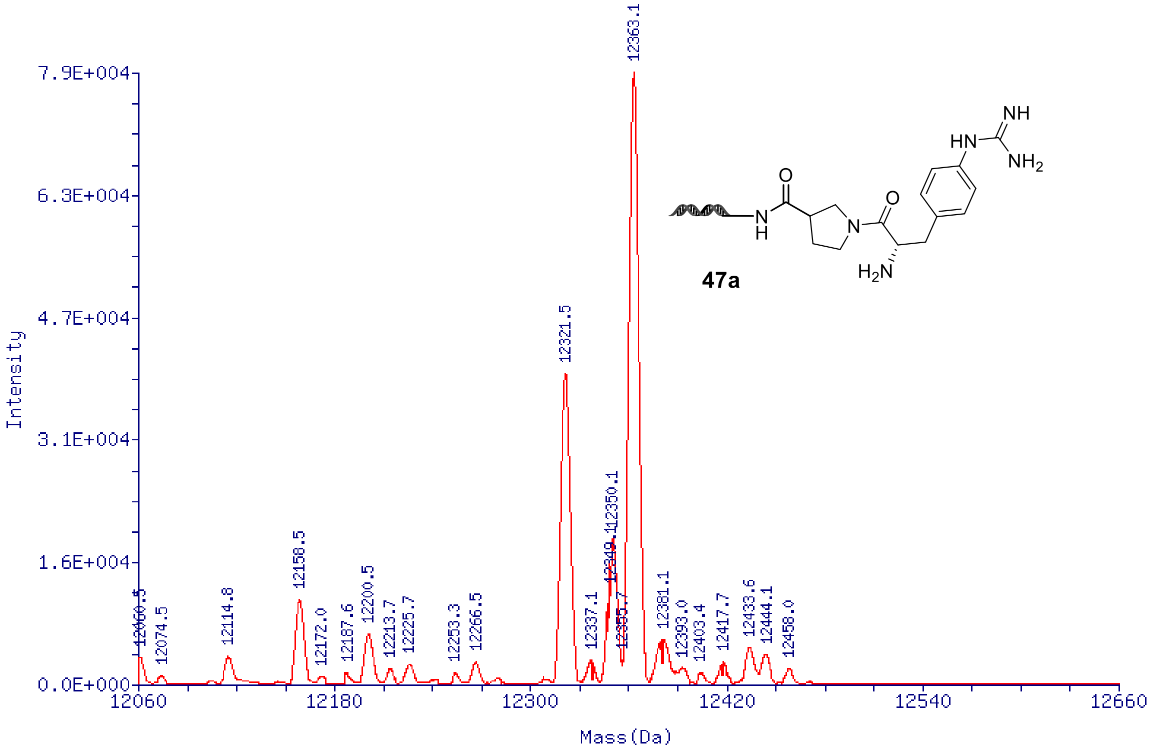
12363.1
12321.5
12350.1
12158.5
12200.5
Intensity
Mass(Da)
NH
HN
NH2
O
O
N
H
N
H2N

47a

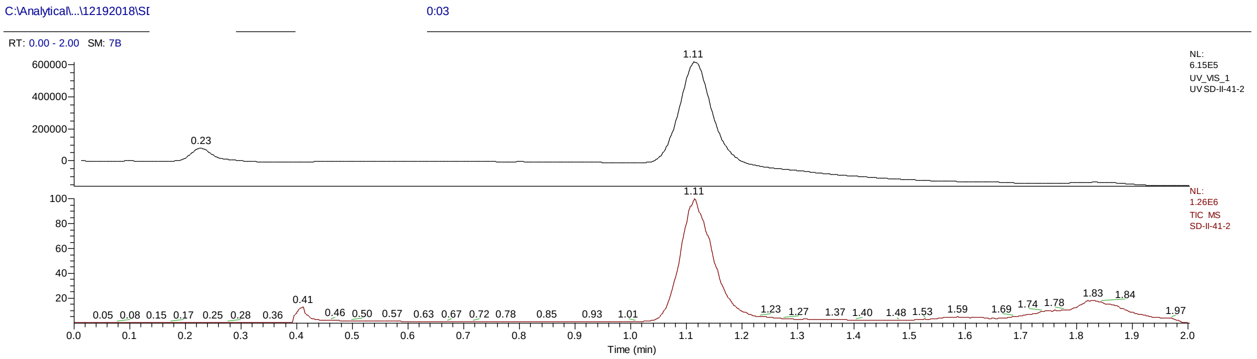
C:\Analytical\...\12192018\SI
0:03
RT: 0.00 - 2.00 SM: 7B
NL: 6.15E5 UV_VIS_1 UV SD-II-41-2
NL: 1.26E6 TIC MS SD-II-41-2
Time (min)

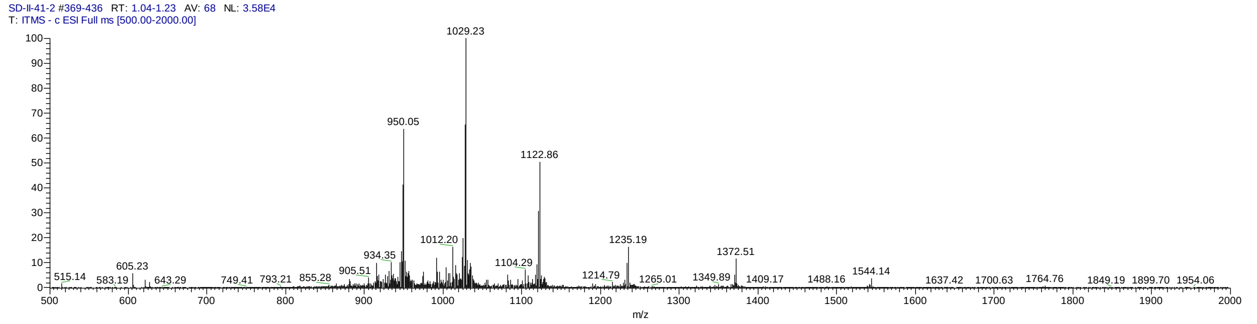
SD-II-41-2 #369-436 RT: 1.04-1.23 AV: 68 NL: 3.58E4
T: ITMS - c ESI Full ms [500.00-2000.00]
1029.23
950.05
1122.86
1235.19
1372.51
m/z

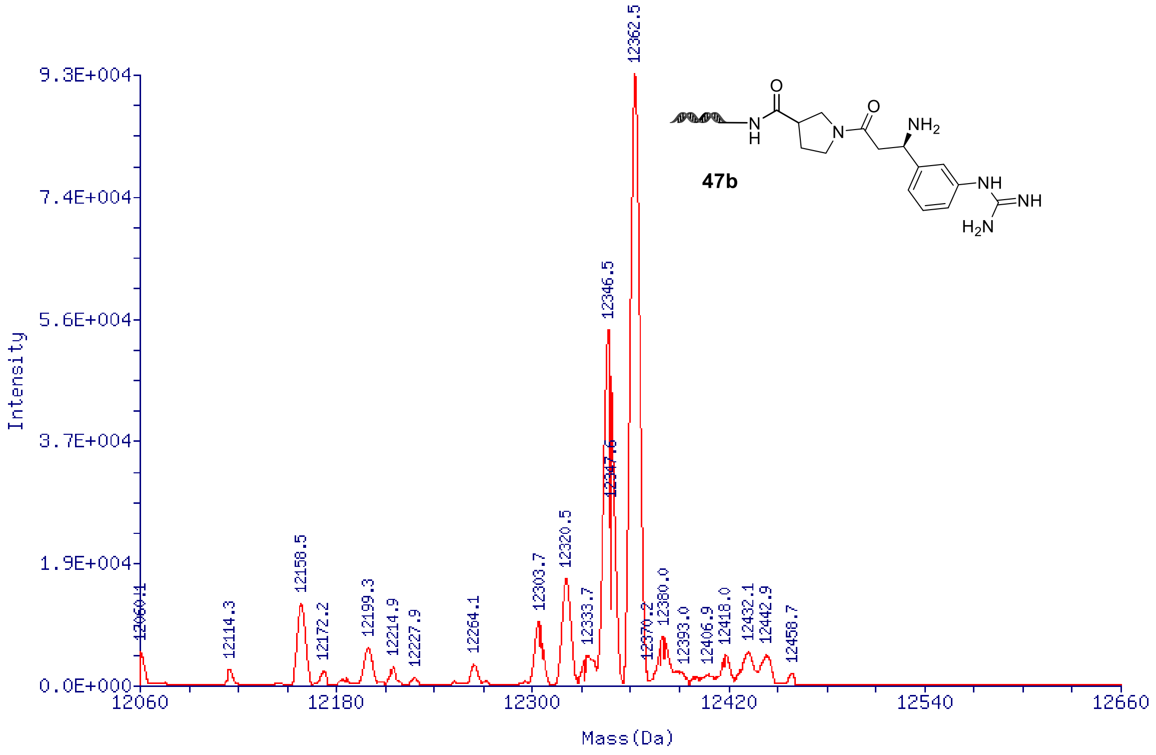
Intensity
Mass (Da)
12362.5
12346.5
12320.5
12303.7
12158.5

47b

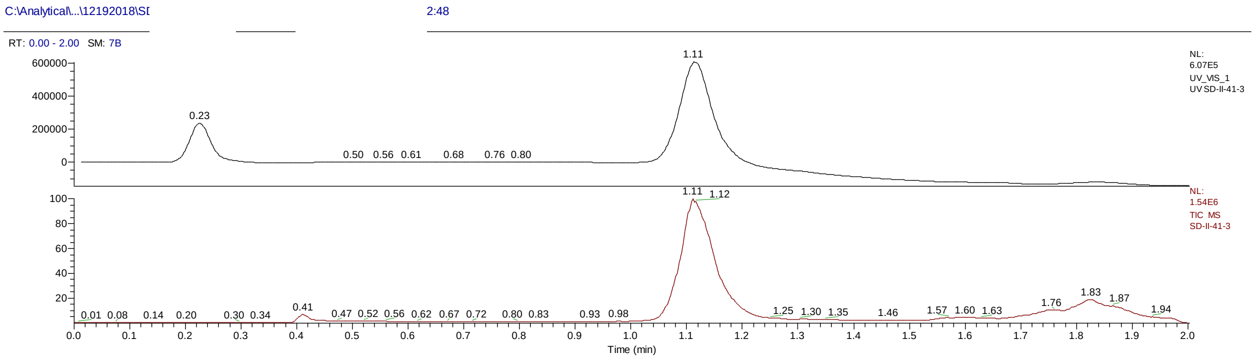

C:\Analytical\...\12192018\SI
2:48
RT: 0.00 - 2.00 SM: 7B
NL: 6.07E5 UV_VIS_1 UV SD-II-41-3
NL: 1.54E6 TIC MS SD-II-41-3
Time (min)

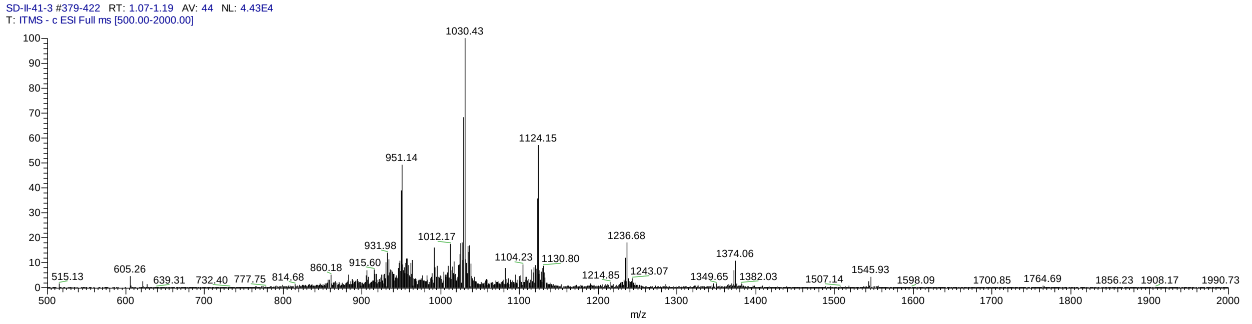

SD-II-41-3 #379-422 RT: 1.07-1.19 AV: 44 NL: 4.43E4
T: ITMS - c ESI Full ms [500.00-2000.00]
1030.43
1124.15
951.14
1236.68
1374.06
m/z

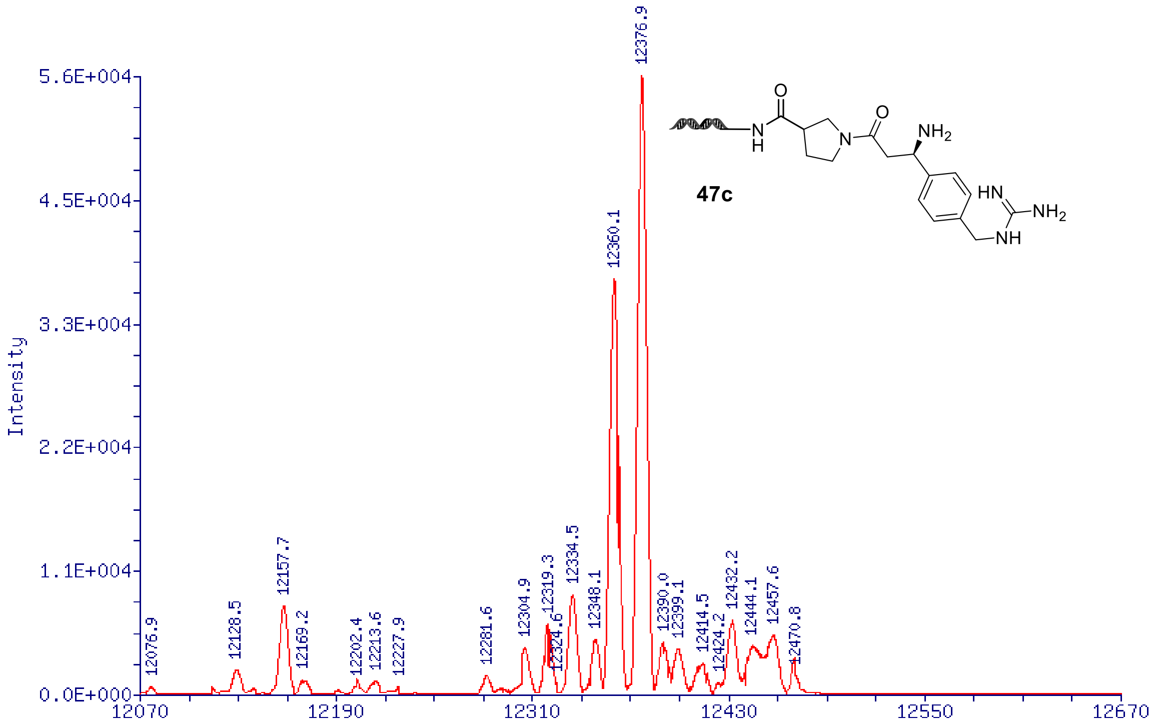

12376.9
12360.1
47c
Intensity
Mass(Da)

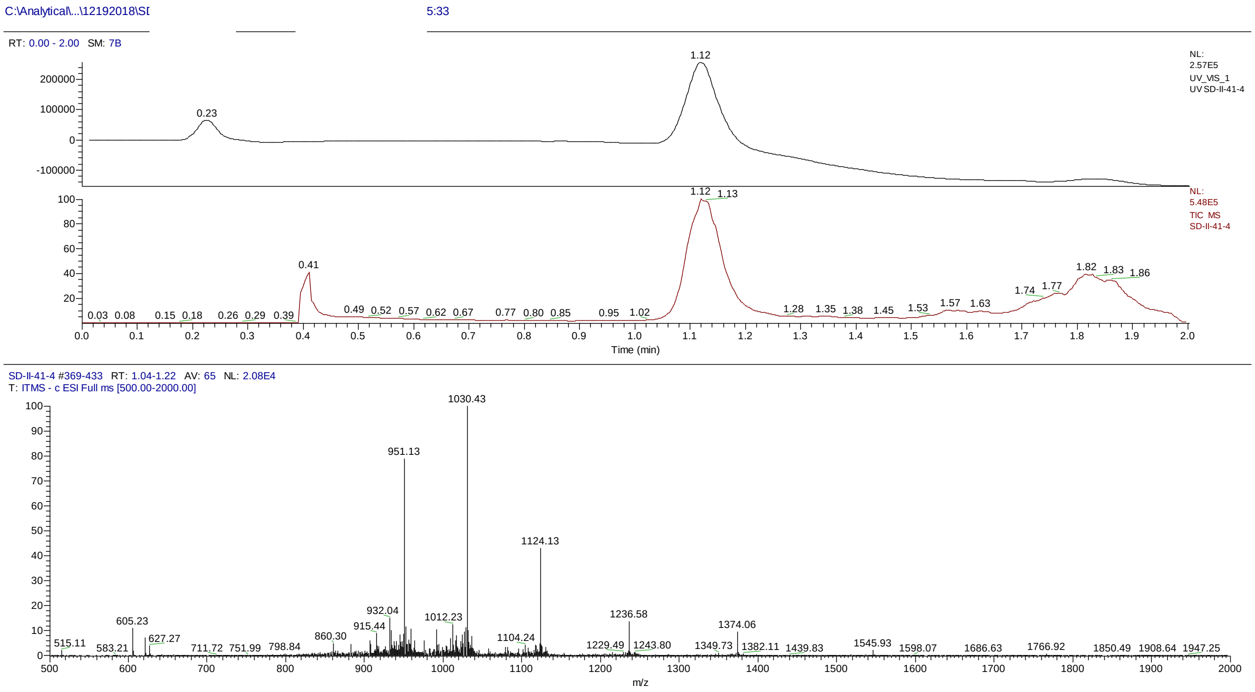
C:\Analytical\...\12192018\SI
5:33
RT: 0.00 - 2.00 SM: 7B
NL: 2.57E5 UV_VIS_1 UV SD-II-41-4
NL: 5.48E5 TIC MS SD-II-41-4
Time (min)
SD-II-41-4 #369-433 RT: 1.04-1.22 AV: 65 NL: 2.08E4
T: ITMS - c ESI Full ms [500.00-2000.00]
1030.43
951.13
1124.13
1236.58
1374.06
m/z

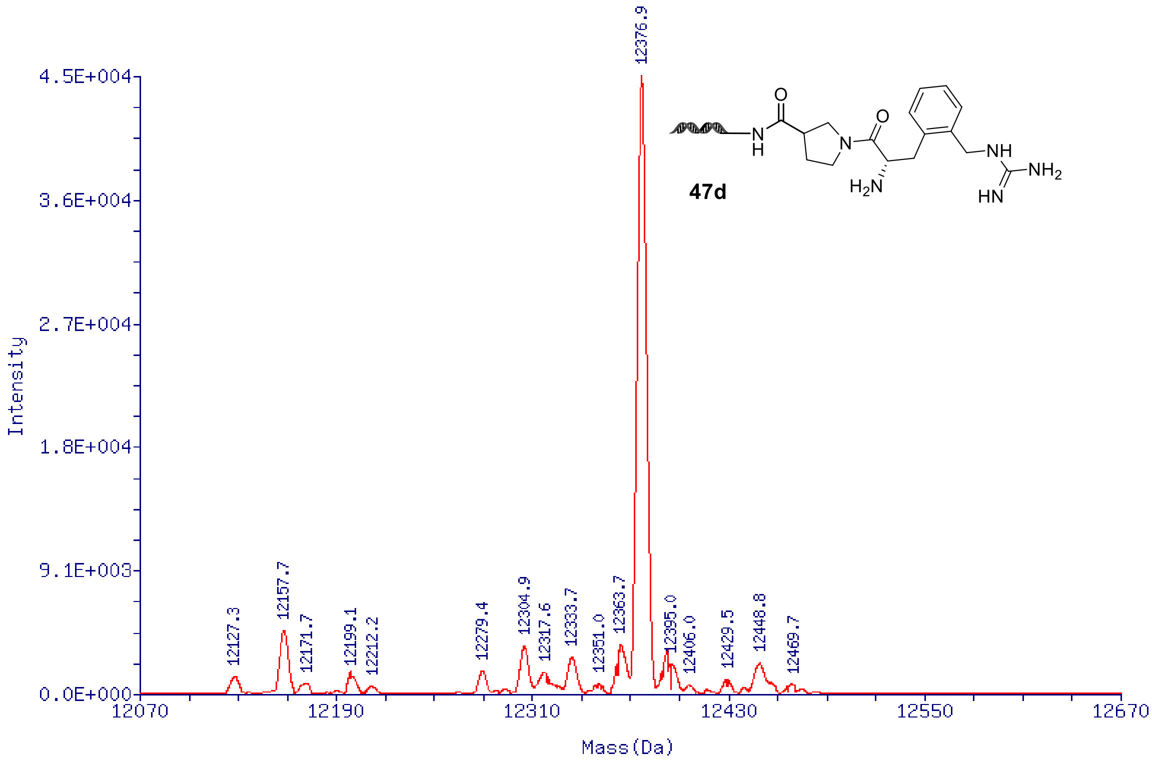
12376.9
Intensity
Mass(Da)
47d

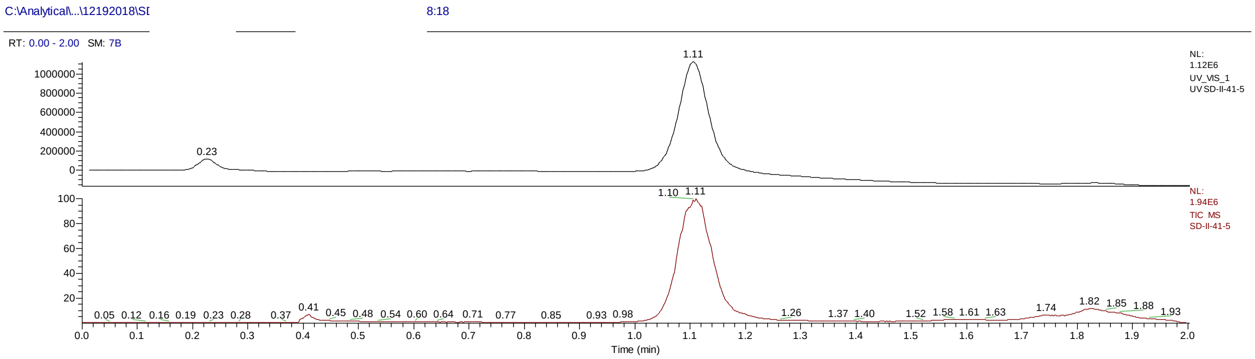
C:\Analytical\...\12192018\SI
8:18
RT: 0.00 - 2.00 SM: 7B
NL: 1.12E6 UV_VIS_1 UV SD-II-41-5
NL: 1.94E6 TIC MS SD-II-41-5
1.11
0.23
1.10 1.11
Time (min)

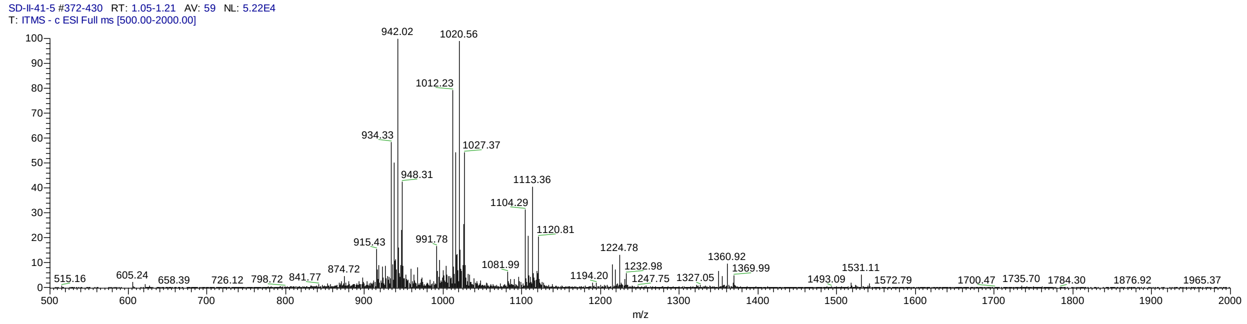
SD-II-41-5 #372-430 RT: 1.05-1.21 AV: 59 NL: 5.22E4
T: ITMS - c ESI Full ms [500.00-2000.00]
942.02
1020.56
1012.23
934.33
1027.37
948.31
1113.36
1104.29
1120.81
m/z

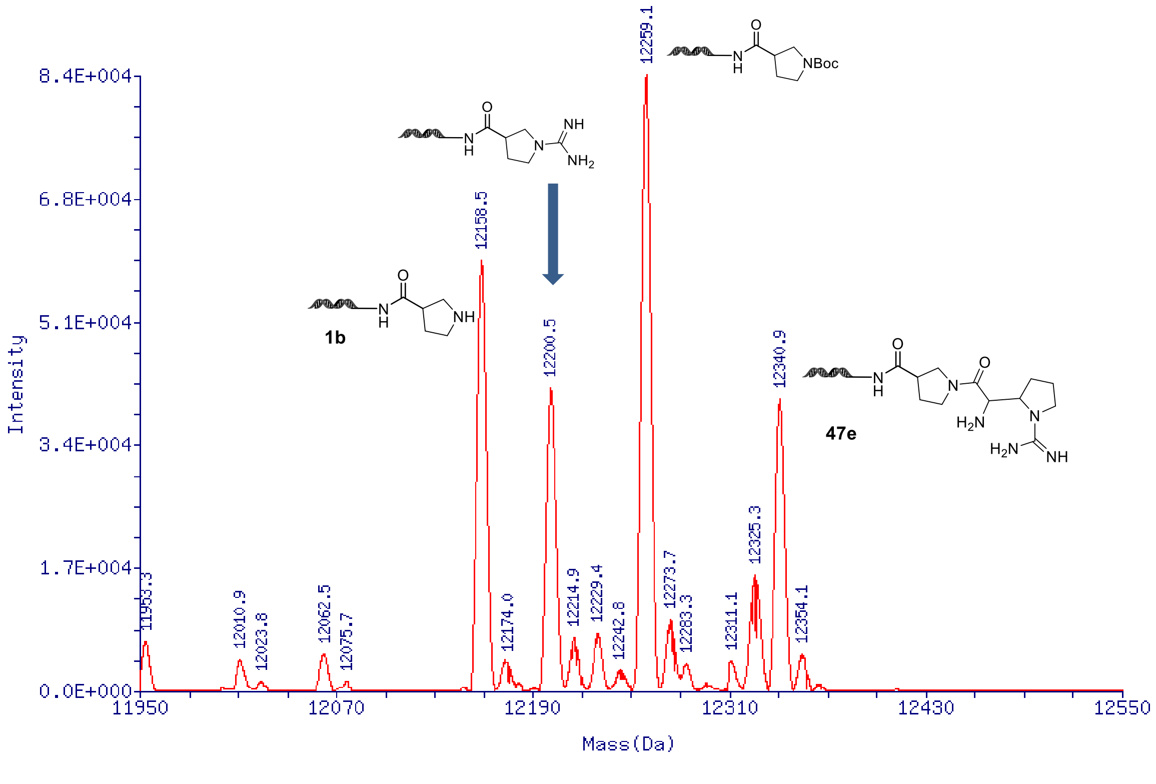
Intensity
Mass(Da)
12259.1
12158.5
12200.5
12340.9
1b
47e
NBoc

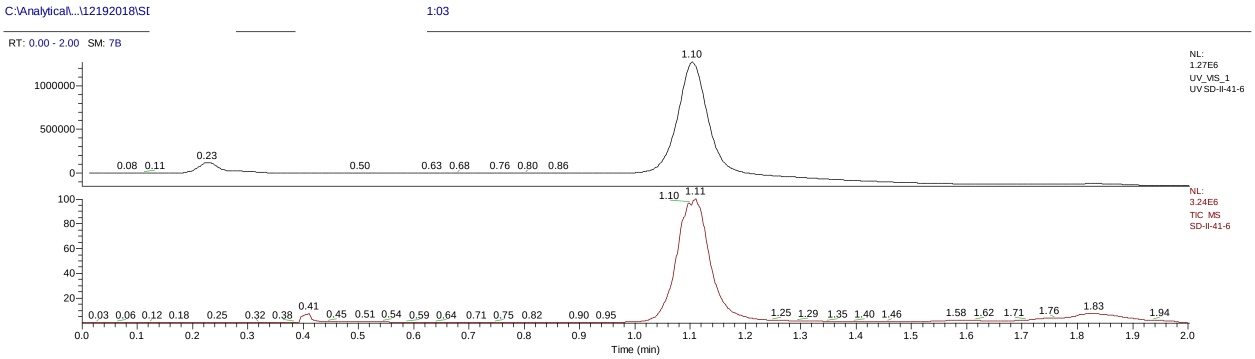
C:\Analytical\...\12192018\SD
1:03
RT: 0.00 - 2.00 SM: 7B
NL: 1.27E6 UV_VIS_1 UV SD-II-41-6
1.10
0.08 0.11 0.23 0.50 0.63 0.68 0.76 0.80 0.86
1000000
500000
0
NL: 3.24E6 TIC MS SD-II-41-6
1.10 1.11
0.03 0.06 0.12 0.18 0.25 0.32 0.38 0.41 0.45 0.51 0.54 0.59 0.64 0.71 0.75 0.82 0.90 0.95 1.25 1.29 1.35 1.40 1.46 1.58 1.62 1.71 1.76 1.83 1.94
100 80 60 40 20
0.0 0.1 0.2 0.3 0.4 0.5 0.6 0.7 0.8 0.9 1.0 1.1 1.2 1.3 1.4 1.5 1.6 1.7 1.8 1.9 2.0
Time (min)

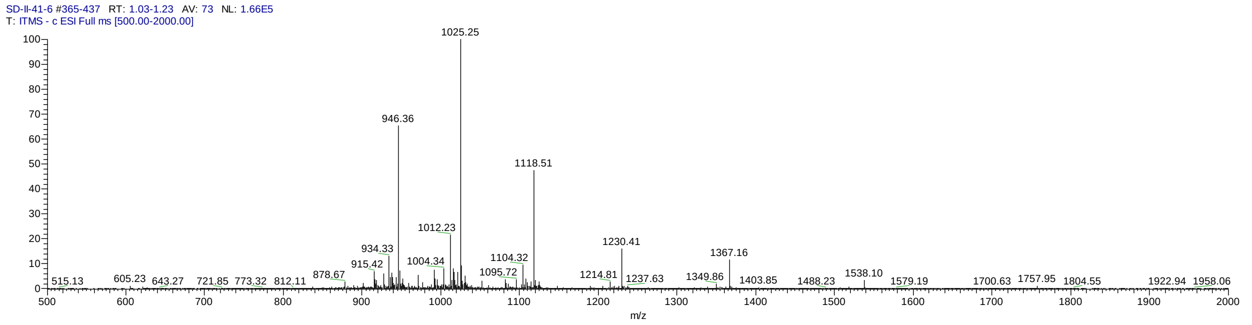
SD-II-41-6 #365-437 RT: 1.03-1.23 AV: 73 NL: 1.66E5
T: ITMS - c ESI Full ms [500.00-2000.00]
1025.25
946.36
1118.51
1012.23
934.33
915.42
1004.34
1104.32
1095.72
1230.41
1367.16
515.13 605.23 643.27 721.85 773.32 812.11 878.67 1214.81 1237.63 1349.86 1403.85 1488.23 1538.10 1579.19 1700.63 1757.95 1804.55 1922.94 1958.06
m/z

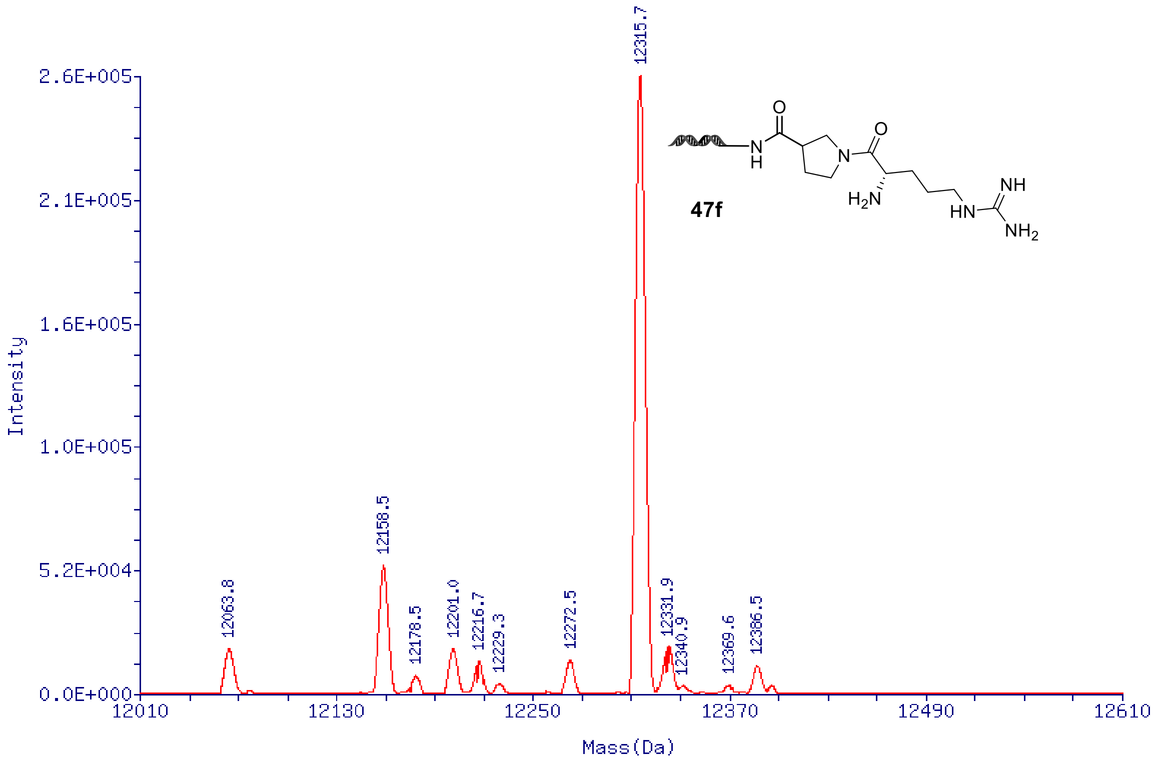
12315.7
12063.8 12158.5 12178.5 12201.0 12216.7 12229.3 12272.5 12331.9 12340.9 12369.6 12386.5
2.6E+005 2.1E+005 1.6E+005 1.0E+005 5.2E+004 0.0E+000
Intensity
12010 12130 12250 12370 12490 12610
Mass(Da)
47f
O
N H
N
O
H_2N
NH
HN
NH_2

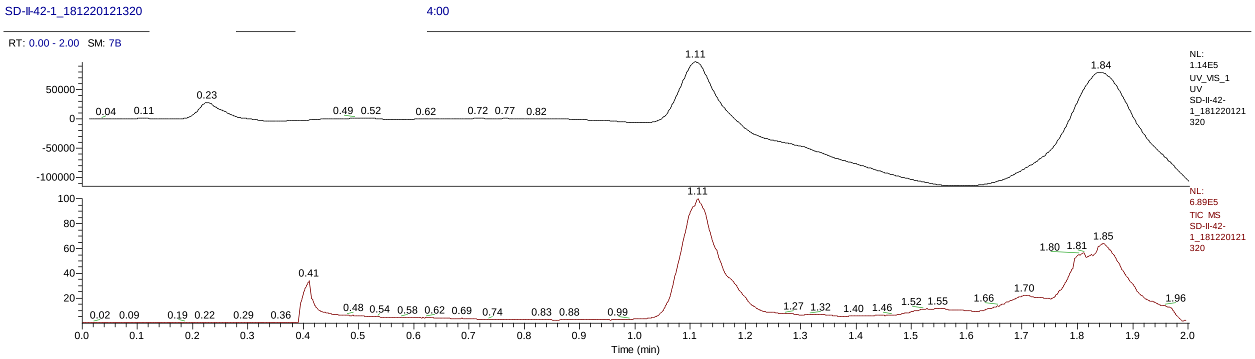
SD-II-42-1_181220121320
4:00
RT: 0.00 - 2.00 SM: 7B
NL: 1.14E5 UV_VIS_1 UV SD-II-42-1_181220121320
NL: 6.89E5 TIC MS SD-II-42-1_181220121320
Time (min)

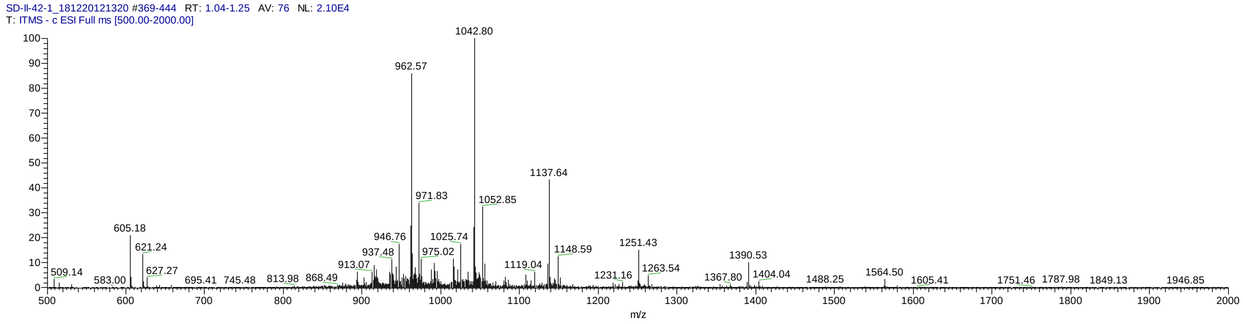
SD-II-42-1_181220121320 #369-444 RT: 1.04-1.25 AV: 76 NL: 2.10E4
T: ITMS - c ESI Full ms [500.00-2000.00]
1042.80
962.57
1137.64
971.83
1052.85
605.18
m/z

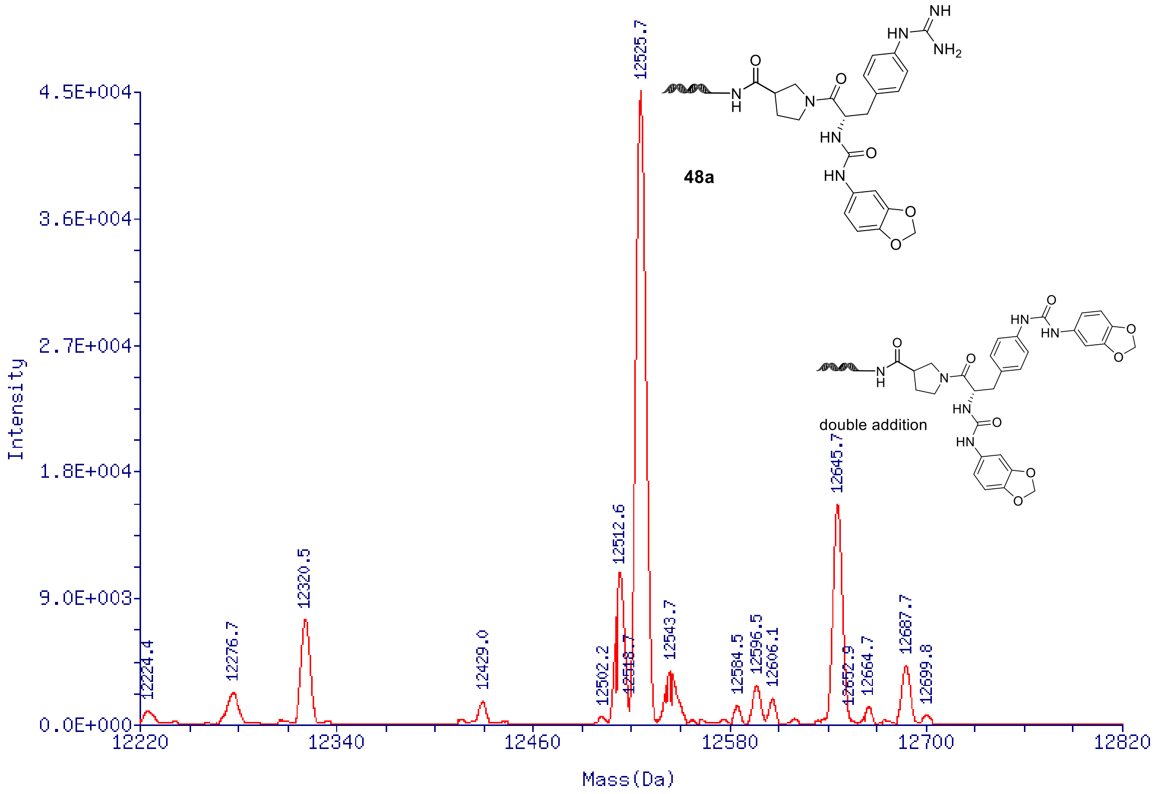
12525.7
12645.7
12512.6
12320.5
Intensity
Mass(Da)
48a
double addition

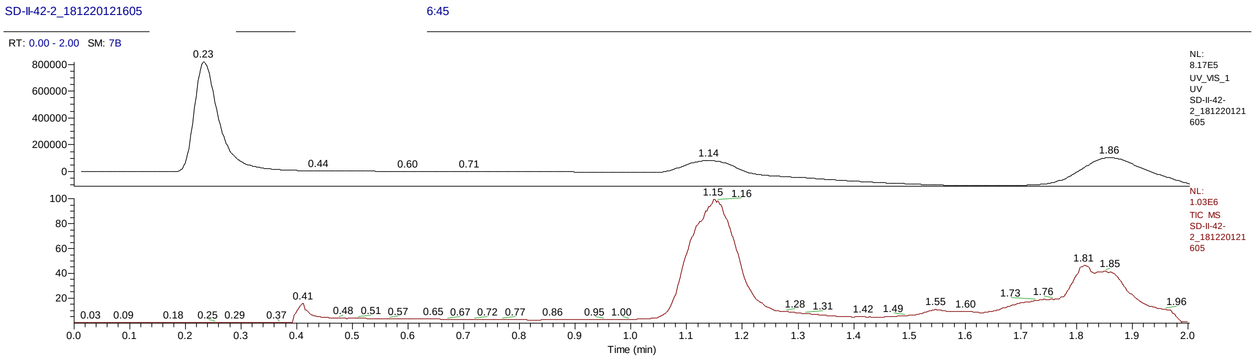
SD-II-42-2_181220121605
6:45
RT: 0.00 - 2.00 SM: 7B
NL: 8.17E5 UV_VIS_1 UV SD-II-42-2_181220121605
NL: 1.03E6 TIC MS SD-II-42-2_181220121605
Time (min)

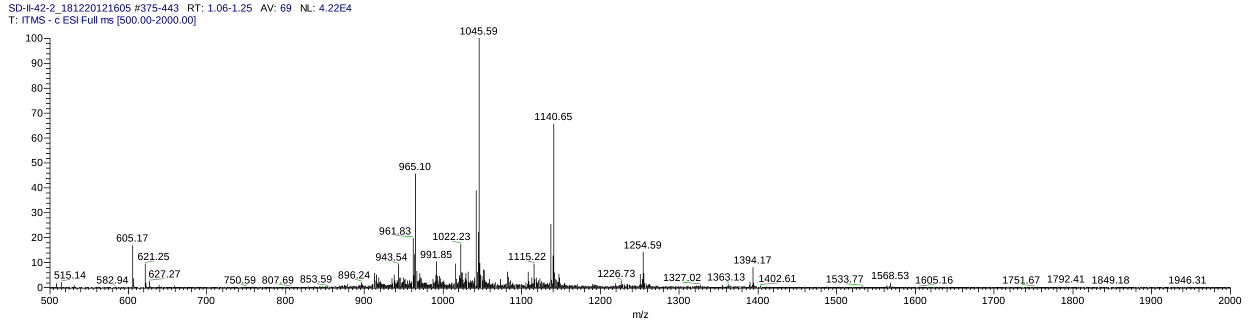
SD-II-42-2_181220121605 #375-443 RT: 1.06-1.25 AV: 69 NL: 4.22E4
T: ITMS - c ESI Full ms [500.00-2000.00]
1045.59
1140.65
965.10
605.17
1254.59
m/z

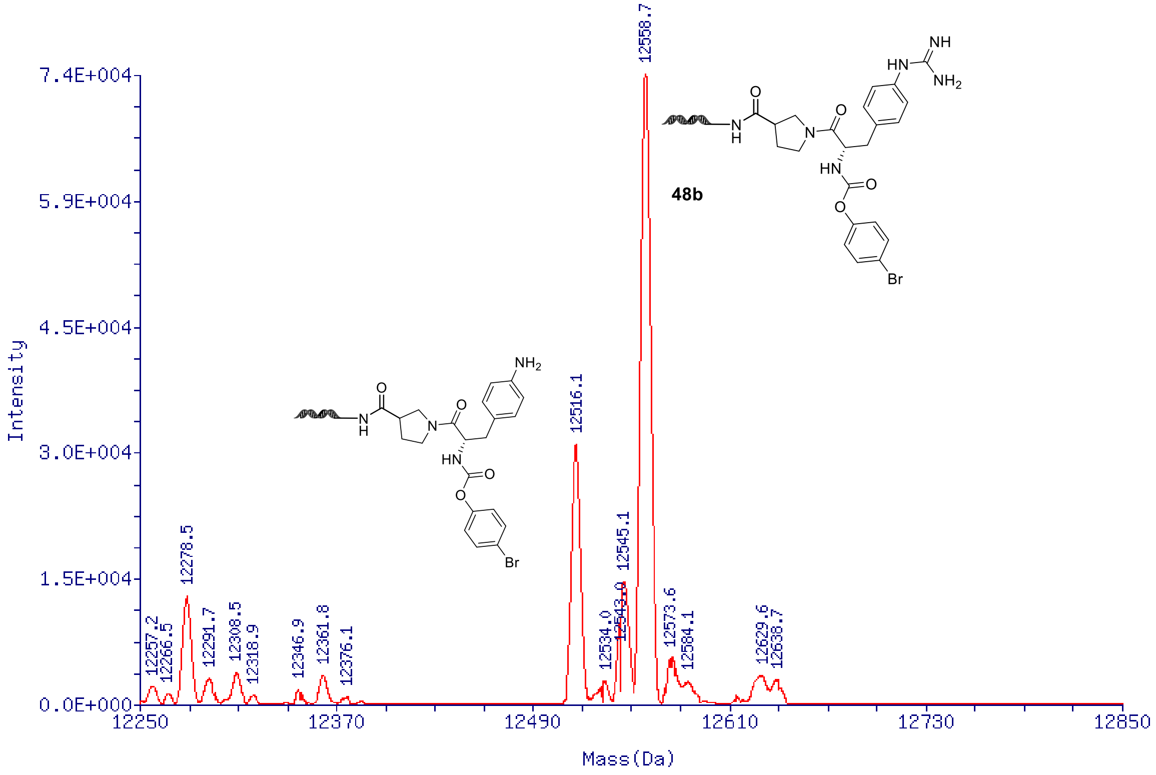
12558.7
12516.1
12545.1
12278.5
48b
Br
Intensity
Mass(Da)

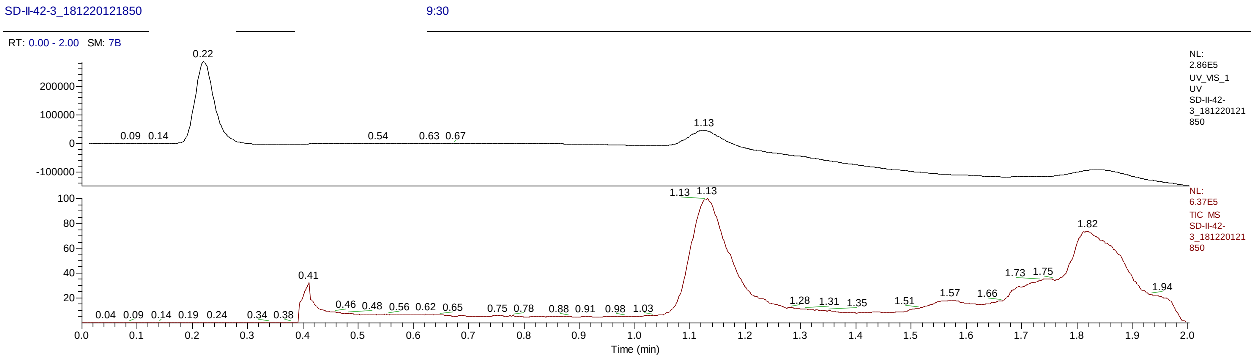
SD-II-42-3_181220121850
9:30
RT: 0.00 - 2.00 SM: 7B
NL: 2.86E5 UV_VIS_1 UV SD-II-42-3_181220121850
NL: 6.37E5 TIC MS SD-II-42-3_181220121850
Time (min)

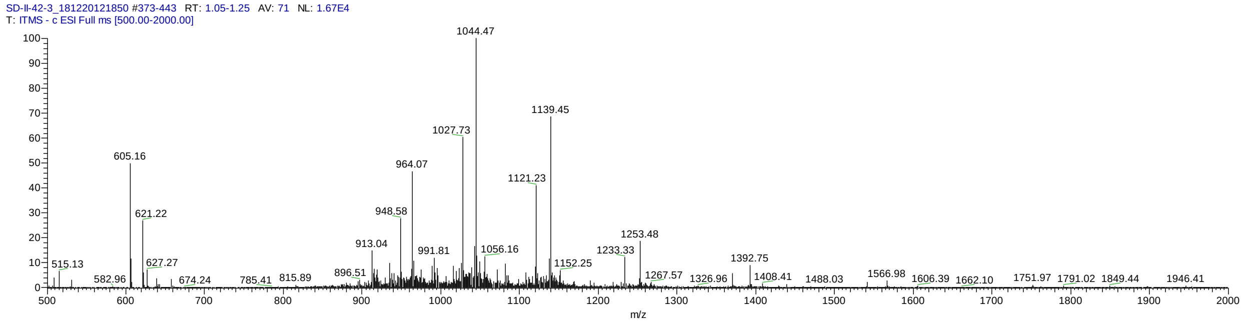
SD-II-42-3_181220121850 #373-443 RT: 1.05-1.25 AV: 71 NL: 1.67E4
T: ITMS - c ESI Full ms [500.00-2000.00]
m/z

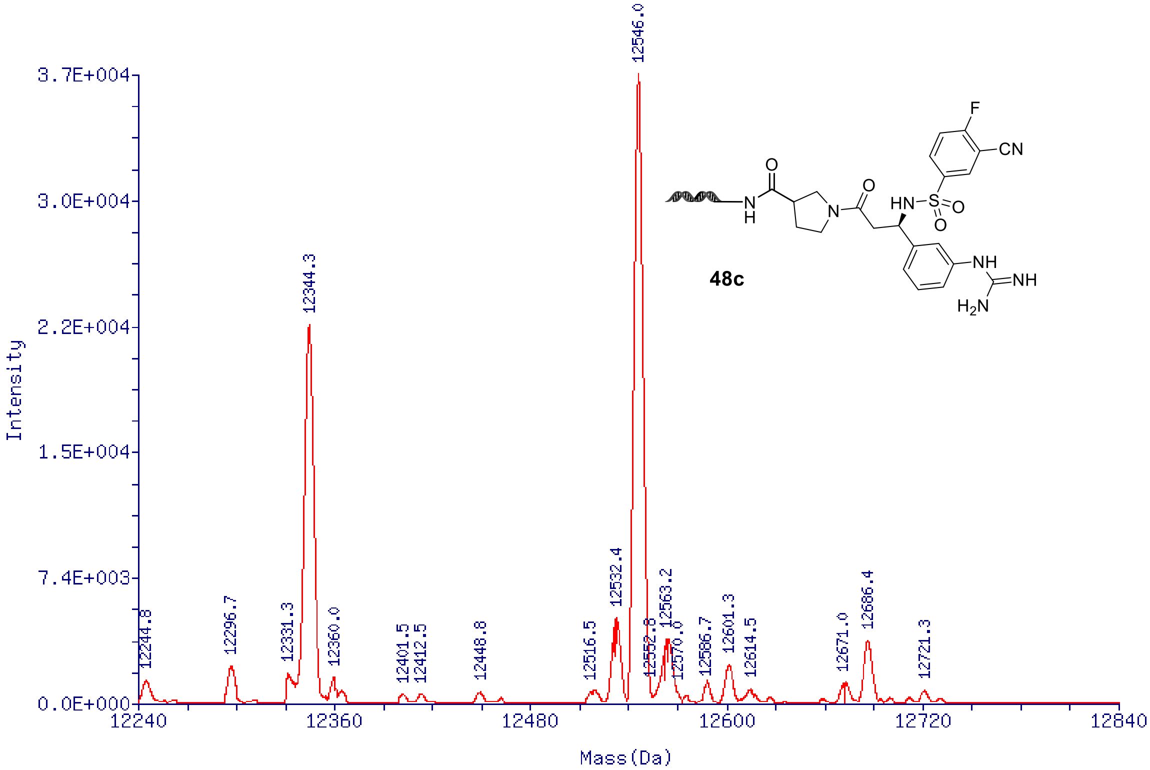
Intensity
Mass(Da)
12546.0
12344.3
48c

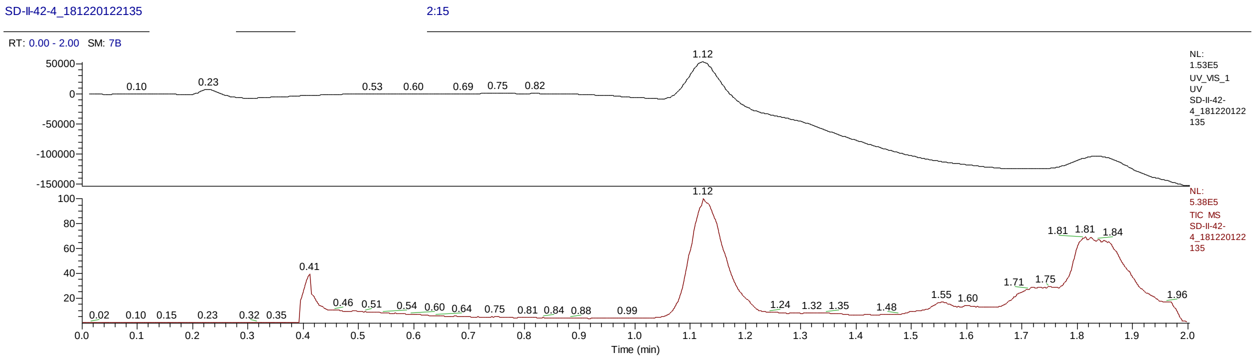
SD-II-42-4_181220122135
2:15
RT: 0.00 - 2.00 SM: 7B
NL: 1.53E5 UV_VIS_1 UV SD-II-42-4_181220122135
NL: 5.38E5 TIC MS SD-II-42-4_181220122135
Time (min)

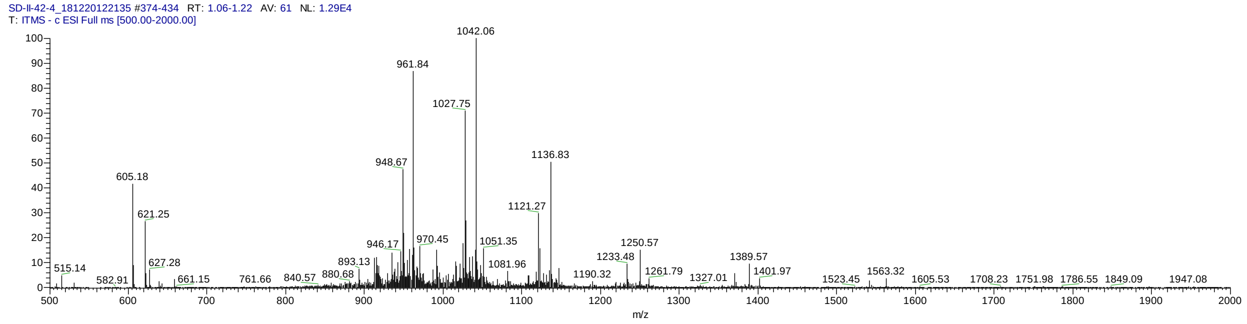
SD-II-42-4_181220122135 #374-434 RT: 1.06-1.22 AV: 61 NL: 1.29E4
T: ITMS - c ESI Full ms [500.00-2000.00]
1042.06
961.84
1027.75
1136.83
948.67
605.18
1121.27
621.25
m/z

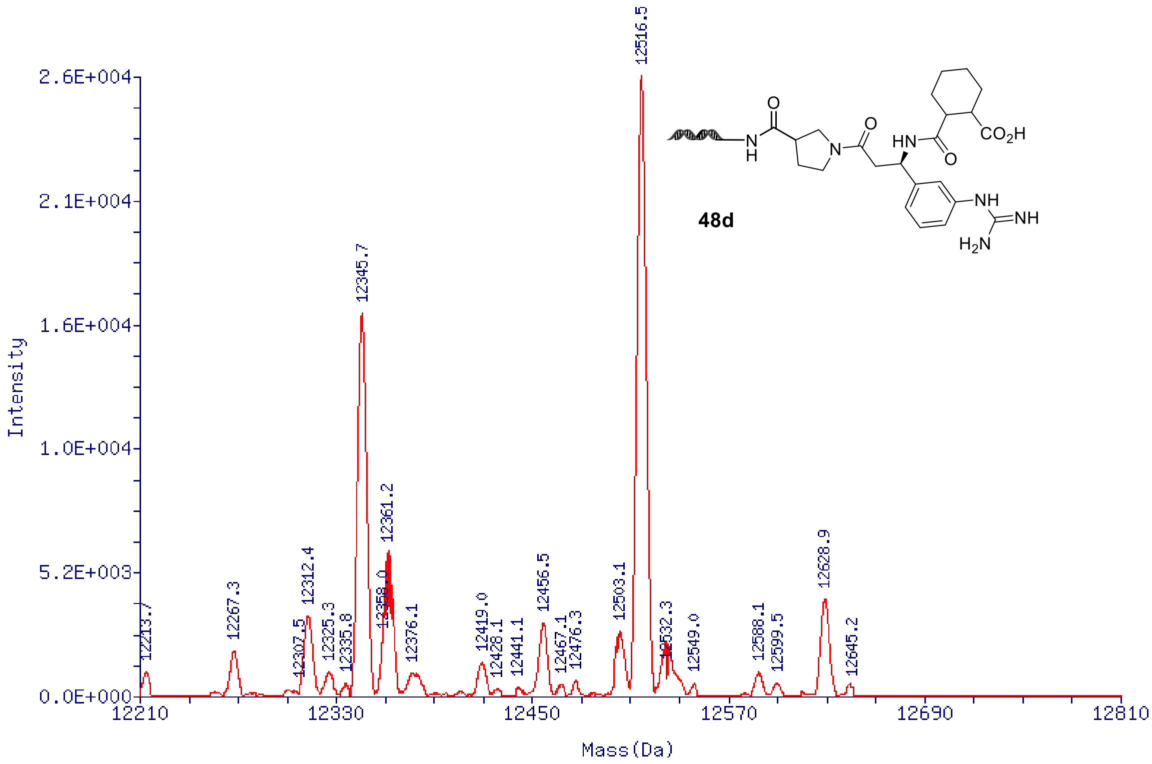
12516.5
12345.7
12361.2
12628.9
Intensity
Mass(Da)
48d
CO2H
HN
NH
H2N

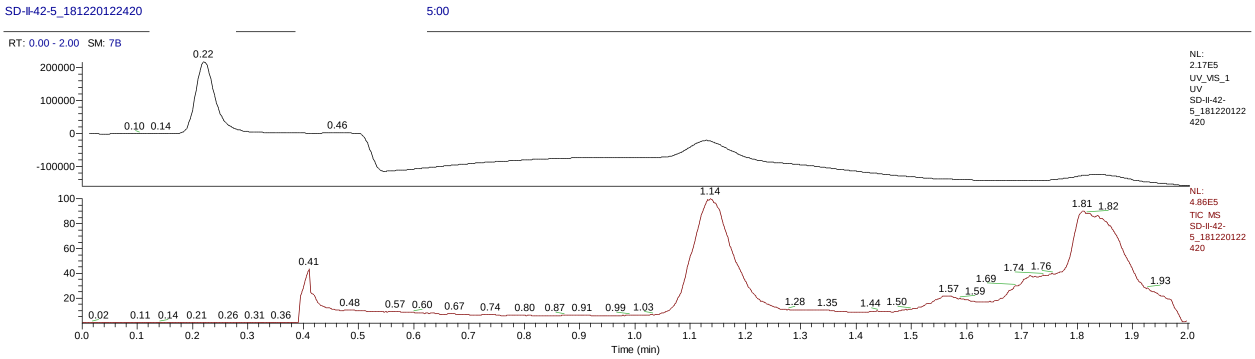
SD-II-42-5_181220122420
5:00
RT: 0.00 - 2.00 SM: 7B
NL: 2.17E5 UV_VIS_1 UV SD-II-42-5_181220122420
NL: 4.86E5 TIC MS SD-II-42-5_181220122420
Time (min)

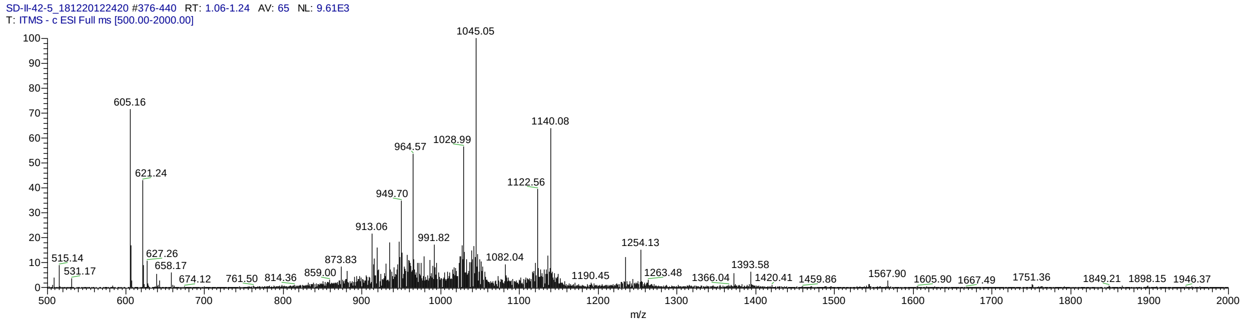
SD-II-42-5_181220122420 #376-440 RT: 1.06-1.24 AV: 65 NL: 9.61E3
T: ITMS - c ESI Full ms [500.00-2000.00]
1045.05
605.16
1140.08
m/z

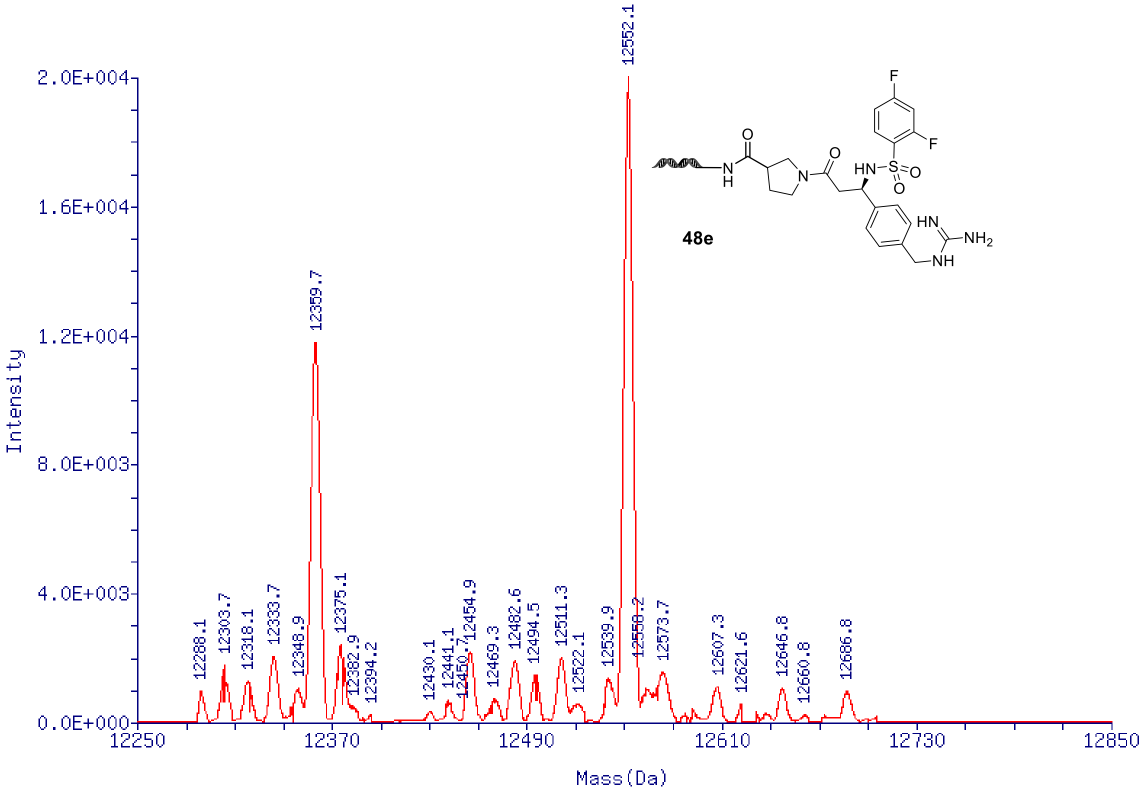
12552.1
12359.7
Intensity
Mass(Da)

48e

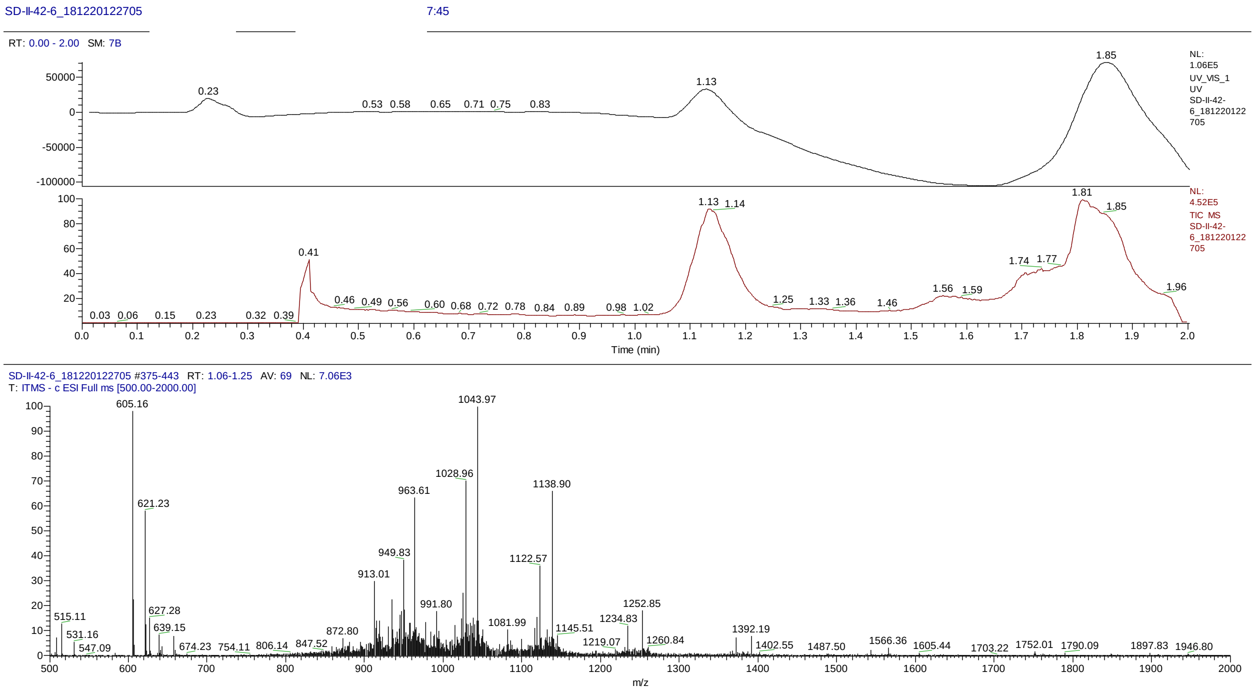
SD-II-42-6_181220122705
7:45
RT: 0.00 - 2.00 SM: 7B
NL: 1.06E5 UV_VIS_1 UV SD-II-42-6_181220122705
NL: 4.52E5 TIC MS SD-II-42-6_181220122705
Time (min)
SD-II-42-6_181220122705 #375-443 RT: 1.06-1.25 AV: 69 NL: 7.06E3
T: ITMS - c ESI Full ms [500.00-2000.00]
m/z

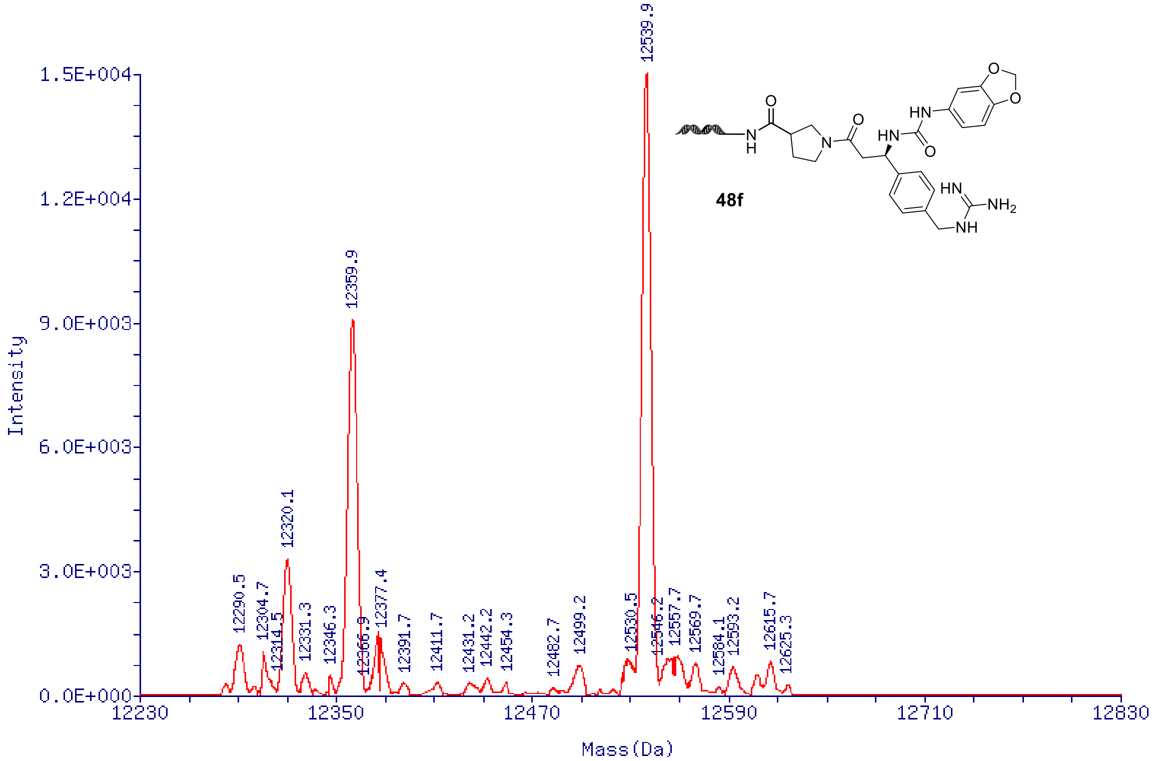
Intensity
Mass(Da)
12539.9
12359.9
12320.1
48f

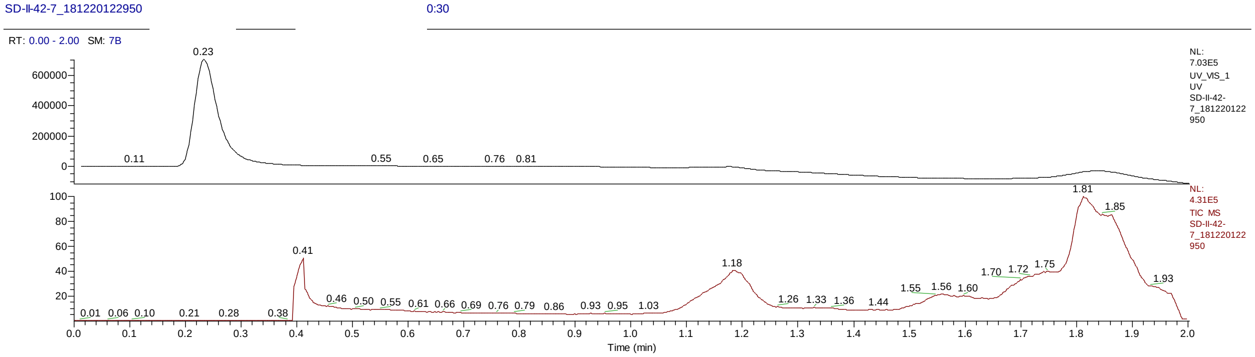
SD-II-42-7_181220122950
0:30
RT: 0.00 - 2.00 SM: 7B
NL: 7.03E5 UV_VIS_1 UV SD-II-42-7_181220122950
NL: 4.31E5 TIC MS SD-II-42-7_181220122950
0.23
0.41
1.18
1.81
1.85
Time (min)

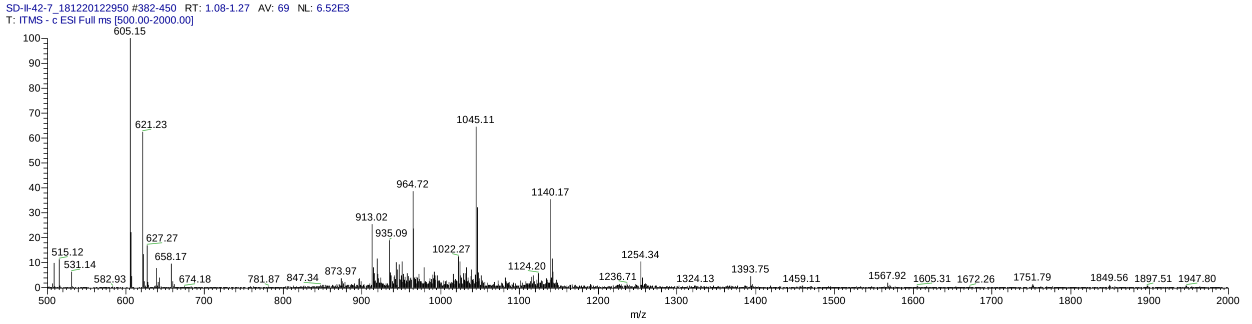
SD-II-42-7_181220122950 #382-450 RT: 1.08-1.27 AV: 69 NL: 6.52E3
T: ITMS - c ESI Full ms [500.00-2000.00]
605.15
621.23
1045.11
964.72
1140.17
913.02
935.09
1022.27
1254.34
m/z

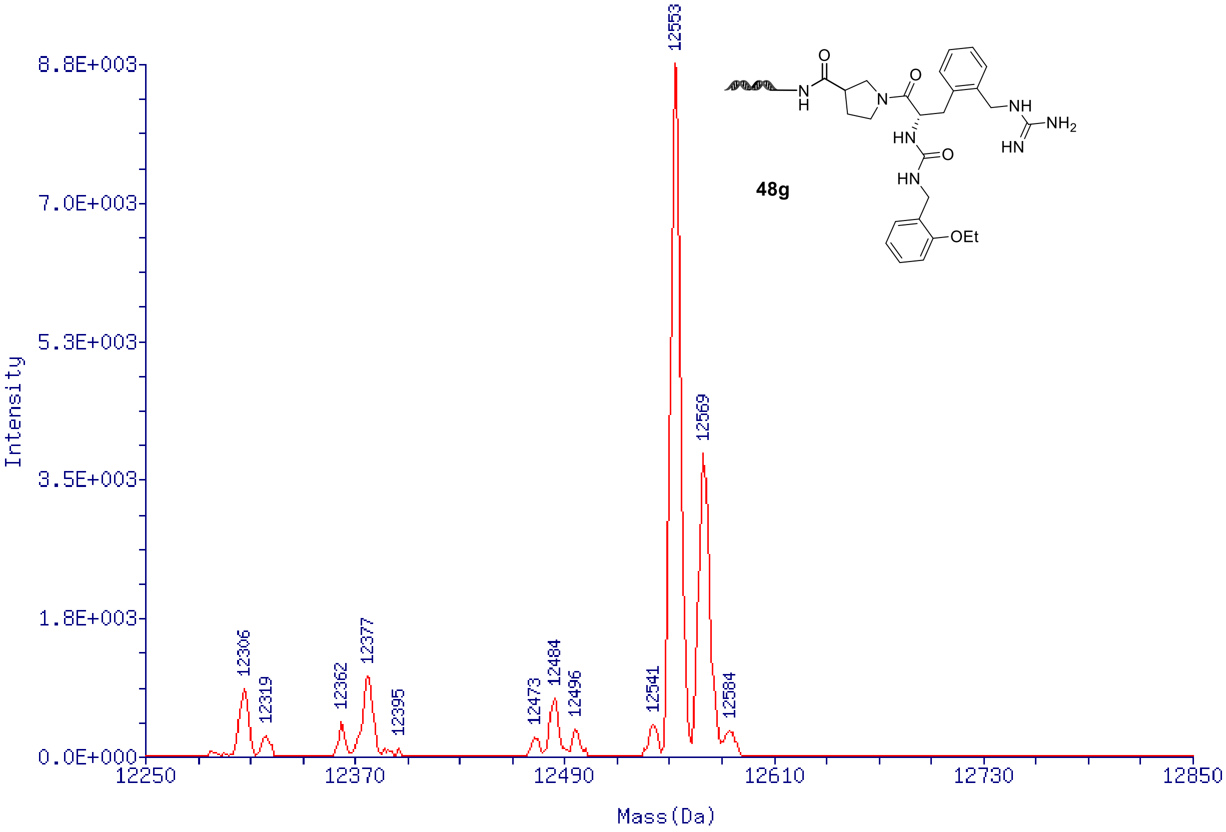
Intensity
Mass(Da)
12553
12569
12306
12319
12362
12377
12395
12473
12484
12496
12541
12584
N
H
O
N
HN
HN
O
NH
NH2
HN
OEt

48g

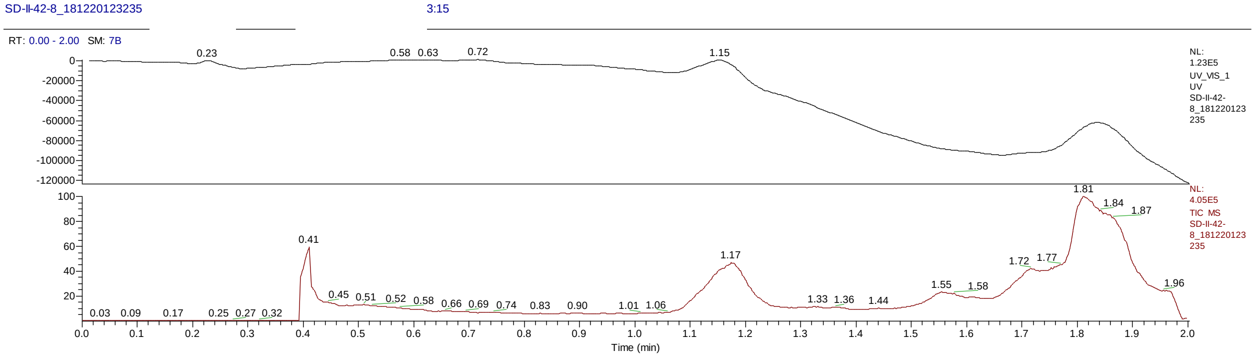
SD-II-42-8_181220123235
3:15
RT: 0.00 - 2.00 SM: 7B
NL: 1.23E5 UV_VIS_1 UV SD-II-42-8_181220123235
NL: 4.05E5 TIC MS SD-II-42-8_181220123235
Time (min)

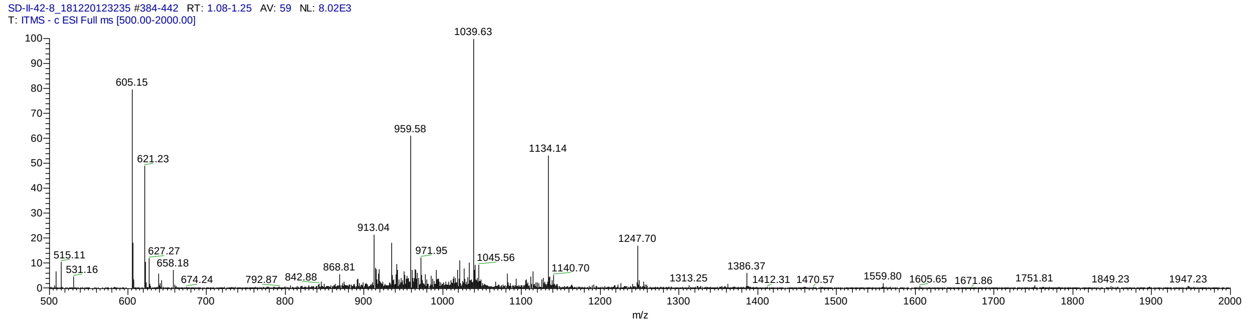
SD-II-42-8_181220123235 #384-442 RT: 1.08-1.25 AV: 59 NL: 8.02E3
T: ITMS - c ESI Full ms [500.00-2000.00]
m/z

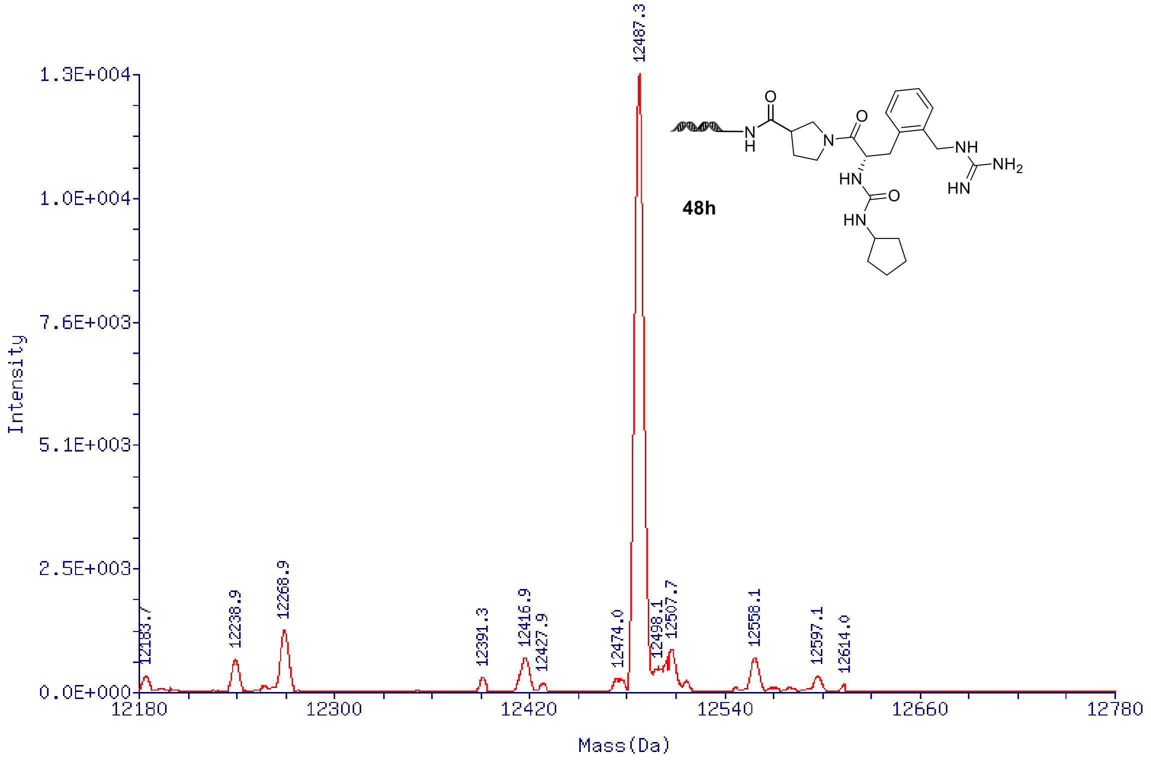
Intensity
Mass(Da)
48h

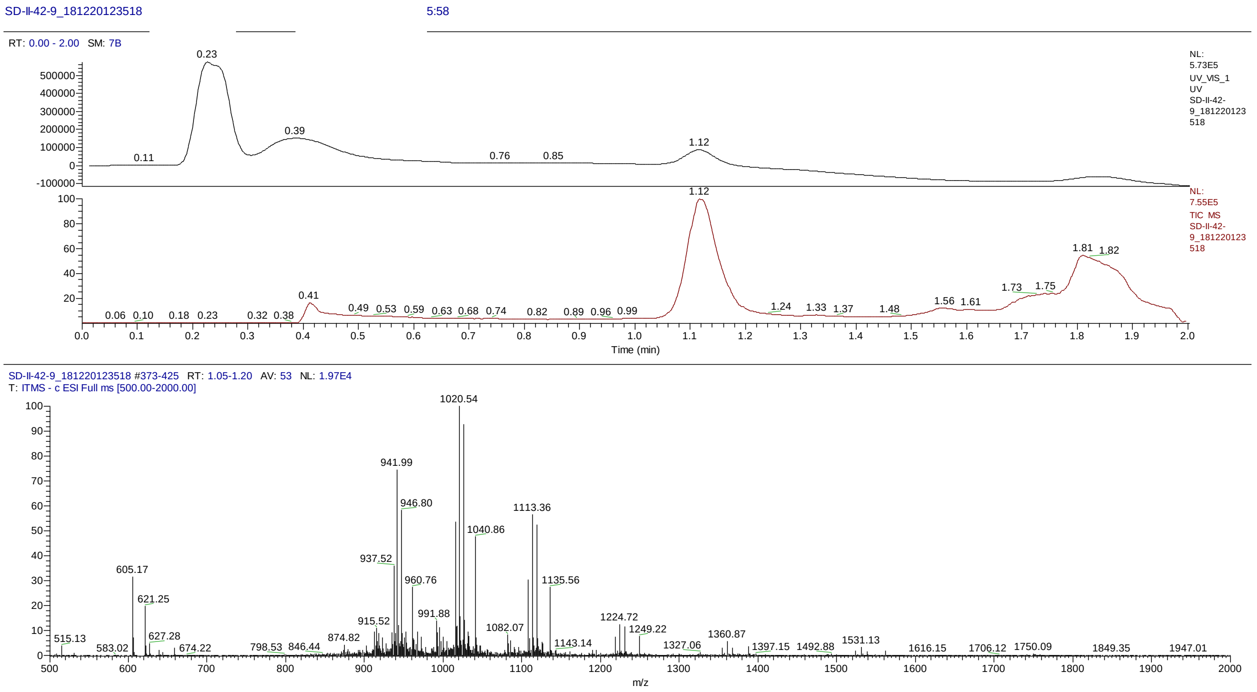

SD-II-42-9_181220123518
5:58
RT: 0.00 - 2.00 SM: 7B
NL: 5.73E5 UV_VIS_1 UV SD-II-42-9_181220123518
NL: 7.55E5 TIC MS SD-II-42-9_181220123518
Time (min)
SD-II-42-9_181220123518 #373-425 RT: 1.05-1.20 AV: 53 NL: 1.97E4
T: ITMS - c ESI Full ms [500.00-2000.00]
m/z

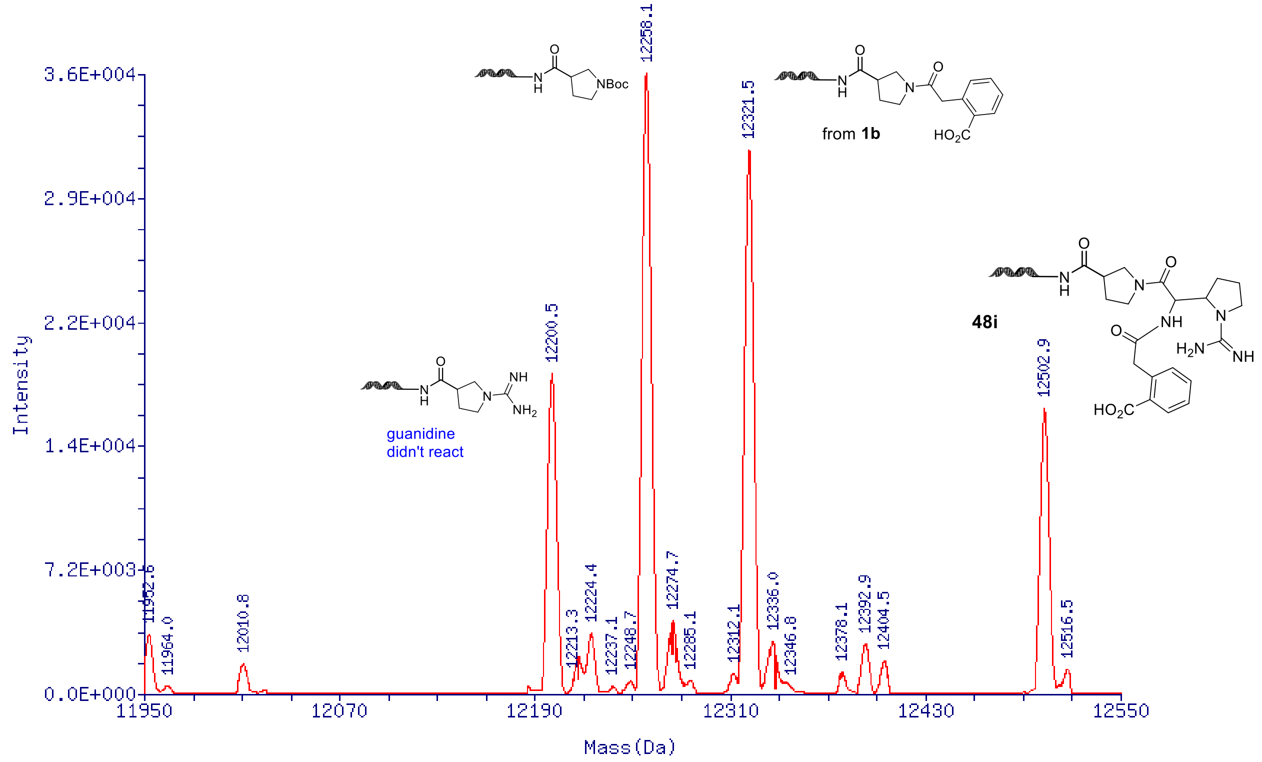

Intensity
Mass(Da)
NBoc
from 1b
HO2C
48i
guanidine
didn't react

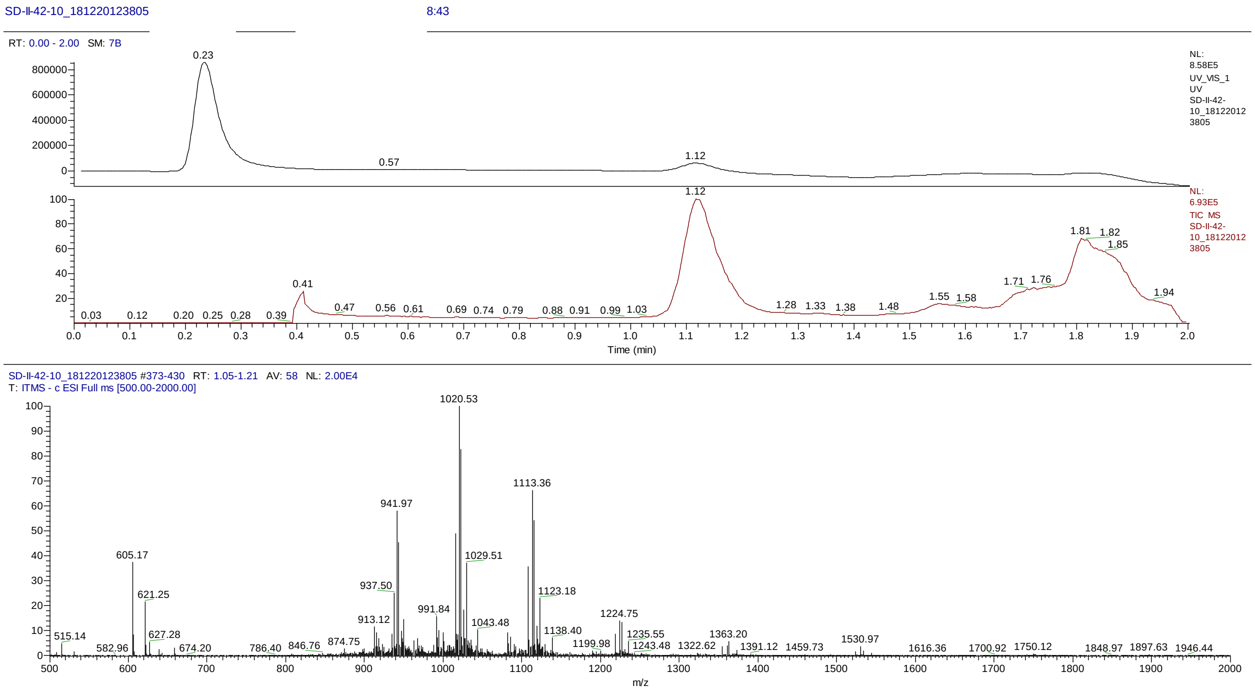
SD-II-42-10_181220123805
8:43
RT: 0.00 - 2.00 SM: 7B
NL: 8.58E5 UV_VIS_1 UV SD-II-42-10_181220123805
NL: 6.93E5 TIC MS SD-II-42-10_181220123805
Time (min)
SD-II-42-10_181220123805 #373-430 RT: 1.05-1.21 AV: 58 NL: 2.00E4
T: ITMS - c ESI Full ms [500.00-2000.00]
1020.53
1113.36
941.97
605.17
m/z

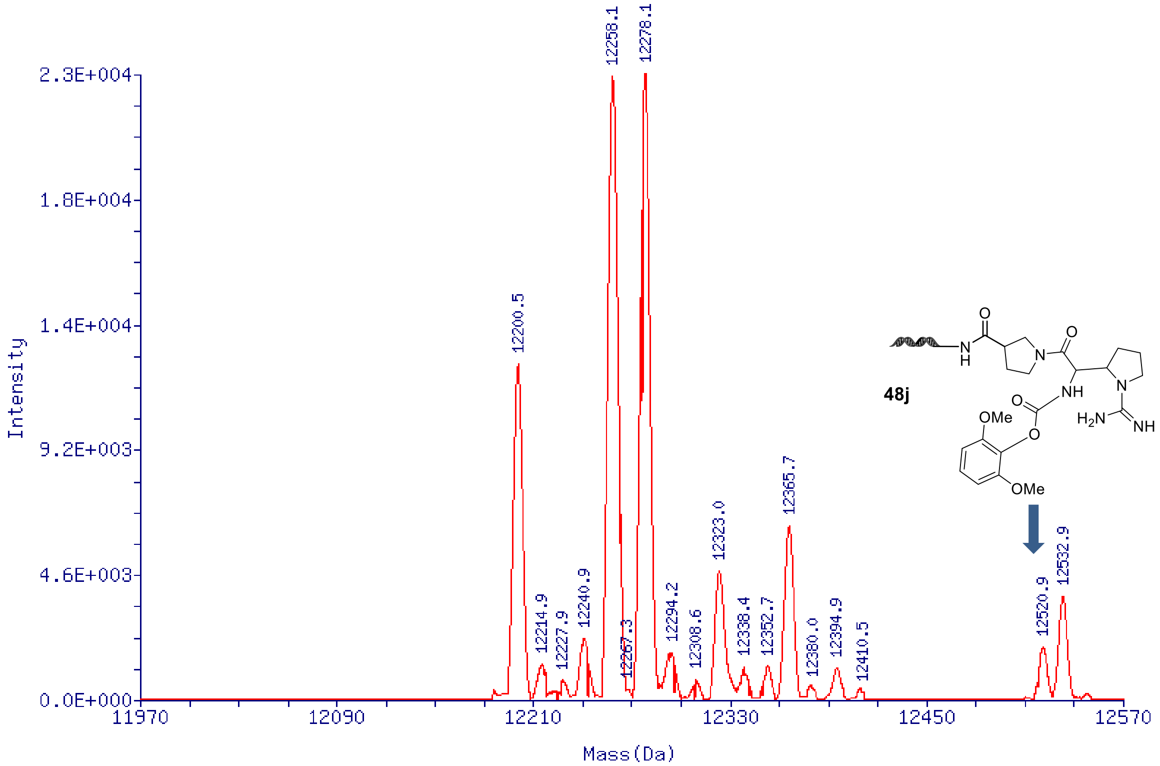
Intensity
Mass(Da)
12258.1
12278.1
12200.5
12365.7
12323.0
12532.9
12520.9
48j

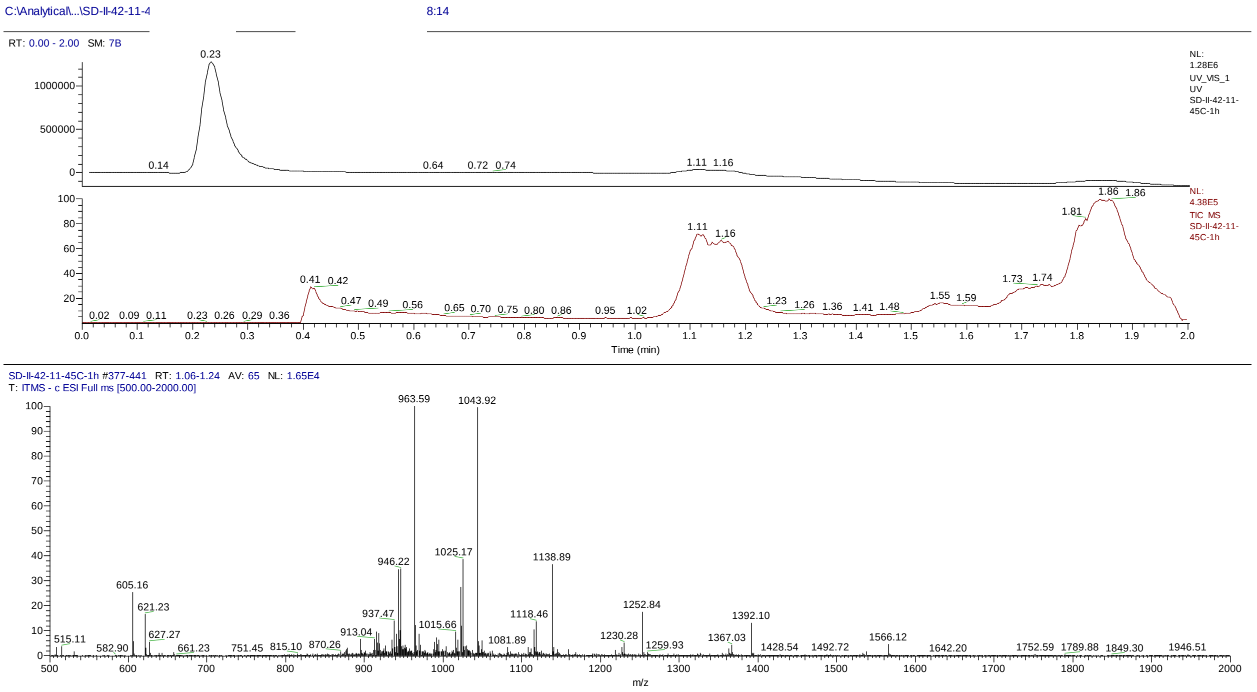
C:\Analytical\...\SD-II-42-11-4
8:14
RT: 0.00 - 2.00 SM: 7B
NL: 1.28E6 UV_VIS_1 UV SD-II-42-11-45C-1h
NL: 4.38E5 TIC MS SD-II-42-11-45C-1h
Time (min)
SD-II-42-11-45C-1h #377-441 RT: 1.06-1.24 AV: 65 NL: 1.65E4
T: ITMS - c ESI Full ms [500.00-2000.00]
963.59
1043.92
1138.89
1025.17
946.22
605.16
1252.84
1392.10
m/z

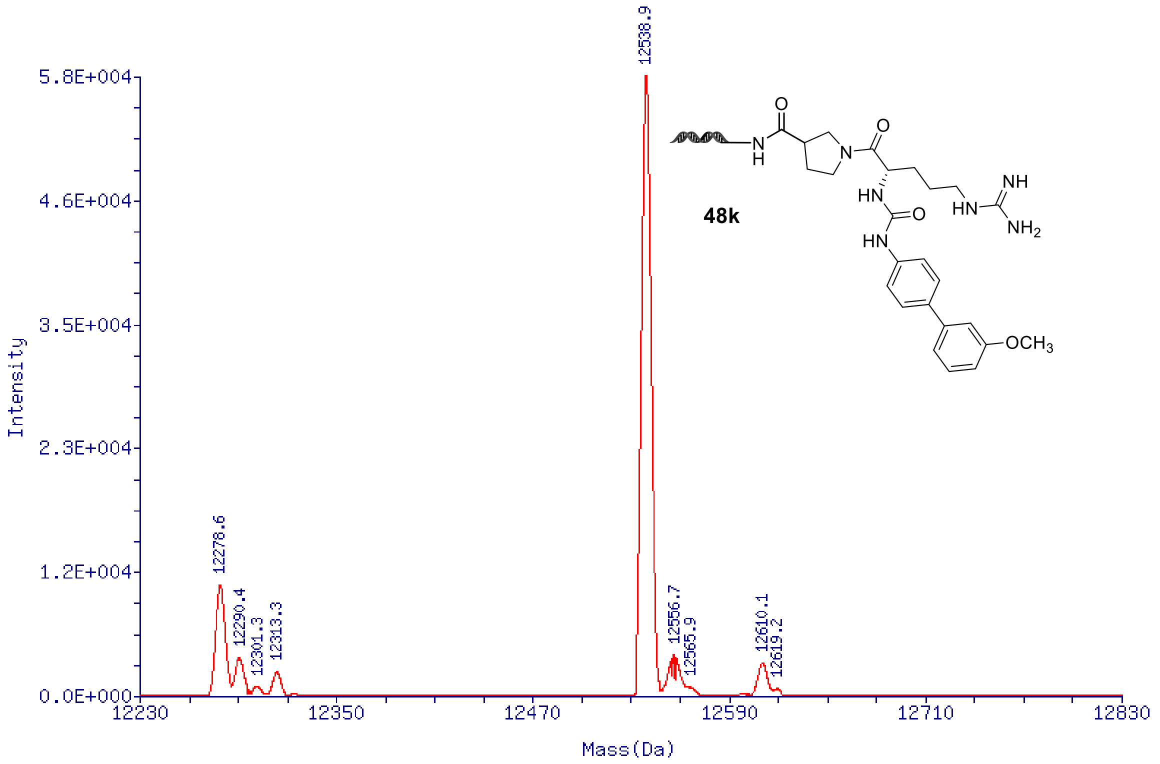
12538.9
12278.6
12290.4
12301.3
12313.3
12556.7
12565.9
12610.1
12619.2
Intensity
Mass(Da)
48k
OCH3
NH2

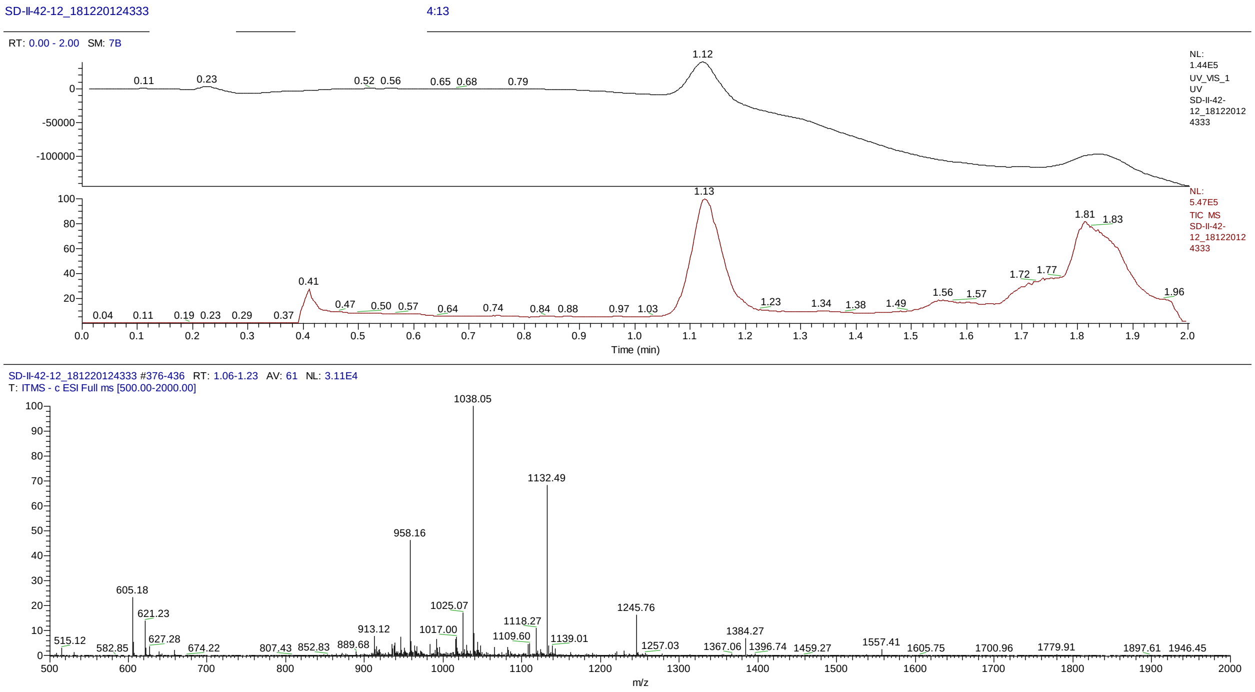

SD-II-42-12_181220124333
4:13
RT: 0.00 - 2.00 SM: 7B
NL: 1.44E5 UV_VIS_1 UV SD-II-42-12_181220124333
NL: 5.47E5 TIC MS SD-II-42-12_181220124333
Time (min)
SD-II-42-12_181220124333 #376-436 RT: 1.06-1.23 AV: 61 NL: 3.11E4
T: ITMS - c ESI Full ms [500.00-2000.00]
1038.05
1132.49
958.16
605.18
1245.76
m/z

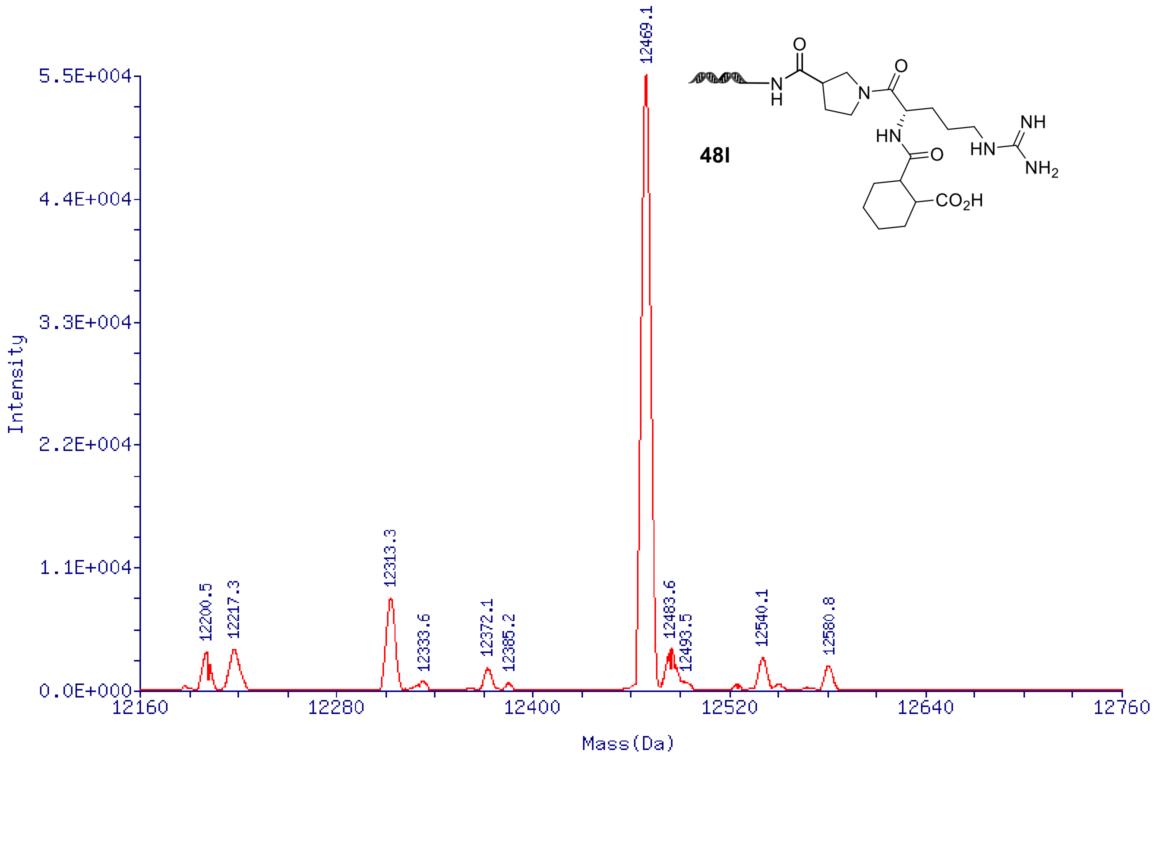

12469.1
12313.3
12200.5
12217.3
12333.6
12372.1
12385.2
12483.6
12493.5
12540.1
12580.8
Intensity
Mass(Da)
48l

7e) On-DNA re-synthesis of hits

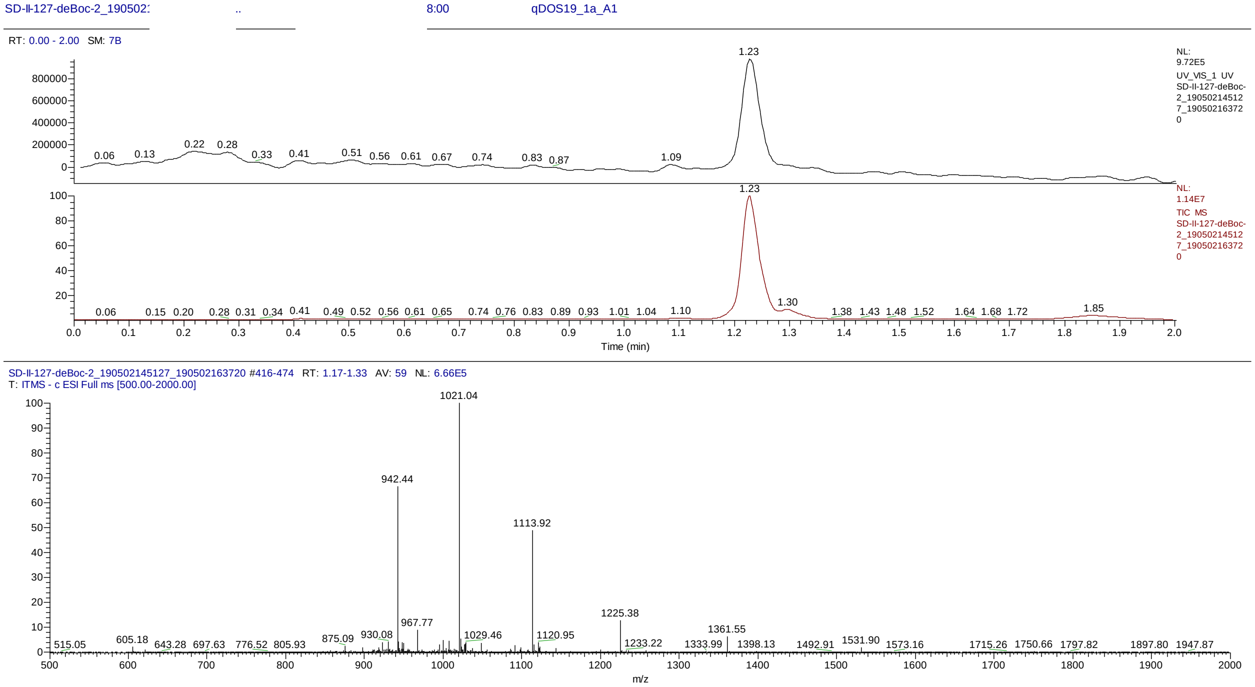

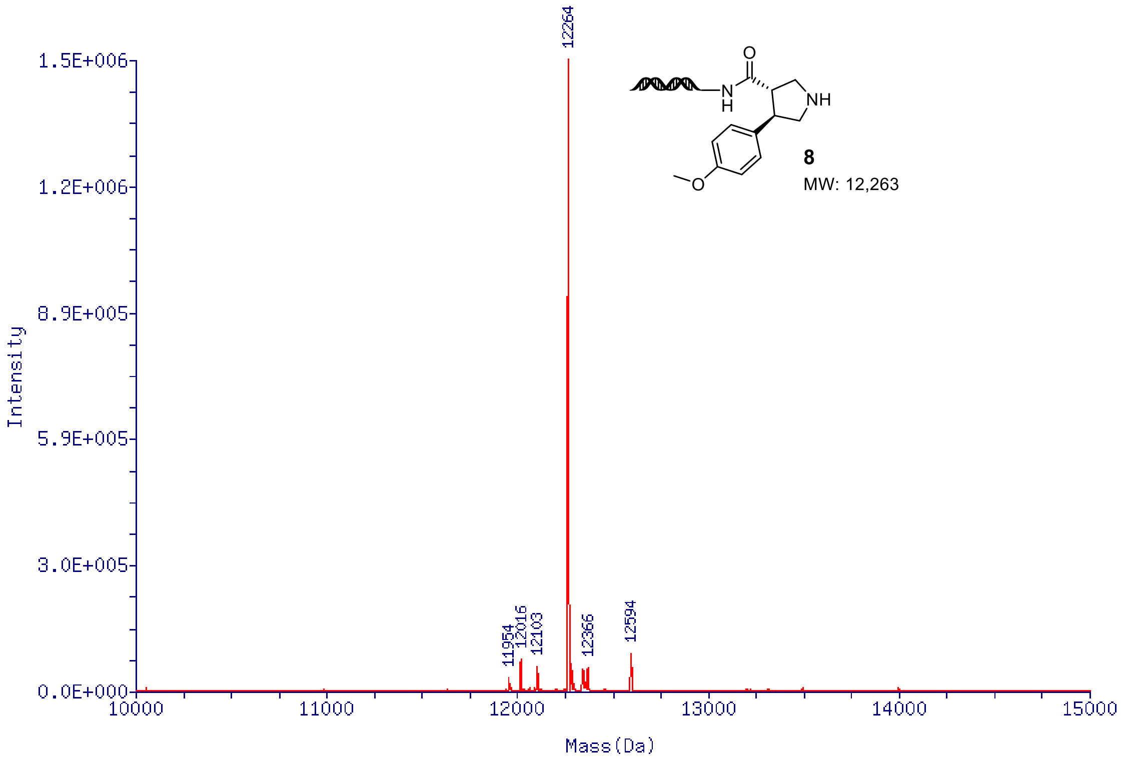

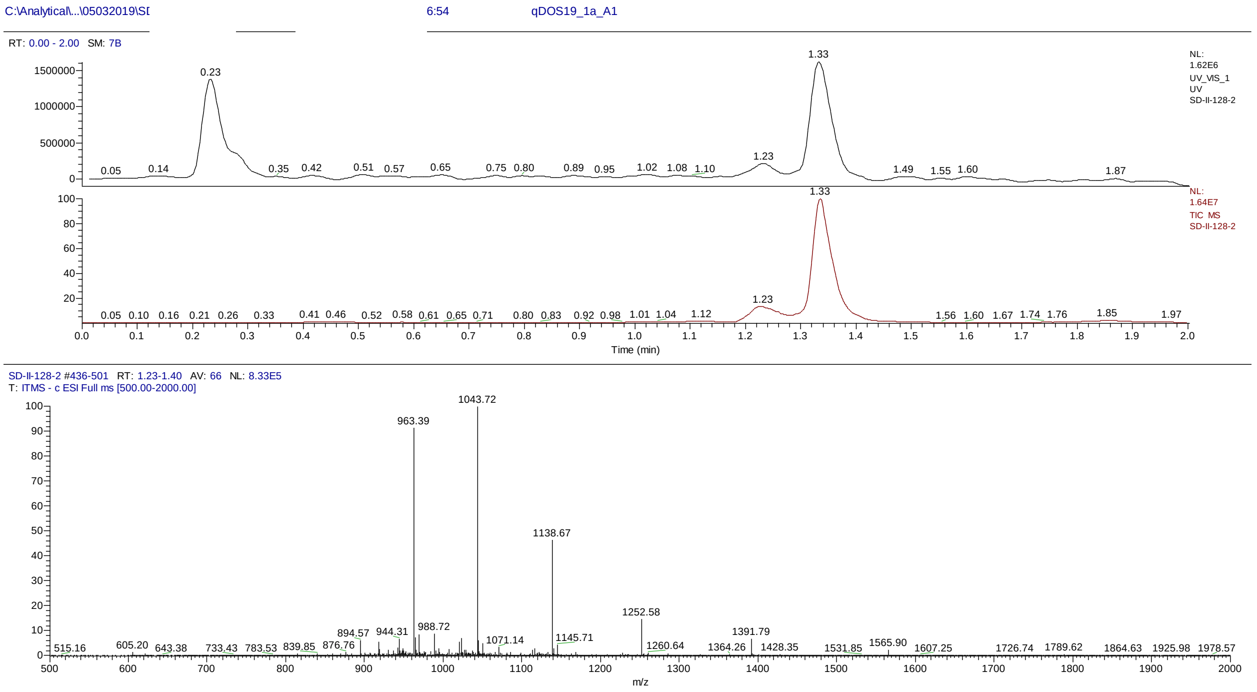
C:\Analytical\...\05032019\SI
6:54
qDOS19_1a_A1
RT: 0.00 - 2.00 SM: 7B
NL: 1.62E6 UV_VIS_1 UV SD-II-128-2
NL: 1.64E7 TIC MS SD-II-128-2
Time (min)
SD-II-128-2 #436-501 RT: 1.23-1.40 AV: 66 NL: 8.33E5
T: ITMS - c ESI Full ms [500.00-2000.00]
963.39
1043.72
1138.67
1252.58
1391.79
m/z

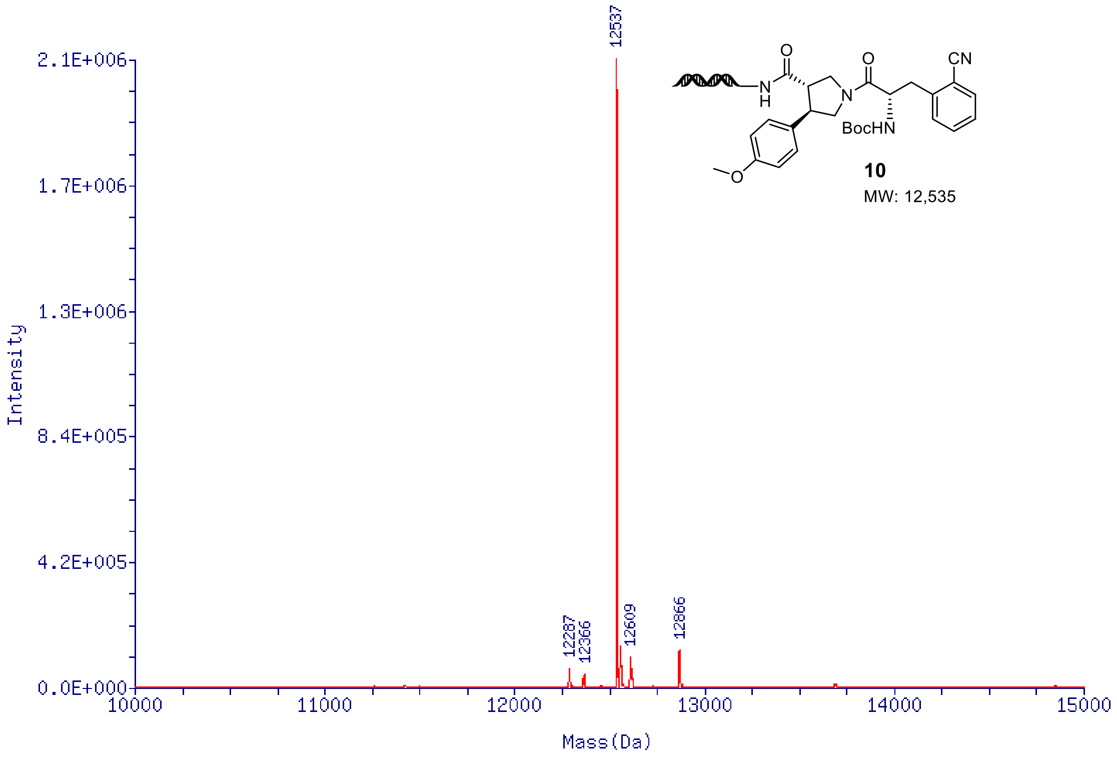
Intensity
Mass(Da)
12537
12287
12366
12609
12866
BocHN
CN
10
MW: 12,535

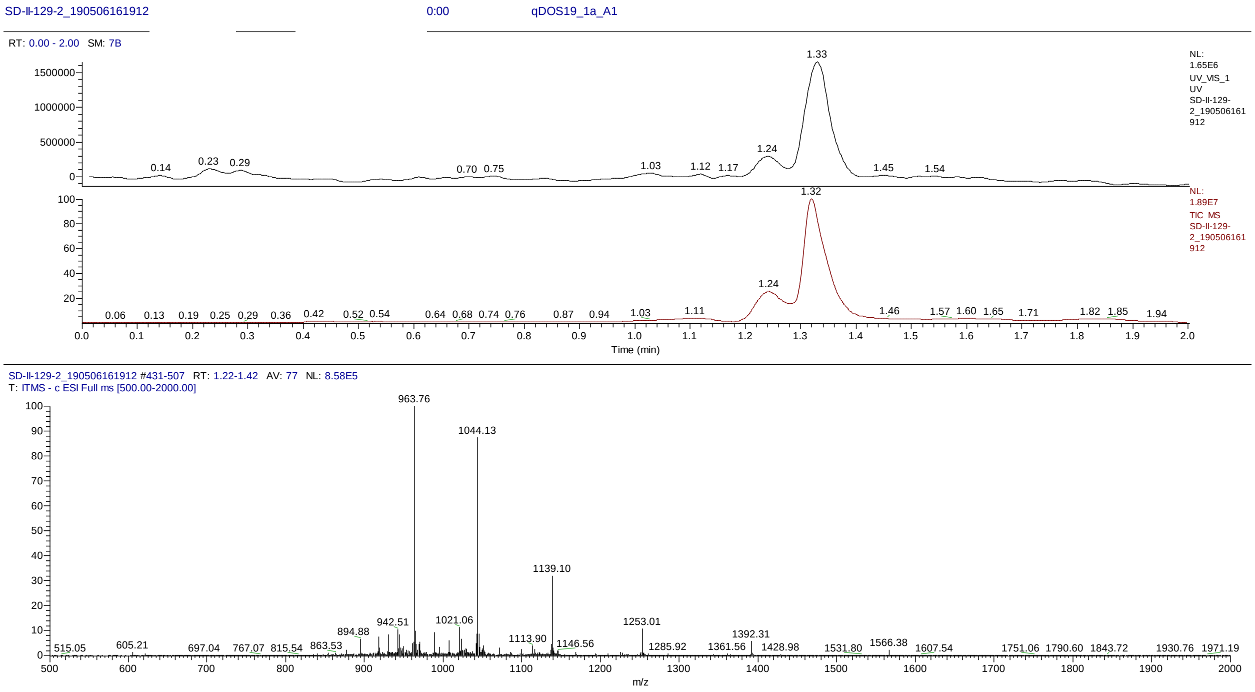
SD-II-129-2_190506161912
0:00
qDOS19_1a_A1
RT: 0.00 - 2.00 SM: 7B
NL: 1.65E6 UV_VIS_1 UV SD-II-129-2_190506161912
NL: 1.89E7 TIC MS SD-II-129-2_190506161912
1.33
1.32
1.24
Time (min)
SD-II-129-2_190506161912 #431-507 RT: 1.22-1.42 AV: 77 NL: 8.58E5
T: ITMS - c ESI Full ms [500.00-2000.00]
963.76
1044.13
1139.10
1253.01
m/z

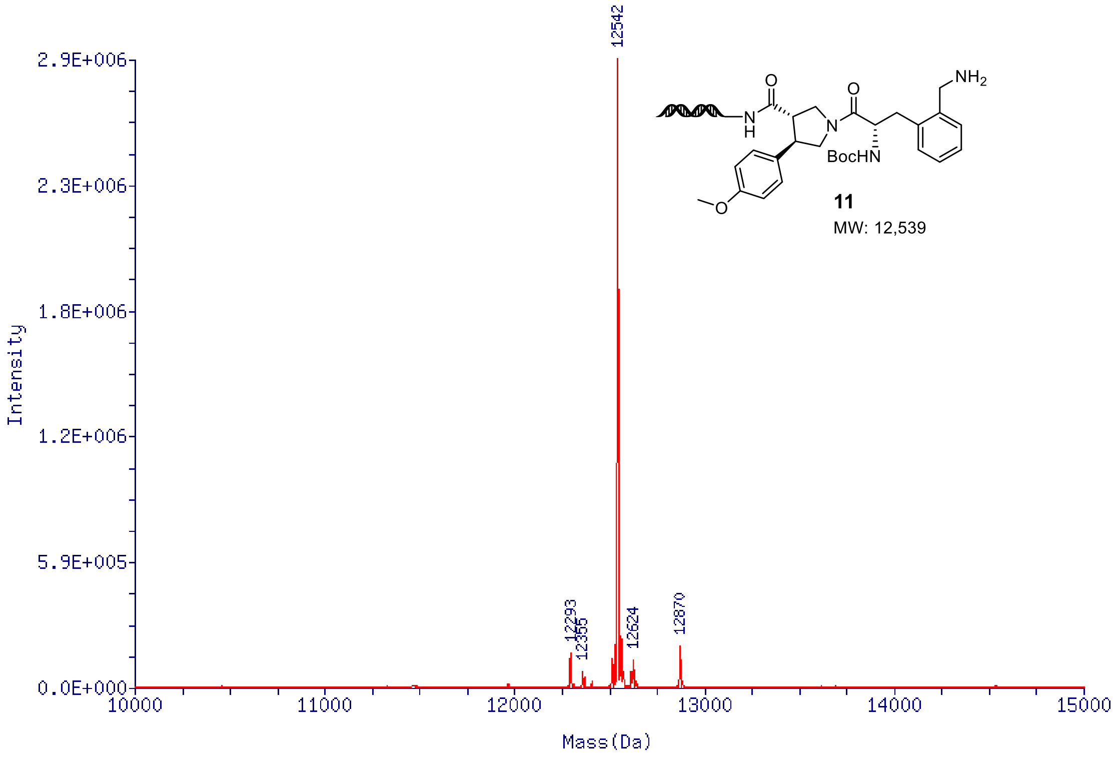
12542
12293
12355
12624
12870
Intensity
Mass(Da)
NH2
O
N
H
BocHN
11
MW: 12,539

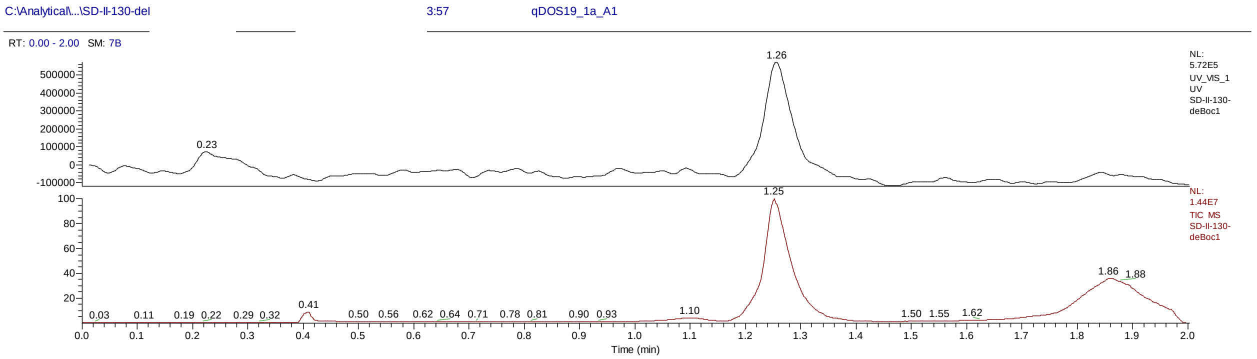
C:\Analytical\...\SD-II-130-del
3:57
qDOS19_1a_A1
RT: 0.00 - 2.00 SM: 7B
NL: 5.72E5 UV_VIS_1 UV SD-II-130-deBoc1
NL: 1.44E7 TIC MS SD-II-130-deBoc1
1.26
1.25
1.86
1.88
Time (min)

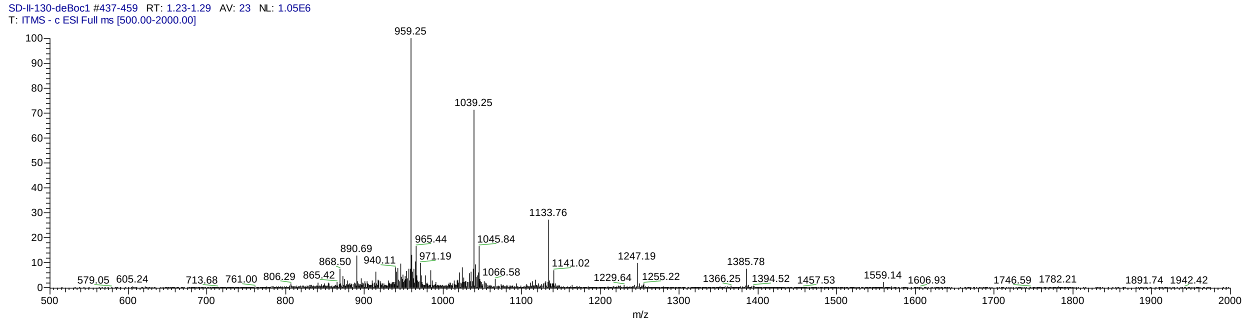
SD-II-130-deBoc1 #437-459 RT: 1.23-1.29 AV: 23 NL: 1.05E6
T: ITMS - c ESI Full ms [500.00-2000.00]
959.25
1039.25
1133.76
m/z

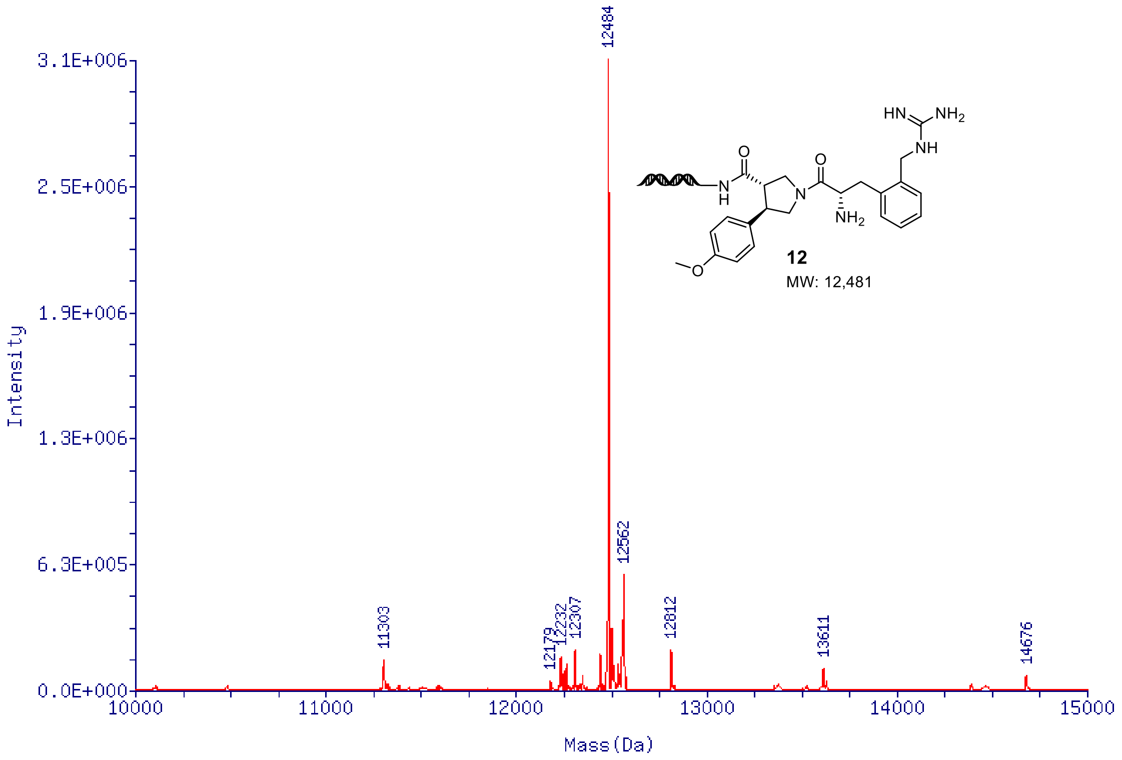
12484
12562
11303
12812
13611
14676
Intensity
Mass(Da)
12
MW: 12,481

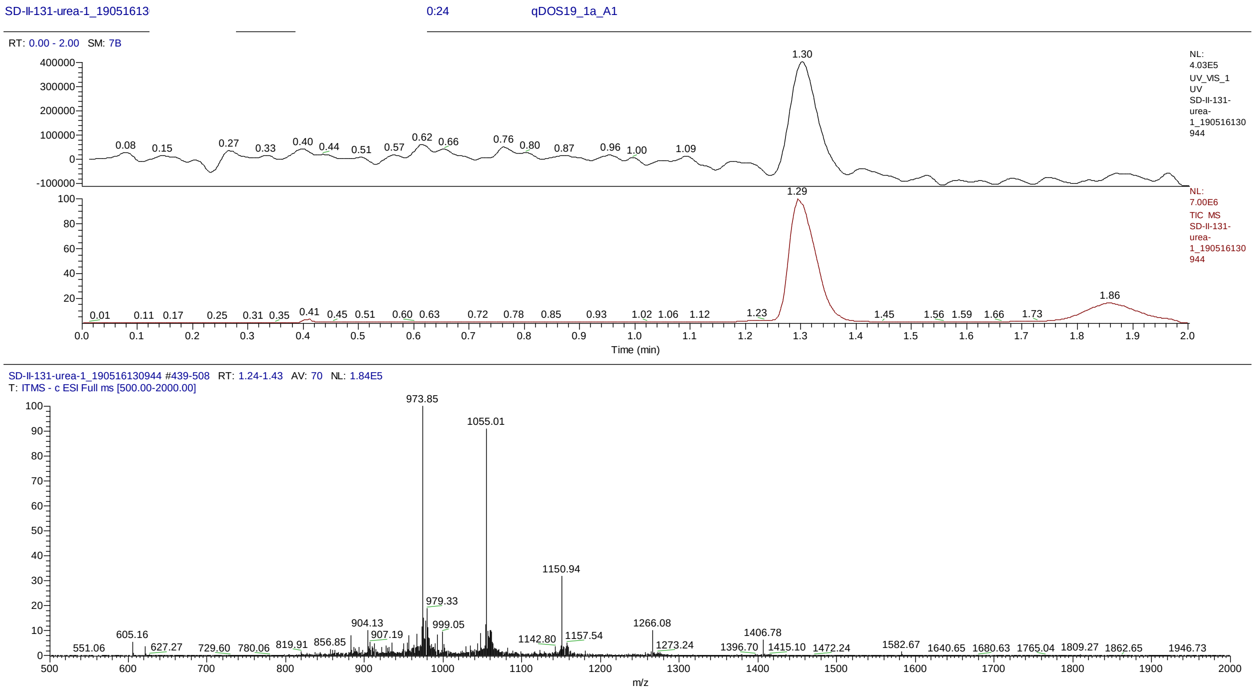
SD-II-131-urea-1_19051613
0:24
qDOS19_1a_A1
RT: 0.00 - 2.00 SM: 7B
NL: 4.03E5 UV_VIS_1 UV SD-II-131-urea-1_190516130944
NL: 7.00E6 TIC MS SD-II-131-urea-1_190516130944
Time (min)
SD-II-131-urea-1_190516130944 #439-508 RT: 1.24-1.43 AV: 70 NL: 1.84E5
T: ITMS - c ESI Full ms [500.00-2000.00]
973.85
1055.01
1150.94
1266.08
1406.78
m/z

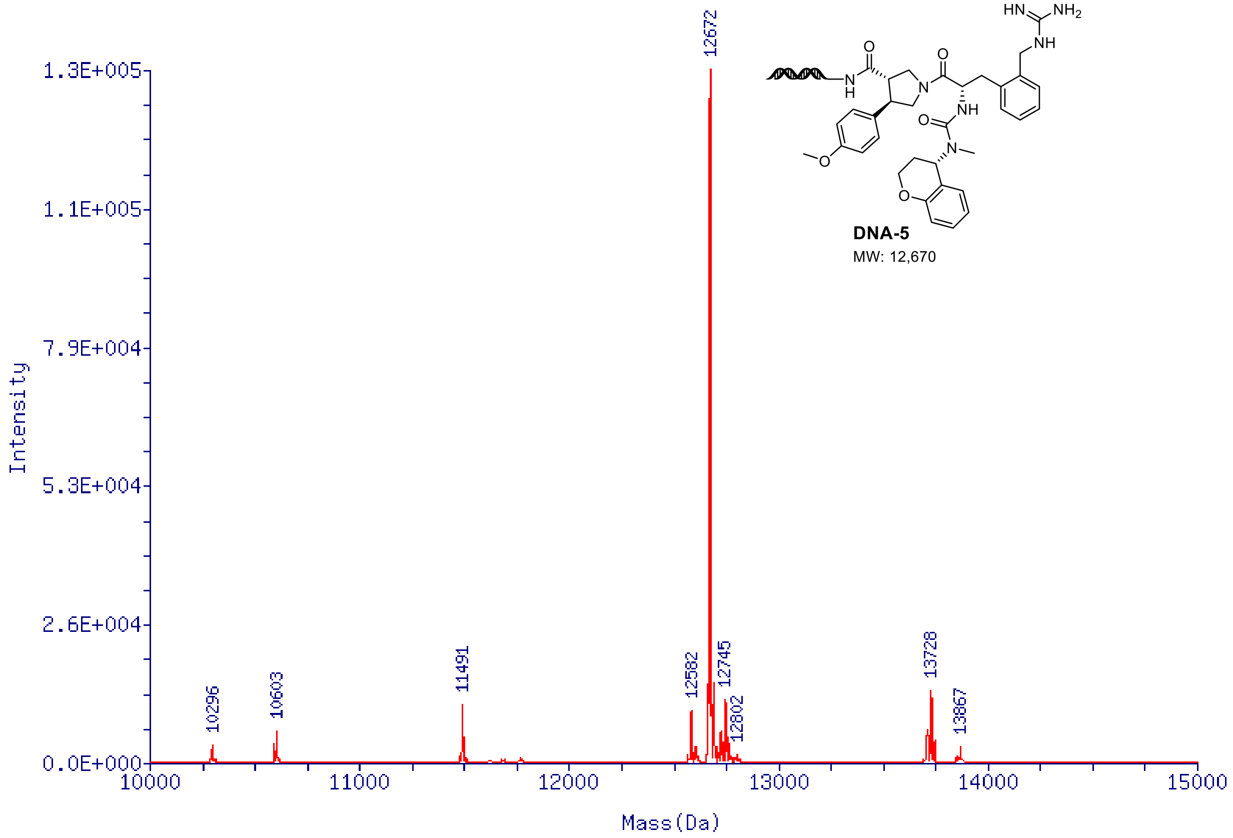
12672
10296
10603
11491
12582
12745
12802
13728
13867
Intensity
Mass(Da)
HN
NH2
NH
O
N
H
NH
O
DNA-5
MW: 12,670

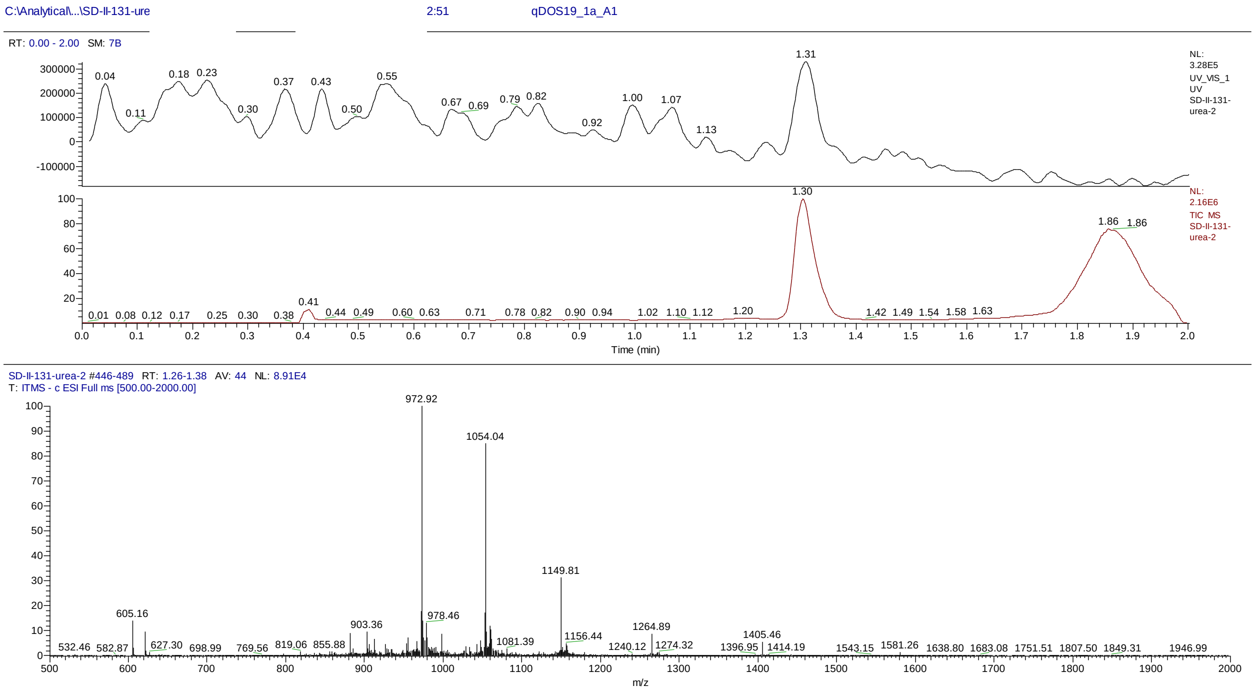
C:\Analytical\...\SD-II-131-ure
2:51
qDOS19_1a_A1
RT: 0.00 - 2.00 SM: 7B
NL: 3.28E5 UV_VIS_1 UV SD-II-131-urea-2
NL: 2.16E6 TIC MS SD-II-131-urea-2
Time (min)
SD-II-131-urea-2 #446-489 RT: 1.26-1.38 AV: 44 NL: 8.91E4
T: ITMS - c ESI Full ms [500.00-2000.00]
972.92
1054.04
1149.81
605.16
903.36
978.46
1264.89
1405.46
m/z

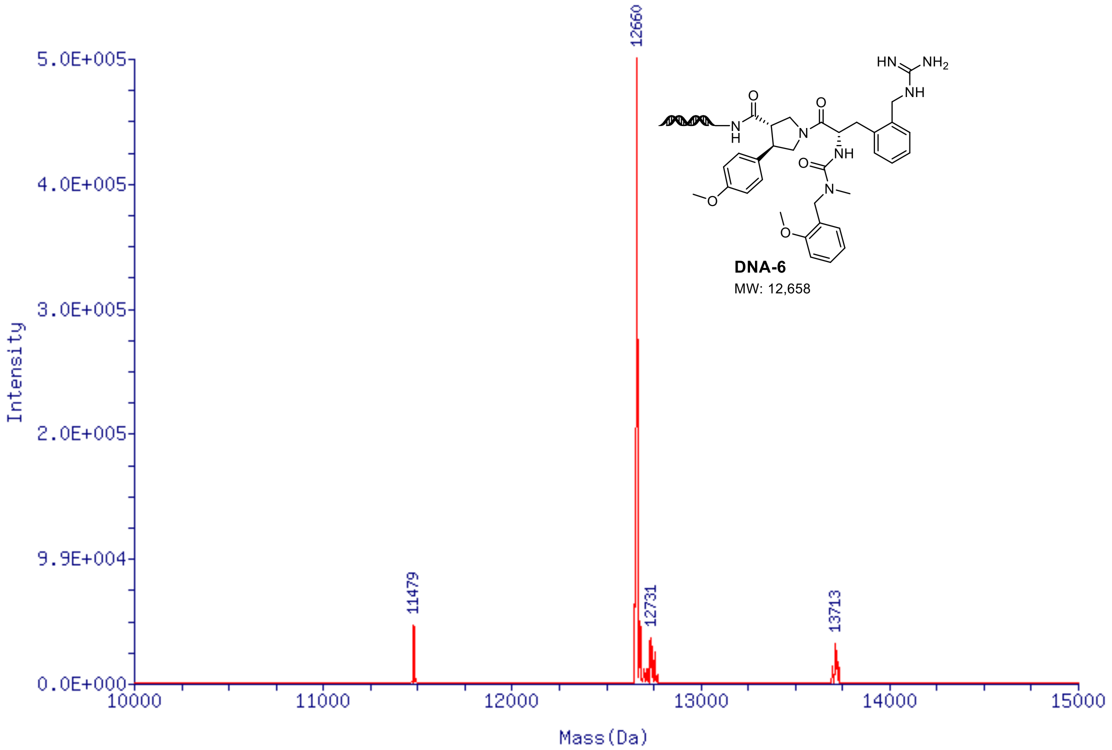
12660
11479
12731
13713
Intensity
Mass(Da)
DNA-6
MW: 12,658

7f) ^{1}H and ^{13}C NMR spectra

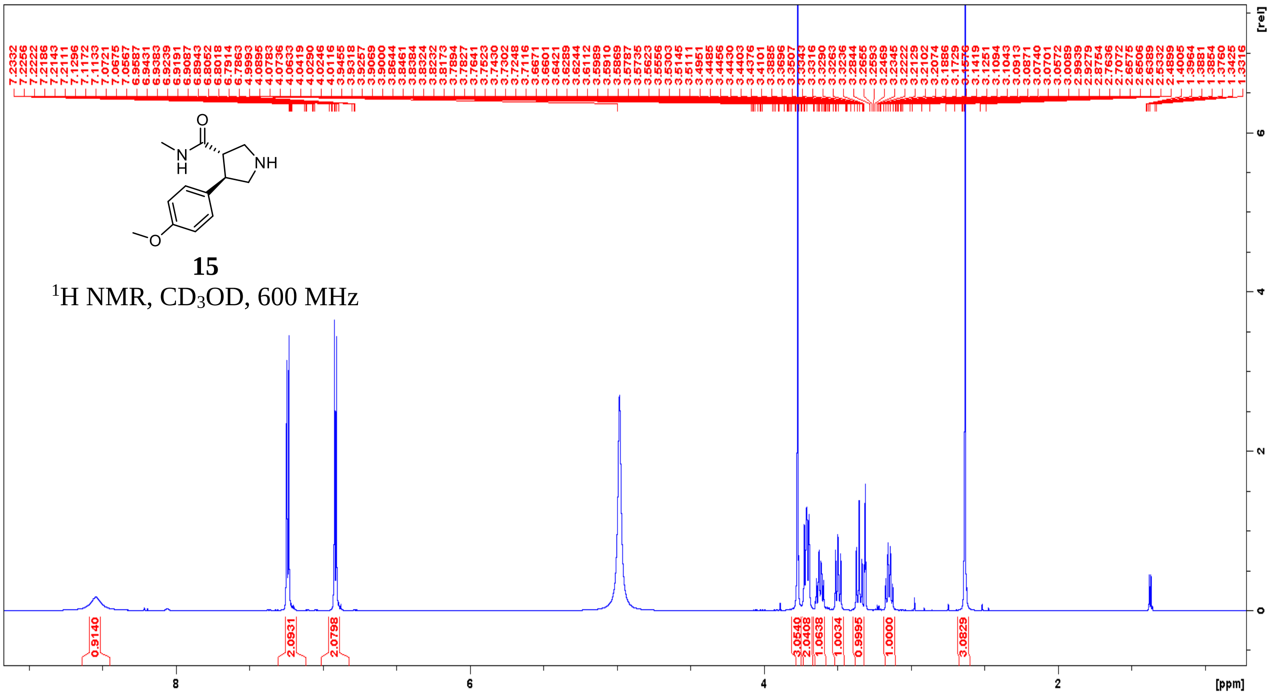

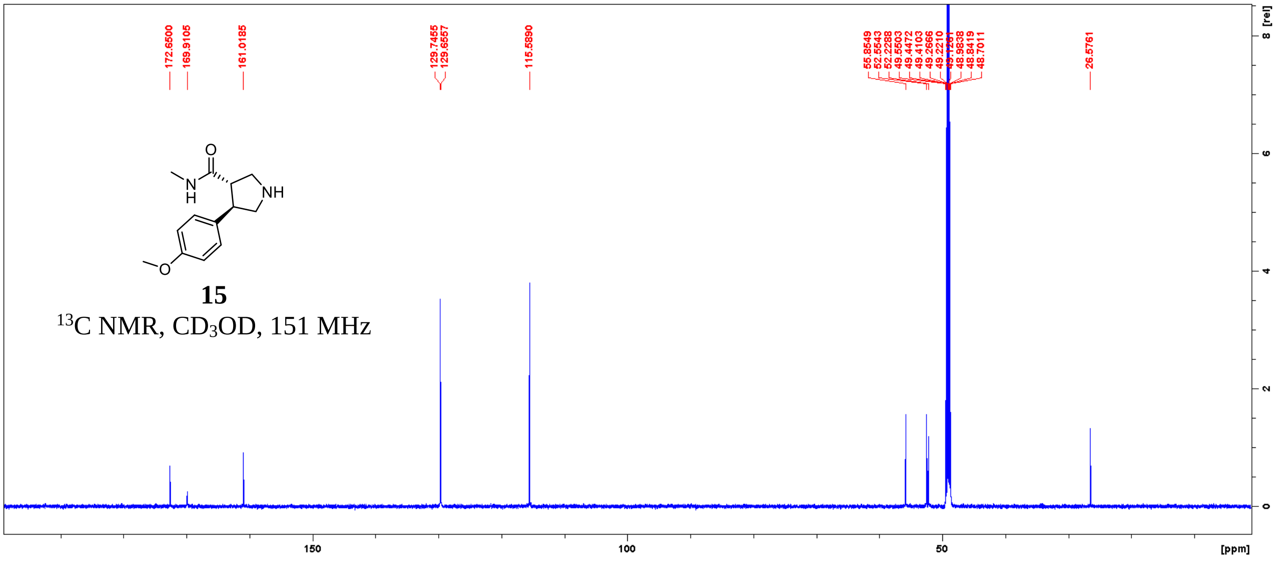

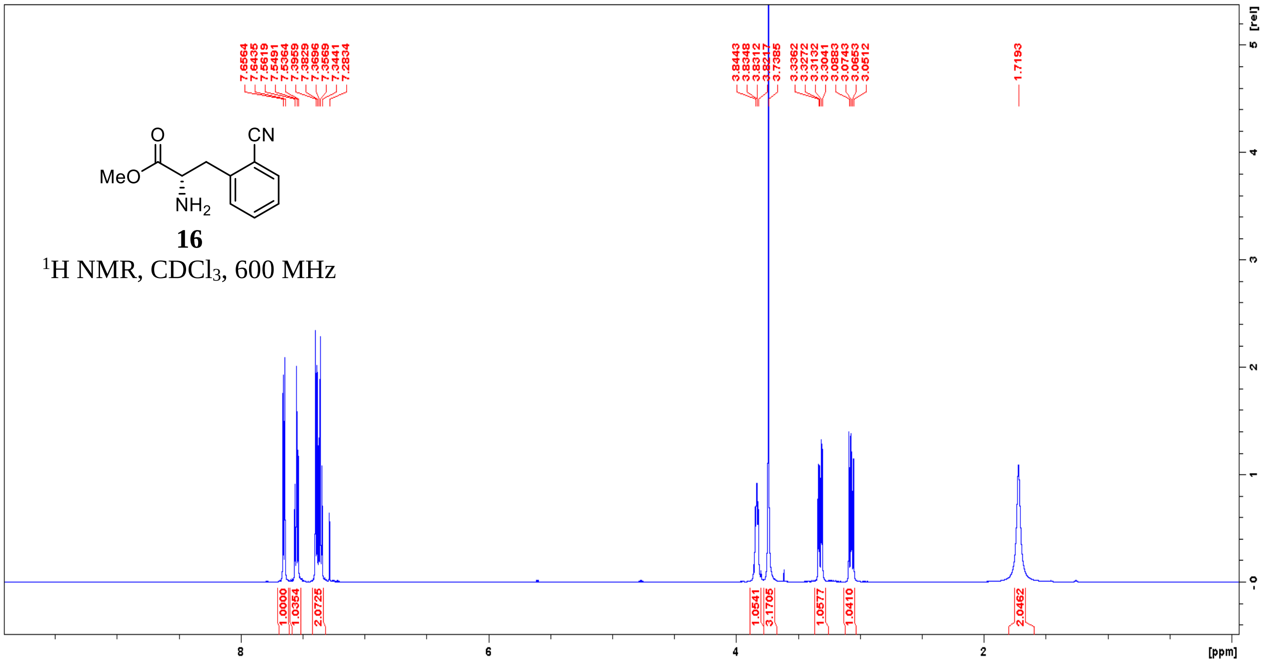
16
^{1}H NMR, $CDCl_3$, 600 MHz

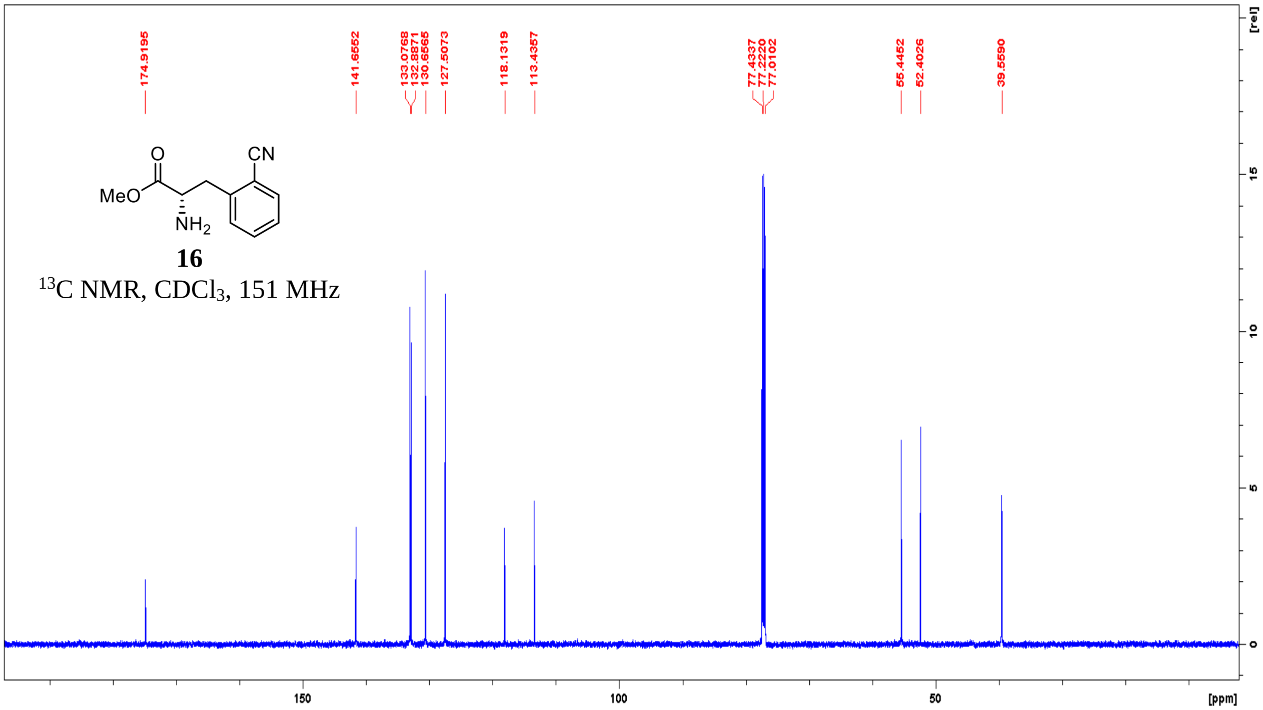
16
^{13}C NMR, $CDCl_3$, 151 MHz

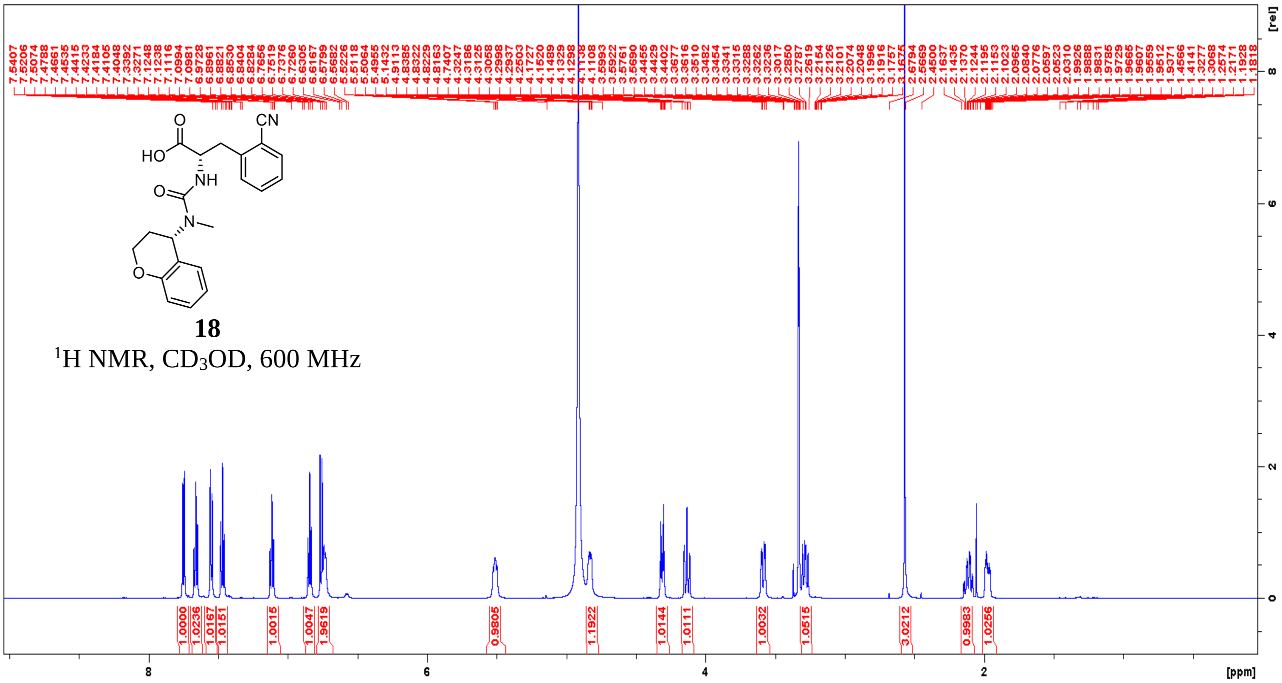
CN
HO
O
NH
O
N
O
18
¹H NMR, CD₃OD, 600 MHz
[rel]
[ppm]

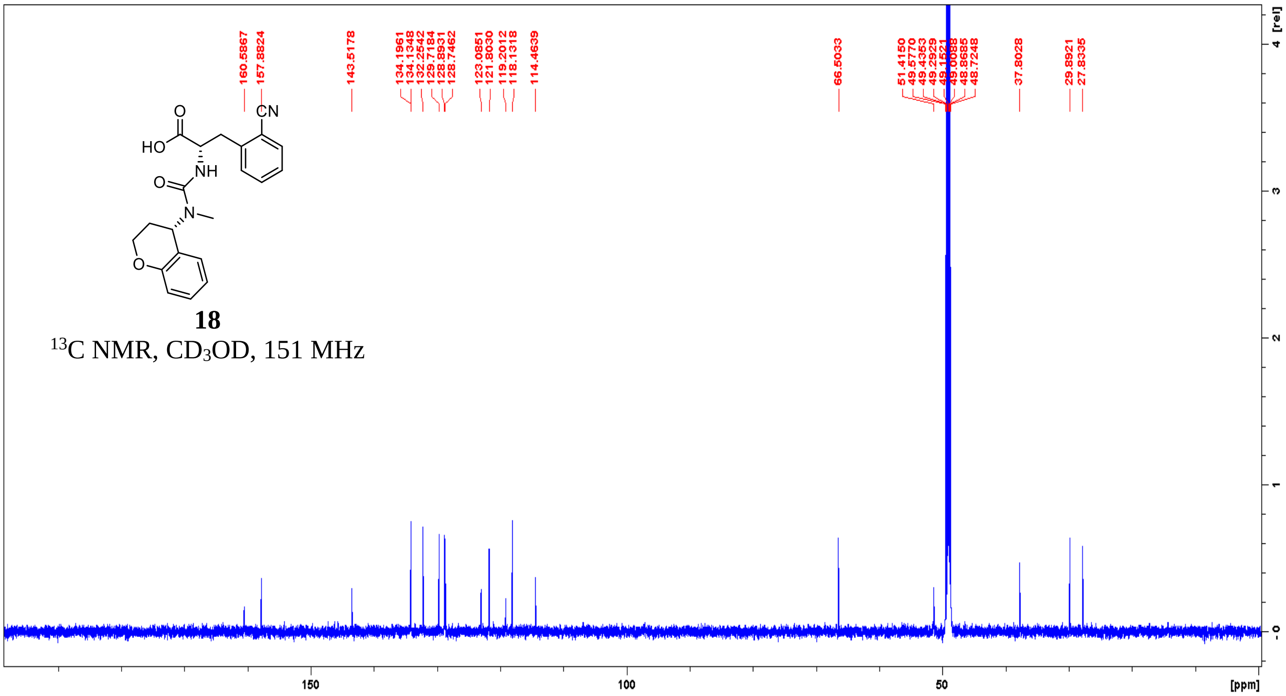
160.5867
157.8824
143.5178
134.1961
134.1348
132.2542
129.7184
128.8931
128.7462
123.0851
121.8030
119.2012
118.1318
114.4639
66.5033
51.4150
49.5770
49.4353
49.2929
49.1521
49.0088
48.8685
48.7248
37.8028
29.8921
27.8335
CN
HO
O
NH
O
N
O
18
¹³C NMR, CD₃OD, 151 MHz
[rel]
[ppm]

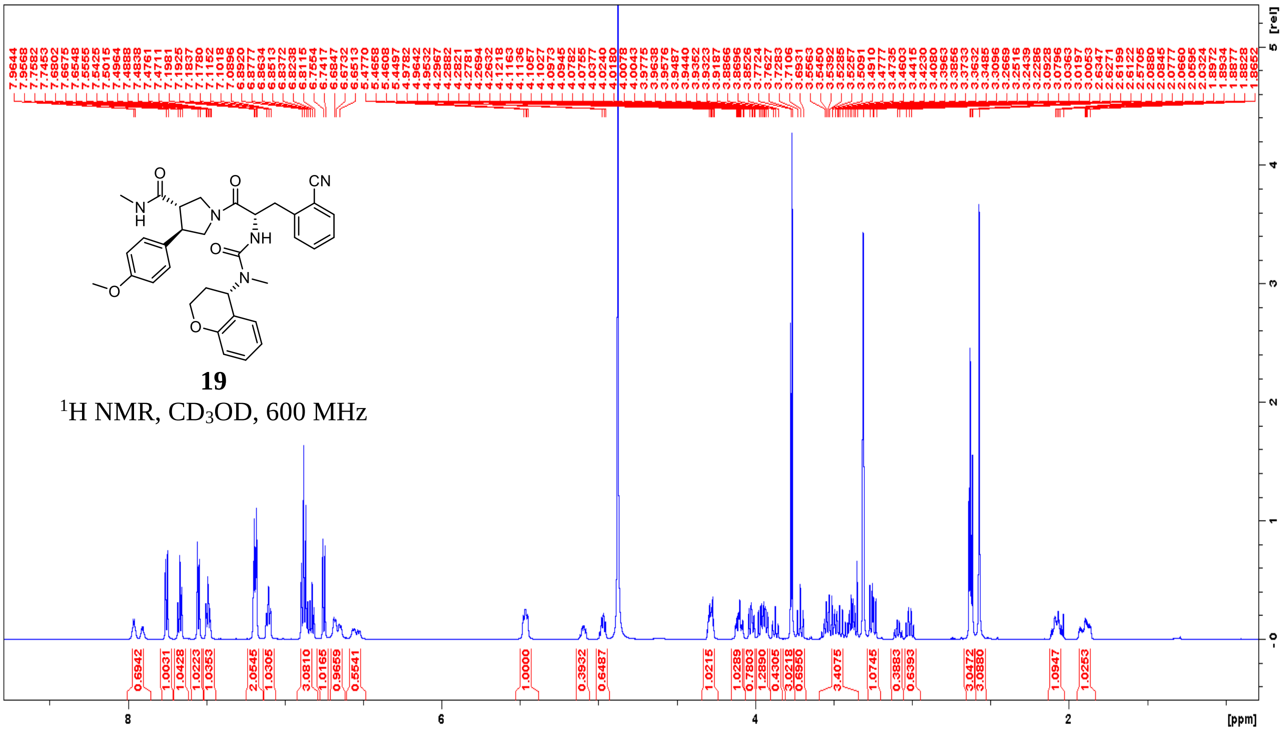
19
1H NMR, CD3OD, 600 MHz

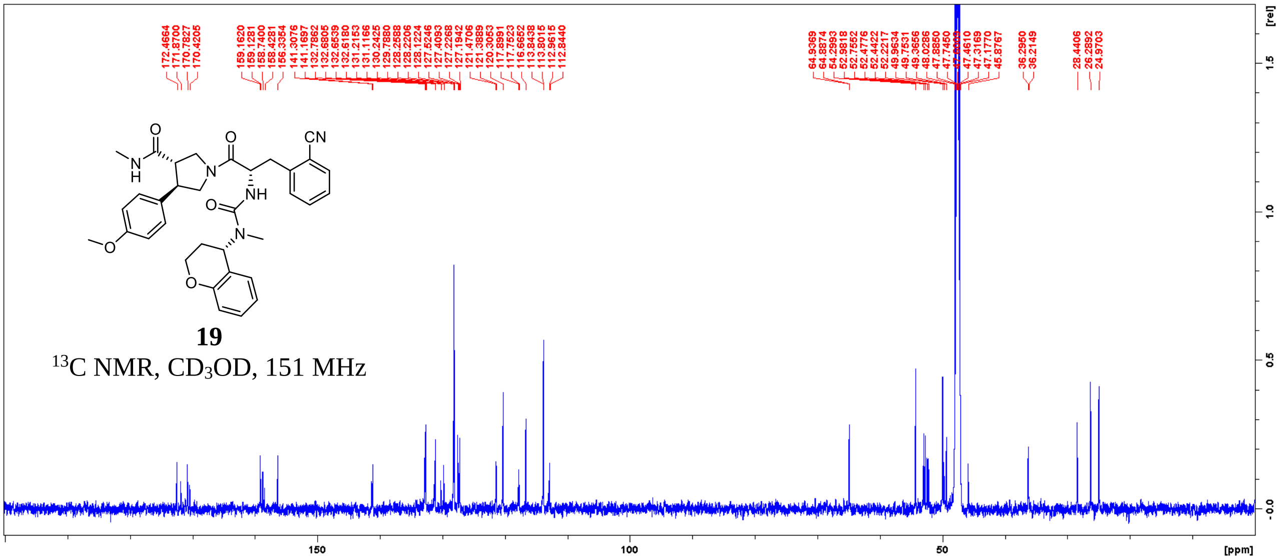
19
13C NMR, CD3OD, 151 MHz

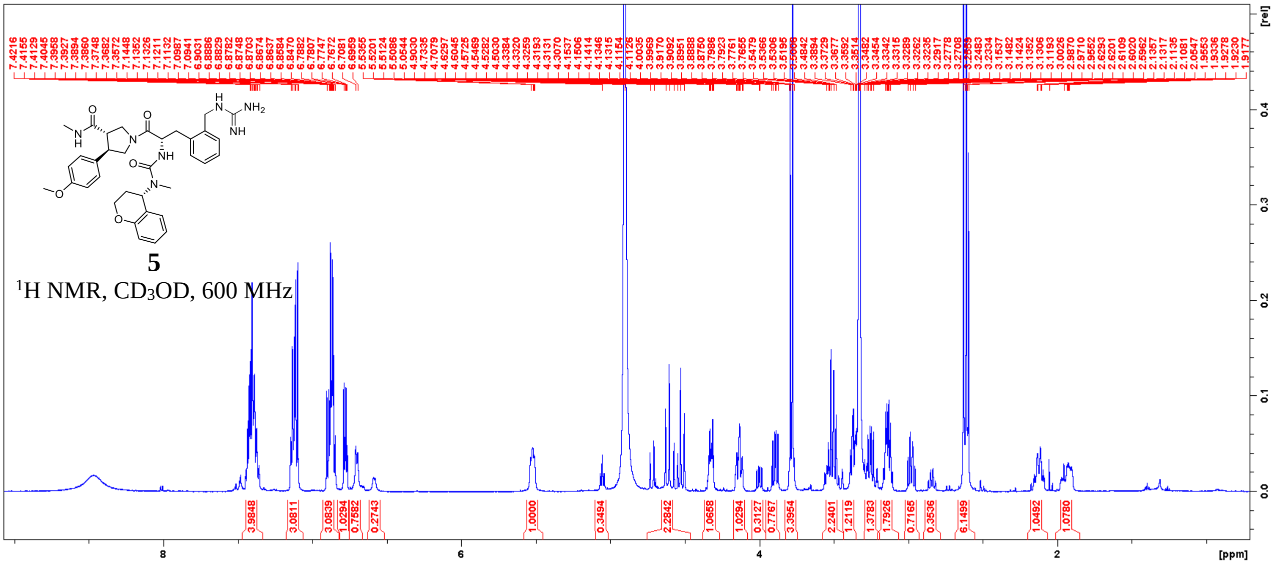
5
^{1}H NMR, CD$_3$OD, 600 MHz

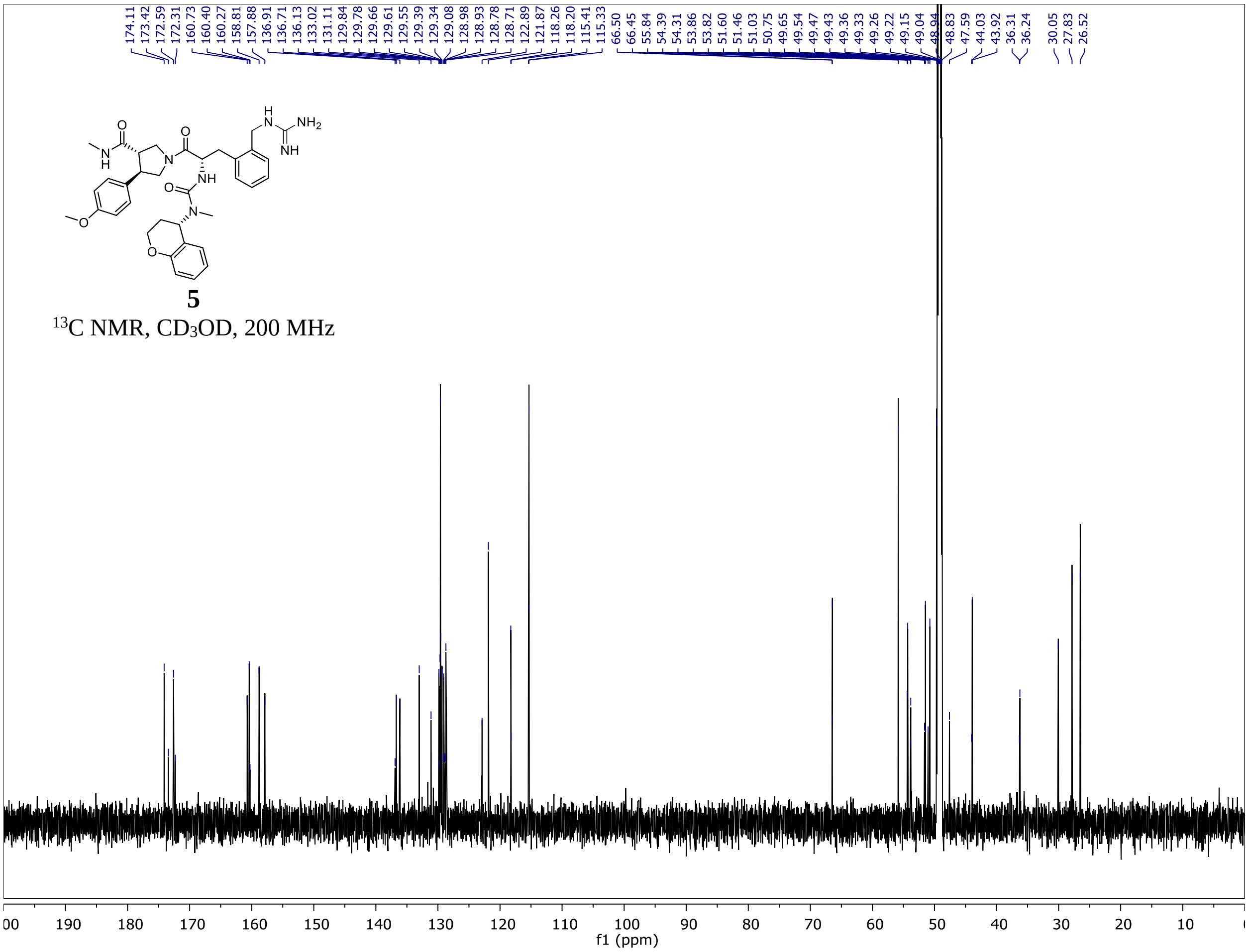
5
^{13}C NMR, CD$_3$OD, 200 MHz
174.11
173.42
172.59
172.31
160.73
160.40
160.27
158.81
157.88
136.91
136.71
136.13
133.02
131.11
129.84
129.78
129.66
129.61
129.55
129.39
129.34
129.08
128.98
128.93
128.78
128.71
122.89
121.87
118.26
118.20
115.41
115.33
66.50
66.45
55.84
54.39
54.31
53.86
53.82
51.60
51.46
51.03
50.75
49.65
49.54
49.47
49.43
49.36
49.33
49.26
49.22
49.15
49.04
48.94
48.83
47.59
44.03
43.92
36.31
36.24
30.05
27.83
26.52
f1 (ppm)

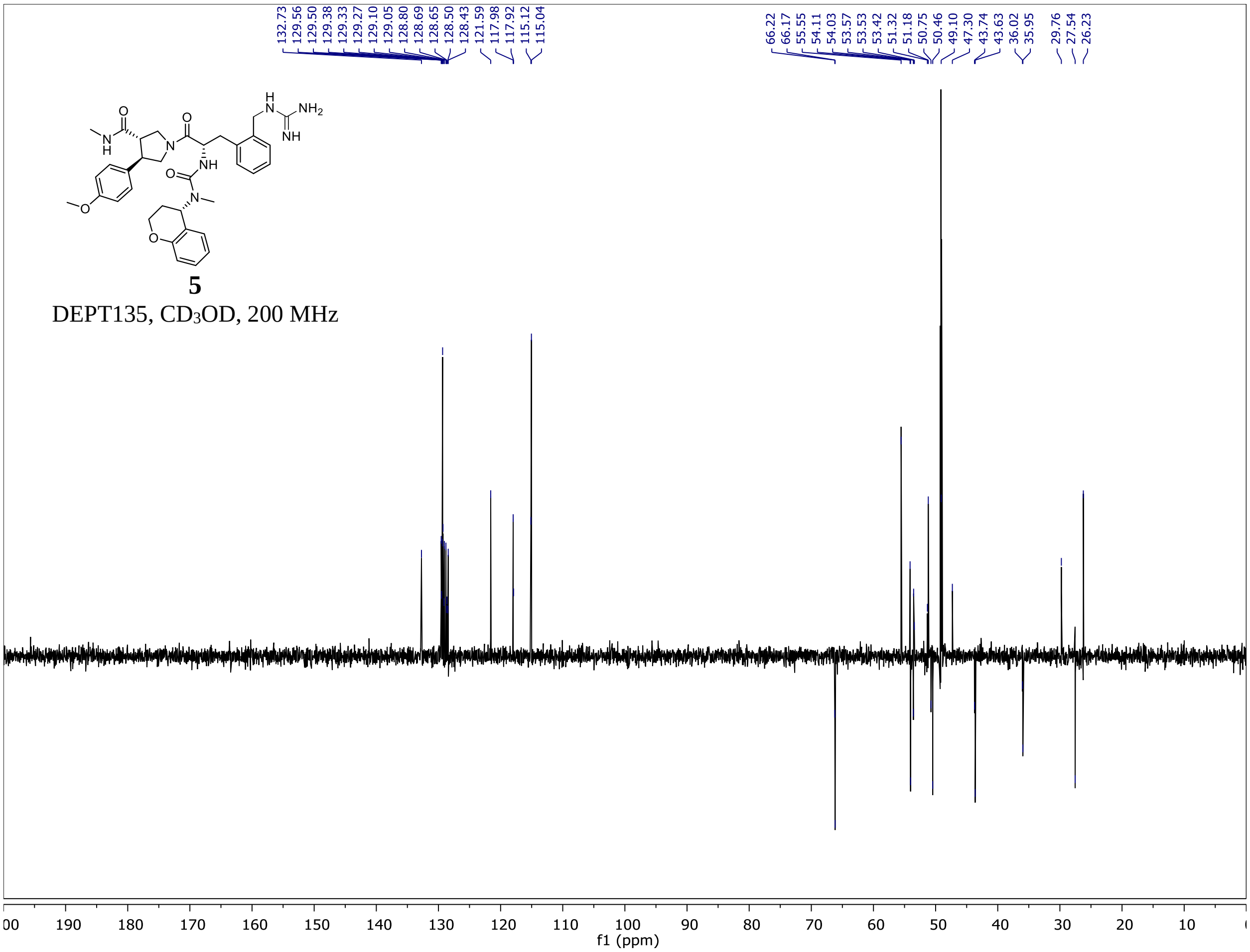
132.73
129.56
129.50
129.38
129.33
129.27
129.10
129.05
128.80
128.69
128.65
128.50
128.43
121.59
117.98
117.92
115.12
115.04
66.22
66.17
55.55
54.11
54.03
53.57
53.53
53.42
51.32
51.18
50.75
50.46
49.10
47.30
43.74
43.63
36.02
35.95
29.76
27.54
26.23
5
DEPT135, CD3OD, 200 MHz
f1 (ppm)
